# Supplementary material for: Metallaphotocatalytic triple couplings for modular synthesis of elaborate N-trifluoroalkyl anilines
Source: Nat Commun. 2024 Nov 15;15:9926. doi: 10.1038/s41467-024-53828-8 (PMC11568185; doi:10.1038/s41467-024-53828-8)
Supplement: Supplementary file 1 — Supplementary Information [file 41467_2024_53828_MOESM1_ESM.pdf]

# Supplementary Information

## Metallaphotocatalytic triple couplings for modular synthesis of elaborate *N*-trifluoroalkyl anilines

Ting Zhou[1], Zhong-Wei Zhang[1], Jing Nie[1], Fuk Yee Kwong[2], Jun-An Ma[1]*, Chi Wai Cheung[1,2]*

[1]Department of Chemistry, Tianjin Key Laboratory of Molecular Optoelectronic Sciences, Frontiers Science Center for Synthetic Biology (Ministry of Education), Tianjin University, Tianjin 300072, P. R. of China

[2]State Key Laboratory of Synthetic Chemistry and Department of Chemistry, The Chinese University of Hong Kong, Shatin, New Territories, Hong Kong 999077, P. R. of China

*Corresponding E-mails: majun_an68@tju.edu.cn (J.-A.M.); cw.cheung@cuhk.edu.hk (C.W.C.)

# Table of contents


| | |
|---|---|
| General Considerations | S3 |
| General Analytical Information | S3 |
| General Reagent Information | S3 |
| General Manipulation Considerations | S4 |
| Supplementary Results | S5 |
| Synthesis of starting materials | S5 |
| General method for the synthesis of 1-aryloxy-4-nitroarenes (**General Procedure A**) | S5 |
| General method for the synthesis of redox active esters (**General Procedure B**) | S6 |
| Optimization of reaction conditions (**Tables S1 and S2** and **Fig. S1**) | S8 |
| Mechanistic study | S14 |
| **(a)** Radical clock experiment (**Fig. S2**) | S14 |
| **(b)** Radical trap experiment (**Fig. S3**) | S16 |
| **(c)** Analysis of reaction co-products in the early reaction stage (**Fig. S4**) | S21 |
| **(d)** Probing the viable nitrogen-containing intermediates (**Fig. S5**) | S25 |
| **(e)** Study of *N*-aryl imine and *N*-methyl aniline as reaction intermediates for product formation (**Fig. S6**) | S27 |
| **(f)** Investigation of the $\alpha$-hydrogen source in *N*-trifluoroalkyl aniline product (**Fig. S7**) | S30 |
| **(g)** Light/dark Experiments (**Fig. S8**) | S34 |
| **(h)** Study on defluorination side-reaction (**Fig. S9**) | S36 |
| **(i)** Cyclic voltammetry of 1-benzyloxy-4-nitrobenzene (**N14**) in MeCN (**Fig. S10**) | S39 |
| Substrate scope study and gram scale synthesis of **14** | S40 |
| Diverse Functionalization of the *N*-trifluoroalkyl aniline products | S102 |
| Stern-Volmer quenching studies (**Figs. S11–S16**) | S111 |

| | |
|---|---|
| X-ray crystallographic analysis | S115 |
| NMR Spectra | S117 |
| References | S355 |

# General Considerations

**General Analytical Information.** $^{1}H$, $^{13}C$ and $^{19}F$ NMR spectra were recorded on Bruker AV 400 MHz instrument at 400 MHz ($^{1}H$ NMR), 101 MHz ($^{13}C$ NMR), and 376 MHz ($^{19}F$ NMR, comp. pulse decoupling), or on Bruker AV 500 MHz instrument at 500 MHz ($^{1}H$ NMR), 125 MHz ($^{13}C$ NMR), and 470 MHz ($^{19}F$ NMR, comp. pulse decoupling), or on *J*EOL *J*NM ECZ600R instrument at 600 MHz ($^{1}H$ NMR), 151 MHz ($^{13}C$ NMR), and 565 MHz ($^{19}F$ NMR, comp. pulse decoupling), or on Bruker AV 800 MHz instrument at 800 MHz ($^{1}H$ NMR), 201 MHz ($^{13}C$ NMR), and 753 MHz ($^{19}F$ NMR, comp. pulse decoupling). All $^{1}H$ NMR spectra were measured in parts per million (ppm) downfield from tetramethylsilane (TMS, 0 ppm), or were measured relative to the residual proton signals of $d_1$-chloroform ($CDCl_3$, 7.26 ppm) or methanol-$d_4$ ($CD_3OD$-$d_4$, 3.31 ppm). All $^{13}C$ NMR spectra were reported in ppm relative to residual carbon signals of $CDCl_3$ (77.16 ppm) or $CD_3OD$-$d_4$ (49.00 ppm) and were obtained with $^{1}H$ decoupling.[1] All $^{19}F$ NMR spectra were measured in parts per million (ppm) relative to trichlorofluoromethane ($CFCl_3$, 0 ppm). Coupling constants (*J*) are reported in hertz (Hz). Multiplicity was indicated as follows: s (singlet), d (doublet), t (triplet), q (quartet), p (pentet), and m (multiplet). High resolution mass spectrometry (HRMS) spectra were obtained on a Bruker micrOTOF-QII instrument. GC-MS analyses were performed on a Thermo Scientific Model Trace 1300 instrument. X-ray structural analysis was conducted on a Bruker APEX-II CCD instrument. Thin-layer chromatography (TLC) was performed on precoated GF254 silica gel plates (Qingdao Marine Chemical Inc.) and compounds were visualized with a UV light at 254 nm. Flash chromatography for purification of compounds were carried out using silica gel (200–300 mesh, Qingdao Marine Chemical Inc.). Stern-Volmer quenching experiments were performed using Edinburgh photofluorescence spectrometer FLS1000. Cyclic Voltammetry was conducted using Shanghai Chenhua CHI 760E Electrochemical Workstation.

**General Reagent Information.** Unless otherwise noted, commercially available materials were used without prior purification. All known starting materials were synthesized according to the literature procedures. Anhydrous Dimethyl sulfoxide (DMSO) was purchased from Energy Chemical and it was added with anhydrous 3Å

molecular sieves for storage. Anhydrous 1,2-Dimethoxyethane (DME) was purchased from Energy Chemical and it was added with anhydrous 3Å molecular sieves for storage.(4,4'-Di-*tert*-butyl-2,2'-bipyridine)bis-[3,5-difluoro-2-[5-trifluoromethyl-2-pyridinyl-κ*N*)phenyl-κ*C*]iridium(III) hexafluoro-phosphate ($Ir[dF(CF_3)ppy]_2(dtbbpy)PF_6$, **PC1**, 98% purity) was purchased from Bidepharm and was stored in refrigerator for storage. Nickel(II) nitrate hexahydrate ($Ni(NO_3)_2$•$6H_2O$, 98% purity) and 4,7-diphenyl-1,10-phenanthroline (bathophenanthroline, BPhen, **L1**, 99% purity) were purchased from Aladdin. Diethyl 1,4-dihydro-2,6-dimethyl-3,5-pyridinedicarboxylate (Hantzsch ester, **HE**, 98% purity), trifluoromethanesulfonic acid (**HOTf**, 99% purity) and *N*,*N*-dimethylcyclohexylamine (99.5% purity) were purchased from HEOWNS. Triethylamine (**$Et_3N$**, 99.5% purity) was purchased from Aladdin. 3,3,3,-Trifluoropropene (**F1**) was purchased from Shang Fluoro.

**General Manipulation Considerations.** Unless otherwise noted, all manipulations for photochemical reaction were performed in Teflon screw-capped Schlenk tubes. Flash chromatography for purification of compounds were carried out using silica gel (200–300 mesh, Qingdao Marine Chemical Inc.). Preparative thin-layer chromatography (PTLC) for purification of compounds were carried out using preparative TLC (Rushan Hailan Experimental Equipment Inc.). Thin-layer chromatography (TLC) was performed on precoated GF254 silica gel plates (Qingdao Marine Chemical Inc.) and compounds were visualized with a UV light at 254 nm. The eluents used for column chromatography, PTLC and TLC were presented as ratios of solvent volumes (v/v). Ethyl acetate (EtOAc) was used as the extraction solvent to extract the products attached on the silica gel of PTLC plate. Yields reported in the publication are of isolated yields unless otherwise noted. All new starting materials and all products obtained from the photochemical reactions were characterized by $^{1}H$, $^{13}C$ and $^{19}F$ NMR spectroscopies (in case the compounds contained F atoms) and high-resolution mass spectrometry (HRMS). The blue LEDs (456–460 nm, 30 W) setup for photocatalytic reactions in 20 mL Schlenk tubes were purchased from Taobao.

# Supplementary Results

## Synthesis of starting materials

### General method for the synthesis of 1-aryloxy-4-nitroarenes (General Procedures A).[1]

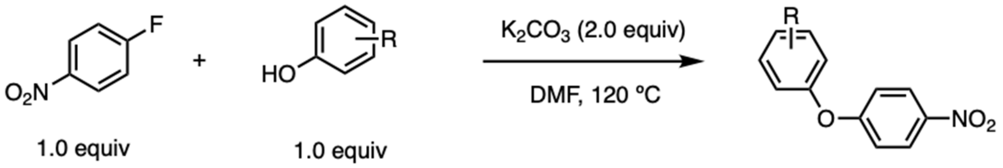


To a Teflon screw-capped 500 mL Schlenk round-bottom flask bottle equipped with a magnetic stir bar was charged with the 1-fluoro-4-nitrobenzene (1.0 equiv., 40 mmol), phenol (1.0 equiv., 40 mmol), $K_2CO_3$ (2.0 equiv., 80 mmol) and DMF (130 mL, 0.3 M). The resulting reaction mixture was then stirred under an argon atmosphere at 120 °C in an oil bath. After 18 h, the solution was allowed to cool to room temperature. The reactiom mixture was diluted with $H_2O$ (300 mL) and then extracted with ethyl acetate (3 × 100 mL). The combined organic fraction was washed with aqueous NaOH solution (100 mL, 1M) and brine (100 mL), dried over anhydrous $Na_2SO_4$, filtered and concentrated *in vacuo*. The residue was recrystallized with $CH_2Cl_2$ and petroleum ether to afford the 1-aryloxy-4-nitroarene as the starting material.

**The synthesis of 7-(4-nitrophenoxy)-3,4-dihydronaphthalen-1(2*H*)-one (N31)**

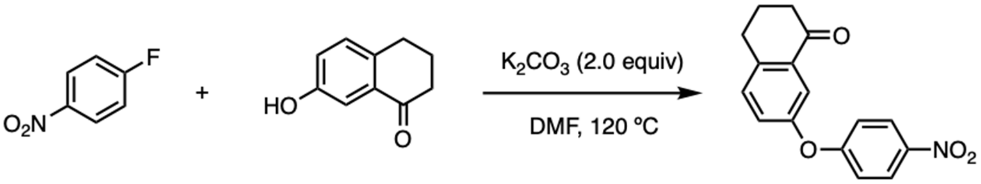


**N31**, 82%

Following the General Procedure A, the title compound **N31** was prepared from 4-fluoronitrobenzene (40 mmol, 4.2 mL), 6-hydroxy-1-tetralone (40 mmol, 6.49 g), $K_2CO_3$ (80 mmol, 11.06 g), and DMF (130 mL) to obtain the title compound as a brown amorphous solid (9.28 g, 82%). **$^1$H NMR** (400 MHz, $CDCl_3$) $\delta$ 8.21 (d, $J$ = 9.2 Hz, 2H), 7.99 (d, $J$ = 7.9 Hz, 1H), 7.39 (t, $J$ = 7.9 Hz, 1H), 7.24 (dd, $J$ = 8.0, 1.3 Hz,

1H), 6.96 (d, *J* = 9.2 Hz, 2H), 2.81 (t, *J* = 6.1 Hz, 2H), 2.66 (t, *J* = 6.0 Hz, 2H), 2.11 (p, *J* = 6.4 Hz, 2H). **$^{13}$C NMR** (101 MHz, $CDCl_3$) $\delta$ 197.4, 162.9, 151.8, 142.9, 136.9, 135.0, 127.9, 126.3, 125.9, 124.8, 116.4, 38.8, 23.3, 22.4. **HRMS** (ESI) m/z: $[M+H]^+$ Calcd for $C_{16}H_{14}NO_4^+$ 284.0923; Found 284.0930.

## General method for the synthesis of redox active esters (RAEs) (General Procedures B).[2]

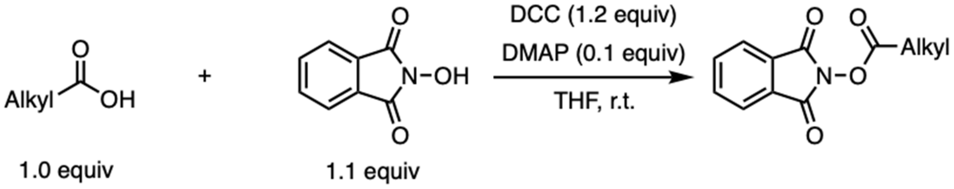


To a 500 mL round-bottom flask equipped with a magnetic stir bar was charged with the alkyl carboxylic acid (1.0 equiv., 20 mmol), *N*,*N*'-dicyclohexylcarbodiimide (DCC, 1.2 equiv., 24 mmol), 4-dimethylaminopyridine (DMAP, 0.1 equiv., 2 mmol), and DCM (50 mL, 0.4 M). The reaction mixture was stirred for 30 minute. *N*-hydroxyphthalimide (1.1 equiv., 22 mmol) was then added, and the resulting reaction mixture was stirred at room temperature overnight. The completion of reaction was determined by TLC analysis. The reaction mixture was filtered and the filtrate was concentrated *in vacuo*. The residue was purified by column chromatography on silica gel using petroleum ether and ethyl acetate as eluent to afford the redox active ester (**RAE**).

### The synthesis of 1,3-dioxoisoindolin-2-yl 3,5,7-trifluoroadamantane-1-carboxylate (R3)

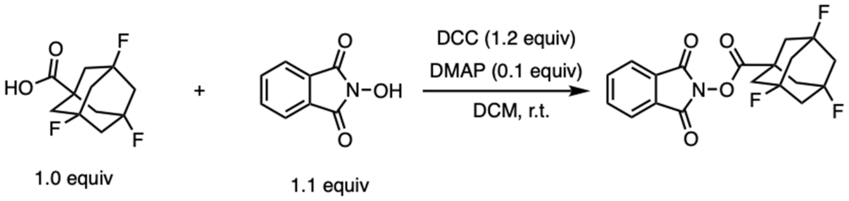


Following the General Procedure B, the title compound **R3** was prepared from 3,5,7-trifluoroadamantane-1-carboxylic acid (20 mmol, 4.68 g), *N*-hydroxyphthalimide (22 mmol, 3.59 g), DCC (24 mmol, 4.95 g), DMAP (2 mmol, 244.3 mg), and DCM (50 mL). After the reaction, the mixture was purified by column

chromatography on silica gel (petroleum ether/ethyl acetate = 10:1) to obtain the title compound as a white amorphous solid (4.02 g, 53%). **$^{1}$H NMR** (400 MHz, $CDCl_3$) $\delta$ 7.91 – 7.88 (m, 2H), 7.84 – 7.80 (m, 2H), 2.26 – 2.20 (m, 9H), 2.19 – 2.14 (m, 3H). **$^{13}$C NMR** (101 MHz, $CDCl_3$) $\delta$ 169.5, 161.7, 135.1, 128.9, 124.3, 92.4 (t, $J$ = 15.3 Hz), 90.5 (t, $J$ = 15.3 Hz), 46.9 – 45.4 (m), 42.8 – 42.5 (m), 42.1 – 40.7 (m). **$^{19}$F NMR** (376 MHz, $CDCl_3$) $\delta$ -143.54 (s, 3F). **HRMS** (ESI) m/z: $[M+Na]^+$ Calcd for $C_{19}H_{16}F_3NNaO_4^+$ 402.0929; Found 402.0934.

**The synthesis of 1,3-dioxoisoindolin-2-yl 2-bicyclo[2.2.1]heptane-2-carboxylate (R24)**

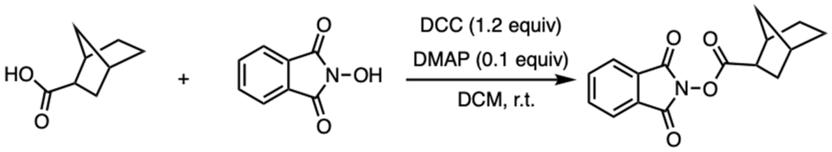


Following the General Procedure B, the title compound **R24** was prepared from 2-bicyclo[2.2.1]heptane-2-carboxylic acid (20 mmol, 2.80 g), *N*-hydroxyphthalimide (22 mmol, 3.59 g), DCC (24 mmol, 4.95 g), DMAP (2 mmol, 244.3 mg), and DCM (50 mL). After the reaction, the mixture was purified by column chromatography on silica gel (petroleum ether/ethyl acetate = 10:1) to obtain the title compound as a white amorphous solid (3.82 g, 67%). **$^{1}$H NMR** (400 MHz, $CDCl_3$) $\delta$ 7.87 – 7.84 (m, 2H), 7.79 – 7.75 (m, 2H), 3.14 – 3.09 (m, 1H), 2.80 – 2.75 (m, 1H), 2.37 – 2.31 (m, 1H), 1.86 – 1.78 (m, 1H), 1.72 – 1.67 (m, 1H), 1.65 – 1.52 (m, 3H), 1.51 – 1.42 (m, 2H), 1.35 – 1.21 (m, 1H). **$^{13}$C NMR** (101 MHz, $CDCl_3$) $\delta$ 171.5, 162.3, 134.8, 129.0, 124.0, 43.3, 41.0, 40.4, 36.9, 32.6, 28.9, 24.8. **HRMS** (ESI) m/z: $[M+Na]^+$ Calcd for $C_{16}H_{15}NNaO_4^+$ 308.0899; Found 308.0902.

# Optimization of reaction conditions

**Table S1.** Optimization of four-component synthesis of *N*-trifluoroalkyl aniline.[a]

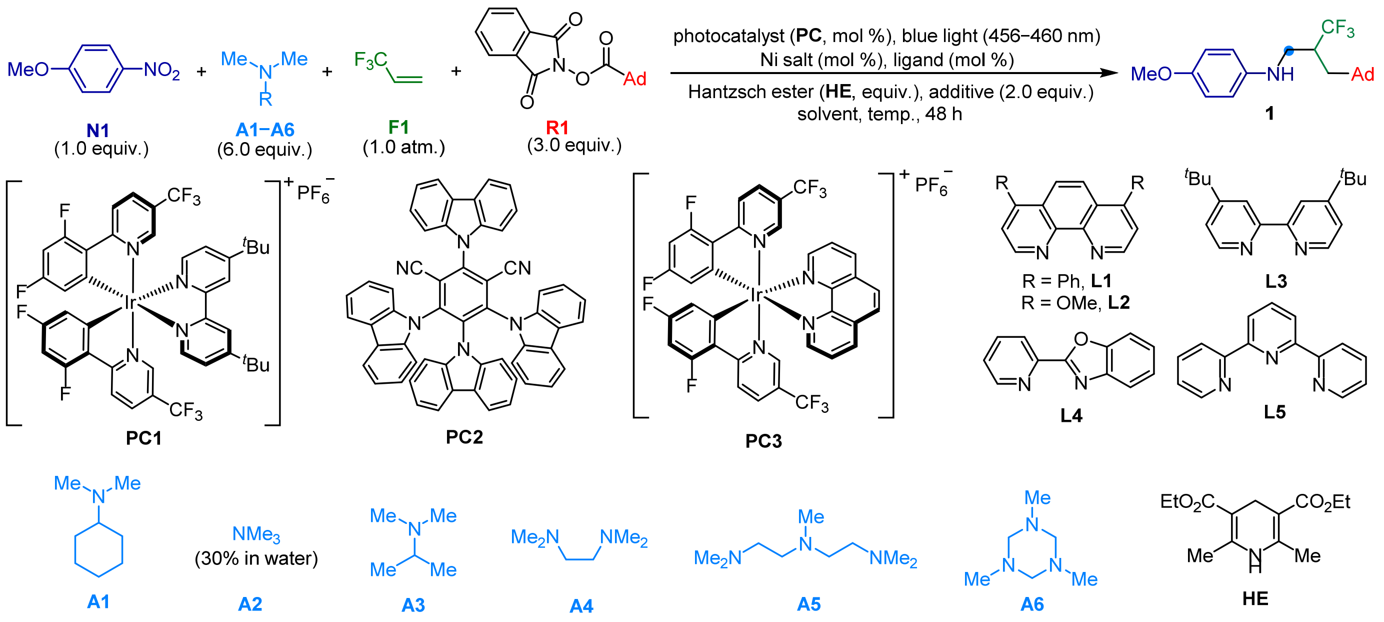


| Entry | PC (mol %) | Ni salt (mol %) | Ligand (mol %) | Amine (A) | HE (equiv.) | Additive (equiv.) | Solvent[b] | Temp./ °C | Yield/%[c] |
|---|---|---|---|---|---|---|---|---|---|
| 1 | **PC1** (3) | Ni(acac)$_2$ (20) | **L1** (20) | **A1** | 3 | HOTf (2) | 1,4-dioxane | ~40 | 46 |
| 2 | **PC2** (3) | Ni(acac)$_2$ (20) | **L1** (20) | **A1** | 3 | HOTf (2) | 1,4-dioxane | ~40 | 20 |
| 3 | **PC3** (3) | Ni(acac)$_2$ (20) | **L1** (20) | **A1** | 3 | HOTf (2) | 1,4-dioxane | ~40 | 34 |
| 4 | **PC1** (3) | Ni(acac)$_2$ (20) | **L1** (20) | **A1** | 3 | HOTf (2) | EtOAc | ~40 | 36 |
| 5 | **PC1** (3) | Ni(acac)$_2$ (20) | **L1** (20) | **A1** | 3 | HOTf (2) | DME | ~40 | 49 |
| 6 | **PC1** (3) | Ni(acac)$_2$ (20) | **L1** (20) | **A1** | 3 | HOTf (2) | MeCN | ~40 | 48 |
| 7 | **PC1** (3) | Ni(acac)$_2$ (20) | **L1** (20) | **A1** | 3 | HOTf (2) | DMSO | ~40 | 40 |
| 8 | **PC1** (3) | Ni(acac)$_2$ (20) | **L1** (20) | **A1** | 3 | HOTf (2) | DMSO/DME (1:1) | ~40 | 53 |
| 9 | **PC1** (3) | Ni(acac)$_2$ (20) | **L1** (20) | **A1** | 3 | HOTf (2) | DMSO/DME (2:1) | ~40 | 55 |
| 10 | **PC1** (4) | Ni(acac)$_2$ (20) | **L1** (20) | **A1** | 3 | HOTf (2) | DMSO/DME (2:1) | ~40 | 56 |
| 11 | **PC1** (4) | Ni(acac)$_2$ (25) | **L1** (25) | **A1** | 3 | HOTf (2) | DMSO/DME (2:1) | ~40 | 54 |
| 12 | **PC1** (4) | Ni(acac)$_2$ (15) | **L1** (15) | **A1** | 3 | HOTf (2) | DMSO/DME (2:1) | ~40 | 54 |
| 13 | **PC1** (4) | Ni(acac)$_2$ (20) | **L1** (20) | **A1** | 3 | HOTf (1) | DMSO/DME (2:1) | ~40 | 46 |
| 14 | **PC1** (4) | Ni(acac)$_2$ (20) | **L1** (20) | **A1** | 3 | HOTf (3) | DMSO/DME (2:1) | ~40 | 58 |

| | | | | | | | | | |
|---|---|---|---|---|---|---|---|---|---|
| 15 | **PC1** (4) | $Ni(acac)_2$ (20) | **L1** (20) | **A1** | 2 | HOTf (3) | DMSO/DME (2:1) | ~40 | 59 |
| 16 | **PC1** (4) | $Ni(acac)_2$ (20) | **L1** (20) | **A1** | 4 | HOTf (3) | DMSO/DME (2:1) | ~40 | 44 |
| 17 | **PC1** (4) | $Ni(acac)_2$ (20) | **L1** (20) | **A1** | 2 | HOTf (3) | DMSO/DME (2:1) | ~80 | 61 |
| 18 | **PC1** (4) | $Ni(NO_3)_2{\cdot}6H_2O$ (20) | **L1** (20) | **A1** | 2 | HOTf (3) | DMSO/DME (2:1) | ~80 | 76 |
| 19 | **PC1** (4) | $Ni(dme)Br_2$ (20) | **L1** (20) | **A1** | 2 | HOTf (3) | DMSO/DME (2:1) | ~80 | 62 |
| 20 | **PC1** (4) | $Ni(BF_4)_2{\cdot}6H_2O$ (20) | **L1** (20) | **A1** | 2 | HOTf (3) | DMSO/DME (2:1) | ~80 | 50 |
| 21 | **PC1** (4) | $Ni(ClO_4)_2{\cdot}6H_2O$ (20) | **L1** (20) | **A1** | 2 | HOTf (3) | DMSO/DME (2:1) | ~80 | 64 |
| 22 | **PC1** (4) | $Ni(NO_3)_2{\cdot}6H_2O$ (20) | **L2** (20) | **A1** | 2 | HOTf (3) | DMSO/DME (2:1) | ~80 | 39 |
| 23 | **PC1** (4) | $Ni(NO_3)_2{\cdot}6H_2O$ (20) | **L3** (20) | **A1** | 2 | HOTf (3) | DMSO/DME (2:1) | ~80 | 58 |
| 24 | **PC1** (4) | $Ni(NO_3)_2{\cdot}6H_2O$ (20) | **L4** (20) | **A1** | 2 | HOTf (3) | DMSO/DME (2:1) | ~80 | 65 |
| 25 | **PC1** (4) | $Ni(NO_3)_2{\cdot}6H_2O$ (20) | **L5** (20) | **A1** | 2 | HOTf (3) | DMSO/DME (2:1) | ~80 | 56 |
| 26 | **PC1** (4) | $Ni(NO_3)_2{\cdot}6H_2O$ (20) | **L1** (20) | **A1** | 2 | TMSCl (3) | DMSO/DME (2:1) | ~80 | 52 |
| 27 | **PC1** (4) | $Ni(NO_3)_2{\cdot}6H_2O$ (20) | **L1** (20) | **A1** | 2 | TMSBr (3) | DMSO/DME (2:1) | ~80 | 63 |
| 28 | **PC1** (4) | $Ni(NO_3)_2{\cdot}6H_2O$ (20) | **L1** (20) | **A1** | 2 | $EtCO_2H$ (3) | DMSO/DME (2:1) | ~80 | 50 |
| 29 | **PC1** (4) | $Ni(NO_3)_2$ $6H_2O$ (20) | **L1** (20) | **A2** | 2 | HOTf (3) | DMSO/DME (2:1) | ~80 | 0 |
| 30 | **PC1** (4) | $Ni(NO_3)_2{\cdot}6H_2O$ (20) | **L1** (20) | **A3** | 2 | HOTf (3) | DMSO/DME (2:1) | ~80 | 17 |
| 31 | **PC1** (4) | $Ni(NO_3)_2{\cdot}6H_2O$ (20) | **L1** (20) | **A4** | 2 | HOTf (3) | DMSO/DME (2:1) | ~80 | 36 |
| 32 | **PC1** (4) | $Ni(NO_3)_2{\cdot}6H_2O$ (20) | **L1** (20) | **A5** | 2 | HOTf (3) | DMSO/DME (2:1) | ~80 | 16 |
| 33 | **PC1** (4) | $Ni(NO_3)_2{\cdot}6H_2O$ (20) | **L1** (20) | **A6** | 2 | HOTf (3) | DMSO/DME (2:1) | ~80 | 38 |
| 34 | **PC1** (0) | $Ni(NO_3)_2{\cdot}6H_2O$ (20) | **L1** (20) | **A1** | 2 | HOTf (3) | DMSO/DME (2:1) | ~80 | 0 |
| 35 | **PC1** (4) | $Ni(NO_3)_2{\cdot}6H_2O$ (0) | **L1** (0) | **A1** | 2 | HOTf (3) | DMSO/DME (2:1) | ~80 | 64 |
| 36 | **PC1** (4) | $Ni(NO_3)_2{\cdot}6H_2O$ (20) | **L1** (20) | **A1** | 0 | HOTf (3) | DMSO/DME (2:1) | ~80 | 60 |
| 37 | **PC1** (4) | $Ni(NO_3)_2{\cdot}6H_2O$ (20) | **L1** (20) | **A1** | 2 | HOTf (3) | DMSO/DME (2:1) | ~80 | 54[d] |

[a]Reaction conditions: nitroarene (**N1**, 0.1 mmol, 1.0 equiv.), tertiary alkylamine (**A**, 6.0 equiv.), 3,3,3-trifluoropropene (**F1**, 1.0 atm.), redox active ester (**R1**, 3.0 equiv.), photocatalyst (**PC**), Ni salt (20 mol %), ligand (**L**, 20 mol %), Hantzsch ester (**HE**, 2.0 equiv.), additive, solvent (4.0 mL, 0.025 M), blue LEDs (456–460 nm, 30 W), 48 h. [b]The ratio in the blanket indicated the volume ratio of the reaction mixture. [c]Isolated yield. [d]blue LEDs (420–430 nm, 30 W).

**General procedure for optimizations of reaction conditions of photocatalytic four-component amination using nitroarenes, tertiary alkylamines, 3,3,3-trifluoropropene and redox active esters (Table S1):** An oven-dried, transparent 20 mL Teflon screw-capped Schlenk tube equipped with a stir bar was sequentially charged with nitroarene (**N1**, 1.0 equiv., 0.10 mmol), redox active ester (**R1**, 3.0 equiv., 0.30 mmol), photocatalyst (**PC1**–**PC3**), Ni catalyst, ligand (**L1**–**L5**), and Hantzsch ester (diethyl 1,4-dihydro-2,6-dimethyl-3,5-pyridinedicarboxylate, **HE**). Dried dimethyl sulfoxide (DMSO, 2.7 mL) and dried 1,2-dimethoxyethane (DME, 1.3 mL) were then transferred into the tube via syringe. Subsequently, tertiary alkylamine (**A1**–**A6**, 6.0 equiv., 0.60 mmol) and liquid additive (HOTf, TMSCl, TMSBr or $EtCO_2H$) were transferred into the tube via syringe. The resulting mixture was degassed via blowing with a balloon filled with 3,3,3-trifluoropropene (**F1**, ~1 L) for 2 min **(Left figure)**, after which time the tube was quickly capped with a Teflon screw cap such that it was filled with 3,3,3-trifluoropropene in atmospheric pressure. The reaction mixture was vigorously stirred and irradiated using 30 W blue LEDs ($\lambda$ = 456–460 nm) for 48 h under the ambient temperature of approximately ~80 ºC (without the use of fans for cooling, **Right figure**). At this point, the reaction mixture was diluted with ethyl acetate (~100 mL) and washed with water (~50 mL × 4). The organic fraction was further dried with anhydrous $Na_2SO_4$ and concentrated *in vacuo* with the aid of rotary evaporator. The residue was purified by preparative thin-layer chromatography using a mixture of petroleum ether and ethyl acetate as an eluent to afford the *N*-trifluoroalkyl aniline product **1** (The experimental setup as shown in **Fig. S1**).

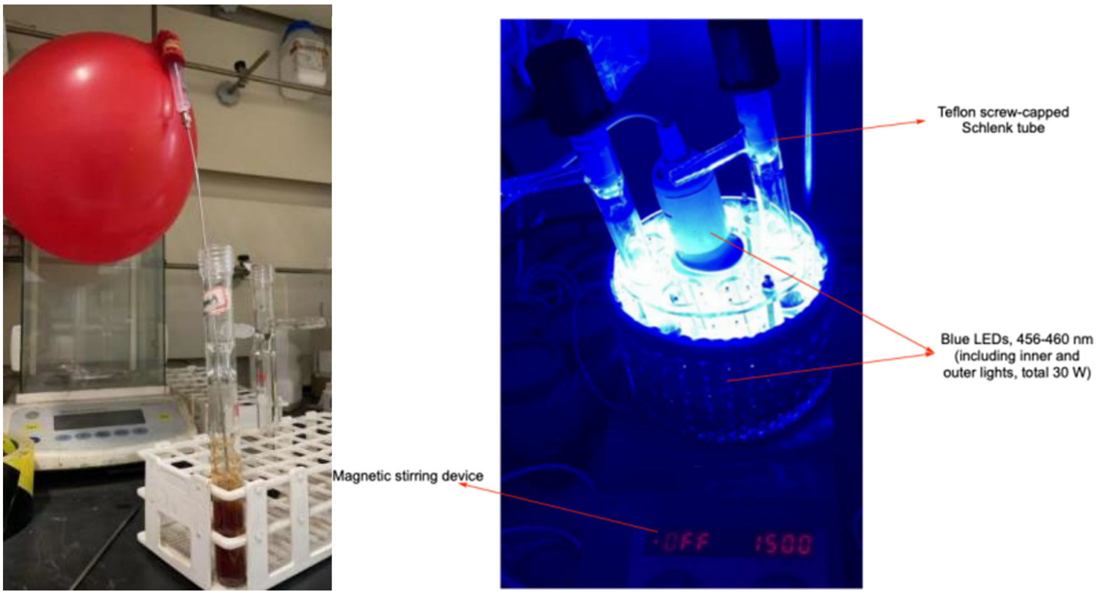


**Fig. S1.** The resulting mixture was degassed via blowing with a balloon filled with 3,3,3-trifluoropropene (**F1**, ~1 L), which is denser than air to promote the displacement of air (**Left**); The reaction mixture was vigorously stirred and irradiated using 30 W blue LEDs (**Right**).

**Table S2.** Optimization of four-component synthesis based on primary and secondary alkyl redox active ester[a]

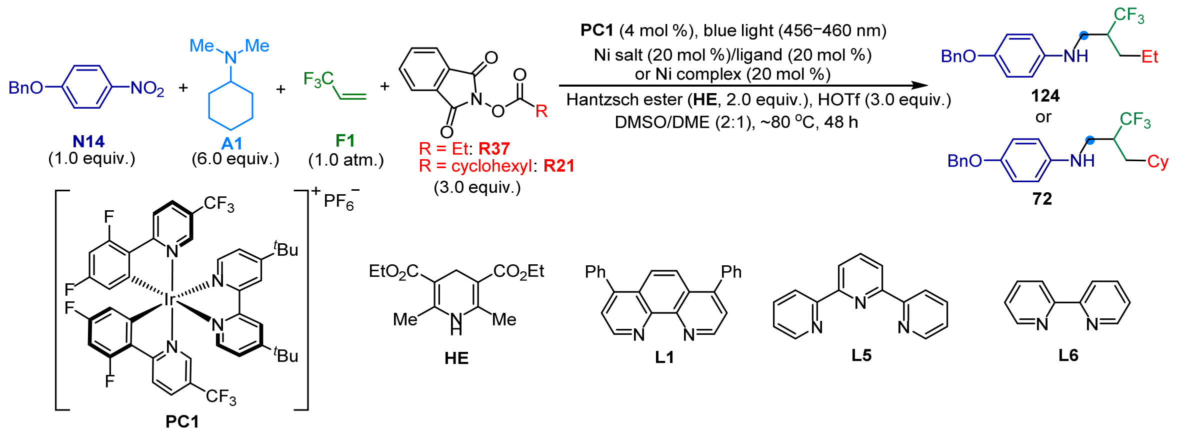


| Entry | Redox active ester | Ni salt/ligand or Ni complex | Yield of **124** or **72**/%[b] |
|---|---|---|---|
| 1 | **R37** | $Ni(NO_3)_2$•$6H_2O$/**L1** | 30 |
| 2 | **R37** | $Ni(ClO_4)_2$•$6H_2O$/**L1** | 31 |
| 3 | **R37** | $Ni(BF_4)_2$•$6H_2O$,/**L1** | 28 |
| 4 | **R37** | $Ni(OAc)_2$•$4H_2O$/**L1** | 33 |
| 5 | **R37** | $Ni(OTf)_2$/**L1** | 29 |
| 6 | **R37** | $Ni(acac)_2$/**L1** | 27 |
| 7 | **R37** | $NiCl_2$/**L1** | 36 |
| 8 | **R37** | $NiBr_2$/**L1** | 37 |
| 9 | **R37** | $NiI_2$/**L1** | 33 |
| 10 | **R37** | Ni(**L1**)$Br_2$ | 34 |
| 11 | **R37** | Ni(**L5**)$Cl_2$ | 36 |
| 12 | **R37** | Ni(**L6**)$Cl_2$ | 43 |
| 13 | **R37** | Ni(**L6**)$Cl_2$ | 36[c] |
| 14 | **R37** | No Ni(**L6**)$Cl_2$ | 27 |
| 15 | **R21** | Ni(**L6**)$Cl_2$ | 57 |
| 16 | **R21** | No Ni(**L6**)$Cl_2$ | 35 |

[a]Reaction conditions: nitroarene (**N14**, 0.1 mmol, 1.0 equiv.), *N*,*N*-dimethylcyclohexylamine (**A1**, 6.0 equiv.), 3,3,3-trifluoropropene (**F1**, 1.0 atm.), redox active ester (**R37** or **R21**, 3.0 equiv), **PC1** (4 mol %), Ni salt (20 mol %)/ligand (**L**, 20 mol %) or Ni complex (20 mol %), Hantzsch ester (**HE**, 2.0 equiv.), HOTf (3.0 equiv.), DMSO/DME (v/v = 2:1, 4.0 mL, 0.025

M), blue light (456–460 nm, 30 W), ~80 °C, 48 h. [b]Isolated yield. [c]$CuBr_2$ (10 mol %) was added as additional additive.

**General procedure for Optimization of four-component synthesis based on primary and secondary alkyl redox active ester (Table S2):** An oven-dried, transparent 20 mL Teflon screw-capped Schlenk tube equipped with a stir bar was sequentially charged with nitroarene (**N14**, 1.0 equiv., 0.10 mmol), redox active ester (**R21** or **R37**, 3.0 equiv., 0.30 mmol), photocatalyst (**PC1**), Ni catalyst (20 mol %, 0.020 mmol), ligand (**L1**, **L5, L6,** 20 mol %, 0.020 mmol), and Hantzsch ester (diethyl 1,4-dihydro-2,6-dimethyl-3,5-pyridinedicarboxylate, **HE,** 2 equiv., 0.20 mmol). Dried dimethyl sulfoxide (DMSO, 2.7 mL) and dried 1,2-dimethoxyethane (DME, 1.3 mL) were then transferred into the tube via syringe. Subsequently, tertiary alkylamine (**A1**, 6.0 equiv., 0.60 mmol) and triflic acid (HOTf, 3.0 equiv., 0.30 mmol) were transferred into the tube via syringe. The resulting mixture was degassed via blowing with a balloon filled with 3,3,3-trifluoropropene (**F1**, ~1 L) for 2 min, after which time the tube was quickly capped with a Teflon screw cap such that it was filled with 3,3,3-trifluoropropene in atmospheric pressure. The reaction mixture was vigorously stirred and irradiated using 30 W blue LEDs ($\lambda$ = 456–460 nm) for 48 h under the ambient temperature of approximately ~80 °C (without the use of fans for cooling). At this point, the reaction mixture was diluted with ethyl acetate (100 mL) and washed with water (50 mL × 4). The organic fraction was further dried with anhydrous $Na_2SO_4$ and concentrated *in vacuo* with the aid of rotary evaporator. The residue was purified by preparative thin-layer chromatography using a mixture of petroleum ether and ethyl acetate as an eluent to afford the *N*-trifluoroalkyl aniline product **124** or **72**.

# Mechanistic Study

## (a) Radical clock experiment

RAE derived from hept-6-enoic acid (**R38**) reacted under the standard reaction conditions to give the ring-closing product **125** (**Fig. S2**), suggesting the formation of a hex-5-enyl radical which cyclizes to form a cyclopentylmethyl radical for amination reaction.

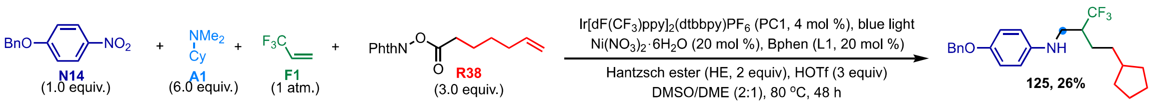


**Fig. S2.** Radical clock experiment using RAE derived from hept-6-enoic acid.

**Reaction Procedure:** An oven-dried, transparent 20 mL Schlenk tube equipped with a stir bar was sequentially charged with nitroarene (**N14**, 1.0 equiv., 0.15 mmol), redox active ester (**R38**, 3.0 equiv., 0.45 mmol), Ir[dF($CF_3$)ppy]$_2$(dtbbpy)$PF_6$ (**PC1**, 4 mol %, 0.0060 mmol), $Ni(NO_3)_2$•$6H_2O$ (20 mol %, 0.030 mmol), bathophenanthroline (BPhen, **L1**, 20 mol %, 0.030 mmol), and Hantzsch ester (diethyl 1,4-dihydro-2,6-dimethyl-3,5-pyridinedicarboxylate, **HE**, 2.0 equiv., 0.30 mmol). Dried dimethyl sulfoxide (DMSO, 4.0 mL) and dried 1,2-dimethoxyethane (DME, 2.0 mL) were then transferred into the tube via syringe. Subsequently, tertiary alkylamine (**A1**, 6.0 equiv., 0.90 mmol) and triflic acid (HOTf, 3.0 equiv., 0.45 mmol) were transferred into the tube via syringe. The resulting mixture was degassed via blowing with a balloon filled with 3,3,3-trifluoropropene (**F1**, ~1 L) for 2 min, after which time the tube was quickly capped with a Teflon screw cap such that it was filled with 3,3,3-trifluoropropene in atmospheric pressure. The reaction mixture was vigorously stirred and irradiated using 30 W blue LEDs ($\lambda$ = 456–460 nm) for 48 h under the ambient temperature of approximately ~80 °C (without the use of fans for cooling). At this point, the reaction mixture was diluted with ethyl acetate (~100 mL) and washed with water (~50 mL × 4). The organic fraction was further dried with anhydrous $Na_2SO_4$ and concentrated in vacuo with the aid of rotary evaporator. The residue was purified by preparative thin-layer chromatography using petroleum ether/ethyl acetate

(20:1) as an eluent to give the yellow viscous oil **125** (15.4 mg, 26%). $^{1}$H NMR (800 MHz, $CDCl_3$) $\delta$ 7.43 (d, $J$ = 7.5 Hz, 2H), 7.38 (t, $J$ = 7.5 Hz, 2H), 7.31 (t, $J$ = 7.3 Hz, 1H), 6.87 (d, $J$ = 8.5 Hz, 2H), 6.58 (d, $J$ = 8.5 Hz, 2H), 5.00 (s, 2H), 3.58 (brs, 1H), 3.36 – 3.34 (m, 1H), 3.25 – 3.23 (m, 1H), 2.37 – 2.32 (m, 1H), 1.77 – 1.65 (m, 4H), 1.60 – 1.57 (m, 2H), 1.54 – 1.50 (m, 3H), 1.46 – 1.39 (m, 2H), 1.10 – 1.05 (m, 2H). $^{13}$C NMR (201 MHz, $CDCl_3$) $\delta$ 151.8, 141.9, 137.6, 128.7, 128.4 (q, $^{1}J_{CF}$ = 281.8 Hz), 128.0, 127.6, 116.4, 114.4, 71.0, 43.2 (q, $^{3}J_{CF}$ = 2.8 Hz), 42.6 (q, $^{2}J_{CF}$ = 23.8 Hz), 40.2, 33.4, 32.7, 32.6, 25.3. $^{19}$F NMR (471 MHz, $CDCl_3$) $\delta$ -69.18 (s, 3F). HRMS (ESI) m/z: $[M+H]^+$ Calcd for $C_{23}H_{29}F_3NO^+$ 392.2196; Found 392.2194.

## (b) Radical trap experiment

When 2,2,6,6-tetramethylpiperidine-1-oxyl (TEMPO), acting as a radical scavenger, was introduced to the model reaction, only a trace amount of *N*-trifluoroalkyl aniline **14** was produced by TCL analysis. High-resolution mass spectrometry (HRMS) analysis identified several TEMPO-captured adducts (**T1**-**T7**) (**Fig. S3**). These findings indicated the presence of these radical intermediates, which are implicated in the reaction pathway leading to the formation of *N*-trifluoroalkyl aniline product **14**.

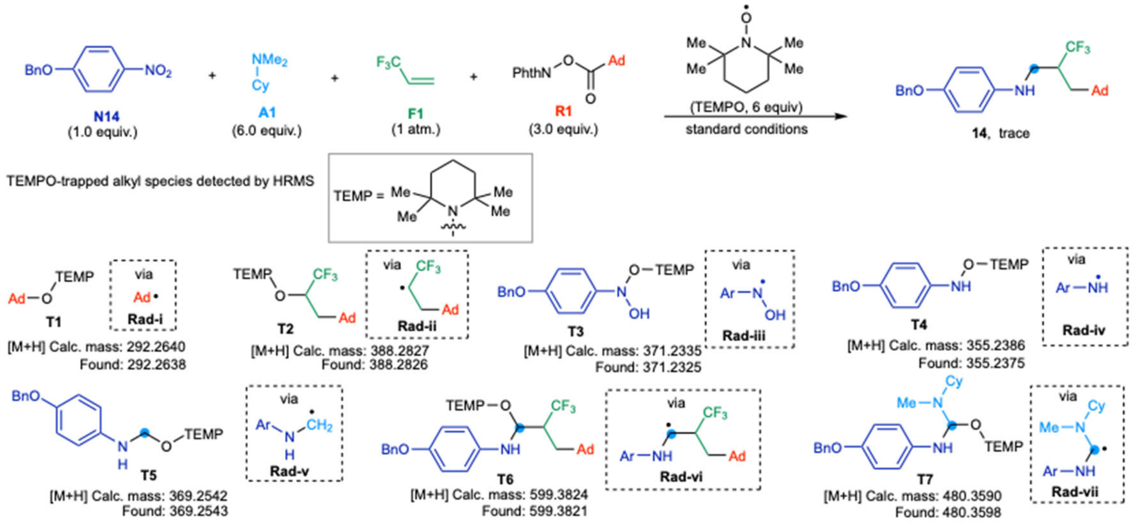


**Fig. S3.** Identification of radical species using TEMPO as the radical trap.

**Reaction Procedure:** An oven-dried, transparent 20 mL Schlenk tube equipped with a stir bar was sequentially charged with nitroarene (**N14**, 1.0 equiv., 0.15 mmol), redox active ester (**R1**, 3.0 equiv., 0.45 mmol), Ir[dF($CF_3$)ppy]$_2$(dtbbpy)$PF_6$ (**PC1**, 4 mol %, 0.0060 mmol), $Ni(NO_3)_2$•$6H_2O$ (20 mol %, 0.030 mmol), bathophenanthroline (BPhen, **L1**, 20 mol %, 0.030 mmol), Hantzsch ester (diethyl 1,4-dihydro-2,6-dimethyl-3,5-pyridinedicarboxylate, **HE**, 2.0 equiv., 0.30 mmol), and 2,2,6,6-tetramethyl-1-piperidinyloxy (TEMPO, 6.0 equiv., 0.90 mmol). Dried dimethyl sulfoxide (DMSO, 4.0 mL) and dried 1,2-dimethoxyethane (DME, 2.0 mL) were then transferred into the tube via syringe. Subsequently, *N*,*N*-dimethylcyclohexylamine (**A1**, 6.0 equiv., 0.90 mmol) and triflic acid (HOTf, 3.0 equiv., 0.45 mmol) were transferred into the tube via syringe. The resulting mixture was degassed via blowing with a balloon filled with 3,3,3-trifluoropropene (**F1**, ~1 L) for 2 min, after which time the tube was quickly capped with a Teflon screw cap such

that it was filled with 3,3,3-trifluoropropene in atmospheric pressure. The reaction mixture was vigorously stirred and irradiated using 30 W blue LEDs (λ = 456–460 nm) for 48 h under the ambient temperature of approximately ~80 ºC (without the use of fans for cooling). After the reaction, the mixture was monitored by TLC. A trace of *N*-trifluoroalkyl aniline **14** was detected by TLC analysis, and **T1**–**T7** was successfully detected by HRMS analysis (as shown below).

**Elemental Composition Report** **Page 1**

**Single Mass Analysis**
Tolerance = 5.0 mDa / DBE: min = -1.5, max = 50.0
Element prediction: Off
Number of isotope peaks used for i-FIT = 3

Monoisotopic Mass, Even Electron Ions
367 formula(e) evaluated with 1 results within limits (up to 50 best isotopic matches for each mass)
Elements Used:
C: 19-19 H: 34-34 N: 0-200 O: 0-200 Na: 0-1

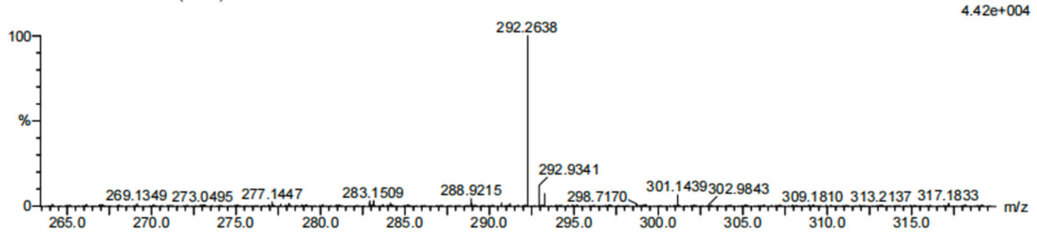


| | | | | | | | | |
|---|---|---|---|---|---|---|---|---|
| Minimum: | | | | -1.5 | | | | |
| Maximum: | | 5.0 | 10.0 | 50.0 | | | | |
| Mass | Calc. Mass | mDa | PPM | DBE | i-FIT | Norm | Conf(%) | Formula |
| 292.2638 | 292.2640 | -0.2 | -0.7 | 3.5 | 363.4 | n/a | n/a | C19 H34 N O |

HRMS of [**T1**+H]$^{+}$ calcd 292.2640; found 292.2638

**Elemental Composition Report** **Page 1**

**Single Mass Analysis**
Tolerance = 5.0 mDa / DBE: min = -1.5, max = 50.0
Element prediction: Off
Number of isotope peaks used for i-FIT = 3

Monoisotopic Mass, Even Electron Ions
475 formula(e) evaluated with 1 results within limits (up to 50 best isotopic matches for each mass)
Elements Used:
C: 22-22 H: 37-37 N: 0-200 O: 0-200 Na: 0-1 F: 3-3

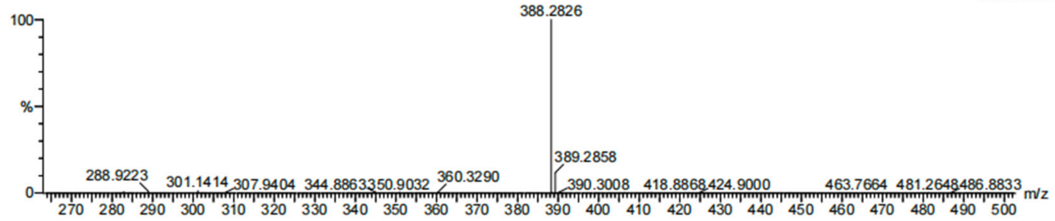


| | | | | | | | | |
|---|---|---|---|---|---|---|---|---|
| Minimum: | | | | -1.5 | | | | |
| Maximum: | | 5.0 | 10.0 | 50.0 | | | | |
| Mass | Calc. Mass | mDa | PPM | DBE | i-FIT | Norm | Conf(%) | Formula |
| 388.2826 | 388.2827 | -0.1 | -0.3 | 3.5 | 269.8 | n/a | n/a | C22 H37 N O F3 |

HRMS of [**T2**+H]$^{+}$ calcd 388.2827; found 388.2826

**Elemental Composition Report** 

**Single Mass Analysis**
Tolerance = 5.0 mDa / DBE: min = -1.5, max = 50.0
Element prediction: Off
Number of isotope peaks used for i-FIT = 3

Monoisotopic Mass, Even Electron Ions
300 formula(e) evaluated with 1 results within limits (up to 50 best isotopic matches for each mass)
Elements Used:
C: 22-22 H: 31-31 N: 0-100 O: 0-100
23
240501-4-ZY-TEMPO-3 3 (0.087) 1: TOF MS ES+ 7.38e+003

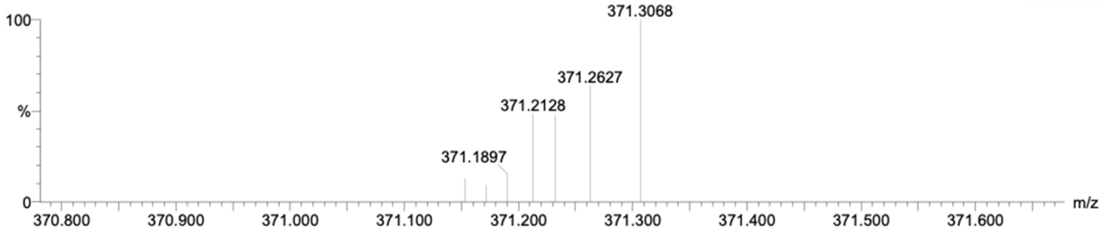


| | | | | | | | | |
|---|---|---|---|---|---|---|---|---|
| Minimum: | | | | -1.5 | | | | |
| Maximum: | | 5.0 | 10.0 | 50.0 | | | | |
| Mass | Calc. Mass | mDa | PPM | DBE | i-FIT | Norm | Conf(%) | Formula |
| 371.2325 | 371.2335 | -1.0 | -2.7 | 8.5 | 61.8 | n/a | n/a | C22 H31 N2 O3 |

HRMS of [**T3**+H]$^+$ calcd 371.2335; found 371.2325

**Elemental Composition Report** Page 1

**Single Mass Analysis**
Tolerance = 5.0 mDa / DBE: min = -1.5, max = 50.0
Element prediction: Off
Number of isotope peaks used for i-FIT = 3

Monoisotopic Mass, Even Electron Ions
280 formula(e) evaluated with 1 results within limits (up to 50 best isotopic matches for each mass)
Elements Used:
C: 22-22 H: 31-31 N: 0-100 O: 0-100
23
240501-4-ZY-TEMPO-3 3 (0.087) 1: TOF MS ES+ 1.09e+004

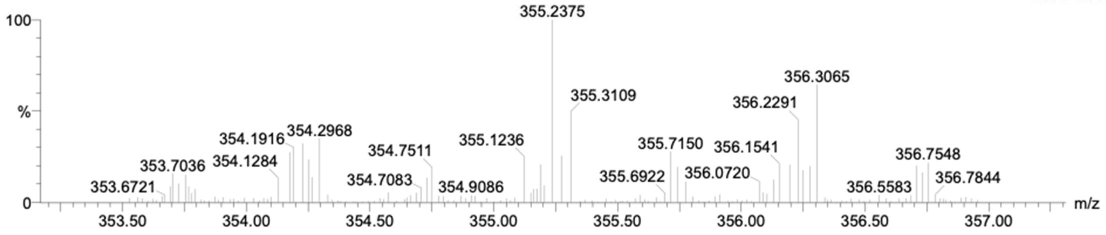


| | | | | | | | | |
|---|---|---|---|---|---|---|---|---|
| Minimum: | | | | -1.5 | | | | |
| Maximum: | | 5.0 | 10.0 | 50.0 | | | | |
| Mass | Calc. Mass | mDa | PPM | DBE | i-FIT | Norm | Conf(%) | Formula |
| 355.2375 | 355.2386 | -1.1 | -3.1 | 8.5 | 823.9 | n/a | n/a | C22 H31 N2 O2 |

HRMS of [**T4**+H]$^+$ calcd 355.2386; found 355.2375

## Elemental Composition Report

Page 1

### Single Mass Analysis

Tolerance = 5.0 mDa / DBE: min = -1.5, max = 50.0
Element prediction: Off
Number of isotope peaks used for i-FIT = 3

Monoisotopic Mass, Even Electron Ions
576 formula(e) evaluated with 1 results within limits (up to 50 best isotopic matches for each mass)
Elements Used:
C: 23-23 H: 33-33 N: 0-200 O: 0-200 Na: 0-1
6
230924-10-ZT-TEMPO 22 (0.222)

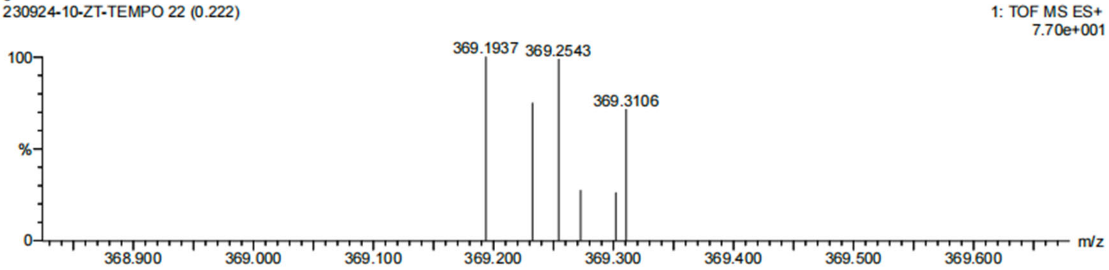


| | | | | | | | | |
|---|---|---|---|---|---|---|---|---|
| Minimum: | | | | -1.5 | | | | |
| Maximum: | | 5.0 | 10.0 | 50.0 | | | | |
| Mass | Calc. Mass | mDa | PPM | DBE | i-FIT | Norm | Conf(%) | Formula |
| 369.2543 | 369.2542 | 0.1 | 0.3 | 8.5 | 37.4 | n/a | n/a | C23 H33 N2 O2 |

HRMS of [**T5**+H]$^+$ calcd 369.2542; found 369.2543

## Elemental Composition Report

Page 1

### Single Mass Analysis

Tolerance = 5.0 mDa / DBE: min = -1.5, max = 50.0
Element prediction: Off
Number of isotope peaks used for i-FIT = 3

Monoisotopic Mass, Even Electron Ions
3282 formula(e) evaluated with 1 results within limits (up to 50 best isotopic matches for each mass)
Elements Used:
C: 36-36 H: 50-50 N: 0-100 O: 0-100 F: 1-5
23
240501-4-ZY-TEMPO-3 3 (0.087)

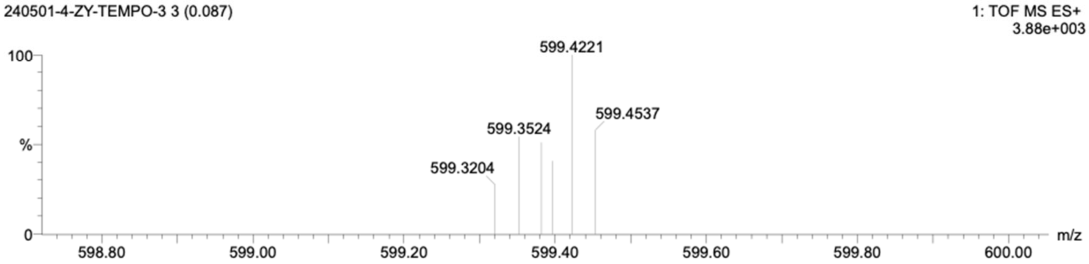


| | | | | | | | | |
|---|---|---|---|---|---|---|---|---|
| Minimum: | | | | -1.5 | | | | |
| Maximum: | | 5.0 | 10.0 | 50.0 | | | | |
| Mass | Calc. Mass | mDa | PPM | DBE | i-FIT | Norm | Conf(%) | Formula |
| 599.3821 | 599.3824 | -0.3 | -0.5 | 11.5 | 73.1 | n/a | n/a | C36 H50 N2 O2 F3 |

HRMS of [**T6**+H]$^+$ calcd 599.3824; found 599.3821

**Elemental Composition Report** 

**Single Mass Analysis**
Tolerance = 5.0 mDa / DBE: min = -1.5, max = 50.0
Element prediction: Off
Number of isotope peaks used for i-FIT = 3

Monoisotopic Mass, Even Electron Ions
1544 formula(e) evaluated with 1 results within limits (up to 50 best isotopic matches for each mass)
Elements Used:
C: 30-30 H: 46-46 N: 0-20 O: 0-100 Na: 0-3

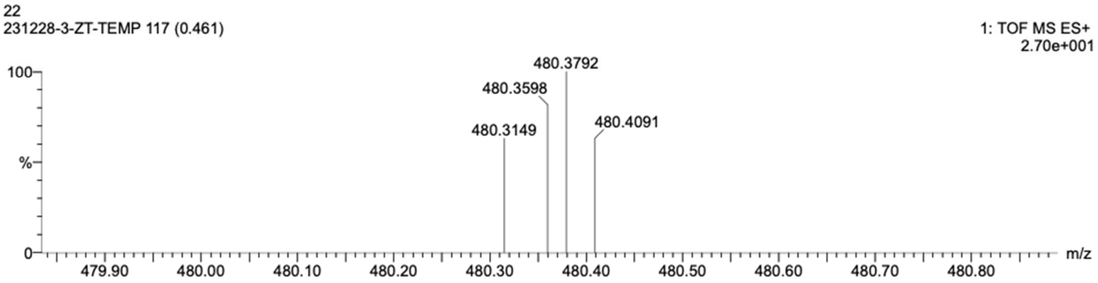


| | | | | | | | | |
|---|---|---|---|---|---|---|---|---|
| Minimum: | | | | -1.5 | | | | |
| Maximum: | | 5.0 | 10.0 | 50.0 | | | | |

| Mass | Calc. Mass | mDa | PPM | DBE | i-FIT | Norm | Conf(%) | Formula |
|---|---|---|---|---|---|---|---|---|
| 480.3598 | 480.3590 | 0.8 | 1.7 | 9.5 | 26.7 | n/a | n/a | C30 H46 N3 O2 |

HRMS of [**T7**+H]$^+$ calcd 480.3590; found 480.3598

## (C) Analysis of reaction co-products in the early reaction stage

During the initial phase of the model reaction (4 h), the reaction mixture was subject to HRMS analysis to identify emerging reaction intermediates and co-products. High-resolution mass spectrometry (HRMS) analysis identified several co-products (**S1**–**S3**, **S5**–**S7**) (**Fig. S4**).

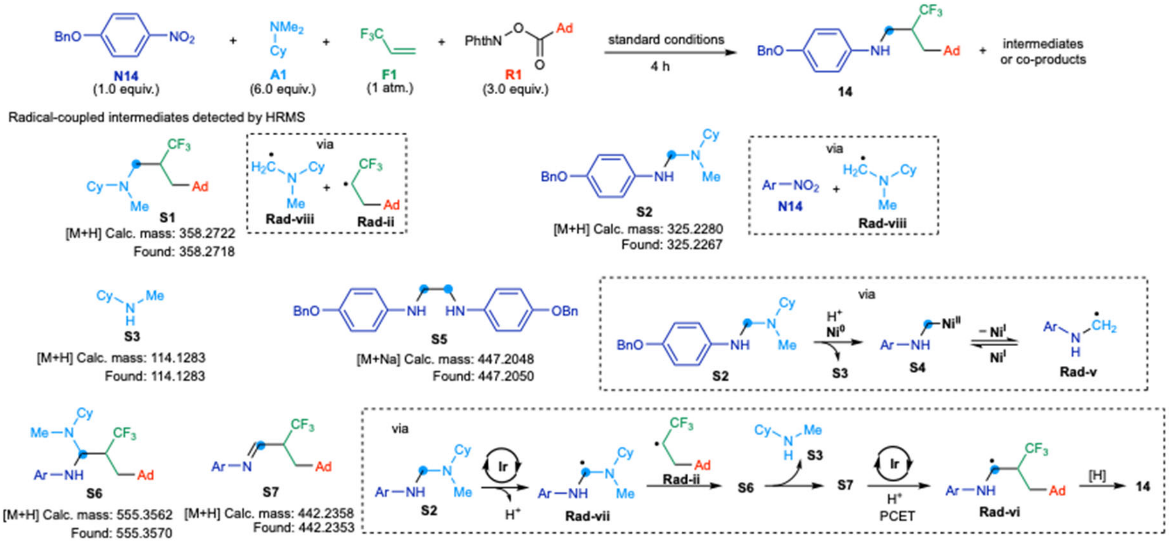


**Fig. S4.** Analysis of reaction co-products in the early reaction stage.

**Reaction Procedure:** An oven-dried, transparent 20 mL Schlenk tube equipped with a stir bar was sequentially charged with nitroarene (**N14**, 1.0 equiv., 0.15 mmol), redox active ester (**R1**, 3.0 equiv., 0.45 mmol), Ir[dF($CF_3$)ppy]$_2$(dtbbpy)$PF_6$ (**PC1**, 4 mol %, 0.0060 mmol), Ni($NO_3$)$_2$•6$H_2O$ (20 mol %, 0.030 mmol), bathophenanthroline (BPhen, **L1**, 20 mol %, 0.030 mmol) and Hantzsch ester (diethyl 1,4-dihydro-2,6-dimethyl-3,5-pyridinedicarboxylate, **HE**, 2.0 equiv., 0.30 mmol). Dried dimethyl sulfoxide (DMSO, 4.0 mL) and dried 1,2-dimethoxyethane (DME, 2.0 mL) were then transferred into the tube via syringe. Subsequently, *N*,*N*-dimethylcyclohexylamine (**A1**, 6.0 equiv., 0.90 mmol) and triflic acid (HOTf, 3.0 equiv., 0.45 mmol) were transferred into the tube via syringe. The resulting mixture was degassed via blowing with a balloon filled with 3,3,3-trifluoropropene (**F1**, ~1 L) for 2 min, after which time the tube was quickly capped with a Teflon screw cap such that it was filled with 3,3,3-trifluoropropene in atmospheric pressure. The reaction mixture was vigorously stirred and irradiated using 30 W blue LEDs (λ = 456–460 nm) for 4 h under the ambient temperature of approximately ~80 ºC (without the use

of fans for cooling). After the reaction, the mixture was detected by HRMS, and **S1**, **S2, S3, S5, S6, S7** was successfully detected by HRMS (as shown below).

**Elemental Composition Report** Page 1

**Single Mass Analysis**
Tolerance = 5.0 mDa / DBE: min = -1.5, max = 50.0
Element prediction: Off
Number of isotope peaks used for i-FIT = 3

Monoisotopic Mass, Even Electron Ions
400 formula(e) evaluated with 1 results within limits (up to 50 best isotopic matches for each mass)
Elements Used:
C: 21-21 H: 35-35 N: 0-200 O: 0-200 Na: 0-1 F: 3-3

6
230924-10-ZT-4H 10 (0.111)

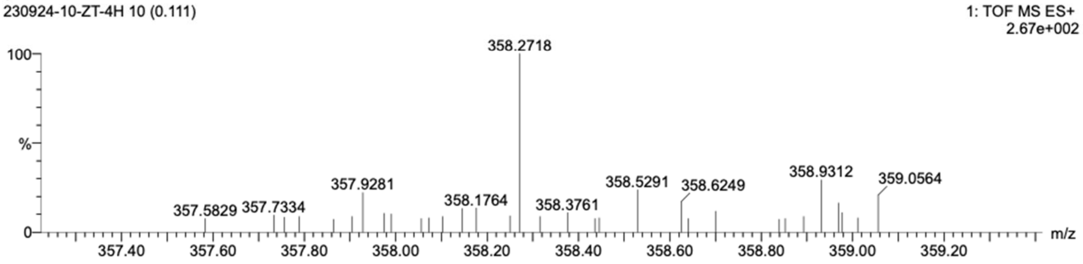


| | | | | |
|---|---|---|---|---|
| Minimum: | | | | -1.5 |
| Maximum: | | 5.0 | 10.0 | 50.0 |

| Mass | Calc. Mass | mDa | PPM | DBE | i-FIT | Norm | Conf(%) | Formula |
|---|---|---|---|---|---|---|---|---|
| 358.2718 | 358.2722 | -0.4 | -1.1 | 3.5 | 130.6 | n/a | n/a | C21 H35 N F3 |

HRMS of [**S1**+H]$^+$ calcd 358.2722; found 358.2718

**Elemental Composition Report** Page 1

**Single Mass Analysis**
Tolerance = 5.0 mDa / DBE: min = -1.5, max = 50.0
Element prediction: Off
Number of isotope peaks used for i-FIT = 3

Monoisotopic Mass, Even Electron Ions
454 formula(e) evaluated with 1 results within limits (up to 50 best isotopic matches for each mass)
Elements Used:
C: 21-21 H: 29-29 N: 0-200 O: 0-200 Na: 0-1
6
230924-10-ZT-4H 5 (0.068)

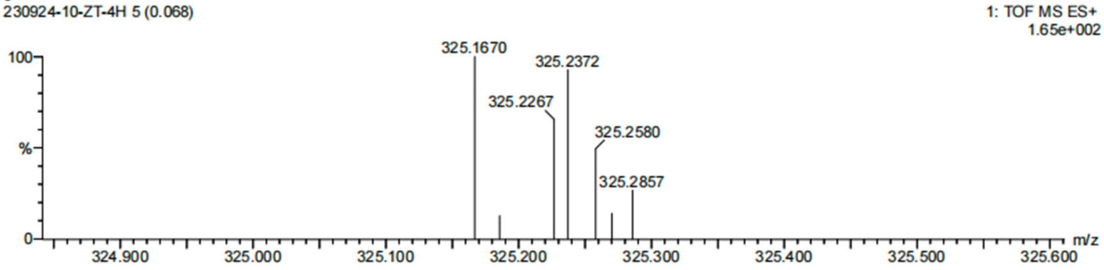


| | | | | |
|---|---|---|---|---|
| Minimum: | | | | -1.5 |
| Maximum: | | 5.0 | 10.0 | 50.0 |

| Mass | Calc. Mass | mDa | PPM | DBE | i-FIT | Norm | Conf(%) | Formula |
|---|---|---|---|---|---|---|---|---|
| 325.2267 | 325.2280 | -1.3 | -4.0 | 8.5 | 50.9 | n/a | n/a | C21 H29 N2 O |

HRMS of [**S2**+H]$^+$ calcd 325.2280; found 325.2267

## Elemental Composition Report



### Single Mass Analysis

Tolerance = 5.0 mDa / DBE: min = -1.5, max = 50.0
Element prediction: Off
Number of isotope peaks used for i-FIT = 3

Monoisotopic Mass, Even Electron Ions
80 formula(e) evaluated with 1 results within limits (up to 50 best isotopic matches for each mass)
Elements Used:
C: 7-7 H: 16-16 N: 0-20 O: 0-100 Na: 0-3
22
231228-3-ZT-4H 19 (0.091)

1: TOF MS ES+
2.04e+003

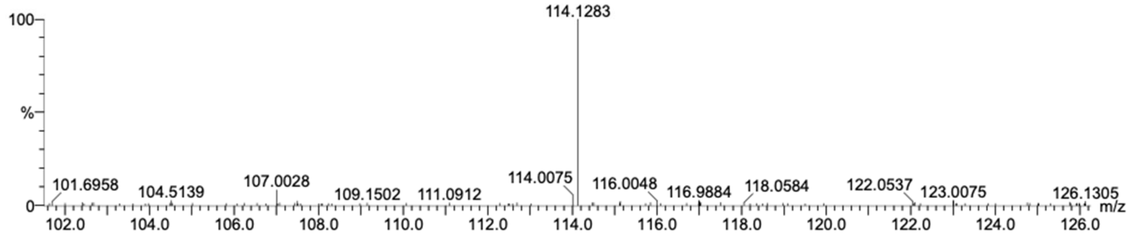


| | Calc. Mass | mDa | PPM | DBE | i-FIT | Norm | Conf(%) | Formula |
|---|---|---|---|---|---|---|---|---|
| Minimum: | | | | -1.5 | | | | |
| Maximum: | | 5.0 | 10.0 | 50.0 | | | | |

| Mass | Calc. Mass | mDa | PPM | DBE | i-FIT | Norm | Conf(%) | Formula |
|---|---|---|---|---|---|---|---|---|
| 114.1283 | 114.1283 | 0.0 | 0.0 | 0.5 | 60.3 | n/a | n/a | C7 H16 N |

HRMS of [**S3**+H]$^+$ calcd 114.1283; found 114.1283

## Elemental Composition Report



### Single Mass Analysis

Tolerance = 5.0 mDa / DBE: min = -1.5, max = 50.0
Element prediction: Off
Number of isotope peaks used for i-FIT = 3

Monoisotopic Mass, Even Electron Ions
1389 formula(e) evaluated with 1 results within limits (up to 50 best isotopic matches for each mass)
Elements Used:
C: 28-28 H: 28-28 N: 0-20 O: 0-100 Na: 0-3
22
231228-3-ZT-4H 79 (0.318)

1: TOF MS ES+
6.28e+001

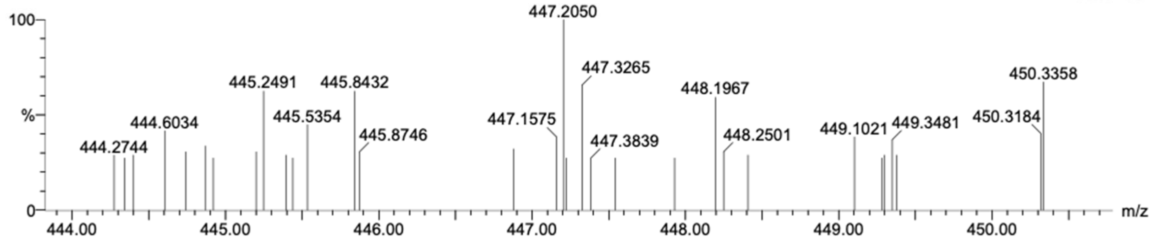


| | Calc. Mass | mDa | PPM | DBE | i-FIT | Norm | Conf(%) | Formula |
|---|---|---|---|---|---|---|---|---|
| Minimum: | | | | -1.5 | | | | |
| Maximum: | | 5.0 | 10.0 | 50.0 | | | | |

| Mass | Calc. Mass | mDa | PPM | DBE | i-FIT | Norm | Conf(%) | Formula |
|---|---|---|---|---|---|---|---|---|
| 447.2050 | 447.2048 | 0.2 | 0.4 | 15.5 | 87.0 | n/a | n/a | C28 H28 N2 O2 Na |

HRMS of [**S5**+Na]$^+$ calcd 447.2048; found 447.2050

**Elemental Composition Report** **Page 1**

**Single Mass Analysis**
Tolerance = 5.0 mDa / DBE: min = -1.5, max = 50.0
Element prediction: Off
Number of isotope peaks used for i-FIT = 3

Monoisotopic Mass, Even Electron Ions
1686 formula(e) evaluated with 1 results within limits (up to 50 best isotopic matches for each mass)
Elements Used:
C: 34-34 H: 46-46 N: 0-20 O: 0-100 F: 3-3 Na: 0-3
22
231228-3-ZT-4H 19 (0.091) 1: TOF MS ES+ 2.48e+002

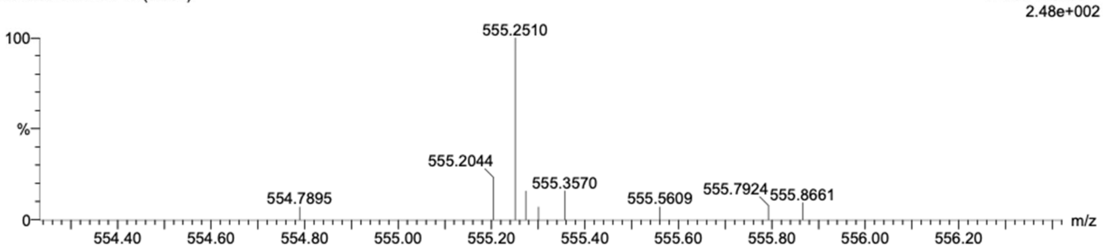


| | | | | | | | | |
|---|---|---|---|---|---|---|---|---|
| Minimum: | | | | -1.5 | | | | |
| Maximum: | | 5.0 | 10.0 | 50.0 | | | | |
| Mass | Calc. Mass | mDa | PPM | DBE | i-FIT | Norm | Conf(%) | Formula |
| 555.3570 | 555.3562 | 0.8 | 1.4 | 11.5 | 34.5 | n/a | n/a | C34 H46 N2 O F3 |

HRMS of [**S6**+H]$^+$ calcd 555.3562; found 555.3570

**Elemental Composition Report** **Page 1**

**Single Mass Analysis**
Tolerance = 5.0 mDa / DBE: min = -1.5, max = 50.0
Element prediction: Off
Number of isotope peaks used for i-FIT = 3

Monoisotopic Mass, Even Electron Ions
1126 formula(e) evaluated with 1 results within limits (up to 50 best isotopic matches for each mass)
Elements Used:
C: 27-27 H: 31-31 N: 0-20 O: 0-100 F: 3-3 Na: 0-3
22
231228-3-ZT-4H 20 (0.095) 1: TOF MS ES+ 1.23e+003

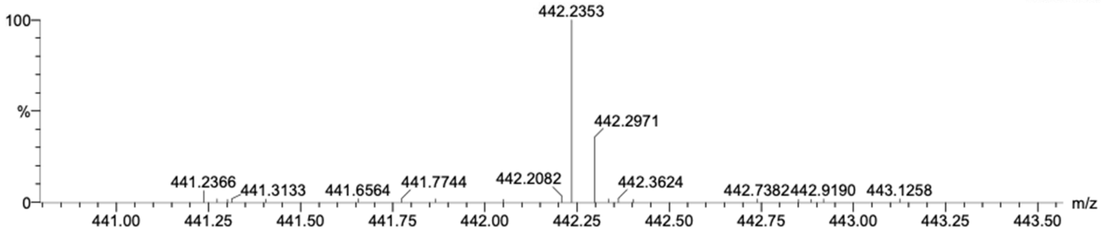


| | | | | | | | | |
|---|---|---|---|---|---|---|---|---|
| Minimum: | | | | -1.5 | | | | |
| Maximum: | | 5.0 | 10.0 | 50.0 | | | | |
| Mass | Calc. Mass | mDa | PPM | DBE | i-FIT | Norm | Conf(%) | Formula |
| 442.2353 | 442.2358 | -0.5 | -1.1 | 11.5 | 85.1 | n/a | n/a | C27 H31 N O F3 |

HRMS of [**S7**+H]$^+$ calcd 442.2358; found 442.2353

## (d) Probing the viable nitrogen-containing intermediates

Nitroarene can undergo reduction to sequentially give nitrosoarene, *N*-aryl hydroxylamine, azoxyarene, azoarene, 1,2-diarylhydrazine, and aniline which are all viable reaction intermediate en route to the *N*-trifluoroalkyl aniline product. When nitrobenzene (**N55**) was engaged in the amination, *N*-trifluoroalkyl aniline **110** was formed in 58% yield. The corresponding nitrogen-containing intermediates derived from **N55**, namely nitrosobenzene (**N55-i**), *N*-phenyl hydroxylamine (**N55-ii**), azobenzene (**N55-iii**), azoxybenzene (**N55-iv**), 1,2-diarylhydrazine (**N56-v**), and aniline (**N55-vi**), were then subjected to amination under otherwise identical reaction conditions (**Fig. S5**). Only nitrosobenzene (**N55-i**) and *N*-phenyl hydroxylamine (**N55-ii**) reacted to deliver **110** in 32% and 34% yields, respectively, suggesting that both nitrosoarene and *N*-aryl hydroxylamine are likely the active reaction intermediate to effect amination.

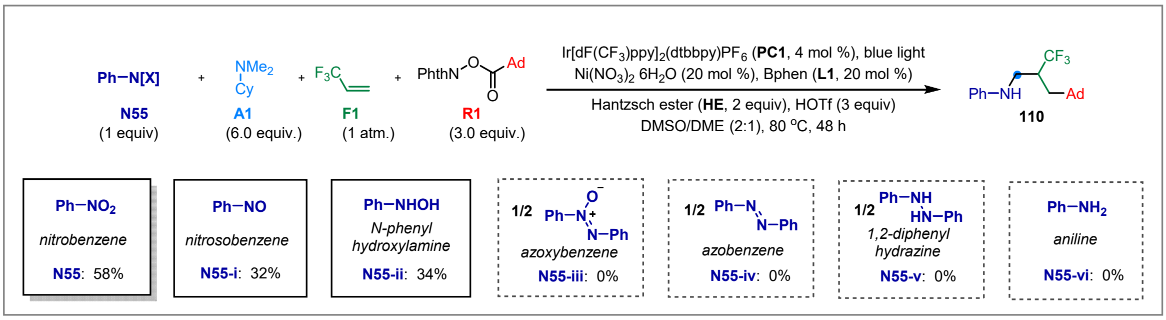


**Fig. S5.** Nitrosoarene and *N*-aryl hydroxylamine are likely the reaction intermediates.

**Reaction Procedure:** An oven-dried, transparent 20 mL Schlenk tube equipped with a stir bar was sequentially charged with nitrogen-containing intermediates (**N55-i** – **N55-vi**, 1.0 equiv., 0.10 mmol), , redox active ester (**R1**, 3.0 equiv., 0.30 mmol), Ir[dF($CF_3$)ppy]$_2$(dtbbpy)$PF_6$ (**PC1**, 4 mol %, 0.0040 mmol), $Ni(NO_3)_2$•$6H_2O$ (20 mol %, 0.020 mmol), bathophenanthroline (BPhen, **L1**, 20 mol %, 0.020 mmol), and Hantzsch ester (diethyl 1,4-dihydro-2,6-dimethyl-3,5-pyridinedicarboxylate, **HE**, 2.0 equiv., 0.20 mmol). Dried dimethyl sulfoxide (DMSO, 2.7 mL) and dried 1,2-dimethoxyethane (DME, 1.3 mL) were then transferred into the tube via syringe. Subsequently, *N*,*N*-dimethylcyclohexylamine (**A1**, 6.0 equiv., 0.60 mmol) and triflic acid (HOTf, 3.0 equiv., 0.30 mmol) were transferred into the tube via syringe. The resulting mixture was degassed via blowing with a balloon filled with 3,3,3-trifluoropropene (**F1**, ~1 L) for 2 min, after which time the tube was quickly

capped with a Teflon screw cap such that it was filled with 3,3,3-trifluoropropene in atmospheric pressure. The reaction mixture was vigorously stirred and irradiated using 30 W blue LEDs (λ = 456–460 nm) for 48 h under the ambient temperature of approximately ~80 ºC (without the use of fans for cooling). At this point, the reaction mixture was diluted with ethyl acetate (~100 mL) and washed with water (~50 mL × 4). The organic fraction was further dried with anhydrous $Na_2SO_4$ and concentrated *in vacuo* with the aid of rotary evaporator. The residue was purified by preparative thin-layer chromatography using using petroleum ether/ethyl acetate (15:1) as an eluent to afford the product **110**.

## (e) Study of *N*-aryl imine and *N*-methyl aniline as reaction intermediates for product formation

We assume that the aminal species **S2**, formed by nitroarene **N14** and *N*,*N*-dimethylcyclohexylamine **A1**, will undergo deamination reaction to produce *N*-aryl imine **S8**. Concurrently, the *N*-aryl *α*-aminoalkyl radical **Rad-iii**, generated from **S2**, is expected to produce *N*-methyl aniline **S9** through HAT with Hantzsch ester (**Fig. S6(a)**). In the control experiment, the reaction of *in-situ* formed imine **S8** based on aniline and paraformaldehyde (9.0 equiv.) with RAE (**R1**) did not lead to the formation of the target compound **14** (**Fig. S6(b)**). The reaction of commercially available **S9** with **R1** also did not afford the target compound **110** (**Fig. S6(c)**), thus precluding the participation of imine **S8** and *N*-alkyl aniline **S9** in the amination reaction. Furthermore, the reaction with 1,3,5-triphenyl-1,3,5-triazine, an alternative precursor of imine **S8**, did not afford the target product under otherwise identical conditions. This observation led us to conclude that **S2** and its subsequent radical form **Rad-v** are most likely the reaction intermediates, facilitating the formation of the desired product through radical-driven processes.

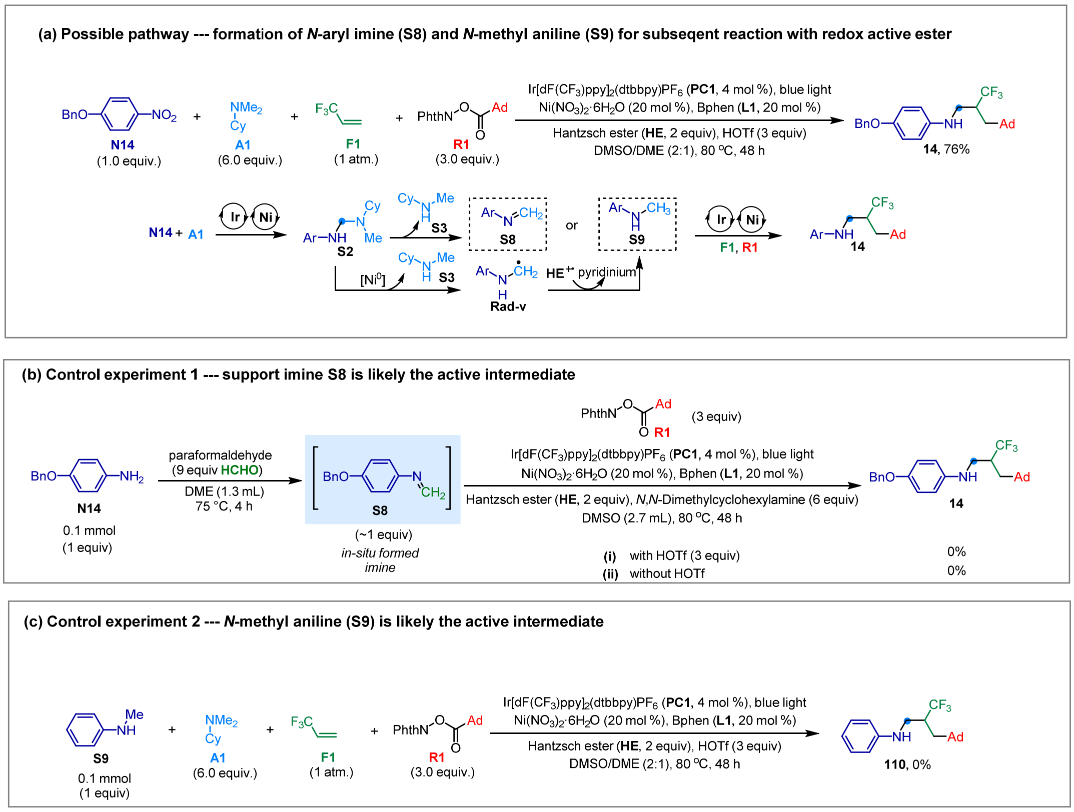


**Fig. S6. S2** are most likely the reaction intermediates without involving the imine **S8** and aniline **S9.**

## Reactions in Fig S6(b)

**Reaction Procedure:** An oven-dried, transparent 20 mL Schlenk tube equipped with a stir bar was sequentially charged with 4-(benzyloxy)aniline (**S8**, 1.0 equiv., 0.10 mmol) and paraformaldehyde (9.0 equiv., 0.9 mmol). The tube was evacuated *in vacuo* and then backfilled with argon for three times, and DME (1.3 mL) was then added via syringe. The resulting mixture was stirred under argon atmosphere at 75 ºC for 4 h, after which time 4-(benzyloxy)aniline was consumed as determined by TLC analysis, indicating the complete conversion to imine. At this point, the tube was charged with redox active ester (**R1**, 3.0 equiv., 0.30 mmol), $Ir[dF(CF_3)ppy]_2(dtbbpy)PF_6$ (**PC1**, 4 mol %, 0.0040 mmol), $Ni(NO_3)_2•6H_2O$ (20 mol %, 0.020 mmol), bathophenanthroline (BPhen, **L1**, 20 mol %, 0.020 mmol), and Hantzsch ester (diethyl 1,4-dihydro-2,6-dimethyl-3,5-pyridinedicarboxylate, **HE**, 2.0 equiv., 0.20 mmol). Dried dimethyl sulfoxide (DMSO, 2.7 mL) was then transferred into the tube via syringe. Subsequently, *N*,*N*-dimethylcyclohexylamine (**A1**, 6.0 equiv., 0.60 mmol) and triflic acid (HOTf, 3.0 equiv., 0.30 mmol) or without HOTf were transferred into the tube via syringe. The resulting mixture was degassed via blowing with a balloon filled with 3,3,3-trifluoropropene (**F1**, ~1 L) for 2 min, after which time the tube was quickly capped with a Teflon screw cap such that it was filled with 3,3,3-trifluoropropene in atmospheric pressure. The reaction mixture was vigorously stirred and irradiated using 30 W blue LEDs ($\lambda$ = 456–460 nm) for 48 h under the ambient temperature of approximately ~80 ºC (without the use of fans for cooling). After the reaction, The product **14** was not formed as determined by TLC analysis.

## Reactions in Fig S6(c)

**Reaction Procedure:** An oven-dried, transparent 20 mL Schlenk tube equipped with a stir bar was sequentially charged with *N*-methylaniline (**S9**, 1.0 equiv., 0.10 mmol), , redox active ester (**R1**, 3.0 equiv., 0.30 mmol), $Ir[dF(CF_3)ppy]_2(dtbbpy)PF_6$ (**PC1**, 4 mol %, 0.0040 mmol), $Ni(NO_3)_2•6H_2O$ (20 mol %, 0.020 mmol), bathophenanthroline (BPhen, **L1**, 20 mol %, 0.020 mmol), and Hantzsch ester (diethyl 1,4-dihydro-2,6-dimethyl-3,5-pyridinedicarboxylate, **HE**, 2.0 equiv., 0.20 mmol).

Dried dimethyl sulfoxide (DMSO, 2.7 mL) and dried 1,2-dimethoxyethane (DME, 1.3 mL) were then transferred into the tube via syringe. Subsequently, *N*,*N*-dimethylcyclohexylamine (**A1**, 6.0 equiv., 0.60 mmol) and triflic acid (HOTf, 3.0 equiv., 0.30 mmol) were transferred into the tube via syringe. The resulting mixture was degassed via blowing with a balloon filled with 3,3,3-trifluoropropene (**F1**, ~1 L) for 2 min, after which time the tube was quickly capped with a Teflon screw cap such that it was filled with 3,3,3-trifluoropropene in atmospheric pressure. The reaction mixture was vigorously stirred and irradiated using 30 W blue LEDs ($\lambda$ = 456–460 nm) for 48 h under the ambient temperature of approximately ~80 ºC (without the use of fans for cooling). After the reaction, the product **110** was not formed as determined by TLC analysis.

## (f) Investigation of the $\alpha$-hydrogen source in *N*-trifluoroalkyl aniline product

We assume that the $\alpha$-amino-trifluoroalkyl radical species (**Rad-vi**) would engage in HAT with Hantzsch ester (**HE**) or its radical cation form (**HE**$^{+\bullet}$), resulting in the formation of *N*-trifluoroalkyl aniline **14**. Consequently, we engaged C4-deuterium-enriched Hantzsch ester (**HE-*d*$_2$**), which serves as deuterium atom source, instead of Hantzsch ester (**HE**), into the amination reaction based on nitroarene **N14**, *N*,*N*-dimethylcyclohexylamine (**A1**) and redox active ester (**R1**). The reaction yielded the conventional product **14**, without incorporating deuterium to afford **14-*d*$_1$** (**Fig. S7(a)**). We also engaged a mixture of *N*,*N*-dimethylcyclohexylamine (**A1,** 3.0 equiv.) and $Et_3N$-$d_{15}$ (4.0 equiv.) instead of *N*,*N*-dimethylcyclohexylamine (**A1**), into the amination reaction based on nitroarene **N14**, and redox active ester (**R1**). The reaction yielded the conventional product **14**, without incorporating deuterium to afford **14-*d*$_1$** (**Fig. S7(b)**). The result implied that **Rad-vi** likely undergoes an inner-sphere electron transfer with low-valent nickel to form a Ni-($\alpha$-amino)trifluoroalkyl complex (**S10**, **Fig. S7(c)**). This complex is then protonated, releasing the target product **14**.

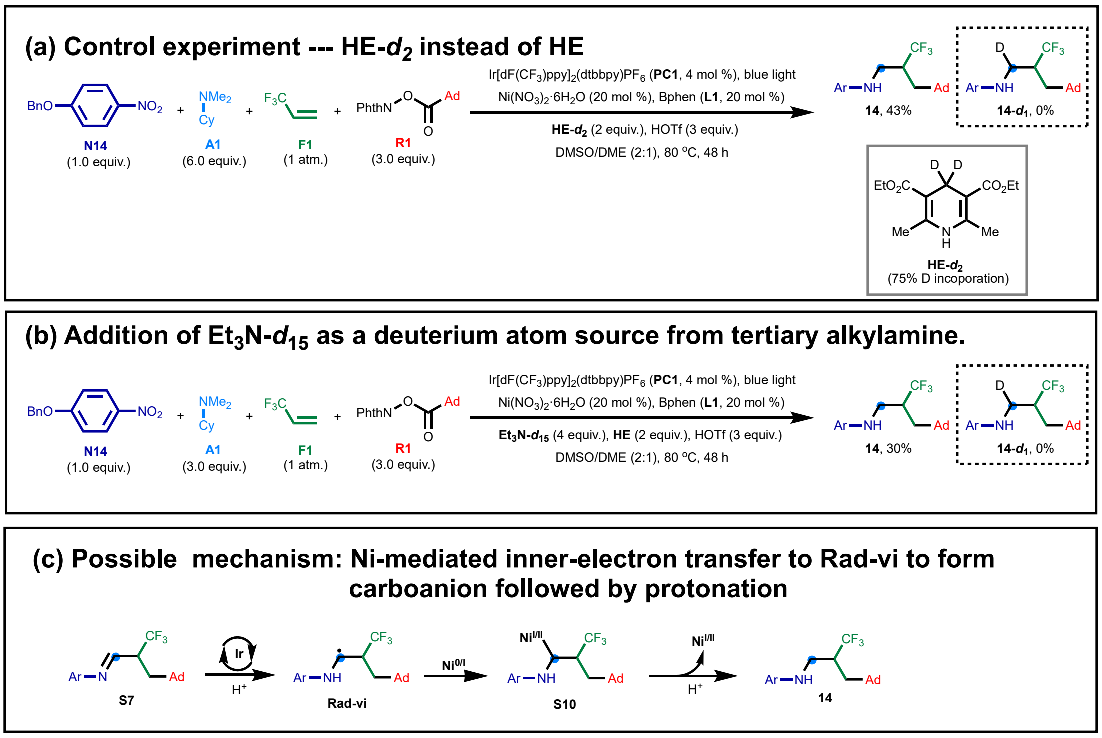


**Fig. S7.** Investigation of the $\alpha$-hydrogen source in *N*-trifluoroalkyl aniline product.

## Reactions in Fig S7(a): HE-$d_2$ instead of HE

**Reaction Procedure:** An oven-dried, transparent 20 mL Schlenk tube equipped with a stir bar was sequentially charged with nitrobenzene (**N14**, 1.0 equiv., 0.10 mmol), , redox active ester (**R1**, 3.0 equiv., 0.30 mmol), Ir[dF($CF_3$)ppy]$_2$(dtbbpy)$PF_6$ (**PC1**, 4 mol %, 0.0040 mmol), Ni($NO_3$)$_2$•6$H_2O$ (20 mol %, 0.020 mmol), bathophenanthroline (BPhen, **L1**, 20 mol %, 0.020 mmol), and **HE-*d*$_2$** (C4-deuterium-enriched Hantzsch ester, 2.0 equiv., 0.20 mmol). Dried dimethyl sulfoxide (DMSO, 2.7 mL) and dried 1,2-dimethoxyethane (DME, 1.3 mL) were then transferred into the tube via syringe. Subsequently, *N*,*N*-dimethylcyclohexylamine (**A1**, 6.0 equiv., 0.60 mmol) and triflic acid (HOTf, 3.0 equiv., 0.30 mmol) were transferred into the tube via syringe. The resulting mixture was degassed via blowing with a balloon filled with 3,3,3-trifluoropropene (**F1**, ~1 L) for 2 min, after which time the tube was quickly capped with a Teflon screw cap such that it was filled with 3,3,3-trifluoropropene in atmospheric pressure. The reaction mixture was vigorously stirred and irradiated using 30 W blue LEDs ($\lambda$ = 456–460 nm) for 48 h under the ambient temperature of approximately ~80 ºC (without the use of fans for cooling). After the reaction, the $\alpha$-H of product **14** was not deuterated based on the ratio of proton integrations by $^1$H NMR spectroscopy (As shown in the figure below). The result suggested that only the conventional product **14** was formed without the formation of deuterated **14-*d*$_1$**.

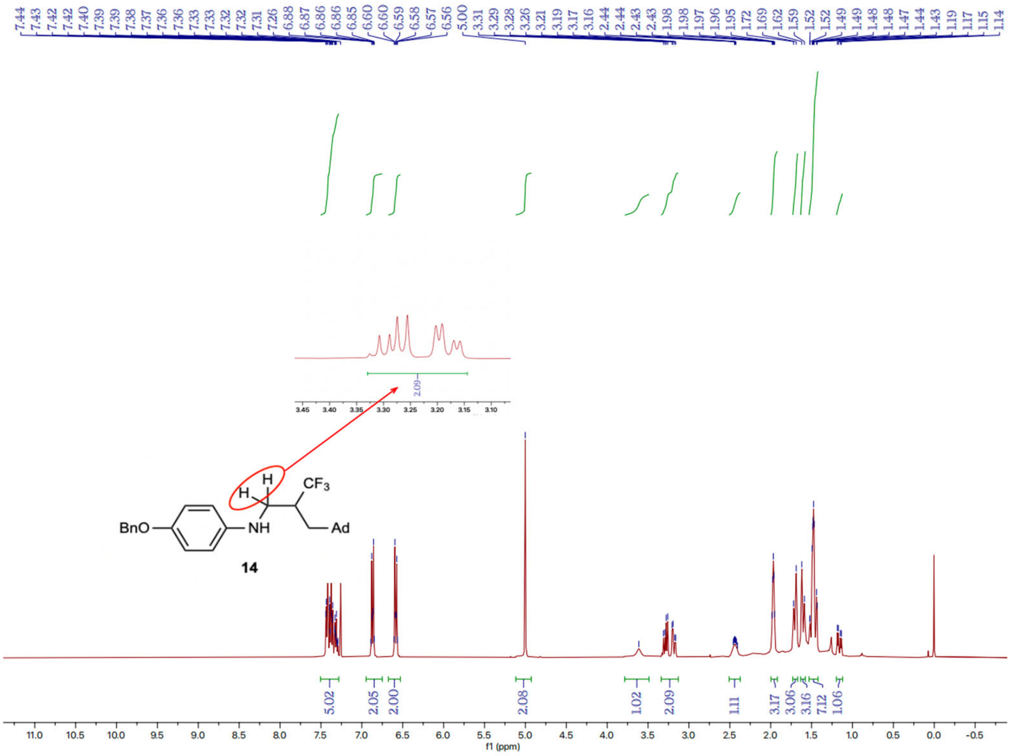

**Preparation of Diethyl2,6-dimethyl-1,4-dihydropyridine-3,5-dicarboxylate-4,4-*$d_2$* (HE-*$d_2$*)**[3]

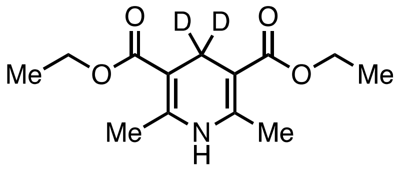


To a 100 mL round-bottom flask equipped with a magnetic stir bar was charged with the ethyl acetoacetate (2.0 equiv., 20 mmol), Formalin-$d_2$ (20% w/w in $D_2O$) (1.0 equiv., 10 mmol), and ethanol (20 mL). To the above solution, ammonium hydroxide (10.0 equiv., 100 mmol) was added slowly. After addition, the system was heated at 70 ºC with stirring. The reaction was monitored by TLC. When the reaction was completed, the crude reaction mixture was allowed to reach room temperature, the solvent was eliminated and a solution of 2 N HCl aqueous solution and dichloromethane were added and the layers were separated. The aqueous phase was extracted with dichloromethane (3 × 100 mL). The combined organic layers were washed with two portions of a saturated solution of $NaHCO_3$, brine and then dried over $MgSO_4$. The reaction mixture was filtered and the filtrate was concentrated *in vacuo*. The residue was purified by column chromatography on silica gel using petroleum ether and ethyl acetate as eluent to afford the **HE-*$d_2$*** as a pale yellow solid (2.10 g, 81%, 75% D-incorporation at $C_4$–H). $^1H$ NMR (400 MHz, $CDCl_3$) δ 5.16 (brs, 1H), 4.16 (q, $J$ = 7.2 Hz, 4H), 2.85 (s, 0.5H), 2.19 (s, 6H), 1.28 (t, $J$ = 7.0 Hz, 6H).

## Reactions in Fig S7(b): Addition of $Et_3N$-*$d_{15}$* as a deuterium atom source from tertiary alkylamine

**Reaction Procedure:** An oven-dried, transparent 20 mL Schlenk tube equipped with a stir bar was sequentially charged with nitrobenzene (**N14**, 1.0 equiv., 0.10 mmol), , redox active ester (**R1**, 3.0 equiv., 0.30 mmol), Ir[dF($CF_3$)ppy]$_2$(dtbbpy)$PF_6$ (**PC1**, 4 mol %, 0.0040 mmol), $Ni(NO_3)_2$•$6H_2O$ (20 mol %, 0.020 mmol), bathophenanthroline (BPhen, **L1**, 20 mol %, 0.020 mmol), and Hantzsch ester (diethyl 1,4-dihydro-2,6-dimethyl-3,5-pyridinedicarboxylate, **HE**, 2.0 equiv., 0.20 mmol). Dried dimethyl sulfoxide (DMSO, 2.7 mL) and dried 1,2-dimethoxyethane (DME, 1.3

mL) were then transferred into the tube via syringe. Subsequently, a mixture of the *N*,*N*-dimethylcyclohexylamine (**A1**, 3.0 equiv., 0.30 mmol) and triethylamine-$d_{15}$ (4 equiv., 0.4 mmol), triflic acid (HOTf, 3.0 equiv., 0.30 mmol) were transferred into the tube via syringe. The resulting mixture was degassed via blowing with a balloon filled with 3,3,3-trifluoropropene (**F1**, ~1 L) for 2 min, after which time the tube was quickly capped with a Teflon screw cap such that it was filled with 3,3,3-trifluoropropene in atmospheric pressure. The reaction mixture was vigorously stirred and irradiated using 30 W blue LEDs ($\lambda$ = 456–460 nm) for 48 h under the ambient temperature of approximately ~80 ºC (without the use of fans for cooling). After the reaction, the $\alpha$-H of product **14** was not deuterated based on the ratio of proton integrations by $^1$H NMR spectroscopy (As shown in the figure below). The result suggested that only the conventional product **14** was formed without the formation of deuterated **14-*d*$_1$**.

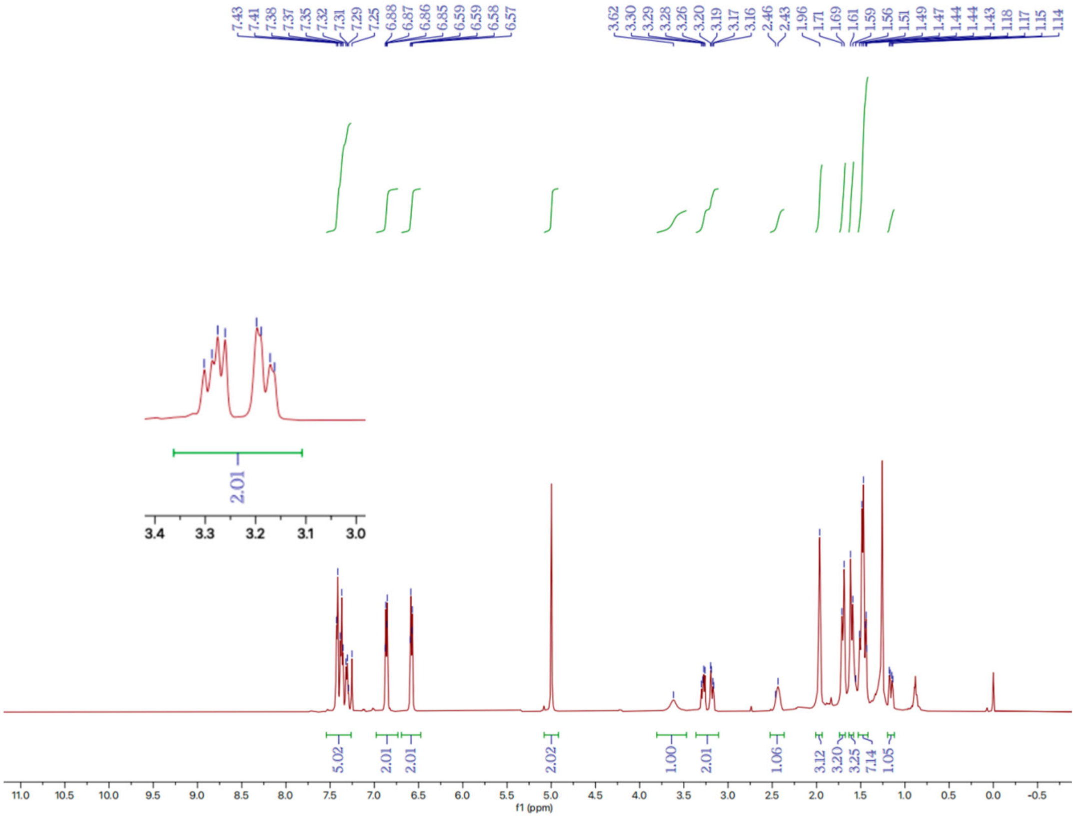

## (g) Light/dark Experiments

To examine whether the reaction is a radical nonchain process, we conducted experiments under alternating periods of irradiation and darkness. The yield of product **14** was determined by crude $^{19}F$ NMR spectroscopic analysis using 4-(trifluoromethyl)biphenyl as an internal standard (**Fig. S8**). The results of light on-off experiments indicated that the reaction proceeds only during the time of irradiation. The reaction likely proceeds by a catalytic process rather than by a radical chain process.

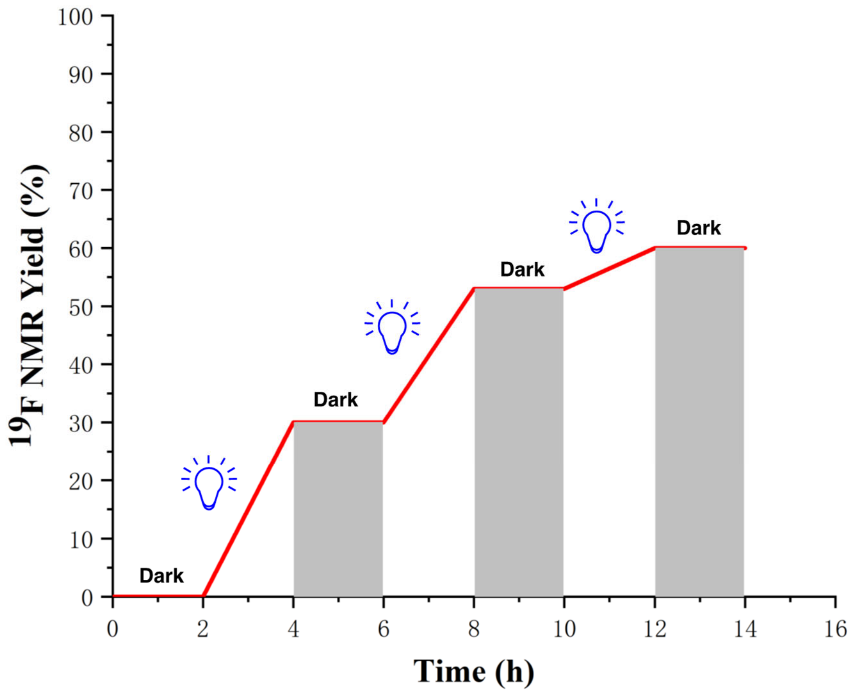


**Fig. S8.** Yield of Light On-Off Experiments in **14**.

**Reaction Procedure:** An oven-dried, transparent 20 mL Schlenk tube equipped with a stir bar was sequentially charged with nitroarene (**N14**, 1.0 equiv., 0.15 mmol), redox active ester (**R1**, 3.0 equiv., 0.45 mmol), Ir[dF($CF_3$)ppy]$_2$(dtbbpy)$PF_6$ (**PC1**, 4 mol %, 0.0060 mmol), $Ni(NO_3)_2$•$6H_2O$ (20 mol %, 0.030 mmol), bathophenanthroline (BPhen, **L1**, 20 mol %, 0.030 mmol), Hantzsch ester (diethyl 1,4-dihydro-2,6-dimethyl-3,5-pyridinedicarboxylate, **HE**, 2.0 equiv., 0.30 mmol), and

4-(trifluoromethyl)biphenyl (1.0 equiv., 0.15 mmol). Dried dimethyl sulfoxide (DMSO, 4.0 mL) and dried 1,2-dimethoxyethane (DME, 2.0 mL) were then transferred into the tube via syringe. Subsequently, *N*,*N*-dimethylcyclohexylamine (**A1**, 6.0 equiv., 0.90 mmol) and triflic acid (HOTf, 3.0 equiv., 0.45 mmol) were transferred into the tube via syringe. The resulting mixture was degassed via blowing with a balloon filled with 3,3,3-trifluoropropene (**F1**, ~1 L) for 2 min, after which time the tube was quickly capped with a Teflon screw cap such that it was filled with 3,3,3-trifluoropropene in atmospheric pressure. A small amount of reaction mixture was extracted from the reaction mixture under the positive pressure of **F1** at the time intervals of 0, 2, 4, 6, 8, 10, 12, and 14 h, during which periods the reaction was performed under irradiation or in darkness (as shown in **Fig. S8**). The yield of product **14** at each time interval was determined by $^{19}$F NMR spectroscopy using 4-(trifluoromethyi)biphenyl as an internal standard.

## (h) Study on the defluorination side-reaction

We hypothesized that some of the trifluoroalkyl radical (formed via the addition of the RAE-derived alkyl radical to 3,3,3-trifluoropropene) might undergo over-reduction, leading to fluoride elimination. Consequently, an excess of saturated aqueous NaOH solution was added to the reaction mixture of the model reaction. Further $^{19}F$ NMR spectroscopic analysis suggests that fluoride is likely formed in the reaction (**Fig. S9**). We surmised that the over-reduction of the trifluoroalkyl radical (**Rad-ii**) via photocatalysis or nickel catalysis is inevitable, leading to the generation of a trifluoroalkyl anion that results in fluorine elimination. However, the addition of excess RAEs and **F1** would ensure sufficient loading of **Rad-ii**, thereby compensating the decomposition **Rad-ii** via defluorination side-reaction and maintaining the reaction productivity.

$^{19}F$ NMR spectrum (471 MHz, $D_2O$) of standard reaction

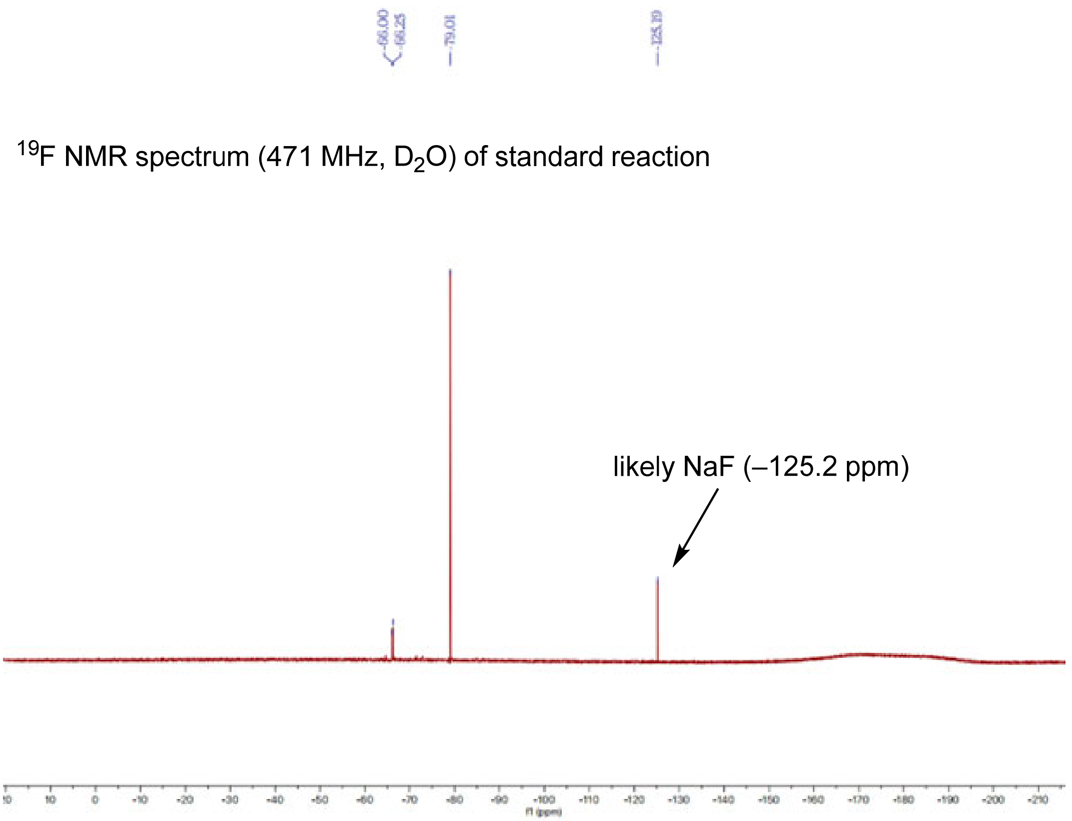


$^{19}F$ NMR spectrum (471 MHz, $D_2O$) of authentic NaF

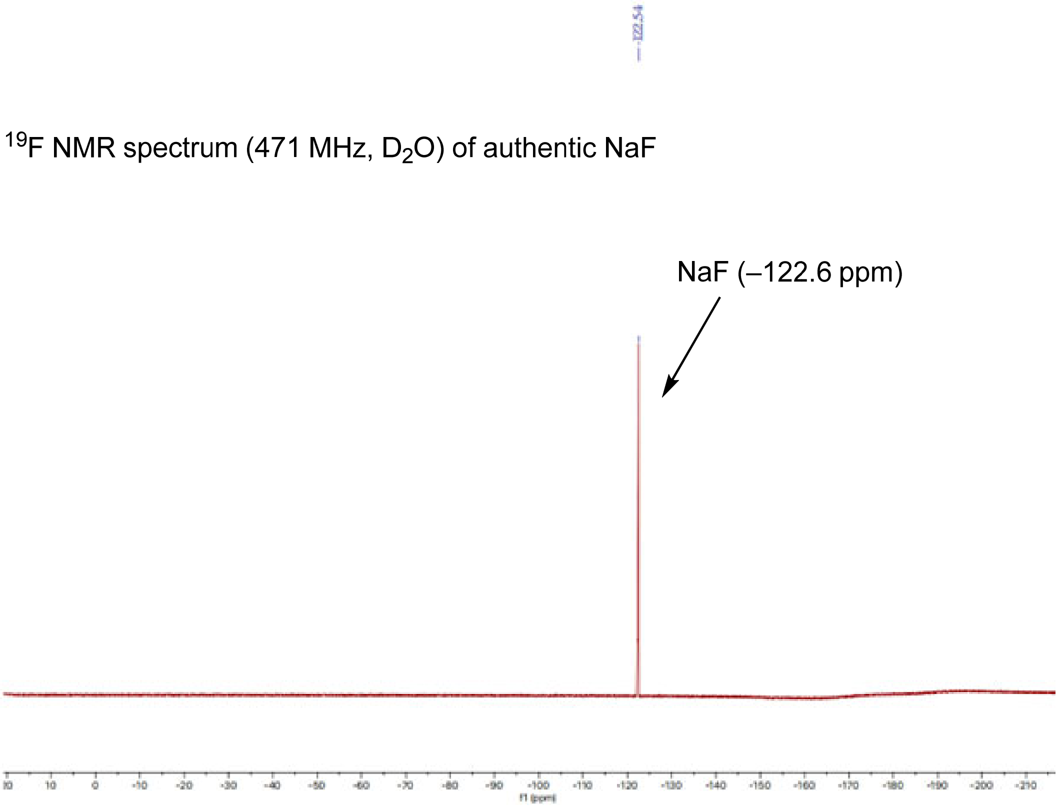


**Fig. S9.** The formation of NaF in the model reaction.

**Reaction Procedure:** An oven-dried, transparent 20 mL Schlenk tube equipped with a stir bar was sequentially charged with nitrobenzene (**N14**, 1.0 equiv., 0.10 mmol), , redox active ester (**R1**, 3.0 equiv., 0.30 mmol), Ir[dF($CF_3$)ppy]$_2$(dtbbpy)$PF_6$ (**PC1**, 4 mol %, 0.0040 mmol), $Ni(NO_3)_2$•$6H_2O$ (20 mol %, 0.020 mmol), bathophenanthroline (BPhen, **L1**, 20 mol %, 0.020 mmol), and Hantzsch ester (diethyl 1,4-dihydro-2,6-dimethyl-3,5-pyridinedicarboxylate, **HE**, 2.0 equiv., 0.20 mmol). Dried dimethyl sulfoxide (DMSO, 2.7 mL) and dried 1,2-dimethoxyethane (DME, 1.3 mL) were then transferred into the tube via syringe. Subsequently, a mixture of the *N*,*N*-dimethylcyclohexylamine (**A1**, 3.0 equiv., 0.30 mmol) and triethylamine-$d_{15}$ (4 equiv., 0.4 mmol), triflic acid (HOTf, 3.0 equiv., 0.30 mmol) were transferred into the tube via syringe. The resulting mixture was degassed via blowing with a balloon filled with 3,3,3-trifluoropropene (**F1**, ~1 L) for 2 min, after which time the tube was quickly capped with a Teflon screw cap such that it was filled with 3,3,3-trifluoropropene in atmospheric pressure. The reaction mixture was vigorously stirred and irradiated using 30 W blue LEDs ($\lambda$ = 456–460 nm) for 48 h under the ambient temperature of approximately ~80 °C (without the use of fans for cooling). After the reaction, saturated aqueous NaOH solution (1 mL) was added, and the reaction mixture was diluted with excess ethyl acetate and stirred vigorously for 1 h at room temperature. This work-up aimed to neutralize the HF and to allow for the formation of NaF in the event that fluoride is eliminated from 3,3,3-trifluoropropene. A small portion of the aqueous layer was extracted and dissolved in $D_2O$ for $^{19}F$ NMR spectroscopic analysis. A $^{19}F$ NMR signal at –125.2 ppm was observed, indicating the likely formation of NaF, as compared to an authentic NaF sample (~ –122.6 ppm in $D_2O$, as shown in **Fig. S9**).

## (i) Cyclic Voltammetry Measurement of N14 in MeCN

Cyclic voltammetry (CV) was performed using a Shanghai Chenhua CHI 760E Electrochemical Workstation. A 3 mm platinum (Pt) wire served as the working electrode (WE), while a coiled Pt wire functioned as both the counter electrode (CE) and quasi-reference electrode (QRE). A solution of 1-benzyloxy-4-nitrobenzene (**N14**, 23 mg) was prepared with a substrate concentration of 0.01 M in anhydrous acetonitrile (10 mL) containing 0.1 M tetrabutylammonium tetrafluoroborate (329 mg) as the supporting electrolyte. The setup was assembled inside a glove box. Data was collected at a sweep rate of 100 mV/s. Following the experiment, the electrochemical potential window was calibrated using ferrocene (Fc) as an internal standard (**Fig. S10**). The redox potential of the ferrocene/ferrocenium ($Fc/Fc^+$) couple was referenced as 0.424 V vs. SCE in MeCN.[13] The half-wave potential for the reduction of **N14** was determined to be –0.88 V vs SCE {$E_{1/2}^{red}$ [*p*-BnOPhNO$_2$/ *p*-BnOPhNO$_2^{-\bullet}$] = –0.88 V vs SCE in MeCN}.

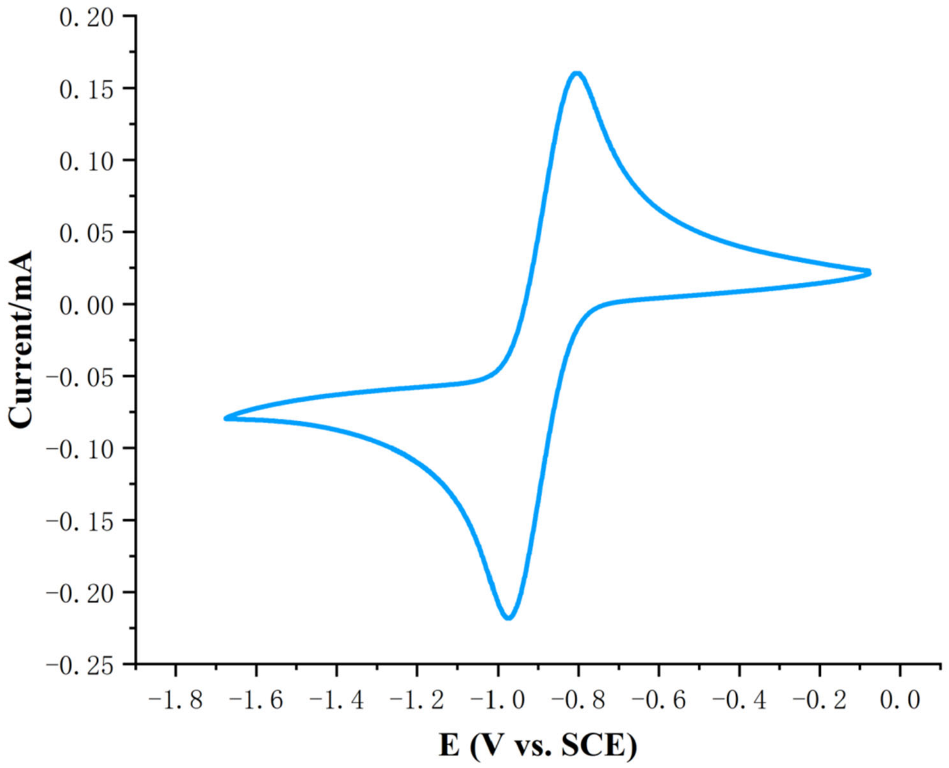


$E_{1/2}^{red}$ [*p*-BnOPhNO$_2$/ *p*-BnOPhNO$_2^{-\bullet}$] = –0.88 V vs SCE in MeCN

**Fig. S10.** Cyclic voltammetry of 1-benzyloxy-4-nitrobenzene (**N14**) in MeCN.

# Substrate scope study

## General procedure for photocatalytic four-component amination using nitroarenes, tertiary alkylamines, 3,3,3-trifluoropropene and redox active esters (General Procedure C):

An oven-dried, transparent 20 mL Schlenk tube equipped with a stir bar was sequentially charged with nitroarene (**N1**–**N59**, 1.0 equiv., 0.15 mmol), redox active ester (**R1**–**R36**, 3.0 equiv., 0.45 mmol), Ir[dF($CF_3$)ppy]$_2$(dtbbpy)$PF_6$ (**PC1**, 4 mol %, 0.0060 mmol), $Ni(NO_3)_2$•$6H_2O$ (20 mol %, 0.030 mmol), bathophenanthroline (BPhen, **L1**, 20 mol %, 0.030 mmol), and Hantzsch ester (diethyl 1,4-dihydro-2,6-dimethyl-3,5-pyridinedicarboxylate, **HE**, 2.0 equiv., 0.30 mmol). Dried dimethyl sulfoxide (DMSO, 4.0 mL) and dried 1,2-dimethoxyethane (DME, 2.0 mL) were then transferred into the tube via syringe. Subsequently, tertiary alkylamine (**A1**, **A7**–**A10**, 6.0 equiv., 0.90 mmol) and triflic acid (HOTf, 3.0 equiv., 0.45 mmol) were transferred into the tube via syringe. The resulting mixture was degassed via blowing with a balloon filled with 3,3,3-trifluoropropene (**F1**, ~1 L) for 2 min, after which time the tube was quickly capped with a Teflon screw cap such that it was filled with 3,3,3-trifluoropropene in atmospheric pressure. The reaction mixture was vigorously stirred and irradiated using 30 W blue LEDs ($\lambda$ = 456–460 nm) for 48 h under the ambient temperature of approximately ~80 $^{o}$C (without the use of fans for cooling). At this point, the reaction mixture was diluted with ethyl acetate (~100 mL) and washed with water (~50 mL × 4). The organic fraction was further dried with anhydrous $Na_2SO_4$ and concentrated in vacuo with the aid of rotary evaporator. The residue was purified by preparative thin-layer chromatography using a mixture of petroleum ether and ethyl acetate as an eluent to afford the *N*-trifluoroalkyl aniline product.

**Variation 1: General procedure for four-component synthesis based on primary and secondary alkyl redox active ester:** Ni(**L6**)$Cl_2$ was added instead of the combination of $Ni(NO_3)_2$•$6H_2O$ and **L1** during the reaction setup when primary and secondary alkyl redox active ester (**R14** – **R29**) were employed as the reaction substates in order to further enhance the product yields.

**Variation 2: General procedure for products 52, 60 and 78:** The reaction mixture was irradiated using Kessil LEDs (456 nm, 40 W × 2) instead of 30 W blue LEDs (λ = 456–460 nm) when **N52** or **R9** or **R27** was employed as the reaction substates in order to further enhance the product yields (as shown below).

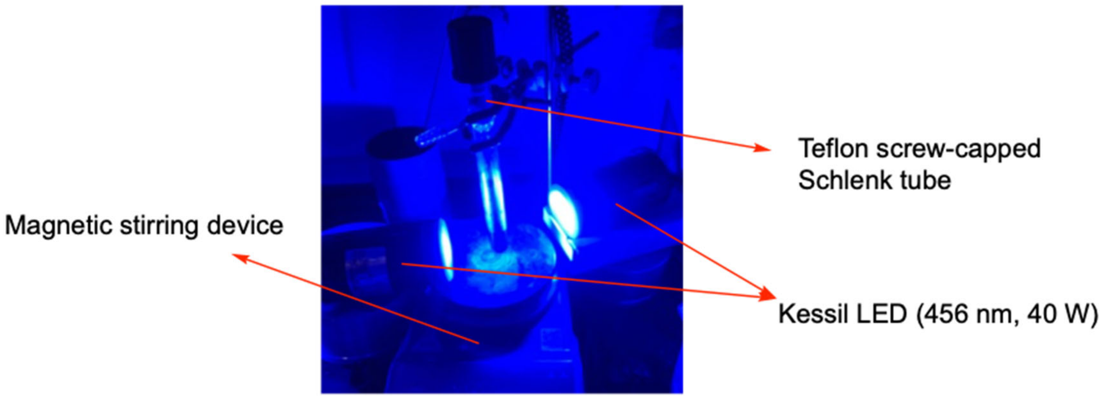


**Variation 3: General procedure for products 84 and 85:** An oven-dried, transparent 20 mL Schlenk tube equipped with a stir bar was sequentially charged with nitroarene (**N14,** 1.0 equiv., 0.15 mmol), redox active ester (**R1**, 3.0 equiv., 0.45 mmol), Ir[dF($CF_3$)ppy]$_2$(dtbbpy)$PF_6$ (**PC1**, 4 mol %, 0.0060 mmol), $Ni(NO_3)_2$•$6H_2O$ (20 mol %, 0.030 mmol), bathophenanthroline (BPhen, **L1**, 20 mol %, 0.030 mmol), and Hantzsch ester (diethyl 1,4-dihydro-2,6-dimethyl-3,5-pyridinedicarboxylate, **HE**, 2.0 equiv., 0.30 mmol). Dried dimethyl sulfoxide (DMSO, 4.0 mL) and dried 1,2-dimethoxyethane (DME, 2.0 mL) were then transferred into the tube via syringe. Subsequently, tertiary alkylamine (**A1**, 6.0 equiv., 0.90 mmol), **F2** or **F3** (20.0 equiv., 3.0 mmol), and triflic acid (HOTf, 3.0 equiv., 0.45 mmol) were transferred into the tube via syringe. The tube was evacuated *in vacuo* and then backfilled with argon for

three times. The reaction mixture was vigorously stirred and irradiated using 30 W blue LEDs (λ = 456–460 nm) for 48 h under the ambient temperature of approximately ~80 ºC (without the use of fans for cooling). At this point, the reaction mixture was diluted with ethyl acetate (~100 mL) and washed with water (~50 mL × 4). The organic fraction was further dried with anhydrous $Na_2SO_4$ and concentrated in vacuo with the aid of rotary evaporator. The residue was purified by preparative thin-layer chromatography using a mixture of petroleum ether and ethyl acetate as an eluent to afford the *N*-trifluoroalkyl aniline product **84** or **85**.

**Variation 4: General procedure for product 86:** An oven-dried, transparent 20 mL Schlenk tube equipped with a stir bar was sequentially charged with nitroarene (**N14**, 1.0 equiv., 0.15 mmol), redox active ester (**R1**, 3.0 equiv., 0.45 mmol), $Ir[dF(CF_3)ppy]_2(dtbbpy)PF_6$ (**PC1**, 4 mol %, 0.0060 mmol), $Ni(NO_3)_2$•$6H_2O$ (20 mol %, 0.030 mmol), bathophenanthroline (BPhen, **L1**, 20 mol %, 0.030 mmol), and Hantzsch ester (diethyl 1,4-dihydro-2,6-dimethyl-3,5-pyridinedicarboxylate, **HE**, 2.0 equiv., 0.30 mmol). Dried dimethyl sulfoxide (DMSO, 4.0 mL) and dried 1,2-dimethoxyethane (DME, 2.0 mL) were then transferred into the tube via syringe. Subsequently, tertiary alkylamine (**A1**, 6.0 equiv., 0.90 mmol) and triflic acid (HOTf, 3.0 equiv., 0.45 mmol) were transferred into the tube via syringe. The resulting mixture was degassed via blowing with a balloon filled with 2,3,3,3-tetrafluoropropene (**F4**, ~1 L) for 2 min, after which time the tube was quickly capped with a Teflon screw cap such that it was filled with 2,3,3,3-tetrafluoropropene in atmospheric pressure. The reaction mixture was vigorously stirred and irradiated using 30 W blue LEDs (λ = 456–460 nm) for 24 h, during which time the proximal temperature was controlled at approximately 40 ºC via cooling with fans. At this point, the reaction mixture was diluted with ethyl acetate (~100 mL) and washed with water (~50 mL × 4). The organic fraction was further dried with anhydrous $Na_2SO_4$ and concentrated in vacuo with the aid of rotary evaporator. The residue was purified by

preparative thin-layer chromatography using a mixture of petroleum ether and ethyl acetate as an eluent to afford the *N*-trifluoroalkyl aniline product.

**Variation 5: General procedure for products 4, 21, 22, 28, 31, 33, 35, 45, 48, 49 and 90:** Using the General Procedure C, LiOH solution (aq, 2 M, 3 mL) was added when the reaction was complete, and the reaction mixture was further heated at ~45 °C in a water bath under an air atmosphere for 1 h (to convert pyridine derivatives derived from Hantzsch esters with similar polarity to the product into carboxylate salts to promote subsequent purification). The reaction mixture was diluted with ethyl acetate (100 mL) and washed with water (50 mL x 4). The organic fraction was further dried with anhydrous $Na_2SO_4$ and concentrated in vacuo with the aid of rotary evaporator. The residue was purified by preparative thin-layer chromatography using a mixture of petroleum ether and ethyl acetate as an eluent to afford the *N*-trifluoroalkyl aniline product.

***N*-(2-((adamantan-1-yl)methyl)-3,3,3-trifluoropropyl)-4-methoxyaniline (1)**

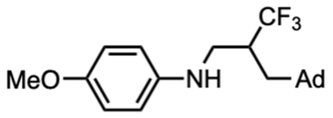


Using the General Procedure C, the title compound was obtained as a yellow viscous oil by preparative TLC using petroleum ether/EtOAc (15:1) as an eluent; $R_f$ = 0.6 (petroleum ether/EtOAc = 10:1); 41.9 mg, 76% yield. **$^1$H NMR** (400 MHz, $CDCl_3$) $\delta$ 6.79 (d, *J* = 8.0 Hz, 2H), 6.59 (d, *J* = 8.0 Hz, 2H), 3.74 (s, 3H), 3.58 (brs, 1H), 3.31 – 3.16 (m, 2H), 2.50 – 2.37 (m, 1H), 1.98 – 1.95 (m, 3H), 1.71 – 1.62 (m, 3H), 1.61 – 1.58 (m, 3H), 1.52 – 1.43 (m, 7H), 1.18 – 1.13 (m, 1H). **$^{13}$C NMR** (101 MHz, $CDCl_3$) $\delta$ 152.6, 141.7, 128.7 (q, $^1J_{CF}$ = 280.0 Hz), 115.1, 114.6, 55.9, 46.1, 42.3, 40.7, 37.1 (q, $^2J_{CF}$ = 24.4 Hz), 36.9, 32.5, 28.5. **$^{19}$F NMR** (376 MHz, $CDCl_3$) $\delta$ -69.20 (s, 3F). **HRMS** (ESI) m/z: $[M+H]^+$ Calcd for $C_{21}H_{29}F_3NO^+$ 368.2201; Found 368.2208.

***N*-(2-((adamantan-1-yl)methyl)-3,3,3-trifluoropropyl)-3-methoxyaniline (2)**

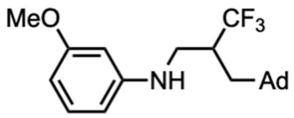

Using the General Procedure C, the title compound was obtained as a yellow viscous oil by preparative TLC using petroleum ether/EtOAc (15:1) as an eluent; $R_f$ = 0.6 (petroleum ether/EtOAc = 10:1); 24.8 mg, 45% yield. **$^1$H NMR** (400 MHz, $CDCl_3$) $\delta$ 7.12 – 7.07 (m, 1H), 6.32 – 6.22 (m, 2H), 6.17 – 6.16 (m, 1H), 3.95 (brs, 1H), 3.77 (s, 3H), 3.34 – 3.20 (m, 2H), 2.49 – 2.41 (m, 1H), 1.98 – 1.95 (m, 3H), 1.72 – 1.69 (m, 3H), 1.63 – 1.59 (m, 3H), 1.53 – 1.44 (m, 7H), 1.18 – 1.13 (m, 1H). **$^{13}$C NMR** (151 MHz, $CDCl_3$) $\delta$ 161.1, 149.0, 130.3, 128.6 (q, $^1J_{CF}$ = 280.9 Hz), 106.2, 103.3, 99.1, 55.3, 44.9, 42.3, 40.6, 37.2 (q, $^2J_{CF}$ = 24.2 Hz), 37.0, 32.5, 28.6. **$^{19}$F NMR** (376 MHz, $CDCl_3$) $\delta$ -69.23 (s, 3F). **HRMS** (ESI) m/z: $[M+H]^+$ Calcd for $C_{21}H_{29}F_3NO^+$ 368.2201; Found 368.2210.

***N*-(2-((adamantan-1-yl)methyl)-3,3,3-trifluoropropyl)-2-methoxyaniline (3)**

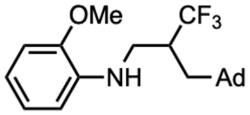

Using the General Procedure C, the title compound was obtained as a yellow viscous oil by preparative TLC using petroleum ether/EtOAc (15:1) as an eluent; $R_f$= 0.6 (petroleum ether/EtOAc = 10:1); 22.3 mg, 40% yield. **$^1$H NMR** (600 MHz, $CDCl_3$) $\delta$ 6.90 – 6.87 (m, 1H), 6.80 – 6.78 (m, 1H), 6.71 – 6.68 (m, 1H), 6.61 – 6.59 (m, 1H), 4.48 (brs, 1H), 3.85 (s, 3H), 3.37 – 3.20 (m, 2H), 2.53 – 2.46 (m, 1H), 1.98 – 1.96 (m, 3H), 1.72 – 1.70 (m, 3H), 1.63 – 1.61 (m, 3H), 1.54 – 1.46 (m, 7H), 1.22 – 1.19 (m, 1H). **$^{13}$C NMR** (151 MHz, $CDCl_3$) $\delta$ 147.1, 137.7, 128.6 (q, $^1J_{CF}$ = 280.9 Hz), 121.4, 117.1, 109.8, 109.7, 55.7, 44.6, 42.3, 40.8, 37.21 (q, $^2J_{CF}$ = 24.2 Hz), 37.0, 32.5, 28.6. **$^{19}$F NMR** (376 MHz, $CDCl_3$) $\delta$ -69.44 (s, 3F). **HRMS** (ESI) m/z: $[M+H]^+$ Calcd for $C_{21}H_{29}F_3NO^+$ 368.2201; Found 368.2210.

**4-((2-((adamantan-1-yl)methyl)-3,3,3-trifluoropropyl)amino)phenyl acetate (4)**

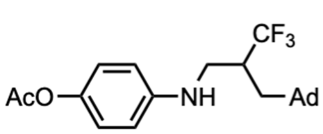

Using the General Procedure C (Variation 5), the title compound was obtained as a pale yellow amorphous solid by preparative TLC using petroleum

ether/EtOAc (10:1) as an eluent; $R_f$= 0.4 (petroleum ether/EtOAc = 10:1); 31.2 mg, 53% yield. **$^1$H NMR** (400 MHz, $CDCl_3$) $\delta$ 6.93 – 6.90 (m, 2H), 6.60 – 6.58 (m, 2H), 3.84 (brs, 1H), 3.33 – 3.19 (m, 2H), 2.48 – 2.40 (m, 1H), 2.26 (s, 3H), 1.98 – 1.96 (m, 3H), 1.73 – 1.69 (m, 3H), 1.63 – 1.53 (m, 3H), 1.50 – 1.44 (m, 7H), 1.19 – 1.14 (m, 1H). **$^{13}$C NMR** (151 MHz, $CDCl_3$) $\delta$ 170.3, 145.6, 142.5, 128.6 (q, $^1J_{CF}$ = 280.9 Hz), 122.4, 113.4, 45.3, 42.3, 40.6, 37.2 (q, $^2J_{CF}$ = 24.2 Hz), 36.9, 32.5, 28.5, 21.2. **$^{19}$F NMR** (376 MHz, $CDCl_3$) $\delta$ -69.25 (s, 3F). **HRMS** (ESI) m/z: $[M+H]^+$ Calcd for $C_{22}H_{29}F_3NO_2^+$ 396.2150; Found 396.2159.

***N*-(2-((adamantan-1-yl)methyl)-3,3,3-trifluoropropyl)-4-(*tert*-butyl)aniline (5)**

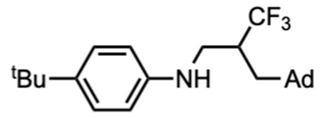


Using the General Procedure C, the title compound was obtained as a pale yellow amorphous solid by preparative TLC using petroleum ether/EtOAc (15:1) as an eluent; $R_f$ = 0.6 (petroleum ether/EtOAc = 10:1); 37.4 mg, 63% yield. **$^1$H NMR** (400 MHz, $CDCl_3$) $\delta$ 7.22 (d, *J* = 8.0 Hz, 2H), 6.58 (d, *J* = 8.0 Hz, 2H), 3.73 (brs, 1H), 3.33 – 3.23 (m, 2H), 2.48 – 2.42 (m, 1H), 1.98 – 1.92 (m, 3H), 1.72 – 1.64 (m, 3H), 1.62 – 1.57 (m, 3H), 1.53 – 1.43 (m, 7H), 1.28 (s, 9H), 1.20 – 1.15 (m, 1H). **$^{13}$C NMR** (151 MHz, $CDCl_3$) $\delta$ 145.3, 140.9, 128.7 (q, $^1J_{CF}$ = 280.9 Hz), 126.2, 112.9, 45.2, 42.3, 40.6, 37.4 (q, $^2J_{CF}$ = 25.7 Hz), 37.0, 34.0, 32.5, 31.7, 28.6. **$^{19}$F NMR** (376 MHz, $CDCl_3$) $\delta$ -69.22 (s, 3F). **HRMS** (ESI) m/z: $[M+H]^+$ Calcd for $C_{24}H_{35}F_3N^+$ 394.2722; Found 394.2730.

***N*-(2-((adamantan-1-yl)methyl)-3,3,3-trifluoropropyl)-4-(methylthio)aniline (6)**

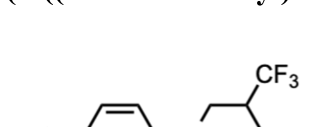


Using the General Procedure C, the title compound was obtained as a red viscous oil by preparative TLC using petroleum ether/EtOAc (15:1) as an eluent; $R_f$= 0.6 (petroleum ether/EtOAc = 10:1); 29.5 mg, 51% yield. **$^1$H NMR** (400 MHz, $CDCl_3$) $\delta$ 7.25 – 7.21 (m, 2H), 6.58 – 6.54 (m, 2H), 3.86 (brs, 1H), 3.34 – 3.20 (m, 2H), 2.48 – 2.39 (m, 4H), 1.98 – 1.95 (m, 3H), 1.72 – 1.69 (m, 3H), 1.63 – 1.57 (m, 3H), 1.52 –

1.43 (m, 7H), 1.18 – 1.13 (m, 1H). **$^{13}$C NMR** (151 MHz, $CDCl_3$) $\delta$ 146.4, 131.5, 128.5 (q, $^1J_{CF}$ = 280.9 Hz), 125.2, 113.7, 44.9, 42.3, 40.6, 37.1 (q, $^2J_{CF}$ = 25.7 Hz), 36.9, 32.5, 28.5, 19.1. **$^{19}$F NMR** (376 MHz, $CDCl_3$) $\delta$ -69.22 (s, 3F). **HRMS** (ESI) m/z: $[M+H]^+$ Calcd for $C_{21}H_{29}F_3NS^+$ 384.1973; Found 384.1979.

***N*-(2-((adamantan-1-yl)methyl)-3,3,3-trifluoropropyl)-4-(phenylthio)aniline (7)**

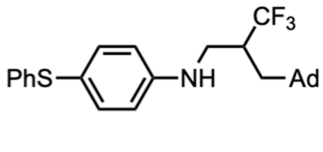


Using the General Procedure C, the title compound was obtained as a pale yellow amorphous solid by preparative TLC using petroleum ether/EtOAc (15:1) as an eluent; $R_f$= 0.6 (petroleum ether/EtOAc = 10:1); 28.1 mg, 42% yield. **$^1$H NMR** (400 MHz, $CDCl_3$) $\delta$ 7.38 – 7.34 (m, 2H), 7.23 – 7.18 (m, 2H), 7.13 – 7.07 (m, 3H), 6.63 – 6.59 (m, 2H), 4.04 (brs, 1H), 3.39 – 3.23 (m, 2H), 2.54 – 2.41 (m, 1H), 2.00 – 1.96 (m, 3H), 1.73 – 1.70 (m, 3H), 1.64 – 1.54 (m, 3H), 1.53 – 1.44 (m, 7H), 1.20 – 1.14 (m, 1H). **$^{13}$C NMR** (151 MHz, $CDCl_3$) $\delta$ 148.1, 140.0, 136.5, 129.46, 128.5 (q, $^1J_{CF}$ = 279.4 Hz), 127.3, 125.3, 119.7, 113.8, 44.6, 42.4, 40.6, 37.1 (q, $^2J_{CF}$ = 24.2 Hz), 36.9, 32.5, 28.6. **$^{19}$F NMR** (376 MHz, $CDCl_3$) $\delta$ -69.24 (s, 3F). **HRMS** (ESI) m/z: $[M+H]^+$ Calcd for $C_{26}H_{31}F_3NS^+$ 446.2129; Found 446.2135.

***N*-(2-((adamantan-1-yl)methyl)-3,3,3-trifluoropropyl)-4'-methoxy-[1,1'-biphenyl] -4-amine (8)**

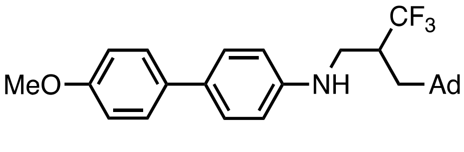


Using the General Procedure C, the title compound was obtained as a pale yellow amorphous solid by preparative TLC using petroleum ether/EtOAc (15:1) as an eluent; $R_f$= 0.6 (petroleum ether/EtOAc = 10:1); 30.0 mg, 45% yield. **$^1$H NMR** (400 MHz, $CDCl_3$) $\delta$ 7.52 – 7.40 (m, 4H), 6.96 – 6.93 (m, 2H), 6.71 – 6.67 (m, 2H), 3.94 (brs, 1H), 3.84 (s, 3H), 3.39 – 3.27 (m, 2H), 2.52 – 2.46 (m, 1H), 2.00 – 1.98 (m, 3H), 1.73 – 1.71 (m, 3H), 1.67 – 1.55 (m, 3H), 1.53 – 1.47 (m, 7H), 1.22 – 1.17 (m, 1H). **$^{13}$C NMR** (101 MHz, $CDCl_3$) $\delta$ 158.5, 146.54, 134.0, 130.8, 128.6 (q, $^1J_{CF}$ = 280.8 Hz), 127.8, 127.5, 114.3, 113.4, 55.5, 45.0, 42.3,

40.6, 37.2 (q, $^2J_{CF}$ = 24.2 Hz), 36.9, 32.5, 28.6. **$^{19}$F NMR** (376 MHz, $CDCl_3$) $\delta$ -69.19 (s, 3F). **HRMS** (ESI) m/z: $[M+H]^+$ Calcd for $C_{27}H_{33}F_3NO^+$ 444.2514; Found 444.2516.

***N*-(3,3,3-trifluoro-2-(methoxymethyl)propyl)-[1,1'-biphenyl]-4-amine (9)**

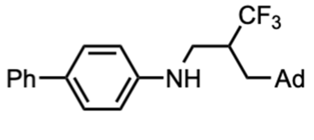


Using the General Procedure C, the title compound was obtained as a white amorphous solid by preparative TLC using petroleum ether/EtOAc (15:1) as an eluent; $R_f$= 0.6 (petroleum ether/EtOAc = 10:1); 25.7 mg, 42% yield. **$^1$H NMR** (400 MHz, $CDCl_3$) $\delta$ 7.55 – 7.53 (m, 2H), 7.47 – 7.45 (m, 2H), 7.41 – 7.37 (m, 2H), 7.28 – 7.24 (m, 1H), 6.70 -6.67 (m, 2H), 3.93 (brs, 1H), 3.40 – 3.26 (m, 2H), 2.52 – 2.43 (m, 1H), 1.98 – 1.96 (m, 3H), 1.72 – 1.69 (m, 3H), 1.63 – 1.59 (m, 3H), 1.54 – 1.46 (m, 7H), 1.21 – 1.16 (m, 1H). **$^{13}$C NMR** (151 MHz, $CDCl_3$) $\delta$ 147.0, 141.2, 131.0, 130.6 (q, $^1J_{CF}$ = 280.9 Hz), 128.8, 128.2, 126.5, 126.3, 113.3, 44.9, 42.3, 40.6, 37.2 (q, $^2J_{CF}$ = 24.2 Hz), 37.0, 32.5, 28.6. **$^{19}$F NMR** (376 MHz, $CDCl_3$) $\delta$ -69.18 (s, 3F). **HRMS** (ESI) m/z: $[M+H]^+$ Calcd for $C_{26}H_{31}F_3N^+$ 414.2409; Found 414.2414.

***N*-(2-((adamantan-1-yl)methyl)-3,3,3-trifluoropropyl)-4-(difluoromethoxy)aniline (10)**

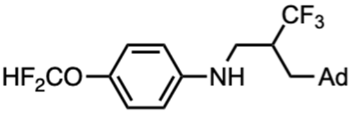


Using the General Procedure C, the title compound was obtained as a yellow viscous oil by preparative TLC using petroleum ether/EtOAc (15:1) as an eluent; $R_f$ = 0.6 (petroleum ether/EtOAc = 10:1); 30.9 mg, 51% yield. **$^1$H NMR** (400 MHz, $CDCl_3$) $\delta$ 7.00 – 6.93 (m, 2H), 6.59 – 6.54 (m, 2H), 6.38 (t, $J$ = 72.0 Hz, 1H), 3.83 (brs, 1H), 3.33 – 3.19 (m, 2H), 2.50 – 2.38 (m, 1H), 1.98 – 1.96 (m, 3H), 1.73 – 1.69 (m, 3H), 1.62 – 1.59 (m, 3H), 1.53 – 1.44 (m, 7H), 1.18 – 1.13 (m, 1H). **$^{13}$C NMR** (151 MHz, $CDCl_3$) $\delta$ 145.6, 142.9, 128.6 (q, $^1J_{CF}$ = 279.4 Hz), 121.7, 116.6 (t, $^1J_{CF}$ = 259.7 Hz), 113.7, 45.3, 42.3, 40.6, 37.2 (q, $^2J_{CF}$ =

24.2 Hz), 36.9, 32.5, 28.6. **$^{19}$F NMR** (376 MHz, $CDCl_3$) $\delta$ -69.25 (s, 3F), -80.10 (s, 2F). **HRMS** (ESI) m/z: $[M+H]^+$ Calcd for $C_{21}H_{27}F_5NO^+$ 404.2013; Found 404.2022.

***N*-(2-((adamantan-1-yl)methyl)-3,3,3-trifluoropropyl)-4-(trifluoromethoxy)-aniline (11)**

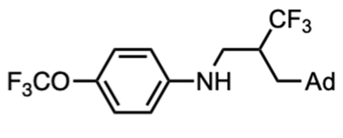


Using the General Procedure C, the title compound was obtained as a yellow viscous oil by preparative TLC using petroleum ether/EtOAc (15:1) as an eluent; $R_f$= 0.6 (petroleum ether/EtOAc = 10:1); 32.3 mg, 51% yield. **$^1$H NMR** (400 MHz, $CDCl_3$) $\delta$ 7.06 (d, $J$ = 8.0 Hz, 2H), 6.57 (d, $J$ = 8.0 Hz, 2H), 3.91 (brs, 1H), 3.35 – 3.19 (m, 2H), 2.49 – 2.41 (m, 1H), 1.99 – 1.97 (m, 3H), 1.73 – 1.70 (m, 3H), 1.63 – 1.60 (m, 3H), 1.53 – 1.44 (m, 7H), 1.19 – 1.14 (m, 1H). **$^{13}$C NMR** (151 MHz, $CDCl_3$) $\delta$ 146.4, 141.0, 128.6 (q, $^1J_{CF}$ = 279.4 Hz), 122.7, 120.9 (q, $^1J_{CF}$ = 256.7 Hz), 113.4, 45.1, 40.6, 37.2 (q, $^2J_{CF}$ = 24.2 Hz), 36.9, 32.5, 28.6. **$^{19}$F NMR** (376 MHz, $CDCl_3$) $\delta$ -58.50 (s, 3F), -69.26 (s, 3F). **HRMS** (ESI) m/z: $[M+H]^+$ Calcd for $C_{21}H_{26}F_6NO^+$ 422.1919; Found 422.1927.

***N*-(2-((adamantan-1-yl)methyl)-3,3,3-trifluoropropyl)-4-chloroaniline (12)**

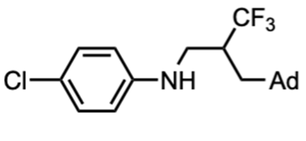


Using the General Procedure C, the title compound was obtained as a yellow viscous oil by preparative TLC using petroleum ether/EtOAc (15:1) as an eluent; $R_f$= 0.6 (petroleum ether/EtOAc = 10:1); 18.7 mg, 34% yield. **$^1$H NMR** (400 MHz, $CDCl_3$) $\delta$ 7.14 (d, $J$ = 8.0 Hz, 2H), 6.53 (d, $J$ = 8.0 Hz, 2H), 3.86 (brs, 1H), 3.33 – 3.18 (m, 2H), 2.49 – 2.38 (m, 1H), 1.99 – 1.96 (m, 3H), 1.73 – 1.69 (m, 3H), 1.63 – 1.58 (m, 3H), 1.53 – 1.43 (m, 7H), 1.17 – 1.12 (m, 1H). **$^{13}$C NMR** (101 MHz, $CDCl_3$) $\delta$ 146.2, 129.3, 128.5 (q, $^1J_{CF}$ = 280.8 Hz), 122.6, 114.1, 45.0 (q, $^3J_{CF}$ = 3.0 Hz), 42.3, 40.6, 37.0 (q, $^2J_{CF}$ = 25.3 Hz), 36.9, 32.5, 28.5. **$^{19}$F NMR** (376 MHz, $CDCl_3$) $\delta$ -69.24 (s, 3F). **HRMS** (ESI) m/z: $[M+H]^+$ Calcd for $C_{20}H_{26}ClF_3N^+$ 372.1706; Found 372.1713.

***N*-(2-((adamantan-1-yl)methyl)-3,3,3-trifluoropropyl)-4-fluoroaniline (13)**

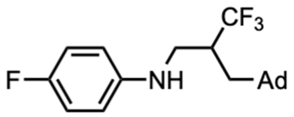

Using the General Procedure C, the title compound was obtained as a yellow viscous oil by preparative TLC using petroleum ether/EtOAc (15:1) as an eluent; $R_f$ = 0.6 (petroleum ether/EtOAc = 10:1); 27.2 mg, 51% yield. **$^1$H NMR** (400 MHz, $CDCl_3$) $\delta$ 6.93 – 6.88 (m, 2H), 6.57 – 6.54 (m, 2H), 3.78 (brs, 1H), 3.32 – 3.17 (m, 2H), 2.49 – 2.40 (m, 1H), 1.99 – 1.96 (m, 3H), 1.72 – 1.69 (m, 3H), 1.62 – 1.57 (m, 3H), 1.52 – 1.44 (m, 7H), 1.18 – 1.13 (m, 1H). **$^{13}$C NMR** (101 MHz, $CDCl_3$) $\delta$ 156.2 (d, $^1J_{CF}$ = 236.3 Hz), 143.9 (d, $^4J_{CF}$ = 2.0 Hz), 128.6 (q, $^1J_{CF}$ = 280.8 Hz), 115.9 (d, $^2J_{CF}$ = 22.2 Hz), 114.1 (d, $^3J_{CF}$ = 7.1 Hz), 45.7, 42.3, 40.6, 37.1 (q, $^2J_{CF}$ = 25.3 Hz), 36.9, 32.5, 28.5. **$^{19}$F NMR** (376 MHz, $CDCl_3$) $\delta$ -69.25 (s, 3F), -127.35 (s, 1F). **HRMS** (ESI) m/z: $[M+H]^+$ Calcd for $C_{20}H_{26}F_4N^+$ 356.2001; Found 356.2010.

***N*-(2-((adamantan-1-yl)methyl)-3,3,3-trifluoropropyl)-4-(benzyloxy)aniline (14)**

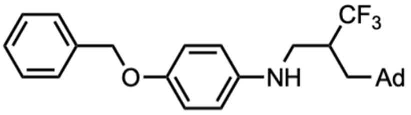

**(a) 0.15 mmol scale:** Using the General Procedure C, the title compound was obtained as a brown crystalline solid by preparative TLC using petroleum ether/EtOAc (15:1) as an eluent; $R_f$= 0.6 (petroleum ether/EtOAc = 10:1); 50.6 mg, 76% yield. **$^1$H NMR** (400 MHz, $CDCl_3$) $\delta$ 7.50 – 7.35 (m, 5H), 6.96 – 6.92 (m, 2H), 6.67 – 6.62 (m, 2H), 5.05 (s, 2H), 3.66 (brs, 1H), 3.38 – 3.22 (m, 2H), 2.56 – 2.45 (m, 1H), 2.05 – 2.02 (m, 3H), 1.79 – 1.76 (m, 3H), 1.70 – 1.64 (m, 3H), 1.60 – 1.51 (m, 7H), 1.25 – 1.20 (m, 1H). **$^{13}$C NMR** (101 MHz, $CDCl_3$) $\delta$ 151.7, 142.0, 137.6, 128.7 (q, $^1J_{CF}$ = 281.8 Hz), 128.6, 127.9, 127.5, 116.2, 114.4, 70.8, 45.9, 42.2, 40.6, 37.05 (q, $^2J_{CF}$ = 25.3 Hz), 36.9, 32.4, 28.5. **$^{19}$F NMR** (376 MHz, $CDCl_3$) $\delta$ -69.07 (s, 3F). **HRMS** (ESI) m/z: $[M+H]^+$ Calcd for $C_{27}H_{33}F_3NO^+$ 444.2514; Found 444.2514.

**(b) 5.0 mmol scale (Gram-scale synthesis):**

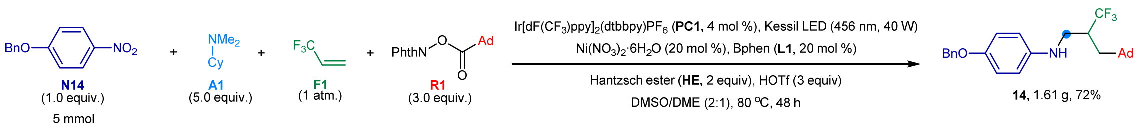

An oven-dried, transparent 500 mL Schlenk tube equipped with a stir bar was sequentially charged with nitroarene (**N14**, 1.0 equiv., 5.0 mmol, 1.15 g), redox active ester (**R1**, 3.0 equiv., 15.0 mmol, 4.90 g), Ir[dF($CF_3$)ppy]$_2$(dtbbpy)$PF_6$ (**PC1**, 4 mol %, 0.2 mmol, 225.38 mg), $Ni(NO_3)_2$•$6H_2O$ (20 mol %, 1.0 mmol, 290.79 mg), bathophenanthroline (BPhen, **L1**, 20 mol %, 1.0 mmol, 332.40 mg), and Hantzsch ester (diethyl 1,4-dihydro-2,6-dimethyl-3,5-pyridinedicarboxylate, **HE**, 2.0 equiv., 10 mmol, 2.60 g). Dried dimethyl sulfoxide (DMSO, 135 mL) and dried 1,2-dimethoxyethane (DME, 65 mL) were then transferred into the tube via syringe. Subsequently, *N*,*N*-dimethylcyclohexylamine (**A1**, 6.0 equiv., 30 mmol, 4.50 mL) and triflic acid (HOTf, 3.0 equiv., 15 mmol, 1.50 mL) were transferred into the tube via syringe. The resulting mixture was degassed via blowing with two balloon filled with 3,3,3-trifluoropropene (**F1**, ~2 L) for 10 min, after which time the tube was quickly capped with a Teflon screw cap such that it was filled with 3,3,3-trifluoropropene in atmospheric pressure. The reaction mixture was vigorously stirred and irradiated using Kessil LEDs (456 nm, 40 W × 2) for 96 h under the ambient temperature of approximately ~80 ºC (without the use of fans for cooling). At this point, the reaction mixture was diluted with ethyl acetate (~500 mL) and washed with water (~100 mL × 4). The organic fraction was further dried with anhydrous $Na_2SO_4$ and concentrated in vacuo with the aid of rotary evaporator. The residue was purified by column chromatography using the silica gel using petroleum ether and ethyl acetate (20:1) as an eluent to afford the *N*-trifluoroalkyl aniline product **14** (1.61 g, 72%) (The experimental setup was shown below).

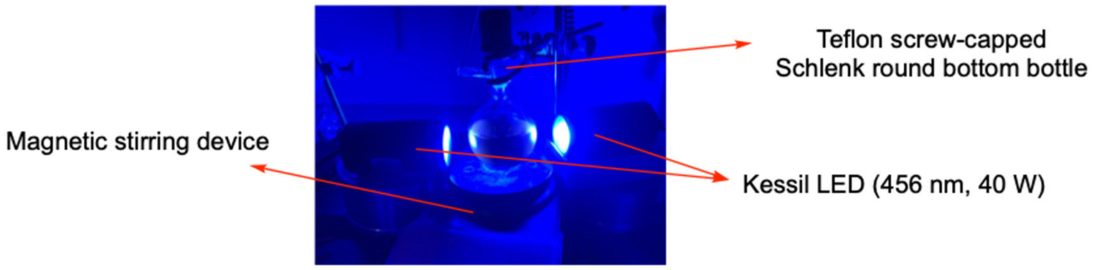


**N-(2-((adamantan-1-yl)methyl)-3,3,3-trifluoropropyl)-4-((4-(*tert*-butyl)benzyl)-oxy)aniline (15)**

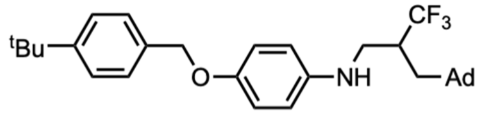

Using the General Procedure C, the title compound was obtained as a yellow amorphous solid by preparative TLC using petroleum ether/EtOAc (15:1) as an eluent; R*f*= 0.6 (petroleum ether/EtOAc = 10:1); 41.2 mg, 55% yield. **$^1$H NMR** (400 MHz, $CDCl_3$) $\delta$ 7.43 – 7.37 (m, 4H), 6.89 (d, $J$ = 8.0 Hz, 2H), 6.60 (d, $J$ = 8.0 Hz, 2H), 4.98 (s, 2H), 3.86 (brs, 1H), 3.33 – 3.18 (m, 2H), 2.52 – 2.40 (m, 1H), 2.00 – 1.97 (m, 3H), 1.74 – 1.71 (m, 3H), 1.65 – 1.59 (m, 3H), 1.55 – 1.45 (m, 7H), 1.34 (s, 9H), 1.20 – 1.15 (m, 1H). **$^{13}$C NMR** (101 MHz, $CDCl_3$) $\delta$ 152.0, 151.0, 141.9, 134.6, 128.7 (q, $^1J_{CF}$ = 280.8 Hz), 127.6, 125.6, 116.2, 114.6, 70.7, 46.0, 42.3, 40.7, 37.2 (q, $^2J_{CF}$ = 24.2 Hz), 36.9, 34.7, 32.5, 31.5, 28.6. **$^{19}$F NMR** (376 MHz, $CDCl_3$) $\delta$ -69.19 (s, 3F). **HRMS** (ESI) m/z: $[M+H]^+$ Calcd for $C_{31}H_{41}F_3NO^+$ 500.3140; Found 500.3147

***N*-(2-((adamantan-1-yl)methyl)-3,3,3-trifluoropropyl)-4-((4-fluorobenzyl)oxy)-aniline (16)**

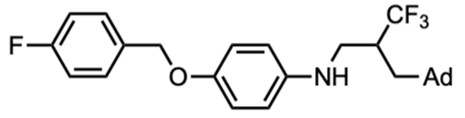

Using the General Procedure C, the title compound was obtained as a brown amorphous solid by preparative TLC using petroleum ether/EtOAc (15:1) as an eluent; R*f*= 0.6 (petroleum ether/EtOAc = 10:1); 47.7 mg, 67% yield. **$^1$H NMR** (400 MHz, $CDCl_3$) $\delta$ 7.42 – 7.37 (m, 2H), 7.09 – 7.04 (m, 2H), 6.88 – 6.82 (m, 2H), 6.61 – 6.57 (m, 2H), 4.96 (s, 2H), 3.52 (brs, 1H), 3.31 – 3.17 (m, 2H), 2.51 – 2.34 (m, 1H), 2.00 – 1.94 (m, 3H), 1.73 – 1.67 (m, 3H), 1.66 – 1.57 (m, 3H), 1.54 – 1.45 (m, 7H), 1.20 – 1.15 (m, 1H). **$^{13}$C NMR** (101 MHz, $CDCl_3$) $\delta$ 163.7, 161.3, 151.5, 142.1, 133.3 (d, $^4J_{CF}$ = 3.0 Hz), 129.4 (d, $^3J_{CF}$ = 8.1 Hz), 128.7 (q, $^1J_{CF}$ = 280.8 Hz), 115.5 (d, $^2J_{CF}$ = 21.2 Hz), 115.4 (d, $^1J_{CF}$ = 188.9 Hz), 70.3, 45.9, 42.3, 40.6, 37.1 (q, $^2J_{CF}$ = 24.2 Hz), 36.9, 32.5, 28.5. **$^{19}$F NMR** (376 MHz, $CDCl_3$) $\delta$ -69.20 (s, 3F), -114.51 (s, 1F). **HRMS** (ESI) m/z: $[M+H]^+$ Calcd for $C_{27}H_{32}F_4NO^+$ 462.2420; Found 462.2423.

**N-(2-((adamantan-1-yl)methyl)-3,3,3-trifluoropropyl)-4-((4-chlorobenzyl)oxy)-aniline (17)**

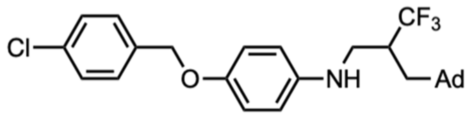


Using the General Procedure C, the title compound was obtained as a pale yellow amorphous solid by preparative TLC using petroleum ether/EtOAc (15:1) as an eluent; $R_f$= 0.6 (petroleum ether/EtOAc = 10:1); 47.9 mg, 67% yield. **$^1$H NMR** (400 MHz, $CDCl_3$) $\delta$ 7.38 – 7.33 (m, 4H), 6.87 – 6.83 (m, 2H), 6.61 – 6.57 (m, 2H), 4.97 (s, 2H), 3.62 (brs, 1H), 3.34 – 3.17 (m, 2H), 2.53 – 2.38 (m, 1H), 1.99 – 1.93 (m, 3H), 1.73 – 1.70 (m, 3H), 1.66 – 1.58 (m, 3H), 1.56 – 1.44 (m, 7H), 1.19 – 1.14 (m, 1H). **$^{13}$C NMR** (101 MHz, $CDCl_3$) $\delta$ 151.4, 142.2, 136.2, 133.7, 128.9, 128.8, 128.7 (q, $^1J_{CF}$ = 281.8 Hz), 116.4, 114.5, 70.2, 46.0, 42.3, 40.7, 37.1 (q, $^2J_{CF}$ = 24.2 Hz), 36.9, 32.5, 28.5. **$^{19}$F NMR** (376 MHz, $CDCl_3$) $\delta$ -69.20 (s, 3F). **HRMS** (ESI) m/z: $[M+H]^+$ Calcd for $C_{27}H_{32}ClF_3NO^+$ 478.2125; Found 478.2131.

**N-(2-((adamantan-1-yl)methyl)-3,3,3-trifluoropropyl)-4-((3-chlorobenzyl)oxy)-aniline (18)**

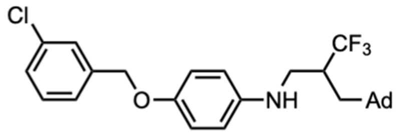


Using the General Procedure C, the title compound was obtained as a pale yellow amorphous solid by preparative TLC using petroleum ether/EtOAc (15:1) as an eluent; $R_f$= 0.6 (petroleum ether/EtOAc = 10:1); 47.1 mg, 66% yield. **$^1$H NMR** (400 MHz, $CDCl_3$) $\delta$ 7.44 – 7.30 (m, 4H), 6.89 – 6.84 (m, 2H), 6.64 – 6.57 (m, 2H), 4.98 (s, 2H), 3.63 (brs, 1H), 3.34 – 3.17 (m, 2H), 2.49 – 2.39 (m, 1H), 2.02 – 1.97 (m, 3H), 1.73 – 1.70 (m, 3H), 1.66 – 1.59 (m, 3H), 1.53 – 1.45 (m, 7H), 1.20 – 1.15 (m, 1H). **$^{13}$C NMR** (101 MHz, $CDCl_3$) $\delta$ 151.4, 142.3, 139.8, 134.6, 129.9, 128.7 (q, $^1J_{CF}$ = 282.8 Hz), 128.0, 127.6, 125.5, 116.4, 114.5, 70.1, 45.9, 42.3, 40.7, 37.1 (q, $^2J_{CF}$ = 24.2 Hz), 36.9, 32.5, 28.6. **$^{19}$F NMR** (376 MHz, $CDCl_3$) $\delta$ -69.19 (s, 3F). **HRMS** (ESI) m/z: $[M+H]^+$ Calcd for $C_{27}H_{32}ClF_3NO^+$ 478.2125; Found 478.2133.

***N*-(2-((adamantan-1-yl)methyl)-3,3,3-trifluoropropyl)-4-((4-(trifluoromethoxy)-benzyl)oxy)aniline (19)**

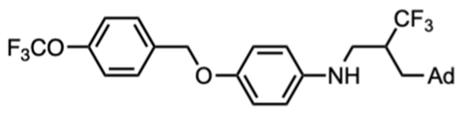

Using the General Procedure C, the title compound was obtained as a yellow amorphous solid by preparative TLC using petroleum ether/EtOAc (15:1) as an eluent; $R_f$= 0.6 (petroleum ether/EtOAc = 10:1); 55.0 mg, 69% yield. **$^1$H NMR** (400 MHz, $CDCl_3$) $\delta$ 7.49 – 7.47 (m, 2H), 7.28 – 7.24 (m, 2H), 6.90 – 6.86 (m, 2H), 6.64 – 6.60 (m, 2H), 5.02 (s, 2H), 3.65 (brs, 1H), 3.35 – 3.20 (m, 2H), 2.52 – 2.41 (m, 1H), 2.02 – 1.96 (m, 3H), 1.76 – 1.72 (m, 3H), 1.66 – 1.61 (m, 3H), 1.56 – 1.47 (m, 7H), 1.22 – 1.17 (m, 1H). **$^{13}$C NMR** (101 MHz, $CDCl_3$) $\delta$ 151.5, 148.9, 142.3, 136.4, 129.0, 128.7 (q, $J$ = 280.8 Hz), 121.2, 120.6 (q, $J$ = 258.6 Hz), 116.4, 114.5, 70.1, 45.9, 42.3, 40.7, 37.2 (q, $J$ = 24.2 Hz), 37.0, 32.5, 28.6. **$^{19}$F NMR** (376 MHz, $CDCl_3$) $\delta$ -57.85 (s, 3F), -69.23 (s, 3F). **HRMS** (ESI) m/z: $[M+H]^+$ Calcd for $C_{28}H_{32}F_6NO_2^+$ 528.2337; Found 528.2343.

***N*-(2-((adamantan-1-yl)methyl)-3,3,3-trifluoropropyl)-4-((4-(trifluoromethyl)-benzyl)oxy)aniline (20)**

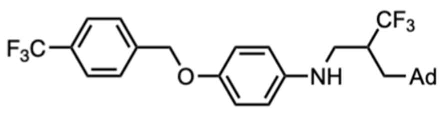

Using the General Procedure C, the title compound was obtained as a pale yellow amorphous solid by preparative TLC using petroleum ether/EtOAc (15:1) as an eluent; $R_f$ = 0.6 (petroleum ether/EtOAc = 10:1); 55.9 mg, 73% yield. **$^1$H NMR** (400 MHz, $CDCl_3$) $\delta$ 7.64 – 7.52 (m, 4H), 6.87 – 6.84 (m, 2H), 6.60 – 6.57 (m, 2H), 5.06 (s, 2H), 3.32 – 3.16 (m, 2H), 2.49 – 2.39 (m, 1H), 1.98 – 1.96 (m, 3H), 1.72 – 1.69 (m, 3H), 1.62 – 1.59 (m, 3H), 1.52 – 1.44 (m, 7H), 1.19 – 1.13 (m, 1H). (Due to the broadening effect and intermolecular hydrogen bonding, one of the proton signals of NH could not be observed.). **$^{13}$C NMR** (151 MHz, $CDCl_3$) $\delta$ 151.4, 142.4, 141.8, 130.1 (q, $J$ = 33.2 Hz), 128.7 (q, $^1J_{CF}$ = 280.9 Hz), 127.5, 125.6, 124.3 (q, $^1J_{CF}$ = 273.3 Hz), 116.4,

114.5, 70.1, 45.9, 42.3, 40.7, 37.2 (q, ${}^{2}J_{CF}$ = 24.2 Hz), 37.0, 32.5, 28.6. **$^{19}$F NMR** (376 MHz, $CDCl_3$) $\delta$ -62.49 (s, 3F), -69.22z (s, 3F). **HRMS** (ESI) m/z: $[M+H]^+$ Calcd for $C_{28}H_{32}F_6NO^+$ 512.2388; Found 512.2396.

**Methyl 4-((4-((2-((-adamantan-1-yl)methyl)-3,3,3-trifluoropropyl)amino)-phenoxy)methyl)benzoate (21)**

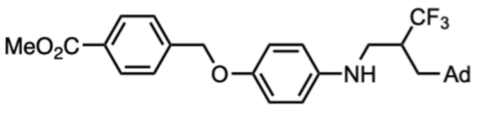


Using the General Procedure C (Variation 5), the title compound was obtained as a brown amorphous solid by preparative TLC using petroleum ether/EtOAc (10:1) as an eluent; $R_f$= 0.4 (petroleum ether/EtOAc = 10:1); 28.0 mg, 37% yield. **$^1$H NMR** (400 MHz, $CDCl_3$) $\delta$ 8.06 – 8.03 (m, 2H), 7.51 – 7.48 (m, 2H), 6.87 – 6.84 (m, 2H), 6.60 – 6.57 (m, 2H), 5.06 (s, 2H), 3.92 (s, 3H), 3.32 – 3.16 (m, 2H), 2.44 – 2.21 (m, 1H), 1.98 – 1.95 (m, 3H), 1.72 – 1.69 (m, 3H), 1.62 – 1.58 (m, 3H), 1.52 – 1.43 (m, 7H), 1.19 – 1.13 (m, 1H). (Due to the broadening effect and intermolecular hydrogen bonding, one of the proton signals of NH could not be observed.). **$^{13}$C NMR** (151 MHz, $CDCl_3$) $\delta$ 167.1, 151.4, 143.0, 142.3, 130.0, 129.7, 128.7 (q, ${}^{1}J_{CF}$ = 280.9 Hz), 127.1, 116.4, 114.5, 70.4, 52.2, 46.0, 42.3, 40.7, 37.2 (q, ${}^{2}J_{CF}$ = 24.2 Hz), 37.0, 32.5, 28.6. **$^{19}$F NMR** (376 MHz, $CDCl_3$) $\delta$ -69.23 (s, 3F). **HRMS** (ESI) m/z: $[M+H]^+$ Calcd for $C_{29}H_{35}F_3NO_3^+$ 502.2569; Found 502.2577.

**4-((4-((2-((adamantan-1-yl)methyl)-3,3,3-trifluoropropyl)amino)phenoxy)methyl) benzonitrile (22)**

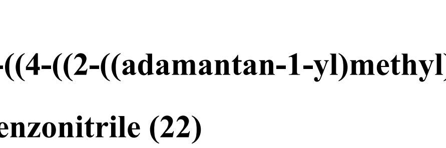


Using the General Procedure C (Variation 5), the title compound was obtained as a brown amorphous solid by preparative TLC using petroleum ether/EtOAc (10:1) as an eluent; $R_f$= 0.4 (petroleum ether/EtOAc = 10:1); 49.2 mg, 70% yield. **$^1$H NMR** (400 MHz, $CDCl_3$) $\delta$ 7.66 (d, $J$ = 8.0 Hz, 2H), 7.53 (d, $J$ = 8.0 Hz, 2H), 6.83 (d, $J$ = 8.0 Hz, 2H), 6.58 (d, $J$ = 8.0 Hz, 2H), 5.06 (s, 2H), 3.31 – 3.16 (m, 2H), 2.47– 2.39 (m, 1H), 1.97 – 1.88 (m, 3H), 1.72 – 1.69 (m, 3H), 1.62 –

1.58 (m, 3H), 1.52 – 1.42 (m, 7H), 1.18 – 1.13 (m, 1H). (Due to the broadening effect and intermolecular hydrogen bonding, one of the proton signals of NH could not be observed.). **$^{13}$C NMR** (151 MHz, $CDCl_3$) $\delta$ 151.1, 143.2, 142.5, 132.4, 128.8 (q, $^1J_{CF}$ = 279.4 Hz), 127.7, 118.9, 116.3, 114.5, 111.6, 69.9, 45.8, 42.3, 40.6, 37.1 (q, $^2J_{CF}$ = 24.2 Hz), 36.9, 32.5, 28.5. **$^{19}$F NMR** (376 MHz, $CDCl_3$) $\delta$ -69.21 (s, 3F). **HRMS** (ESI) m/z: $[M+H]^+$ Calcd for $C_{28}H_{32}F_3N_2O^+$ 469.2467; Found 469.2474.

***N*-(2-((adamantan-1-yl)methyl)-3,3,3-trifluoropropyl)-4-(4-methoxyphen-oxy)-aniline (23)**

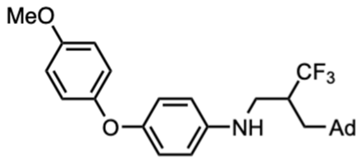


Using the General Procedure C, the title compound was obtained as a yellow viscous oil by preparative TLC using petroleum ether/EtOAc (15:1) as an eluent; $R_f$ = 0.6 (petroleum ether/EtOAc = 10:1); 44.1 mg, 64% yield. **$^1$H NMR** (400 MHz, $CDCl_3$) $\delta$ 6.93 – 6.83 (m, 6H), 6.61 – 6.58 (m, 2H), 3.79 – 3.74 (m, 4H), 3.35 – 3.19 (m, 2H), 2.50 – 2.45 (m, 1H), 1.98 – 1.97 (m, 3H), 1.73 – 1.70 (m, 3H), 1.63 – 1.60 (m, 3H), 1.53 – 1.41 (m, 7H), 1.20 – 1.15 (m, 1H). **$^{13}$C NMR** (101 MHz, $CDCl_3$) $\delta$ 155.1, 152.3, 149.7, 143.6, 128.6 (q, $^1J_{CF}$ = 280.8 Hz), 120.3, 119.1, 114.8, 114.2, 55.8, 45.6, 42.3, 40.6, 37.1 (q, $^2J_{CF}$ = 23.2 Hz), 36.9, 32.5, 28.5. **$^{19}$F NMR** (376 MHz, $CDCl_3$) $\delta$ -69.21 (s, 3F). **HRMS** (ESI) m/z: $[M+H]^+$ Calcd for $C_{27}H_{33}F_3NO_2^+$ 460.2463; Found 460.2469.

**4-([1,1'-biphenyl]-4-yloxy)-*N*-(2-((adamantan-1-yl)methyl)-3,3,3-trifluoropropyl) aniline (24)**

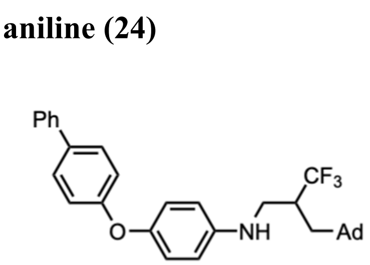


Using the General Procedure C, the title compound was obtained as a yellow viscous oil by preparative TLC using petroleum ether/EtOAc (15:1) as an eluent; $R_f$= 0.6 (petroleum ether/EtOAc = 10:1); 33.2 mg, 44% yield. **$^1$H NMR** (400 MHz, $CDCl_3$) $\delta$ 7.58 – 7.30 (m, 7H), 7.01 – 6.96 (m, 4H), 6.66 – 6.62 (m, 2H), 3.85 (brs, 1H), 3.37 – 3.21 (m, 2H), 2.51 – 2.44 (m, 1H), 1.99 – 1.97 (m,

3H), 1.73 – 1.70 (m, 3H), 1.64 – 1.60 (m, 3H), 1.54 – 1.45 (m, 7H), 1.21 – 1.16 (m, 1H). **$^{13}$C NMR** (101 MHz, $CDCl_3$) $\delta$ 158.8, 148.1, 144.3, 140.8, 135.2, 128.9, 128.6 (q, $^1J_{CF}$ = 280.8 Hz), 128.4, 127.0, 123.4, 121.6, 117.4, 114.3, 45.5, 42.3, 40.6, 37.1 (q, $^2J_{CF}$ = 24.2 Hz), 36.9, 32.5, 28.5. **$^{19}$F NMR** (376 MHz, $CDCl_3$) $\delta$ -69.19 (s, 3F). **HRMS** (ESI) m/z: $[M+H]^+$ Calcd for $C_{32}H_{35}F_3NO^+$ 506.2671; Found 506.2579.

***N*-(2-((adamantan-1-yl)methyl)-3,3,3-trifluoropropyl)-4-phenoxyaniline (25)**

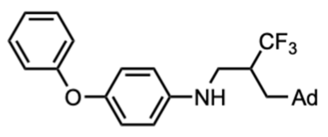


Using the General Procedure C, the title compound was obtained as a yellow viscous oil by preparative TLC using petroleum ether/EtOAc (15:1) as an eluent; $R_f$ = 0.6 (petroleum ether/EtOAc = 10:1); 38.5 mg, 60% yield. **$^1$H NMR** (400 MHz, $CDCl_3$) $\delta$ 7.29 – 7.24 (m, 2H), 7.02 – 7.00 (m, 1H), 6.99 – 6.89 (m, 4H), 6.63 – 6.59 (m, 2H), 3.77 (brs, 1H), 3.35 – 3.19 (m, 2H), 2.52 – 2.40 (m, 1H), 2.01 – 1.95 (m, 3H), 1.72 – 1.69 (m, 3H), 1.63 – 1.59 (m, 3H), 1.53 – 1.43 (m, 7H), 1.20 – 1.15 (m, 1H). **$^{13}$C NMR** (151 MHz, $CDCl_3$) $\delta$ 159.1, 148.3, 144.2, 129.6, 128.7 (q, $^1J_{CF}$ = 279.4 Hz), 122.2, 121.4, 117.3, 114.3, 45.6, 42.3, 40.6, 37.1 (q, $^2J_{CF}$ = 24.2 Hz), 36.9, 32.5, 28.6. **$^{19}$F NMR** (376 MHz, $CDCl_3$) $\delta$ -69.19 (s, 3F). **HRMS** (ESI) m/z: $[M+H]^+$ Calcd for $C_{26}H_{31}F_3NO^+$ 430.2358; Found 430.2366.

***N*-(2-((adamantan-1-yl)methyl)-3,3,3-trifluoropropyl)-4-(3-chlorophenoxy)-aniline (26)**

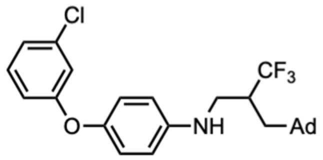


Using the General Procedure C, the title compound was obtained as a yellow viscous oil by preparative TLC using petroleum ether/EtOAc (15:1) as an eluent; $R_f$ = 0.6 (petroleum ether/EtOAc = 10:1); 39.6 mg, 57% yield. **$^1$H NMR** (400 MHz, $CDCl_3$) $\delta$ 7.20 – 7.17 (m, 1H), 6.99 – 6.80 (m, 5H), 6.65 – 6.61 (m, 2H), 3.83 (brs, 1H), 3.36 – 3.21 (m, 2H), 2.51 – 2.44 (m, 1H), 2.02 – 1.97 (m, 3H), 1.77 – 1.70 (m, 3H), 1.63 – 1.60 (m, 3H), 1.54 – 1.45 (m, 7H), 1.21 – 1.17 (m, 1H). **$^{13}$C NMR** (151 MHz, $CDCl_3$) $\delta$ 160.1, 147.3, 144.7, 135.0, 130.4, 128.6 (q, $^1J_{CF}$ =

280.9 Hz), 122.2, 121.8, 117.3, 115.4, 114.2, 45.5, 42.4, 40.6, 37.1 (q, $^2J_{CF}$ = 24.2 Hz), 37.0, 32.5, 28.6. **$^{19}$F NMR** (376 MHz, $CDCl_3$) $\delta$ -69.22 (s, 3F). **HRMS** (ESI) m/z: $[M+H]^+$ Calcd for $C_{26}H_{30}ClF_3NO^+$ 464.1968; Found 464.1975.

**$N$-(2-((adamantan-1-yl)methyl)-3,3,3-trifluoropropyl)-4-(4-(trifluoromethyl)-phenoxy)aniline (27)**

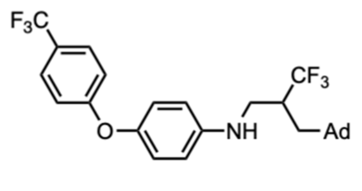


Using the General Procedure C, the title compound was obtained as a brown amorphous solid by preparative TLC using petroleum ether/EtOAc (15:1) as an eluent; $R_f$ = 0.6 (petroleum ether/EtOAc = 10:1); 31.3 mg, 42% yield. **$^1$H NMR** (400 MHz, $CDCl_3$) $\delta$ 7.53 – 7.51 (m, 2H), 6.98 – 6.88 (m, 4H), 6.67 – 6.62 (m, 2H), 3.86 (brs, 1H), 3.37 – 3.22 (m, 2H), 2.53 – 2.42 (m, 1H), 2.00 – 1.97 (m, 3H), 1.74 – 1.71 (m, 3H), 1.64 – 1.60 (m, 3H), 1.54 – 1.46 (m, 7H), 1.21 – 1.16 (m, 1H). **$^{13}$C NMR** (151 MHz, $CDCl_3$) $\delta$ 162.1, 146.8, 145.0, 128.6 (q, $^1J_{CF}$ = 279.4 Hz), 127.1, 124.5 (q, $^1J_{CF}$ = 271.8 Hz), 124.0 (q, $^2J_{CF}$ = 33.2 Hz), 122.0, 116.7, 114.3, 45.4, 42.4, 40.6, 37.1 (q, $^2J_{CF}$ = 24.2 Hz), 36.9, 32.5, 28.6. **$^{19}$F NMR** (376 MHz, $CDCl_3$) $\delta$ -61.59 (s, 3F), -69.23 (s, 3F). **HRMS** (ESI) m/z: $[M+H]^+$ Calcd for $C_{27}H_{30}F_6NO^+$ 498.2232; Found 498.2237.

**4-(4-((2-((adamantan-1-yl)methyl)-3,3,3-trifluoropropyl)amino)phenoxy)benz-aldehyde (28)**

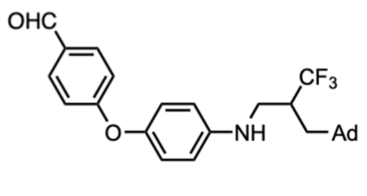


Using the General Procedure C (Variation 5), the title compound was obtained as a yellow viscous oil by preparative TLC using petroleum ether/EtOAc (12:1) as an eluent; $R_f$= 0.5 (petroleum ether/EtOAc = 10:1); 15.8 mg, 23% yield. **$^1$H NMR** (400 MHz, $CDCl_3$) $\delta$ 9.89 (s, 1H), 7.82 – 7.79 (m, 2H), 7.01 – 6.93 (m, 4H), 6.66 – 6.64 (m, 2H), 3.91 (brs, 1H), 3.38 – 3.22 (m, 2H), 2.52 – 2.44 (m, 1H), 2.00 – 1.96 (m, 3H), 1.73 – 1.70 (m, 3H), 1.64 – 1.60 (m, 3H), 1.54 – 1.45 (m, 7H), 1.24 – 1.16 (m, 1H). **$^{13}$C NMR** (101 MHz, $CDCl_3$) $\delta$ 191.0, 164.7,

146.3, 145.2, 132.1, 128.6 (q, $^{1}J_{CF}$ = 281.8 Hz), 122.2, 116.7, 114.2, 45.3, 42.3, 40.6, 37.1 (q, $^{2}J_{CF}$ = 24.2 Hz), 36.9, 32.5, 28.6. **$^{19}$F NMR** (376 MHz, $CDCl_3$) $\delta$ -69.22 (s, 3F). **HRMS** (ESI) m/z: $[M+H]^+$ Calcd for $C_{27}H_{31}F_3NO_2^+$ 458.2307; Found 458.2312.

***N*-(2-((adamantan-1-yl)methyl)-3,3,3-trifluoropropyl)-4-(3-chloropropoxy)-aniline (29)**

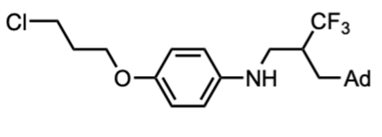

Using the General Procedure C, the title compound was obtained as a yellow viscous oil by preparative TLC using petroleum ether/EtOAc (15:1) as an eluent; $R_f$= 0.6 (petroleum ether/EtOAc = 10:1); 31.0 mg, 48% yield. **$^{1}$H NMR** (400 MHz, $CDCl_3$) $\delta$ 6.80 (d, $J$ = 8.0 Hz, 2H), 6.59 (d, $J$ = 8.0 Hz, 2H), 4.05 (t, $J$ = 5.9 Hz, 2H), 3.74 (t, $J$ = 6.4 Hz, 2H), 3.63 (brs, 1H), 3.31 – 3.17 (m, 2H), 2.48 – 2.41 (m, 1H), 2.23 – 2.17 (m, 2H), 1.98 – 1.96 (m, 3H), 1.76 – 1.72 (m, 3H), 1.69 – 1.59 (m, 3H), 1.53 – 1.42 (m, 7H), 1.19 – 1.14 (m, 1H). **$^{13}$C NMR** (101 MHz, $CDCl_3$) $\delta$ 151.6, 142.0, 128.7 (q, $^{1}J_{CF}$ = 279.8 Hz), 116.0, 114.6, 65.2, 46.0, 42.3, 41.8, 40.7, 37.1 (q, $^{2}J_{CF}$ = 24.2 Hz), 36.9, 32.6, 32.5, 28.5. **$^{19}$F NMR** (376 MHz, $CDCl_3$) $\delta$ -69.22 (s, 3F). **HRMS** (ESI) m/z: $[M+H]^+$ Calcd for $C_{23}H_{32}ClF_3NO^+$ 430.2125; Found 430.2129.

***N*-(2-((adamantan-1-yl)methyl)-3,3,3-trifluoropropyl)-4-(allyloxy)aniline (30)**

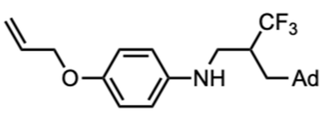

Using the General Procedure C, the title compound was obtained as a yellow viscous oil by preparative TLC using petroleum ether/EtOAc (15:1) as an eluent; $R_f$= 0.6 (petroleum ether/EtOAc = 10:1); 25.6 mg, 43% yield. **$^{1}$H NMR** (400 MHz, $CDCl_3$) $\delta$ 6.84 – 6.78 (m, 2H), 6.60 – 6.56 (m, 2H), 6.10 – 6.00 (m, 1H), 5.39 (dd, $J$ = 17.2, 1.7 Hz, 1H), 5.26 (dd, $J$ = 10.5, 1.5 Hz, 1H), 4.47 (d, $J$ = 5.4 Hz, 2H), 3.31 – 3.16 (m, 2H), 2.49 – 2.39 (m, 1H), 1.98 – 1.95 (m, 3H), 1.72 – 1.69 (m, 3H), 1.63 – 1.58 (m, 3H), 1.53 – 1.43 (m, 7H), 1.19 – 1.14 (m, 1H). (Due to the broadening effect and intermolecular hydrogen bonding, one of the proton signals of NH could not be observed.). **$^{13}$C NMR** (151 MHz, $CDCl_3$) $\delta$ 151.6, 142.0, 134.0, 128.7 (q, $^{1}J_{CF}$ = 279.7

Hz), 117.5, 116.3, 114.5, 69.8, 46.0, 42.3, 40.7, 37.2 (q, $^2J_{CF}$ = 24.6 Hz), 36.9, 32.5, 28.6. **$^{19}$F NMR** (376 MHz, $CDCl_3$) $\delta$ -69.23 (s, 3F). **HRMS** (ESI) m/z: $[M+H]^+$ Calcd for $C_{23}H_{31}F_3NO^+$ 394.2358; Found 394.2365.

**7-(4-((2-((adamantan-1-yl)methyl)-3,3,3-trifluoropropyl)amino)phenoxy)-3,4-dihydronaphthalen-1(2*H*)-one (31)**

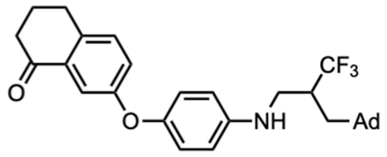


Using the General Procedure C (Variation 5), the title compound was obtained as a brown amorphous solid by preparative TLC using petroleum ether/EtOAc (10:1) as an eluent; $R_f$ = 0.5 (petroleum ether/EtOAc = 10:1); 28.3 mg, 38% yield. **$^1$H NMR** (400 MHz, $CDCl_3$) $\delta$ 7.77 – 7.75 (m, 1H), 7.21 – 7.17 (m, 1H), 6.96 – 6.94 (m, 1H), 6.88 – 6.84 (m, 2H), 6.64 – 6.59 (m, 2H), 3.35 – 3.20 (m, 2H), 2.99 (t, $J$ = 6.1 Hz, 2H), 2.66 (t, $J$ = 5.8 Hz, 2H), 2.50 – 2.43 (m, 1H), 2.17 – 2.11 (m, 2H), 1.99 – 1.96 (m, 3H), 1.73 – 1.70 (m, 3H), 1.63 – 1.59 (m, 3H), 1.54 – 1.44 (m, 7H), 1.20 – 1.15 (m, 1H). (Due to the broadening effect and intermolecular hydrogen bonding, one of the proton signals of NH could not be observed.). **$^{13}$C NMR** (101 MHz, $CDCl_3$) $\delta$ 198.5, 156.0, 148.5, 144.2, 135.1, 134.3, 128.6 (q, $^1J_{CF}$ = 281.4 Hz), 126.9, 121.4, 121.2, 120.5, 114.3, 45.6, 42.3, 40.6, 39.0, 37.1 (q, $^2J_{CF}$ = 24.2 Hz), 36.9, 32.5, 28.5, 23.3, 22.7. **$^{19}$F NMR** (376 MHz, $CDCl_3$) $\delta$ -69.22 (s, 3F). **HRMS** (ESI) m/z: $[M+H]^+$ Calcd for $C_{30}H_{35}F_3NO_2^+$ 498.2620; Found 498.2629.

***N*-(2-((adamantan-1-yl)methyl)-3,3,3-trifluoropropyl)-4-(naphthalen-2-yl-oxy)-aniline (32)**

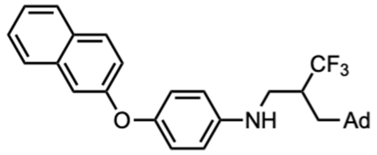


Using the General Procedure C, the title compound was obtained as a yellow viscous oil by preparative TLC using petroleum ether/EtOAc (15:1) as an eluent; $R_f$= 0.6 (petroleum ether/EtOAc = 10:1); 52.2 mg, 73% yield. **$^1$H NMR** (400 MHz, $CDCl_3$) $\delta$ 7.72 (d, $J$ = 8.6 Hz, 2H), 7.57 (d, $J$ =

8.1 Hz, 1H), 7.36 – 7.27 (m, 2H), 7.19 – 7.16 (m, 1H), 7.08 – 7.07 (m, 1H), 6.92 (d, *J* = 8.6 Hz, 2H), 6.58 (d, *J* = 8.8 Hz, 2H), 3.76 (brs, 1H), 3.32 – 3.15 (m, 2H), 2.45 – 2.38 (m, 1H), 1.96 – 1.90 (m, 3H), 1.66 – 1.63 (m, 3H), 1.56 – 1.53 (m, 3H), 1.48 – 1.39 (m, 7H), 1.15 – 1.10 (m, 1H). **$^{13}$C NMR** (101 MHz, $CDCl_3$) $\delta$ 157.1, 148.2, 144.4, 134.5, 129.8, 129.8, 128.7 (q, $^1J_{CF}$ = 280.0 Hz), 127.8, 127.1, 126.5, 124.3, 121.6, 119.3, 114.3, 111.7, 45.6, 42.4, 40.7, 37.2 (q, $^2J_{CF}$ = 24.4 Hz), 37.0, 32.5, 28.6. **$^{19}$F NMR** (376 MHz, $CDCl_3$) $\delta$ -69.19 (s, 3F). **HRMS** (ESI) m/z: $[M+H]^+$ Calcd for $C_{30}H_{33}F_3NO^+$ 480.2514; Found 480.2521.

**$N$-(2-((adamantan-1-yl)methyl)-3,3,3-trifluoropropyl)-4-(pyridin-2-yloxy)-aniline (33)**

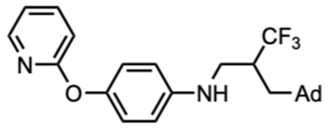


Using the General Procedure C (Variation 5), the title compound was obtained as a green amorphous solid by preparative TLC using petroleum ether/EtOAc (10:1) as an eluent; $R_f$ = 0.5 (petroleum ether/EtOAc = 10:1); 33.3 mg, 52% yield. **$^1$H NMR** (400 MHz, $CDCl_3$) $\delta$ 8.19 – 8.18 (m, 1H), 7.65 – 7.61 (m, 1H), 7.01 – 6.92 (m, 3H), 6.84 – 6.83 (m, 1H), 6.66 – 6.62 (m, 2H), 3.84 (brs, 1H), 3.34 – 3.21 (m, 2H), 2.50 – 2.43 (m, 1H), 2.02 – 1.97 (m, 3H), 1.73 – 1.70 (m, 3H), 1.66 – 1.59 (m, 3H), 1.54 – 1.45 (m, 7H), 1.21 – 1.17 (m, 1H). **$^{13}$C NMR** (101 MHz, $CDCl_3$) $\delta$ 164.7, 147.9, 145.7, 144.9, 139.3, 128.7 (q, $^1J_{CF}$ = 279.4 Hz), 122.6, 118.0, 114.0, 110.9, 45.4, 42.3, 40.6, 37.3 (q, $^2J_{CF}$ = 24.4 Hz), 36.9, 32.5, 28.6. **$^{19}$F NMR** (376 MHz, $CDCl_3$) $\delta$ -69.23 (s, 3F). **HRMS** (ESI) m/z: $[M+H]^+$ Calcd for $C_{25}H_{30}F_3N_2O^+$ 431.2310; Found 431.2318.

**$N$-(2-((adamantan-1-yl)methyl)-3,3,3-trifluoropropyl)-4-(thiophen-2-yl-methoxy) aniline (34)**

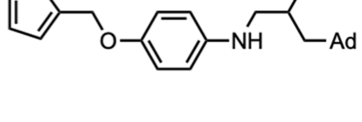


Using the General Procedure C, the title compound was obtained as a brown amorphous solid by preparative TLC using petroleum ether/EtOAc (15:1)

as an eluent; $R_f$= 0.6 (petroleum ether/EtOAc = 10:1); 45.7 mg, 68% yield. **$^1$H NMR** (400 MHz, $CDCl_3$) $\delta$ 7.32 – 7.30 (m, 1H), 7.08 – 7.07 (m, 1H), 7.01 – 6.99 (m, 1H), 6.90 – 6.86 (m, 2H), 6.60 – 6.57 (m, 2H), 5.16 (s, 2H), 3.63 (brs, 1H), 3.32 – 3.18 (m, 2H), 2.50 – 2.40 (m, 1H), 2.01 – 1.97 (m, 3H), 1.73 – 1.70 (m, 3H), 1.65 – 1.59 (m, 3H), 1.53 – 1.45 (m, 7H), 1.19 – 1.15 (m, 1H). **$^{13}$C NMR** (151 MHz, $CDCl_3$) $\delta$ 151.2, 142.4, 140.0, 128.7 (q, $^1J_{CF}$ = 280.2 Hz), 126.8, 126.7, 126.1, 116.8, 114.4, 66.2, 45.9, 42.3, 40.7, 37.1 (q, $^2J_{CF}$ = 24.4 Hz), 36.9, 32.5, 28.6. **$^{19}$F NMR** (376 MHz, $CDCl_3$) $\delta$ -69.20 (s, 3F). **HRMS** (ESI) m/z: $[M+H]^+$ Calcd for $C_{25}H_{31}F_3NOS^+$ 450.2078; Found 450.2086.

***N*-(2-((adamantan-1-yl)methyl)-3,3,3-trifluoropropyl)-4-((tetrahydro-2*H*-pyran-4-yl)methoxy)aniline (35)**

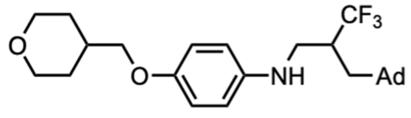


Using the General Procedure C (Variation 5), the title compound was obtained as a green amorphous solid by preparative TLC using petroleum ether/EtOAc (10:1) as an eluent; $R_f$= 0.4 (petroleum ether/EtOAc = 10:1); 43.5 mg, 64% yield. **$^1$H NMR** (400 MHz, $CDCl_3$) $\delta$ 6.79 (d, $J$ = 8.9 Hz, 2H), 6.58 (d, $J$ = 8.9 Hz, 2H), 4.03 – 3.99 (m, 2H), 3.74 (d, $J$ = 6.4 Hz, 2H), 3.46 – 3.40 (m, 2H), 3.31 – 3.16 (m, 2H), 2.49 – 2.38 (m, 1H), 2.07 – 1.92 (m, 4H), 1.77 – 1.69 (m, 5H), 1.63 – 1.58 (m, 3H), 1.52 – 1.39 (m, 9H), 1.19 – 1.14 (m, 1H). (Due to the broadening effect and intermolecular hydrogen bonding, one of the proton signals of NH could not be observed.). **$^{13}$C NMR** (151 MHz, $CDCl_3$) $\delta$ 152.0, 141.9, 128.7 (q, $^1J_{CF}$ = 279.6 Hz), 115.9, 114.6, 73.6, 67.8, 46.1, 42.3, 40.7, 37.2 (q, $^2J_{CF}$ = 24.5 Hz), 37.0, 35.4, 32.5, 30.0, 28.6. **$^{19}$F NMR** (376 MHz, $CDCl_3$) $\delta$ -69.21 (s, 3F). **HRMS** (ESI) m/z: $[M+H]^+$ Calcd for $C_{26}H_{37}F_3NO_2^+$ 452.2776; Found 452.2781.

***N*-(2-((adamantan-1-yl)methyl)-3,3,3-trifluoropropyl)-4-(1,3-dioxolan-2-yl)-aniline (36)**

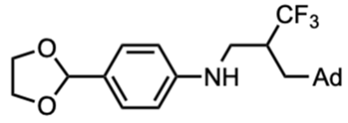


Using the General Procedure C, the title compound was obtained as a pale yellow amorphous solid by preparative TLC using petroleum ether/EtOAc (15:1) as an eluent; $R_f$= 0.6 (petroleum ether/EtOAc = 10:1); 25.2 mg, 41% yield. **$^1$H NMR** (400 MHz, $CDCl_3$) $\delta$ 7.30 (d, $J$ = 8.6 Hz, 2H), 6.60 (d, $J$ = 8.5 Hz, 2H), 5.71 (s, 1H), 4.15 – 4.09 (m, 2H), 4.05 – 3.99 (m, 2H), 3.97 (brs, 1H), 3.36 – 3.23 (m, 2H), 2.50 – 2.39 (m, 1H), 1.99 – 1.92 (m, 3H), 1.71 – 1.68 (m, 3H), 1.62 – 1.58 (m, 3H), 1.48 – 1.44 (m, 7H), 1.17 – 1.12 (m, 1H). **$^{13}$C NMR** (201 MHz, $CDCl_3$) $\delta$ 148.5, 128.6 (q, $^1J_{CF}$ = 280.1 Hz), 128.0, 127.0, 112.7, 104.3, 65.3, 44.8, 42.3, 40.6, 37.1 (q, $^2J_{CF}$ = 24.2 Hz), 36.9, 32.5, 28.54. **$^{19}$F NMR** (376 MHz, $CDCl_3$) $\delta$ -69.23 (s, 3F). **HRMS** (ESI) m/z: $[M+Na]^+$ Calcd for $C_{23}H_{31}F_3NNaO_2^+$ 432.2126; Found 432.2128.

***N*-(2-((adamantan-1-yl)methyl)-3,3,3-trifluoropropyl)-4-methoxy-3-methylaniline (37)**

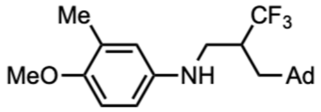


Using the General Procedure C, the title compound was obtained as a yellow viscous oil by preparative TLC using petroleum ether/EtOAc (15:1) as an eluent; $R_f$= 0.6 (petroleum ether/EtOAc = 10:1); 25.0 mg, 44% yield. **$^1$H NMR** (400 MHz, $CDCl_3$) $\delta$ 6.73 – 6.70 (m, 1H), 6.50 – 6.41 (m, 2H), 3.77 – 3.76 (m, 3H), 3.30 – 3.16 (m, 2H), 2.48 – 2.42 (m, 1H), 2.19 – 2.18 (m, 3H), 1.98 – 1.96 (m, 3H), 1.72 – 1.69 (m, 3H), 1.63 – 1.59 (m, 3H), 1.53 – 1.42 (m, 7H), 1.19 – 1.12 (m, 1H). (Due to the broadening effect and intermolecular hydrogen bonding, one of the proton signals of NH could not be observed.). **$^{13}$C NMR** (151 MHz, $CDCl_3$) $\delta$ 151.0, 141.5, 128.8 (q, $^1J_{CF}$ = 280.0 Hz), 127.9, 117.q, 111.9, 111.1, 56.2, 46.1, 42.3, 40.7, 37.2 (q, $^2J_{CF}$ = 24.3 Hz), 37.0, 32.5, 28.6, 16.5. **$^{19}$F NMR** (376 MHz, $CDCl_3$) $\delta$ -69.20 (s, 3F). **HRMS** (ESI) m/z: $[M+H]^+$ Calcd for $C_{22}H_{31}F_3NO^+$ 382.2358; Found 382.2365.

***N*-(2-((adamantan-1-yl)methyl)-3,3,3-trifluoropropyl)-4-methoxy-3-(tri-fluoro-methyl)aniline (38)**

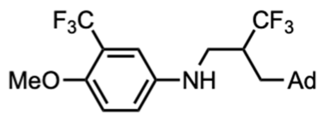

Using the General Procedure C, the title compound was obtained as a yellow viscous oil by preparative TLC using petroleum ether/EtOAc (15:1) as an eluent; $R_f$= 0.6 (petroleum ether/EtOAc = 10:1); 33.5 mg, 51% yield. **$^1$H NMR** (400 MHz, $CDCl_3$) $\delta$ 6.91 – 6.85 (m, 2H), 6.75 – 6.73 (m, 1H), 3.83 (s, 3H), 3.74 (brs, 1H), 3.32 – 3.19 (m, 2H), 2.47 – 2.40 (m, 1H), 2.00 – 1.96 (m, 3H), 1.73 – 1.70 (m, 3H), 1.63 – 1.57 (m, 3H), 1.53 – 1.44 (m, 7H), 1.16 – 1.11 (m, 1H). **$^{13}$C NMR** (101 MHz, $CDCl_3$) $\delta$ 150.1, 141.2, 128.6 (q, $^1J_{CF}$ = 279.9 Hz), 123.8 (q, $^1J_{CF}$ = 272.5 Hz), 119.7 (q, $^2J_{CF}$ = 30.5 Hz), 117.6, 114.4, 111.9 (q, $^3J_{CF}$ = 5.4 Hz), 56.9, 45.8, 42.3, 40.5, 36.9 (q, $^2J_{CF}$ = 24.5 Hz), 36.8, 32.5, 28.5. **$^{19}$F NMR** (376 MHz, $CDCl_3$) $\delta$ -62.27 (s, 3F), -69.19 (s, 3F). **HRMS** (ESI) m/z: $[M+H]^+$ Calcd for $C_{22}H_{28}F_6NO^+$ 436.2075; Found 436.2083.

***N*-(2-((adamantan-1-yl)methyl)-3,3,3-trifluoropropyl)-3-bromo-4-methoxyaniline (39)**

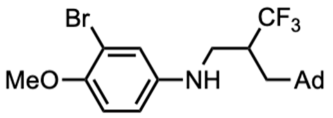

Using the General Procedure C, the title compound was obtained as a yellow viscous oil by preparative TLC using petroleum ether/EtOAc (15:1) as an eluent; $R_f$= 0.6 (petroleum ether/EtOAc = 10:1); 24.5 mg, 37% yield. **$^1$H NMR** (400 MHz, $CDCl_3$) $\delta$ 6.87 – 6.78 (m, 2H), 6.62 – 6.53 (m, 1H), 3.82 – 3.76 (m, 3H), 3.62 (brs, 1H), 3.31 – 3.15 (m, 2H), 2.48 – 2.39 (m, 1H), 2.00 – 1.95 (m, 3H), 1.73 – 1.69 (m, 3H), 1.64 – 1.59 (m, 3H), 1.53 – 1.43 (m, 7H), 1.19 – 1.11 (m, 1H). **$^{13}$C NMR** (101 MHz, $CDCl_3$) $\delta$ 148.8, 142.7, 128.6 (q, $^1J_{CF}$ = 280.3 Hz), 118.3, 114.0, 113.3, 112.8, 57.2, 45.7, 42.3, 40.6, 37.0 (q, $^2J_{CF}$ = 24.4 Hz), 36.9, 32.5, 28.5. **$^{19}$F NMR** (376 MHz, $CDCl_3$) $\delta$ -69.21 (s, 3F). **HRMS** (ESI) m/z: $[M+H]^+$ Calcd for $C_{21}H_{28}BrF_3NO^+$ 446.1306; Found 446.1316.

***N*-(2-((adamantan-1-yl)methyl)-3,3,3-trifluoropropyl)-4-bromo-3-methoxyaniline (40)**

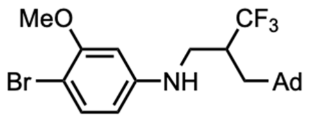


Using the General Procedure C, the title compound was obtained as a yellow viscous oil by preparative TLC using petroleum ether/EtOAc (15:1) as an eluent; R*f* = 0.6 (petroleum ether/EtOAc = 10:1); 23.2 mg, 35% yield. **$^{1}$H NMR** (400 MHz, $CDCl_3$) $\delta$ 7.30 – 7.28 (m, 1H), 6.18 – 6.17 (m, 1H), 6.12 – 6.10 (m, 1H), 3.92 (brs, 1H), 3.85 (s, 3H), 3.34 – 3.21 (m, 2H), 2.47 – 2.41 (m, 1H), 1.98 – 1.97 (m, 3H), 1.73 – 1.70 (m, 3H), 1.62 – 1.60 (m, 3H), 1.52 – 1.46 (m, 7H), 1.16 – 1.12 (m, 1H). **$^{13}$C NMR** (151 MHz, $CDCl_3$) $\delta$ 156.7, 148.3, 133.6, 128.5 (q, $^{1}J_{CF}$ = 280.1 Hz), 106.3, 99.1, 97.8, 56.1, 45.0, 42.3, 40.6, 37.1 (q, $^{2}J_{CF}$ = 24.5 Hz), 36.9, 32.5, 28.5. **$^{19}$F NMR** (376 MHz, $CDCl_3$) $\delta$ -69.18 (s, 3F). **HRMS** (ESI) m/z: $[M+H]^+$ Calcd for $C_{21}H_{28}BrF_3NO^+$ 446.1306; Found 446.1313.

***N*-(2-((adamantan-1-yl)methyl)-3,3,3-trifluoropropyl)-4-methoxy-2-meth-yl-aniline (41)**

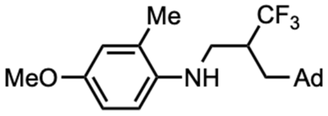


Using the General Procedure C, the title compound was obtained as a yellow viscous oil by preparative TLC using petroleum ether/EtOAc (15:1) as an eluent; R*f*= 0.6 (petroleum ether/EtOAc = 10:1); 27.2 mg, 48% yield. **$^{1}$H NMR** (400 MHz, $CDCl_3$) $\delta$ 6.73 – 6.70 (m, 2H), 6.56 – 6.54 (m, 1H), 3.75 (s, 3H), 3.45 (brs, 1H), 3.33 – 3.20 (m, 2H), 2.54 – 2.47 (m, 1H), 2.14 (s, 3H), 1.99 – 1.96 (m, 3H), 1.73 – 1.70 (m, 3H), 1.64 – 1.59 (m, 3H), 1.55 – 1.46 (m, 7H), 1.24 – 1.19 (m, 1H). **$^{13}$C NMR** (101 MHz, $CDCl_3$) $\delta$ 152.1, 139.9, 128.7 (q, $^{1}J_{CF}$ = 279.9 Hz), 124.5, 117.3, 111.7, 111.0, 55.9, 45.7, 42.3, 40.8, 37.2 (q, $^{2}J_{CF}$ = 24.4 Hz), 37.0, 32.5, 28.6, 17.8. **$^{19}$F NMR** (376 MHz, $CDCl_3$) $\delta$ -69.18 (s, 3F). **HRMS** (ESI) m/z: $[M+H]^+$ Calcd for $C_{22}H_{31}F_3NO^+$ 382.2358; Found 382.2365.

***N*-(2-((adamantan-1-yl)methyl)-3,3,3-trifluoropropyl)-2-fluoro-4-methoxyaniline (42)**

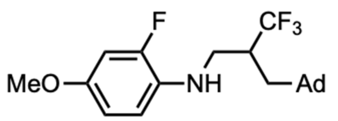

Using the General Procedure C, the title compound was obtained as a yellow viscous oil by preparative TLC using petroleum ether/EtOAc (15:1) as an eluent; $R_f$ = 0.6 (petroleum ether/EtOAc = 10:1); 27.2 mg, 48% yield. **$^1$H NMR** (600 MHz, $CDCl_3$) $\delta$ 6.67 – 6.60 (m, 3H), 3.78 (brs, 1H), 3.74 (s, 3H), 3.35 – 3.19 (m, 2H), 2.49 – 2.42 (m, 1H), 1.98 – 1.96 (m, 3H), 1.72 – 1.70 (m, 3H), 1.63 – 1.59 (m, 3H), 1.52 – 1.45 (m, 7H), 1.20 – 1.16 (m, 1H). **$^{13}$C NMR** (101 MHz, $CDCl_3$) $\delta$ 152.2 (d, $^1J_{CF}$ = 239.9 Hz), 152.1 (d, $^3J_{CF}$ = 9.8 Hz), 130.0 (d, $^3J_{CF}$ = 12.4 Hz), 128.6 (q, $^1J_{CF}$ = 282.8 Hz), 127.2, 113.2 (d, $^4J_{CF}$ = 4.5 Hz), 109.4 (d, $^4J_{CF}$ = 3.3 Hz), 102.7 (d, $^2J_{CF}$ = 22.4 Hz), 56.0, 45.5, 42.3, 40.7, 37.2 (q, $^2J_{CF}$ = 24.4 Hz), 36.9, 32.4, 28.6. **$^{19}$F NMR** (470 MHz, $CDCl_3$) $\delta$ -69.43 (s, 3F), -132.73 (s, 1F). **HRMS** (ESI) m/z: $[M+H]^+$ Calcd for $C_{21}H_{28}F_4NO^+$ 386.2107; Found 386.2114.

***N*-(2-((adamantan-1-yl)methyl)-3,3,3-trifluoropropyl)-4-methoxy-3,5-di-methyl-aniline (43)**

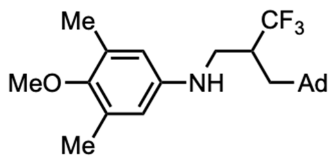

Using the General Procedure C, the title compound was obtained as a yellow viscous oil by preparative TLC using petroleum ether/EtOAc (15:1) as an eluent; $R_f$= 0.6 (petroleum ether/EtOAc = 10:1); 37.3 mg, 63% yield. **$^1$H NMR** (400 MHz, $CDCl_3$) $\delta$ 6.30 (s, 2H), 3.67 (s, 3H), 3.29 – 3.17 (m, 2H), 2.48 – 2.37 (m, 1H), 2.23 (s, 6H), 1.99 – 1.98 (m, 3H), 1.73 – 1.70 (m, 3H), 1.64 – 1.54 (m, 3H), 1.52 – 1.43 (m, 7H), 1.18 – 1.13 (m, 1H). (Due to the broadening effect and intermolecular hydrogen bonding, one of the proton signals of NH could not be observed.). **$^{13}$C NMR** (151 MHz, $CDCl_3$) $\delta$ 149.4, 143.6, 131.7, 128.7 (q, $^1J_{CF}$ = 279.6 Hz), 113.4, 60.2, 45.6, 42.3, 40.6, 37.2 (q, $^2J_{CF}$ = 24.7 Hz), 37.0, 32.5, 28.6, 16.4. **$^{19}$F NMR** (376 MHz, $CDCl_3$) $\delta$ -69.23 (s, 3F). **HRMS** (ESI) m/z: $[M+H]^+$ Calcd for $C_{23}H_{33}F_3NO^+$ 396.2514; Found 396.2523.

***N*-(2-((adamantan-1-yl)methyl)-3,3,3-trifluoropropyl)naphthalen-2-amine (44)**

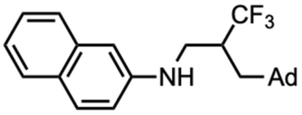


Using the General Procedure C, the title compound was obtained as a red amorphous solid by preparative TLC using petroleum ether/EtOAc (15:1) as an eluent; $R_f$= 0.6 (petroleum ether/EtOAc = 10:1); 25.0 mg, 43% yield. **$^1$H NMR** (400 MHz, $CDCl_3$) $\delta$ 7.69 – 7.60 (m, 3H), 7.39 – 7.35 (m, 1H), 7.24 – 7.19 (m, 1H), 6.88 – 6.81 (m, 2H), 4.02 (brs, 1H), 3.46 – 3.32 (m, 2H), 2.60 – 2.47 (m, 1H), 1.99 – 1.96 (m, 3H), 1.72 – 1.69 (m, 3H), 1.63 – 1.58 (m, 3H), 1.55 – 1.47 (m, 7H), 1.23 – 1.18 (m, 1H). **$^{13}$C NMR** (151 MHz, $CDCl_3$) $\delta$ 145.3, 135.2, 129.3, 128.7 (q, $^1J_{CF}$ = 282.8 Hz), 127.9, 127.8, 126.6, 126.1, 122.4, 118.2, 104.7, 44.9, 42.4, 40.8, 37.1 (q, $^2J_{CF}$ = 24.2 Hz), 37.0, 32.5, 28.6. **$^{19}$F NMR** (376 MHz, $CDCl_3$) $\delta$ -69.16 (s, 3F). **HRMS** (ESI) m/z: $[M+H]^+$ Calcd for $C_{24}H_{29}F_3N^+$ 388.2252; Found 388.2261.

***N*-(2-((adamantan-1-yl)methyl)-3,3,3-trifluoropropyl)-6-methoxypyridin-3-amine (45)**

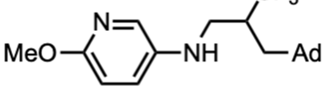


Using the General Procedure C (Variation 5), the title compound was obtained as a green amorphous solid by preparative TLC using petroleum ether/EtOAc (10:1) as an eluent; $R_f$= 0.5 (petroleum ether/EtOAc = 10:1); 20.4 mg, 37% yield. **$^1$H NMR** (400 MHz, $CDCl_3$) $\delta$ 7.57 (s, 1H), 6.99 (d, $J$ = 8.4 Hz, 1H), 6.64 (d, $J$ = 8.6 Hz, 1H), 3.87 (s, 3H), 3.32 – 3.16 (m, 2H), 2.45 – 2.41 (m, 1H), 1.97 – 1.96 (m, 3H), 1.72 – 1.68 (m, 3H), 1.61 – 1.58 (m, 3H), 1.52 – 1.42 (m, 7H), 1.18 – 1.13 (m, 1H). (Due to the broadening effect and intermolecular hydrogen bonding, one of the proton signals of NH could not be observed.). **$^{13}$C NMR** (101 MHz, $CDCl_3$) $\delta$ 157.9, 138.5, 130.9, 128.6 (q, $^1J_{CF}$ = 279.5 Hz), 126.4, 111.1, 53.5, 46.1, 42.3, 40.6, 37.2 (q, $^2J_{CF}$ = 24.3 Hz), 36.9, 32.5, 28.5. **$^{19}$F NMR** (376 MHz, $CDCl_3$) $\delta$ -69.23 (s, 3F). **HRMS** (ESI) m/z: $[M+H]^+$ Calcd for $C_{20}H_{28}F_3N_2O^+$ 369.2154; Found 369.2163.

***N*-(2-((adamantan-1-yl)methyl)-3,3,3-trifluoropropyl)-4-(1H-pyrrol-1-yl)-aniline (46)**

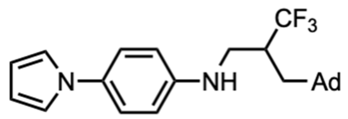

Using the General Procedure C, the title compound was obtained as a yellow amorphous solid by preparative TLC using petroleum ether/EtOAc (15:1) as an eluent; R*f* = 0.6 (petroleum ether/EtOAc = 10:1); 25.0 mg, 41% yield. **$^1$H NMR** (400 MHz, $CDCl_3$) $\delta$ 7.23 (d, $J$ = 8.8 Hz, 2H), 6.99 – 6.98 (m, 2H), 6.66 (d, $J$ = 8.8 Hz, 2H), 6.32 – 6.30 (m, 2H), 3.90 (brs, 1H), 3.39 – 3.24 (m, 2H), 2.53 – 2.44 (m, 1H), 2.00 – 1.97 (m, 3H), 1.74 – 1.71 (m, 3H), 1.64 – 1.60 (m, 3H), 1.55 – 1.46 (m, 7H), 1.21 – 1.16 (m, 1H). **$^{13}$C NMR** (101 MHz, $CDCl_3$) $\delta$ 145.8, 132.5, 128.6 (q, $^1J_{CF}$ = 279.9 Hz), 122.6, 119.8, 113.6, 109.6, 45.2, 42.4, 40.6, 37.2 (q, $^2J_{CF}$ = 24.4 Hz), 36.9, 32.5, 28.6. **$^{19}$F NMR** (376 MHz, $CDCl_3$) $\delta$ -69.20 (s, 3F). **HRMS** (ESI) m/z: $[M+H]^+$ Calcd for $C_{24}H_{30}F_3N_2^+$ 403.2361; Found 403.2370.

***N*-(2-((adamantan-1-yl)methyl)-3,3,3-trifluoropropyl)benzo[b]thiophen-6-amine (47)**

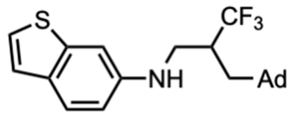

Using the General Procedure C, the title compound was obtained as a yellow viscous oil by preparative TLC using petroleum ether/EtOAc (15:1) as an eluent; R*f* = 0.6 (petroleum ether/EtOAc = 10:1); 21.4 mg, 36% yield. **$^1$H NMR** (400 MHz, $CDCl_3$) $\delta$ 7.65 (d, $J$ = 8.7 Hz, 1H), 7.39 (d, $J$ = 5.4 Hz, 1H), 7.18 (d, $J$ = 5.4 Hz, 1H), 7.00 (d, $J$ = 2.3 Hz, 1H), 6.73 (dd, $J$ = 8.7, 2.4 Hz, 1H). 3.91 (brs, 1H), 3.42 – 3.27 (m, 2H), 2.54 – 2.47 (m, 1H), 1.99 – 1.96 (m, 3H), 1.72 – 1.69 (m, 3H), 1.63 – 1.59 (m, 3H), 1.55 – 1.47 (m, 7H), 1.23 – 1.18 (m, 1H). **$^{13}$C NMR** (151 MHz, $CDCl_3$) $\delta$ 145.1, 141.1, 130.0, 128.7 (q, $^1J_{CF}$ = 280.1 Hz), 127.2, 123.4, 123.1, 114.3, 105.2, 45.5, 42.4, 40.8, 37.0, 36.9 (q, $^2J_{CF}$ = 25.0 Hz), 32.5, 28.6. **$^{19}$F NMR** (376 MHz, $CDCl_3$) $\delta$ -69.20 (s, 3F). **HRMS** (ESI) m/z: $[M+H]^+$ Calcd for $C_{22}H_{27}F_3NS^+$ 394.1816; Found 394.1823.

***Tert*-butyl 6-((2-((adamantan-1-yl)methyl)-3,3,3-trifluoropropyl)amino)-1*H*-indole-1-carboxylate (48)**

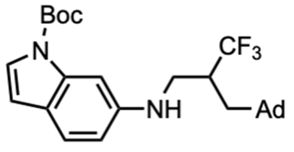

Using the General Procedure C (Variation 5), the title compound was obtained as a yellow viscous oil by preparative TLC using petroleum ether/EtOAc (10:1) as an eluent; $R_f$= 0.5 (petroleum ether/EtOAc = 10:1); 41.0 mg, 57% yield. **$^1$H NMR** (400 MHz, $CDCl_3$) $\delta$ 7.93 – 7.92 (m, 1H), 7.52 – 7.51 (m, 1H), 6.76 – 6.75 (m, 1H), 6.68 – 6.65 (m, 1H), 6.43 – 6.41 (m, 1H), 3.40 – 3.25 (m, 2H), 2.53 – 2.46 (m, 1H), 1.98 – 1.96 (m, 3H), 1.72 – 1.59 (m, 15H), 1.51 – 1.46 (m, 7H), 1.22 – 1.17 (m, 1H). (Due to the broadening effect and intermolecular hydrogen bonding, one of the proton signals of NH could not be observed.). **$^{13}$C NMR** (151 MHz, $CDCl_3$) $\delta$ 149.9, 143.7, 131.8, 128.7 (q, $^1J_{CF}$ = 280.1 Hz), 126.4, 116.0, 115.8, 112.8, 107.1, 103.3, 83.4, 45.9, 42.3, 40.8, 37.1 (q, $^2J_{CF}$ = 24.4 Hz), 37.0, 32.5, 28.6, 28.4. **$^{19}$F NMR** (376 MHz, $CDCl_3$) $\delta$ -69.19 (s, 3F). **HRMS** (ESI) m/z: $[M+H]^+$ Calcd for $C_{27}H_{36}F_3N_2O_2^+$ 477.2729; Found 477.2728.

**$N$-(2-((adamantan-1-yl)methyl)-3,3,3-trifluoropropyl)-1-benzyl-1H-indazol-6-amine (49)**

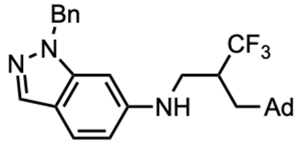

Using the General Procedure C (Variation 5), the title compound was obtained as a green viscous oil by preparative TLC using petroleum ether/EtOAc (8:1) as an eluent; $R_f$= 0.4 (petroleum ether/EtOAc = 10:1); 41.0 mg, 57% yield. **$^1$H NMR** (400 MHz, $CDCl_3$) $\delta$ 7.89 (s, 1H), 7.33 – 7.27 (m, 3H), 7.20 – 7.17 (m, 3H), 6.83 – 6.77 (m, 2H), 5.56 (s, 2H), 3.83 (brs, 1H), 3.41 – 3.25 (m, 2H), 2.56 – 2.49 (m, 1H), 2.00 – 1.97 (m, 3H), 1.74 – 1.71 (m, 3H), 1.64 – 1.61 (m, 3H), 1.55 – 1.47 (m, 7H), 1.25 – 1.20 (m, 1H). **$^{13}$C NMR** (101 MHz, $CDCl_3$) $\delta$ 142.2, 137.2, 135.1, 132.1, 128.8, 128.7 (q, $^1J_{CF}$ = 280.2 Hz), 127.8, 127.2, 125.4, 118.6, 110.5, 100.0, 53.2, 45.9, 42.3, 40.7, 37.1 (q, $^2J_{CF}$ = 24.4 Hz), 36.9, 32.5, 28.5. **$^{19}$F NMR** (376 MHz, $CDCl_3$) $\delta$ -69.20 (s, 3F). **HRMS** (ESI) m/z: $[M+H]^+$ Calcd for $C_{28}H_{33}F_3N_3^+$ 468.2627; Found 468.2635.

***N*-(2-((adamantan-1-yl)methyl)-3,3,3-trifluoropropyl)benzo[*d*][1,3]dioxol-5-amine (50)**

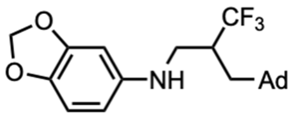

Using the General Procedure C, the title compound was obtained as a brown viscous oil by preparative TLC using petroleum ether/EtOAc (15:1) as an eluent; R*f* = 0.6 (petroleum ether/EtOAc = 10:1); 30.2 mg, 53% yield. **$^{1}$H NMR** (400 MHz, $CDCl_3$) $\delta$ 6.68 – 6.65 (m, 1H), 6.25 – 6.24 (m, 1H), 6.06 – 6.03 (m, 1H), 5.86 – 5.85 (m, 2H), 3.64 (brs, 1H), 3.29 – 3.13 (m, 2H), 2.47 – 2.41 (m, 1H), 1.98 – 1.95 (m, 3H), 1.72 – 1.68 (m, 3H), 1.62 – 1.59 (m, 3H), 1.52 – 1.41 (m, 7H), 1.17 – 1.11 (m, 1H). **$^{13}$C NMR** (151 MHz, $CDCl_3$) $\delta$ 148.6, 143.3, 140.2, 128.7 (q, $^{1}J_{CF}$ = 279.7 Hz), 108.8, 104.9, 100.8, 96.5, 46.1, 42.3, 40.7, 37.1 (q, $^{2}J_{CF}$ = 24.5 Hz), 37.0, 32.5, 28.6. **$^{19}$F NMR** (376 MHz, $CDCl_3$) $\delta$ -69.25 (s, 3F). **HRMS** (ESI) m/z: $[M+H]^+$ Calcd for $C_{21}H_{27}F_3NO_2^+$ 382.1994; Found 382.1996.

***N*-(2-((adamantan-1-yl)methyl)-3,3,3-trifluoropropyl)-2,2-difluorobenzo-[d][1,3] dioxol-5-amine (51)**

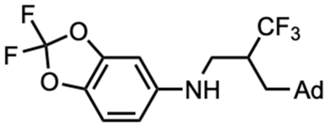

Using the General Procedure C, the title compound was obtained as a yellow viscous oil by preparative TLC using petroleum ether/EtOAc (15:1) as an eluent; R*f*= 0.6 (petroleum ether/EtOAc = 10:1); 26.5 mg, 43% yield. **$^{1}$H NMR** (400 MHz, $CDCl_3$) $\delta$ 6.86 (d, $J$ = 8.6 Hz, 1H), 6.38 – 6.37 (m, 1H), 6.25 – 6.23 (m, 1H), 3.84 (brs, 1H), 3.30 – 3.15 (m, 2H), 2.48 – 2.38 (m, 1H), 1.99 – 1.97 (m, 3H), 1.73 – 1.70 (m, 3H), 1.63 – 1.60 (m, 3H), 1.53 – 1.45 (m, 7H), 1.17 – 1.12 (m, 1H). **$^{13}$C NMR** (101 MHz, $CDCl_3$) $\delta$ 144.9, 144.7, 136.3, 131.9 (t, $^{1}J_{CF}$ = 253.6 Hz), 128.5 (q, $^{1}J_{CF}$ = 279.9 Hz), 110.0, 106.9, 95.7, 45.6, 42.3, 40.6, 37.1 (q, $^{2}J_{CF}$ = 24.6 Hz), 36.9, 32.5, 28.5. **$^{19}$F NMR** (376 MHz, $CDCl_3$) $\delta$ -50.44 (s, 2F), -69.28 (s, 3F). **HRMS** (ESI) m/z: $[M+H]^+$ Calcd for $C_{22}H_{25}F_5NO_2^+$ 418.1805; Found 418.1812.

**$N$-(2-((adamantan-1-yl)methyl)-3,3,3-trifluoropropyl)dibenzo[*b*,*d*]furan-3-amine (52)**

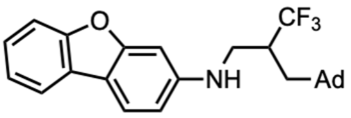


Using the General Procedure C (Variation 2), the title compound was obtained as a yellow viscous oil by preparative TLC using petroleum ether/EtOAc (10:1) as an eluent; $R_f$= 0.5 (petroleum ether/EtOAc = 10:1); 21.2 mg, 33% yield. **$^1$H NMR** (400 MHz, $CDCl_3$) $\delta$ 7.79 (d, $J$ = 8.1 Hz, 1H), 7.71 (d, $J$ = 8.3 Hz, 1H), 7.48 (d, $J$ = 7.4 Hz, 1H), 7.37 – 7.27 (m, 2H), 6.76 (s, 1H), 6.63 (dd, $J$ = 8.4, 2.1 Hz, 1H), 4.16 (brs, 1H), 3.45 – 3.30 (m, 2H), 2.56 – 2.49 (m, 1H), 1.99 – 1.96 (m, 3H), 1.72 – 1.69 (m, 3H), 1.63 – 1.46 (m, 10H), 1.23 – 1.18 (m, 1H). **$^{13}$C NMR** (101 MHz, $CDCl_3$) $\delta$ 158.4, 156.0, 148.0, 128.6 (q, $^1J_{CF}$ = 279.9 Hz), 125.1, 125.0, 122.7, 121.4, 119.4, 115.0, 111.3, 110.3, 94.6, 45.1, 42.3, 40.7, 37.1 (q, $^2J_{CF}$ = 73.8 Hz), 36.9, 32.5, 28.5. **$^{19}$F NMR** (376 MHz, $CDCl_3$) $\delta$ -69.20 (s, 3F). **HRMS** (ESI) m/z: $[M+H]^+$ Calcd for $C_{26}H_{29}F_3NO^+$ 428.2201; Found 428.2205.

**4-(benzyloxy)-$N$-(2-(((1,3,5,7)-3,5-dimethyladamantan-1-yl)methyl)-3,3,3-trifluoropropyl)aniline (53)**

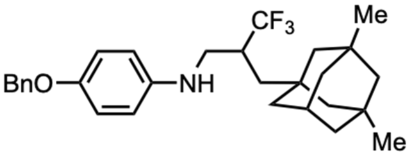


Using the General Procedure C, the title compound was obtained as a yellow viscous oil by preparative TLC using petroleum ether/EtOAc (5:1) as an eluent; $R_f$ = 0.2 (petroleum ether/EtOAc = 10:1); 51.9 mg, 71% yield. **$^1$H NMR** (600 MHz, $CDCl_3$) $\delta$ 7.44 – 7.42 (m, 2H), 7.40 – 7.37 (m, 2H), 7.33 – 7.31 (m, 1H), 6.89 – 6.87 (m, 2H), 6.60 – 6.58 (m, 2H), 5.01 (s, 2H), 3.33 – 3.18 (m, 2H), 2.46 – 2.39 (m, 1H), 2.07 – 2.05 (m, 1H), 1.52 – 1.49 (m, 1H), 1.33 – 1.28 (m, 6H), 1.21 – 1.17 (m, 1H), 1.16 – 1.08 (m, 4H), 1.07 – 1.04 (m, 2H), 0.81 (s, 6H). (Due to the broadening effect and intermolecular hydrogen bonding, one of the proton signals of NH could not be observed.). **$^{13}$C NMR** (151 MHz, $CDCl_3$) $\delta$ 151.8, 141.9, 137.7, 128.7 (q, $^1J_{CF}$ = 279.8 Hz), 128.6, 127.9, 127.6, 116.4, 114.6, 71.0, 51.1, 48.7, 48.6, 46.0, 43.2, 40.9, 40.0,

37.2 (q, $^{2}J_{CF}$ = 24.3 Hz), 34.2, 31.3, 30.7, 29.6. **$^{19}$F NMR** (376 MHz, $CDCl_3$) $\delta$ -69.19 (s, 3F). **HRMS** (ESI) m/z: $[M+H]^+$ Calcd for $C_{29}H_{37}F_3NO^+$ 472.2827; Found 472.2836.

**4-(benzyloxy)-*N*-(3,3,3-trifluoro-2-((3,5,7-trifluoroadamantan-1-yl)methyl)propyl)aniline (54)**

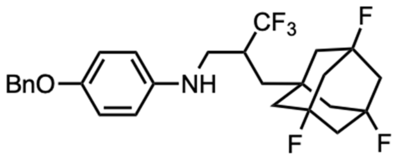


Using the General Procedure C, the title compound was obtained as a yellow viscous oil by preparative TLC using petroleum ether/EtOAc (10:1) as an eluent; $R_f$ = 0.5 (petroleum ether/EtOAc = 10:1); 25.2 mg, 34% yield. **$^{1}$H NMR** (600 MHz, $CDCl_3$) $\delta$ 7.43 – 7.36 (m, 4H), 7.33 – 7.30 (m, 1H), 6.89 – 6.87 (m, 2H), 6.59 – 6.57 (m, 2H), 5.01 (s, 2H), 3.55 (brs, 1H), 3.40 – 3.15 (m, 2H), 2.42 – 2.37 (m, 1H), 2.14 – 2.11 (m, 3H), 2.01 – 1.97 (m, 3H), 1.80 – 1.77 (m, 1H), 1.63 – 1.54 (m, 7H). **$^{13}$C NMR** (151 MHz, $CDCl_3$) $\delta$ 152.2, 141.3, 137.5, 128.7, 128.0, 127.9 (q, $^{1}J_{CF}$ = 280.0 Hz), 127.6, 116.5, 114.8, 92.5 (t, $^{2}J_{CF}$ = 15.4 Hz), 91.3 (t, $^{2}J_{CF}$ = 15.5 Hz), 70.9, 46.5 (t, $^{2}J_{CF}$ = 18.9 Hz), 45.6, 45.1 (d, $^{2}J_{CF}$ = 15.6 Hz), 37.9 (q, $^{2}J_{CF}$ = 24.5 Hz), 37.8, 36.3 (q, $^{3}J_{CF}$ = 11.0 Hz). **$^{19}$F NMR** (565 MHz, $CDCl_3$) $\delta$ -69.26 (s, 3F), -143.32 (s, 3F). **HRMS** (ESI) m/z: $[M+H]^+$ Calcd for $C_{27}H_{30}F_6NO^+$ 498.2232; Found 498.2242.

**4-(benzyloxy)-*N*-(2-(((1,3,5,7)-3-bromoadamantan-1-yl)methyl)-3,3,3-trifluoropropyl)aniline (55)**

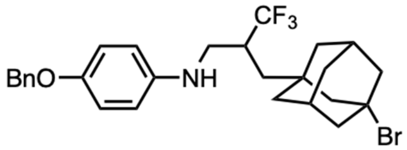


Using the General Procedure C, the title compound was obtained as a yellow viscous oil by preparative TLC using petroleum ether/EtOAc (10:1) as an eluent; $R_f$ = 0.5 (petroleum ether/EtOAc = 10:1); 25.7 mg, 33% yield. **$^{1}$H NMR** (400 MHz, $CDCl_3$) $\delta$ 7.44 – 7.29 (m, 5H), 6.90 – 6.83 (m, 2H), 6.61 – 6.56 (m, 2H), 5.01 (s, 2H), 3.36 – 3.15 (m, 2H), 2.45 – 2.36 (m, 1H), 2.32 – 2.29 (m, 2H), 2.24 – 2.20 (m, 2H), 2.17 – 2.05 (m, 4H),

1.69 – 1.44 (m, 8H). (Due to the broadening effect and intermolecular hydrogen bonding, one of the proton signals of NH could not be observed.). **$^{13}$C NMR** (151 MHz, $CDCl_3$) $\delta$ 152.0, 141.6, 137.6, 128.6, 128.4 (q, $^1J_{CF}$ = 280.1 Hz), 127.9, 127.6, 116.4, 114.7, 70.9, 65.2, 53.6, 48.5, 45.9, 40.3, 40.2, 39.7, 37.7, 37.2 (q, $^2J_{CF}$ = 24.8 Hz), 34.8, 32.3. **$^{19}$F NMR** (376 MHz, $CDCl_3$) $\delta$ -69.27 (s, 3F), -143.32 (s, 3F). **HRMS** (ESI) m/z: $[M+H]^+$ Calcd for $C_{27}H_{32}BrF_3NO^+$ 522.1619; Found 522.1624.

**5-(2-(((4-(benzyloxy)phenyl)amino)methyl)-3,3,3-trifluoropropyl)-adamantan-2-one (56)**

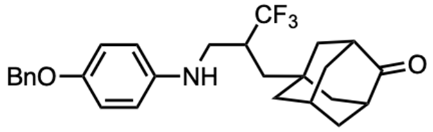


Using the General Procedure C, the title compound was obtained as a yellow viscous oil by preparative TLC using petroleum ether/EtOAc (10:1) as an eluent; $R_f$= 0.5 (petroleum ether/EtOAc = 10:1); 25.7 mg, 33% yield. **$^1$H NMR** (400 MHz, $CDCl_3$) $\delta$ 7.44 – 7.29 (m, 5H), 6.89 – 6.85 (m, 2H), 6.59 – 6.54 (m, 2H), 5.00 (s, 2H), 3.37 – 3.13 (m, 2H), 2.54 – 2.53 (m, 2H), 2.45 – 2.35 (m, 1H), 2.15 – 2.12 (m, 1H), 1.99 – 1.91 (m, 4H), 1.83 – 1.67 (m, 7H), 1.60 – 1.56 (m, 1H). (Due to the broadening effect and intermolecular hydrogen bonding, one of the proton signals of NH could not be observed.). **$^{13}$C NMR** (151 MHz, $CDCl_3$) $\delta$ 217.7, 152.0, 141.6, 137.6, 128.7, 128.3 (q, $^1J_{CF}$ = 279.9 Hz), 128.0, 127.6, 116.4, 114.5, 70.9, 46.3, 45.7, 43.3, 43.2, 40.9, 38.9, 38.6, 37.6 (q, $^2J_{CF}$ = 24.2 Hz), 32.6, 27.7. **$^{19}$F NMR** (376 MHz, $CDCl_3$) $\delta$ -69.33 (s, 3F). **HRMS** (ESI) m/z: $[M+H]^+$ Calcd for $C_{27}H_{31}F_3NO_2^+$ 458.2307; Found 458.2305.

**4-(benzyloxy)-*N*-(3,3,3-trifluoro-2-(((2,3,5,6)-hexahydro-2,5-methanopentalen-3a (1*H*)-yl)methyl)propyl)aniline (57)**

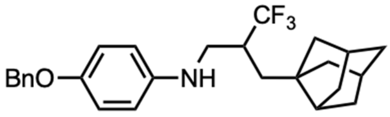


Using the General Procedure C, the title compound was obtained as a yellow viscous oil by preparative TLC using petroleum ether/EtOAc (15:1) as an eluent; $R_f$= 0.6 (petroleum ether/EtOAc = 10:1); 35.0 mg, 54% yield. **$^1$H NMR** (400

MHz, $CDCl_3$) $\delta$ 7.45 – 7.30 (m, 5H), 6.90 – 6.86 (m, 2H), 6.62 – 6.58 (m, 2H), 5.01 (s, 2H), 3.62 (brs, 1H), 3.34 – 3.32 (m, 2H), 2.50 – 2.40 (m, 1H), 2.23 – 2.22 (m, 2H), 2.03 – 2.00 (m, 1H), 1.94 – 1.90 (m, 1H), 1.75 – 1.52 (m, 11H). **$^{13}$C NMR** (101 MHz, $CDCl_3$) $\delta$ 151.8, 142.0, 137.6, 128.6, 128.6 (q, $^1J_{CF}$ = 280.3 Hz), 127.9, 127.6, 116.3, 114.6, 70.9, 49.1, 48.6, 48.3, 45.0, 44.4, 44.0, 43.9, 40.4 (q, $^2J_{CF}$ = 23.9 Hz), 37.9, 37.6, 35.5, 35.1. **$^{19}$F NMR** (376 MHz, $CDCl_3$) $\delta$ -69.16 (s, 3F). **HRMS** (ESI) m/z: $[M+H]^+$ Calcd for $C_{26}H_{31}F_3NO^+$ 430.2358; Found 430.2365.

**Methyl 4-(2-(((4-(benzyloxy)phenyl)amino)methyl)-3,3,3-trifluoropropyl)bicyclo [2.2.2]octane-1-carboxylate (58)**

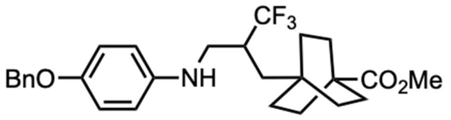


Using the General Procedure C, the title compound was obtained as a yellow amorphous solid by preparative TLC using petroleum ether/EtOAc (15:1) as an eluent; R*f* = 0.6 (petroleum ether/EtOAc = 10:1); 35.0 mg, 54% yield. **$^1$H NMR** (600 MHz, $CDCl_3$) $\delta$ 7.44 – 7.30 (m, 5H), 6.88 – 6.86 (m, 2H), 6.59 – 6.56 (m, 2H), 5.01 (s, 2H), 3.64 (s, 3H), 3.31 – 3.36 (m, 2H), 2.50 – 2.39 (m, 1H), 1.79 – 1.74 (m, 6H), 1.54 – 1.52 (m, 1H), 1.47 – 1.38 (m, 6H), 1.27 – 1.24 (m, 1H). (Due to the broadening effect and intermolecular hydrogen bonding, one of the proton signals of NH could not be observed.). **$^{13}$C NMR** (101 MHz, $CDCl_3$) $\delta$ 178.3, 151.8, 141.8, 137.6, 128.6, 128.5 (q, $^1J_{CF}$ = 280.0 Hz), 127.9, 127.6, 116.3, 114.5, 70.9, 51.8, 45.6, 38.9, 38.1 (q, $^2J_{CF}$ = 24.5 Hz), 37.6, 30.9, 30.4, 28.4. **$^{19}$F NMR** (376 MHz, $CDCl_3$) $\delta$ -69.29 (s, 3F). **HRMS** (ESI) m/z: $[M+H]^+$ Calcd for $C_{27}H_{33}F_3NO_3^+$ 476.2413; Found 476.2419.

**Methyl 5-(2-(((4-(benzyloxy)phenyl)amino)methyl)-3,3,3-trifluoropropyl)bicyclo [3.1.1]heptane-1-carboxylate (59)**

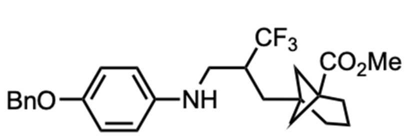


Using the General Procedure C, the title compound was obtained as a yellow viscous oil by preparative TLC using petroleum

ether/EtOAc (15:1) as an eluent; $R_f$= 0.6 (petroleum ether/EtOAc = 10:1); 22.8 mg, 33% yield. **$^1$H NMR** (400 MHz, $CDCl_3$) $\delta$ 7.44 – 7.30 (m, 5H), 6.87 (d, $J$ = 8.5 Hz, 2H), 6.56 (d, $J$ = 8.5 Hz, 2H), 5.00 (s, 1H), 3.66 (s, 3H), 3.61 (brs, 1H), 3.31 – 3.16 (m, 2H), 2.51 – 2.37 (m, 1H), 2.02 – 1.93 (m, 4H), 1.87 – 1.80 (m, 2H), 1.76 – 1.61 (m, 5H), 1.58 – 1.52 (m, 1H). **$^{13}$C NMR** (201 MHz, $CDCl_3$) $\delta$ 176.2, 151.9, 141.7, 137.6, 128.7, 128.2 (q, $^1J_{CF}$ = 280.4 Hz), 128.0, 127.6, 116.4, 114.5, 70.9, 51.8, 44.3, 43.1, 40.4, 40.3, 38.9 (q, $^2J_{CF}$ = 24.4 Hz), 37.8, 37.2, 32.3, 30.0, 16.7. **$^{19}$F NMR** (376 MHz, $CDCl_3$) $\delta$ -69.27 (s, 3F). **HRMS** (ESI) m/z: $[M+H]^+$ Calcd for $C_{26}H_{31}F_3NO_3^+$ 462.2256; Found 462.2254.

**Methyl 3-(2-(((4-(benzyloxy)phenyl)amino)methyl)-3,3,3-trifluoropropyl)bicyclo [1.1.1]pentane-1-carboxylate (60)**

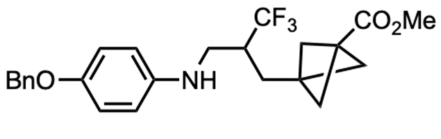


Using the General Procedure C (Variation 2), the title compound was obtained as a yellow viscous oil by preparative TLC using petroleum ether/EtOAc (15:1) as an eluent; $R_f$= 0.6 (petroleum ether/EtOAc = 10:1); 25.6 mg, 40% yield. **$^1$H NMR** (400 MHz, $CDCl_3$) $\delta$ 7.44 – 7.29 (m, 5H), 6.89 – 6.85 (m, 2H), 6.59 – 6.55 (m, 2H), 5.00 (s, 2H), 3.66 (s, 3H), 3.38 – 3.18 (m, 2H), 2.42 – 2.32 (m, 1H), 1.96 (s, 6H), 1.92 – 1.87 (m, 1H), 1.75 – 1.69 (m, 1H). (Due to the broadening effect and intermolecular hydrogen bonding, one of the proton signals of NH could not be observed.). **$^{13}$C NMR** (101 MHz, $CDCl_3$) $\delta$ 170.3, 152.0, 141.6, 137.6, 128.6, 128.0, 127.9 (q, $^1J_{CF}$ = 280.4 Hz), 127.6, 116.4, 114.6, 70.9, 52.2, 51.8, 43.8, 40.7 (q, $^2J_{CF}$ = 24.6 Hz), 38.7, 38.0, 28.3. **$^{19}$F NMR** (376 MHz, $CDCl_3$) $\delta$ -69.62 (s, 3F). **HRMS** (ESI) m/z: $[M+H]^+$ Calcd for $C_{24}H_{27}F_3NO_3^+$ 434.1943; Found 434.1952.

**Methyl 4-(2-(((4-(benzyloxy)phenyl)amino)methyl)-3,3,3-trifluoropropyl)cubane -1-carboxylate (61)**

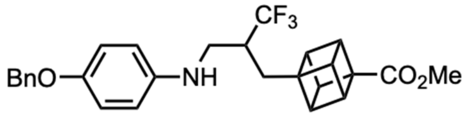

Using the General Procedure C, the title compound was obtained as a yellow amorphous solid by preparative TLC using petroleum ether/EtOAc (15:1) as an eluent; R*f*= 0.6 (petroleum ether/EtOAc = 10:1); 19.1 mg, 27% yield. **$^1$H NMR** (400 MHz, $CDCl_3$) $\delta$ 7.44 – 7.29 (m, 5H), 6.89 – 6.85 (m, 2H), 6.58 – 6.53 (m, 2H), 5.00 (s, 2H), 4.12 – 4.08 (m, 3H), 3.77 – 3.72 (m, 3H), 3.70 (s, 3H), 3.36 – 3.21 (m, 2H), 2.50 – 2.40 (m, 1H), 2.01 – 1.88 (m, 2H). (Due to the broadening effect and intermolecular hydrogen bonding, one of the proton signals of NH could not be observed.). **$^{13}$C NMR** (151 MHz, $CDCl_3$) $\delta$ 172.8, 151.9, 141.6, 137.6, 128.7, 128.0 (q, $^1J_{CF}$ = 279.3 Hz), 127.6, 127.1, 116.4, 114.3, 71.0, 57.6, 56.1, 51.7, 46.6, 46.2, 43.7, 39.9 (q, $^2J_{CF}$ = 24.4 Hz), 29.9. **$^{19}$F NMR** (376 MHz, $CDCl_3$) $\delta$ -69.52 (s, 3F). **HRMS** (ESI) m/z: $[M+H]^+$ Calcd for $C_{27}H_{27}F_3NO_3^+$ 470.1943; Found 470.1951.

**4-(benzyloxy)-*N*-(3,3,3-trifluoro-2-((1-methylcyclohexyl)methyl)propyl)aniline (62)**

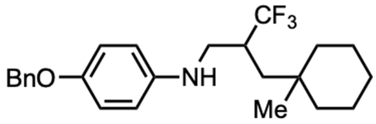

Using the General Procedure C, the title compound was obtained as a yellow viscous oil by preparative TLC using petroleum ether/EtOAc (15:1) as an eluent; R*f*= 0.6 (petroleum ether/EtOAc = 10:1); 18.3 mg, 30% yield. **$^1$H NMR** (400 MHz, $CDCl_3$) $\delta$ 7.44 – 7.26 (m, 5H), 6.90 – 6.85 (m, 2H), 6.62 – 6.57 (m, 2H), 5.01 (s, 2H), 3.64 (brs, 1H), 3.34 – 3.22 (m, 2H), 2.42 – 2.39 (m, 1H), 1.64 – 1.58 (m, 1H), 1.47 – 1.23 (m, 11H), 0.92 – 0.89 (m, 3H). **$^{13}$C NMR** (201 MHz, $CDCl_3$) $\delta$ 151.8, 142.0, 137.7, 128.8 (q, $^1J_{CF}$ = 280.2 Hz), 128.7, 128.0, 127.6, 116.3, 114.6, 71.0, 46.0, 38.2 (q, $^2J_{CF}$ = 23.9 Hz), 37.8, 37.6, 33.2, 26.4, 22.1, 22.0. **$^{19}$F NMR** (376 MHz, $CDCl_3$) $\delta$ -69.03 (s, 3F). **HRMS** (ESI) m/z: $[M+H]^+$ Calcd for $C_{24}H_{31}F_3NO^+$ 406.2358; Found 406.2362.

**4-(benzyloxy)-*N*-(3,3,3-trifluoro-2-((4-methyltetrahydro-2*H*-pyran-4-yl)methyl)-propyl)aniline (63)**

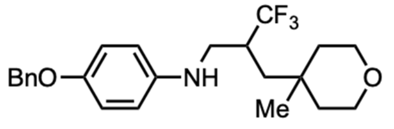


Using the General Procedure C, the title compound was obtained as a yellow viscous oil by preparative TLC using petroleum ether/EtOAc (10:1) as an eluent; $R_f$= 0.5 (petroleum ether/EtOAc = 10:1); 19.2 mg, 31% yield. **$^1$H NMR** (400 MHz, $CDCl_3$) $\delta$ 7.44 – 7.29 (m, 5H), 6.89 – 6.84 (m, 2H), 6.60 – 6.55 (m, 2H), 5.00 (s, 2H), 3.74 – 3.69 (m, 2H), 3.63 – 3.56 (m, 2H), 3.38 – 3.21 (m, 2H), 2.46 – 2.37 (m, 1H), 1.73 – 1.68 (m, 1H), 1.53 – 1.46 (m, 2H), 1.42 – 1.37 (m, 1H), 1.34 – 1.28 (m, 2H), 1.01 (s, 3H). (Due to the broadening effect and intermolecular hydrogen bonding, one of the proton signals of NH could not be observed.). **$^{13}$C NMR** (201 MHz, $CDCl_3$) $\delta$ 151.9, 141.7, 137.6, 128.7, 128.5 (q, $^1J_{CF}$ = 280.0 Hz), 128.0, 127.6, 116.4, 114.6, 70.9, 63.8, 45.9, 37.9 (q, $^2J_{CF}$ = 24.0 Hz), 37.7, 37.3, 31.1, 29.8. **$^{19}$F NMR** (376 MHz, $CDCl_3$) $\delta$ -69.07 (s, 3F). **HRMS** (ESI) m/z: $[M+H]^+$ Calcd for $C_{23}H_{29}F_3NO_2^+$ 408.2150; Found 408.2157.

**4-(benzyloxy)-*N*-(4,4-dimethyl-2-(trifluoromethyl)heptyl)aniline (64)**

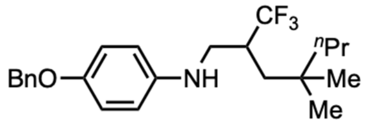


Using the General Procedure C, the title compound was obtained as a yellow viscous oil by preparative TLC using petroleum ether/EtOAc (15:1) as an eluent; $R_f$= 0.6 (petroleum ether/EtOAc = 10:1); 13.0 mg, 22% yield. **$^1$H NMR** (400 MHz, $CDCl_3$) $\delta$ 7.44 – 7.30 (m, 5H), 6.88 – 6.86 (m, 2H), 6.60 – 6.57 (m, 2H), 5.00 (s, 2H), 3.61 (brs, 1H), 3.33 – 3.21 (m, 2H), 2.40 – 2.36 (m, 1H), 1.62 – 1.57 (m, 1H), 1.32 – 1.28 (m, 3H), 1.20 – 1.15 (m, 2H), 0.90 – 0.87 (m, 9H). **$^{13}$C NMR** (201 MHz, $CDCl_3$) $\delta$ 151.8, 141.9, 137.6, 128.7 (q, $^1J_{CF}$ = 280.1 Hz), 128.6, 127.9, 127.6, 116.3, 114.6, 70.9, 45.9, 44.9, 38.7 (q, $^2J_{CF}$ = 24.0 Hz), 38.2, 33.3, 26.8, 17.3, 15.0. **$^{19}$F NMR** (376 MHz, $CDCl_3$) $\delta$ -69.10 (s, 3F). **HRMS** (ESI) m/z: $[M+H]^+$ Calcd for $C_{23}H_{31}F_3NO^+$ 394.2358; Found 394.2362.

**N-(2-(((1,2,5)-adamantan-2-yl)methyl)-3,3,3-trifluoropropyl)-4-(benzyloxy)-aniline (65)**

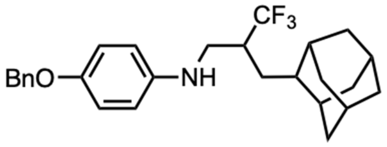

Using the General Procedure C (Variation 1), the title compound was obtained as a yellow viscous oil by preparative TLC using petroleum ether/EtOAc (15:1) as an eluent; R*f*= 0.5 (petroleum ether/EtOAc = 10:1); 26.4 mg, 40% yield. **$^1$H NMR** (400 MHz, $CDCl_3$) $\delta$ 7.44 – 7.29 (m, 5H), 6.89 – 6.85 (m, 2H), 6.59 – 6.55 (m, 2H), 5.00 (s, 2H), 3.61 (brs, 1H), 3.38 – 3.22 (m, 2H), 2.40 – 2.33 (m, 1H), 1.91 – 1.82 (m, 8H), 1.76 – 1.69 (m, 5H), 1.54 – 1.46 (m, 3H), 0.86 – 0.83 (m, 1H). **$^{13}$C NMR** (201 MHz, $CDCl_3$) $\delta$ 151.8, 141.8, 137.6, 128.7, 128.6 (q, $^1J_{CF}$ = 280.4 Hz), 128.0, 127.6, 116.4, 114.4, 71.0, 43.7, 41.5, 40.2 (q, $^2J_{CF}$ = 23.8 Hz), 39.3, 39.1, 38.3, 32.6, 31.6, 31.2, 31.0, 29.3, 28.2, 28.0. **$^{19}$F NMR** (376 MHz, $CDCl_3$) $\delta$ -69.63 (s, 3F). **HRMS** (ESI) m/z: $[M+H]^+$ Calcd for $C_{27}H_{33}F_3NO^+$ 444.2514; Found 444.2518.

**4-(benzyloxy)-N-(4-methyl-2-(trifluoromethyl)pentyl)aniline (66)**

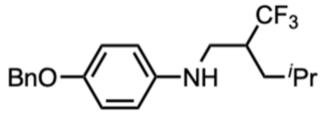

Using the General Procedure C (Variation 1), the title compound was obtained as a yellow viscous oil by preparative TLC using petroleum ether/EtOAc (15:1) as an eluent; R*f*= 0.6 (petroleum ether/EtOAc = 10:1); 16.1 mg, 31% yield. **$^1$H NMR** (400 MHz, $CDCl_3$) $\delta$ 7.44 – 7.30 (m, 5H), 6.89 – 6.85 (m, 2H), 6.60 – 6.56 (m, 2H), 5.00 (s, 2H), 3.60 (brs, 1H), 3.36 – 3.19 (m, 2H), 2.46 – 2.38 (m, 1H), 1.80 – 1.69 (m, 1H), 1.53 – 1.46 (m, 1H), 1.41 – 1.34 (m, 1H), 0.93 – 0.91 (m, 6H). **$^{13}$C NMR** (101 MHz, $CDCl_3$) $\delta$ 151.8, 141.8, 137.6, 128.7, 128.5 (q, $^1J_{CF}$ = 280.4 Hz), 128.0, 127.6, 116.3, 114.4, 70.9, 43.8, 40.4 (q, $^2J_{CF}$ = 24.1 Hz), 35.7, 25.7, 23.0, 22.2. **$^{19}$F NMR** (376 MHz, $CDCl_3$) $\delta$ -69.54 (s, 3F). **HRMS** (ESI) m/z: $[M+H]^+$ Calcd for $C_{20}H_{25}F_3NO^+$ 352.1888; Found 352.1894.

**4-(benzyloxy)-N-(4-ethyl-2-(trifluoromethyl)octyl)aniline (67)**

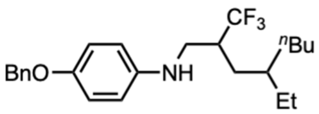

Using the General Procedure C (Variation 1), the title compound was obtained as a yellow viscous oil by preparative TLC using petroleum ether/EtOAc (15:1) as an eluent; $R_f$= 0.6 (petroleum ether/EtOAc = 10:1); 28.2 mg, 46% yield (d.r. = 1.1:1). **$^1$H NMR** (400 MHz, $CDCl_3$) $\delta$ 7.43 – 7.29 (m, 5H), 6.87 – 6.82 (m, 2H), 6.58 – 6.54 (m, 2H), 4.99 (s, 2H), 3.53 (brs, 1H), 3.35 – 3.18 (m, 2H), 2.47 – 2.33 (m, 1H), 1.54 – 1.48 (m, 1H), 1.42 – 1.23 (m, 9H), 0.92 – 0.82 (m, 7H). **$^{13}$C NMR** (101 MHz, $CDCl_3$) $\delta$ 151.8, 151.1, 143.5, 141.8, 137.6, 128.7, 128.6, 128.5 (q, $^1J_{CF}$ = 280.4 Hz), 128.0, 127.9, 127.6, 116.3, 116.3, 114.4, 113.9, 71.0, 70.9, 48.1, 43.8, 40.2 (q, $^2J_{CF}$ = 23.6 Hz), 39.2, 36.5, 36.4, 32.9, 32.5, 30.6, 30.4, 28.8, 28.5, 26.0, 25.5, 23.3, 23.2, 14.3, 14.2, 10.7, 10.3. (All $^{13}$C NMR signals were listed without classifying to the corresponding diastereomers due to the complexity). **$^{19}$F NMR** (376 MHz, $CDCl_3$) $\delta$ -69.48, -69.54. Diastereomeric ratio (d.r.) = 1.1:1 (according to the ratio of integration of the two $^{19}$F NMR signals). **HRMS** (ESI) m/z: $[M+H]^+$ Calcd for $C_{24}H_{33}F_3NO^+$ 408.2514; Found 408.2518.

**4-(benzyloxy)-*N*-(4-methyl-2-(trifluoromethyl)hexyl)aniline (68)**

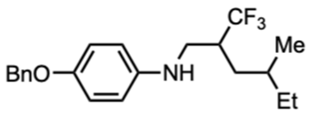

Using the General Procedure C (Variation 1), the title compound was obtained as a yellow viscous oil by preparative TLC using petroleum ether/EtOAc (15:1) as an eluent; $R_f$= 0.5 (petroleum ether/EtOAc = 10:1); 25.3 mg, 46% yield (d.r. = 1.2:1). **$^1$H NMR** (400 MHz, $CDCl_3$) $\delta$ 7.46 – 7.27 (m, 5H), 6.90 – 6.85 (m, 2H), 6.61 – 6.57 (m, 2H), 5.02 – 5.01 (m, 2H), 3.55 (brs, 1H), 3.40 – 2.85 (m, 2H), 2.48 – 2.43 (m, 1H), 1.71 – 1.32 (m, 4H), 1.23 – 1.10 (m, 1H), 0.98 – 0.87 (m, 6H). **$^{13}$C NMR** (201 MHz, $CDCl_3$) $\delta$ 151.8, 141.9, 141.8, 137.6, 128.7, 128.5 (q, $^1J_{CF}$ = 280.3 Hz), 128.5 (q, $^1J_{CF}$ = 280.3 Hz), 128.0, 127.6, 116.4, 114.4, 114.4, 71.0, 44.1, 43.6, 40.3 (q, $^2J_{CF}$ = 23.8 Hz), 40.2 (q, $^2J_{CF}$ = 23.7 Hz), 33.9, 33.3, 32.1, 31.9, 29.9, 29.1, 19.5, 18.9, 11.4, 11.1. (All $^{13}$C NMR signals were listed without classifying to the corresponding diastereomers due to the complexity). **$^{19}$F NMR** (376 MHz, $CDCl_3$) $\delta$ -69.43, -69.64.

Diastereomeric ratio (d.r.) = 1.2:1 (according to the ratio of integration of the two $^{19}F$ NMR signals). **HRMS** (ESI) m/z: $[M+H]^+$ Calcd for $C_{21}H_{27}F_3NO^+$ 366.2045; Found 366.2053.

**4-(benzyloxy)-*N*-(4-ethyl-2-(trifluoromethyl)hexyl)aniline (69)**

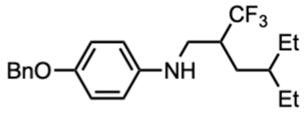


Using the General Procedure C (Variation 1), the title compound was obtained as a yellow viscous oil by preparative TLC using petroleum ether/EtOAc (15:1) as an eluent; $R_f$= 0.6 (petroleum ether/EtOAc = 10:1); 28.9 mg, 51% yield. **$^1$H NMR** (400 MHz, $CDCl_3$) $\delta$ 7.45 – 7.30 (m, 5H), 6.90 – 6.84 (m, 2H), 6.61 – 6.57 (m, 2H), 5.01 (s, 2H), 3.59 (brs, 1H), 3.38 – 2.98 (m, 2H), 2.40 – 2.35 (m, 1H), 1.59 – 1.27 (m, 7H), 0.95 – 0.84 (m, 6H). **$^{13}$C NMR** (201 MHz, $CDCl_3$) $\delta$ 151.8, 141.8, 137.6, 128.7, 128.6 (q, $^1J_{CF}$ = 280.5 Hz), 128.0, 127.6, 116.4, 114.4, 71.0, 43.8, 40.3 (q, $^2J_{CF}$ = 23.8 Hz), 37.8, 30.1, 25.6, 25.0, 10.8, 10.4. **$^{19}$F NMR** (376 MHz, $CDCl_3$) $\delta$ -69.56 (s, 3F). **HRMS** (ESI) m/z: $[M+H]^+$ Calcd for $C_{22}H_{29}F_3NO^+$ 380.2201; Found 380.2199.

**4-(benzyloxy)-*N*-(2-(cyclopropylmethyl)-3,3,3-trifluoropropyl)aniline (70)**

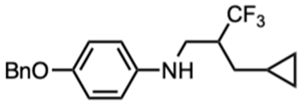


Using the General Procedure C (Variation 1), the title compound was obtained as a yellow viscous oil by preparative TLC using petroleum ether/EtOAc (15:1) as an eluent; $R_f$= 0.6 (petroleum ether/EtOAc = 10:1); 24.1 mg, 46% yield. **$^1$H NMR** (400 MHz, $CDCl_3$) $\delta$ 7.45 – 7.32 (m, 5H), 6.89 – 6.86 (m, 2H), 6.61 – 6.58 (m, 2H), 5.01 (s, 2H), 3.64 (brs, 1H), 3.48 – 3.34 (m, 2H), 2.60 – 2.44 (m, 1H), 1.62 – 1.42 (m, 2H), 0.89 – 0.81 (m, 1H), 0.57 – 0.49 (m, 2H), 0.16 – 0.08 (m, 2H). **$^{13}$C NMR** (101 MHz, $CDCl_3$) $\delta$ 151.7, 141.8, 137.6, 128.7, 128.2 (q, $^1J_{CF}$ = 280.4 Hz), 128.0, 127.6, 116.3, 114.3, 70.9, 43.1 (q, $^2J_{CF}$ = 23.3 Hz), 43.0, 31.6, 8.7, 5.6, 4.8. **$^{19}$F NMR** (376 MHz, $CDCl_3$) $\delta$ -69.26 (s, 3F). **HRMS** (ESI) m/z: $[M+H]^+$ Calcd for $C_{20}H_{23}F_3NO^+$ 350.1732; Found 350.1739.

**4-(benzyloxy)-*N*-(2-(cyclopentylmethyl)-3,3,3-trifluoropropyl)aniline (71)**

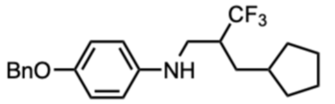

Using the General Procedure C (Variation 1), the title compound was obtained as a yellow viscous oil by preparative TLC using petroleum ether/EtOAc (15:1) as an eluent; $R_f$= 0.6 (petroleum ether/EtOAc = 10:1); 17.6 mg, 31% yield. **$^1$H NMR** (400 MHz, $CDCl_3$) $\delta$ 7.47 – 7.28 (m, 5H), 6.91 – 6.84 (m, 2H), 6.62 – 6.56 (m, 2H), 5.01 (s, 2H), 3.53 (brs, 1H), 3.40 – 3.23 (m, 2H), 2.44 – 2.38 (m, 1H), 2.01 – 1.93 (m, 1H), 1.84 – 1.52 (m, 9H), 1.14 – 1.06 (m, 1H). **$^{13}$C NMR** (201 MHz, $CDCl_3$) $\delta$ 151.8, 141.9, 137.6, 128.7, 128.4 (q, $^1J_{CF}$ = 280.6 Hz), 128.0, 127.6, 116.4, 114.4, 71.0, 43.6, 41.7 (q, $^2J_{CF}$ = 23.8 Hz), 37.5, 33.1, 32.8, 32.5, 25.2, 25.2. **$^{19}$F NMR** (376 MHz, $CDCl_3$) $\delta$ -69.52 (s, 3F). **HRMS** (ESI) m/z: $[M+H]^+$ Calcd for $C_{22}H_{27}F_3NO^+$ 378.2045; Found 378.2051.

**4-(benzyloxy)-*N*-(2-(cyclohexylmethyl)-3,3,3-trifluoropropyl)aniline (72)**

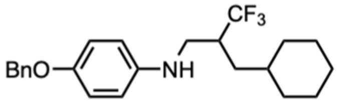

Using the General Procedure C (Variation 1), the title compound was obtained as a yellow viscous oil by preparative TLC using petroleum ether/EtOAc (15:1) as an eluent; $R_f$= 0.6 (petroleum ether/EtOAc = 10:1); 33.8 mg, 57% yield. **$^1$H NMR** (400 MHz, $CDCl_3$) $\delta$ 7.45 – 7.30 (m, 5H), 6.90 – 6.85 (m, 2H), 6.61 – 6.57 (m, 2H), 5.00 (s, 2H), 3.58 (brs, 1H), 3.35 – 3.18 (m, 2H), 2.40 – 2.35 (m, 1H), 1.75 – 1.66 (m, 3H), 1.55 – 1.12 (m, 8H), 0.95 – 0.82 (m, 2H). **$^{13}$C NMR** (201 MHz, $CDCl_3$) $\delta$ 151.8, 141.8, 137.6, 128.7, 128.5 (q, $^1J_{CF}$ = 280.5 Hz), 128.0, 127.6, 116.4, 114.5, 71.0, 43.9, 39.7 (q, $^2J_{CF}$ = 23.9 Hz), 35.1, 34.2, 33.8, 33.0, 26.5, 26.3, 26.2. **$^{19}$F NMR** (376 MHz, $CDCl_3$) $\delta$ -69.59 (s, 3F). **HRMS** (ESI) m/z: $[M+H]^+$ Calcd for $C_{23}H_{29}F_3NO^+$ 392.2201; Found 392.2207.

**4-(benzyloxy)-*N*-(3,3,3-trifluoro-2-((4-isopropylcyclohexyl)methyl)propyl)aniline (73)**

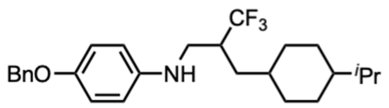

Using the General Procedure C (Variation 1), the title compound was obtained as a yellow viscous oil by preparative TLC using petroleum ether/EtOAc (15 1) as an eluent; $R_f$= 0.6 (petroleum ether/EtOAc = 10:1); 29.3 mg, 45% yield (d.r. = 1:1. **$^1$H NMR** (400 MHz, $CDCl_3$) $\delta$ 7.44 – 7.30 (m, 5H), 6.88 – 6.86 (m, 2H), 6.59 – 6.57 (m, 2H), 5.00 (s, 2H), 3.61 (brs, 1H), 3.36 – 3.18 (m, 2H), 2.45 – 2.38 (m, 1H), 1.79 – 1.66 (m, 3H), 1.53 – 1.31 (m, 7H), 1.09 – 0.90 (m, 3H), 0.87 – 0.85 (m, 6H). **$^{13}$C NMR** (201 MHz, $CDCl_3$) $\delta$ 151.8, 151.7, 141.8, 137.6, 128.7, 128.6 (q, $^1J_{CF}$ = 280.8 Hz), 128.5 (q, $^1J_{CF}$ = 280.5 Hz), 128.0, 127.6, 116.4, 114.5, 114.4, 71.0, 44.2, 43.9, 43.7, 43.0, 40.3 (q, $^2J_{CF}$ = 24.1 Hz), 39.8 (q, $^2J_{CF}$ = 23.8 Hz), 35.3, 34.2, 33.9, 33.2, 33.0, 31.9, 30.0, 29.6, 29.5, 28.7, 25.6, 25.3, 20.4, 20.3, 20.0, 19.9. (All $^{13}$C NMR signals were listed without classifying to the corresponding diastereomers due to the complexity). **$^{19}$F NMR** (376 MHz, $CDCl_3$) $\delta$ -69.57, -69.58. Diastereomeric ratio (d.r.) = 1:1 (according to the ratio of integration of the two $^{19}$F NMR signals). **HRMS** (ESI) m/z: $[M+H]^+$ Calcd for $C_{26}H_{35}F_3NO^+$ 434.2671; Found 434.2676.

**4-(benzyloxy)-*N*-(2-(cycloheptylmethyl)-3,3,3-trifluoropropyl)aniline (74)**

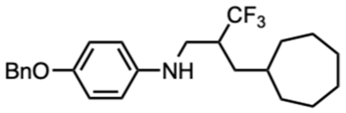

Using the General Procedure C (Variation 1), the title compound was obtained as a yellow viscous oil by preparative TLC using petroleum ether/EtOAc (15:1) as an eluent; $R_f$= 0.6 (petroleum ether/EtOAc = 10:1); 19.5 mg, 32% yield. **$^1$H NMR** (400 MHz, $CDCl_3$) $\delta$ 7.44 – 7.30 (m, 5H), 6.91 – 6.86 (m, 2H), 6.59 – 6.57 (m, 2H), 5.00 (s, 2H), 3.60 (brs, 1H), 3.36 – 3.19 (m, 2H), 2.45 – 2.37 (m, 1H), 1.71 – 1.32 (m, 13H), 1.21 – 1.11 (m, 2H). **$^{13}$C NMR** (201 MHz, $CDCl_3$) $\delta$ 151.8, 141.8, 137.6, 128.6, 128.5 (q, $^1J_{CF}$ = 280.6 Hz), 127.9, 127.6, 116.4, 114.4, 70.9, 43.8, 40.3 (q, $^2J_{CF}$ = 23.7 Hz), 36.6, 35.2, 34.6, 33.9, 28.5, 28.4, 26.3, 26.2. **$^{19}$F NMR** (376 MHz, $CDCl_3$) $\delta$ -69.53 (s, 3F). **HRMS** (ESI) m/z: $[M+H]^+$ Calcd for $C_{24}H_{31}F_3NO^+$ 406.2358; Found 406.2360.

**4-(benzyloxy)-*N*-(2-((2-bicyclo[2.2.1]heptan-2-yl)methyl)-3,3,3-trifluoropropyl)-aniline (75)**

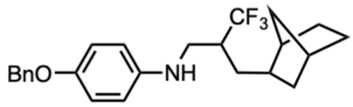


Using the General Procedure C (Variation 1), the title compound was obtained as a yellow viscous oil by preparative TLC using petroleum ether/EtOAc (15:1) as an eluent; $R_f$= 0.6 (petroleum ether/EtOAc = 10:1); 23.7 mg, 39% yield (d.r. = 1:1). **$^1$H NMR** (400 MHz, $CDCl_3$) $\delta$ 7.45 – 7.29 (m, 5H), 6.89 – 6.86 (m, 2H), 6.60 – 6.56 (m, 2H), 5.01 (s, 2H), 3.60 (brs, 1H), 3.37 – 3.22 (m, 2H), 2.40 – 2.35 (m, 1H), 2.23 – 2.21 (m, 1H), 1.94 – 1.90 (m, 1H), 1.55 – 1.38 (m, 5H), 1.32 – 1.25 (m, 2H), 1.20 – 1.09 (m, 3H), 1.02 – 0.95 (m, 1H). **$^{13}$C NMR** (201 MHz, $CDCl_3$) $\delta$ 151.8, 141.8, 141.8, 137.6, 128.7, 128.5 (q, $^1J_{CF}$ = 280.6 Hz), 128.4 (q, $^1J_{CF}$ = 280.5 Hz), 128.0, 127.6, 116.4, 114.4, 114.4, 71.0, 43.6, 43.4, 41.6, 41.0 (q, $^2J_{CF}$ = 23.7 Hz), 40.6, 40.4 (q, $^2J_{CF}$ = 24.0 Hz), 39.5, 39.4, 38.4, 37.9, 36.8, 36.7, 35.3, 35.2, 33.3, 33.2, 30.2, 30.1, 28.7, 28.7. (All $^{13}$C NMR signals were listed without classifying to the corresponding diastereomers due to the complexity). **$^{19}$F NMR** (376 MHz, $CDCl_3$) $\delta$ -69.34, -69.46. Diastereomeric ratio (d.r.) = 1:1 (according to the ratio of integration of the two $^{19}$F NMR signals). **HRMS** (ESI) m/z: $[M+H]^+$ Calcd for $C_{24}H_{29}F_3NO^+$ 404.2201; Found 404.2204.

***N*-(4-(adamantan-1-yl)-2-(trifluoromethyl)butyl)-4-(benzyloxy)aniline (76)**

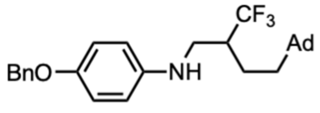


Using the General Procedure C (Variation 1), the title compound was obtained as a yellow viscous oil by preparative TLC using petroleum ether/EtOAc (15:1) as an eluent; $R_f$= 0.6 (petroleum ether/EtOAc = 10:1); 24.8 mg, 36% yield. **$^1$H NMR** (400 MHz, $CDCl_3$) $\delta$ 7.44 – 7.30 (m, 5H), 6.88 – 6.85 (m, 2H), 6.60 – 6.55 (m, 2H), 5.00 (s, 2H), 3.55 (brs, 1H), 3.37 – 3.21 (m, 2H), 2.27 – 2.17 (m, 1H), 2.01 – 1.95 (m, 3H), 1.72 – 1.59 (m, 7H), 1.50 – 1.41 (m, 7H), 1.20 – 1.12 (m, 2H). **$^{13}$C NMR** (201 MHz, $CDCl_3$) $\delta$ 151.8, 141.9, 137.7, 128.7, 128.4 (q, $^1J_{CF}$ = 280.7 Hz), 128.0, 127.6, 116.4, 114.5, 71.0, 43.2, 43.2 (q, $^2J_{CF}$ = 23.6 Hz), 42.3, 41.6, 37.3, 32.3, 28.8, 19.5.

**$^{19}$F NMR** (376 MHz, $CDCl_3$) $\delta$ -69.09 (s, 3F). **HRMS** (ESI) m/z: $[M+H]^+$ Calcd for $C_{28}H_{35}F_3NO^+$ 458.2671; Found 458.2676.

**4-(benzyloxy)-*N*-(5,5-dimethyl-2-(trifluoromethyl)hexyl)aniline (77)**

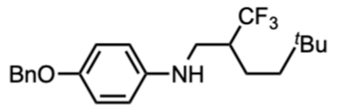


Using the General Procedure C (Variation 1), the title compound was obtained as a yellow viscous oil by preparative TLC using petroleum ether/EtOAc (15:1) as an eluent; $R_f$= 0.6 (petroleum ether/EtOAc = 10:1); 17.7 mg, 31% yield. **$^1$H NMR** (400 MHz, $CDCl_3$) $\delta$ 7.44 – 7.29 (m, 5H), 6.88 – 6.86 (m, 2H), 6.60 – 6.54 (m, 2H), 5.00 (s, 2H), 3.55 (brs, 1H), 3.38 – 3.22 (m, 2H), 2.31 – 2.24 (m, 1H), 1.69 – 1.60 (m, 1H), 1.53 – 1.43 (m, 1H), 1.34 – 1.27 (m, 2H), 0.90 (s, 9H). **$^{13}$C NMR** (201 MHz, $CDCl_3$) $\delta$ 151.8, 141.9, 137.7, 129.8 (q, $^1J_{CF}$ = 280.8 Hz), 128.7, 128.0, 127.6, 116.4, 114.5, 71.0, 43.2, 43.2 (q, $^2J_{CF}$ = 23.9 Hz), 41.1, 30.5, 29.3, 21.6. **$^{19}$F NMR** (376 MHz, $CDCl_3$) $\delta$ -69.11 (s, 3F). **HRMS** (ESI) m/z: $[M+H]^+$ Calcd for $C_{22}H_{29}F_3NO^+$ 380.2201; Found 380.2206.

**4-(benzyloxy)-*N*-(4-ethoxy-2-(trifluoromethyl)butyl)aniline (78)**

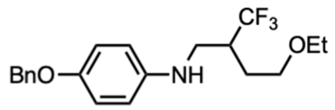


Using the General Procedure C (Variation 1, 2), the title compound was obtained as a yellow viscous oil by preparative TLC using petroleum ether/EtOAc (15:1) as an eluent; $R_f$= 0.6 (petroleum ether/EtOAc = 10:1); 14.9 mg, 27% yield. **$^1$H NMR** (800 MHz, $CDCl_3$) $\delta$ 7.43 – 7.30 (m, 5H), 6.87 – 6.86 (m, 2H), 6.59 – 6.57 (m, 2H), 5.00 (s, 2H), 3.89 (brs, 1H), 3.64 – 3.40 (m, 5H), 3.26 – 3.23 (m, 1H), 2.62 – 2.56 (m, 1H), 2.01 – 1.97 (m, 1H), 1.79 – 1.75 (m, 1H), 1.22 (t, $J$ = 7.0 Hz, 3H). **$^{13}$C NMR** (201 MHz, $CDCl_3$) $\delta$ 151.6, 142.0, 137.7, 128.7, 128.2 (q, $^1J_{CF}$ = 280.4 Hz), 127.9, 127.6, 116.4, 114.2, 71.0, 67.8, 66.6, 43.2, 40.2 (q, $^2J_{CF}$ = 24.5 Hz), 27.0, 15.4. **$^{19}$F NMR** (376 MHz, $CDCl_3$) $\delta$ -69.92 (s, 3F). **HRMS** (ESI) m/z: $[M+H]^+$ Calcd for $C_{20}H_{25}F_3NO_2^+$ 368.1837; Found 368.1841.

**4-(benzyloxy)-*N*-(4-cyclopentyl-2-(trifluoromethyl)butyl)aniline (79)**

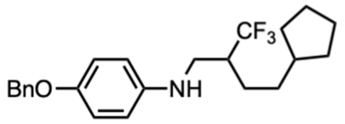


Using the General Procedure C (Variation 1), the title compound was obtained as a yellow viscous oil by preparative TLC using petroleum ether/EtOAc (15:1) as an eluent; R*f*= 0.6 (petroleum ether/EtOAc = 10:1); 21.7 mg, 37% yield. **$^{1}$H NMR** (400 MHz, $CDCl_3$) $\delta$ 7.44 – 7.30 (m, 5H), 6.89 – 6.84 (m, 2H), 6.60 – 6.55 (m, 2H), 5.00 (s, 2H), 3.62 (brs, 1H), 3.38 – 3.22 (m, 2H), 2.34 – 2.22 (m, 1H), 1.78 – 1.40 (m, 11H), 1.10 – 1.06 (m, 2H). **$^{13}$C NMR** (201 MHz, $CDCl_3$) $\delta$ 151.8, 141.8, 137.6, 128.7, 128.4 (q, $^{1}J_{CF}$ = 280.8 Hz), 128.0, 127.6, 116.4, 114.4, 71.0, 43.3, 42.6 (q, $^{2}J_{CF}$ = 23.9 Hz), 40.2, 33.4, 32.7, 32.6, 25.3. **$^{19}$F NMR** (376 MHz, $CDCl_3$) $\delta$ -69.17 (s, 3F). **HRMS** (ESI) m/z: $[M+H]^+$ Calcd for $C_{23}H_{29}F_3NO^+$ 392.2201; Found 392.2207.

**4-(benzyloxy)-*N*-(6-cyclohexyl-2-(trifluoromethyl)hexyl)aniline (80)**

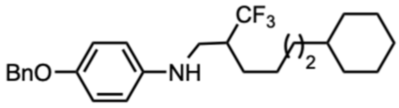


Using the General Procedure C (Variation 1), the title compound was obtained as a yellow viscous oil by preparative TLC using petroleum ether/EtOAc (15:1) as an eluent; R*f*= 0.5 (petroleum ether/EtOAc = 10:1); 17.6 mg, 27% yield. **$^{1}$H NMR** (800 MHz, $CDCl_3$) $\delta$ 7.42 – 7.41 (m, 2H), 7.38 – 7.36 (m, 2H), 7.32 – 7.30 (m, 1H), 6.87 – 6.85 (m, 2H), 6.58 – 6.56 (m, 2H), 4.99 (s, 2H), 3.56 (brs, 1H), 3.35 – 3.33 (m, 1H), 3.24 – 3.22 (m, 1H), 2.37 – 2.32 (m, 1H), 1.69 – 1.62 (m, 6H), 1.53 – 1.48 (m, 1H), 1.43 – 1.35 (m, 2H), 1.31 – 1.27 (m, 2H), 1.21 – 1.12 (m, 6H), 0.85 – 0.82 (m, 2H). **$^{13}$C NMR** (201 MHz, $CDCl_3$) $\delta$ 151.8, 141.8, 137.6, 128.7, 128.4 (q, $^{1}J_{CF}$ = 281.2 Hz), 128.0, 127.6, 116.4, 114.4, 70.9, 43.2 (q, $^{3}J_{CF}$ = 2.8 Hz), 42.4 (q, $^{2}J_{CF}$ = 24.0 Hz), 37.7, 37.3, 33.5 (q, $^{3}J_{CF}$ = 4.5 Hz), 27.4, 27.0, 26.8, 26.5. **$^{19}$F NMR** (376 MHz, $CDCl_3$) $\delta$ -69.23 (s, 3F). **HRMS** (ESI) m/z: $[M+H]^+$ Calcd for $C_{26}H_{35}F_3NO^+$ 434.2671; Found 434.2672.

***N*-(2-((adamantan-1-yl)methyl)-1,1,1-trifluoropentan-3-yl)-4-(benzyloxy)aniline (81)**

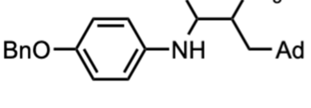

Using the General Procedure C, the title compound was obtained as a yellow viscous oil by preparative TLC using petroleum ether/EtOAc (15:1) as an eluent; $R_f = 0.6$ (petroleum ether/EtOAc = 10:1); 21.9 mg, 31% yield (d.r. = 1.5:1). **$^1$H NMR** (400 MHz, $CDCl_3$) $\delta$ 7.44 – 7.30 (m, 5H), 6.88 – 6.83 (m, 2H), 6.61 – 6.51 (m, 2H), 5.00 (s, 2H), 3.45 – 3.23 (m, 2H), 2.52 – 2.47 (m, 1H), 1.97 – 1.92 (m, 3H), 1.71 – 1.66 (m, 3H), 1.60 – 1.57 (m, 3H), 1.47 – 1.34 (m, 7H), 1.16 – 1.12 (m, 1H), 1.05 – 0.98 (m, 3H), 0.92 – 0.84 (m, 2H). **$^{13}$C NMR** (201 MHz, $CDCl_3$) $\delta$ 151.7, 151.2, 142.1, 142.0, 137.7, 137.7, 129.0 (q, $^1J_{CF}$ = 281.6 Hz), 128.6, 127.9, 127.9, 127.7, 127.6, 116.5, 116.3, 115.8, 114.0, 113.3, 71.0, 70.9, 59.6, 56.7, 42.5, 42.4, 40.5, 39.9 (q, $^2J_{CF}$ = 22.2 Hz), 39.3, 38.8 (q, $^2J_{CF}$ = 22.3 Hz), 37.0, 36.9, 32.8, 32.5, 28.6, 28.6, 25.9, 24.9, 12.6, 11.9. (All $^{13}$C NMR signals were listed without classifying to the corresponding diastereomers due to the complexity). **$^{19}$F NMR** (376 MHz, $CDCl_3$) $\delta$ -64.46, -64.79. Diastereomeric ratio (d.r.) = 1.5:1 (according to the ratio of integration of the two $^{19}$F NMR signals). **HRMS** (ESI) m/z: $[M+H]^+$ Calcd for $C_{29}H_{37}F_3NO^+$ 472.2827; Found 472.2833.

***N*-(2-((adamantan-1-yl)methyl)-1,1,1-trifluoroheptan-3-yl)-4-(benzyloxy)aniline (82)**

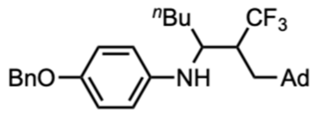


Using the General Procedure C, the title compound was obtained as a yellow viscous oil by preparative TLC using petroleum ether/EtOAc (15:1) as an eluent; $R_f =$ 0.6 (petroleum ether/EtOAc = 10:1); 38.0 mg, 51% yield (d.r. = 1.5:1). **$^1$H NMR** (400 MHz, $CDCl_3$) $\delta$ 7.44 – 7.29 (m, 5H), 6.86 – 6.79 (m, 2H), 6.61 – 6.49 (m, 2H), 5.00 – 4.97 (m, 2H), 3.57 – 3.31 (m, 1H), 2.53 – 2.45 (m, 1H), 1.97 – 1.91 (m, 3H), 1.73 – 1.54 (m, 7H), 1.47 – 1.28 (m, 12H), 1.13 – 1.08 (m, 1H), 0.92 – 0.86 (m, 3H). (Due to the broadening effect and intermolecular hydrogen bonding, one of the proton signals of NH could not be observed.). **$^{13}$C NMR** (201 MHz, $CDCl_3$) $\delta$ 151.7, 151.1, 141.9, 141.8, 137.7, 137.7, 129.0 (q, $^1J_{CF}$ = 281.6 Hz), 128.6, 128.6, 127.9, 127.9, 127.7, 127.6, 116.5, 116.3, 115.8, 113.9, 71.0, 70.9, 57.8, 54.5, 42.5, 42.4, 40.6, 39.8 (q, $^2J_{CF}$

= 22.1 Hz), 39.3, 39.1 (q, $^2J_{CF}$ = 22.9 Hz), 38.6, 37.4, 37.0, 32.9, 32.5, 31.5, 29.9, 29.9, 28.6, 28.6, 22.8, 22.7, 14.2, 14.1. (All $^{13}$C NMR signals were listed without classifying to the corresponding diastereomers due to the complexity). **$^{19}$F NMR** (376 MHz, $CDCl_3$) $\delta$ -64.28, -64.93. Diastereomeric ratio (d.r.) = 1.5:1 (according to the ratio of integration of the two $^{19}$F NMR signals). **HRMS** (ESI) m/z: $[M+H]^+$ Calcd for $C_{31}H_{41}F_3NO^+$ 500.3140; Found 500.3146.

***N*-(3-((adamantan-1-yl)methyl)-4,4,4-trifluoro-1-(2-methoxyethoxy)butan-2-yl)-4-(benzyloxy)aniline (83)**

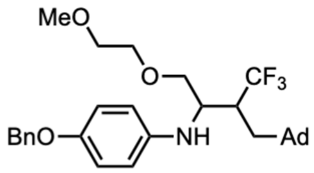


Using the General Procedure C, the title compound was obtained as a brown amorphous solid by preparative TLC using petroleum ether/EtOAc (15:1) as an eluent; R*f*= 0.6 (petroleum ether/EtOAc = 10:1); 28.6 mg, 36% yield (d.r. = 1.5:1). **$^1$H NMR** (400 MHz, $CDCl_3$) $\delta$ 7.44 – 7.29 (m, 5H), 6.86 – 6.80 (m, 2H), 6.64 – 6.60 (m, 2H), 4.99 – 4.98 (m, 2H), 3.67 – 3.45 (m, 7H), 3.37 – 3.34 (m, 3H), 2.66 – 2.56 (m, 1H), 1.99 – 1.95 (m, 3H), 1.75 – 1.58 (m, 9H), 1.49 – 1.48 (m, 4H), 1.38 – 1.36 (m, 1H). (Due to the broadening effect and intermolecular hydrogen bonding, one of the proton signals of NH could not be observed.). Diastereomeric ratio (d.r.) = 1.5:1 (according to the ratio of integration of the two $^1$H NMR signals). **$^{13}$C NMR** (201 MHz, $CDCl_3$) $\delta$ 151.9, 151.0, 144.1, 141.6, 137.9, 137.7, 128.7 (q, $^1J_{CF}$ = 281.1 Hz), 128.6, 128.6, 127.9, 127.9, 127.6, 127.6, 116.2, 116.2, 115.7, 114.9, 72.1, 72.0, 71.0, 70.9, 70.8, 70.7, 70.6, 70.5, 59.2, 55.8, 42.2, 39.6, 39.0, 38.4 (q, $^2J_{CF}$ = 23.6 Hz), 37.3, 37.0, 36.9, 32.7, 32.7, 32.1, 29.8, 29.8, 29.5, 28.6. (All $^{13}$C NMR signals were listed without classifying to the corresponding diastereomers due to the complexity). **$^{19}$F NMR** (376 MHz, $CDCl_3$) $\delta$ -66.14 (s, 3F). **HRMS** (ESI) m/z: $[M+H]^+$ Calcd for $C_{31}H_{41}F_3NO_3^+$ 532.3039; Found 532.3045.

***N*-(2-((adamantan-1-yl)methyl)-3,3,4,4,5,5,6,6,6-nonafluorohexyl)-4-(benzyloxy)-aniline (84)**

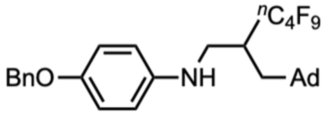

Using the General Procedure C (Variation 3), the title compound was obtained as a yellow viscous oil by preparative TLC using petroleum ether/EtOAc (15:1) as an eluent; $R_f$= 0.6 (petroleum ether/EtOAc = 10:1); 50.8 mg, 57% yield. **$^1$H NMR** (400 MHz, $CDCl_3$) $\delta$ 7.47 – 7.32 (m, 5H), 6.92 – 6.88 (m, 2H), 6.64 – 6.58 (m, 2H), 5.03 (s, 2H), 3.56 (brs, 1H), 3.43 – 3.23 (m, 2H), 2.68 – 2.58 (m, 1H), 2.01 – 1.99 (m, 3H), 1.76 – 1.73 (m, 3H), 1.67 – 1.64 (m, 3H), 1.61 – 1.49 (m, 7H), 1.37 – 1.30 (m, 1H). **$^{13}$C NMR** (101 MHz, $CDCl_3$) $\delta$ 151.8, 142.3, 137.7, 128.6, 128.0, 127.6, 116.3, 114.6, 71.0, 45.5, 42.4, 39.7, 36.9, 36.5 (t, $^2J_{CF}$ = 19.8 Hz), 32.7, 28.6. **$^{19}$F NMR** (376 MHz, $CDCl_3$) $\delta$ -80.80 – -80.87 (m, 3F), -108.64 – -111.08 (m, 1F), -112.54 – -114.76 (m, 1F), -118.94 – -122.01 (m, 2F), -125.78 – -125.96 (m, 2F). **HRMS** (ESI) m/z: $[M+H]^+$ Calcd for $C_{30}H_{33}F_9NO^+$ 594.2418; Found 594.2427.

**_N_-(2-((adamantan-1-yl)methyl)-3,3,4,4,5,5,6,6,7,7,8,8,8-tridecafluorooctyl)-4-(ben -zyloxy)aniline (85)**

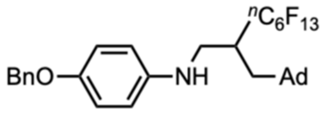

Using the General Procedure C (Variation 3), the title compound was obtained as a yellow viscous oil by preparative TLC using petroleum ether/EtOAc (15:1) as an eluent; $R_f$= 0.5 (petroleum ether/EtOAc = 10:1); 46.0 mg, 49% yield. **$^1$H NMR** (400 MHz, $CDCl_3$) $\delta$ 7.44 – 7.29 (m, 5H), 6.89 – 6.85 (m, 2H), 6.61 – 6.55 (m, 2H), 5.00 (s, 2H), 3.39 – 3.20 (m, 2H), 2.60 – 2.51 (m, 1H), 1.99 – 1.96 (m, 3H), 1.73 – 1.70 (m, 3H), 1.64 – 1.60 (m, 3H), 1.57 – 1.46 (m, 7H), 1.34 – 1.30 (m, 1H). (Due to the broadening effect and intermolecular hydrogen bonding, one of the proton signals of NH could not be observed.). **$^{13}$C NMR** (101 MHz, $CDCl_3$) $\delta$ 151.9, 142.3, 137.7, 128.7, 128.0, 127.6, 116.4, 114.6, 71.0, 45.6, 42.5, 39.7, 37.0, 36.6 (t, $^2J_{CF}$ = 20.2 Hz), 32.7, 28.6. **$^{19}$F NMR** (376 MHz, $CDCl_3$) $\delta$ -80.12 – -81.32 (m, 3F), -108.27 – -110.83 (m, 1F), -112.30 – -114.56 (m, 1F), -118.89 – -120.91 (m, 2F), -121.08 – -122.09 (m, 2F), -122.61 – -122.69 (m, 2F), -124.80 – -126.67 (m, 2F). **HRMS** (ESI) m/z: $[M+H]^+$ Calcd for $C_{32}H_{33}F_{13}NO^+$ 694.2355; Found 694.2362.

***N*-(2-((adamantan-1-yl)methyl)-2,3,3,3-tetrafluoropropyl)-4-(benzyloxy)aniline (86)**

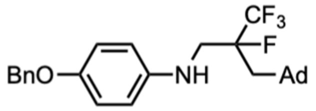

Using the General Procedure C (Variation 4), the title compound was obtained as a yellow viscous oil by preparative TLC using petroleum ether/EtOAc (15:1) as an eluent; $R_f$= 0.6 (petroleum ether/EtOAc = 10:1); 32.7 mg, 47% yield. **$^1$H NMR** (600 MHz, $CDCl_3$) $\delta$ 7.45 – 7.31 (m, 5H), 6.90 – 6.87 (m, 2H), 6.64 – 6.61 (m, 2H), 5.02 (s, 2H), 3.58 – 3.50 (m, 3H), 1.98 – 1.97 (m, 3H), 1.80 – 1.65 (m, 14H). **$^{13}$C NMR** (201 MHz, $CDCl_3$) $\delta$ 152.0, 141.8, 137.6, 128.7, 128.0, 127.6, 124.1 (qd, $^1J_{CF}$ = 285.7, 29.6 Hz), 116.3, 114.6, 95.9 (dq, $^2J_{CF}$ = 191.0, 28.5 Hz), 70.9, 48.1 (q, $^3J_{CF}$ = 24.7 Hz), 43.3, 42.7 (q, $^3J_{CF}$ = 19.3 Hz), 36.9, 33.8, 28.8. **$^{19}$F NMR** (565 MHz, $CDCl_3$) $\delta$ -79.61 (d, $J$ = 4.8 Hz, 3F), -179.40 (q, $J$ = 11.3 Hz, 1F). **HRMS** (ESI) m/z: $[M+H]^+$ Calcd for $C_{27}H_{32}F_4NO^+$ 462.2420; Found 462.2422.

**Methyl 4-(3-((4-(benzyloxy)phenyl)amino)-2-(trifluoromethyl)butyl)bicyclo[2.2.2] octane-1-carboxylate (87)**

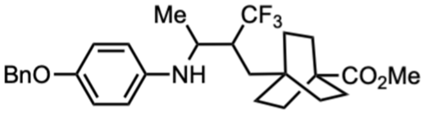

Using the General Procedure C, the title compound was obtained as a yellow viscous oil by preparative TLC using petroleum ether/EtOAc (15:1) as an eluent; $R_f$= 0.6 (petroleum ether/EtOAc = 10:1); 40.3 mg, 55% yield (d.r. = 1:1). **$^1$H NMR** (400 MHz, $CDCl_3$) $\delta$ 7.42 – 7.30 (m, 5H), 6.88 – 6.84 (m, 2H), 6.60 – 6.48 (m, 2H), 5.00 – 4.98 (m, 2H), 3.82 – 3.46 (m, 4H), 3.36 (brs, 1H), 2.52 – 2.43 (m, 1H), 1.79 – 1.70 (m, 6H), 1.47 – 1.39 (m, 4H), 1.36 – 1.29 (m, 3H), 1.24 – 1.18 (m, 4H). **$^{13}$C NMR** (201 MHz, $CDCl_3$) $\delta$ 178.4, 178.3, 152.1, 151.5, 140.9, 140.8, 137.6, 137.5, 128.8 (q, $^1J_{CF}$ = 281.5 Hz), 128.7, 128.6, 128.4 (q, $^1J_{CF}$ = 281.1 Hz), 128.0, 127.9, 127.7, 127.6, 116.5, 116.3, 116.2, 114.2, 70.9, 70.8, 52.8, 51.8, 51.7, 48.8, 42.0 (q, $^2J_{CF}$ = 22.8 Hz), 40.7 (q, $^2J_{CF}$ = 23.0 Hz), 38.9, 37.8, 34.8, 31.3, 30.9, 30.6, 30.5, 28.5, 28.5, 17.6, 16.3. (All $^{13}$C NMR signals were listed

without classifying to the corresponding diastereomers due to the complexity). **$^{19}$F NMR** (376 MHz, $CDCl_3$) $\delta$ -64.30, -65.46. Diastereomeric ratio (d.r.) = 1:1 (according to the ratio of integration of the two $^{19}$F NMR signals).**HRMS** (ESI) m/z: $[M+H]^+$ Calcd for $C_{28}H_{35}F_3NO_3^+$ 490.2569; Found 490.2575.

**4-(benzyloxy)-*N*-(4,4,4-trifluoro-3-((1-methylcyclohexyl)methyl)butan-2-yl)-aniline (88)**

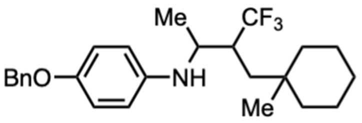


Using the General Procedure C, the title compound was obtained as a yellow viscous oil by preparative TLC using petroleum ether/EtOAc (15:1) as an eluent; $R_f$ = 0.6 (petroleum ether/EtOAc = 10:1); 39.7 mg, 63% yield (d.r. = 1:1). **$^1$H NMR** (400 MHz, $CDCl_3$) $\delta$ 7.45 – 7.31 (m, 5H), 6.88 – 6.85 (m, 2H), 6.62 – 6.51 (m, 2H), 5.01 – 5.00 (m, 2H), 3.85 – 3.55 (m, 1H), 3.43 (brs, 1H), 2.52 – 2.44 (m, 1H), 1.60 – 1.54 (m, 1H), 1.52 – 1.38 (m, 7H), 1.31 – 1.27 (m, 3H), 1.18 – 1.11 (m, 4H), 0.91 – 0.79 (m, 3H). **$^{13}$C NMR** (201 MHz, $CDCl_3$) $\delta$ 152.1, 151.4, 141.1, 141.0, 137.7, 137.6, 129.0 (q, $^1J_{CF}$ = 281.7 Hz), 128.7 (q, $^1J_{CF}$ = 281.1 Hz), 128.6, 128.0, 127.9, 127.7, 116.5, 116.3, 114.2, 71.0, 70.9, 53.1, 49.1, 41.9 (q, $^2J_{CF}$ = 22.3 Hz), 40.8 (q, $^2J_{CF}$ = 22.9 Hz), 38.1, 37.9, 37.8, 37.6, 33.5, 33.0, 26.4, 26.3, 22.2, 22.1, 22.0, 21.9, 17.9, 16.1. (All $^{13}$C NMR signals were listed without classifying to the corresponding diastereomers due to the complexity). **$^{19}$F NMR** (376 MHz, $CDCl_3$) $\delta$ -64.89, -65.00. Diastereomeric ratio (d.r.) = 1:1 (according to the ratio of integration of the two $^{19}$F NMR signals). **HRMS** (ESI) m/z: $[M+H]^+$ Calcd for $C_{25}H_{33}F_3NO^+$ 420.2514; Found 420.2519.

**4-(benzyloxy)-*N*-(4,4,4-trifluoro-3-((4-methyltetrahydro-2*H*-pyran-4-yl)methyl)-butan-2-yl)aniline (89)**

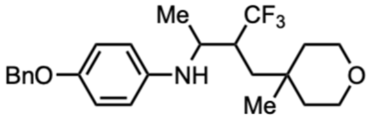


Using the General Procedure C, the title compound was obtained as a yellow viscous oil by preparative TLC using petroleum ether/EtOAc (15:1) as an

eluent; $R_f$ = 0.6 (petroleum ether/EtOAc = 10:1); 34.8 mg, 55% yield (d.r. = 1.2:1). **$^1$H NMR** (400 MHz, $CDCl_3$) $\delta$ 7.44 – 7.30 (m, 5H), 6.87 – 6.84 (m, 2H), 6.60 – 6.49 (m, 2H), 5.00 – 4.99 (m, 2H), 3.86 – 3.34 (m, 6H), 2.52 – 2.47 (m, 1H), 1.71 – 1.32 (m, 6H), 1.29 – 1.24 (m, 3H), 1.06 – 0.89 (m, 3H). **$^{13}$C NMR** (101 MHz, $CDCl_3$) $\delta$ 152.1, 151.4, 140.8, 140.6, 137.6, 137.5, 128.8 (q, $^1J_{CF}$ = 281.8 Hz), 128.6, 128.3 (q, $^1J_{CF}$ = 281.0 Hz), 128.0, 127.7, 127.6, 116.5, 116.3, 116.2, 116.1, 114.2, 114.1, 70.9, 70.8, 64.0, 63.9, 63.8, 63.7, 53.3, 48.7, 41.6 (q, $^2J_{CF}$ = 22.7 Hz), 40.1 (q, $^2J_{CF}$ = 23.0 Hz), 38.0, 37.8, 37.6, 37.2, 36.3, 31.4, 31.0, 30.7, 23.2, 23.0, 17.6, 16.4. (All $^{13}$C NMR signals were listed without classifying to the corresponding diastereomers due to the complexity). **$^{19}$F NMR** (376 MHz, $CDCl_3$) $\delta$ -63.97, -65.17. Diastereomeric ratio (d.r.) = 1.2:1 (according to the ratio of integration of the two $^{19}$F NMR signals). **HRMS** (ESI) m/z: $[M+H]^+$ Calcd for $C_{24}H_{31}F_3NO_2^+$ 422.2307; Found 422.2313.

***Tert*-butyl 4-(3-((4-(benzyloxy)phenyl)amino)-2-(trifluoromethyl)butyl)-4-methyl piperidine-1-carboxylate (90)**

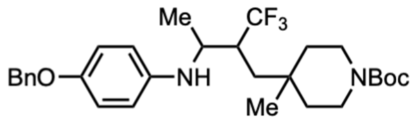


Using the General Procedure C (Variation 5), the title compound was obtained as a yellow viscous oil by preparative TLC using petroleum ether/EtOAc (5:1) as an eluent; $R_f$ = 0.2 (petroleum ether/EtOAc = 10:1); 39.0 mg, 50% yield (d.r. = 1.1:1). **$^1$H NMR** (400 MHz, $CDCl_3$) $\delta$ 7.44 – 7.29 (m, 5H), 6.88 – 6.84 (m, 2H), 6.60 – 6.49 (m, 2H), 5.00 – 4.99 (m, 2H), 3.83 – 3.53 (m, 4H), 3.14 – 3.01 (m, 2H), 2.53 – 2.38 (m, 1H), 1.54 – 1.43 (m, 11H), 1.33 – 1.23 (m, 7H), 0.96 – 0.82 (m, 3H). Diastereomeric ratio (d.r.) = 1.1:1 (according to the ratio of integration of the two $^1$H NMR signals). **$^{13}$C NMR** (201 MHz, $CDCl_3$) $\delta$ 155.0, 154.9, 152.2, 151.5, 140.8, 137.6, 137.5, 128.8 (q, $^1J_{CF}$ = 282.1 Hz), 128.3 (q, $^1J_{CF}$ = 281.4 Hz), 128.6, 128.0, 127.9, 127.7, 116.5, 116.4, 116.3, 114.1, 79.6, 79.5, 70.9, 70.8, 53.3, 41.8 (q, $^2J_{CF}$ = 22.4 Hz), 39.0, 32.2, 31.8, 28.6, 22.7, 22.6, 17.6, 16.4. (All $^{13}$C NMR signals were listed without classifying to the corresponding diastereomers due to the complexity). **$^{19}$F NMR** (376 MHz, $CDCl_3$) $\delta$

-63.99 (s, 3F). **HRMS** (ESI) m/z: $[M+Na]^+$ Calcd for $C_{29}H_{39}F_3N_2NaO_3^+$ 543.2810; Found 543.2817.

**4-(benzyloxy)-*N*-(4,4,4-trifluoro-3-((1-methylcyclobutyl)methyl)butan-2-yl)-aniline (91)**

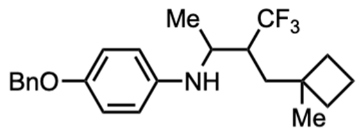


Using the General Procedure C, the title compound was obtained as a yellow viscous oil by preparative TLC using petroleum ether/EtOAc (15:1) as an eluent; $R_f$= 0.6 (petroleum ether/EtOAc = 10:1); 21.7 mg, 37% yield (d.r. = 1:1). **$^1$H NMR** (400 MHz, $CDCl_3$) $\delta$ 7.45 – 7.31 (m, 5H), 6.88 – 6.84 (m, 2H), 6.61 – 6.52 (m, 2H), 5.01 – 5.00 (m, 2H), 3.88 – 3.33 (m, 2H), 2.51 – 2.44 (m, 1H), 2.02 – 1.52 (m, 8H), 1.29 – 1.22 (m, 3H), 1.17 – 1.00 (m, 3H). **$^{13}$C NMR** (201 MHz, $CDCl_3$) $\delta$ 152.0, 151.5, 141.0, 140.7, 137.7, 137.6, 128.7 (q, $^1J_{CF}$ = 281.8 Hz), 128.6, 128.5, 128.3 (q, $^1J_{CF}$ = 281.4 Hz), 128.0, 127.9, 127.7, 116.5, 116.3, 116.2, 115.9, 114.5, 70.9, 70.8, 51.1, 48.0, 43.1 (q, $^2J_{CF}$ = 22.5 Hz), 41.6 (q, $^2J_{CF}$ = 23.2 Hz), 38.1, 37.9, 34.4, 34.3, 34.0, 33.7, 31.6, 31.5, 25.2, 25.1, 17.2, 16.2, 15.4, 15.0. (All $^{13}$C NMR signals were listed without classifying to the corresponding diastereomers due to the complexity). **$^{19}$F NMR** (376 MHz, $CDCl_3$) $\delta$ -64.23, -65.37. Diastereomeric ratio (d.r.) = 1:1 (according to the ratio of integration of the two $^{19}$F NMR signals). **HRMS** (ESI) m/z: $[M+H]^+$ Calcd for $C_{23}H_{29}F_3NO^+$ 392.2201; Found 392.2207.

**4-(benzyloxy)-*N*-(4,4,4-trifluoro-3-((1-methylcyclopropyl)methyl)butan-2-yl)-aniline (92)**

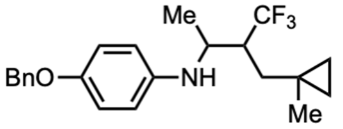


Using the General Procedure C, the title compound was obtained as a yellow viscous oil by preparative TLC using petroleum ether/EtOAc (15:1) as an eluent; $R_f$ = 0.6 (petroleum ether/EtOAc = 10:1); 32.8 mg, 58% yield (d.r. = 2:1). **$^1$H NMR** (800 MHz, $CDCl_3$) $\delta$ 7.44 – 7.31 (m, 5H), 6.89 – 6.86 (m, 2H), 6.62 – 6.55 (m, 2H), 5.01 – 5.00 (m, 2H), 3.99 – 3.86 (m, 1H), 3.42 (brs, 1H), 2.72 – 2.68 (m, 1H), 1.66 – 1.64 (m, 1H),

1.48 – 1.41 (m, 1H), 1.25 – 1.20 (m, 3H), 1.05 – 1.04 (m, 1H), 0.91 – 0.86 (m, 2H), 0.46 – 0.28 (m, 4H). **$^{13}$C NMR** (201 MHz, $CDCl_3$) $\delta$ 151.9, 151.6, 141.0, 140.7, 137.6, 137.6, 128.6, 128.6 (q, $^1J_{CF}$ = 282.2 Hz), 128.3 (q, $^1J_{CF}$ = 281.4 Hz), 127.9, 127.7, 116.5, 116.4, 115.6, 114.7, 70.9, 70.9, 49.6, 47.4, 43.8 (q, $^2J_{CF}$ = 22.3 Hz), 42.4 (q, $^2J_{CF}$ = 23.2 Hz), 35.8, 32.6, 22.5, 22.4, 17.0, 16.1, 14.0, 13.9, 13.8, 13.5, 13.3, 13.0. (All $^{13}$C NMR signals were listed without classifying to the corresponding diastereomers due to the complexity). **$^{19}$F NMR** (376 MHz, $CDCl_3$) $\delta$ -64.50, -65.52. Diastereomeric ratio (d.r.) = 2:1 (according to the ratio of integration of the two $^{19}$F NMR signals). **HRMS** (ESI) m/z: $[M+H]^+$ Calcd for $C_{22}H_{27}F_3NO^+$ 378.2045; Found 378.2052.

**4-(benzyloxy)-*N*-(5,5-dimethyl-3-(trifluoromethyl)hexan-2-yl)aniline (93)**

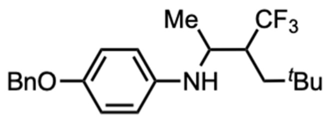


Using the General Procedure C, the title compound was obtained as a yellow viscous oil by preparative TLC using petroleum ether/EtOAc (15:1) as an eluent; $R_f$= 0.5 (petroleum ether/EtOAc = 10:1); 23.9 mg, 42% yield (d.r. = 1.5:1). **$^1$H NMR** (400 MHz, $CDCl_3$) $\delta$ 7.45 – 7.31 (m, 5H), 6.89 – 6.86 (m, 2H), 6.62 – 6.51 (m, 2H), 5.01 – 5.00 (m, 2H), 3.86 – 3.55 (m, 1H), 3.40 (brs, 1H), 2.50 – 2.39 (m, 1H), 1.60 – 1.44 (m, 2H), 1.28 – 1.24 (m, 3H), 0.95 – 0.86 (m, 9H). **$^{13}$C NMR** (101 MHz, $CDCl_3$) $\delta$ 152.0, 151.4, 141.0, 140.9, 137.7, 137.6, 128.9 (q, $^1J_{CF}$ = 282.8 Hz), 128.6, 128.5 (q, $^1J_{CF}$ = 281.0 Hz), 128.0, 127.9, 127.7, 116.5, 116.3, 116.2, 114.1, 70.9, 70.8, 53.0, 48.7, 42.8 (q, $^2J_{CF}$ = 22.6 Hz), 41.6 (q, $^2J_{CF}$ = 23.1 Hz), 40.0, 36.9, 31.1, 30.8, 29.7, 29.5, 17.7, 16.3. (All $^{13}$C NMR signals were listed without classifying to the corresponding diastereomers due to the complexity). **$^{19}$F NMR** (376 MHz, $CDCl_3$) $\delta$ -64.14, -65.37. Diastereomeric ratio (d.r.) = 1.5:1 (according to the ratio of integration of the two $^{19}$F NMR signals). **HRMS** (ESI) m/z: $[M+H]^+$ Calcd for $C_{22}H_{29}F_3NO^+$ 380.2201; Found 380.2207.

**4-(benzyloxy)-*N*-(5,5-dimethyl-3-(trifluoromethyl)heptan-2-yl)aniline (94)**

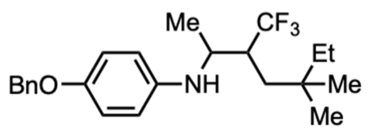

Using the General Procedure C, the title compound was obtained as a yellow viscous oil by preparative TLC using petroleum ether/EtOAc (15:1) as an eluent; $R_f$= 0.5 (petroleum ether/EtOAc = 10:1); 36.6 mg, 62% yield (d.r. = 1.4:1). **$^1$H NMR** (400 MHz, $CDCl_3$) $\delta$ 7.46 – 7.31 (m, 5H), 6.89 – 6.85 (m, 2H), 6.63 – 6.51 (m, 2H), 5.02 – 5.00 (m, 2H), 3.84 – 3.56 (m, 1H), 3.43 (brs, 1H), 2.51 – 2.42 (m, 1H), 1.55 – 1.47 (m, 1H), 1.29 – 1.19 (m, 6H), 0.89 – 0.76 (m, 9H). **$^{13}$C NMR** (201 MHz, $CDCl_3$) $\delta$ 152.1, 151.4, 141.0, 140.9, 137.7, 137.6, 129.0 (q, $^1J_{CF}$ = 281.6 Hz), 128.6, 128.5 (q, $^1J_{CF}$ = 281.1 Hz), 128.0, 127.9, 127.7, 116.5, 116.3, 116.2, 114.2, 70.9, 70.8, 53.0, 49.1, 42.5 (q, $^2J_{CF}$ = 22.4 Hz), 41.4 (q, $^2J_{CF}$ = 23.0 Hz), 38.0, 35.1, 34.9, 34.6, 33.6, 33.2, 26.5, 26.4, 26.2, 26.2, 17.9, 16.2, 8.5, 8.4. (All $^{13}$C NMR signals were listed without classifying to the corresponding diastereomers due to the complexity). **$^{19}$F NMR** (376 MHz, $CDCl_3$) $\delta$ -63.96, -65.10. Diastereomeric ratio (d.r.) = 1.4:1 (according to the ratio of integration of the two $^{19}$F NMR signals). **HRMS** (ESI) m/z: $[M+H]^+$ Calcd for $C_{23}H_{31}F_3NO^+$ 394.2358; Found 394.2365.

**4-(benzyloxy)-*N*-(5,5-dimethyl-3-(trifluoromethyl)nonan-2-yl)aniline (95)**

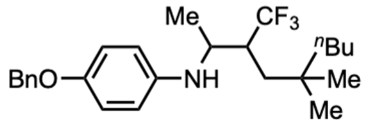

Using the General Procedure C, the title compound was obtained as a yellow viscous oil by preparative TLC using petroleum ether/EtOAc (15:1) as an eluent; $R_f$= 0.5 (petroleum ether/EtOAc = 10:1); 27.8 mg, 44% yield (d.r. = 1.2:1). **$^1$H NMR** (400 MHz, $CDCl_3$) $\delta$ 7.44 – 7.30 (m, 5H), 6.88 – 6.83 (m, 2H), 6.61 – 6.49 (m, 2H), 5.00 – 4.99 (m, 2H), 3.83 – 3.33 (m, 2H), 2.48 – 2.42 (m, 1H), 1.58 – 1.42 (m, 2H), 1.25 – 1.09 (m, 8H), 0.95 – 0.85 (m, 7H), 0.80 – 0.76 (m, 3H). **$^{13}$C NMR** (201 MHz, $CDCl_3$) $\delta$ 152.1, 151.4, 141.0, 140.9, 137.7, 137.6, 129.0 (q, $^1J_{CF}$ = 281.8 Hz), 128.7, 128.6, 128.6 (q, $^1J_{CF}$ = 281.3 Hz), 128.0, 127.9, 127.7, 127.6, 116.5, 116.3, 116.2, 114.2, 71.0, 70.9, 53.1, 49.0, 42.6, 42.4 (q, $^2J_{CF}$ = 22.4 Hz), 42.3, 41.3 (q, $^2J_{CF}$ = 22.9 Hz), 38.6, 35.6, 33.5, 33.1, 26.9, 26.8, 26.7, 26.4, 26.3, 23.7, 17.8, 16.2, 14.3, 14.2. (All $^{13}$C NMR signals were listed without

classifying to the corresponding diastereomers due to the complexity). **$^{19}$F NMR** (376 MHz, $CDCl_3$) $\delta$ -63.94, -65.09. Diastereomeric ratio (d.r.) = 1.2:1 (according to the ratio of integration of the two $^{19}$F NMR signals). **HRMS** (ESI) m/z: $[M+H]^+$ Calcd for $C_{25}H_{35}F_3NO^+$ 422.2671; Found 422.2668.

**4-(benzyloxy)-*N*-(5-(2,4-dimethylphenoxy)-4,4-dimethyl-2-(trifluoromethyl)pentyl)aniline (96)**

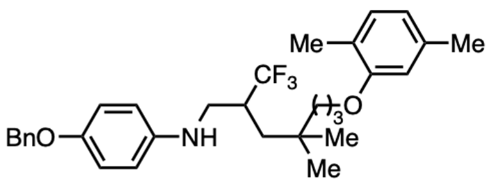


Using the General Procedure C, the title compound was obtained as a yellow viscous oil by preparative TLC using petroleum ether/EtOAc (15:1) as an eluent; $R_f$= 0.6 (petroleum ether/EtOAc = 10:1); 23.2 mg, 31% yield. **$^1$H NMR** (400 MHz, $CDCl_3$) $\delta$ 7.43 – 7.29 (m, 5H), 7.01 (d, $J$ = 7.4 Hz, 1H), 6.86 – 6.81 (m, 2H), 6.66 (d, $J$ = 7.5 Hz, 1H), 6.61 (s, 1H), 6.59 – 6.56 (m, 2H), 4.97 (s, 2H), 3.93 – 3.84 (m, 2H), 3.61 (brs, 1H), 3.37 – 3.22 (m, 2H), 2.46 – 2.34 (m, 1H), 2.40 (s, 3H), 2.30 (s, 3H), 1.80 – 1.73 (m, 2H), 1.63 – 1.62 (m, 1H), 1.43 – 1.33 (m, 3H), 1.00 – 0.93 (m, 6H). **$^{13}$C NMR** (201 MHz, $CDCl_3$) $\delta$ 157.1, 151.8, 141.9, 137.6, 136.6, 130.5, 128.7 (q, $^1J_{CF}$ = 280.5 Hz), 128.6, 127.9, 127.6, 123.7, 120.8, 116.3, 114.6, 112.1, 70.9, 68.5, 45.8, 38.7 (q, $^2J_{CF}$ = 23.9 Hz), 38.6, 38.2, 33.1, 26.7, 25.7, 24.4, 21.6, 15.9. **$^{19}$F NMR** (376 MHz, $CDCl_3$) $\delta$ -69.06 (s, 3F). **HRMS** (ESI) m/z: $[M+H]^+$ Calcd for $C_{31}H_{39}F_3NO_2^+$ 514.2933; Found 514.2938.

**4-(benzyloxy)-*N*-(6-(2,4-dimethylphenoxy)-5,5-dimethyl-3-(trifluoromethyl)-hexan-2-yl)aniline (97)**

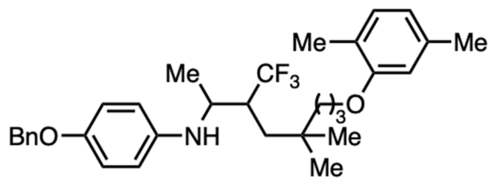


Using the General Procedure C, the title compound was obtained as a yellow viscous oil by preparative TLC using petroleum ether/EtOAc (15:1) as an eluent; $R_f$= 0.6 (petroleum ether/EtOAc =

10:1); 53.0 mg, 67% yield (d.r. = 1:1). **$^1$H NMR** (400 MHz, $CDCl_3$) $\delta$ 7.46 – 7.30 (m, 5H), 7.07 – 7.02 (m, 1H), 6.87 – 6.82 (m, 2H), 6.69 – 6.50 (m, 4H), 5.01 – 4.96 (m, 2H), 3.98 – 3.38 (m, 4H), 2.59 – 2.37 (m, 1H), 2.35 – 2.29 (m, 3H), 2.21 – 2.18 (m, 3H), 1.82 – 1.64 (m, 3H), 1.57 – 1.39 (m, 3H), 1.34 – 1.27 (m, 3H), 1.03 – 0.95 (m, 3H), 0.89 – 0.84 (m, 3H). **$^{13}$C NMR** (201 MHz, $CDCl_3$) $\delta$ 157.2, 157.1, 152.1, 151.4, 140.9, 140.8, 137.7, 137.6, 136.7, 136.6, 130.5, 130.4, 130.3 (q, $^1J_{CF}$ = 283.6 Hz), 128.7, 128.6, 128.5 (q, $^1J_{CF}$ = 281.2 Hz), 127.9, 127.8, 127.7, 127.6, 123.7, 123.6, 120.9, 120.8, 116.5, 116.3, 116.2, 114.1, 112.2, 112.1, 70.9, 70.8, 68.5, 53.2, 48.8, 42.5 (q, $^2J_{CF}$ = 22.5 Hz), 41.2 (q, $^2J_{CF}$ = 22.9 Hz), 38.7, 38.6, 35.5, 33.4, 33.0, 30.7, 27.0, 26.8, 26.7, 24.4, 24.3, 21.5, 19.3, 17.7, 16.3, 15.9, 15.8, 13.9. (All $^{13}$C NMR signals were listed without classifying to the corresponding diastereomers due to the complexity). **$^{19}$F NMR** (376 MHz, $CDCl_3$) $\delta$ -63.92, -65.06. Diastereomeric ratio (d.r.) = 1:1 (according to the ratio of integration of the two $^{19}$F NMR signals). **HRMS** (ESI) m/z: $[M+H]^+$ Calcd for $C_{32}H_{41}F_3NO_2^+$ 528.3089; Found 528.3090.

***N*-(4-((2-((adamantan-1-yl)methyl)-3,3,3-trifluoropropyl)amino)-3-(trifluorometh -yl)phenyl)isobutyramide (98)**

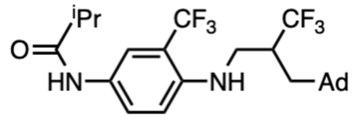


Using the General Procedure C, the title compound was obtained as a yellow viscous oil by preparative TLC using petroleum ether/EtOAc (10:1) as an eluent; $R_f$= 0.5 (petroleum ether/EtOAc = 10:1); 28.8 mg, 39% yield. **$^1$H NMR** (400 MHz, $CDCl_3$) $\delta$ 7.60 – 7.51 (m, 2H), 6.64 – 6.63 (m, 1H), 4.47 (brs, 1H), 3.36 – 3.21 (m, 2H), 2.53 – 2.42 (m, 2H), 1.99 – 1.94 (m, 3H), 1.71 – 1.67 (m, 3H), 1.61 – 1.55 (m, 3H), 1.52 – 1.42 (m, 7H), 1.21 (d, $J$ = 6.9 Hz, 6H), 1.15 – 1.09 (m, 1H). **$^{13}$C NMR** (151 MHz, $CDCl_3$) $\delta$ 175.8, 142.0, 128.3 (q, $^1J_{CF}$ = 279.6 Hz), 127.9, 126.2, 124.7 (q, $^1J_{CF}$ = 272.3 Hz), 119.8, 114.0 (q, $^2J_{CF}$ = 30.0 Hz), 112.1, 44.6, 42.3, 40.5, 37.0 (q, $^2J_{CF}$ = 25.7 Hz), 36.9, 36.4, 32.4, 28.5, 19.7. **$^{19}$F NMR** (376 MHz, $CDCl_3$) $\delta$ -62.48 (s, 3F), -69.47 (s, 3F). **HRMS** (ESI) m/z: $[M+Na]^+$ Calcd for $C_{25}H_{32}F_6N_2NaO^+$ 513.2317.; Found 513.2332.

***N*-(2-((adamantan-1-yl)methyl)-3,3,3-trifluoropropyl)-4-(2,4-dichlorophenoxy)-aniline (99)**

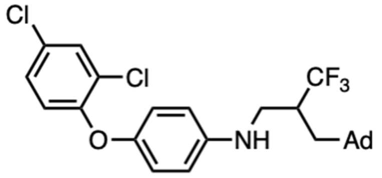


Using the General Procedure C, the title compound was obtained as a green viscous oil by preparative TLC using petroleum ether/EtOAc (10:1) as an eluent; $R_f$ = 0.5 (petroleum ether/EtOAc = 10:1); 39.0 mg, 52% yield. **$^1$H NMR** (400 MHz, $CDCl_3$) $\delta$ 7.42 (d, $J$ = 2.5 Hz, 1H), 7.09 (dd, $J$ = 8.8, 2.5 Hz, 1H), 6.88 (d, $J$ = 8.9 Hz, 2H), 6.73 (d, $J$ = 8.8 Hz, 1H), 6.60 (d, $J$ = 8.9 Hz, 2H), 3.83 (brs, 1H), 3.33 – 3.31 (m, 1H), 3.23 – 3.20 (m, 1H), 2.49 – 2.43 (m, 1H), 1.98 – 1.96 (m, 3H), 1.72 – 1.70 (m, 3H), 1.62 – 1.59 (m, 3H), 1.52 – 1.45 (m, 7H), 1.18 – 1.16 (m, 1H). **$^{13}$C NMR** (201 MHz, $CDCl_3$) $\delta$ 153.4, 147.6, 144.6, 130.3, 128.6 (q, $^1J_{CF}$ = 279.9 Hz), 127.8, 127.6, 124.9, 120.9, 118.8, 114.2, 45.4 (d, $^3J_{CF}$ = 2.7 Hz), 42.3, 40.6, 37.1 (q, $^2J_{CF}$ = 24.3 Hz), 36.9, 32.5, 28.5. **$^{19}$F NMR** (376 MHz, $CDCl_3$) $\delta$ -69.22 (s, 3F). **HRMS** (ESI) m/z: $[M+H]^+$ Calcd for $C_{26}H_{29}Cl_2F_3NO^+$ 498.1578; Found 498.1586.

***N*-(4,4-dimethyl-2-(trifluoromethyl)pentyl)aniline (103)**

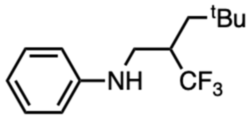


Using the General Procedure C, the title compound was obtained as a yellow viscous oil by preparative TLC using petroleum ether/EtOAc (15:1) as an eluent; $R_f$ = 0.6 (petroleum ether/EtOAc = 10:1); 12.0 mg, 31% yield. **$^1$H NMR** (400 MHz, $CDCl_3$) $\delta$ 7.20 (t, $J$ = 7.7 Hz, 2H), 6.7 (t, $J$ = 7.3 Hz, 1H), 6.62 (d, $J$ = 8.0 Hz, 2H), 3.87 (brs, 1H), 3.39 – 3.26 (m, 2H), 2.39 – 2.35 (m, 1H), 1.64 – 1.59 (m, 1H), 1.33 – 1.29 (m, 1H), 0.93 (s, 9H). **$^{13}$C NMR** (201 MHz, $CDCl_3$) $\delta$ 147.5, 129.5, 128.6 (q, $^1J_{CF}$ = 280.2 Hz), 118.1, 113.1, 44.7, 39.8, 39.1 (q, $^2J_{CF}$ = 24.3 Hz), 30.8, 29.4. **$^{19}$F NMR** (376 MHz, $CDCl_3$) $\delta$ -69.23 (s, 3F). **HRMS** (ESI) m/z: $[M+H]^+$ Calcd for $C_{14}H_{21}F_3N^+$ 260.1626; Found 260.1631.

**N-(4,4-dimethyl-2-(trifluoromethyl)pentyl)-2-methylaniline (104)**

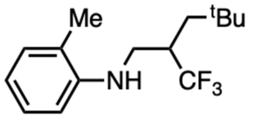

Using the General Procedure C, the title compound was obtained as a yellow amorphous solid by preparative TLC using petroleum ether/EtOAc (15:1) as an eluent; R*f* = 0.6 (petroleum ether/EtOAc = 10:1); 8.9 mg, 22% yield. **$^1$H NMR** (400 MHz, $CDCl_3$) $\delta$ 7.17 – 7.07 (m, 2H), 6.71 – 6.59 (m, 2H), 3.75 (brs, 1H), 3.42 – 3.29 (m, 2H), 2.47 – 2.40 (m, 1H), 2.13 (s, 3H), 1.67 – 1.63 (m, 1H), 1.38 – 1.33 (m, 1H), 0.95 (s, 9H). **$^{13}$C NMR** (201 MHz, $CDCl_3$) $\delta$ 145.5, 130.5, 128.6 (q, $^1J_{CF}$ = 280.0 Hz), 127.3, 122.4, 117.6, 109.5, 44.6, 39.9, 39.1 (q, $^2J_{CF}$ = 24.3 Hz), 30.8, 29.4, 17.5. **$^{19}$F NMR** (376 MHz, $CDCl_3$) $\delta$ -69.62 (s, 3F). **HRMS** (ESI) m/z: $[M+H]^+$ Calcd for $C_{15}H_{23}F_3N^+$ 274.1783; Found 274.1780.

**N-(4,4-dimethyl-2-(trifluoromethyl)pentyl)-3-fluoroaniline (105)**

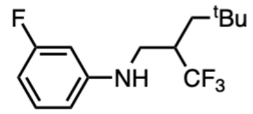

Using the General Procedure C, the title compound was obtained as a yellow viscous oil by preparative TLC using petroleum ether/EtOAc (15:1) as an eluent; R*f* = 0.6 (petroleum ether/EtOAc = 10:1); 9.3 mg, 22% yield. **$^1$H NMR** (400 MHz, $CDCl_3$) $\delta$ 7.14 – 7.08 (m, 1H), 6.44 – 6.27 (m, 3H), 3.99 (brs, 1H), 3.37 – 3.24 (m, 2H), 2.40 – 2.33 (m, 1H), 1.65 – 1.60 (m, 1H), 1.31 – 1.28 (m, 1H), 0.94 (s, 9H). **$^{13}$C NMR** (201 MHz, $CDCl_3$) $\delta$ 164.3 (d, $^1J_{CF}$ = 243.0 Hz), 149.3 (d, $^3J_{CF}$ = 10.9 Hz), 130.6 (d, $^3J_{CF}$ = 10.1 Hz), 128.5 (q, $^1J_{CF}$ = 279.9 Hz), 108.9, 104.5 (d, $^2J_{CF}$ = 21.5 Hz), 99.7 (d, $^2J_{CF}$ = 25.3 Hz), 44.6 (q, $^3J_{CF}$ = 3.4 Hz), 39.7, 39.1 (q, $^2J_{CF}$ = 24.4 Hz), 30.8, 29.4. **$^{19}$F NMR** (376 MHz, $CDCl_3$) $\delta$ -69.28 (s, 3F), -112.55 (s, 1F). **HRMS** (ESI) m/z: $[M+H]^+$ Calcd for $C_{14}H_{20}F_4N^+$ 278.1526; Found 278.1532.

**N-(4,4-dimethyl-2-(trifluoromethyl)pentyl)-4-methylaniline (106)**

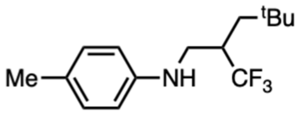

Using the General Procedure C, the title compound was obtained as a yellow viscous oil by preparative TLC using petroleum ether/EtOAc (15:1) as an eluent; R*f* = 0.6

(petroleum ether/EtOAc = 10:1); 15.0 mg, 37% yield. **$^1$H NMR** (600 MHz, $CDCl_3$) $\delta$ 7.02 – 7.00 (m, 2H), 6.56 – 6.54 (m, 2H), 3.36 – 3.25 (m, 2H), 2.41 – 2.34 (m, 1H), 2.25 (s, 3H), 1.63 – 1.60 (m, 1H), 1.32 – 1.28 (m, 1H), 0.94 (s, 9H). (Due to the broadening effect and intermolecular hydrogen bonding, one of the proton signals of NH could not be observed.). **$^{13}$C NMR** (101 MHz, $CDCl_3$) $\delta$ 145.2, 130.0, 128.5 (q, $^1J_{CF}$ = 260.0 Hz), 127.4, 113.3, 45.1, 39.8, 39.0 (q, $^2J_{CF}$ = 24.1 Hz), 30.8, 29.4, 20.5. **$^{19}$F NMR** (565 MHz, $CDCl_3$) $\delta$ -69.13 (s, 3F). **HRMS** (ESI) m/z: $[M+H]^+$ Calcd for $C_{15}H_{23}F_3N^+$ 274.1783; Found 274.1787.

**4-chloro-*N*-(4,4-dimethyl-2-(trifluoromethyl)pentyl)aniline (107)**

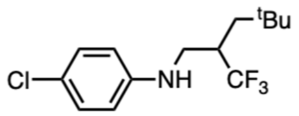


Using the General Procedure C, the title compound was obtained as a yellow viscous oil by preparative TLC using petroleum ether/EtOAc (15:1) as an eluent; R*f* = 0.6 (petroleum ether/EtOAc = 10:1); 15.1 mg, 34% yield. **$^1$H NMR** (400 MHz, $CDCl_3$) $\delta$ 7.14 (d, $J$ = 9.2 Hz, 2H), 6.53 (d, $J$ = 9.1 Hz, 2H), 3.88 (brs, 1H), 3.36 – 3.22 (m, 2H), 2.40 – 2.29 (m, 1H), 1.64 – 1.60 (m, 1H), 1.30 – 1.29 (m, 1H), 0.93 (s, 9H). **$^{13}$C NMR** (101 MHz, $CDCl_3$) $\delta$ 146.1, 129.4, 128.5 (q, $^1J_{CF}$ = 278.9 Hz), 122.7, 114.1, 44.8, 39.7, 39.0 (q, $^2J_{CF}$ = 24.3 Hz), 30.8, 29.4. **$^{19}$F NMR** (376 MHz, $CDCl_3$) $\delta$ -69.26 (s, 3F). **HRMS** (ESI) m/z: $[M+H]^+$ Calcd for $C_{14}H_{20}ClF_3N^+$ 294.1236; Found 294.1230.

***N*-(2-((adamantan-1-yl)methyl)-3,3,3-trifluoropropyl)aniline (110)**

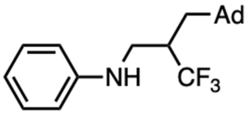


Using the General Procedure C, the title compound was obtained as a yellow viscous oil by preparative TLC using petroleum ether/EtOAc (15:1) as an eluent; R*f* = 0.6 (petroleum ether/EtOAc = 5:1); 34.3 mg, 58% yield. **$^1$H NMR** (400 MHz, $CDCl_3$) $\delta$ 7.24 – 7.19 (m, 2H), 6.78 – 6.73 (m, 1H), 6.65 – 6.62 (m, 2H), 3.85 (brs, 1H), 3.37 – 3.23 (m, 2H), 2.52 – 2.42 (m, 1H), 2.00 – 1.97 (m, 3H), 1.74 – 1.71 (m, 3H), 1.65 – 1.59 (m, 3H), 1.55 – 1.46 (m, 7H), 1.21 – 1.16 (m, 1H). **$^{13}$C NMR** (151 MHz, $CDCl_3$) $\delta$ 147.6, 129.5, 128.7 (q, $^1J_{CF}$ = 279.9 Hz), 118.1, 113.1, 44.9, 42.3, 40.7, 37.2 (q, $^2J_{CF}$

= 24.5 Hz). 37.0, 32.5, 28.6. **$^{19}$F NMR** (376 MHz, $CDCl_3$) $\delta$ -69.21 (s, 3F). **HRMS** (ESI) m/z: $[M+H]^+$ Calcd for $C_{20}H_{27}F_3N^+$ 338.2096; Found 338.2101.

***N*-(2-((adamantan-1-yl)methyl)-3,3,3-trifluoropropyl)-3-chloroaniline (111)**

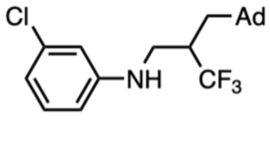


Using the General Procedure C, the title compound was obtained as a red viscous oil by preparative TLC using petroleum ether/EtOAc (15:1) as an eluent; R*f* = 0.6 (petroleum ether/EtOAc = 10:1); 16.9 mg, 30% yield. **$^1$H NMR** (800 MHz, $CDCl_3$) $\delta$ 7.09 (t, *J* = 8.0 Hz, 1H), 6.70 (d, *J* = 8.0 Hz, 1H), 6.59 (s, 1H), 6.48 (d, *J* = 8.3 Hz, 1H), 3.93 (brs, 1H), 3.31 – 3.29 (m, 1H), 3.23 – 3.21 (m, 1H), 2.47 – 2.41 (m, 1H), 1.99 – 1.97 (m, 3H), 1.72 – 1.70 (m, 3H), 1.63 – 1.60 (m, 3H), 1.52 – 1.46 (m, 7H), 1.16 – 1.13 (m, 1H). **$^{13}$C NMR** (201 MHz, $CDCl_3$) $\delta$ 148.7, 135.3, 130.4, 128.5 (q, $^1J_{CF}$ = 280.0 Hz), 117.9, 112.7, 111.5, 44.7 (q, $^3J_{CF}$ = 2.9 Hz), 42.3, 40.5, 37.1 (q, $^2J_{CF}$ = 24.2 Hz), 36.9, 32.5, 28.5. **$^{19}$F NMR** (376 MHz, $CDCl_3$) $\delta$ -69.25 (s, 3F). **HRMS** (ESI) m/z: $[M+H]^+$ Calcd for $C_{20}H_{26}ClF_3N^+$ 372.1706; Found 372.1711.

***N*-(2-((adamantan-1-yl)methyl)-3,3,3-trifluoropropyl)-3-fluoroaniline (112)**

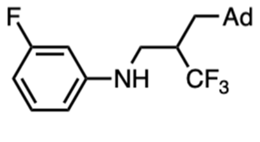


Using the General Procedure C, the title compound was obtained as a yellow viscous oil by preparative TLC using petroleum ether/EtOAc (15:1) as an eluent; R*f* = 0.6 (petroleum ether/EtOAc = 10:1); 20.3 mg, 38% yield. **$^1$H NMR** (400 MHz, $CDCl_3$) $\delta$ 7.14 – 7.08 (m, 1H), 6.44 – 6.27 (m, 3H), 3.97 (brs, 1H), 3.30 – 3.19 (m, 2H), 2.48 – 2.38 (m, 1H), 1.98 – 1.96 (m, 3H), 1.72 – 1.69 (m, 3H), 1.62 – 1.59 (m, 3H), 1.52 – 1.44 (m, 7H), 1.18 – 1.12 (m, 1H). **$^{13}$C NMR** (101 MHz, $CDCl_3$) $\delta$ 164.3 (d, $^1J_{CF}$ = 243.2 Hz), 149.4 (d, $^3J_{CF}$ = 10.6 Hz), 130.6 (d, $^3J_{CF}$ = 10.1 Hz), 128.5 (q, $^1J_{CF}$ = 279.9 Hz), 108.9 (d, $^4J_{CF}$ = 2.3 Hz), 104.5 (d, $^2J_{CF}$ = 21.6 Hz), 99.7 (d, $^2J_{CF}$ = 25.4 Hz), 44.7 (q, $^3J_{CF}$ = 2.9 Hz), 42.3, 40.5, 37.1 (q, $^2J_{CF}$ = 24.5 Hz), 36.9, 32.5, 28.5. **$^{19}$F NMR** (376 MHz, $CDCl_3$) $\delta$ -69.26 (s, 3F), -112.57 (s, 1F). **HRMS** (ESI) m/z: $[M+H]^+$ Calcd for $C_{20}H_{26}F_4N^+$ 356.2001; Found 356.2008.

**N-(2-((adamantan-1-yl)methyl)-3,3,3-trifluoropropyl)-4-methylaniline (113)**

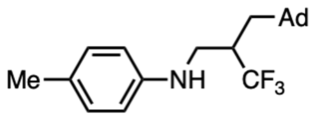


Using the General Procedure C, the title compound was obtained as a yellow amorphous solid by preparative TLC using petroleum ether/EtOAc (15:1) as an eluent; R*f* = 0.6 (petroleum ether/EtOAc = 10:1); 34.3 mg, 65% yield. **$^1$H NMR** (400 MHz, $CDCl_3$) $\delta$ 7.02 (d, $J$ = 8.1 Hz, 2H), 6.56 (d, $J$ = 8.4 Hz, 2H), 3.71 (brs, 1H), 3.34 – 3.21 (m, 2H), 2.51 – 2.39 (m, 1H), 2.26 (s, 3H), 2.00 – 1.97 (m, 3H), 1.73 – 1.70 (m, 3H), 1.64 – 1.59 (m, 3H), 1.55 – 1.45 (m, 7H), 1.20 – 1.15 (m, 1H). **$^{13}$C NMR** (101 MHz, $CDCl_3$) $\delta$ 145.3, 130.0, 128.7 (q, $^1J_{CF}$ = 279.8 Hz), 127.3, 113.3, 45.3, 42.3, 40.7, 37.2 (q, $^2J_{CF}$ = 24.3 Hz). 37.0, 32.5, 28.6, 20.5. **$^{19}$F NMR** (376 MHz, $CDCl_3$) $\delta$ -69.21 (s, 3F). **HRMS** (ESI) m/z: $[M+H]^+$ Calcd for $C_{21}H_{29}F_3N^+$ 352.2252; Found 352.2260.

**4-(benzyloxy)-N-(2-(trifluoromethyl)pentyl)aniline (124)**

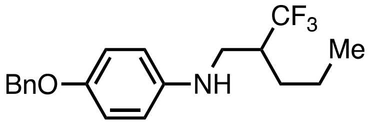


Using the General Procedure C, the title compound was obtained as a yellow viscous oil by preparative TLC using petroleum ether/EtOAc (15:1) as an eluent; R*f*= 0.6 (petroleum ether/EtOAc = 10:1); 21.8 mg, 43% yield. **$^1$H NMR** (400 MHz, $CDCl_3$) $\delta$ 7.44 – 7.29 (m, 5H), 6.87 (d, $J$ = 8.3 Hz, 2H), 6.58 (d, $J$ = 8.4 Hz, 2H), 5.00 (s, 2H), 3.58 (brs, 1H), 3.38 – 3.21 (m, 2H), 2.43 – 2.30 (m, 1H), 1.67 – 1.61 (m, 1H), 1.53 – 1.42 (m, 3H), 0.94 (t, $J$ = 7.1 Hz, 3H). **$^{13}$C NMR** (101 MHz, $CDCl_3$) $\delta$ 151.8, 141.8, 137.7, 128.7, 128.3 (q, $^1J_{CF}$ = 280.5 Hz), 127.9, 127.6, 116.4, 114.4, 71.0, 43.3 (q, $^3J_{CF}$ = 2.9 Hz), 42.3 (q, $^2J_{CF}$ = 23.9 Hz), 28.7 (q, $^3J_{CF}$ = 2.4 Hz), 20.3, 14.2. **$^{19}$F NMR** (376 MHz, $CDCl_3$) $\delta$ -69.28 (s, 3F). **HRMS** (ESI) m/z: $[M+H]^+$ Calcd for $C_{19}H_{23}F_3NO^+$ 338.1726; Found 338.1729.

**4-(benzyloxy)-N-(4-cyclopentyl-2-(trifluoromethyl)butyl)aniline (125)**

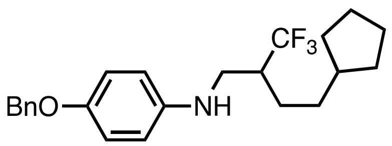

Using the General Procedure C, the title compound was obtained as a pale yellow amorphous solid by preparative TLC using petroleum ether/EtOAc (20:1) as an eluent; $R_f$= 0.4 (petroleum ether/EtOAc = 10:1); 15.4 mg, 26% yield. $^1$H NMR (800 MHz, $CDCl_3$) $\delta$ 7.43 (d, $J$ = 7.5 Hz, 2H), 7.38 (t, $J$ = 7.5 Hz, 2H), 7.31 (t, $J$ = 7.3 Hz, 1H), 6.87 (d, $J$ = 8.5 Hz, 2H), 6.58 (d, $J$ = 8.5 Hz, 2H), 5.00 (s, 2H), 3.58 (brs, 1H), 3.36 – 3.34 (m, 1H), 3.25 – 3.23 (m, 1H), 2.37 – 2.32 (m, 1H), 1.77 – 1.65 (m, 4H), 1.60 – 1.57 (m, 2H), 1.54 – 1.50 (m, 3H), 1.46 – 1.39 (m, 2H), 1.10 – 1.05 (m, 2H). $^{13}$C NMR (201 MHz, $CDCl_3$) $\delta$ 151.8, 141.9, 137.6, 128.7, 128.4 (q, $^1J_{CF}$ = 281.8 Hz), 128.0, 127.6, 116.4, 114.4, 71.0, 43.2 (q, $^3J_{CF}$ = 2.8 Hz), 42.6 (q, $^2J_{CF}$ = 23.8 Hz), 40.2, 33.4, 32.7, 32.6, 25.3. $^{19}$F NMR (471 MHz, $CDCl_3$) $\delta$ -69.18 (s, 3F). HRMS (ESI) m/z: $[M+H]^+$ Calcd for $C_{23}H_{28}F_3NO^+$ 406.1964; Found 512.2396.

## Diverse Functionalization of the *N*-trifluoroalkyl aniline products[4-12].

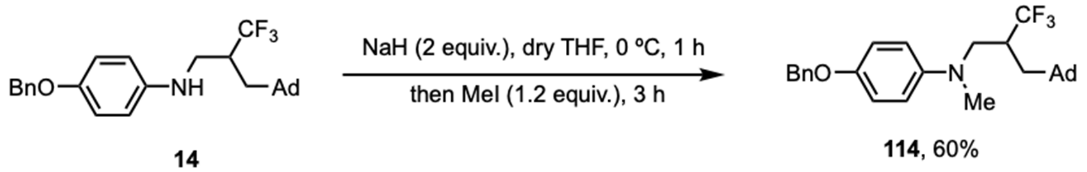


To a 10 mL round bottom flask equipped with a magnetic stirring bar was added, **14** (0.2 mmol, 88.71 mg, 1.0 equiv.) was dissolved in dry THF (0.3 mL, 0.7 M) and cooled to 0 °C in an ice-bath.. To this solution was added NaH (60% in mineral oil, 12.00 mg, 1.5 equiv.) portionwise. The mixture was stirred at 0 ºC for 1 h before adding MeI (15.0 $\mu$L, 1.2 equiv.) dropwise. Then the mixture was allowed to warm to room temperature and stirred for another 3 h. Upon completion of the reactions (TLC showed complete consumption of starting material). The reaction was quenched with saturated $NH_4Cl$ (10 mL) and extracted with EtOAc (3 × 10 mL). The combined organic layers were washed with brine, dried over anhydrous $Na_2SO_4$, filtered and concentrated in *vacuo*. The residue was purified by preparative thin-layer chromatography using a mixture of petroleum ether and ethyl acetate (PE:EA = 15:1) as an eluent to afford the product **114**.

***N*-(2-((adamantan-1-yl)methyl)-3,3,3-trifluoropropyl)-4-(benzyloxy)-*N*-methylaniline (114).** The title compound was obtained as a white yellow amorphous solid by preparative TLC using petroleum ether/EtOAc (15:1) as an eluent; $R_f$= 0.6 (petroleum ether/EtOAc = 10:1); 54.9 mg, 60% yield. **$^1$H NMR** (400 MHz, $CDCl_3$) $\delta$ 7.47 – 7.32 (m, 5H), 6.96 – 6.92 (m, 2H), 6.73 – 6.68 (m, 2H), 5.03 (s, 2H), 3.51 – 3.22 (m, 2H), 2.92 (s, 3H), 2.60 – 2.47 (m, 1H), 1.98 – 1.96 (m, 3H), 1.73 – 1.70 (m, 3H), 1.63 – 1.60 (m, 3H), 1.55 – 1.42 (m, 7H), 1.10 – 1.06 (m, 1H). **$^{13}$C NMR** (101 MHz, $CDCl_3$) $\delta$ 151.1, 144.2, 137.7, 128.6, 128.4 (q, $^1J_{CF}$ = 279.6 Hz), 127.9, 127.6, 116.1, 114.3, 70.8, 55.5, 42.3, 41.2, 40.2, 36.9, 35.4 (q, $^2J_{CF}$ = 23.8 Hz), 32.4, 28.6. **$^{19}$F NMR** (376

MHz, $CDCl_3$) δ -68.74 (s, 3F). **HRMS** (ESI) m/z: $[M+H]^+$ Calcd for $C_{28}H_{35}F_3NO^+$ 458.2671; Found 458.2681.

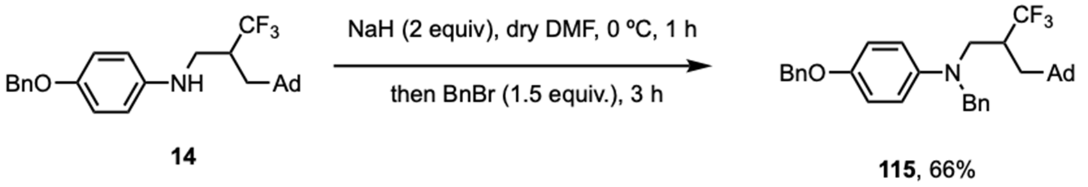

To a 10 mL round bottom flask equipped with a magnetic stirring bar was added, **14** (0.2 mmol, 88.71 mg, 1.0 equiv.) was dissolved in dry *N*,*N*-dimethylformamide (0.4 mL) and cooled to 0 °C in an ice-bath. To this solution was added NaH (60% in mineral oil, 12.00 mg, 1.5 equiv.) portionwise and it was stirred for 1 h. A solution of benzyl bromide (36.0 μL, 1.5 equiv.) in *N*,*N*-dimethylformamide solution (0.1 mL) was added dropwise to the reaction mixture at 0 ºC. Then the mixture was allowed to warm to room temperature and stirred for another 3 h. Upon completion of the reactions (TLC showed complete consumption of starting material). The reaction was quenched with saturated $NH_4Cl$ (10 mL) and extracted with EtOAc (3 × 10 mL). The combined organic layers were were washed with brine, dried over anhydrous $Na_2SO_4$, filtered and concentrated in *vacuo*. The residue was purified by preparative thin-layer chromatography using a mixture of petroleum ether and ethyl acetate (PE:EA = 12:1) as an eluent to afford the product **115**.

***N*-(2-((adamantan-1-yl)methyl)-3,3,3-trifluoropropyl)-*N*-benzyl-4-(benzyloxy)aniline (115).** The title compound was obtained as a brown amorphous solid by preparative TLC using petroleum ether/EtOAc (12:1) as an eluent; $R_f$= 0.5 (petroleum ether/EtOAc = 10:1); 85.3 mg, 66% yield. **$^1$H NMR** (400 MHz, $CDCl_3$) δ 7.45 – 7.29 (m, 8H), 7.24 – 7.21 (m, 2H), 6.91 (d, *J* = 8.7 Hz, 2H), 6.77 (d, *J* = 8.6 Hz, 2H), 5.00 (s, 2H), 4.59 – 4.51 (m, 2H), 3.64 – 3.27 (m, 2H), 2.64 – 2.57 (m, 1H), 1.95 – 1.93 (m, 3H), 1.71 – 1.68 (m, 3H), 1.61 – 1.57 (m, 3H), 1.50 – 1.36 (m, 7H), 1.04 – 0.96 (m, 1H). **$^{13}$C NMR** (101 MHz, $CDCl_3$) δ 151.5, 142.7, 138.6, 137.6, 128.6, 128.5, 128.4 (q, $^1J_{CF}$ = 280.8 Hz), 127.9, 127.6, 127.4, 127.02 116.1, 115.9, 72.2, 70.61 57.0, 42.3, 41.3, 36.9, 35.1 (q, $^2J_{CF}$ = 24.1 Hz), 32.4, 28.5. **$^{19}$F NMR** (376 MHz, $CDCl_3$) δ -68.66

(s, 3F). **HRMS** (ESI) m/z: $[M+H]^+$ Calcd for $C_{34}H_{39}F_3NO^+$ 534.2984; Found 534.2993.

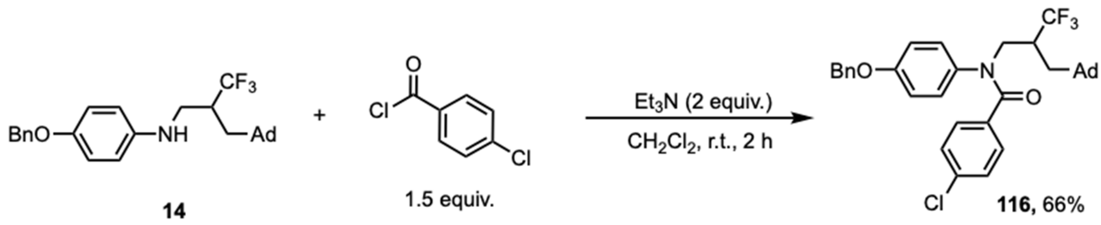

To a stirred solution of **14** (0.2 mmol, 88.71 mg, 1.0 equiv.) in dry dichloromethane (2 mL, 0.1 M) were added triethylamine (56 $\mu$L, 2.0 equiv.) and 4-Chlorobenzoyl chloride (31 $\mu$L, 1.2 equiv.) dropwisely under 0 °C. Then the mixture was allowed to warm to room temperature and stirred for 2 h. Upon completion of the reactions (TLC showed complete consumption of starting material). The reaction was quenched with saturated $H_2O$ (10 mL) and extracted with $CH_2Cl_2$ (3 × 10 mL). The combined organic layers were were washed with brine, dried over anhydrous $MgSO_4$, filtered and concentrated in *vacuo*. The residue was purified by preparative thin-layer chromatography using a mixture of petroleum ether and ethyl acetate (PE:EA = 12:1) as an eluent to afford the product **116**.

***N*-(2-((adamantan-1-yl)methyl)-3,3,3-trifluoropropyl)-*N*-(4-(benzyloxy)phenyl)-4-chlorobenzamide (116).** The title compound was obtained as a white amorphous solid by preparative TLC using petroleum ether/EtOAc (12:1) as an eluent; $R_f$= 0.5 (petroleum ether/EtOAc = 10:1); 76.8 mg, 66% yield. **$^1$H NMR** (400 MHz, $CDCl_3$) $\delta$ 7.39 – 7.32 (m, 5H), 7.23 – 7.13 (m, 4H), 6.98 – 6.82 (m, 4H), 5.00 (s, 2H), 4.14 – 3.77 (m, 2H), 3.03 – 2.84 (m, 1H), 2.01 – 1.87 (m, 3H), 1.70 – 1.67 (m, 3H), 1.61 – 1.57 (m, 3H), 1.53 – 1.39 (m, 7H), 0.97 – 0.89 (m, 1H). **$^{13}$C NMR** (101 MHz, $CDCl_3$) $\delta$ 169.9, 157.5, 136.4, 135.6, 134.6, 130.0, 128.9, 128.7, 128.4 (q, $^1J_{CF}$ = 280.1 Hz), 128.2, 128.1, 127.5, 115.5, 70.2, 52.2, 42.0, 40.9, 36.8, 34.5 (q, $^2J_{CF}$ = 24.9 Hz), 32.5, 28.4. **$^{19}$F NMR** (376 MHz, $CDCl_3$) $\delta$ -68.82 (s, 3F). **HRMS** (ESI) m/z: $[M+Na]^+$ Calcd for $C_{34}H_{35}ClF_3NNaO_2^+$ 604.2206; Found 604.2216.

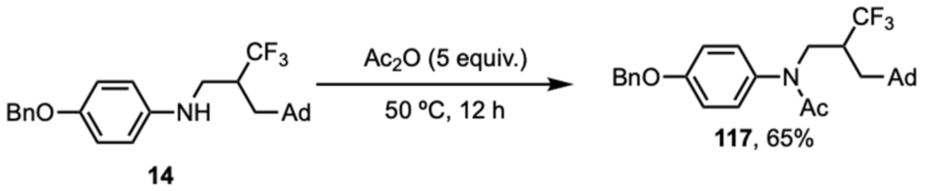

To a 10 mL round bottom flask equipped with a magnetic stirring bar was added, a mixture of $Ac_2O$ (94 $\mu$L, 5.0 equiv.) and **14** (0.2 mmol, 88.71 mg, 1.0 equiv.) were stirred at 50 °C for 12 h. Then the mixture was allowed to cooled to room temperature. Upon completion of the reactions (TLC showed complete consumption of starting material). The reaction was neutralized with saturated NaHCO3 (10 mL) and extracted with CH2Cl2 (3 × 10 mL). The combined organic layers were were washed with brine, dried over anhydrous $MgSO_4$, filtered and concentrated in *vacuo*. The residue was purified by preparative thin-layer chromatography using a mixture of petroleum ether and ethyl acetate (PE:EA = 12:1) as an eluent to afford the product **117**.

***N*-(2-((adamantan-1-yl)methyl)-3,3,3-trifluoropropyl)-*N*-(4-(benzyloxy)phenyl)acetamide (117).** The title compound was obtained as a white amorphous solid by preparative TLC using petroleum ether/EtOAc (12:1) as an eluent; $R_f$= 0.5 (petroleum ether/EtOAc = 10:1); 63.1 mg, 65% yield. **$^1$H NMR** (400 MHz, $CDCl_3$) $\delta$ 7.45 – 7.33 (m, 5H), 7.15 – 7.11 (m, 2H), 7.08 – 7.00 (m, 2H), 5.08 (s, 2H), 4.27 – 4.21 (m, 1H), 3.36 – 3.32 (m, 1H), 2.68 – 2.61 (m, 1H), 1.88 – 1.84 (m, 6H), 1.66 – 1.63 (m, 3H), 1.53 – 1.50 (m, 3H), 1.37 – 1.26 (m, 7H), 0.90 – 0.85 (m, 1H). **$^{13}$C NMR** (101 MHz, $CDCl_3$) $\delta$ 171.2, 158.3, 136.5, 135.9, 129.3, 128.8, 128.3 (q, $^1J_{CF}$ = 279.9 Hz), 128.3, 127.5, 115.8, 70.4, 50.2, 42.0, 40.6, 36.8, 34.8 (q, $^2J_{CF}$ = 24.8 Hz), 32.4, 28.4, 22.9. **$^{19}$F NMR** (376 MHz, $CDCl_3$) $\delta$ -69.33 (s, 3F). **HRMS** (ESI) m/z: $[M+Na]^+$ Calcd for $C_{29}H_{34}F_3NNaO_2^+$ 508.2439; Found 508.2447.

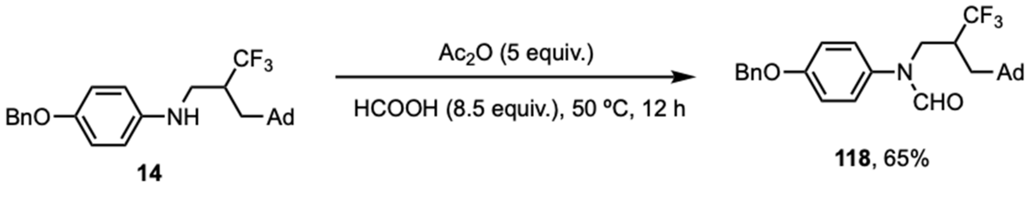

To a 10 mL round bottom flask equipped with a magnetic stirring bar was added, a mixture of formic acid (64 $\mu$L, 8.5 equiv.) and $Ac_2O$ (94 $\mu$L, 5.0 equiv.) was stirred at 70 °C for 1 h. Then the mixture was allowed to cooled to room temperature. **14** (0.2 mmol, 88.71 mg, 1.0 equiv.) was added slowly and the resulting mixture was stirred at 50 °C for 12 h. Upon completion of the reactions (TLC showed complete consumption of starting material). The reaction was neutralized with saturated NaHCO3 (10 mL) and extracted with CH2Cl2 (3 × 10 mL). The combined organic layers were were washed with brine, dried over anhydrous $MgSO_4$, filtered and concentrated in *vacuo*. The residue was purified by preparative thin-layer chromatography using a mixture of petroleum ether and ethyl acetate (PE:EA = 12:1) as an eluent to afford the product **118**.

***N*-(2-((adamantan-1-yl)methyl)-3,3,3-trifluoropropyl)-*N*-(4-(benzyloxy)phenyl)formamide (118).** The title compound was obtained as a white amorphous solid by preparative TLC using petroleum ether/EtOAc (12:1) as an eluent; $R_f$= 0.5 (petroleum ether/EtOAc = 10:1); 61.3 mg, 65% yield. **$^1$H NMR** (400 MHz, $CDCl_3$) $\delta$ 8.26 (s, 1H), 7.45 – 7.33 (m, 5H), 7.12 – 7.09 (m, 2H), 7.05 – 7.00 (m, 2H), 5.08 (s, 2H), 4.20 – 4.14 (m, 1H), 3.64 – 3.59 (m, 1H), 2.62 – 2.55 (m, 1H), 1.94 – 1.85 (m, 3H), 1.69 – 1.62 (m, 3H), 1.57 – 1.54 (m, 3H), 1.47 – 1.36 (m, 7H), 1.00 – 0.95 (m, 1H). **$^{13}$C NMR** (101 MHz, $CDCl_3$) $\delta$ 163.0, 157.9, 136.5, 133.5, 128.8, 128.3, 128.0 (q, $^1J_{CF}$ = 280.2 Hz), 127.6, 126.3, 115.8, 70.4, 46.3, 42.1, 40.7, 36.8, 34.9 (q, $^2J_{CF}$ = 26.0 Hz), 32.4, 28.4. **$^{19}$F NMR** (376 MHz, $CDCl_3$) $\delta$ -69.51 (s, 3F). **HRMS** (ESI) m/z: $[M+Na]^+$ Calcd for $C_{28}H_{32}F_3NNaO_2^+$ 494.2283; Found 494.2291.

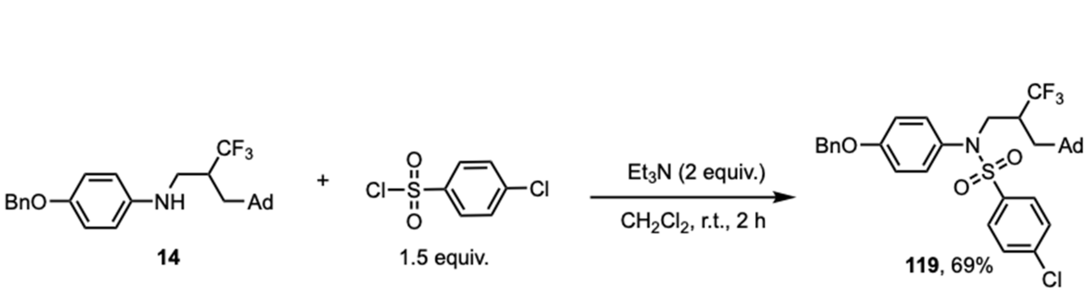


**14** 1.5 equiv. **119**, 69%

To a stirred solution of **14** (0.2 mmol, 88.71 mg, 1.0 equiv.) in dry dichloromethane (2 mL, 0.1 M) were added triethylamine (56 $\mu$L, 2.0 equiv.) and

4-chlorobenzenesulfonyl chloride (34 $\mu$L, 1.2 equiv.) dropwisely under 0 °C. Then the mixture was allowed to warm to room temperature and stirred for 2 h. Upon completion of the reactions (TLC showed complete consumption of starting material). The reaction was quenched with saturated $H_2O$ (10 mL) and extracted with $CH_2Cl_2$ (3 × 10 mL). The combined organic layers were were washed with brine, dried over anhydrous $MgSO_4$, filtered and concentrated in *vacuo*. The residue was purified by preparative thin-layer chromatography using a mixture of petroleum ether and ethyl acetate (PE:EA = 12:1) as an eluent to afford the product **119**.

***N*-(2-((adamantan-1-yl)methyl)-3,3,3-trifluoropropyl)-*N*-(4-(benzyloxy)phenyl)-4-chlorobenzenesulfonamide (119).** The title compound was obtained as a brown amorphous solid by preparative TLC using petroleum ether/EtOAc (12:1) as an eluent; $R_f$= 0.5 (petroleum ether/EtOAc = 10:1); 85.3 mg, 69% yield. **$^1$H NMR** (400 MHz, $CDCl_3$) $\delta$ 7.48 – 7.33 (m, 9H), 6.98 – 6.90 (m, 4H), 5.06 (s, 2H), 3.88 – 3.83 (m, 1H), 3.38 – 3.33 (m, 1H), 2.43 – 2.34 (m, 1H), 1.91 – 1.89 (m, 3H), 1.67 – 1.64 (m, 3H), 1.57 – 1.51 (m, 3H), 1.45 – 1.29 (m, 7H), 1.07 – 1.02 (m, 1H). **$^{13}$C NMR** (101 MHz, $CDCl_3$) $\delta$ 158.7, 139.5, 136.5, 136.1, 131.5, 130.2, 129.3, 129.2, 128.8, 128.3, 127.8 (q, $^1J_{CF}$ = 280.8 Hz), 127.7, 115.0, 70.4, 52.0, 42.1, 40.5, 36.9 (q, $^2J_{CF}$ = 24.8 Hz), 36.8, 32.4, 28.4. **$^{19}$F NMR** (376 MHz, $CDCl_3$) $\delta$ -69.14 (s, 3F). **HRMS** (ESI) m/z: $[M+Na]^+$ Calcd for $C_{33}H_{35}ClF_3NNaO_3S^+$ 640.1876; Found 640.1884.

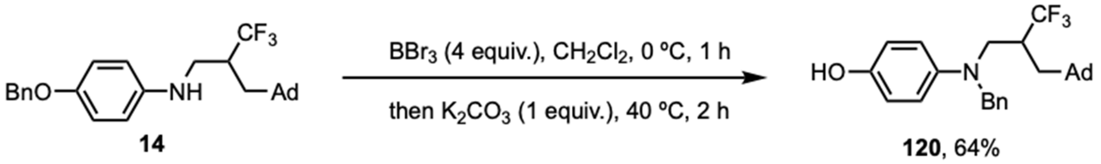


To a 10 mL round bottom flask equipped with a magnetic stirring bar was added, $BBr_3$ (1 M in DCM, 0.8 mL, 4.0 equiv.) was added dropwise to a solution of **14** (0.2 mmol, 88.71 mg, 1.0 equiv.) in dichloromethane (0.2 mL, 1 M) at 0 ºC. The mixture was stirred at 0 ºC for 1 h. Upon completion of the reactions (TLC showed complete consumption of starting material). $K_2CO_3$ (27.64 mg, 1.0 equiv.) was added to the reaction mixture. Then the mixture was allowed to warm to 40 ºC and stirred for 2 h.

The reaction was distilled with water (10 mL), extracted with $CH_2Cl_2$ (3 × 10 mL). The combined organic layers were washed with brine, dried over anhydrous $MgSO_4$, filtered and concentrated in *vacuo*. The residue was purified by preparative thin-layer chromatography using a mixture of petroleum ether and ethyl acetate (PE:EA = 5:1) as an eluent to afford the product **120**.

**4-((2-((adamantan-1-yl)methyl)-3,3,3-trifluoropropyl)(benzyl)amino)phenol (120).** The title compound was obtained as a brown amorphous solid by preparative TLC using petroleum ether/EtOAc (5:1) as an eluent; $R_f$ = 0.5 (petroleum ether/EtOAc = 2:1); 56.8 mg, 64% yield. **$^1$H NMR** (400 MHz, $CD_3OD$_SPE) $\delta$ 7.28 – 7.17 (m, 5H), 6.77 – 6.66 (m, 4H), 4.42 – 4.34 (m, 2H), 3.5 (dd, $J$ = 14.4, 6.7 Hz, 1H), 3.1 (dd, $J$ = 14.4, 7.3 Hz, 1H), 2.53 – 2.46 (m, 1H), 1.87 – 1.84 (m, 3H), 1.69 – 1.66 (m, 3H), 1.58 – 1.55 (m, 3H), 1.40 – 1.28 (m, 7H), 1.06 – 1.01 (m, 1H). **$^{13}$C NMR** (101 MHz, $CD_3OD$_SPE) $\delta$ 151.6, 143.4, 140.1, 129.8 (q, $^1J_{CF}$ = 279.8 Hz), 129.3, 129.0, 128.0, 119.7, 116.8, 59.7, 54.1, 43.4, 42.3, 37.9, 36.4 (q, $^2J_{CF}$ = 23.7 Hz), 33.4, 29.9. **$^{19}$F NMR** (376 MHz, $CD_3OD$_SPE) $\delta$ -70.33 (s, 3F). **HRMS** (ESI) m/z: $[M+H]^+$ Calcd for $C_{27}H_{33}F_3NO^+$ 444.2514; Found 444.2516.

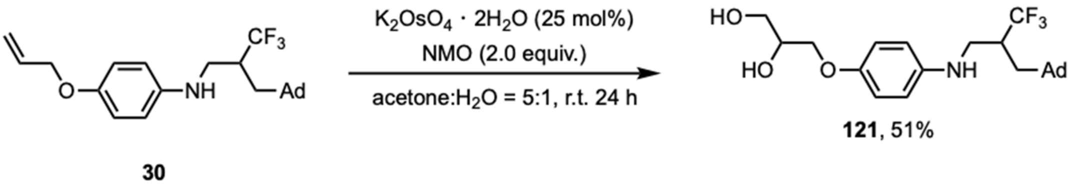


An oven-dried, transparent 10 mL round bottom flask equipped with a stir bar was sequentially charged with $K_2OSO_4$•$2H_2O$ (15.57 mg, 25 mol%), *N*-methylmorpholine *N*-oxide (NMO) (46.86 mg, 2 equiv.), acetone (5 mL), $H_2O$ (1 mL) and **30** (0.2 mmol, 78.70 mg, 1.0 equiv.) at room temperature. The mixture was stirred at the same temperature for 24 h. Upon completion of the reactions (TLC showed complete consumption of starting material). Then the mixture was distilled with water (10 mL), and extracted with EtOAc (3 × 10 mL). The combined organic layers were washed with brine, dried over $Na_2SO_4$, filtered and concentrated in *vacuo*. The residue was

purified by preparative thin-layer chromatography using a mixture of petroleum ether and ethyl acetate (PE:EA = 5:1) as an eluent to afford the product **121**.

**3-(4-((2-((adamantan-1-yl)methyl)-3,3,3-trifluoropropyl)amino)phenoxy)propane-1,2-diol (121).** The title compound was obtained as a yellow amorphous solid by preparative TLC using petroleum ether/EtOAc (5:1) as an eluent; $R_f$= 0.5 (petroleum ether/EtOAc = 1:1); 43.6 mg, 51% yield. **$^1$H NMR** (400 MHz, $CDCl_3$) $\delta$ 6.82 – 6.79 (m, 2H), 6.59 – 6.57 (m, 2H), 4.15 – 4.05 (m, 2H), 4.02 – 3.96 (m, 2H), 3.85 – 3.72 (m, 3H), 3.31 – 3.16 (m, 2H), 2.66 (brs, 1H), 2.52 – 2.38 (m, 1H), 2.00 – 3.19 (m, 2H), 2.48 – 2.40 (m, 1H), 2.00 – 1.96 (m, 3H), 1.71 – 1.68 (m, 3H), 1.65 – 1.61 (m, 3H), 1.51 – 1.43 (m, 7H), 1.17 – 1.12 (m, 1H). **$^{13}$C NMR** (201 MHz, $CDCl_3$) $\delta$ 151.3, 142.4, 128.7 (q, $^1J_{CF}$ = 279.6 Hz), 116.1, 114.5, 70.6, 70.3, 63.9, 45.9, 42.3, 40.7, 37.1 (q, $^2J_{CF}$ = 24.5 Hz), 37.0, 32.5, 28.6. **$^{19}$F NMR** (376 MHz, $CDCl_3$) $\delta$ -69.21 (s, 3F). **HRMS** (ESI) m/z: $[M+H]^+$ Calcd for $C_{23}H_{33}F_3NO_3^+$ 428.2413; Found 428.2416.

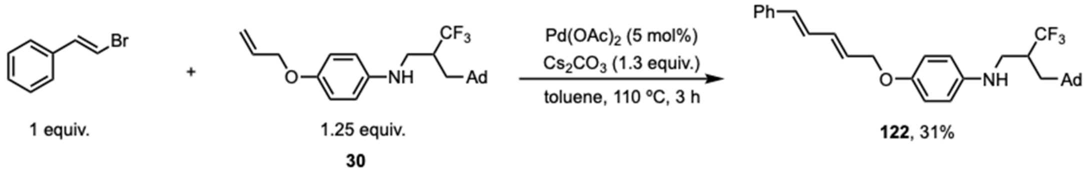


An oven-dried, transparent 10 mL Teflon screw-capped Schlenk tube equipped with a stir bar was sequentially charged with **30** (98.4 mg, 1.25 equiv.), vinyl bromide (0.2 mmol, 26 $\mu$L, 1.0 equiv.), $Cs_2CO_3$ (84.7 mg, 1.3 equiv.), and $Pd(OAc)_2$ (2.3 mg, 5 mol%). The tube was evacuated *in vacuo* and then backfilled with argon for three times. Dry toluene (4 mL, 0.05M) was transferred into the tube via a syringe. The resulting mixture was stirred under an argon atmosphere was stirred at 110 °C for 3 h. Upon completion of the reactions (TLC showed complete consumption of starting material). Then the mixture was distilled with water (10 mL), and extracted with EtOAc (3 × 10 mL). The combined organic layers were washed with brine, dried over Na2SO4, filtered and concentrated in *vacuo*. The residue was purified by preparative thin-layer chromatography using a mixture of petroleum ether and ethyl acetate (PE:EA = 12:1) as an eluent to afford the product **122**.

***N*-(2-((adamantan-1-yl)methyl)-3,3,3-trifluoropropyl)-4-(((2*E*,4*E*)-5-phenylpenta-2,4-dien-1-yl)oxy)aniline (122).** The title compound was obtained as a white amorphous solid by preparative TLC using petroleum ether/EtOAc (12:1) as an eluent; $R_f$= 0.3 (petroleum ether/EtOAc = 10:1); 30.7 mg, 31% yield. **$^1$H NMR** (400 MHz, $CDCl_3$) $\delta$ 7.41 – 7.30 (m, 4H), 7.25 – 7.21 (m, 1H), 6.84 – 6.78 (m, 3H), 6.60 – 6.48 (m, 4H), 6.04 – 5.97 (m, 1H), 4.56 (d, $J$ = 5.9 Hz, 2H), 3.64 (brs, 1H), 3.31 – 3.16 (m, 2H), 2.49 – 2.38 (m, 1H), 1.98 – 1.94 (m, 3H), 1.71 – 1.65 (m, 3H), 1.61 – 1.58 (m, 3H), 1.52 – 1.43 (m, 7H), 1.18 – 1.13 (m, 1H). **$^{13}$C NMR** (201 MHz, $CDCl_3$) $\delta$ 151.6, 142.0, 137.2, 133.3, 133.2, 129.0, 128.8, 128.7 (q, $^1J_{CF}$ = 279.9 Hz), 128.2, 127.8, 126.6, 116.3, 114.6, 69.3, 46.0, 42.3, 40.7, 37.2 (q, $^2J_{CF}$ = 24.2 Hz), 37.0, 32.5, 28.6. **$^{19}$F NMR** (376 MHz, $CDCl_3$) $\delta$ -69.21 (s, 3F). **HRMS** (ESI) m/z: $[M+H]^+$ Calcd for $C_{31}H_{37}F_3NO^+$ 496.2827; Found 496.2829.

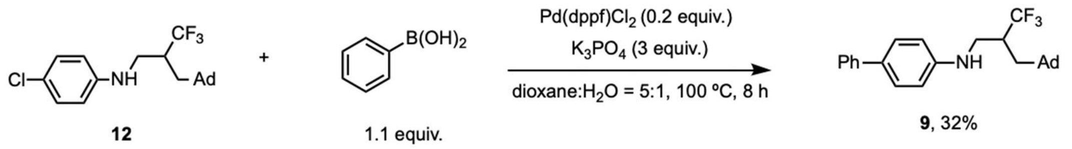


An oven-dried, transparent 10 mL Teflon screw-capped Schlenk tube equipped with a stir bar was sequentially charged with **12** (74.4 mg, 0.2 mmol, 1.0 equiv.), Phenylboronic acid (26.8 mg, 1.1 equiv.), $K_3PO_4$ (127.2 mg, 3.0 equiv.), and $Pd(dppf)Cl_2$ (29.3 mg, 0.2 equiv.). The tube was evacuated *in vacuo* and then backfilled with argon for three times. Dioxane (1.0 mL) and $H_2O$ (0.2 mL) were transferred into the tube via a syringe. The resulting mixture was stirred under an argon atmosphere was stirred at 100 °C for 8 h. Upon completion of the reactions (TLC showed complete consumption of starting material). Then the mixture was distilled with water (10 mL) and extracted with EtOAc (3 × 10 mL). The combined organic layers were washed with brine, dried over Na2SO4, filtered and concentrated in *vacuo*. The residue was purified by preparative thin-layer chromatography using a mixture of petroleum ether and ethyl acetate (PE:EA = 15:1) as an eluent to afford the product **9**. The spectroscopic analysis of compound **9** obtained in this reaction was in line with the identical compound obtained in scope study.

## Stern-Volmer quenching studies

Stern-Volmer quenching experiments were carried using a solution of photocatalyst [Ir(dF($CF_3$)ppy)$_2$(dtbbpy)]$PF_6$ (**PC1**, 0.010 mM) and variable concentrations (0.005, 0.010, 0.015, 0.020, 0.025 mM) of Hantzsch ester (**HE**), *N*,*N*-dimethylcyclohexylamine (**CyNMe$_2$**, **A1**), 1-(benzyloxy)-4-nitrobenzene (***p*-BnOPhNO$_2$**, **N14**), redox active ester (**AdCO$_2$NPhth**, **R1**), and **Ni($NO_3$)$_2$•6$H_2O$/BPhen** (**L1**, in 1:1 molar ratio) in DMSO/DME (v/v = 2:1). The standard solutions of samples were prepared in the nitrogen-filled glovebox. 3 mL of sample solutions were transferred via syringe into the PTFE-stopped, parafilm-sealed quartz cuvettes (3.5 mL, 10 mm) under the positive argon pressure prior to the measurement. The intensity of the emission peak at 484 nm ($\lambda_{ex}$ = 369 nm), which was expressed as the ratio $I_0/I$ ($I_0$: emission intensity of photocatalyst **PC1** at 484 nm in the absence of quencher; I: observed intensity) as a function of the quencher concentration, was measured. The Stern-Volmer plots for each component are provided in the **Figs. S11–S16**. The results suggested that Hantzsch ester (**HE**), **CyNMe$_2$** (**A1**), 1-(benzyloxy)-4-nitrobenzene (***p*-BnOPhNO$_2$**, **N14**), **AdCO$_2$NPhth** (**R1**), and **Ni($NO_3$)$_2$•6$H_2O$/BPhen** were all able to quench the photoexcited **PC1**. Among them, 1-(benzyloxy)-4-nitrobenzene (***p*-BnOPhNO$_2$**, **N14**) is the most effective quencher to initiate the subsequent photocatalytic events.

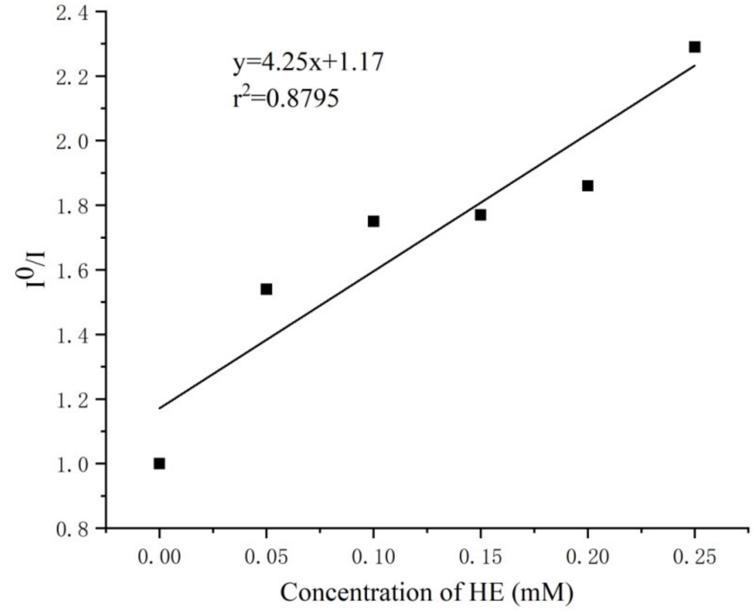


**Fig. S11.** Stern-Volmer fluorescence quenching studies of photocatalyst **PC1** (0.01 mM) with varying concentrations of Hantzsch ester (**HE**).

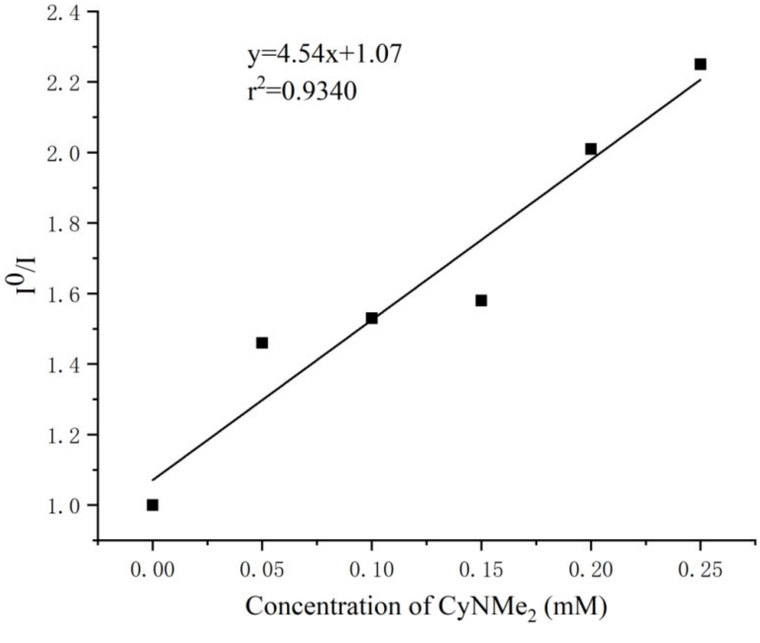


**Fig. S12.** Stern-Volmer fluorescence quenching studies of photocatalyst **PC1** (0.01 mM) with varying concentrations of *N*,*N*-dimethylcyclohexylamine (**$CyNMe_2$**, **A1**).

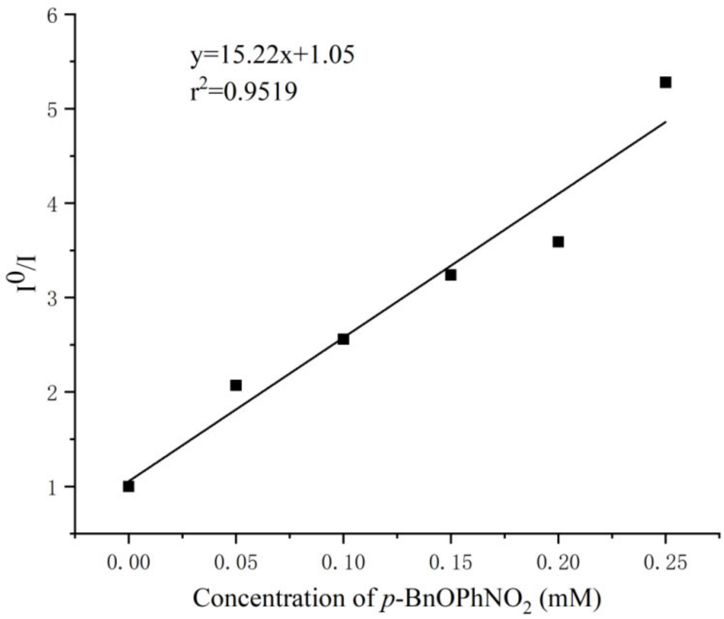


**Fig. S13.** Stern-Volmer fluorescence quenching studies of photocatalyst **PC1** (0.01 mM) with varying concentrations of 1-(benzyloxy)-4-nitrobenzene (***p*-BnOPhNO$_2$**, **N14**).

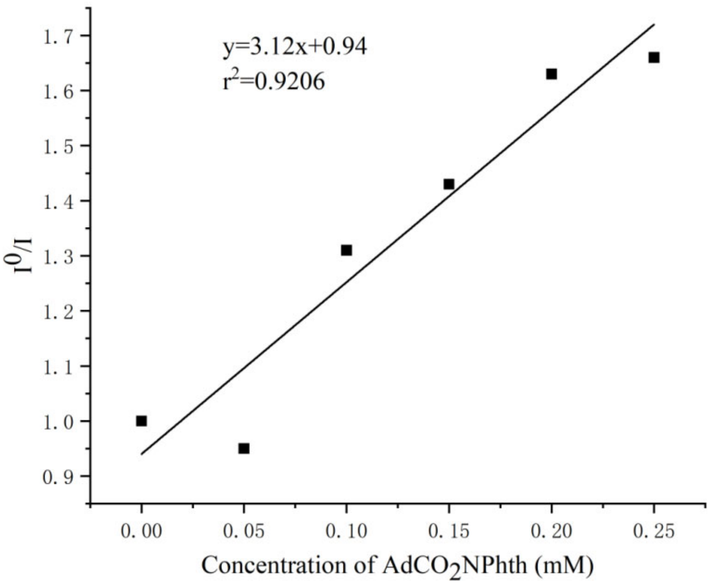


**Fig. S14.** Stern-Volmer fluorescence quenching studies of photocatalyst **PC1** (0.01 mM) with varying concentrations of redox active ester (**AdCO$_2$NPhth**, **R1**).

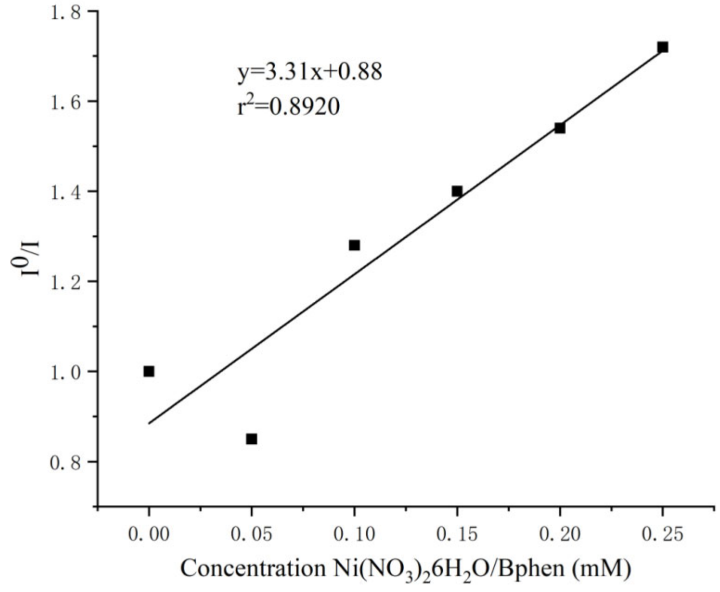


**Fig. S15.** Stern-Volmer fluorescence quenching studies of photocatalyst **PC1** (0.01 mM) with varying concentrations of **Ni(NO$_3$)$_2$•6H$_2$O/BPhen (L1)**.

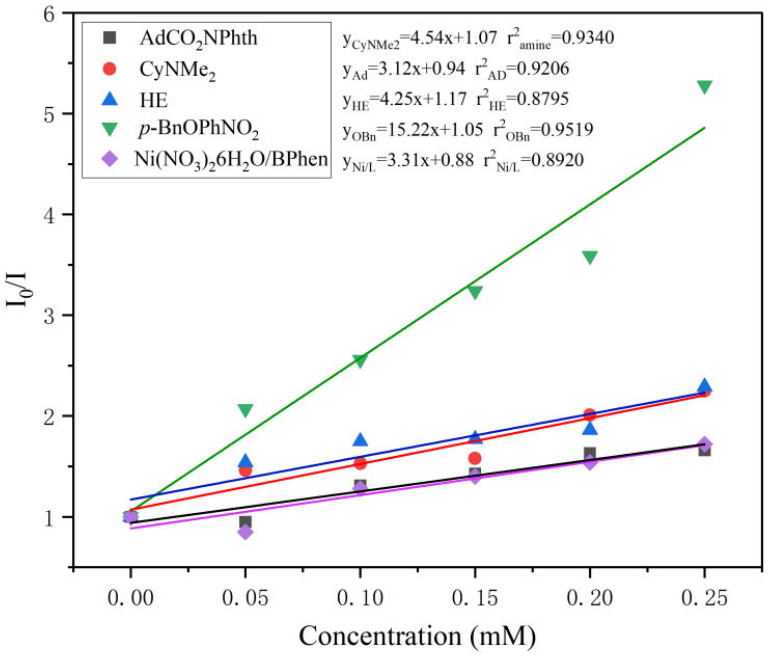


**Fig. S16** Stern-Volmer fluorescence quenching studies of photocatalyst **PC1** (0.01 mM) with varying concentrations of redox active ester (**AdCO$_2$NPhth**, **R1**), *N*,*N*-dimethylcyclohexylamine (**CyNMe$_2$** , **A1**), Hantzsch ester (**HE**), **Ni(NO$_3$)$_2$•6H$_2$O/BPhen (L1)**, and 1-(benzyloxy)-4-nitrobenzene (***p*-BnOPhNO$_2$**, **N14**).

# X-ray crystallographic analysis

The single crystal of *N*-alkyl aniline product **14** was obtained by slow evaporation of the solvent system based on $CH_2Cl_2$ and cyclohexane. The ORTEP representation with 50% probability thermal ellipsoids were presented. A disordered X-ray structure was obtained. Crystallographic data for the compound **14** has been deposited at the Cambridge Crystallographic Data Centre, under deposition numbers CCDC 2350147 (**14**). Copies of the data can be obtained free of charge via https://www.ccdc.cam.ac.uk/structures/.

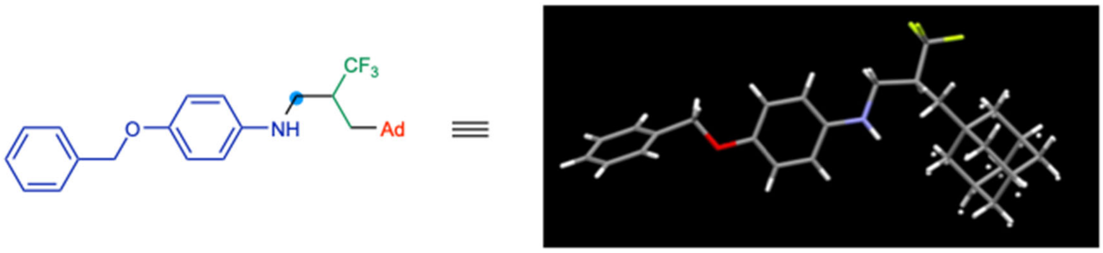


| | |
|---|---|
| Empirical formula of **14** | $C_{27}H_{32}F_3NO$ |
| CCDC number | 2350147 |
| Formula weight | 443.53 |
| Temperature | 150.00 K |
| Crystal system, space group | triclinic, P-1 |
| Unit cell dimensions | a = 6.58510(10) Å $\alpha$ = 93.369(2) °. |
| | b = 9.7090(2) Å $\beta$ = 99.9900(10) °. |
| | c = 18.7404(3) Å $\gamma$ = 101.8690(10) °. |
| Volume/Å3 | 1149.21(4) |
| Z, $\rho_{calc}$ / g/cm$^3$ | 2, 1.282 |
| $\mu$ / mm$^{-1}$ | 0.769 |
| F (000) | 427.0 |
| Crystal size / mm$^3$ | 0.2 × 0.15 × 0.1 |

| | |
|---|---|
| Radiation | CuK$\alpha$($\lambda$ = 1.54178) |
| 2θ range for data collection / ° | 4.81 to 150.266 |
| Index ranges | $-7 \leq h \leq 8$, $-12 \leq k \leq 12$, $-23 \leq l \leq 23$ |
| Reflections collected | 17282, |
| Independent reflections | 4686 [$R_{int}$ = 0.0592, $R_{sigma}$ = 0.0571] |
| Data / restraints / parameters | 4686/0/389 |
| Goodness-of-fit on $F^2$ | 1.089 |
| Final R indexes [I>2σ (I)] | $R_1$ = 0.0695, $wR_2$ = 0.1861 |
| Final R indexes [all data] | $R_1$ = 0.0849, $wR_2$ = 0.2006 |
| Largest diff. Peak / hole / e $Å^{-3}$ | 0.48/-0.52 |

## NMR Spectra

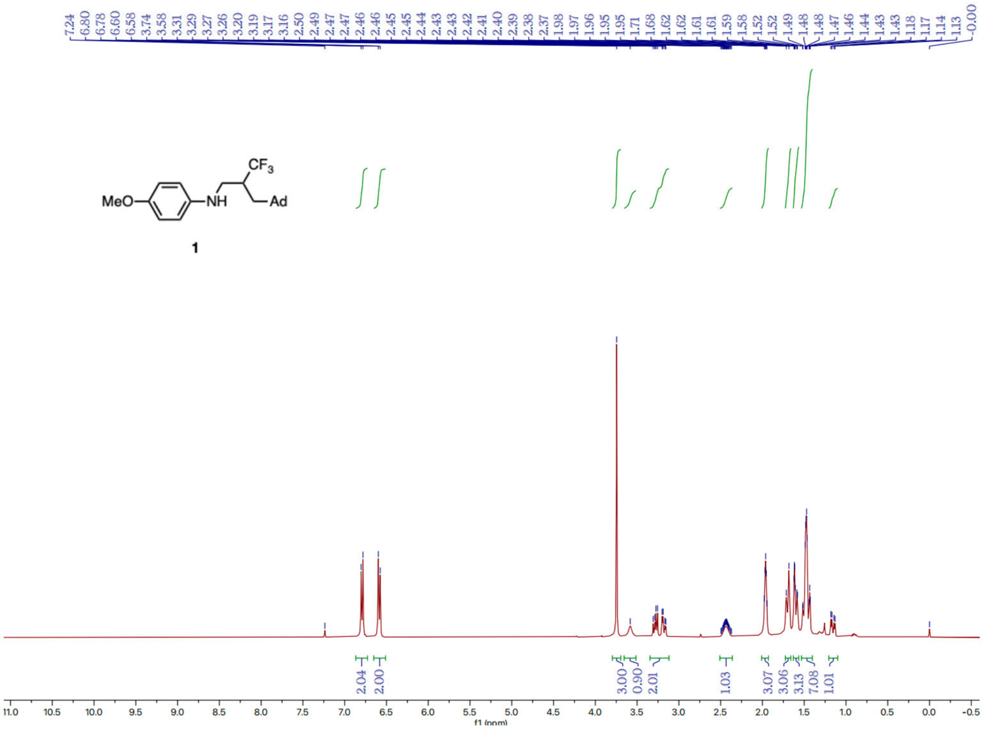

$^{1}$H NMR spectrum (400 MHz, Chloroform-*d*) of compound **1**

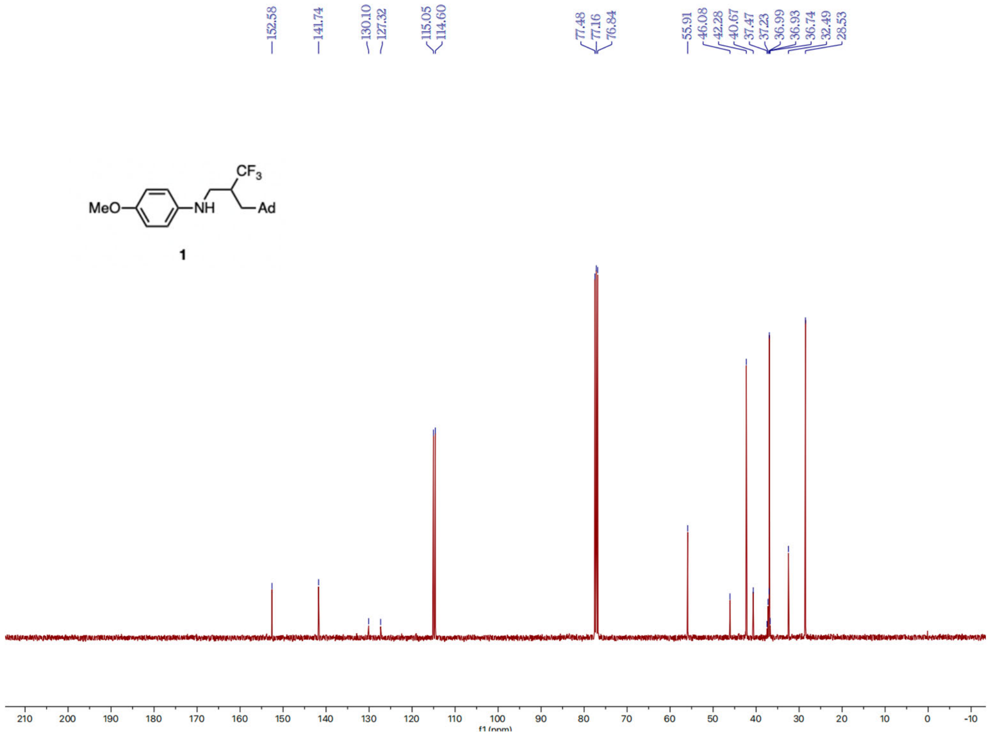

$^{13}$C NMR spectrum (101 MHz, Chloroform-*d*) of compound **1**

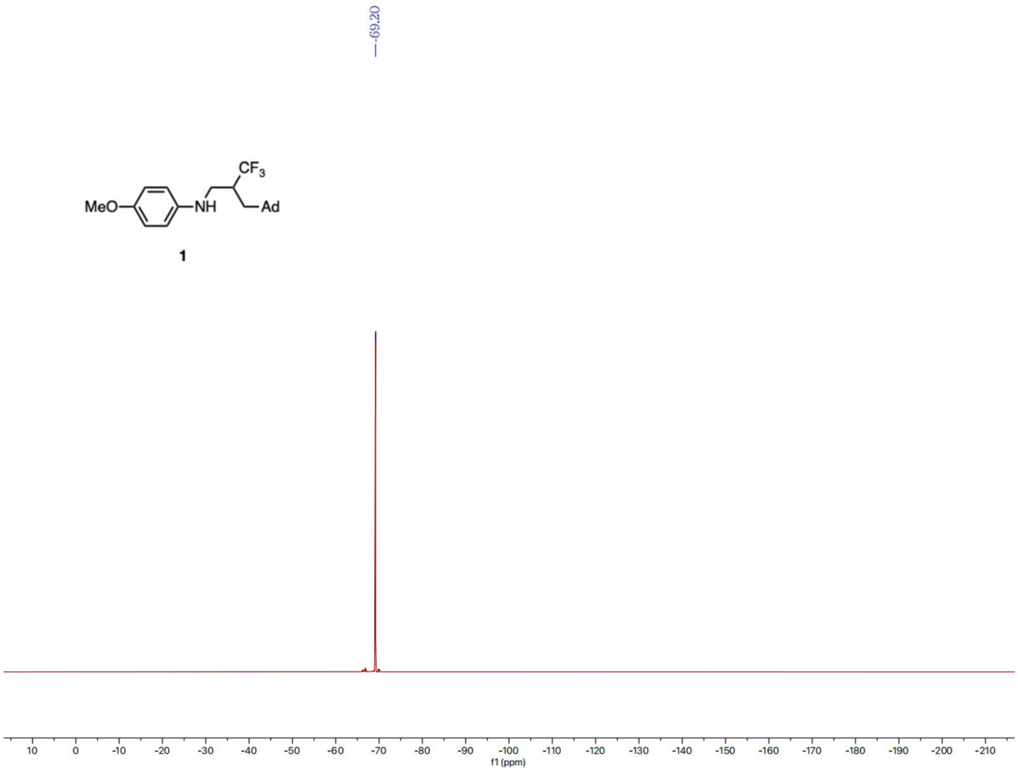


$^{19}F$ NMR spectrum (376 MHz, Chloroform-*d*) of compound **1**

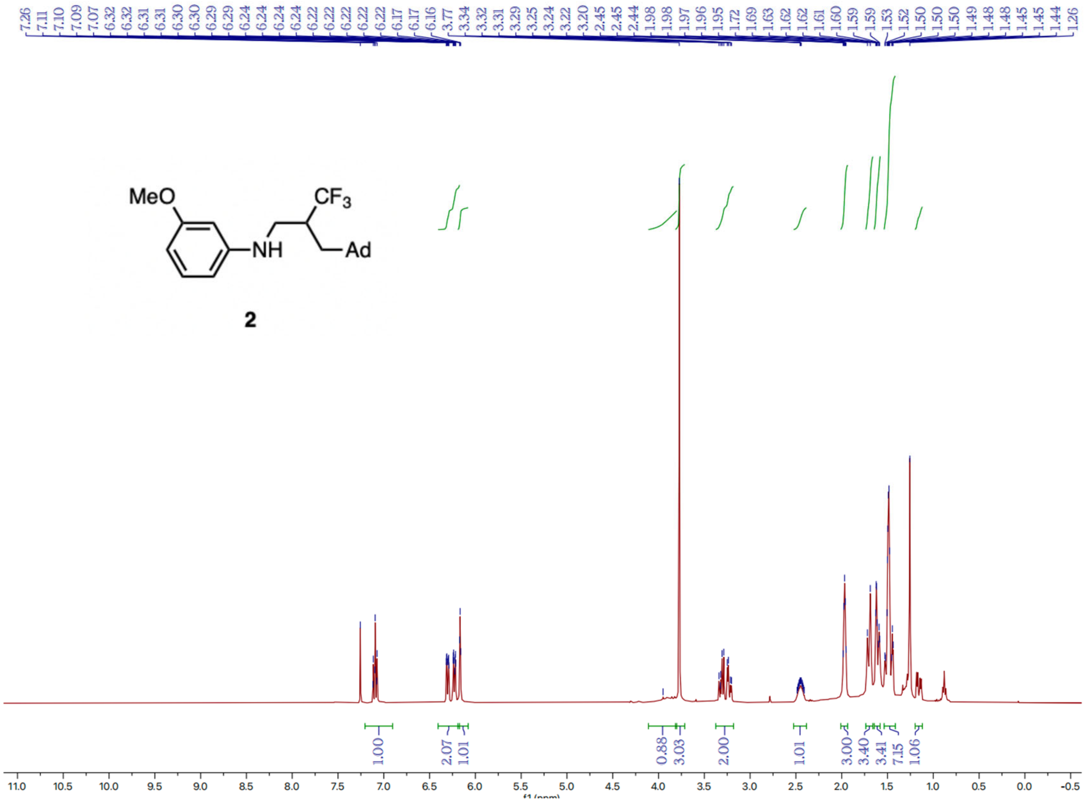

$^{1}H$ NMR spectrum (400 MHz, Chloroform-*d*) of compound **2**

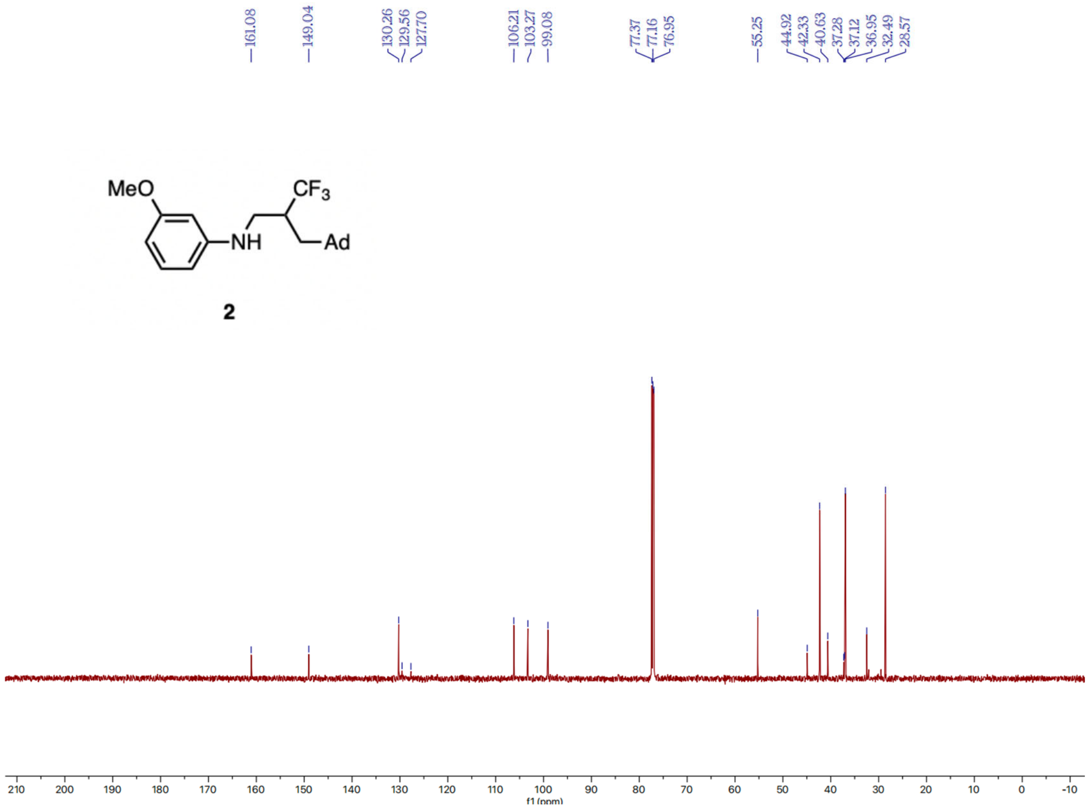

$^{13}C$ NMR spectrum (151 MHz, Chloroform-*d*) of compound **2**

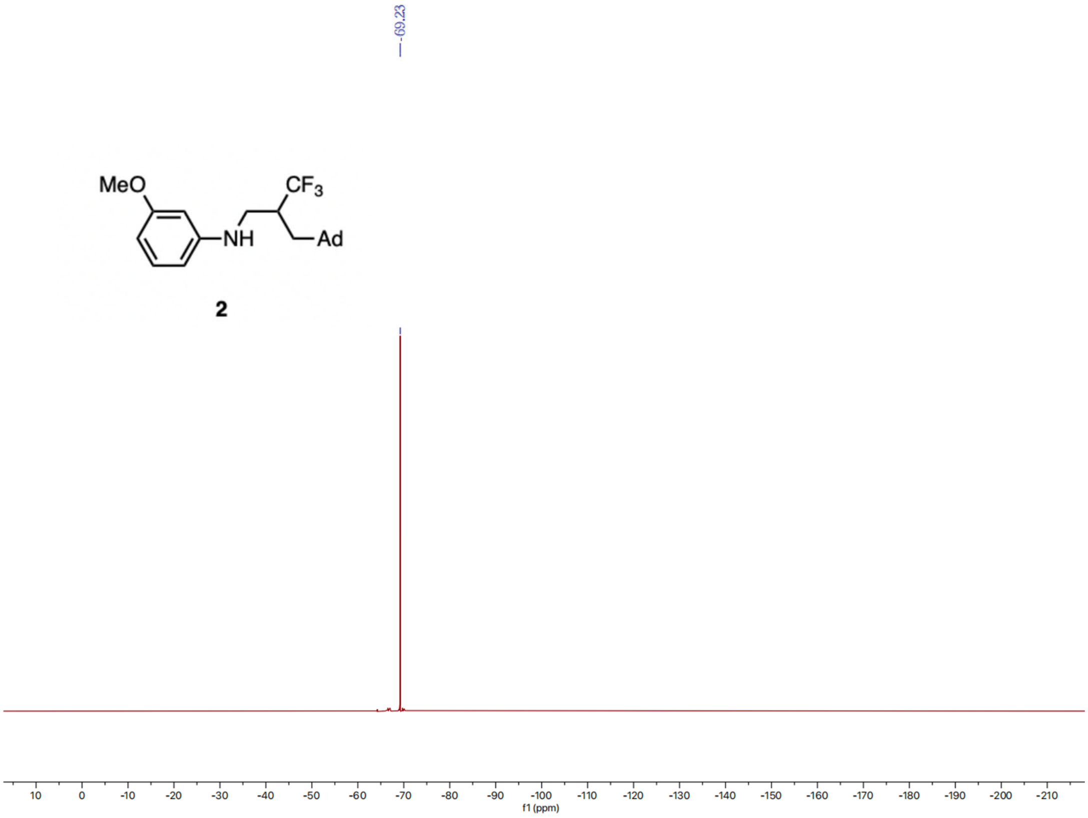


$^{19}$F NMR spectrum (376 MHz, Chloroform-*d*) of compound **2**

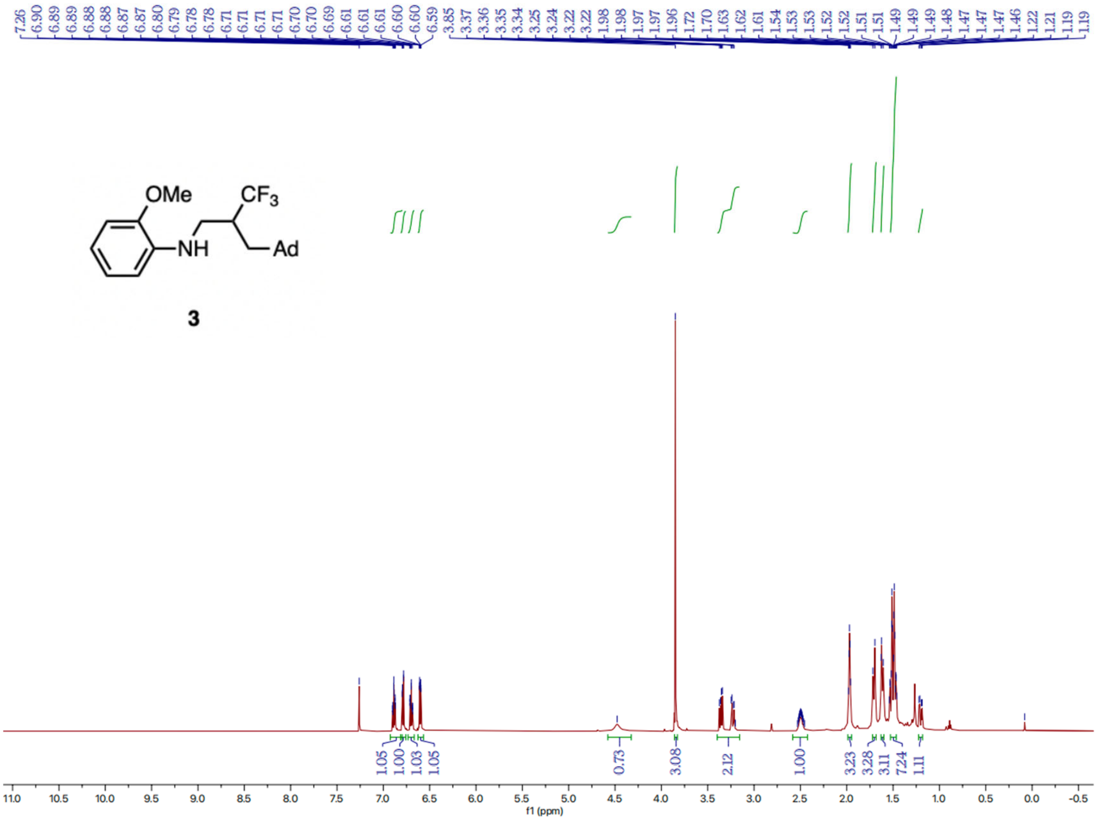

$^1$H NMR spectrum (600 MHz, Chloroform-*d*) of compound **3**

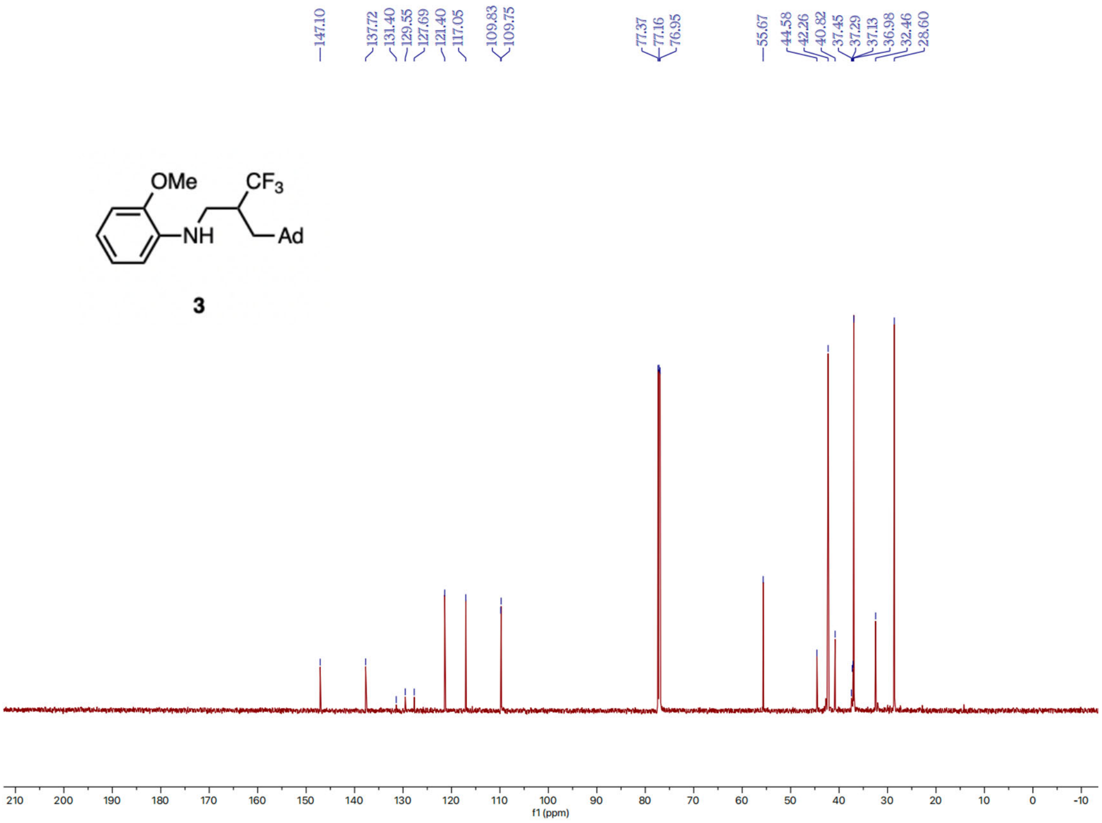

$^{13}$C NMR spectrum (151 MHz, Chloroform-*d*) of compound **3**

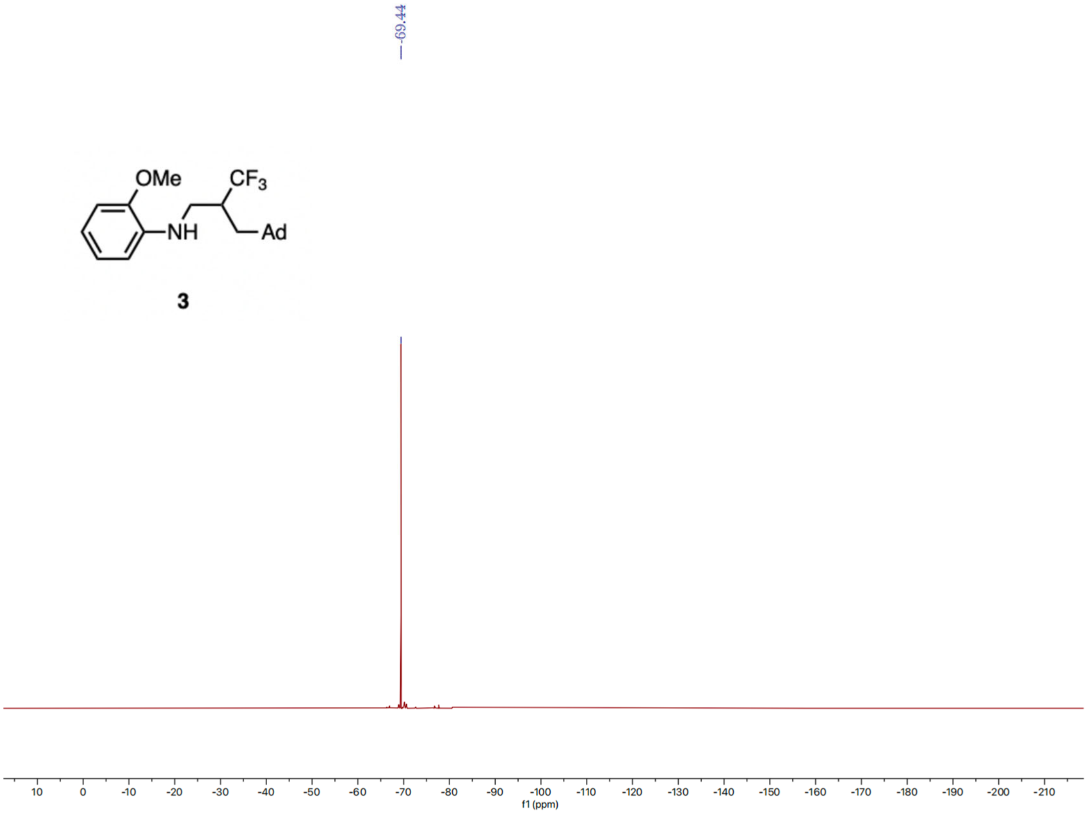


$^{19}F$ NMR spectrum (376 MHz, Chloroform-*d*) of compound **3**

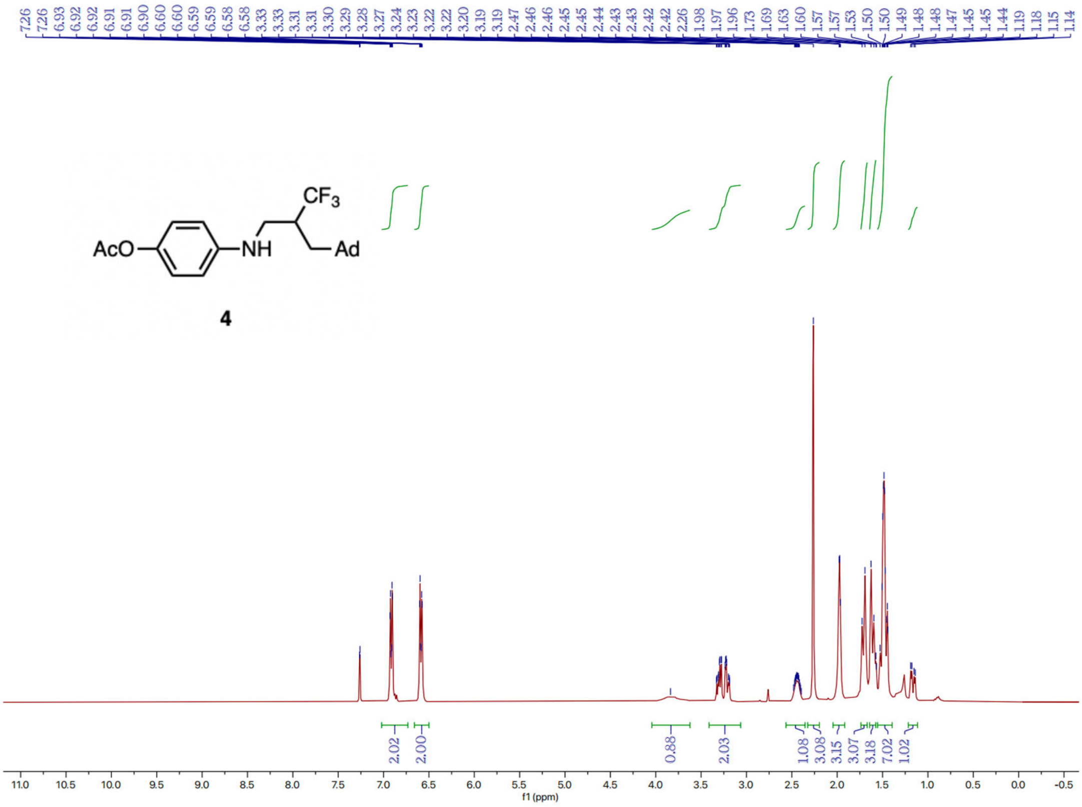

$^{1}$H NMR spectrum (400 MHz, Chloroform-*d*) of compound **4**

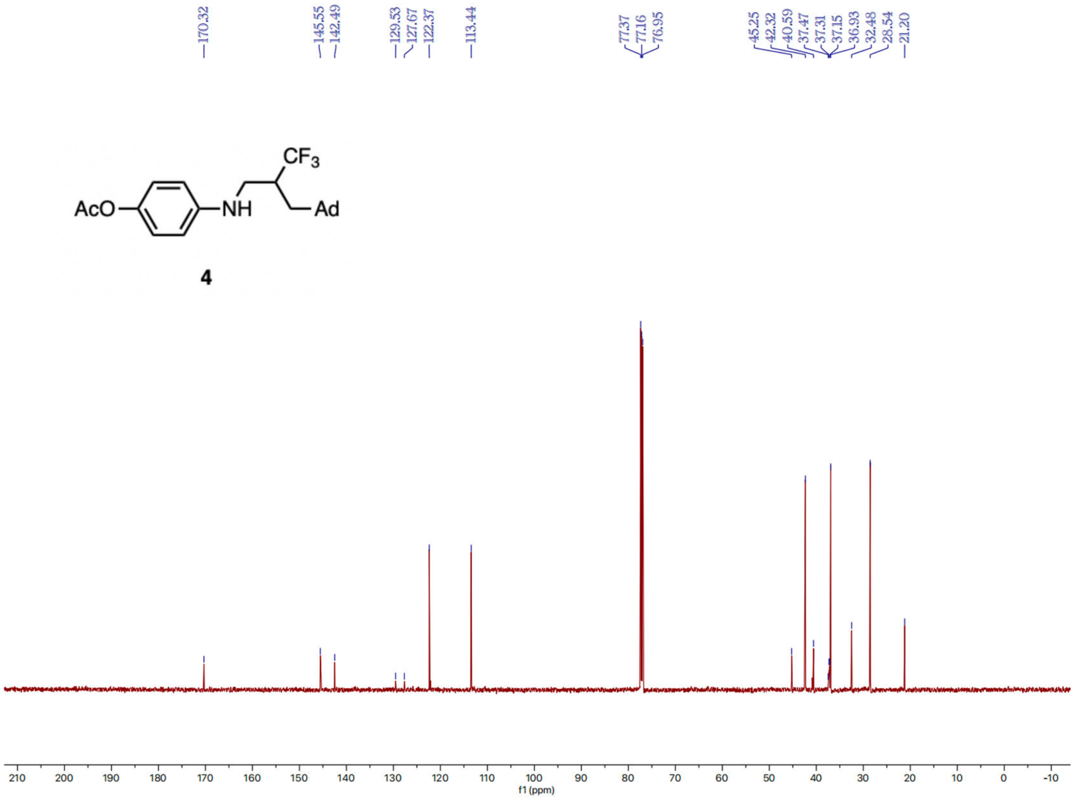

$^{13}$C NMR spectrum (151 MHz, Chloroform-*d*) of compound 4

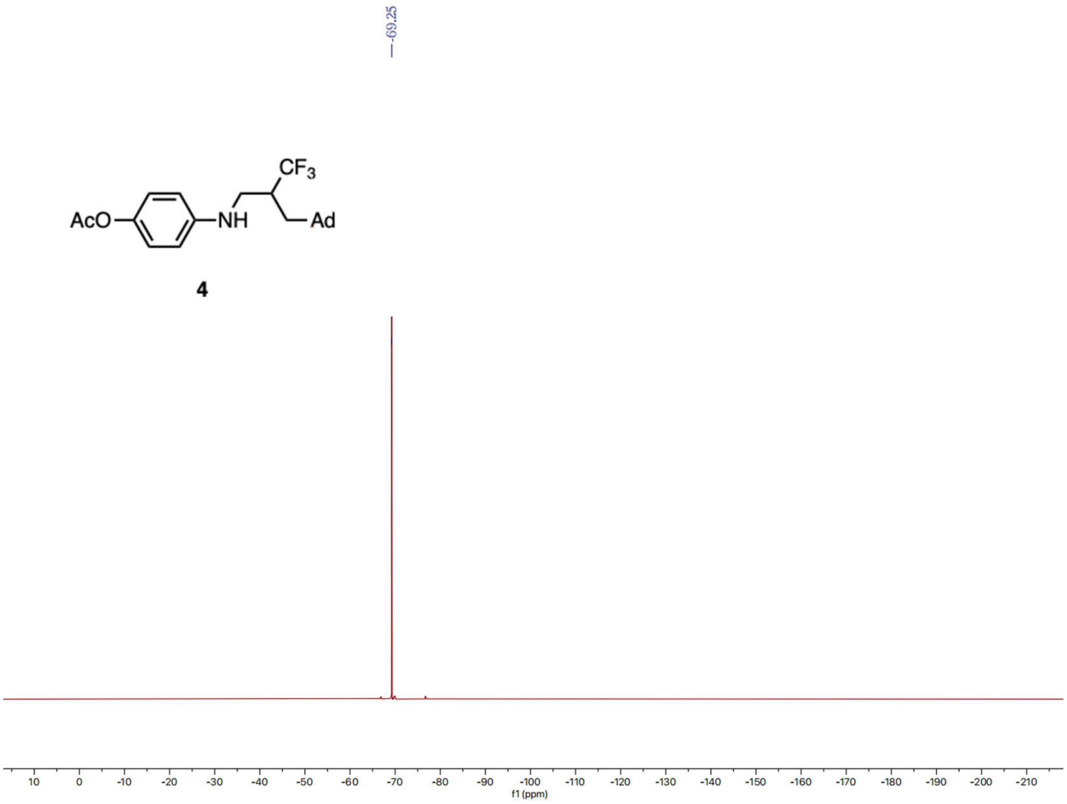


$^{19}$F NMR spectrum (376 MHz, Chloroform-*d*) of compound **4**

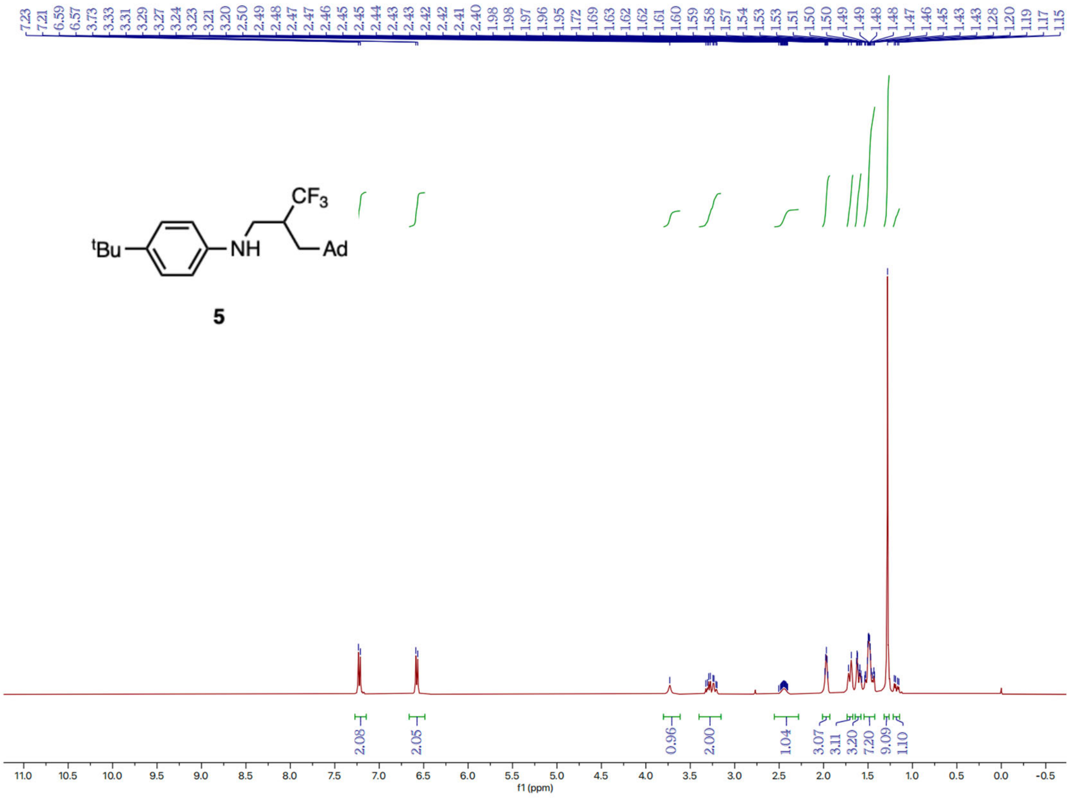

$^{1}$H NMR spectrum (400 MHz, Chloroform-*d*) of compound **5**

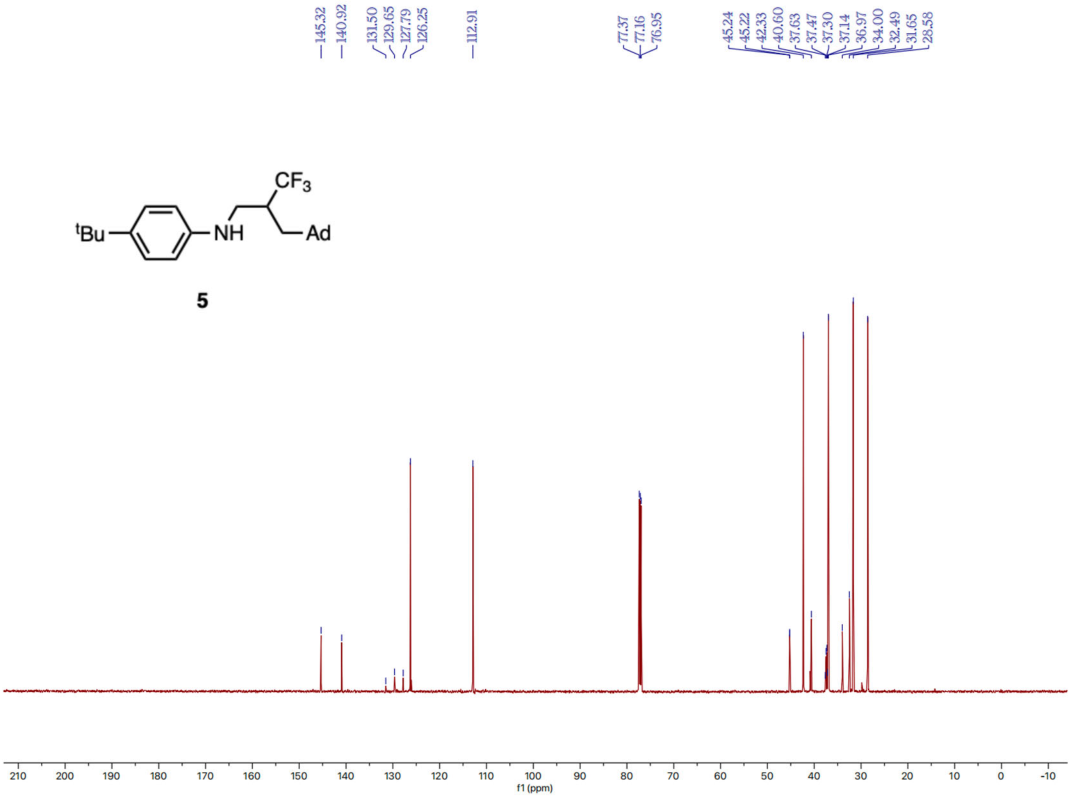

$^{13}$C NMR spectrum (151 MHz, Chloroform-*d*) of compound **5**

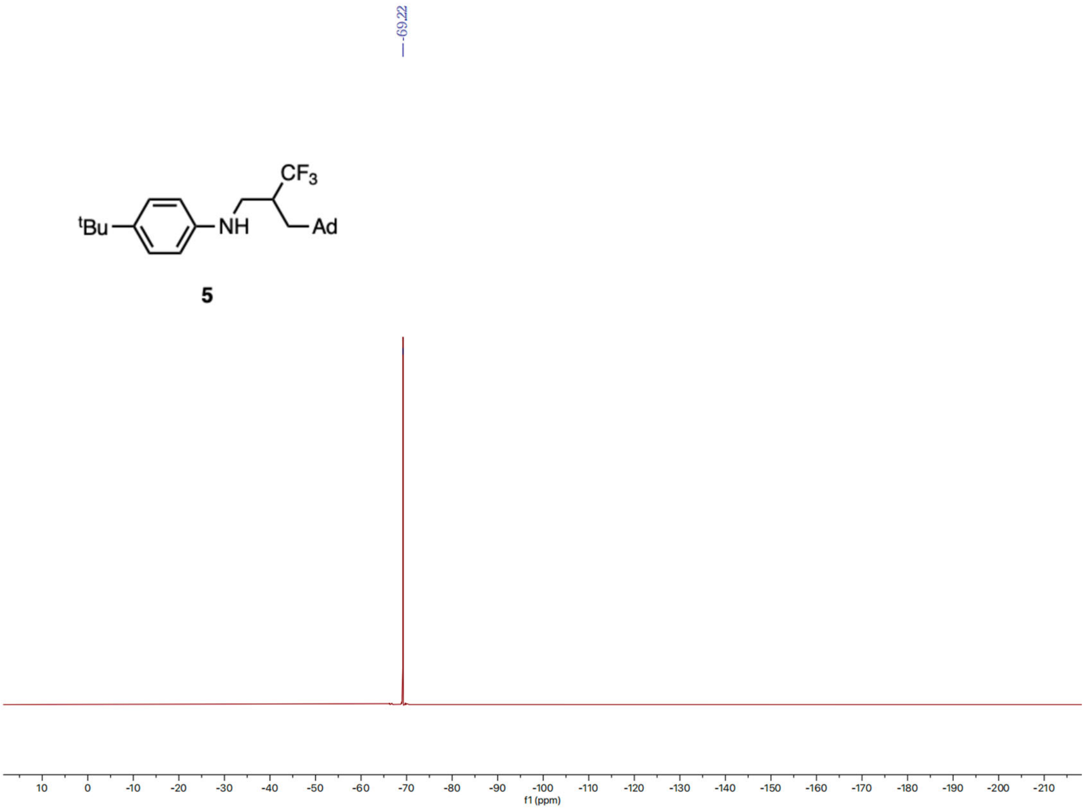


$^{19}$F NMR spectrum (376 MHz, Chloroform-*d*) of compound **5**

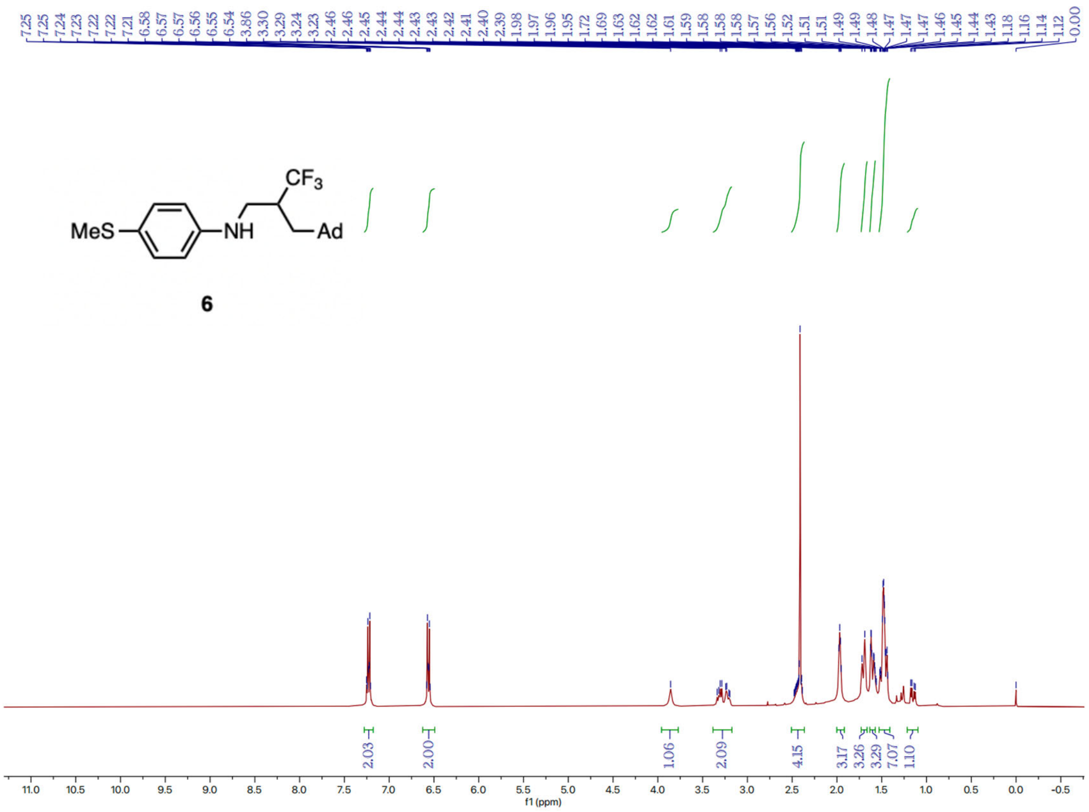

$^{1}H$ NMR spectrum (400 MHz, Chloroform-*d*) of compound **6**

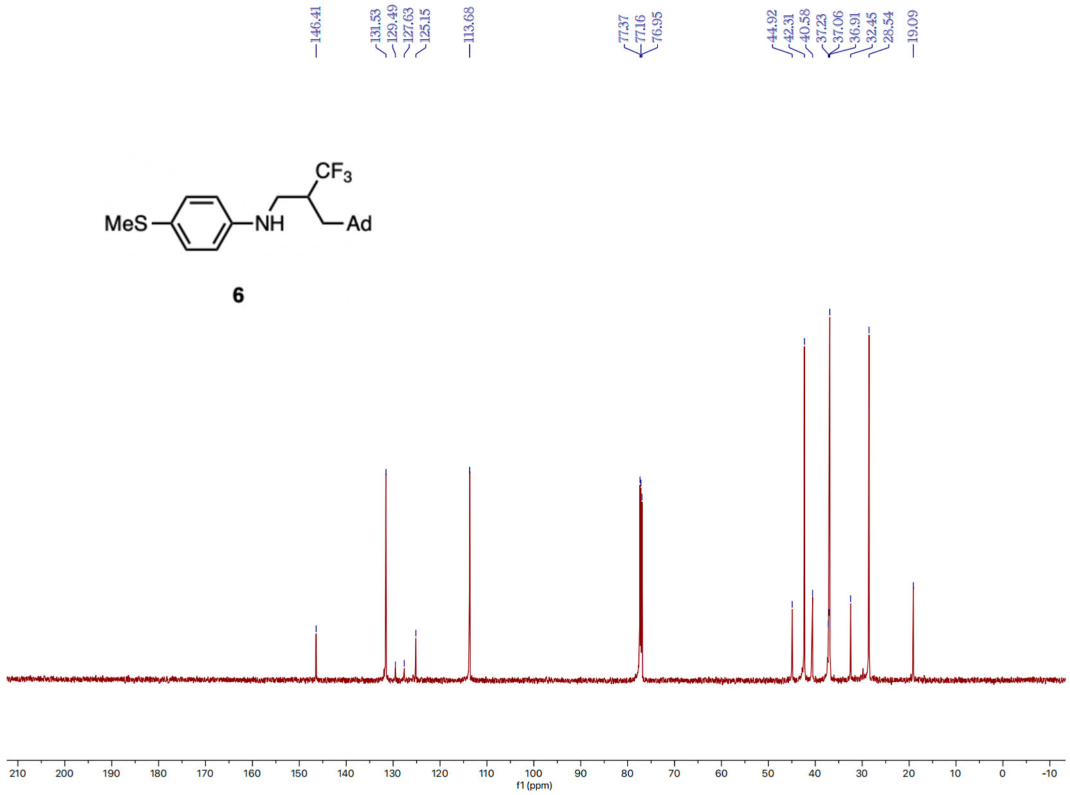

$^{13}C$ NMR spectrum (151 MHz, Chloroform-*d*) of compound **6**

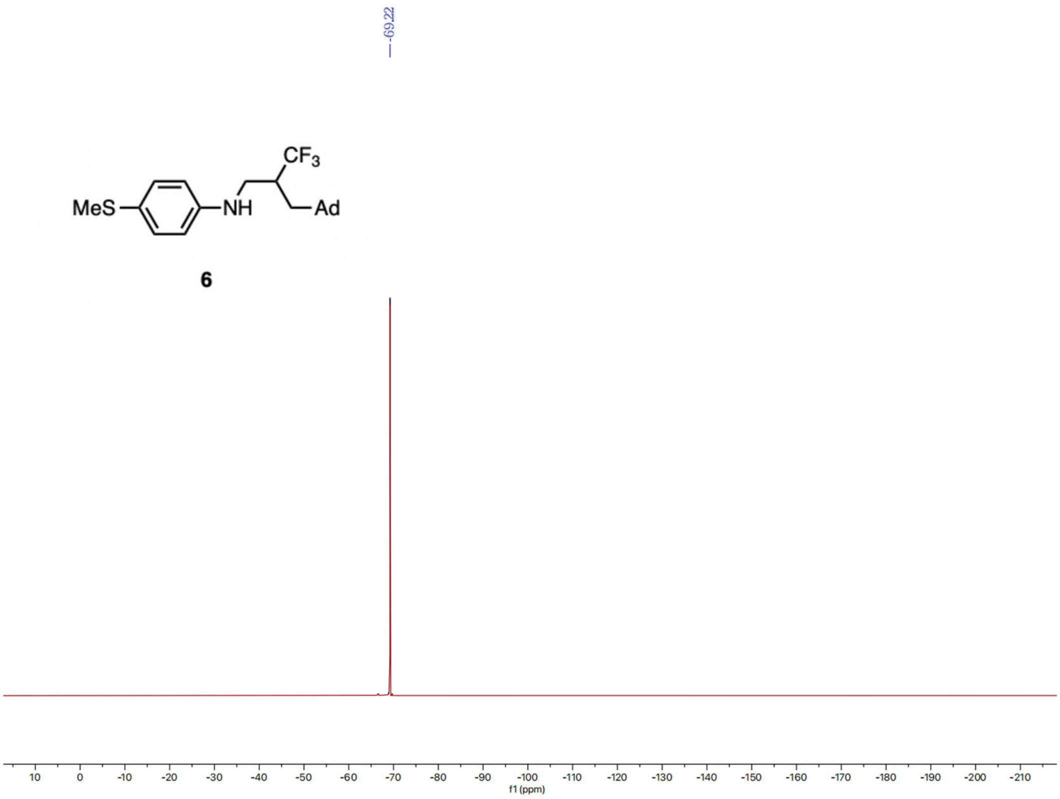


$^{19}F$ NMR spectrum (376 MHz, Chloroform-*d*) of compound **6**

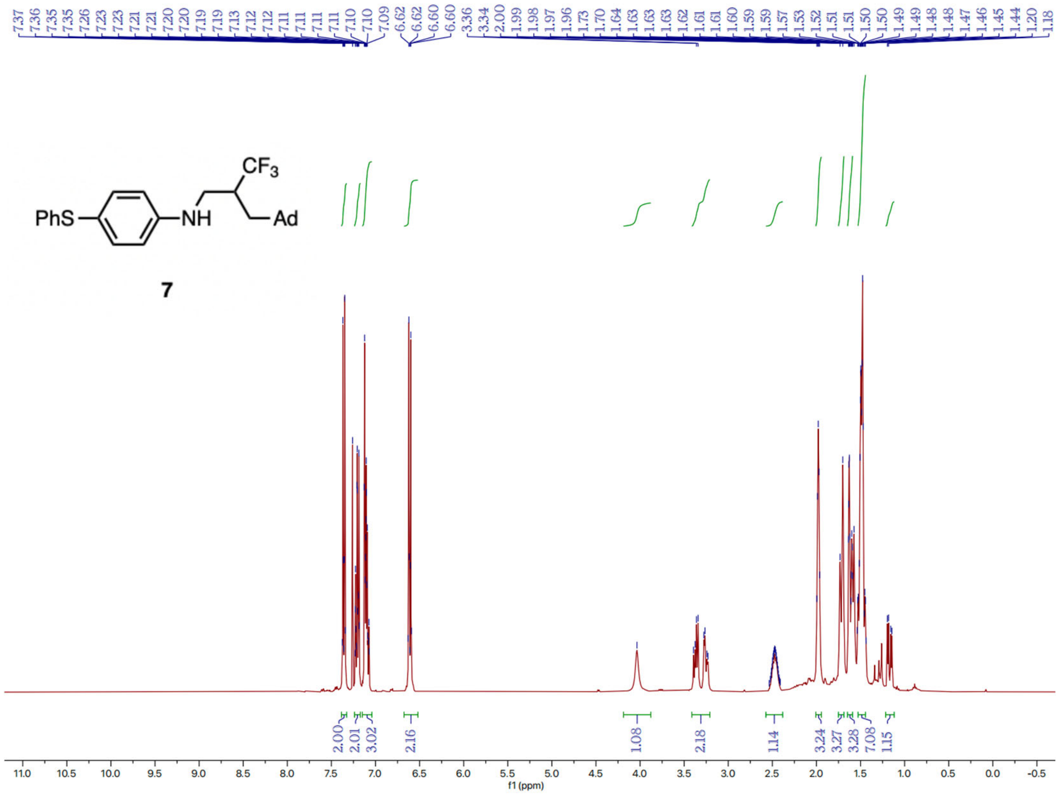

$^{1}$H NMR spectrum (400 MHz, Chloroform-*d*) of compound **7**

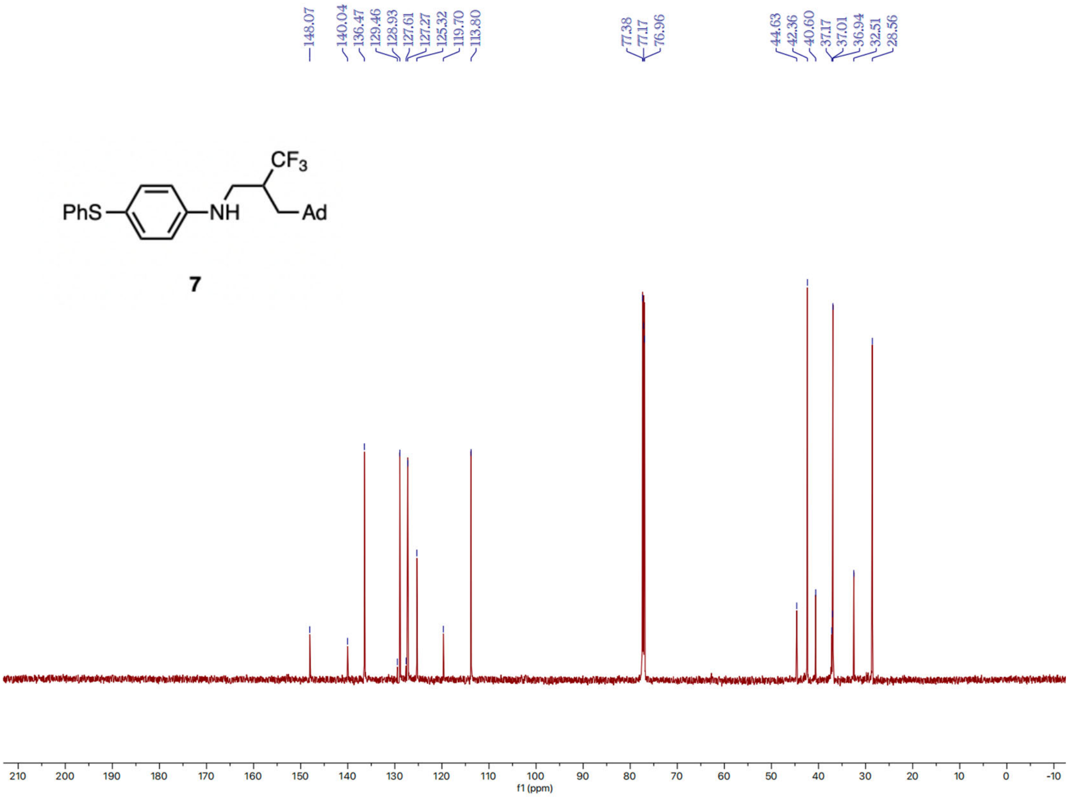

$^{13}$C NMR spectrum (151 MHz, Chloroform-*d*) of compound **7**

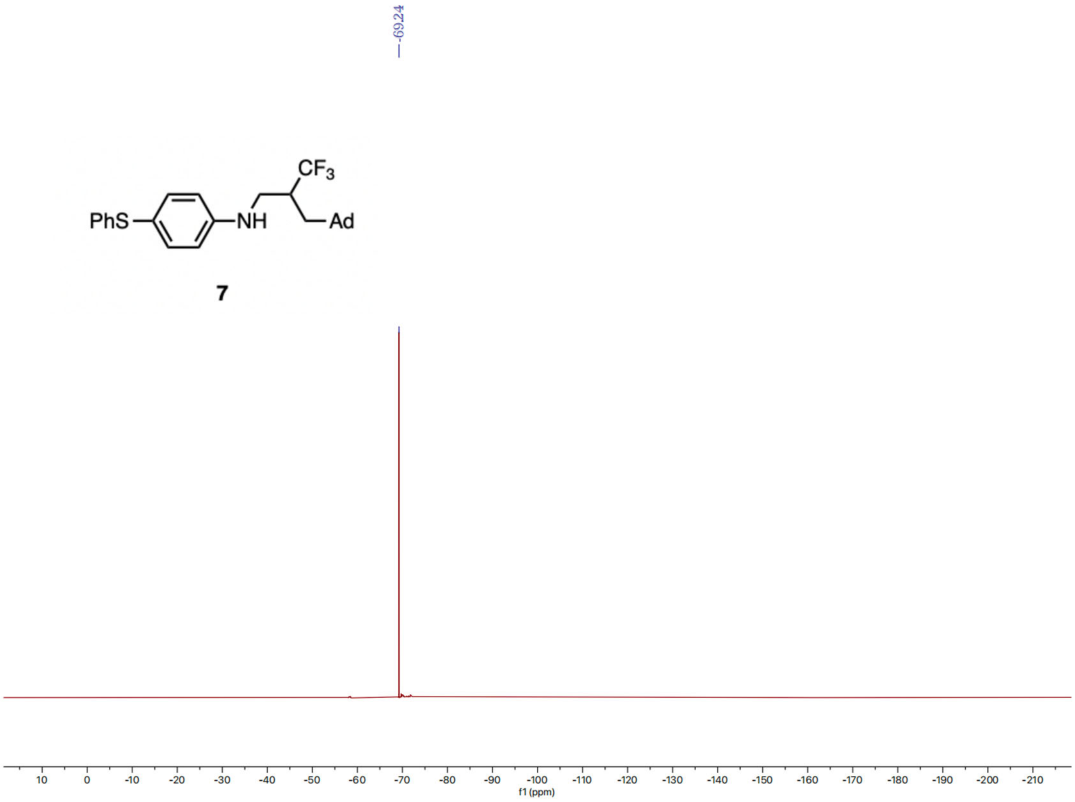


$^{19}$F NMR spectrum (376 MHz, Chloroform-*d*) of compound **7**

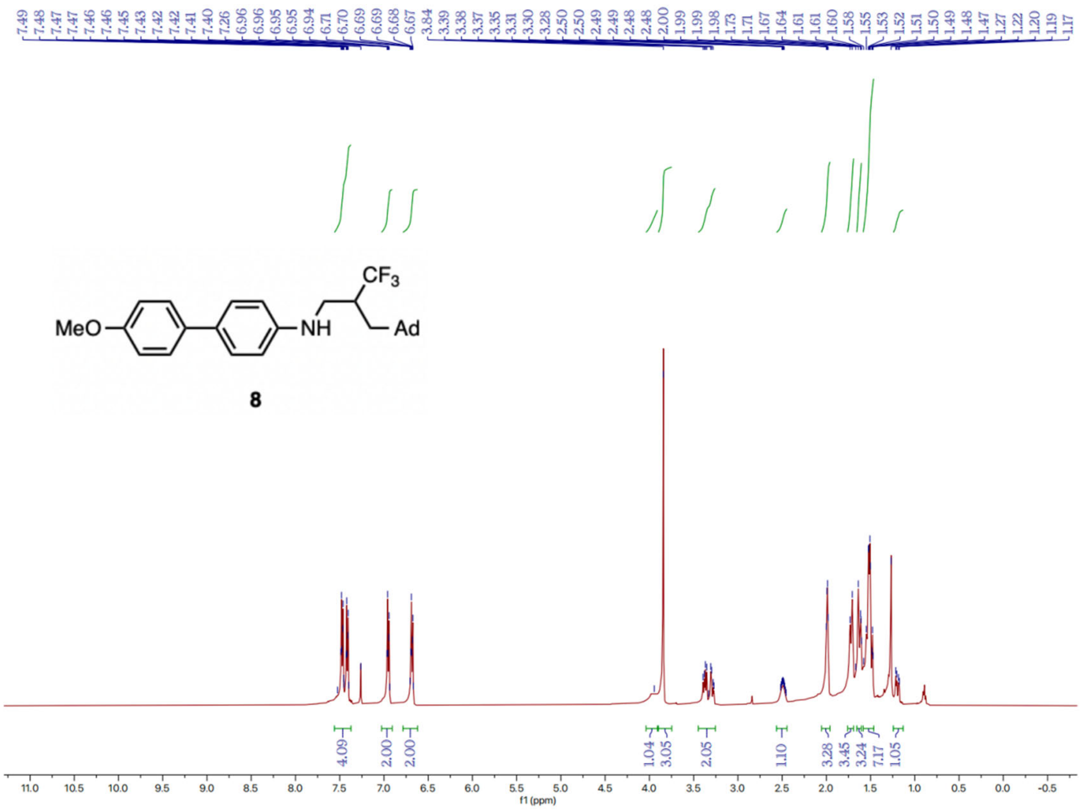

$^{1}$H NMR spectrum (400 MHz, Chloroform-*d*) of compound **8**

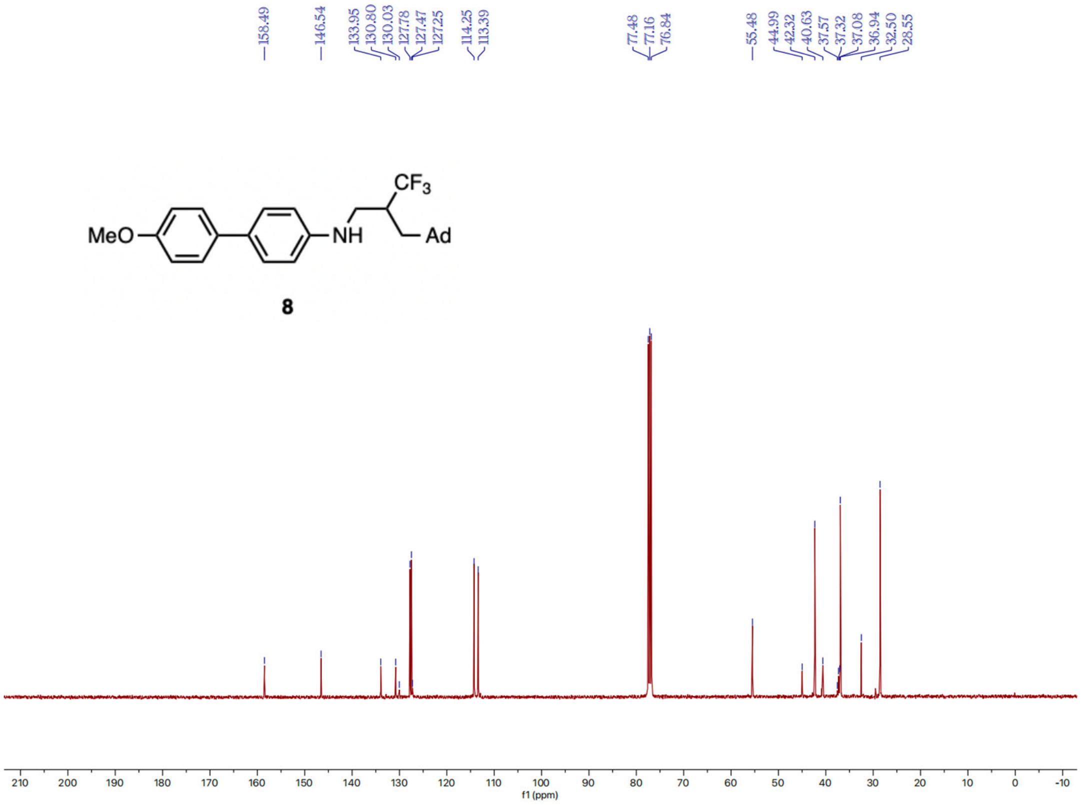

$^{13}$C NMR spectrum (101 MHz, Chloroform-*d*) of compound **8**

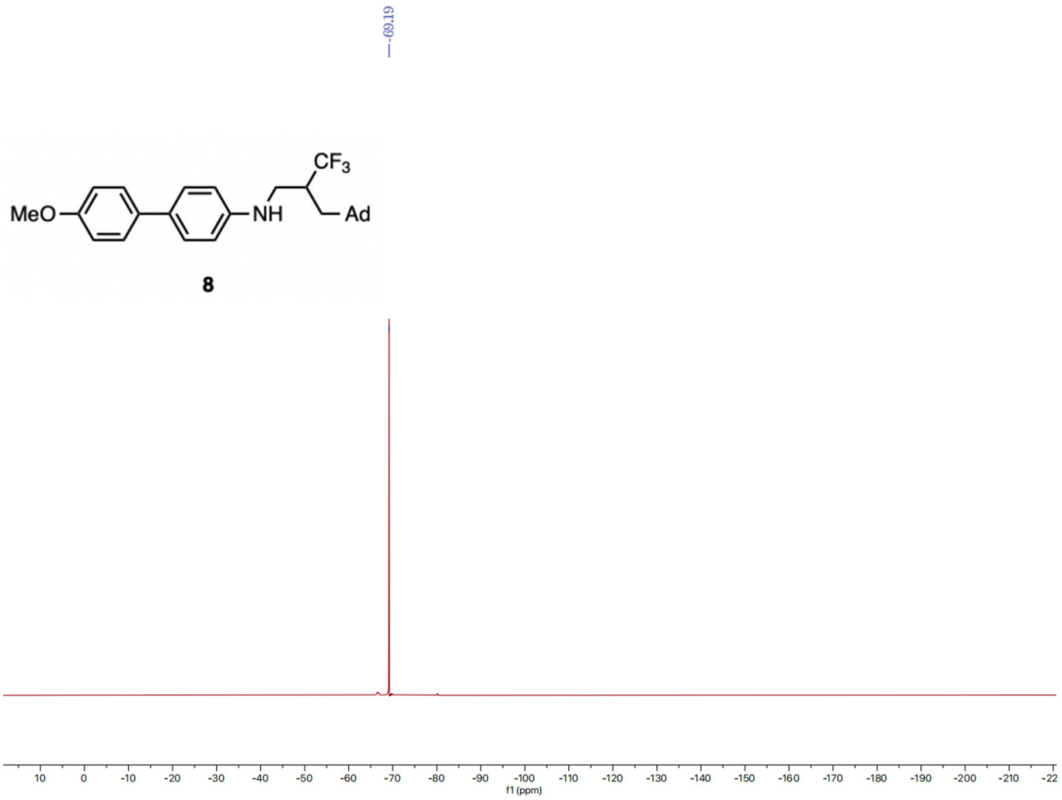


$^{19}$F NMR spectrum (376 MHz, Chloroform-*d*) of compound **8**

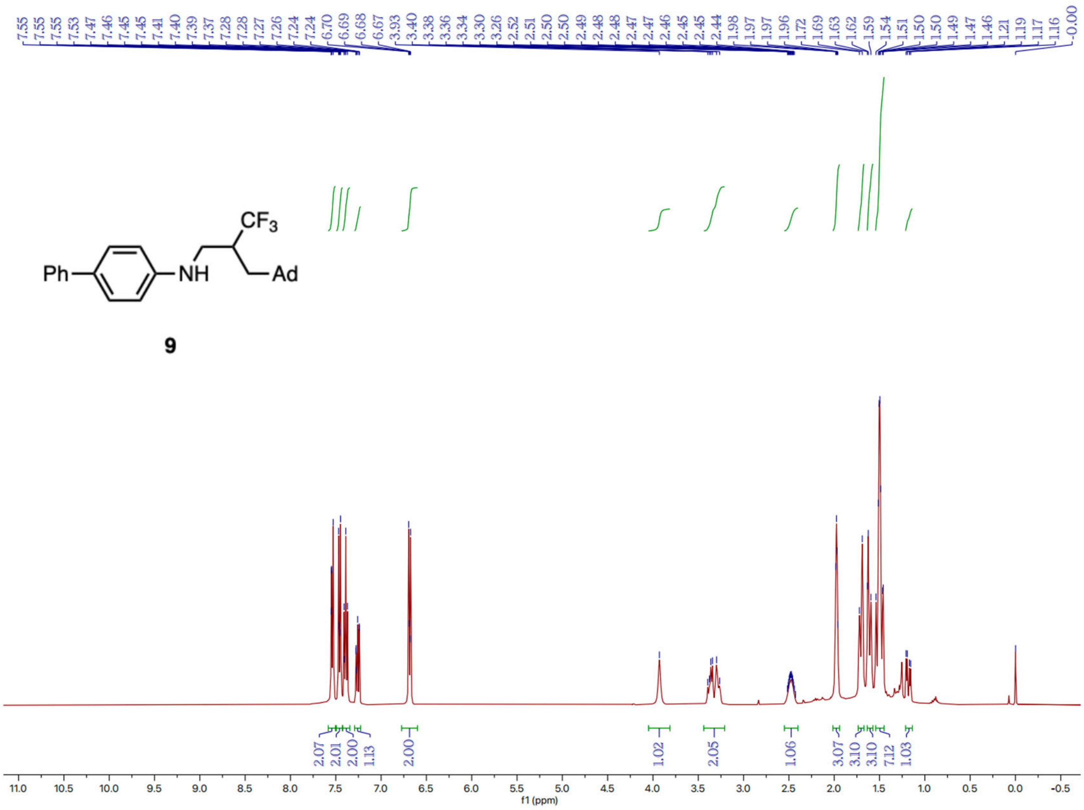

$^{1}$H NMR spectrum (400 MHz, Chloroform-*d*) of compound **9**

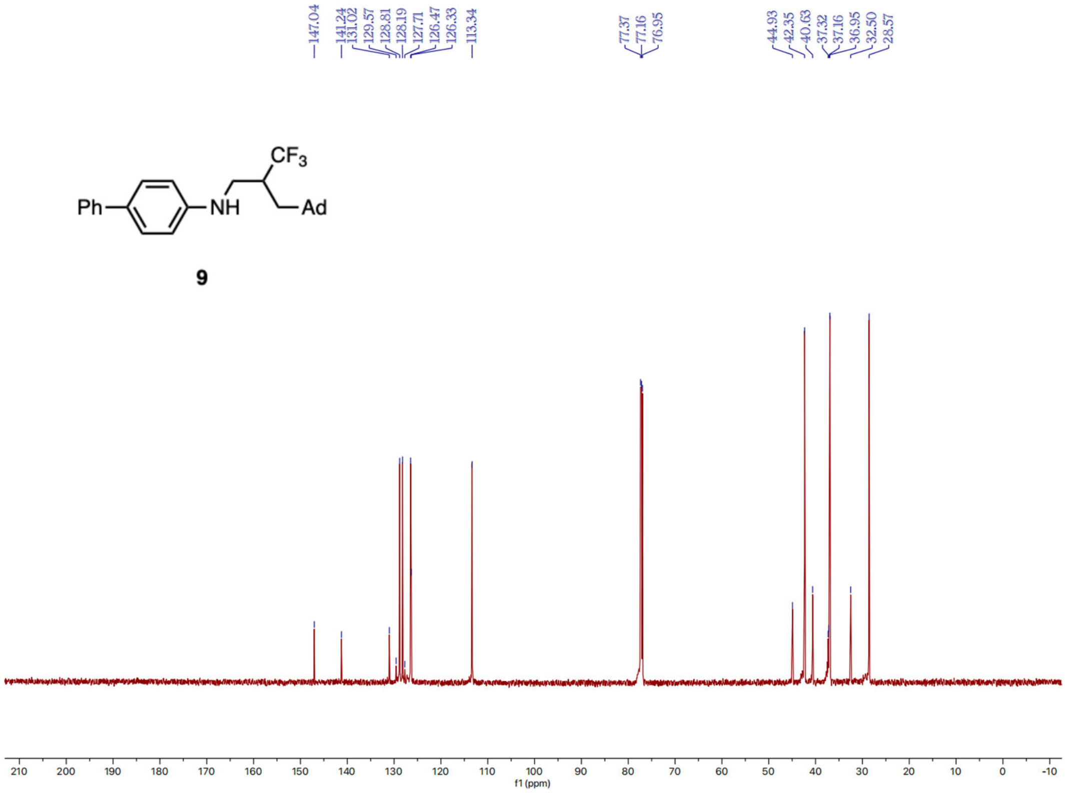

$^{13}$C NMR spectrum (151 MHz, Chloroform-*d*) of compound **9**

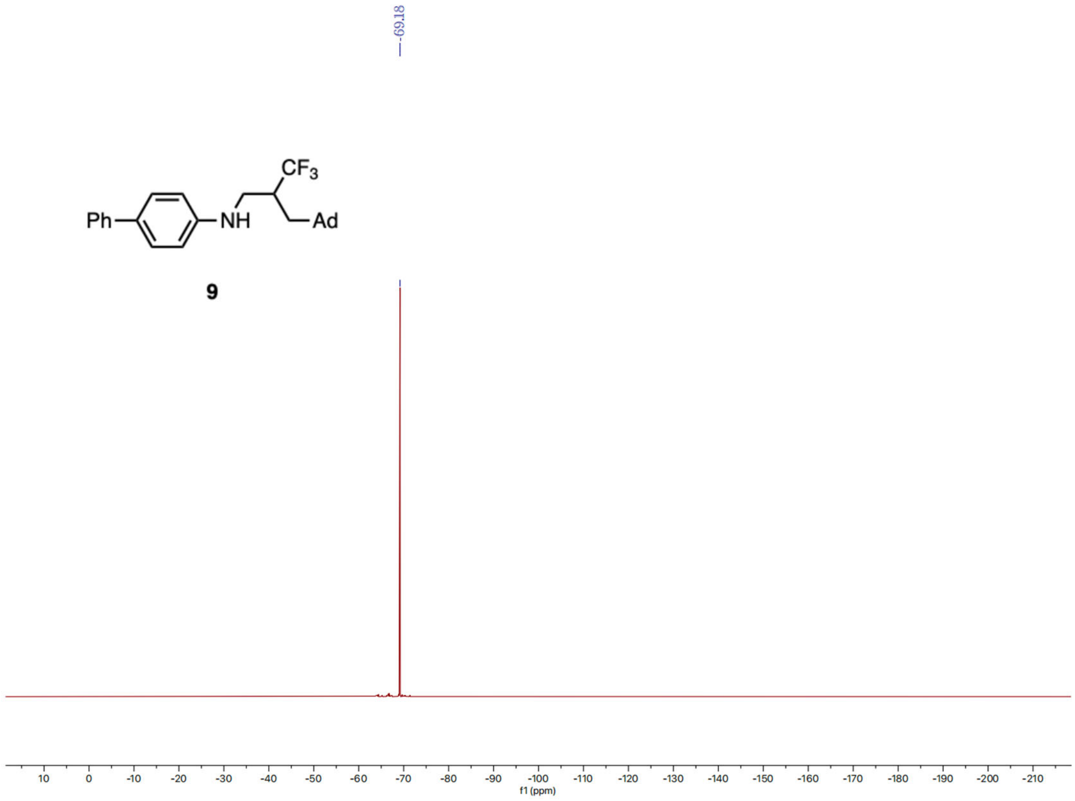


$^{19}$F NMR spectrum (376 MHz, Chloroform-*d*) of compound **9**

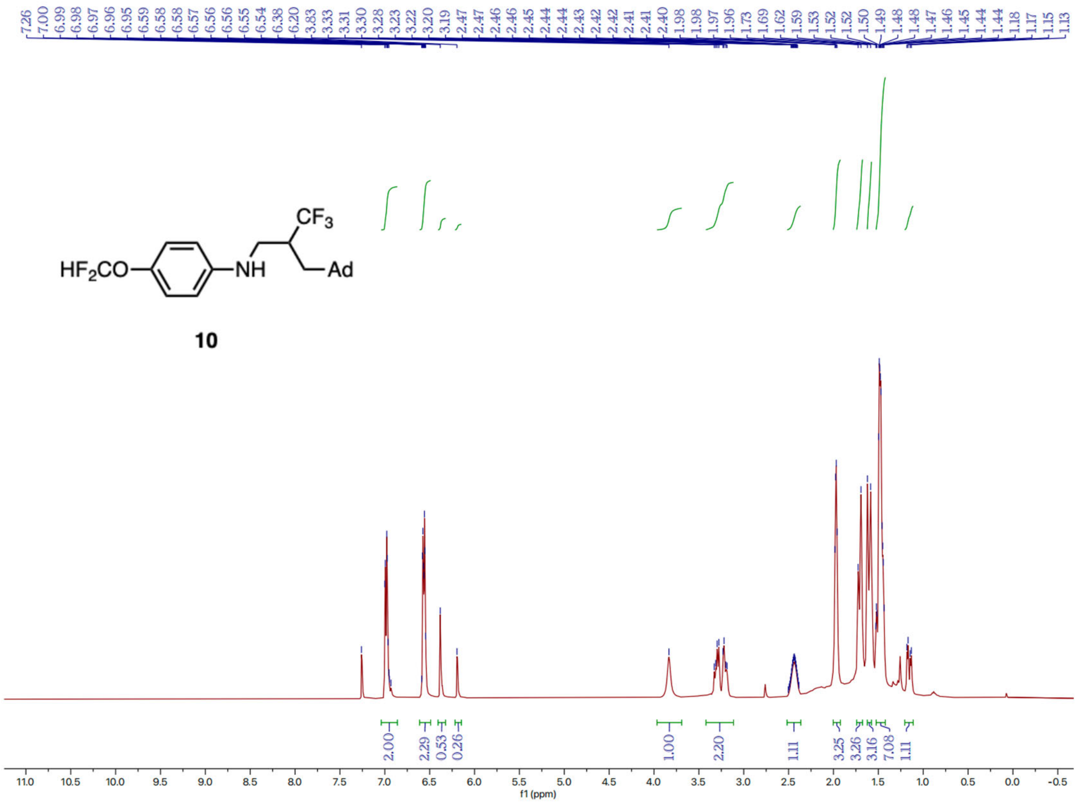


$^1$H NMR spectrum (400 MHz, Chloroform-*d*) of compound **10**

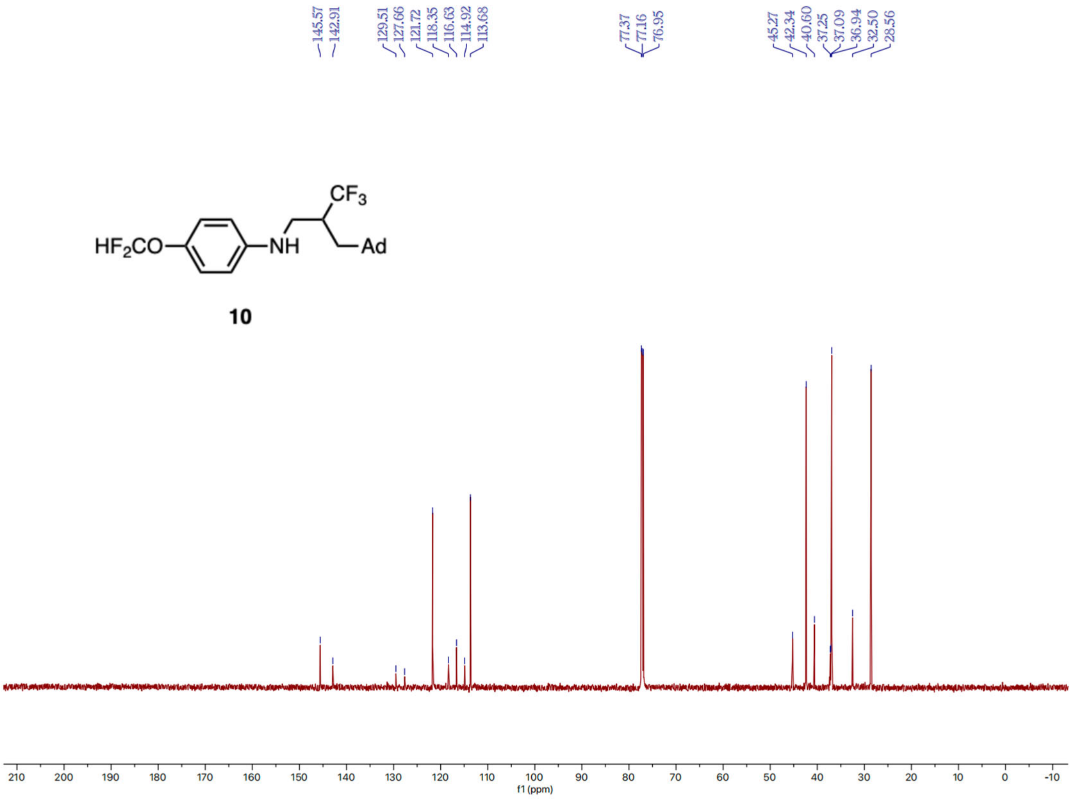


$^{13}$C NMR spectrum (151 MHz, Chloroform-*d*) of compound **10**

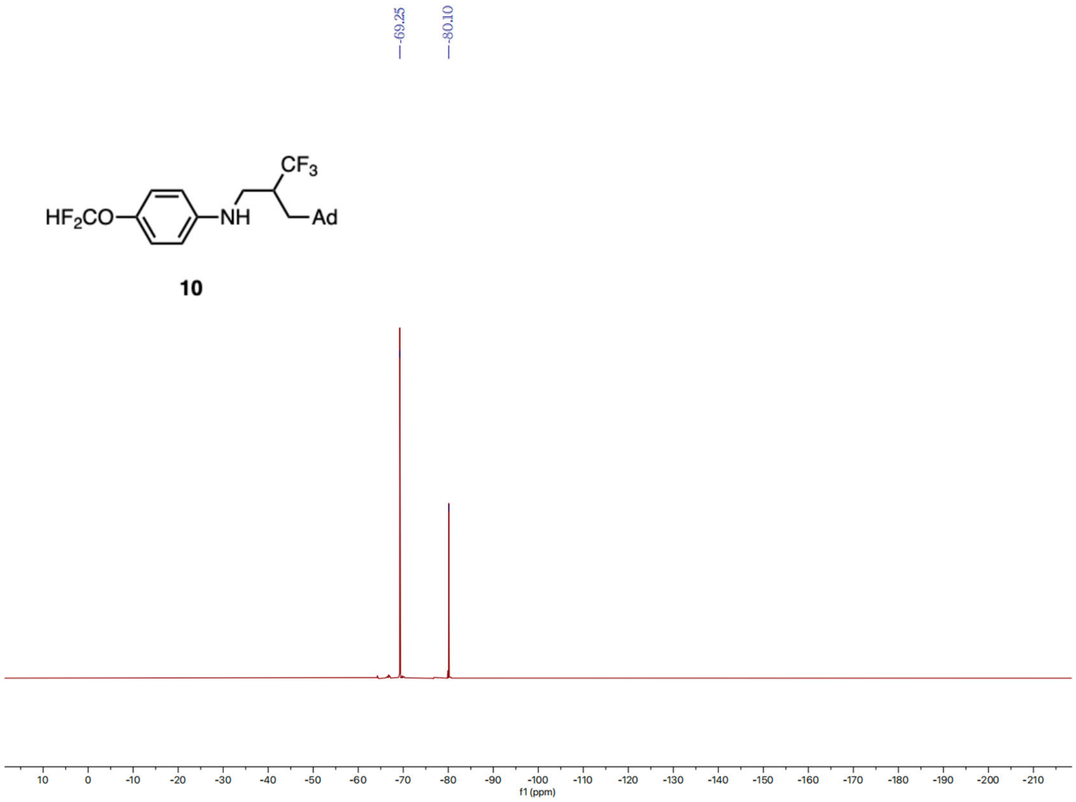


$^{19}$F NMR spectrum (376 MHz, Chloroform-*d*) of compound **10**

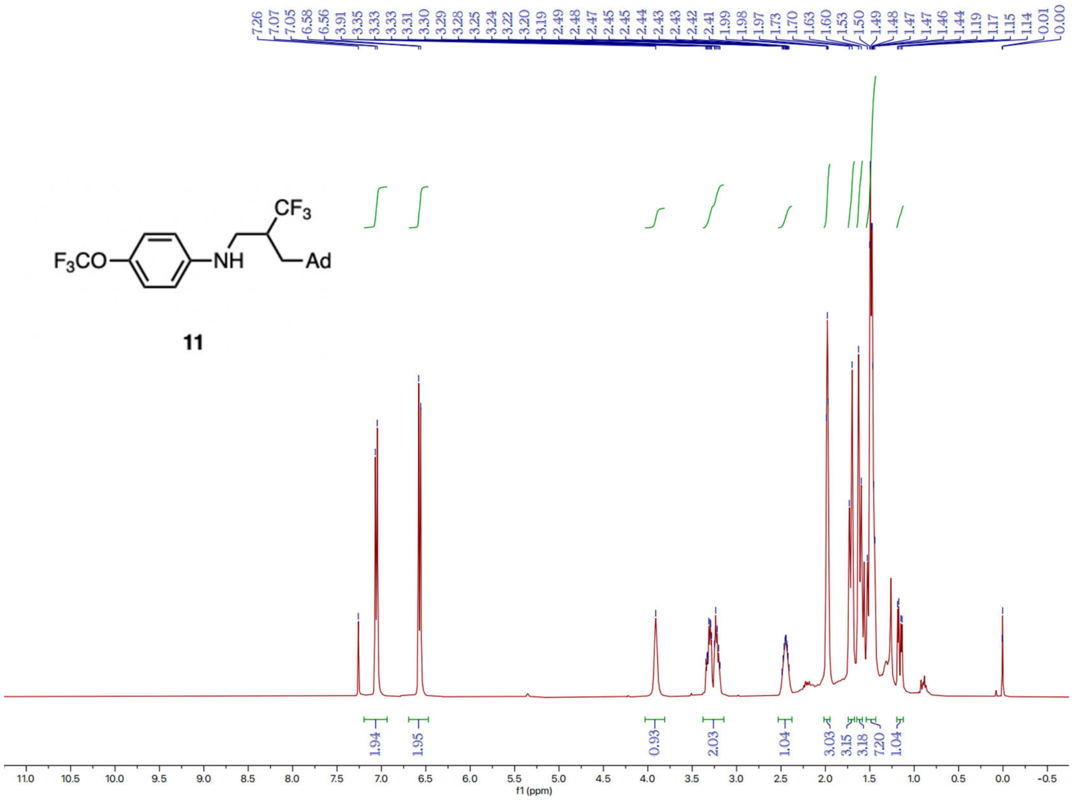

$^{1}$H NMR spectrum (400 MHz, Chloroform-*d*) of compound **11**

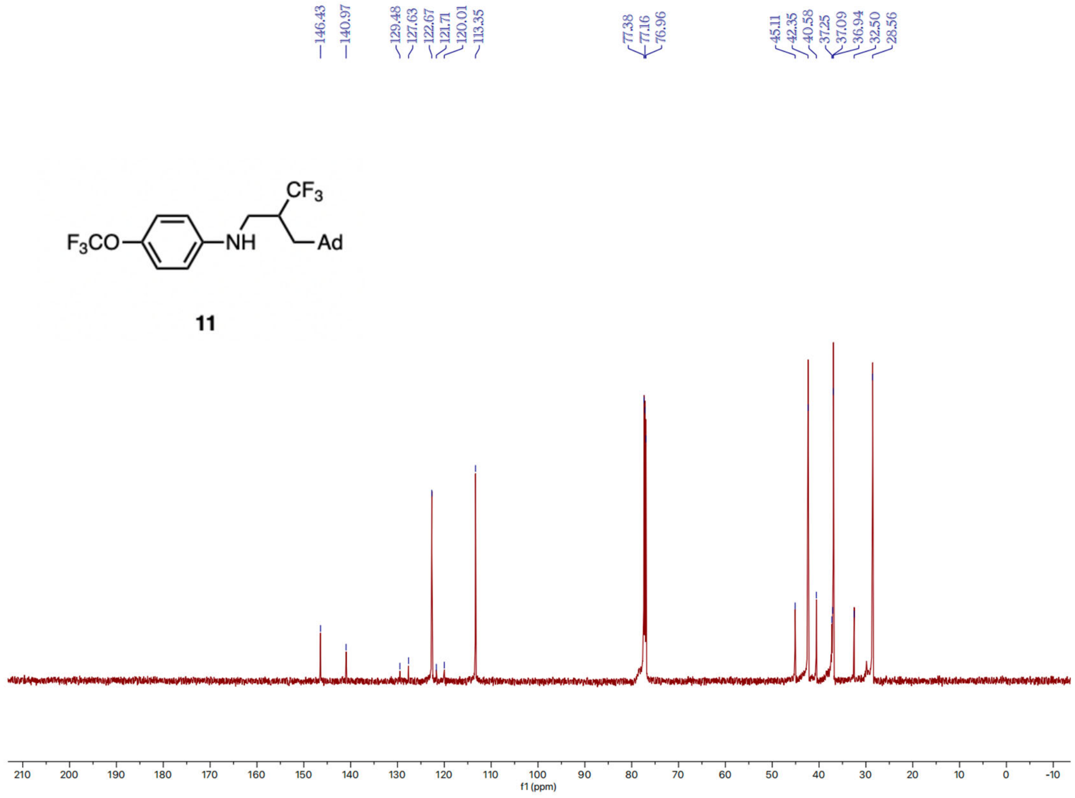

$^{13}$C NMR spectrum (151 MHz, Chloroform-*d*) of compound **11**

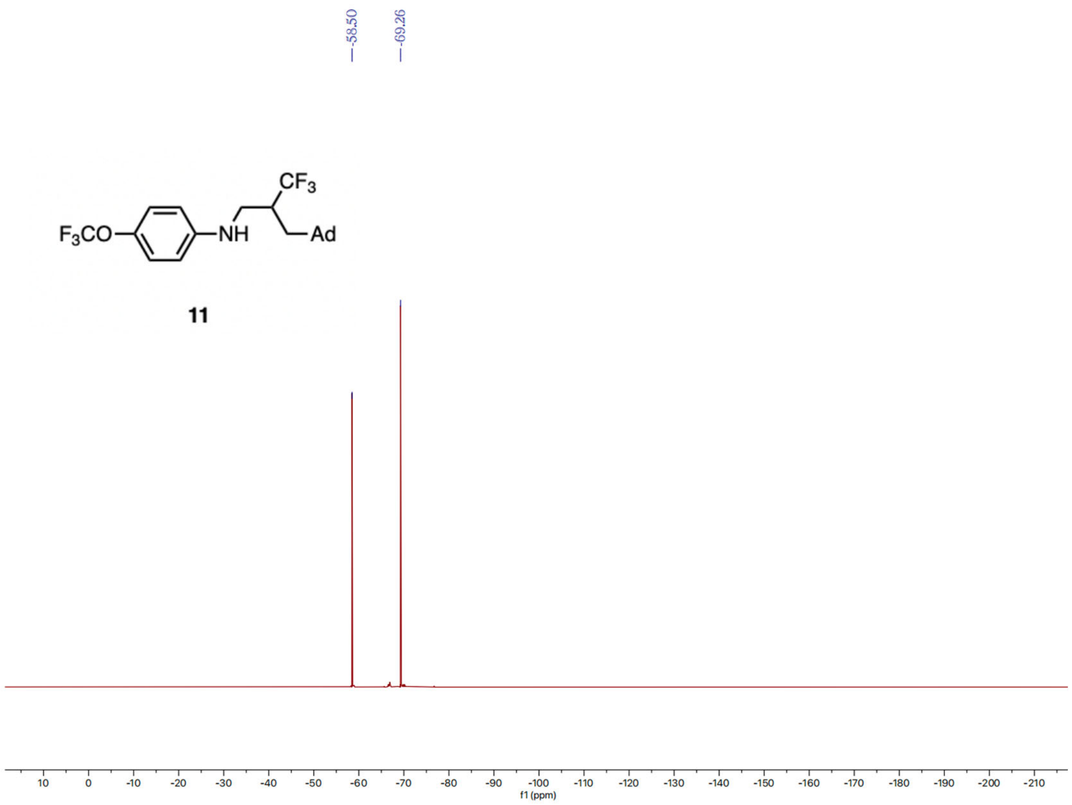


$^{19}$F NMR spectrum (376 MHz, Chloroform-*d*) of compound **11**

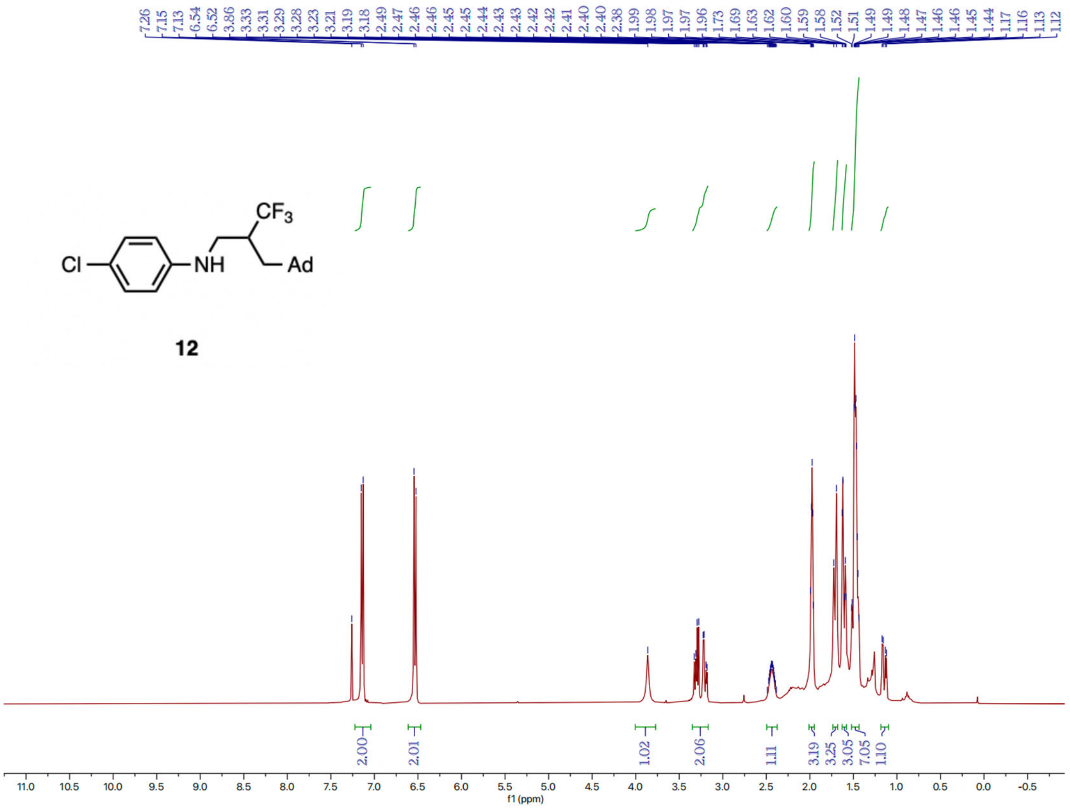

$^{1}$H NMR spectrum (400 MHz, Chloroform-*d*) of compound **12**

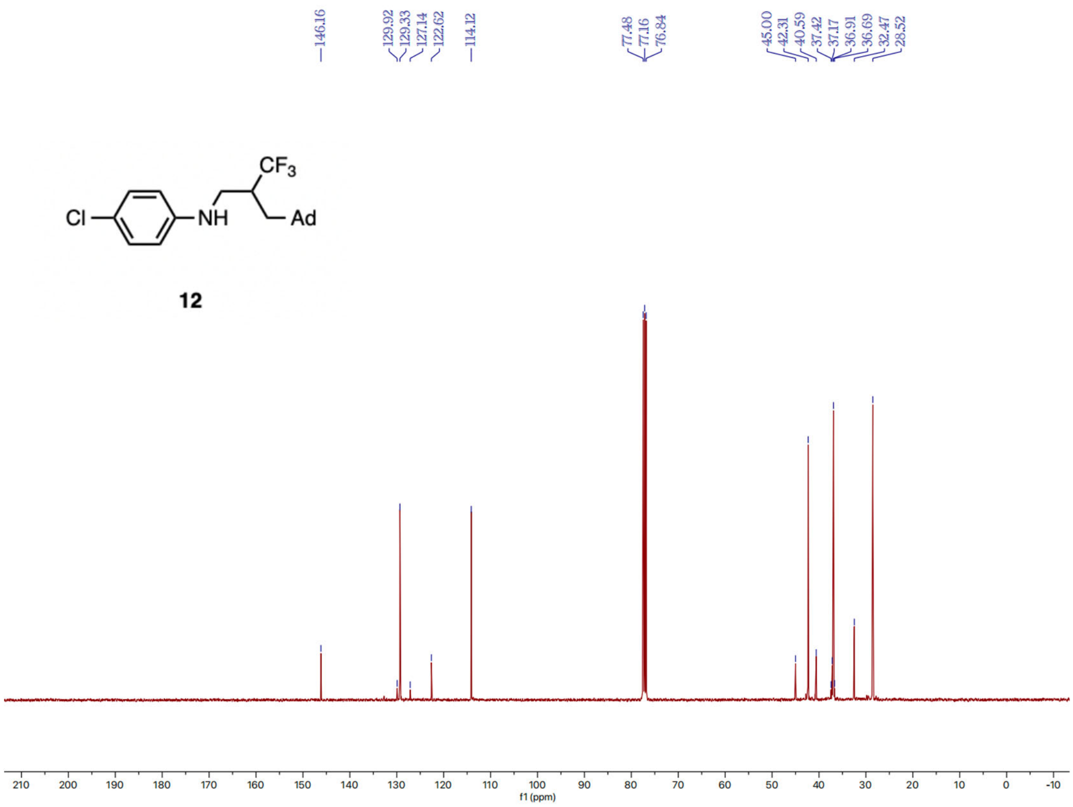

$^{13}$C NMR spectrum (151 MHz, Chloroform-*d*) of compound **12**

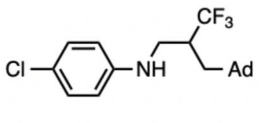


**12**

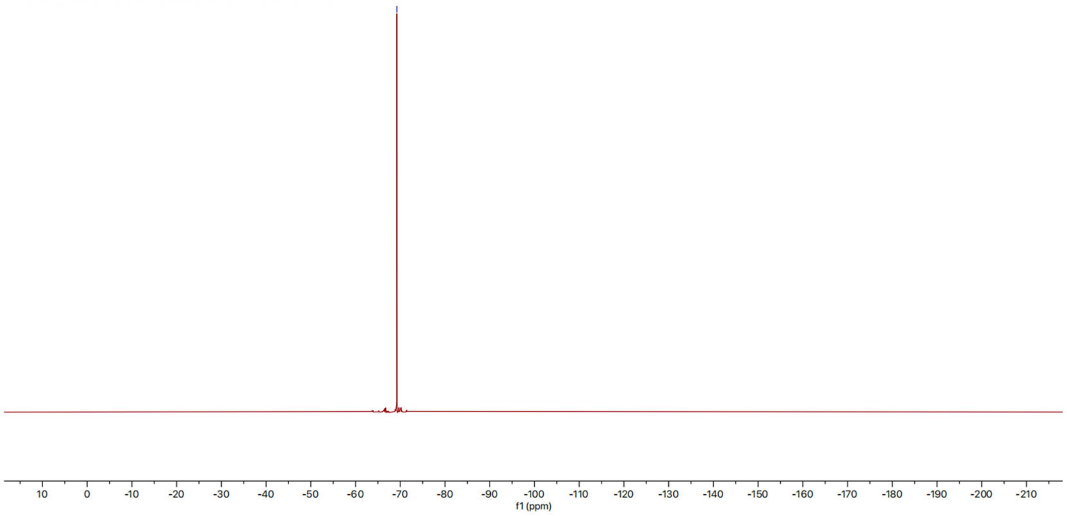


$^{19}$F NMR spectrum (376 MHz, Chloroform-*d*) of compound **12**

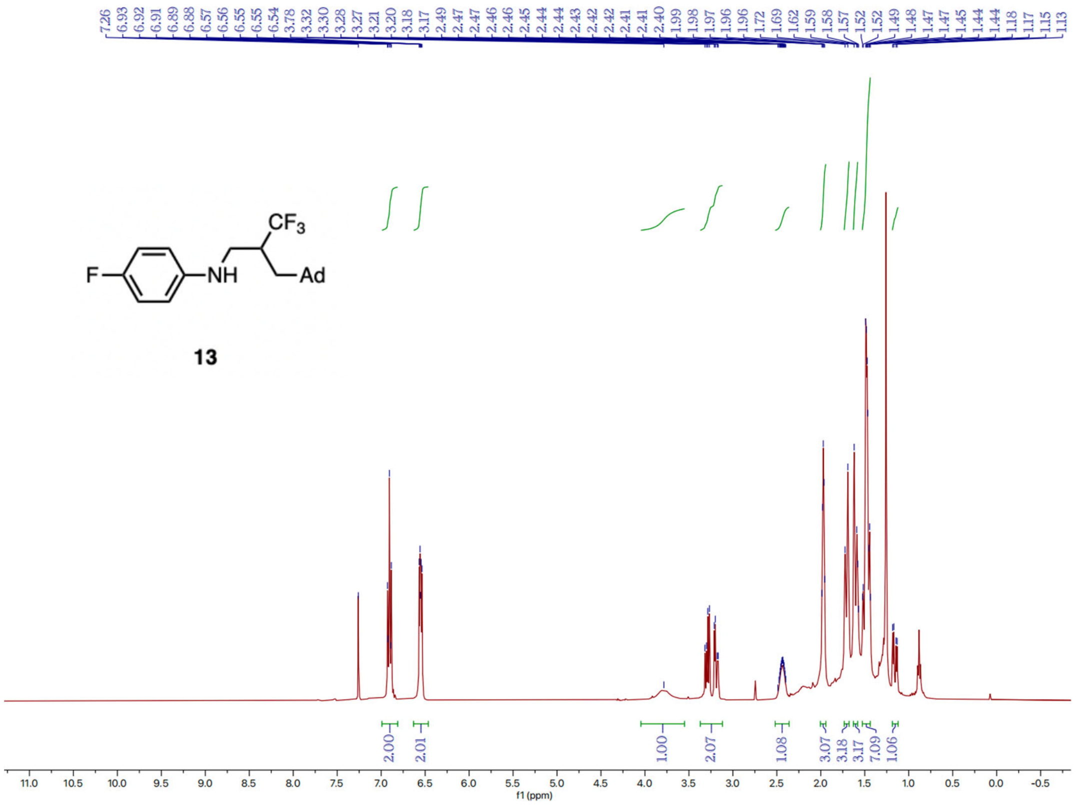

$^{1}$H NMR spectrum (400 MHz, Chloroform-*d*) of compound **13**

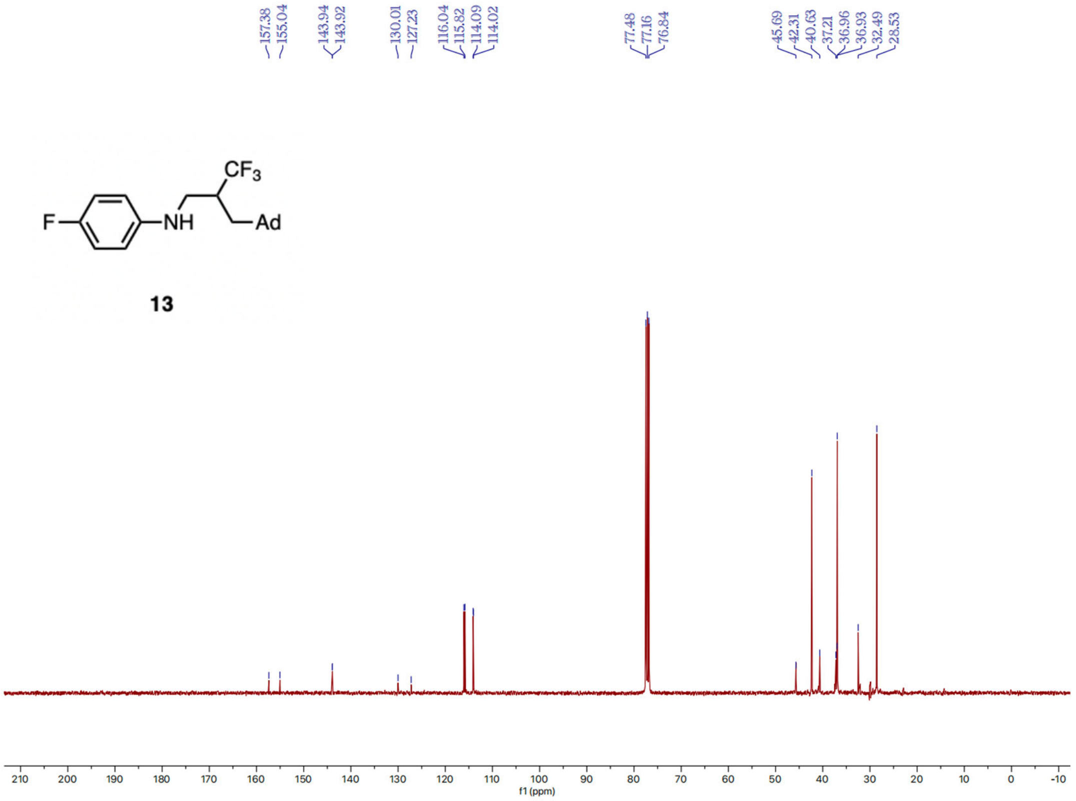

$^{13}$C NMR spectrum (101 MHz, Chloroform-*d*) of compound **13**

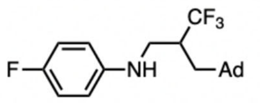


**13**

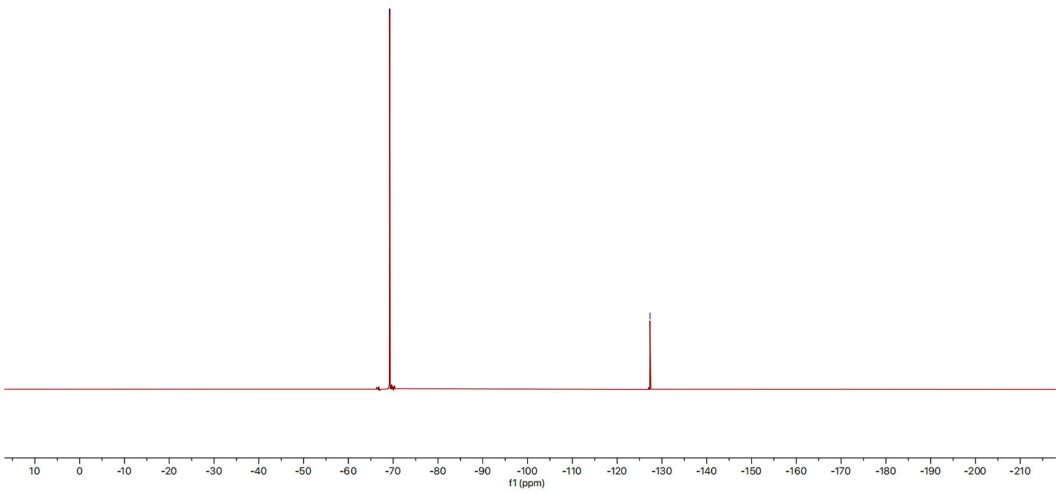


$^{19}$F NMR spectrum (376 MHz, Chloroform-*d*) of compound **13**

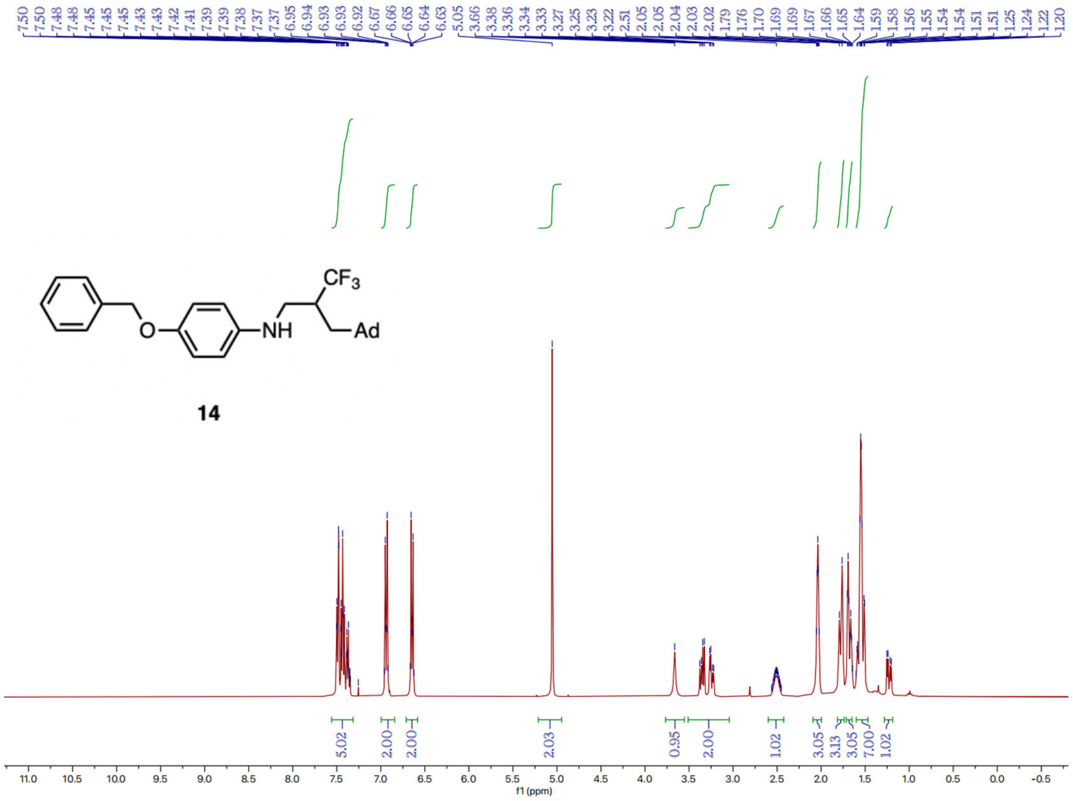

$^{1}$H NMR spectrum (400 MHz, Chloroform-*d*) of compound **14**

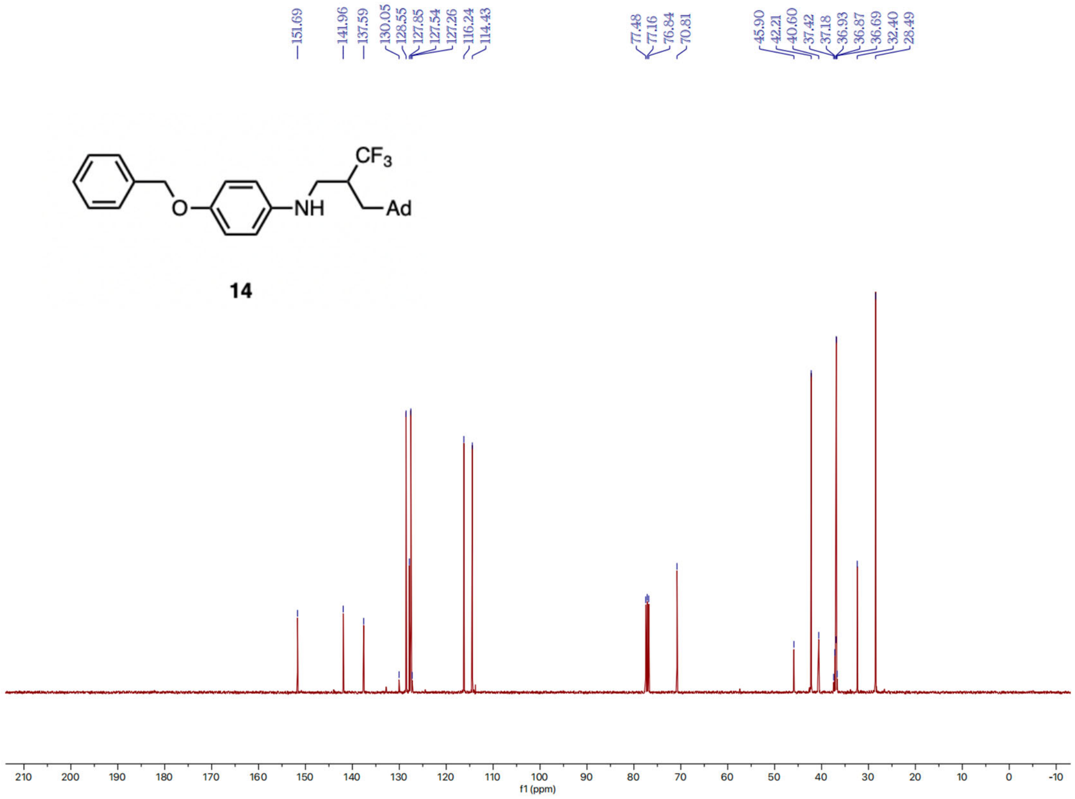

$^{13}$C NMR spectrum (101 MHz, Chloroform-*d*) of compound **14**

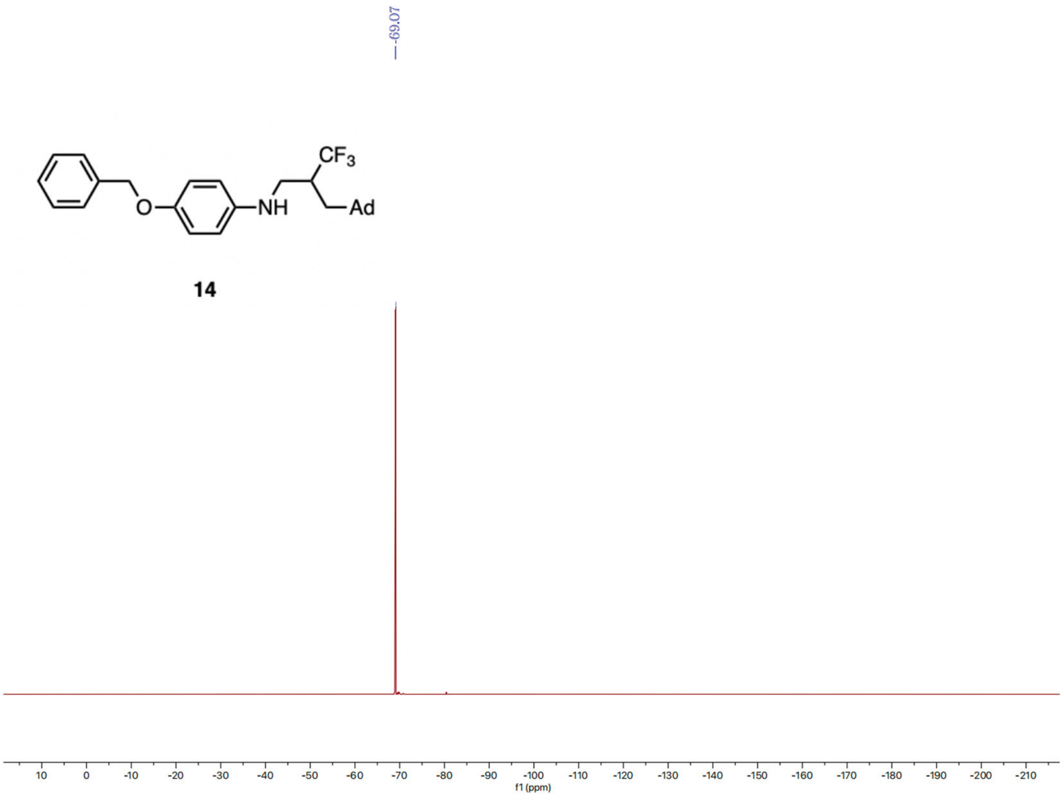


$^{19}$F NMR spectrum (376 MHz, Chloroform-*d*) of compound **14**

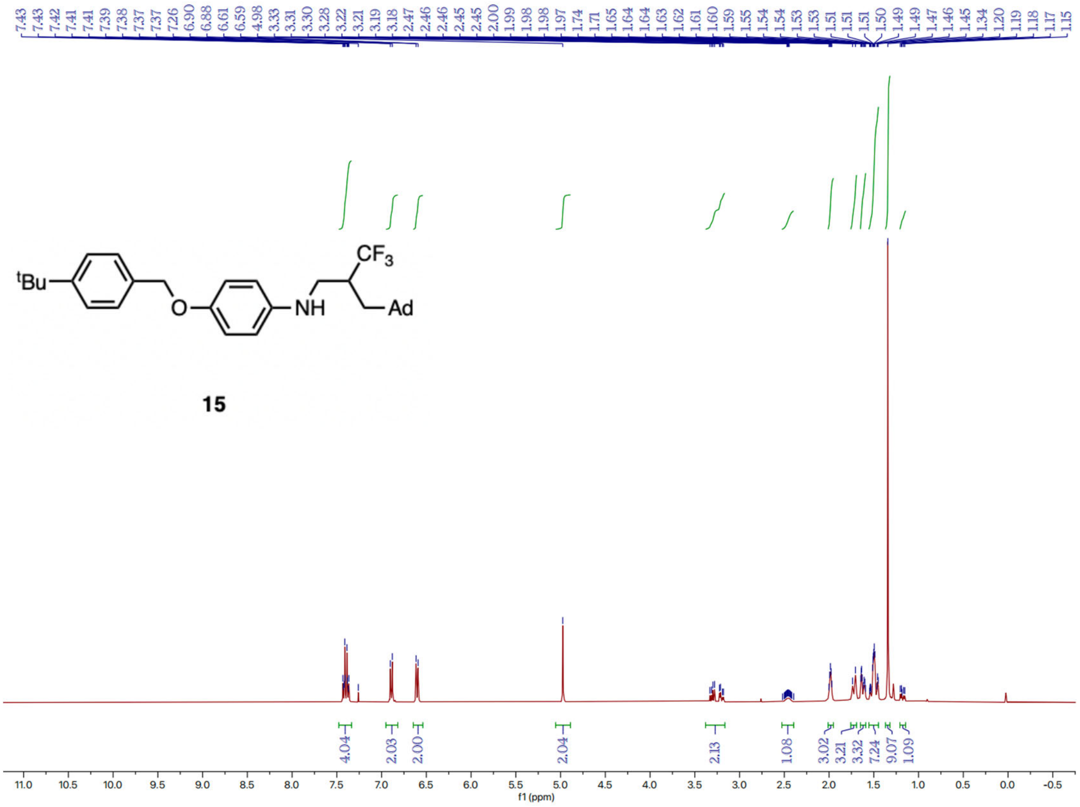

$^{1}$H NMR spectrum (400 MHz, Chloroform-$d$) of compound **15**

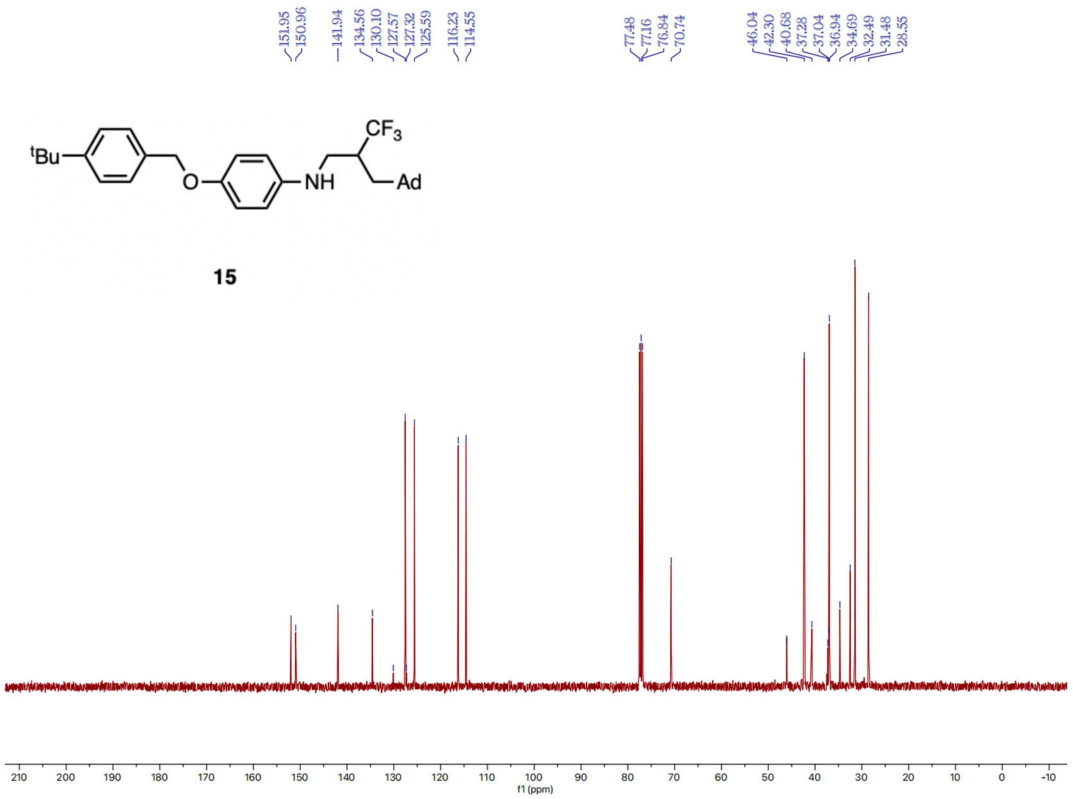

$^{13}$C NMR spectrum (101 MHz, Chloroform-$d$) of compound **15**

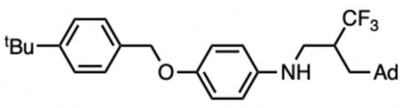

**15**

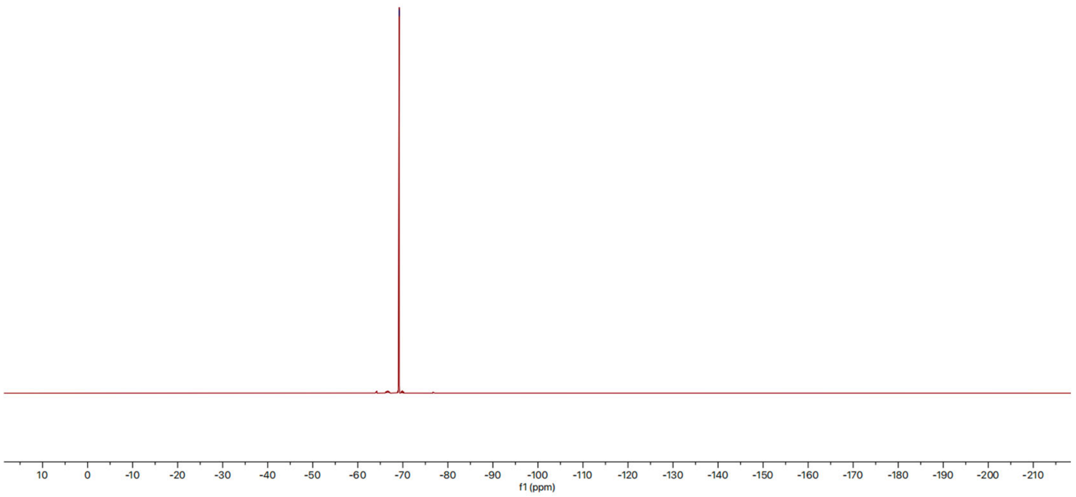


$^{19}$F NMR spectrum (376 MHz, Chloroform-*d*) of compound **15**

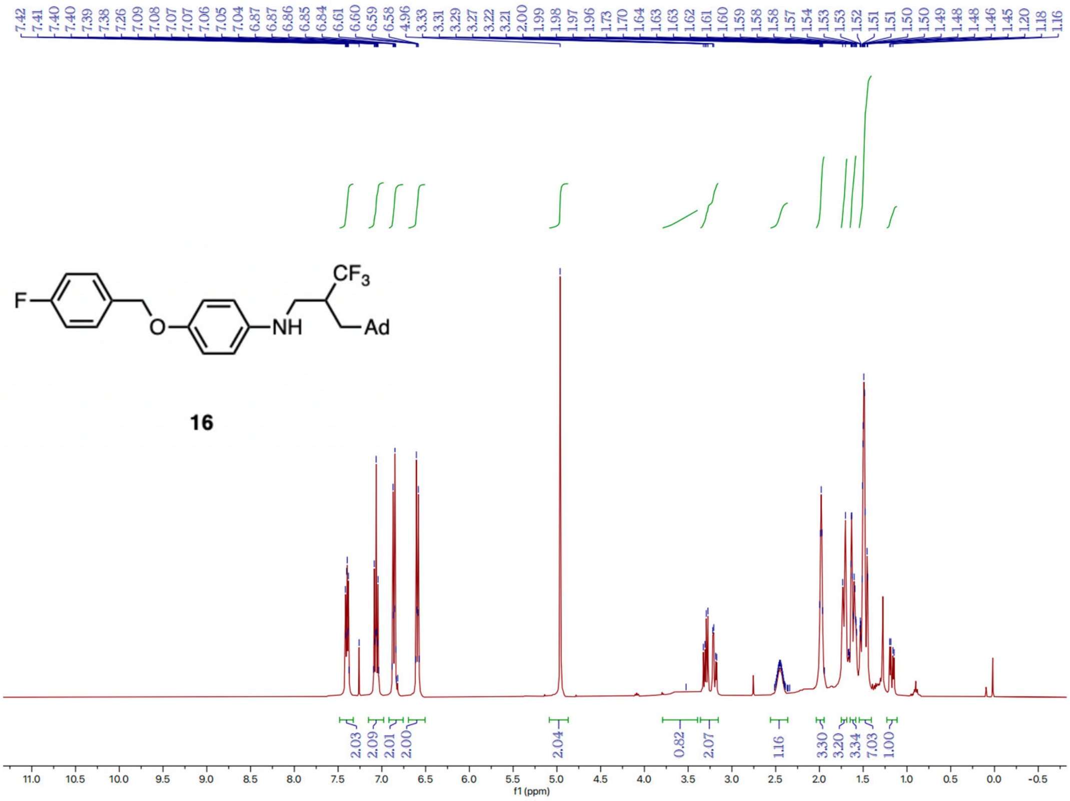


$^{1}$H NMR spectrum (400 MHz, Chloroform-*d*) of compound **16**

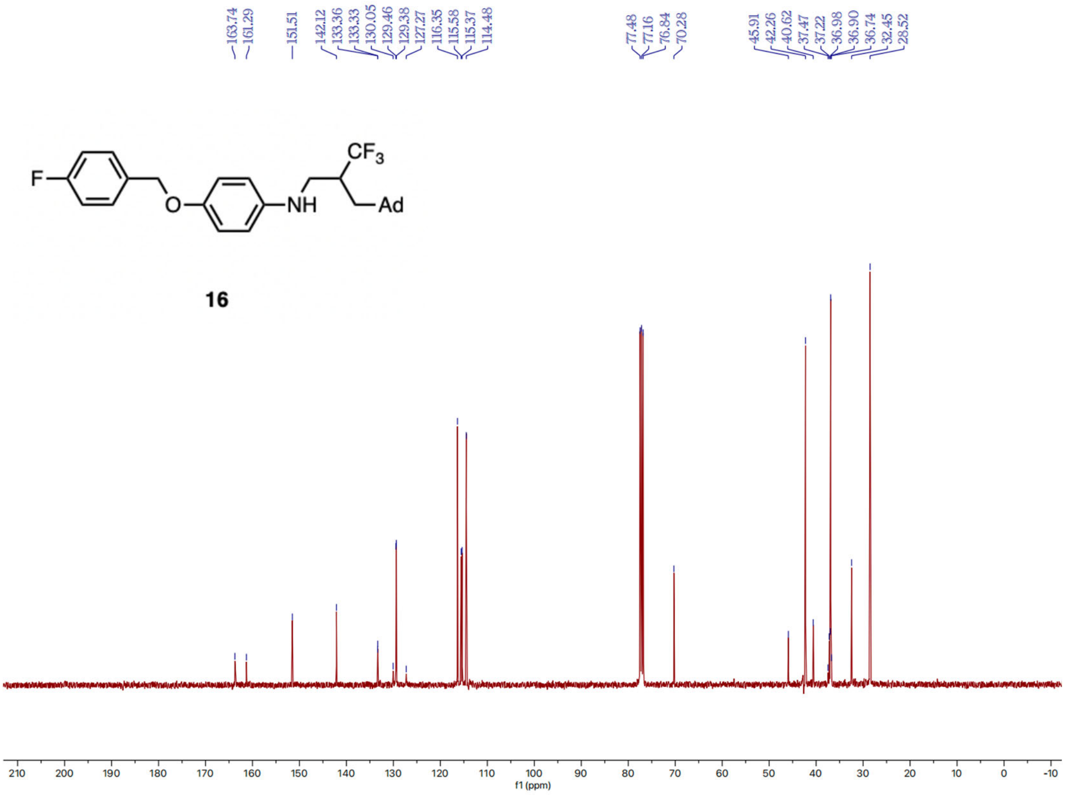


$^{13}$C NMR spectrum (101 MHz, Chloroform-*d*) of compound **16**

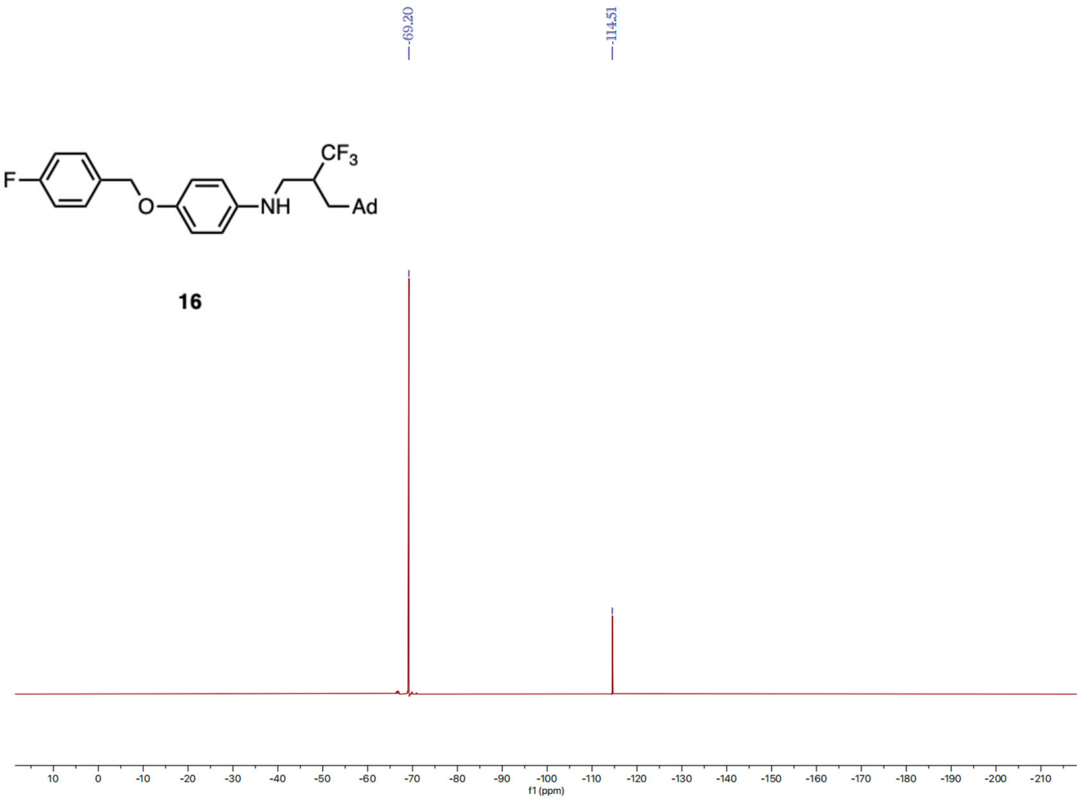


$^{19}$F NMR spectrum (376 MHz, Chloroform-*d*) of compound **16**

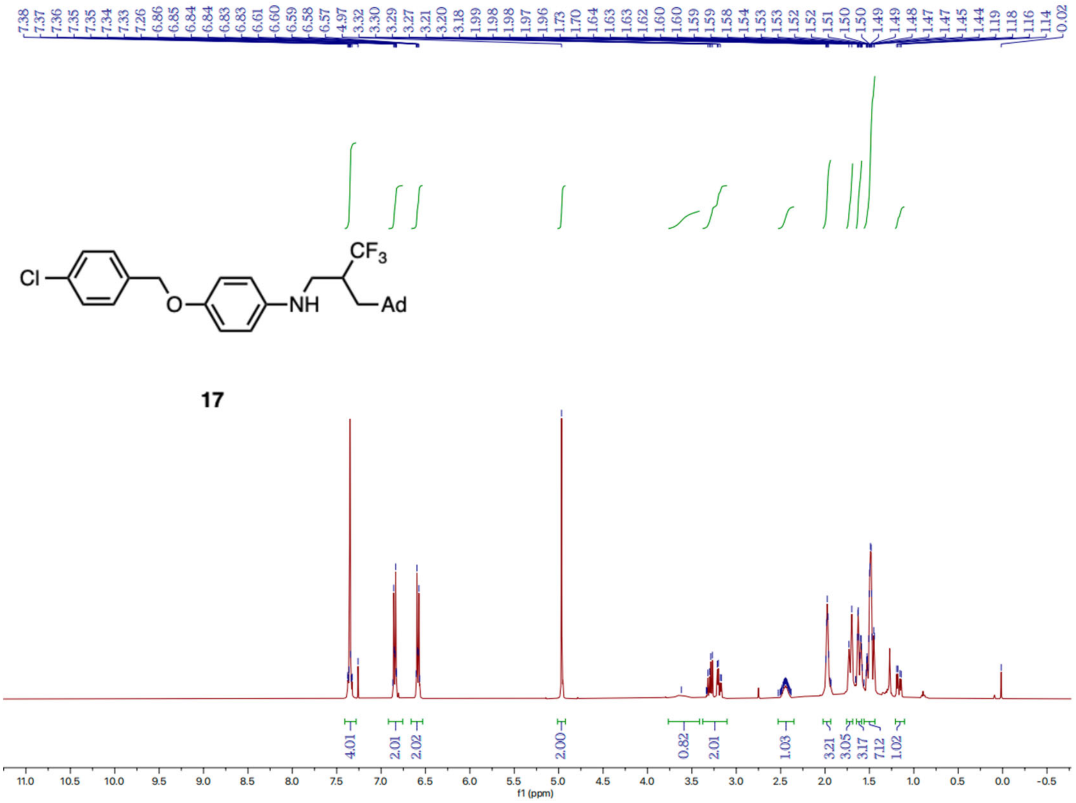

$^1$H NMR spectrum (400 MHz, Chloroform-*d*) of compound **17**

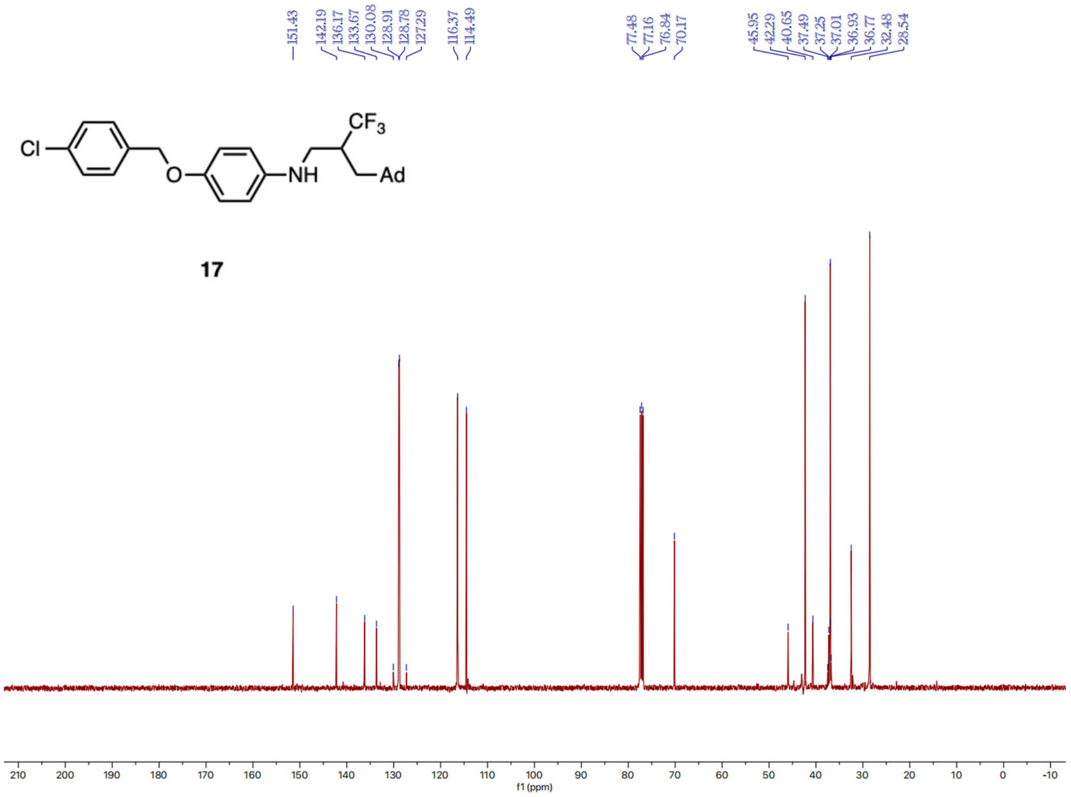

$^{13}$C NMR spectrum (101 MHz, Chloroform-*d*) of compound **17**

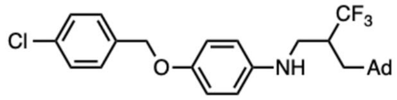


**17**

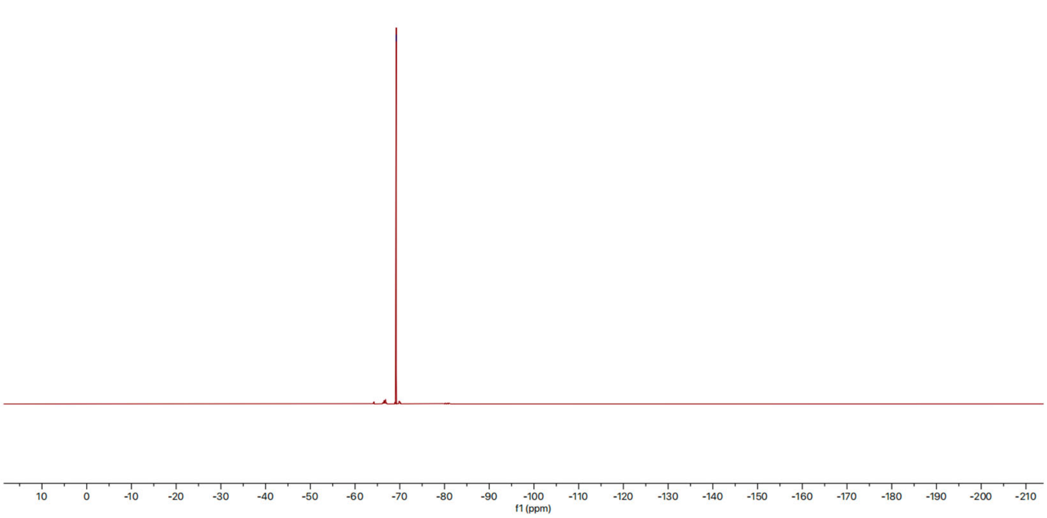


$^{19}$F NMR spectrum (376 MHz, Chloroform-*d*) of compound **17**

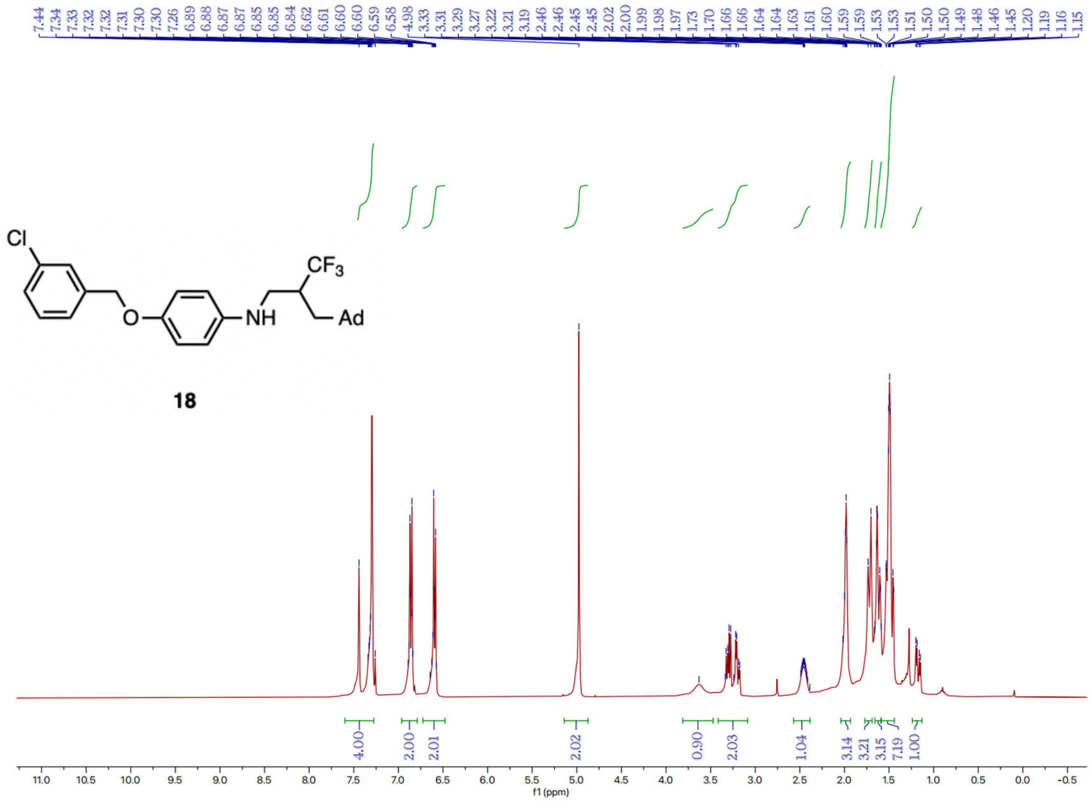

$^{1}$H NMR spectrum (400 MHz, Chloroform-*d*) of compound **18**

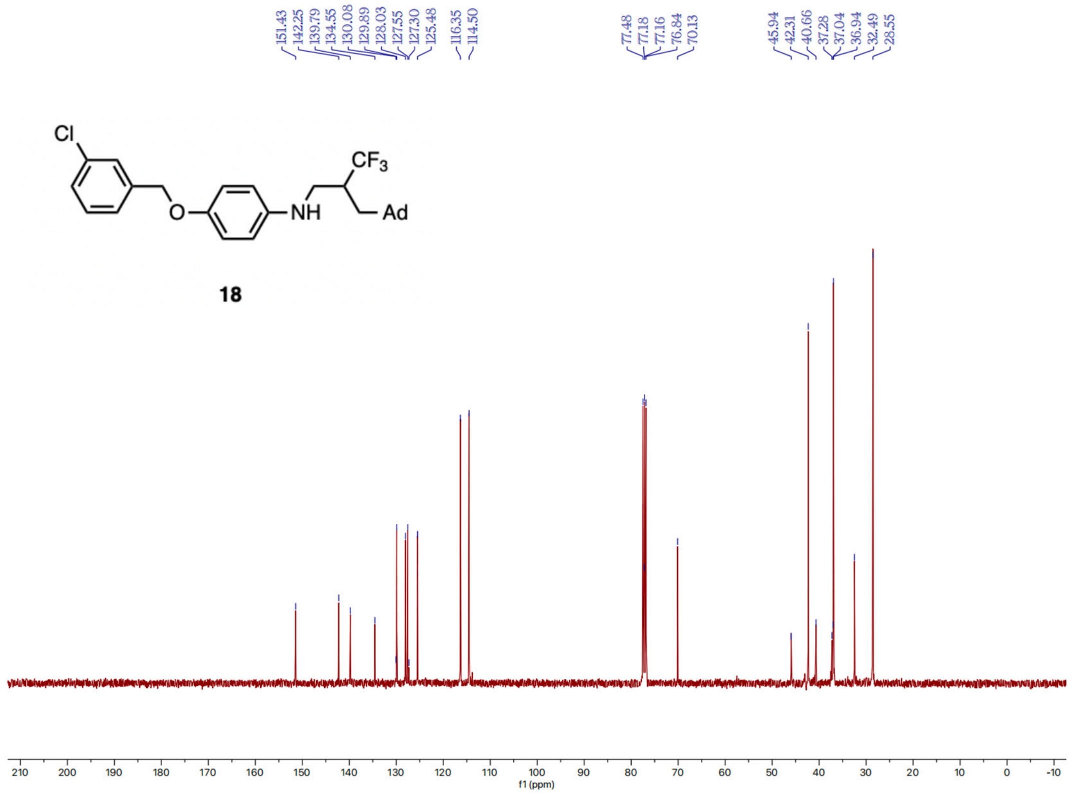

$^{13}$C NMR spectrum (101 MHz, Chloroform-*d*) of compound **18**

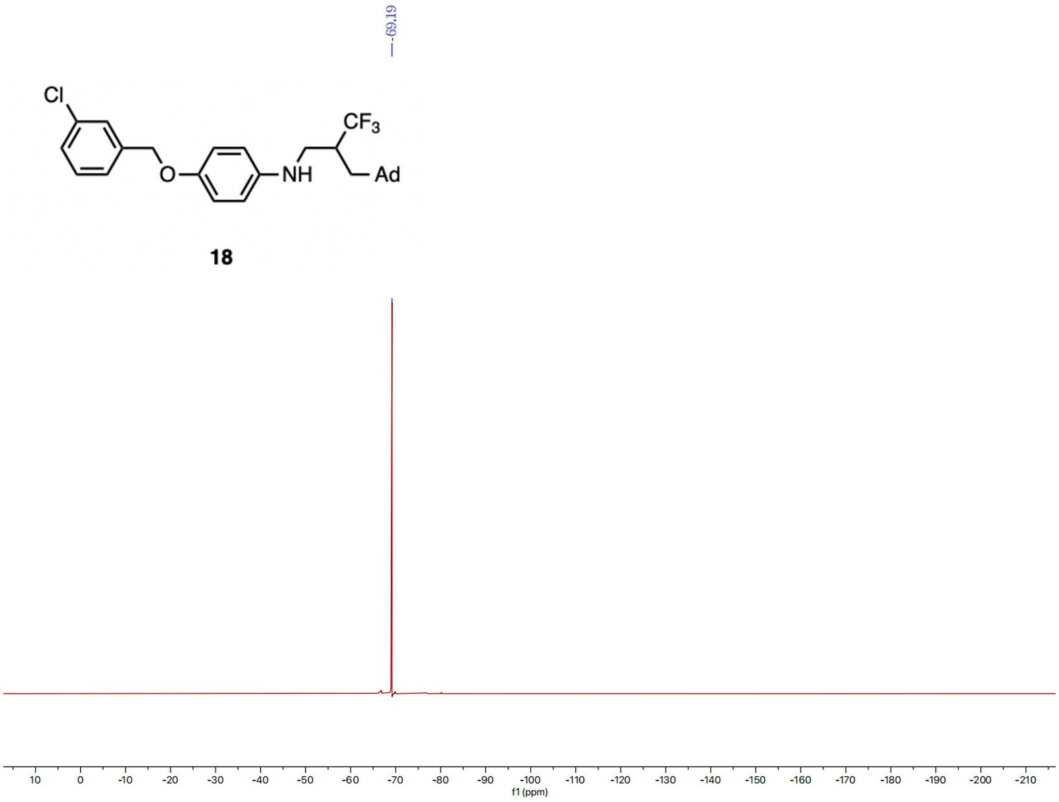


$^{19}$F NMR spectrum (376 MHz, Chloroform-*d*) of compound **18**

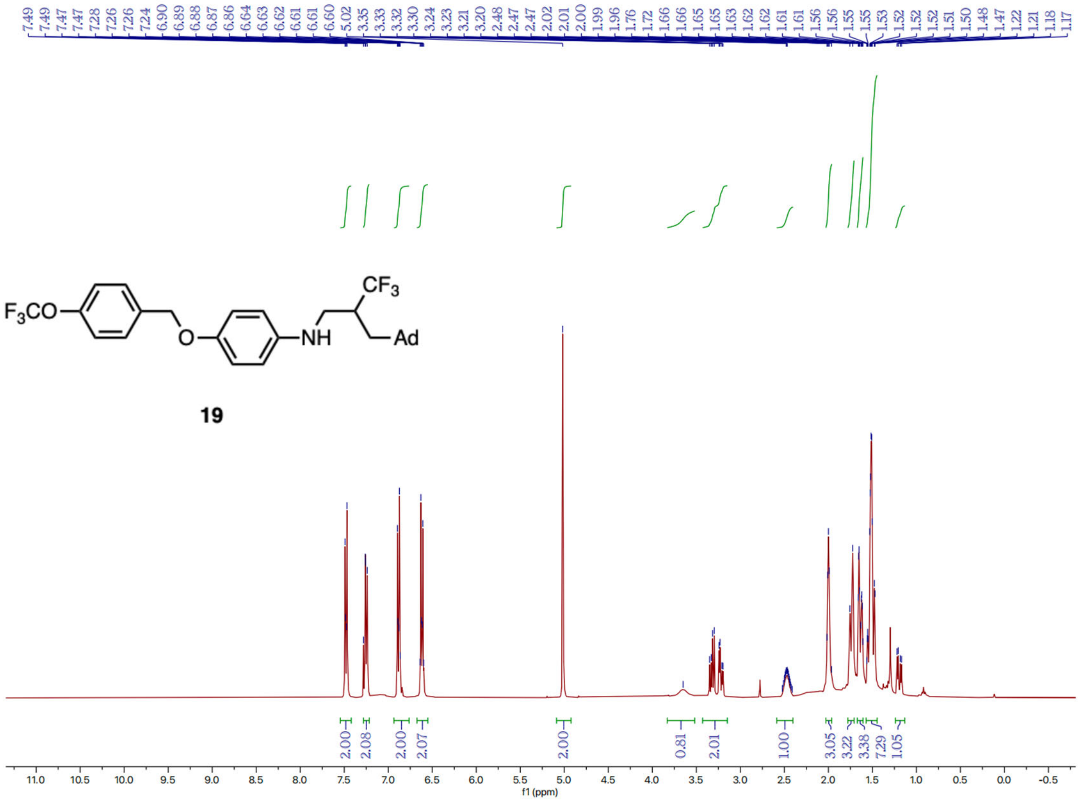

$^{1}$H NMR spectrum (400 MHz, Chloroform-*d*) of compound **19**

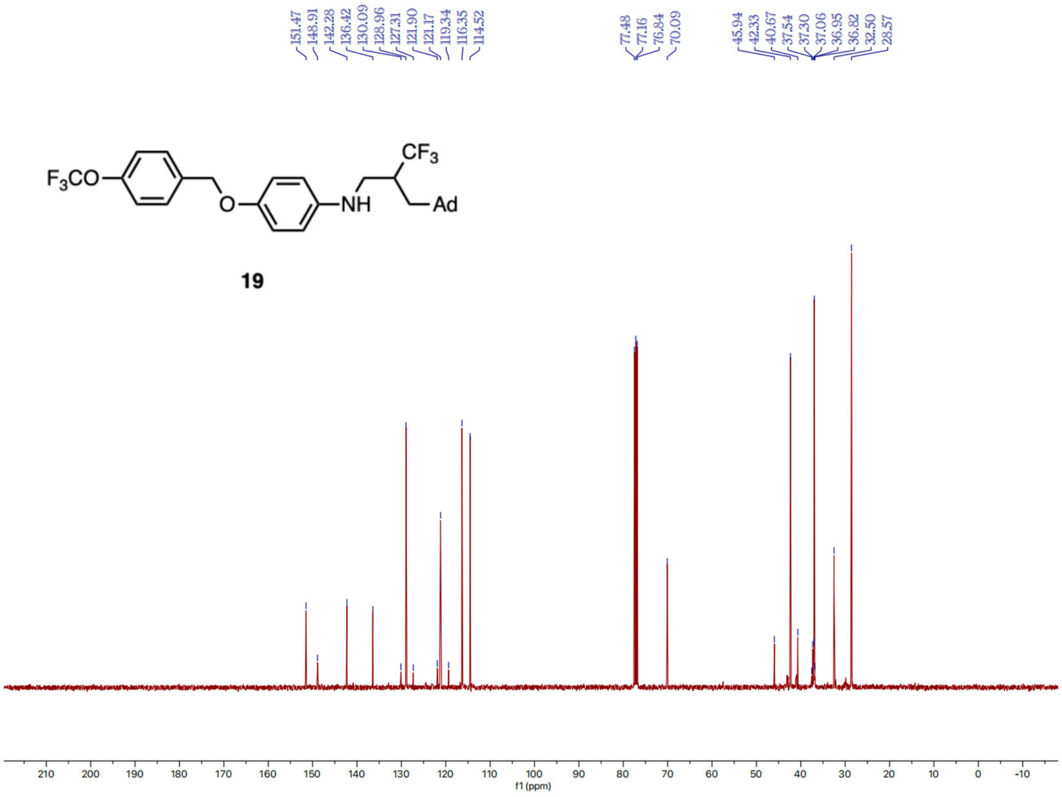

$^{13}$C NMR spectrum (101 MHz, Chloroform-*d*) of compound **19**

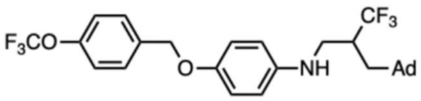


**19**

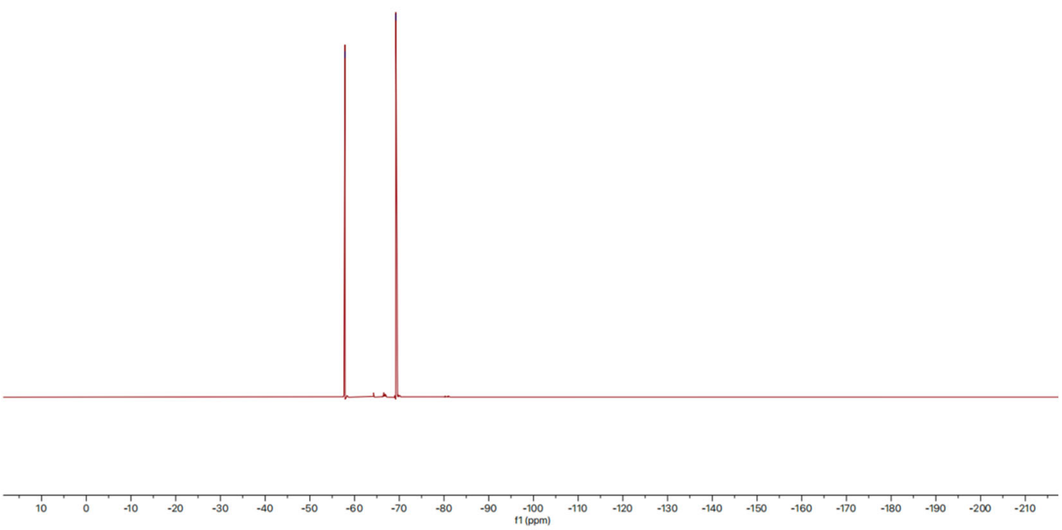


$^{19}$F NMR spectrum (376 MHz, Chloroform-*d*) of compound **19**

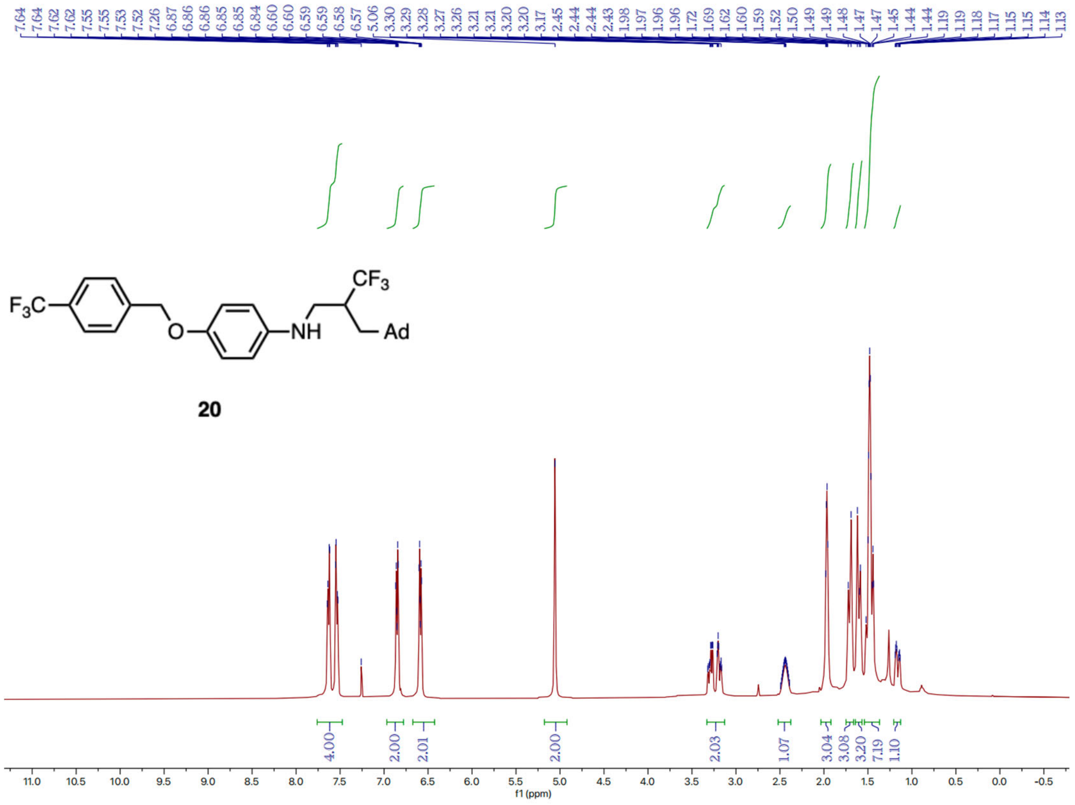

$^{1}$H NMR spectrum (400 MHz, Chloroform-*d*) of compound **20**

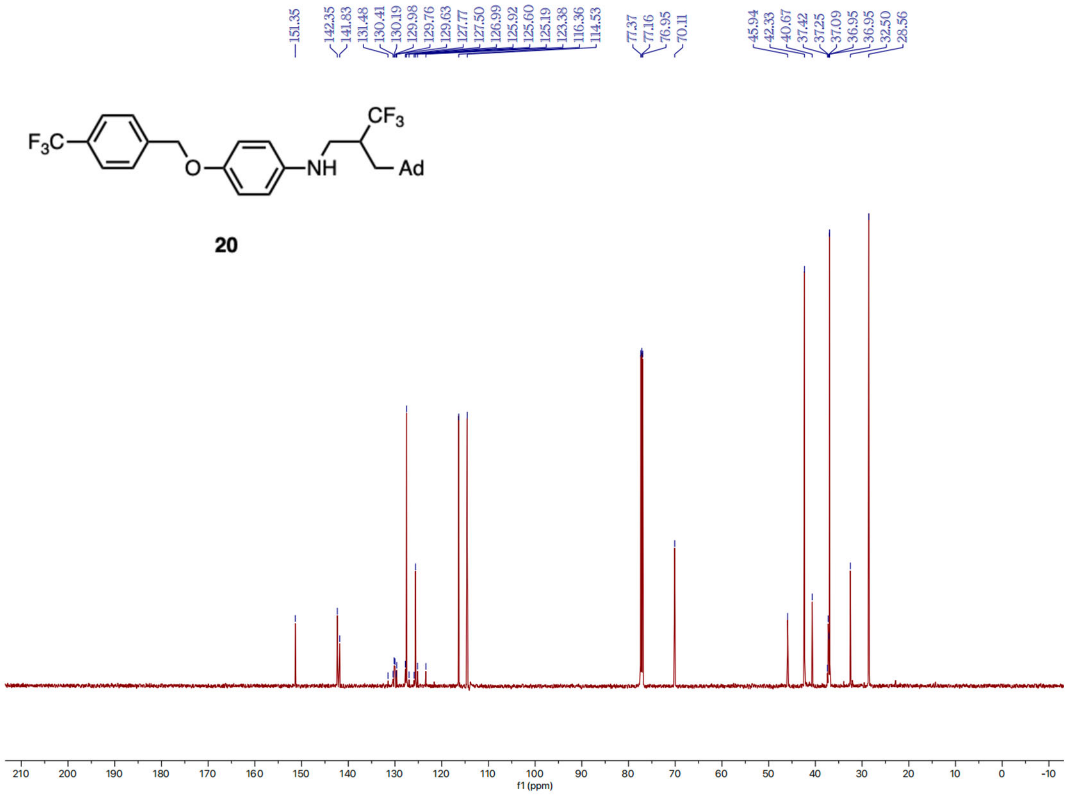

$^{13}$C NMR spectrum (151 MHz, Chloroform-*d*) of compound **20**

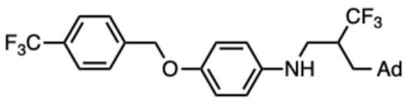

**20**

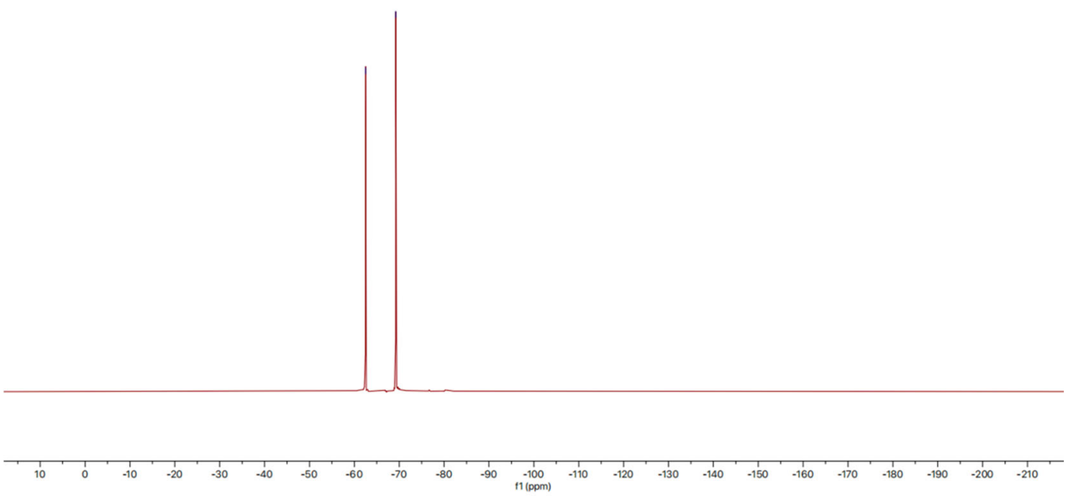


$^{19}$F NMR spectrum (376 MHz, Chloroform-*d*) of compound **20**

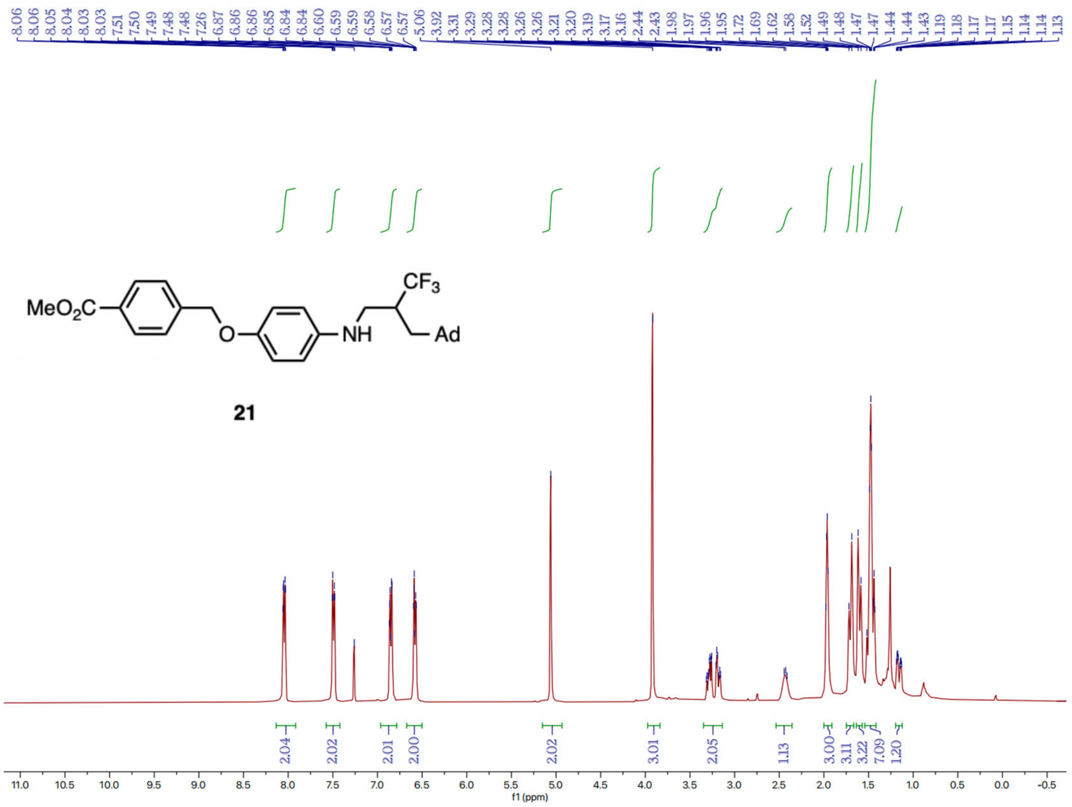

$^{1}H$ NMR spectrum (400 MHz, Chloroform-*d*) of compound **21**

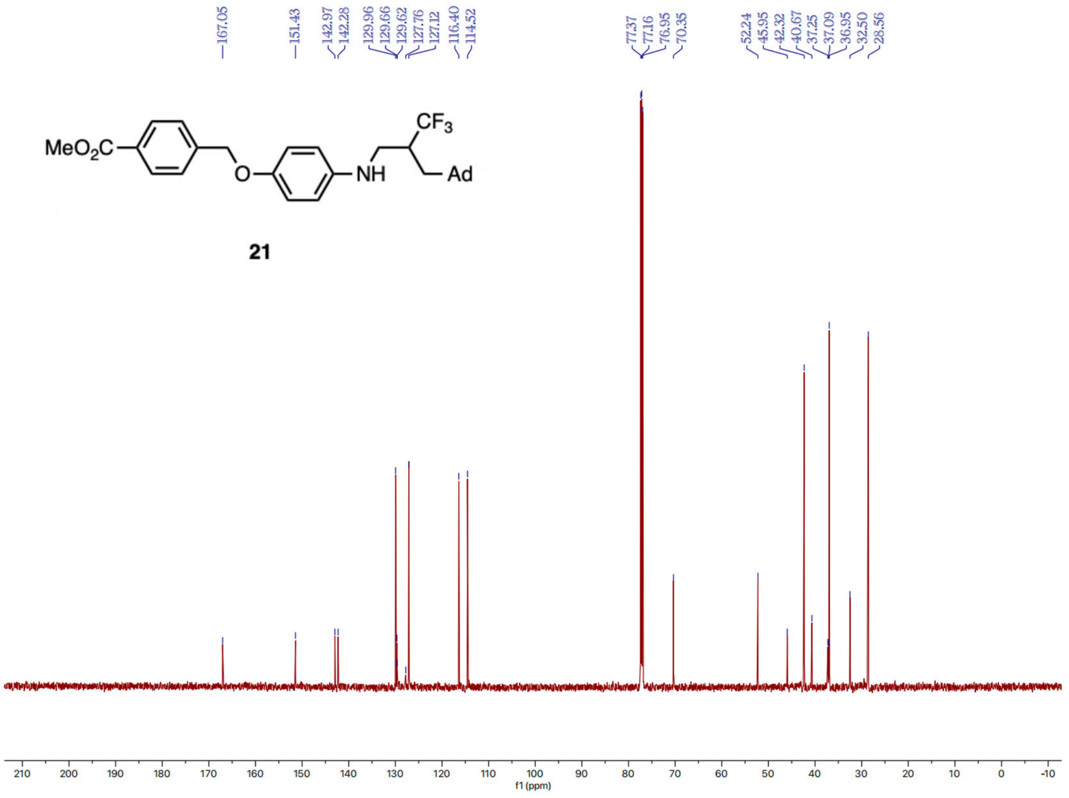

$^{13}C$ NMR spectrum (151 MHz, Chloroform-*d*) of compound **21**

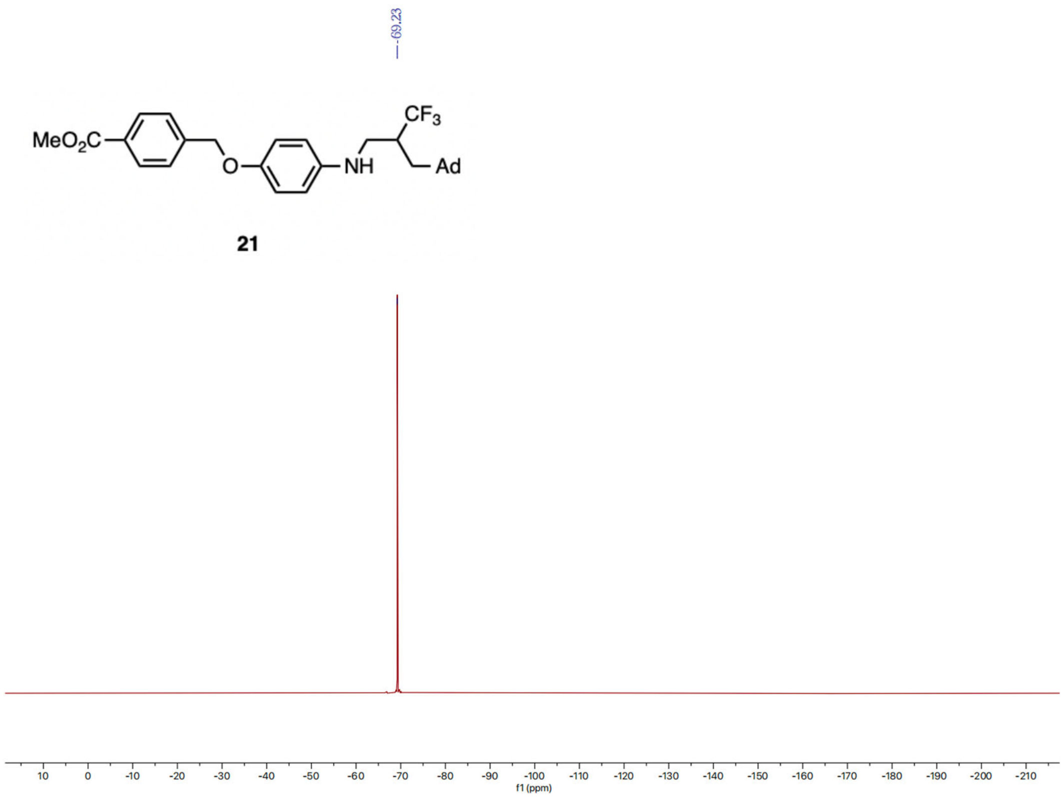


$^{19}$F NMR spectrum (376 MHz, Chloroform-*d*) of compound **21**

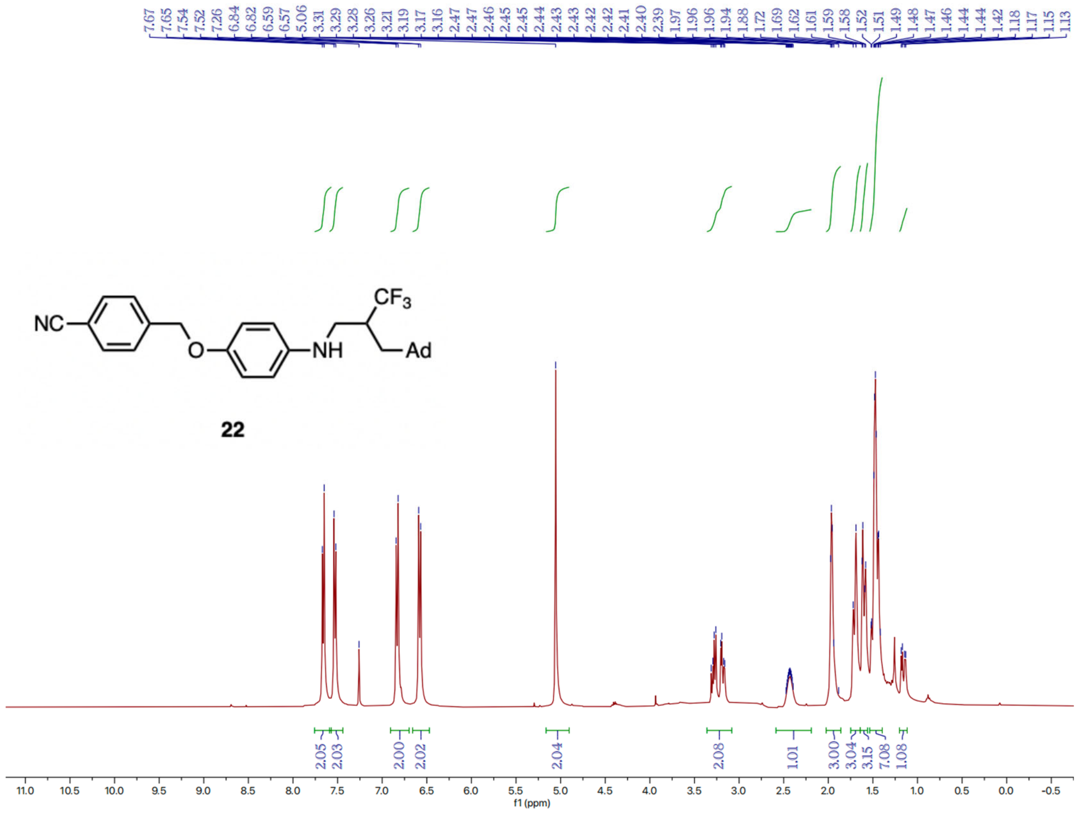

$^{1}$H NMR spectrum (400 MHz, Chloroform-*d*) of compound **22**

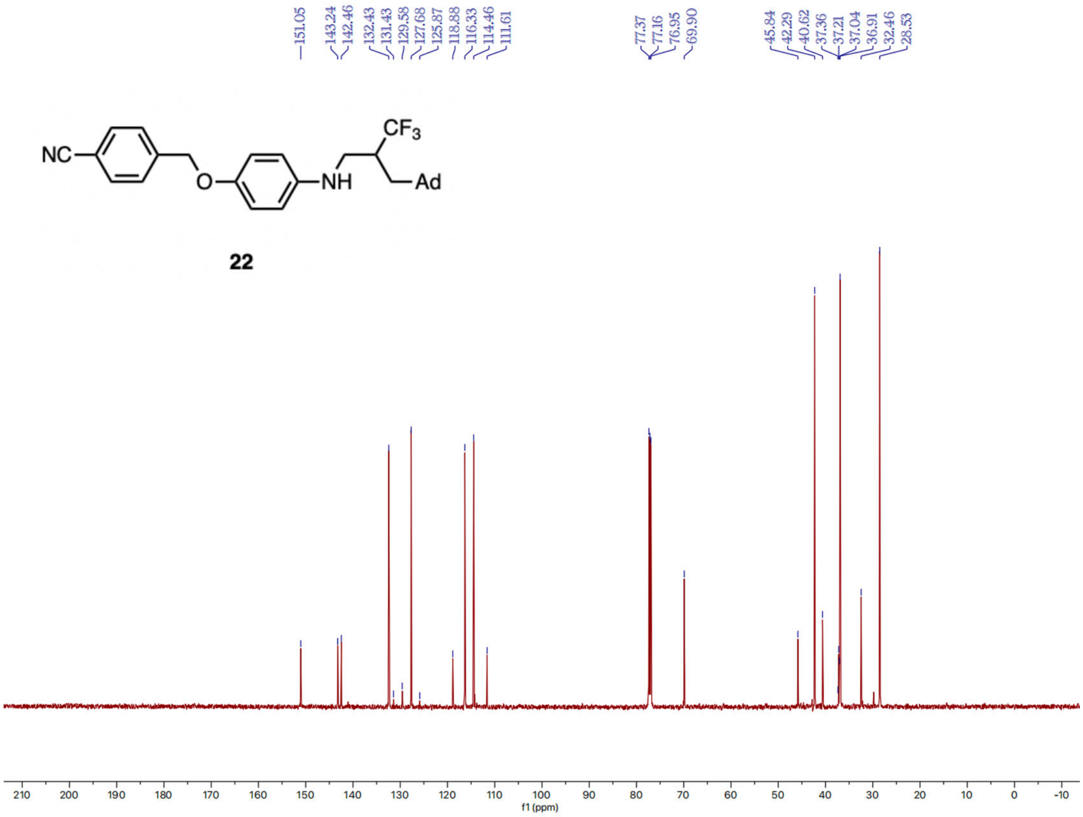

$^{13}$C NMR spectrum (151 MHz, Chloroform-*d*) of compound **22**

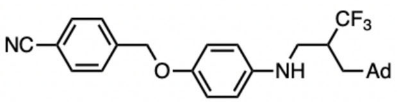


**22**

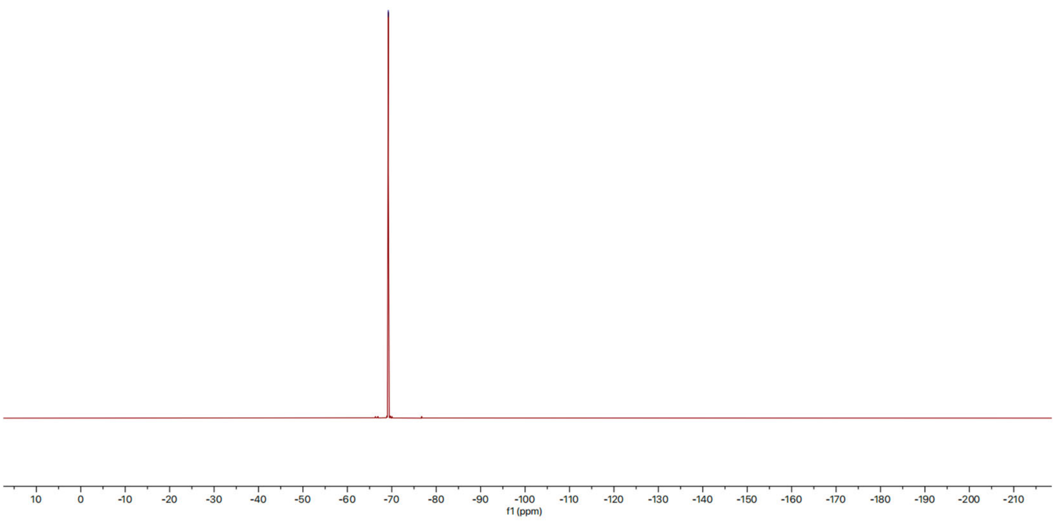


$^{19}$F NMR spectrum (376 MHz, Chloroform-*d*) of compound **22**

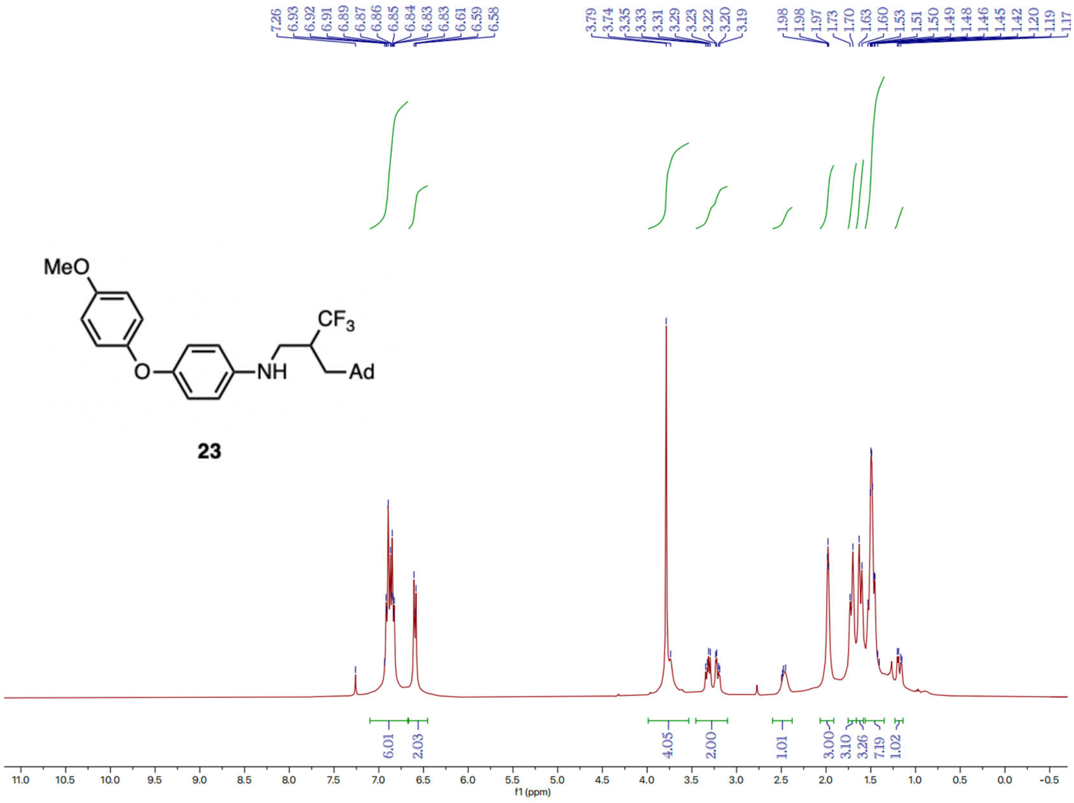

$^{1}$H NMR spectrum (400 MHz, Chloroform-*d*) of compound **23**

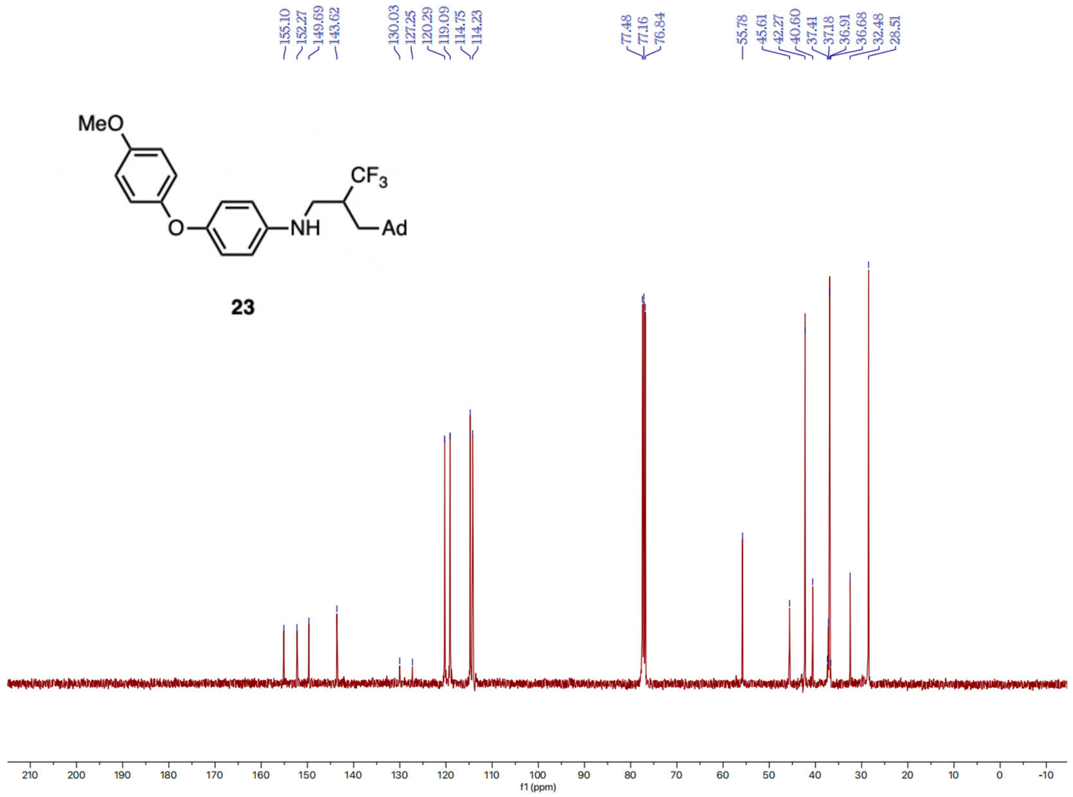

$^{13}$C NMR spectrum (101 MHz, Chloroform-*d*) of compound **23**

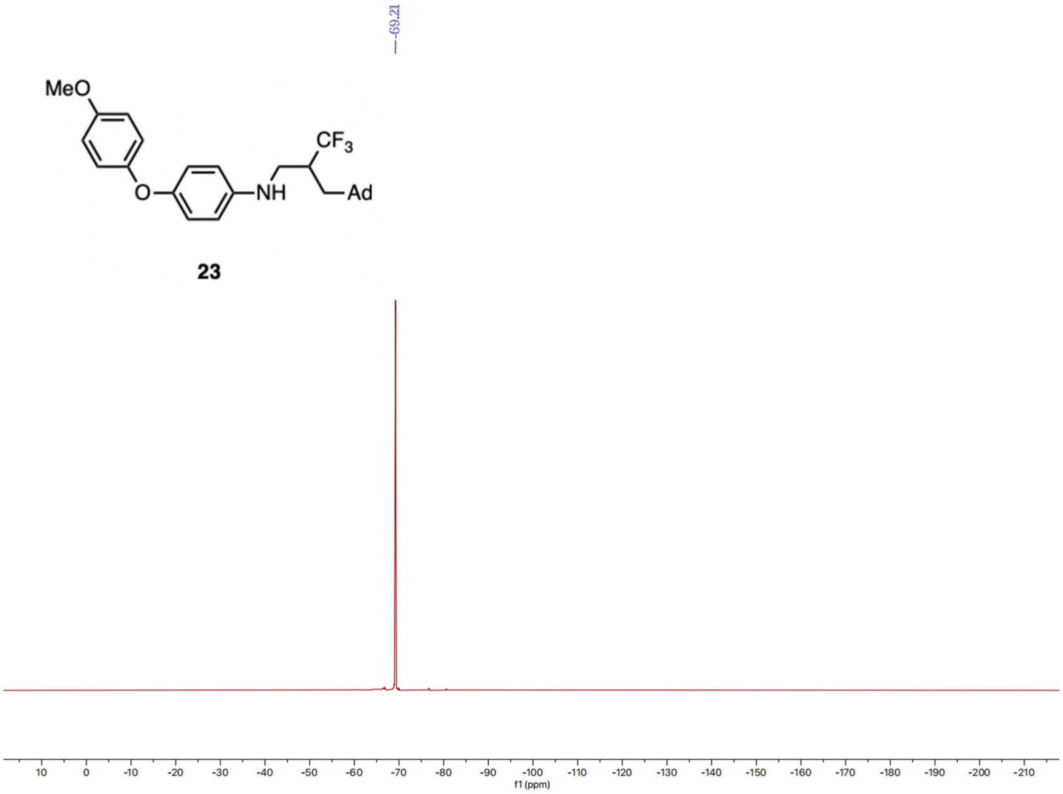


$^{19}$F NMR spectrum (376 MHz, Chloroform-*d*) of compound **23**

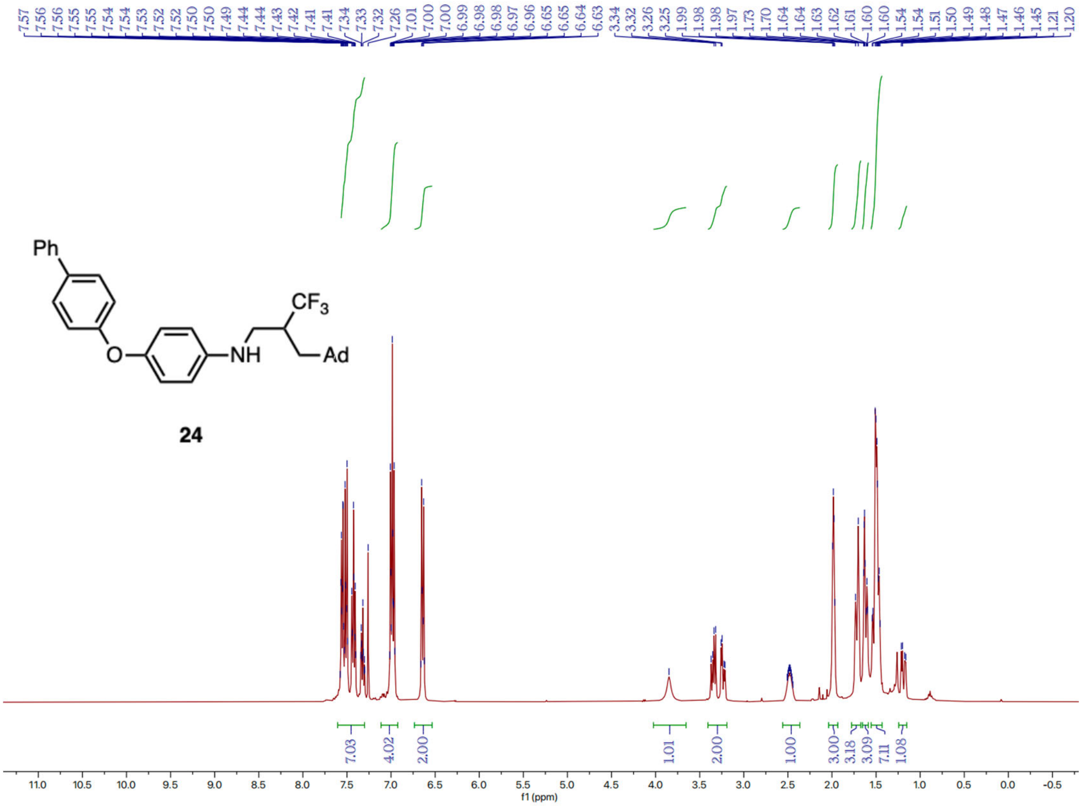


$^{1}$H NMR spectrum (400 MHz, Chloroform-*d*) of compound **24**

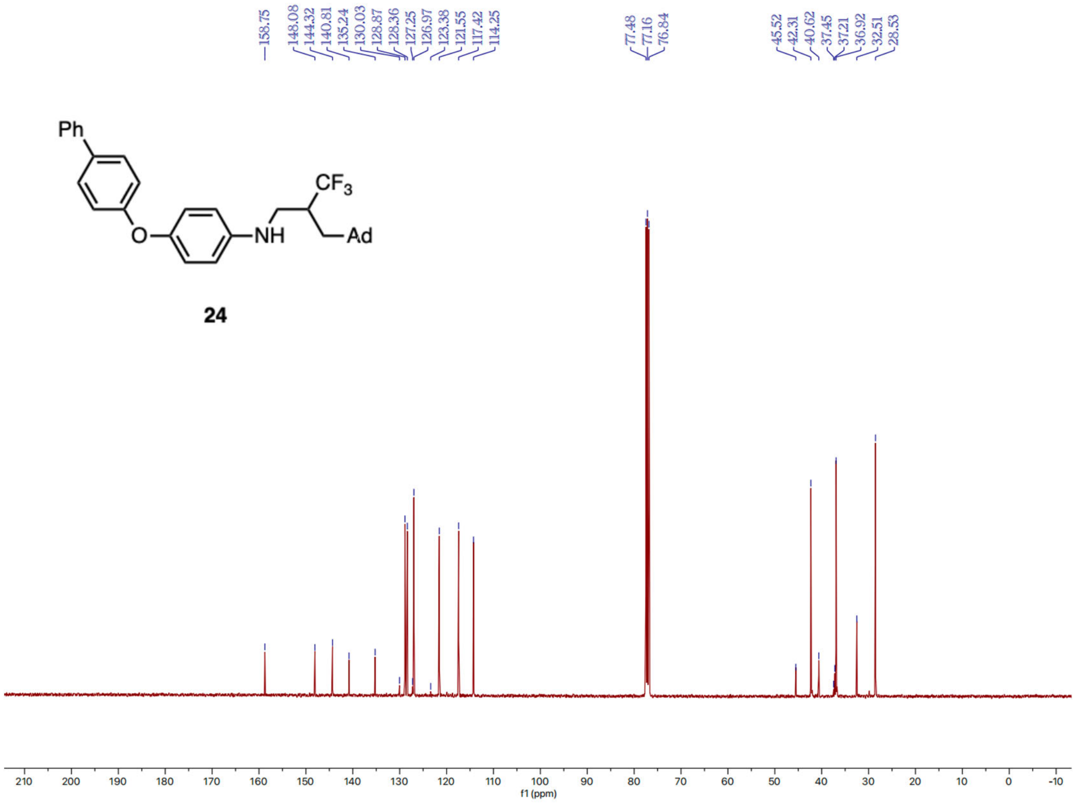


$^{13}$C NMR spectrum (101 MHz, Chloroform-*d*) of compound **24**

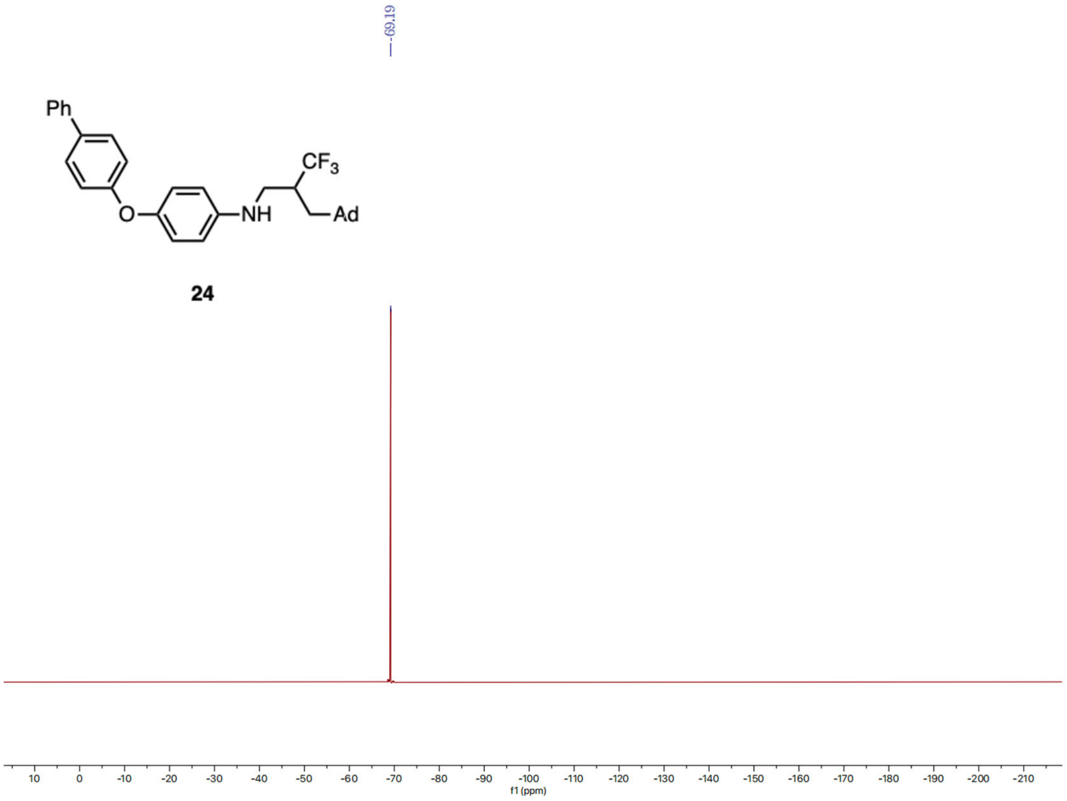


$^{19}$F NMR spectrum (376 MHz, Chloroform-*d*) of compound **24**

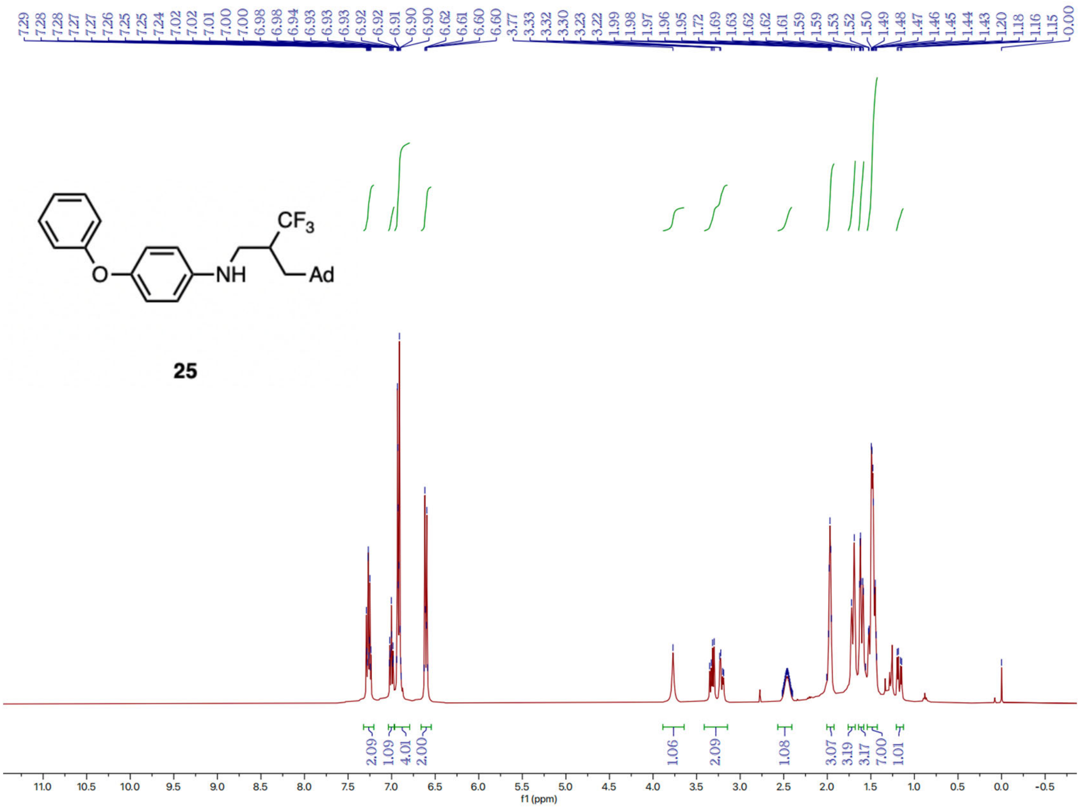

$^{1}H$ NMR spectrum (400 MHz, Chloroform-*d*) of compound **25**

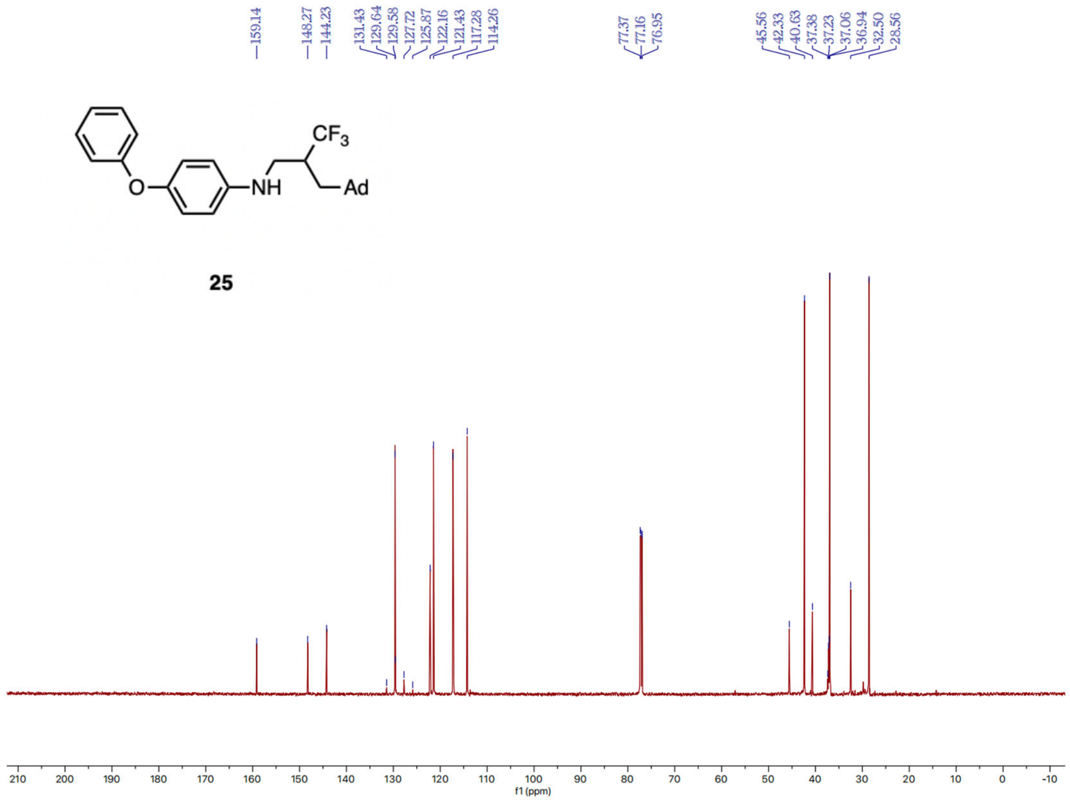

$^{13}C$ NMR spectrum (151 MHz, Chloroform-*d*) of compound **25**

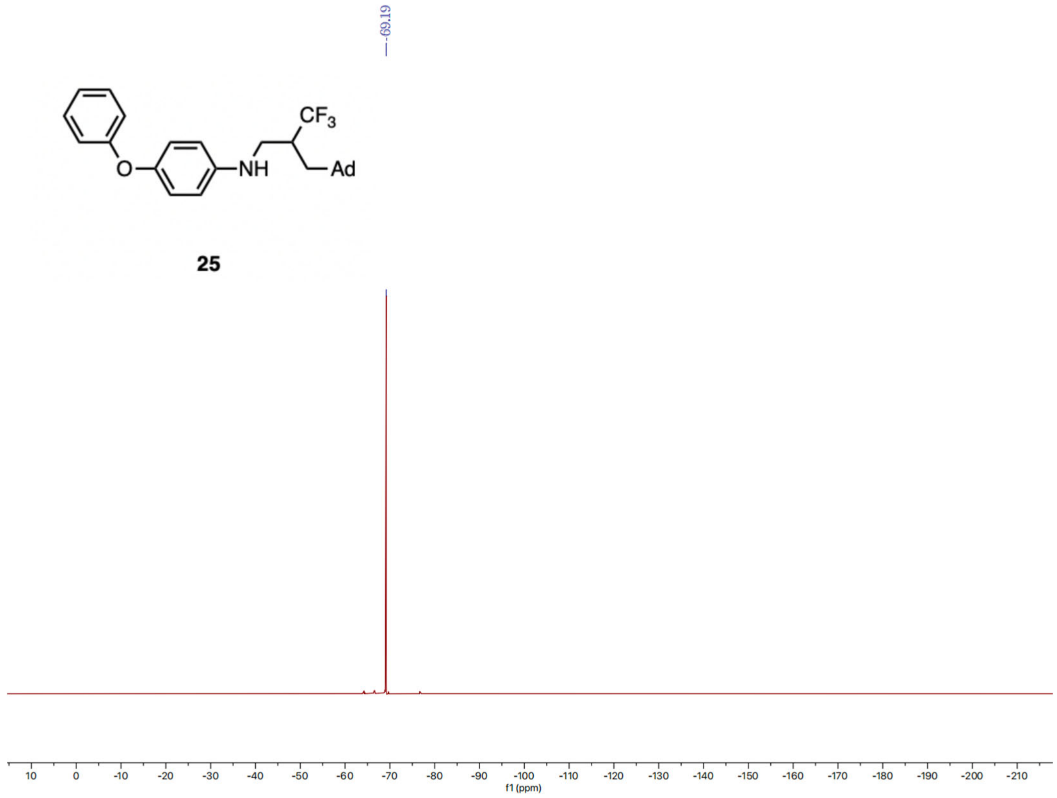


$^{19}$F NMR spectrum (376 MHz, Chloroform-*d*) of compound **25**

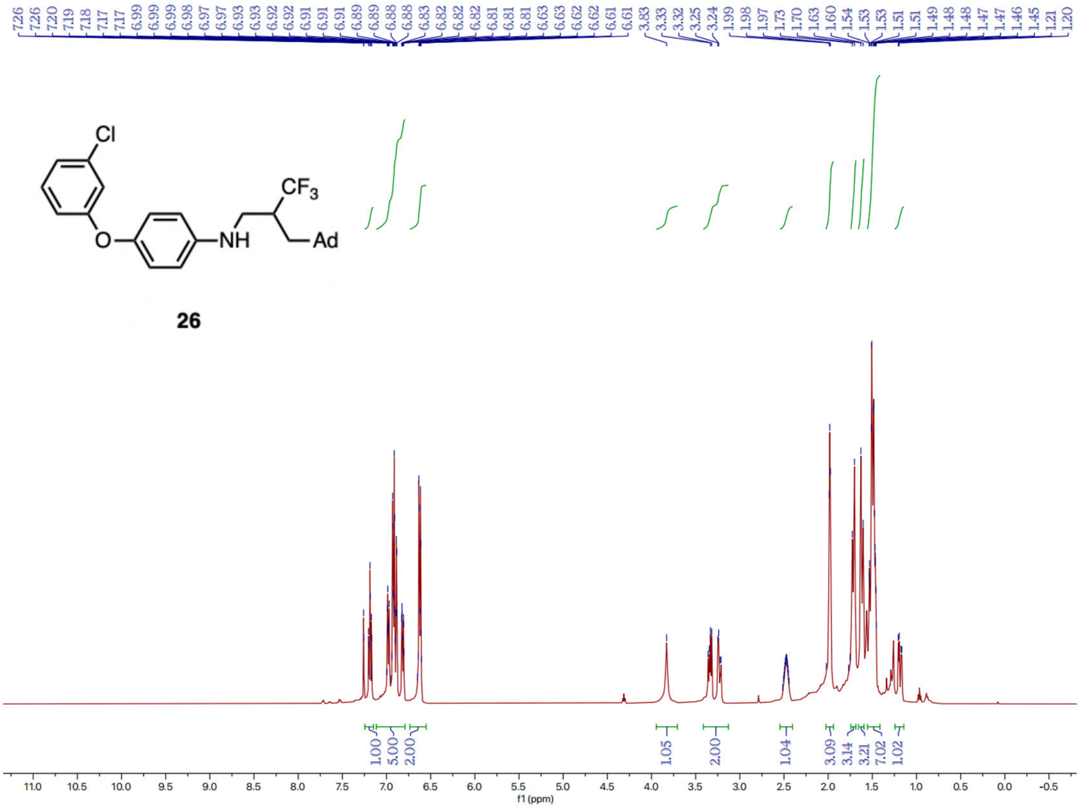

$^{1}$H NMR spectrum (400 MHz, Chloroform-*d*) of compound **26**

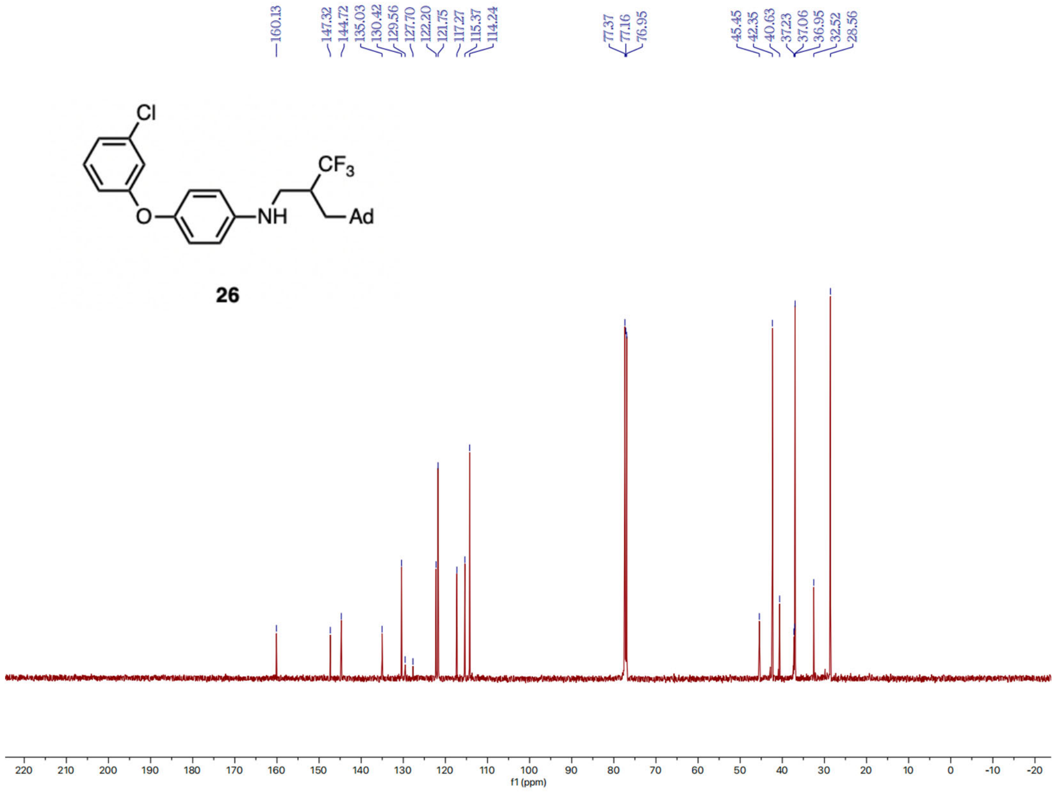

$^{13}$C NMR spectrum (151 MHz, Chloroform-*d*) of compound **26**

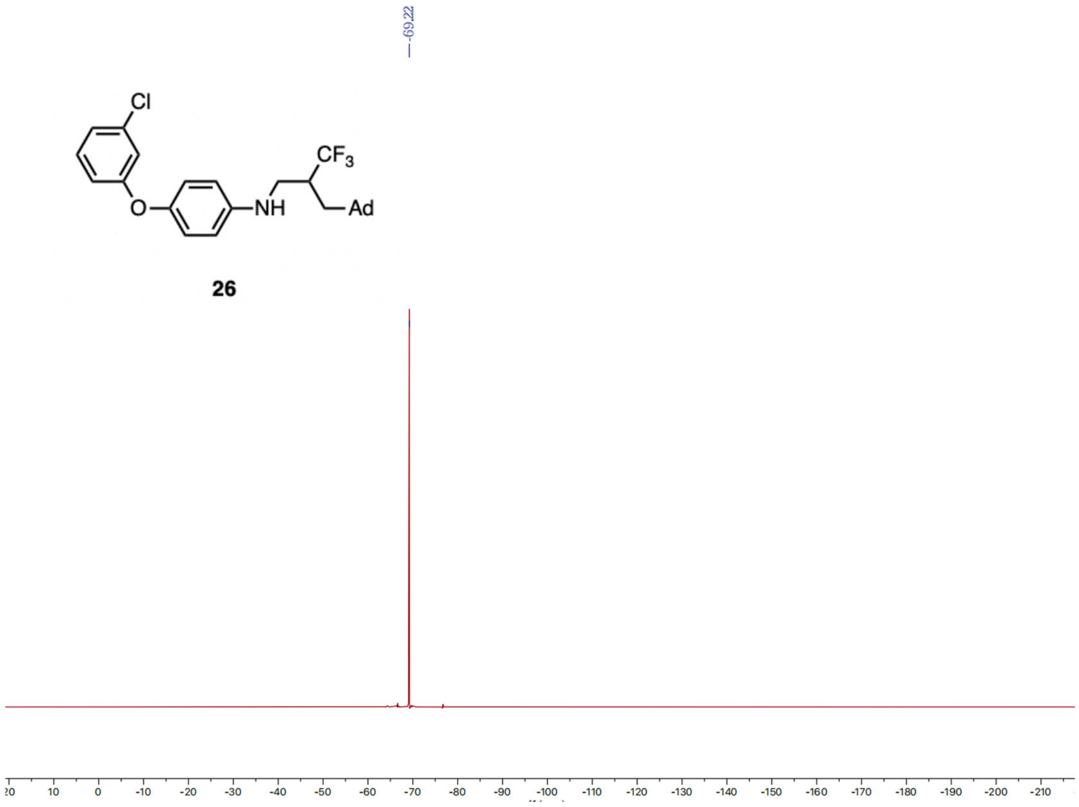


$^{19}F$ NMR spectrum (376 MHz, Chloroform-*d*) of compound **26**

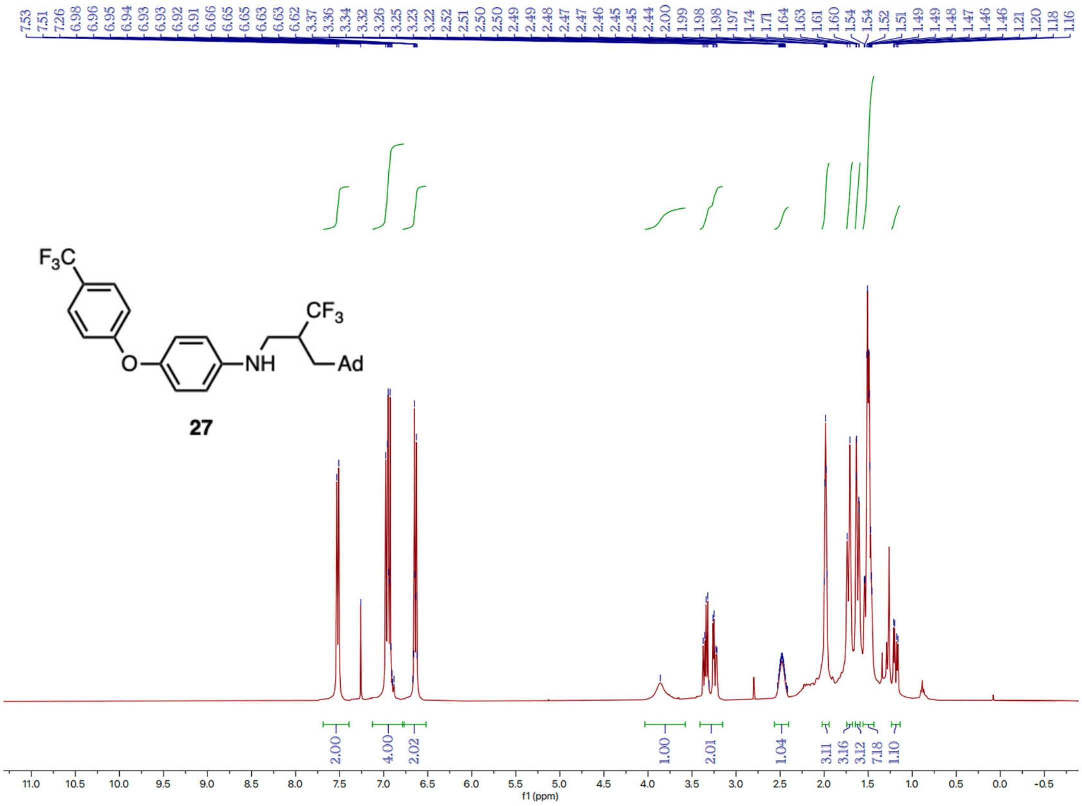


$^1$H NMR spectrum (400 MHz, Chloroform-*d*) of compound **27**

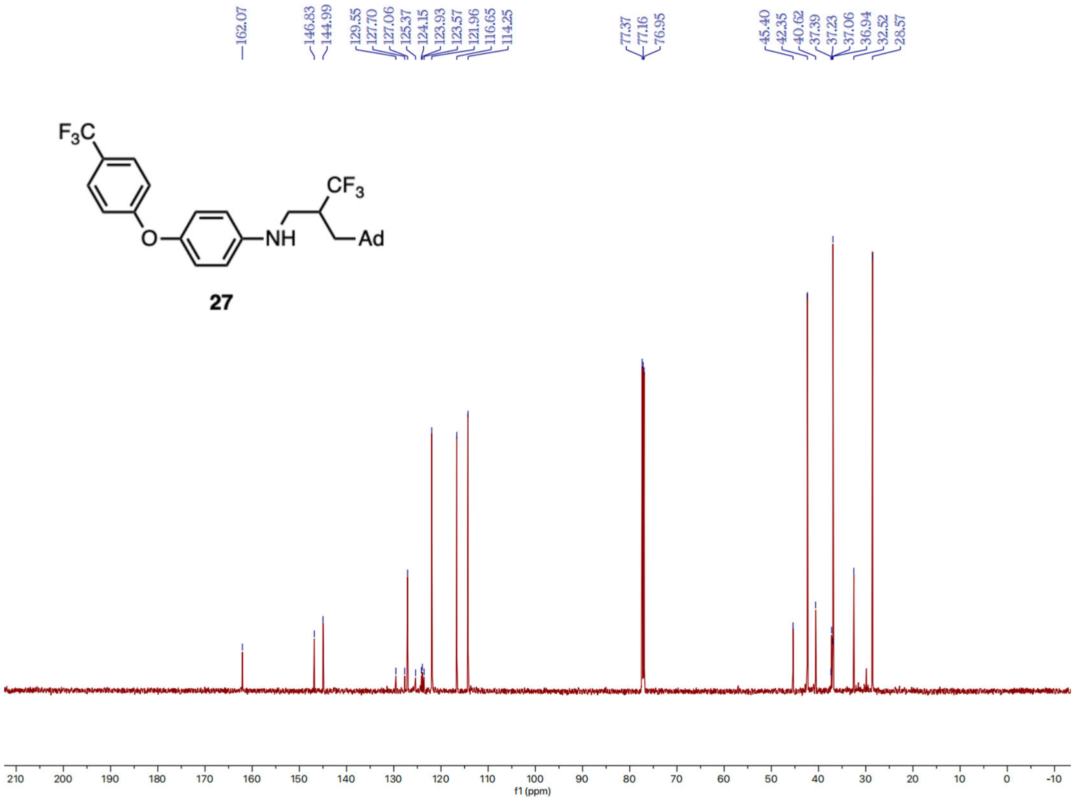


$^{13}$C NMR spectrum (151 MHz, Chloroform-*d*) of compound **27**

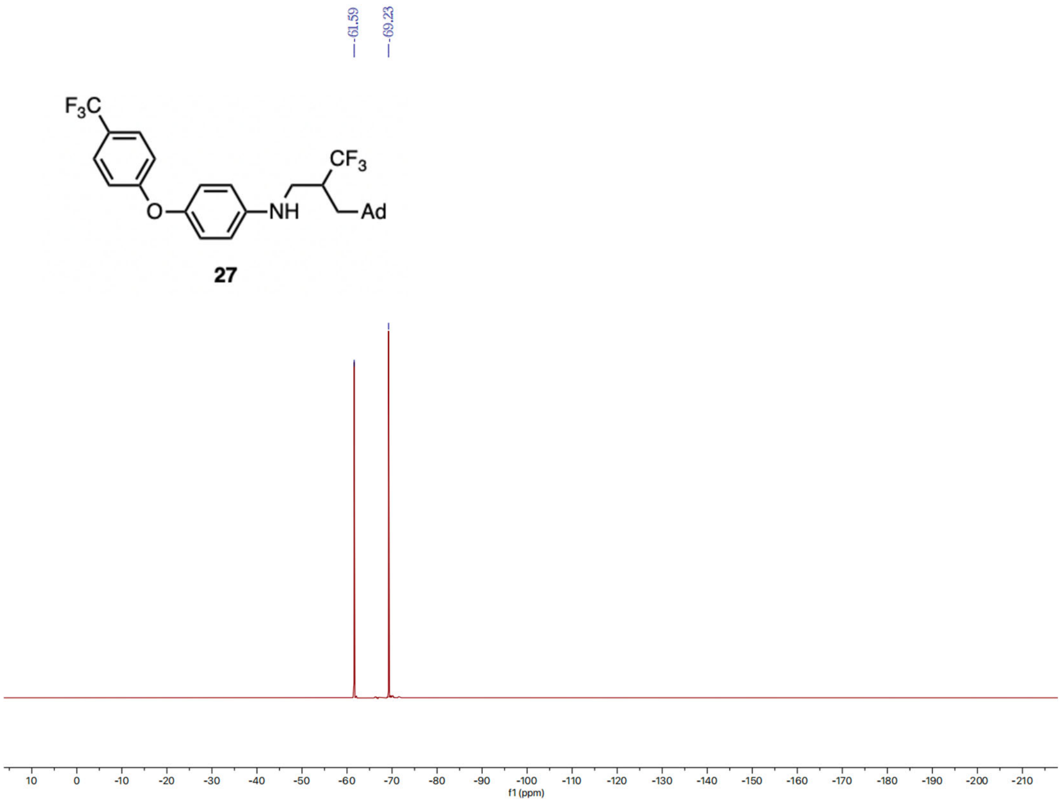


$^{19}$F NMR spectrum (376 MHz, Chloroform-*d*) of compound **27**

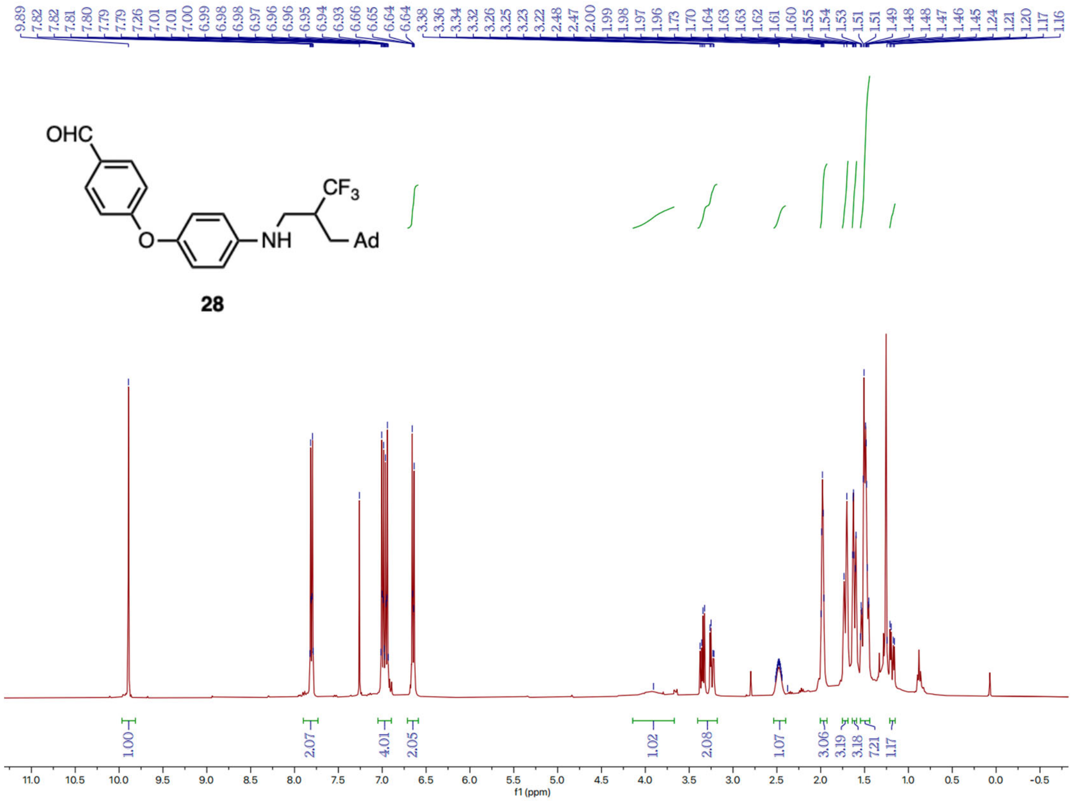


$^{1}$H NMR spectrum (400 MHz, Chloroform-*d*) of compound **28**

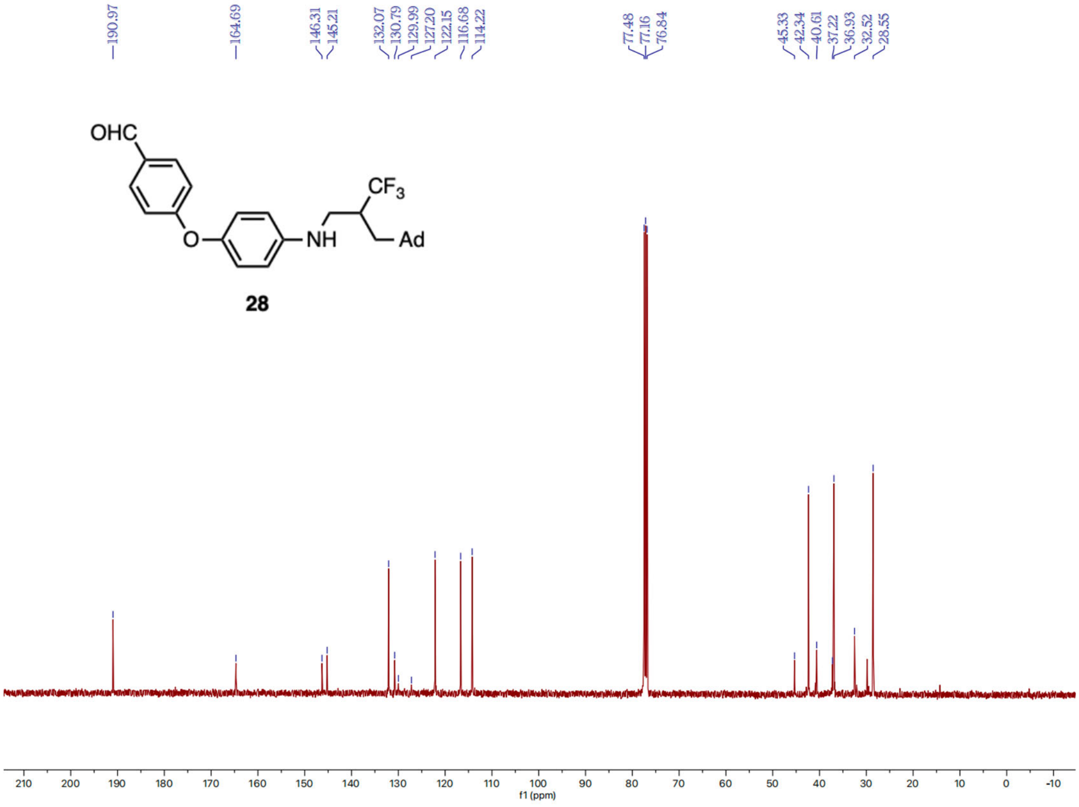


$^{13}$C NMR spectrum (101 MHz, Chloroform-*d*) of compound **28**

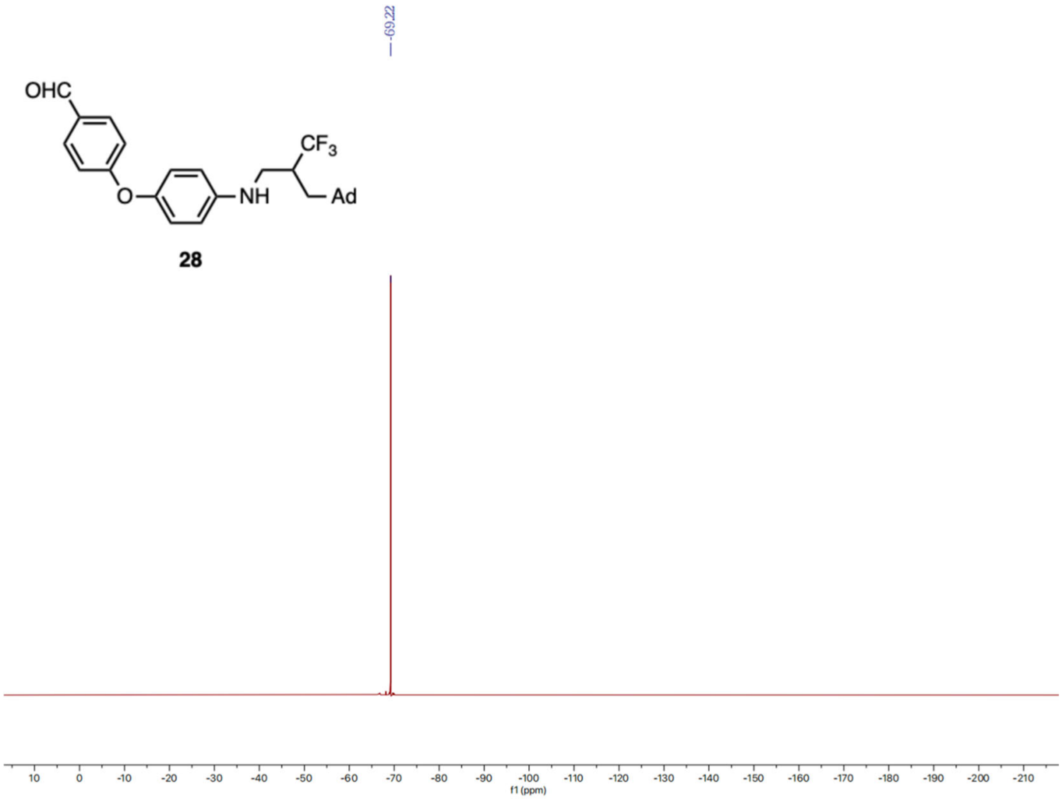


28

$^{19}$F NMR spectrum (376 MHz, Chloroform-*d*) of compound **28**

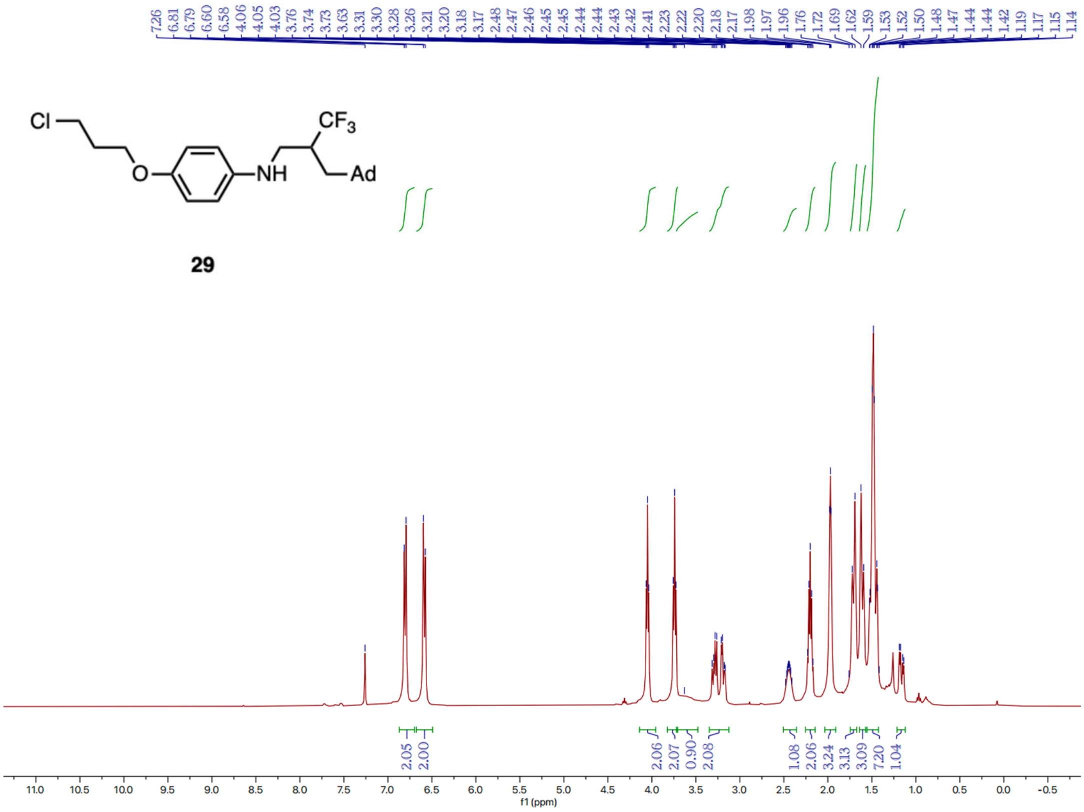


$^{1}$H NMR spectrum (400 MHz, Chloroform-*d*) of compound **29**

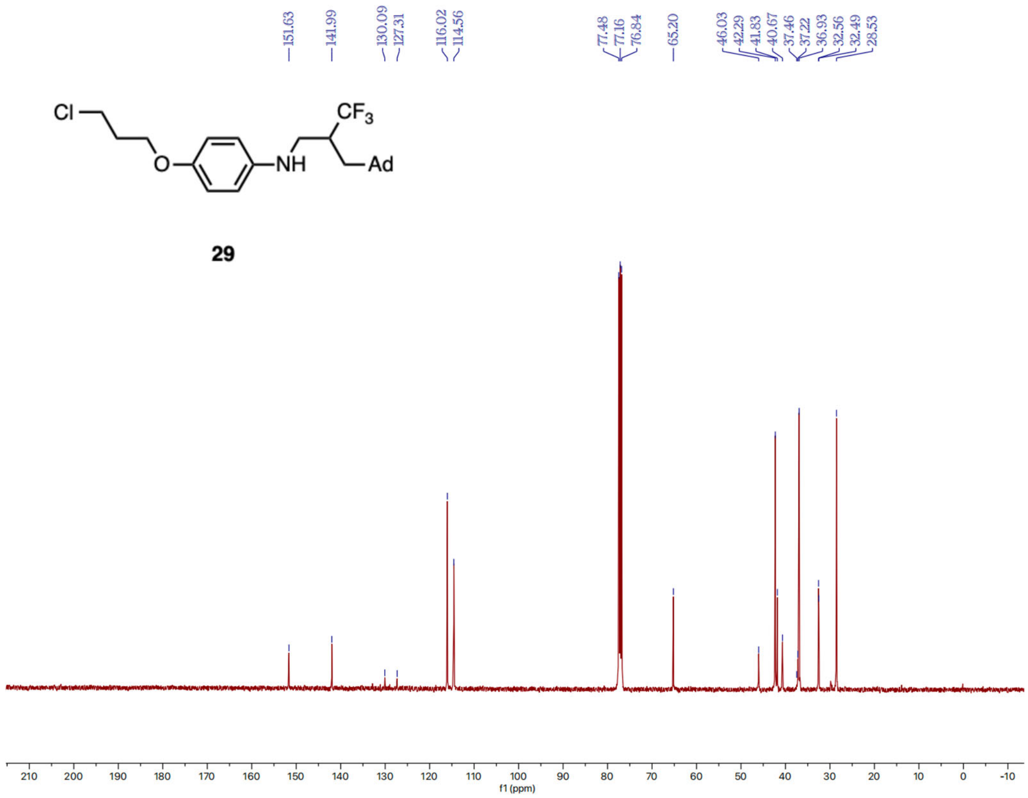


$^{13}$C NMR spectrum (101 MHz, Chloroform-*d*) of compound **29**

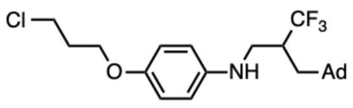


**29**

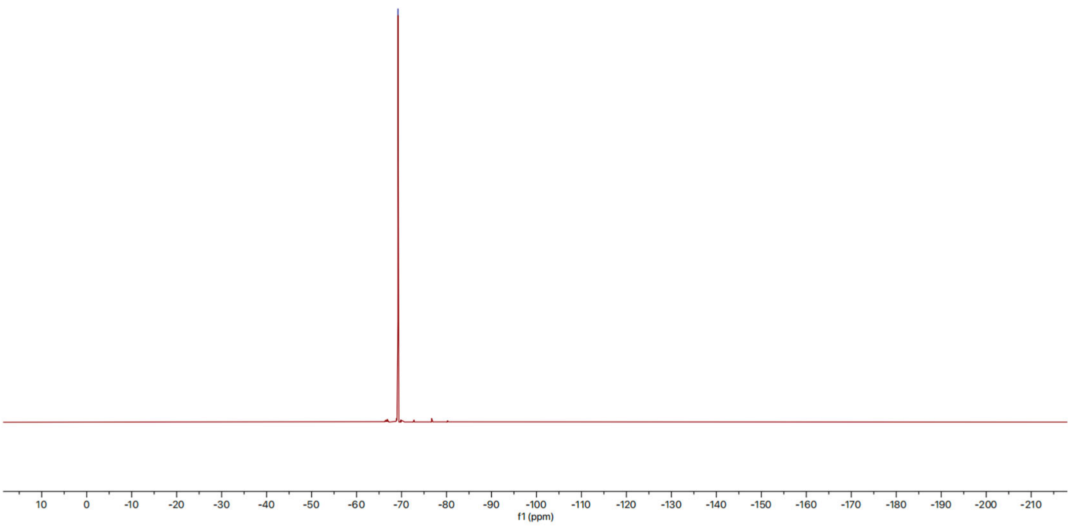


$^{19}F$ NMR spectrum (376 MHz, Chloroform-*d*) of compound **29**

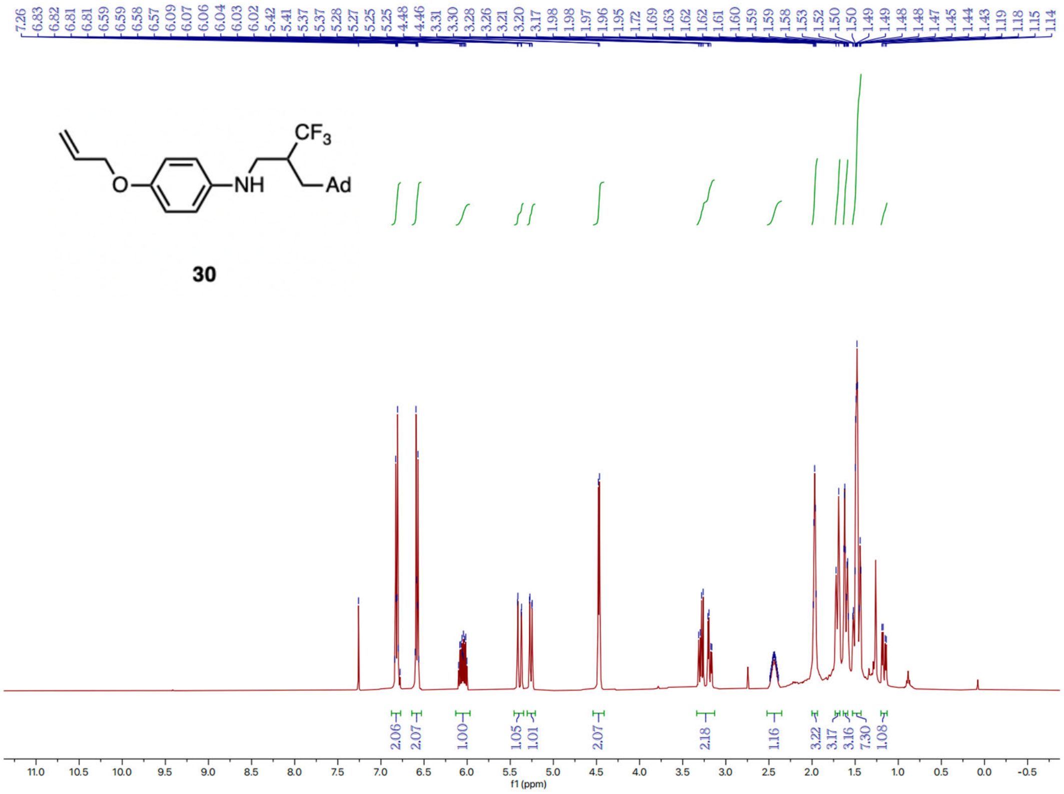


$^{1}$H NMR spectrum (400 MHz, Chloroform-*d*) of compound **30**

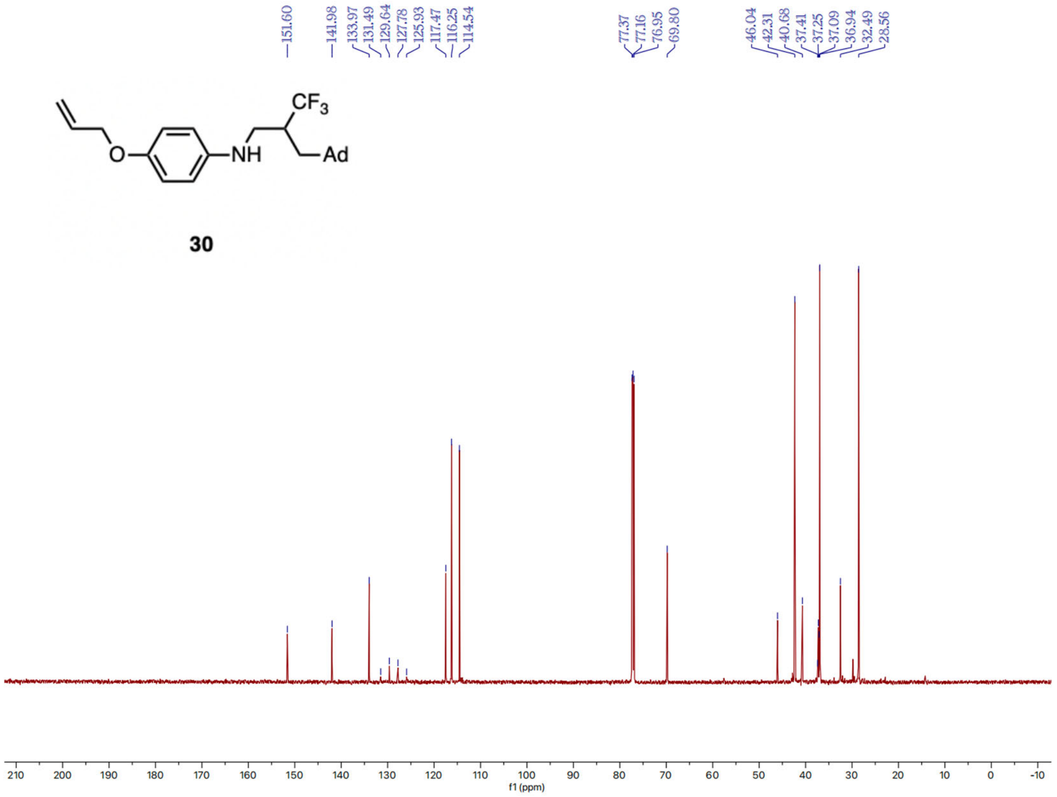


$^{13}$C NMR spectrum (151 MHz, Chloroform-*d*) of compound **30**

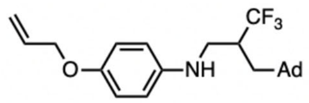

**30**

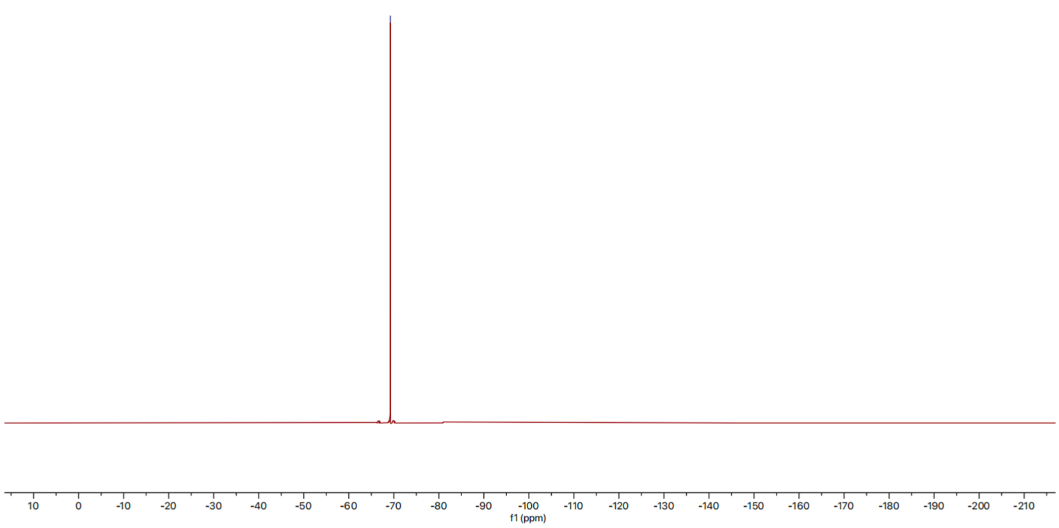


$^{19}$F NMR spectrum (376 MHz, Chloroform-*d*) of compound **30**

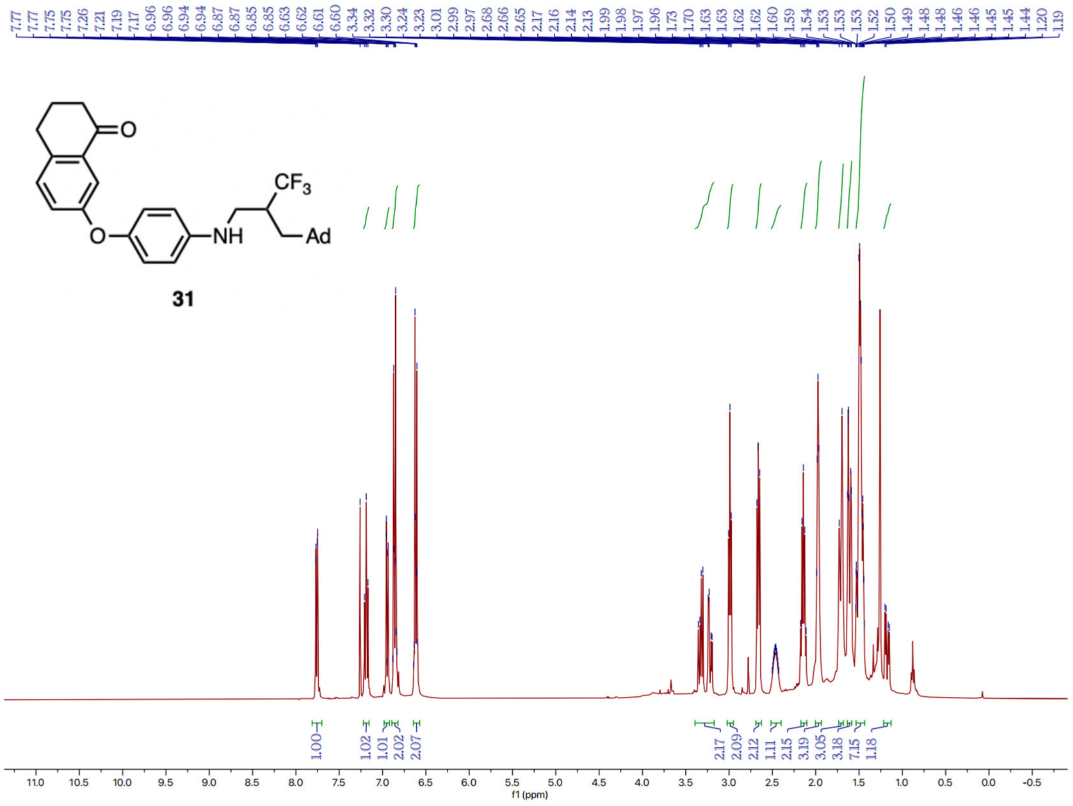



$^{1}$H NMR spectrum (400 MHz, Chloroform-*d*) of compound **31**

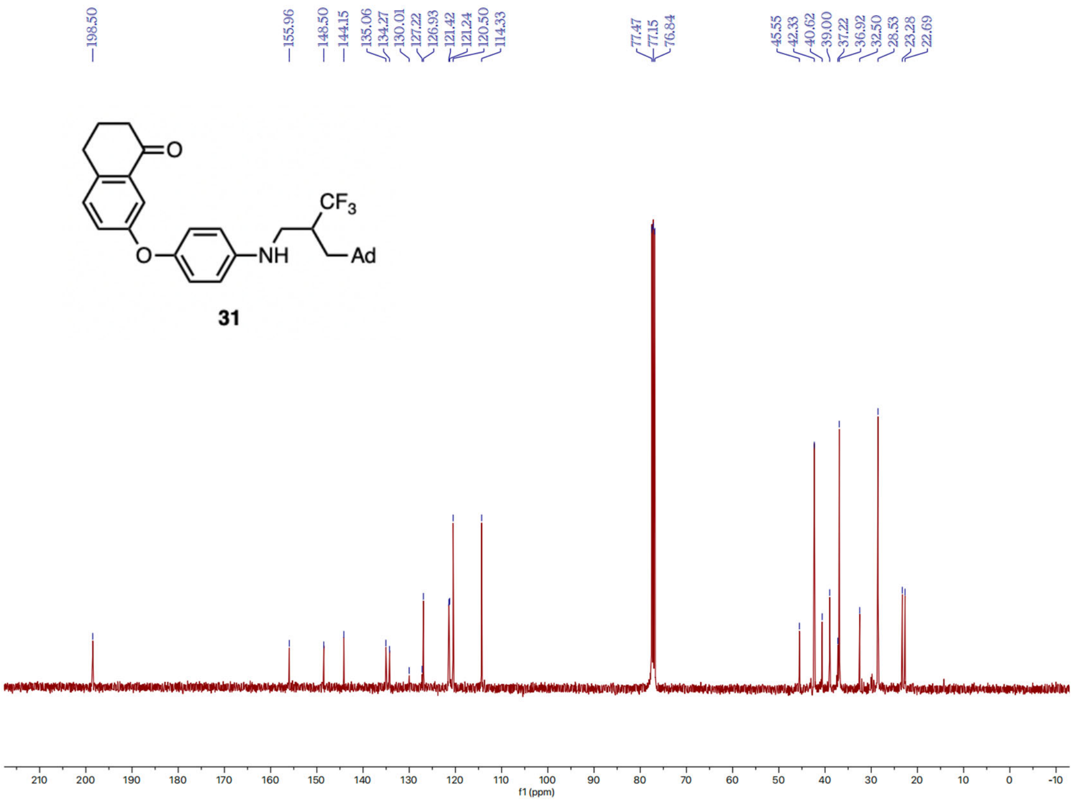



$^{13}$C NMR spectrum (101 MHz, Chloroform-*d*) of compound **31**

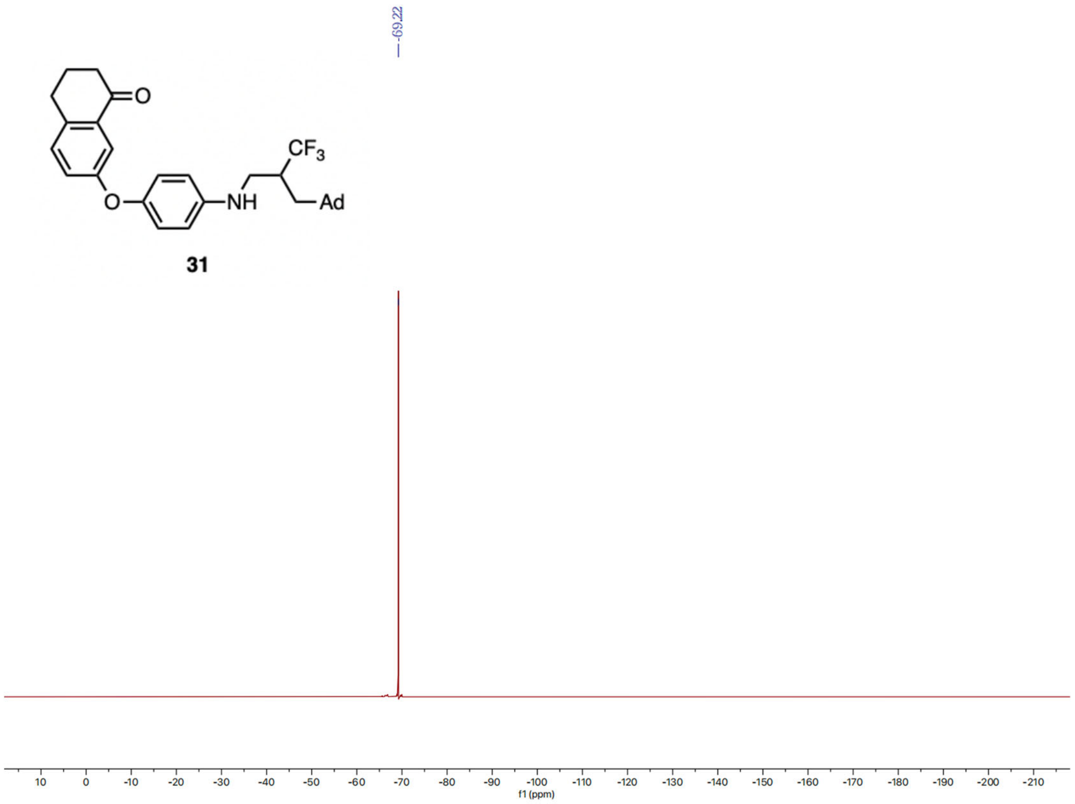


$^{19}F$ NMR spectrum (376 MHz, Chloroform-*d*) of compound **31**

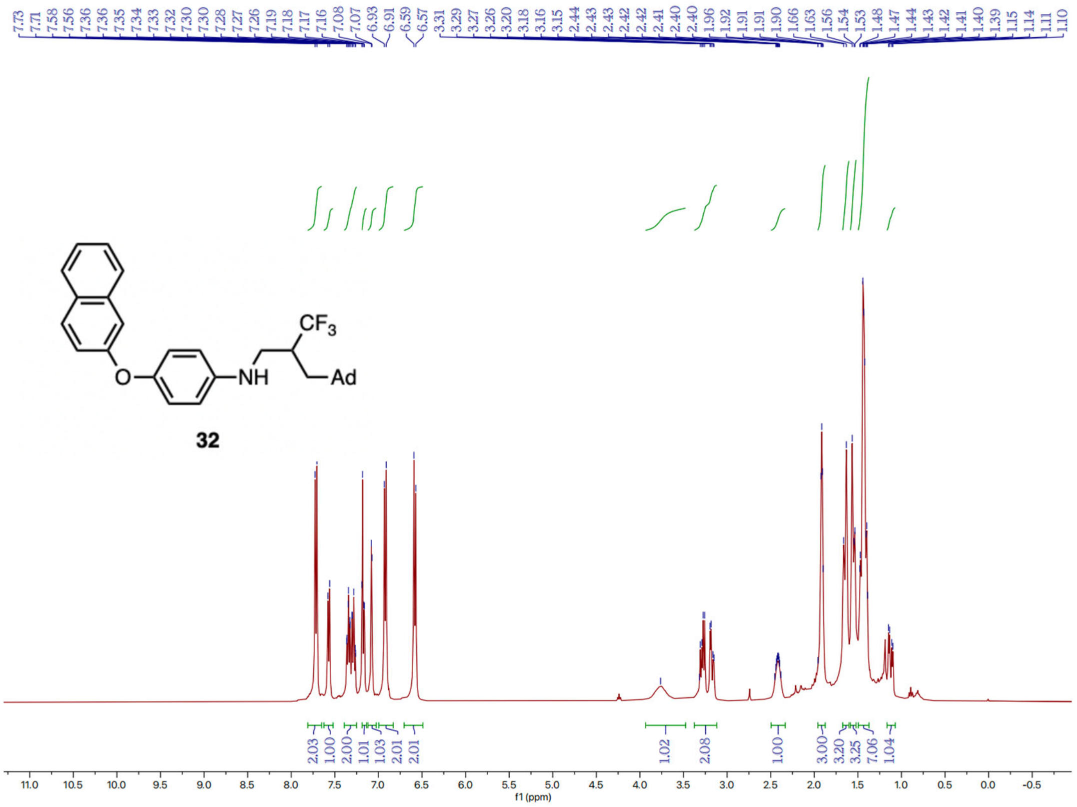

$^{1}H$ NMR spectrum (400 MHz, Chloroform-*d*) of compound **32**

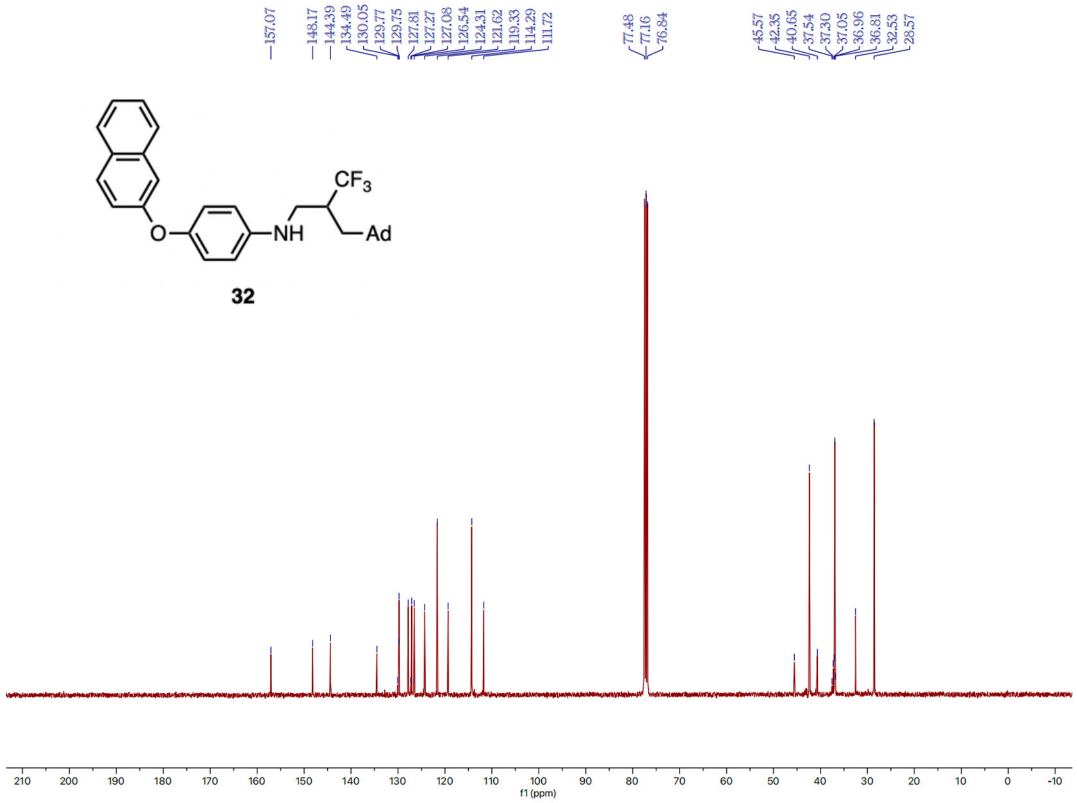

$^{13}C$ NMR spectrum (101 MHz, Chloroform-*d*) of compound **32**

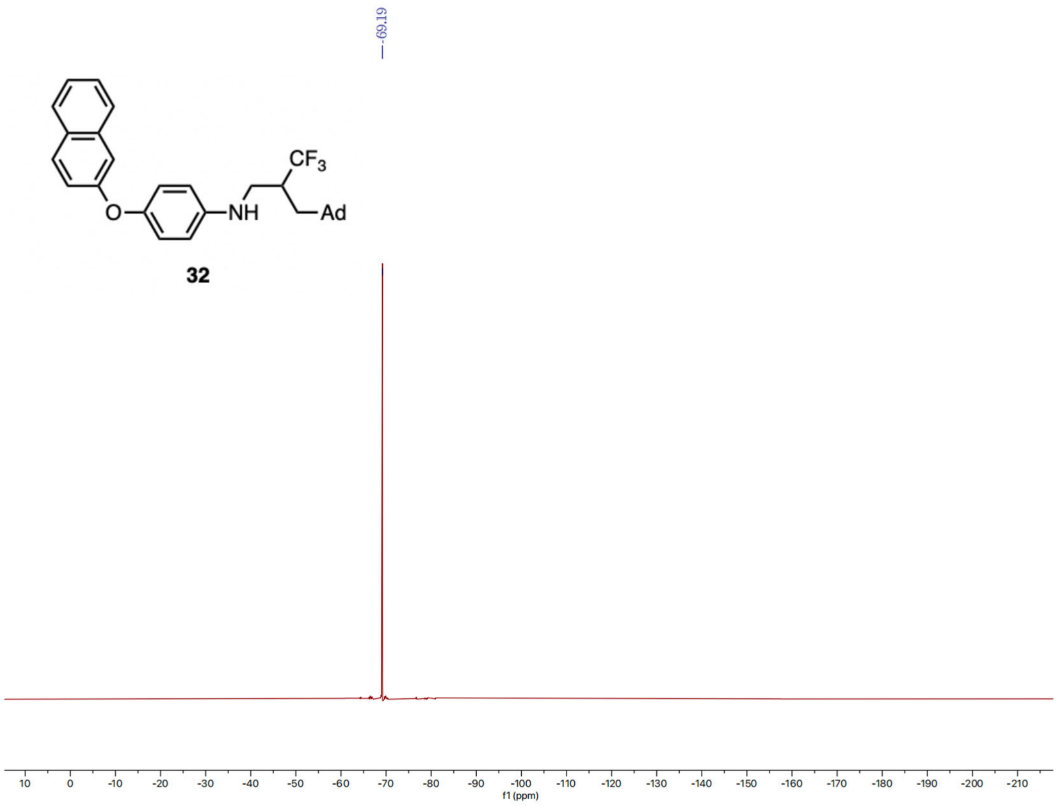


$^{19}$F NMR spectrum (376 MHz, Chloroform-*d*) of compound **32**

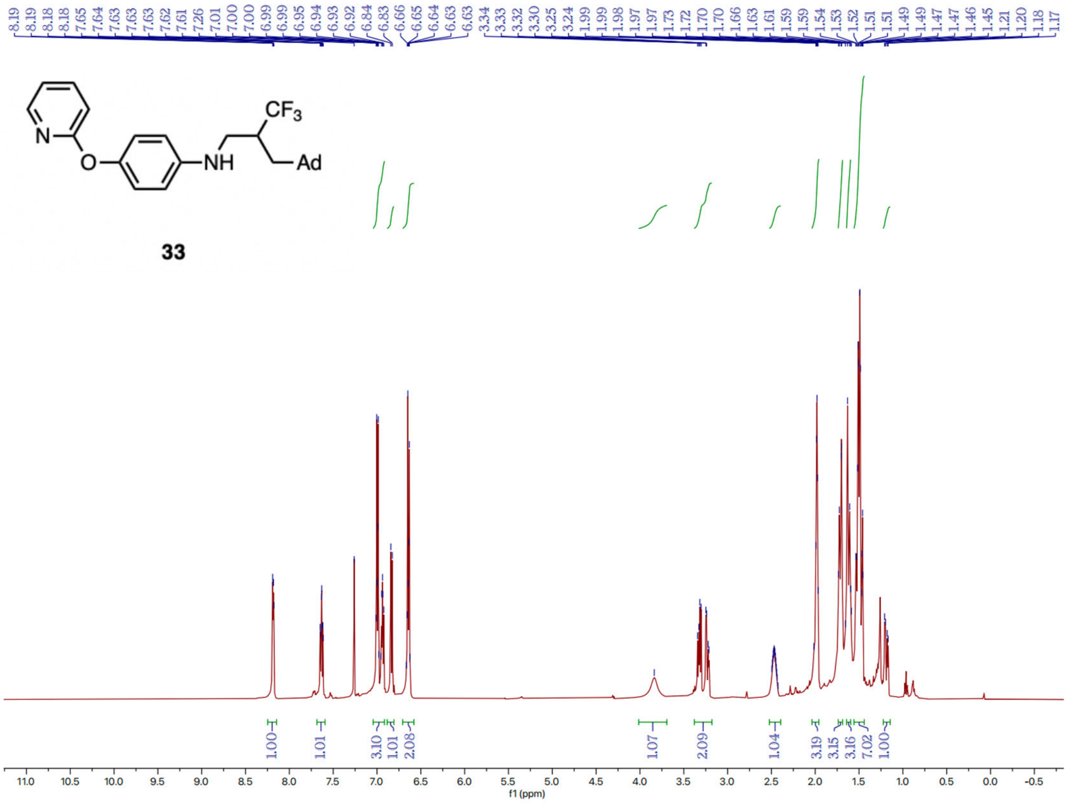

$^{1}$H NMR spectrum (400 MHz, Chloroform-*d*) of compound **33**

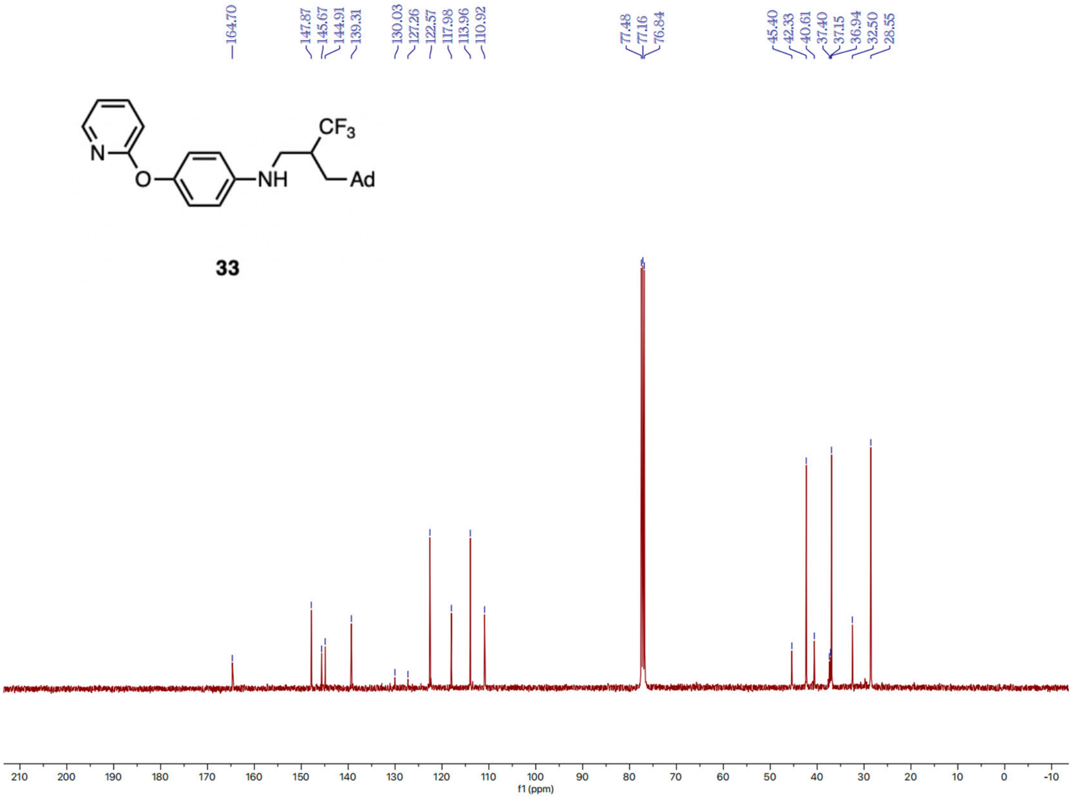

$^{13}$C NMR spectrum (101 MHz, Chloroform-*d*) of compound **33**

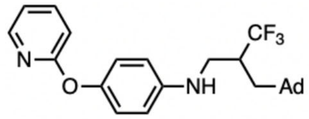


**33**

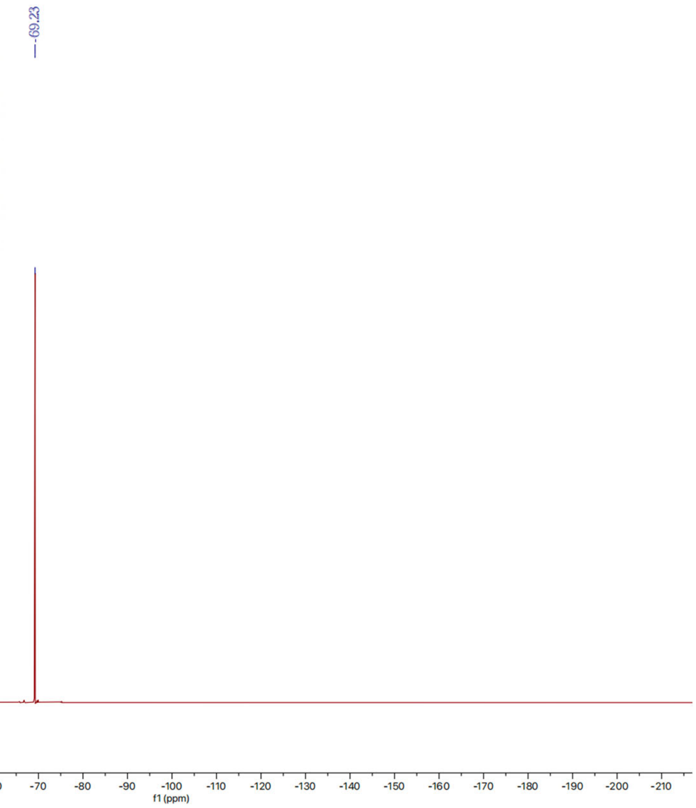


$^{19}$F NMR spectrum (376 MHz, Chloroform-*d*) of compound **33**

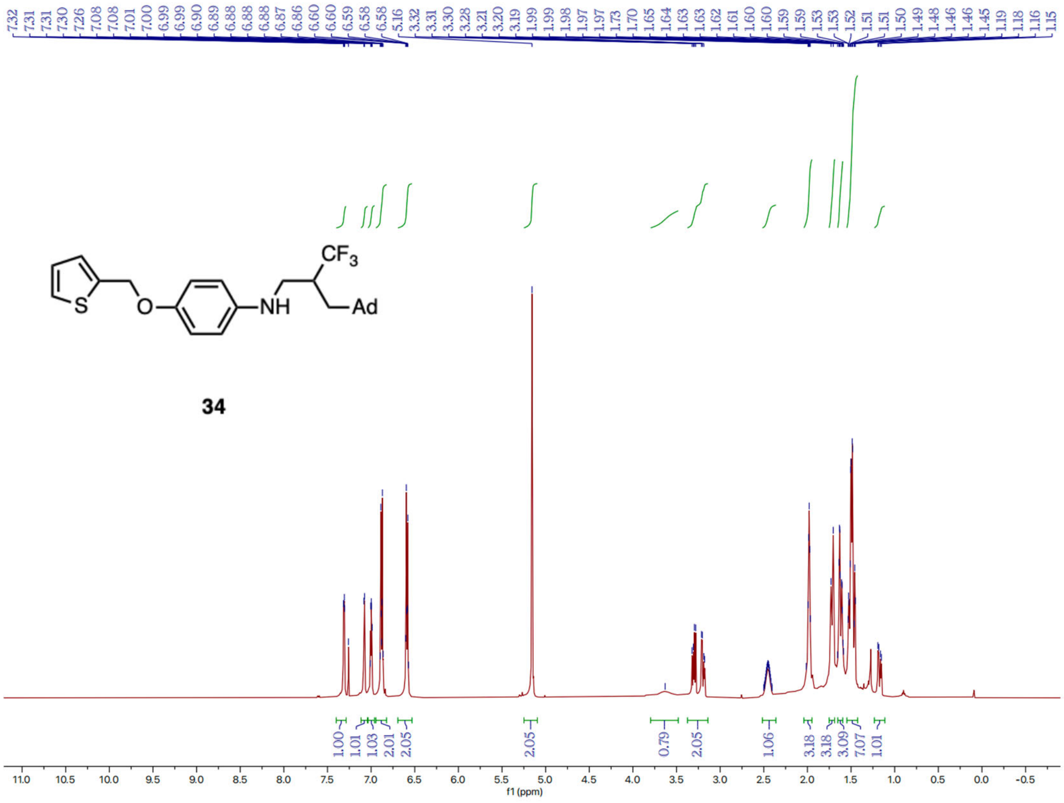

$^{1}H$ NMR spectrum (400 MHz, Chloroform-*d*) of compound **34**

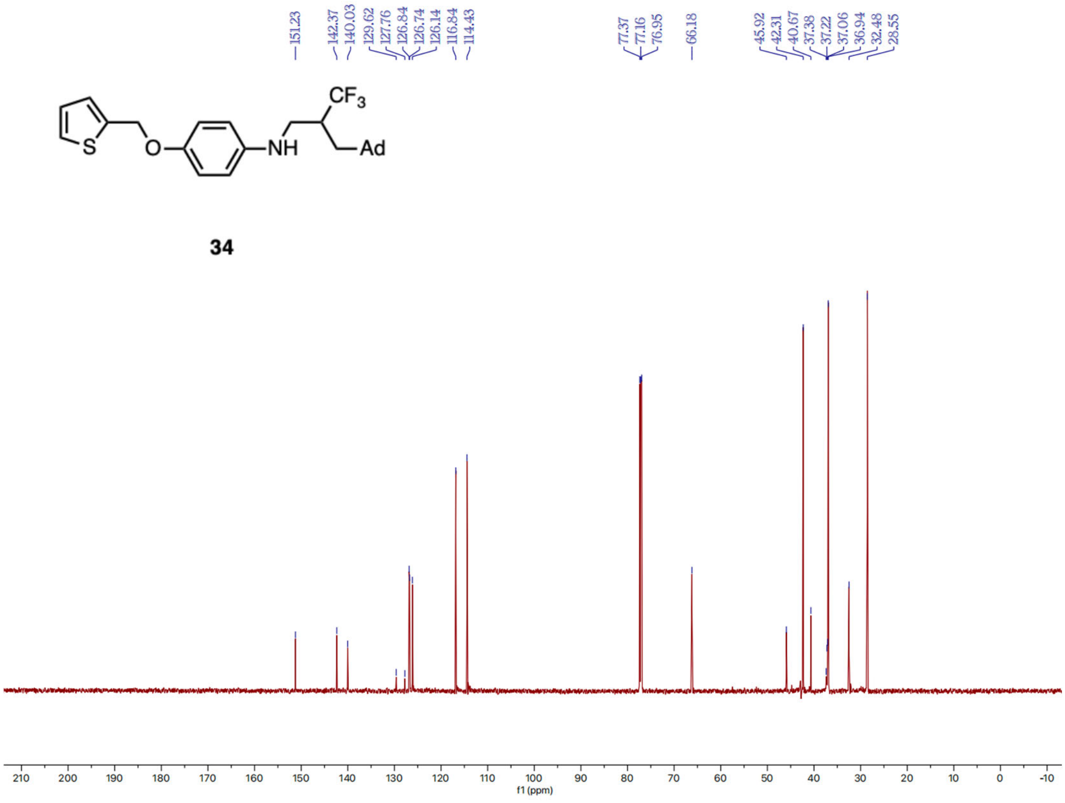

$^{13}C$ NMR spectrum (151 MHz, Chloroform-*d*) of compound **34**

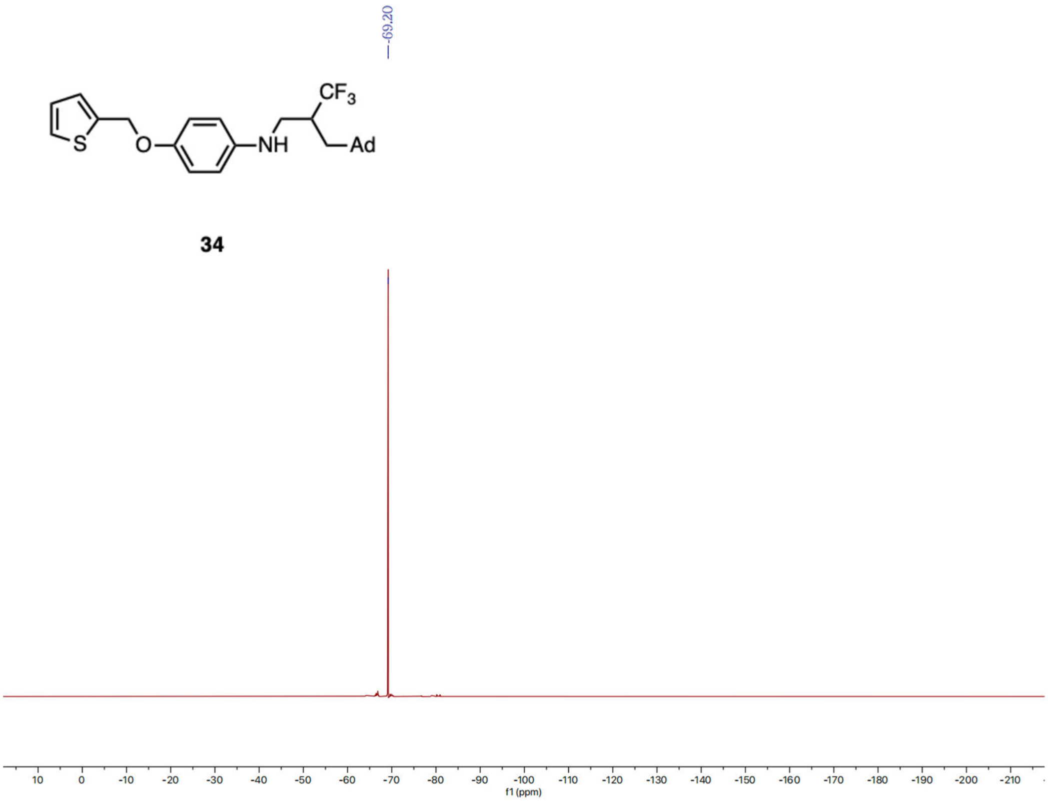


$^{19}$F NMR spectrum (376 MHz, Chloroform-*d*) of compound **34**

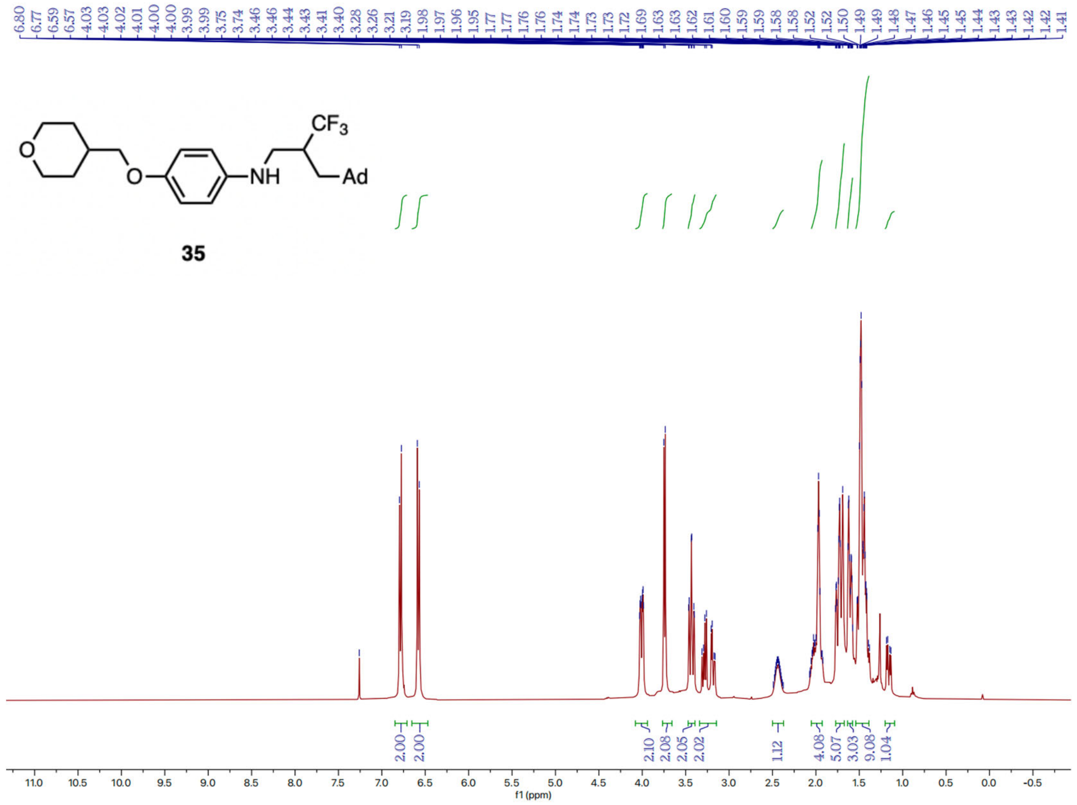


$^{1}$H NMR spectrum (400 MHz, Chloroform-*d*) of compound **35**

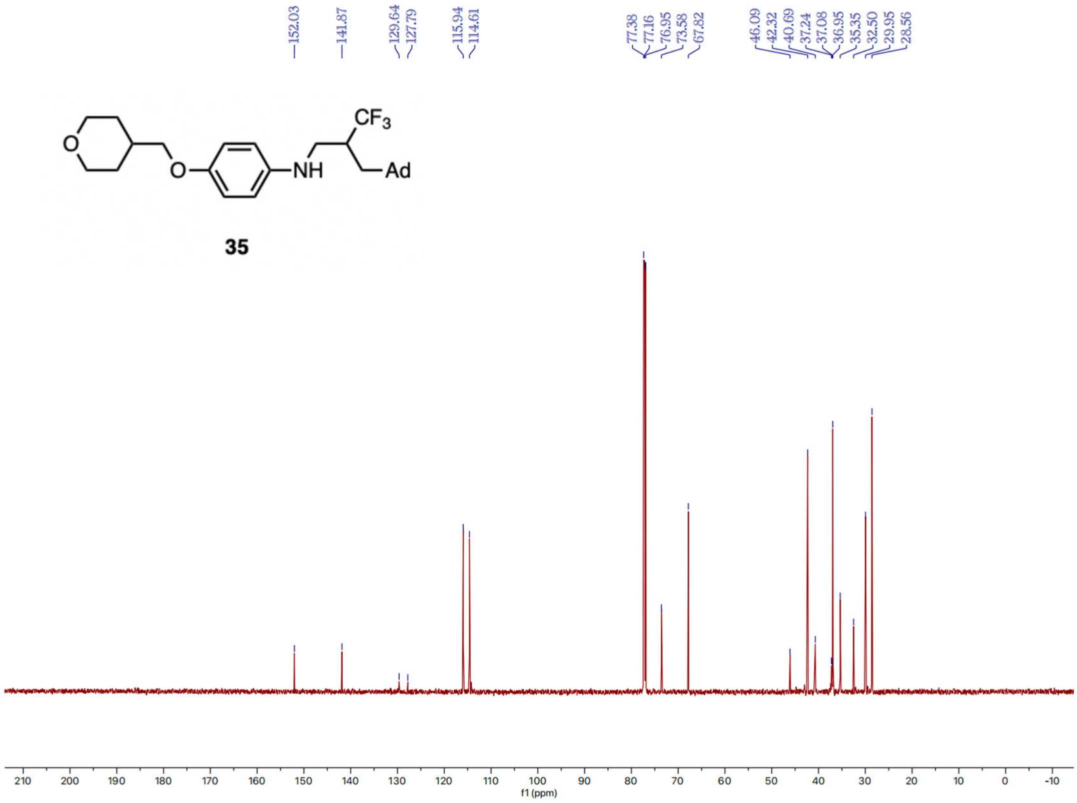


$^{13}$C NMR spectrum (151 MHz, Chloroform-*d*) of compound **35**

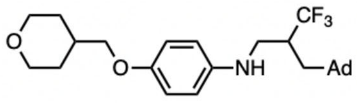

**35**

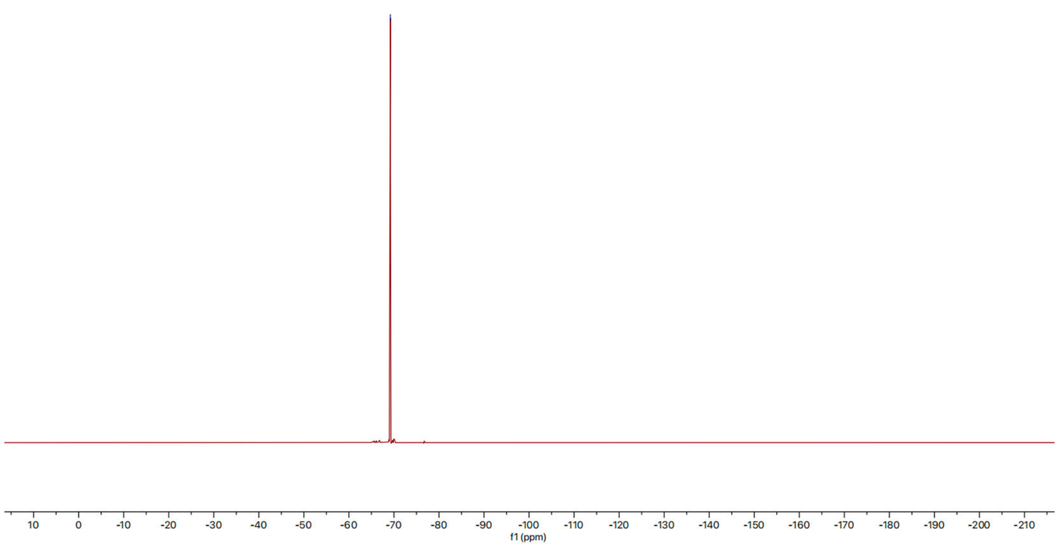


$^{19}F$ NMR spectrum (376 MHz, Chloroform-*d*) of compound **35**

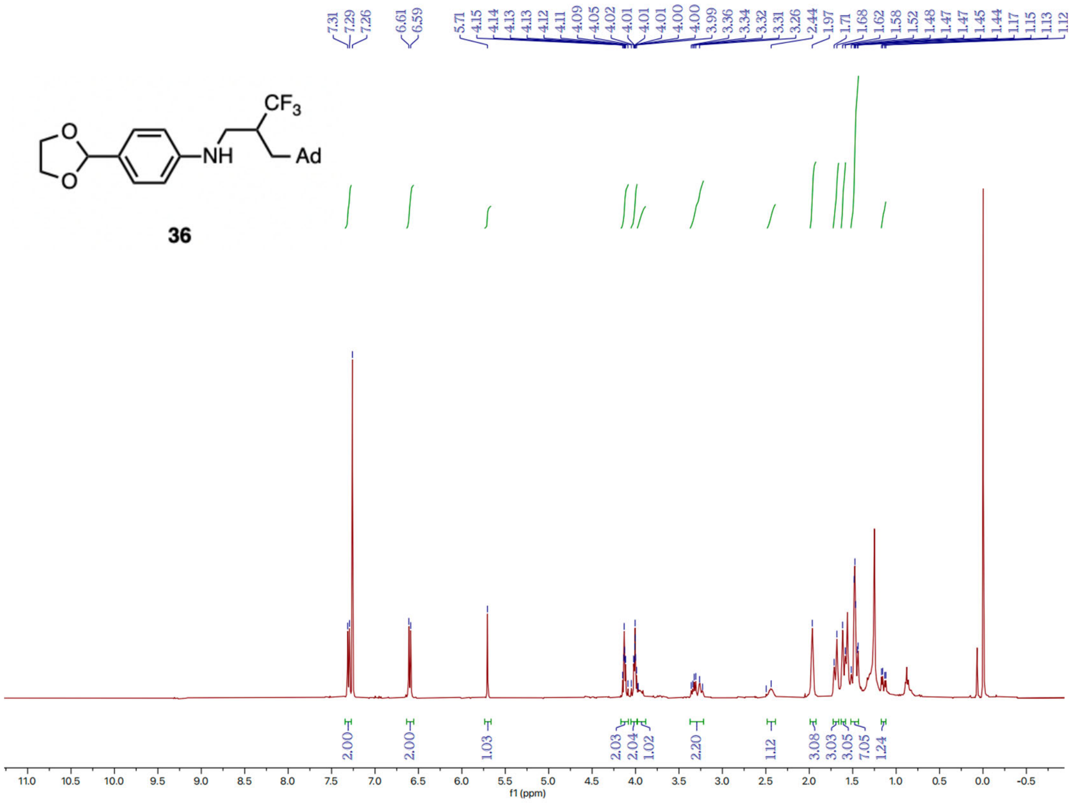

$^1$H NMR spectrum (400 MHz, Chloroform-*d*) of compound **36**

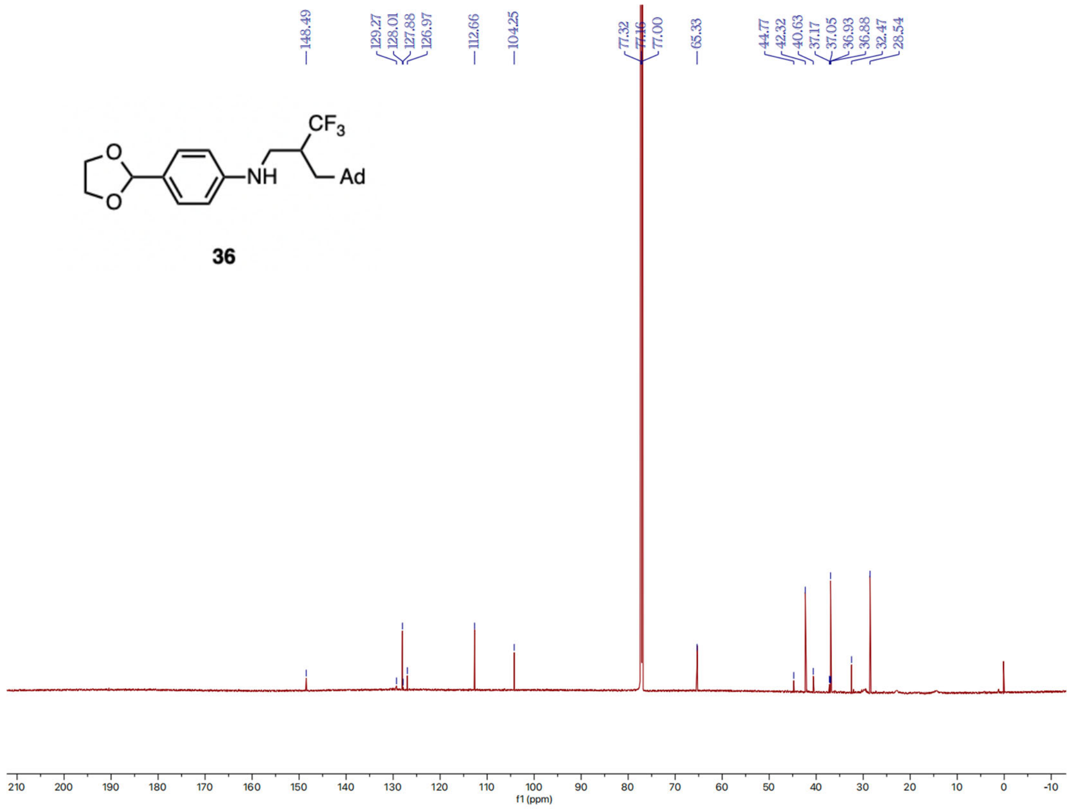

$^{13}$C NMR spectrum (201 MHz, Chloroform-*d*) of compound **36**

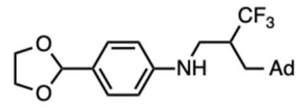


**36**

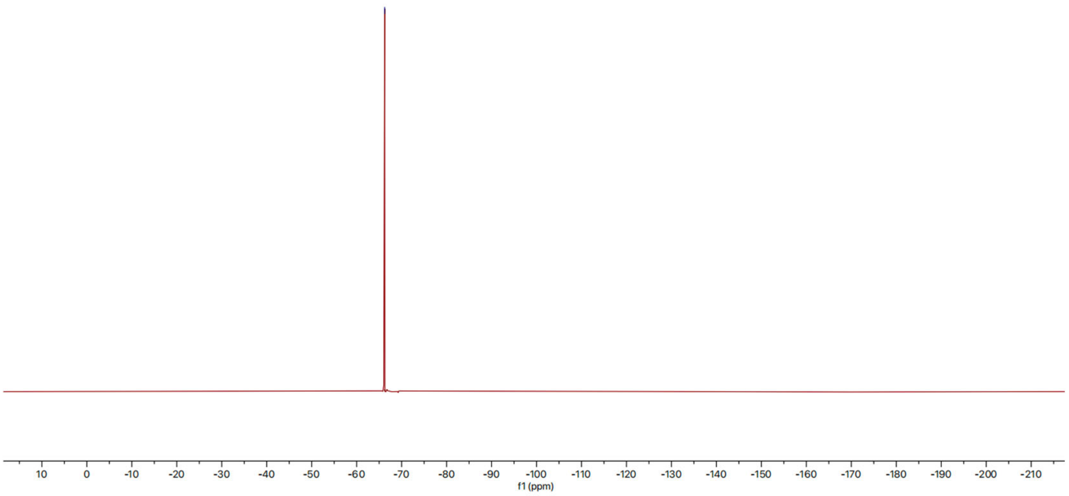


$^{19}F$ NMR spectrum (376 MHz, Chloroform-*d*) of compound **36**

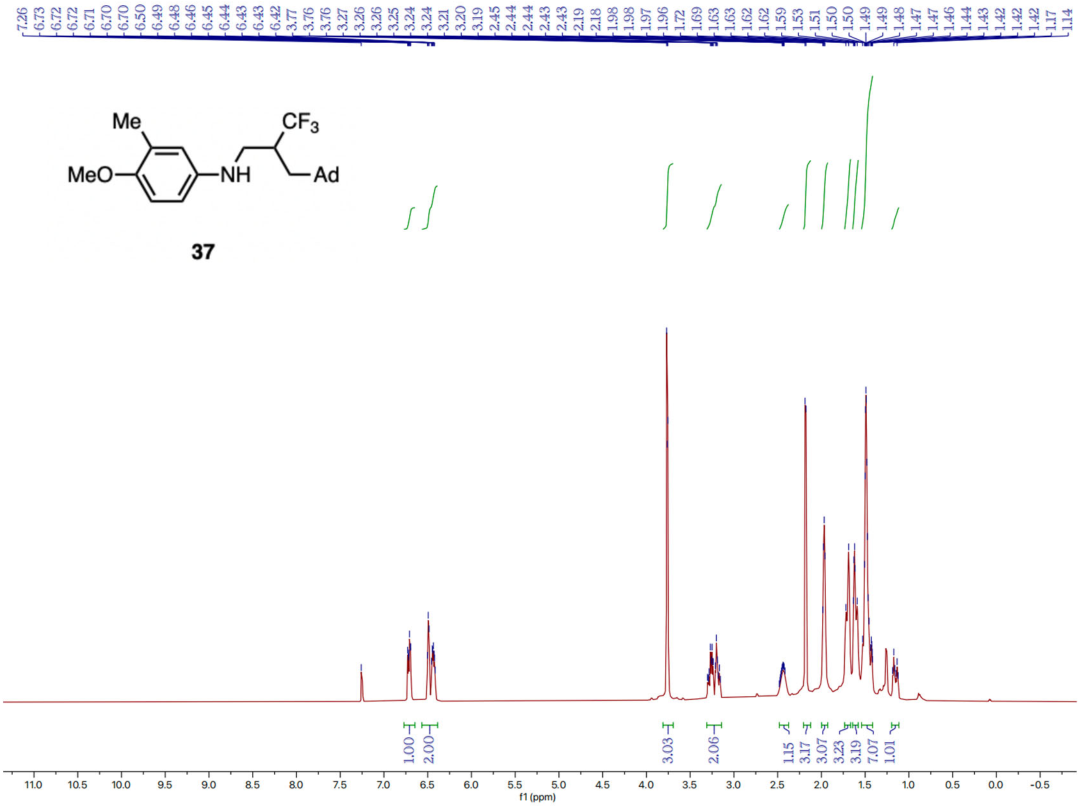


$^{1}$H NMR spectrum (400 MHz, Chloroform-*d*) of compound **37**

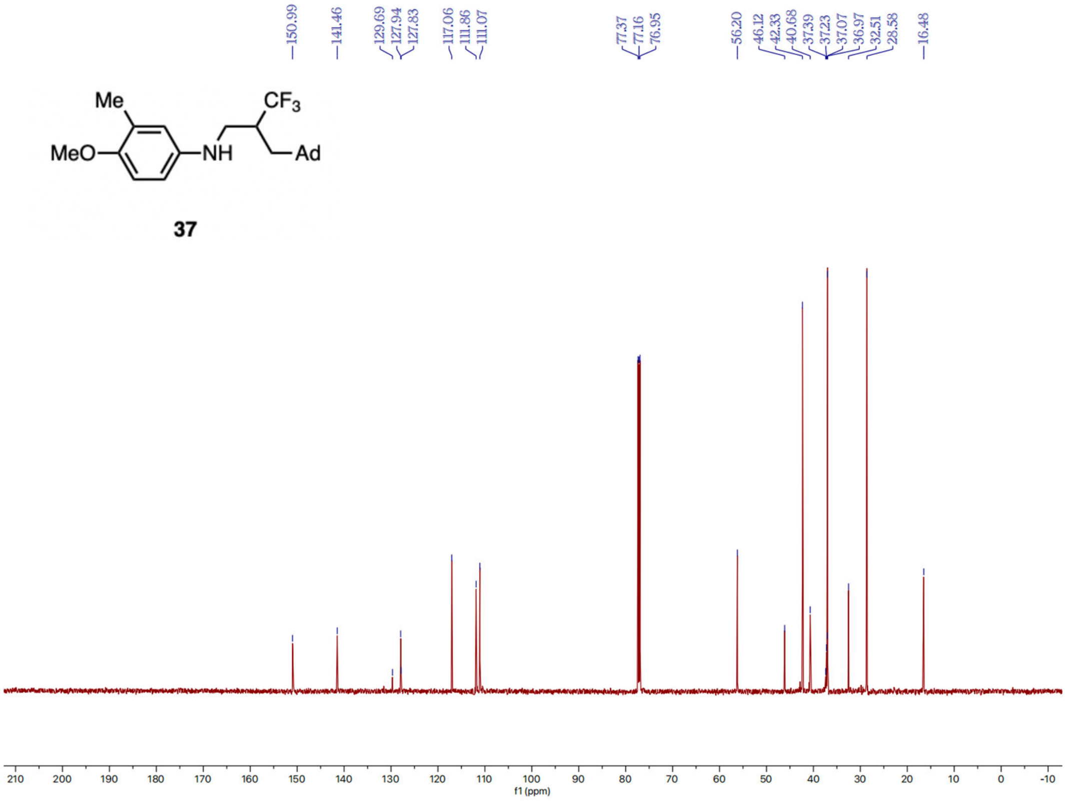


$^{13}$C NMR spectrum (151 MHz, Chloroform-*d*) of compound **37**

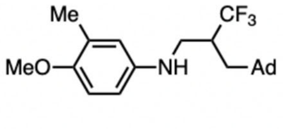


**37**

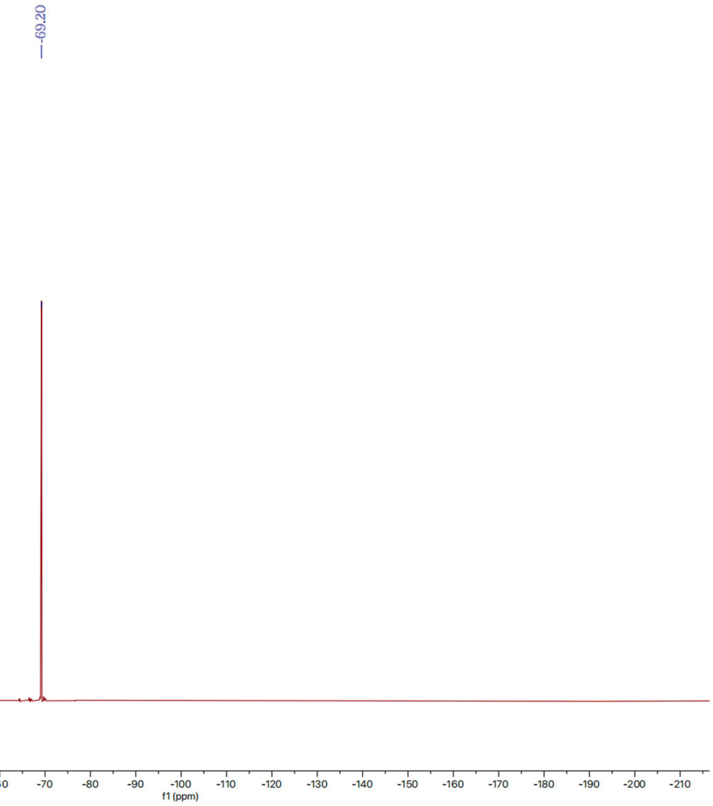


$^{19}$F NMR spectrum (376 MHz, Chloroform-*d*) of compound **37**

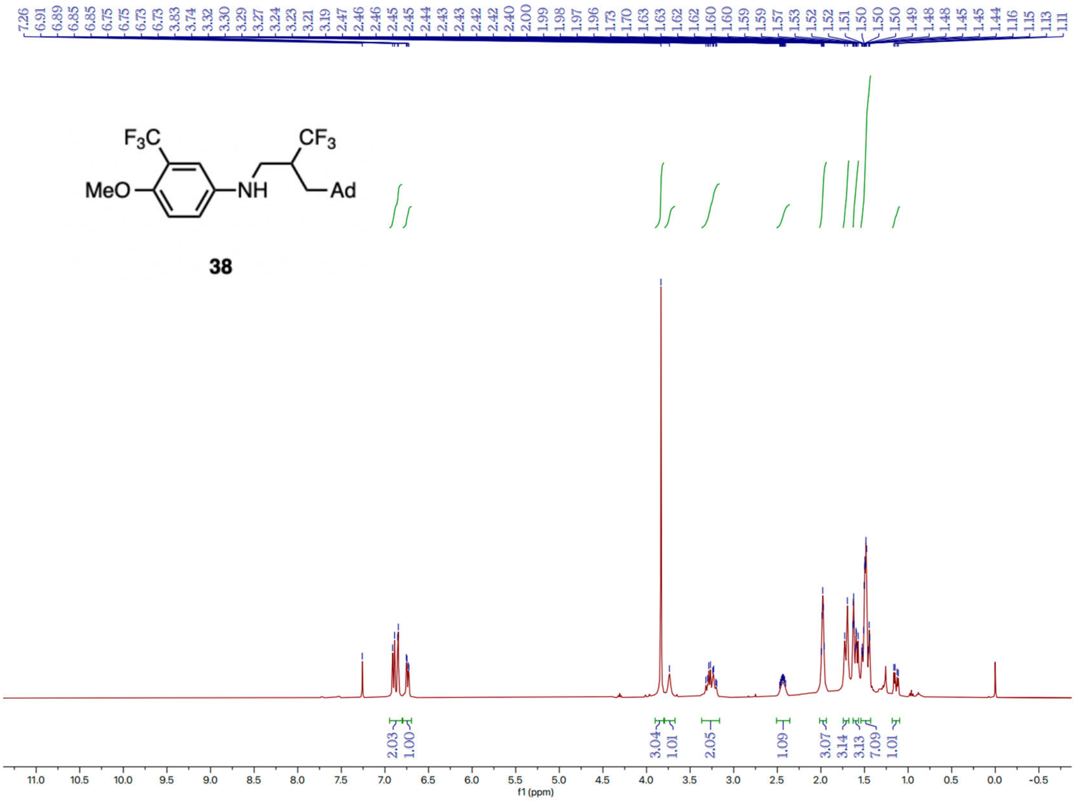

$^{1}$H NMR spectrum (400 MHz, Chloroform-*d*) of compound **38**

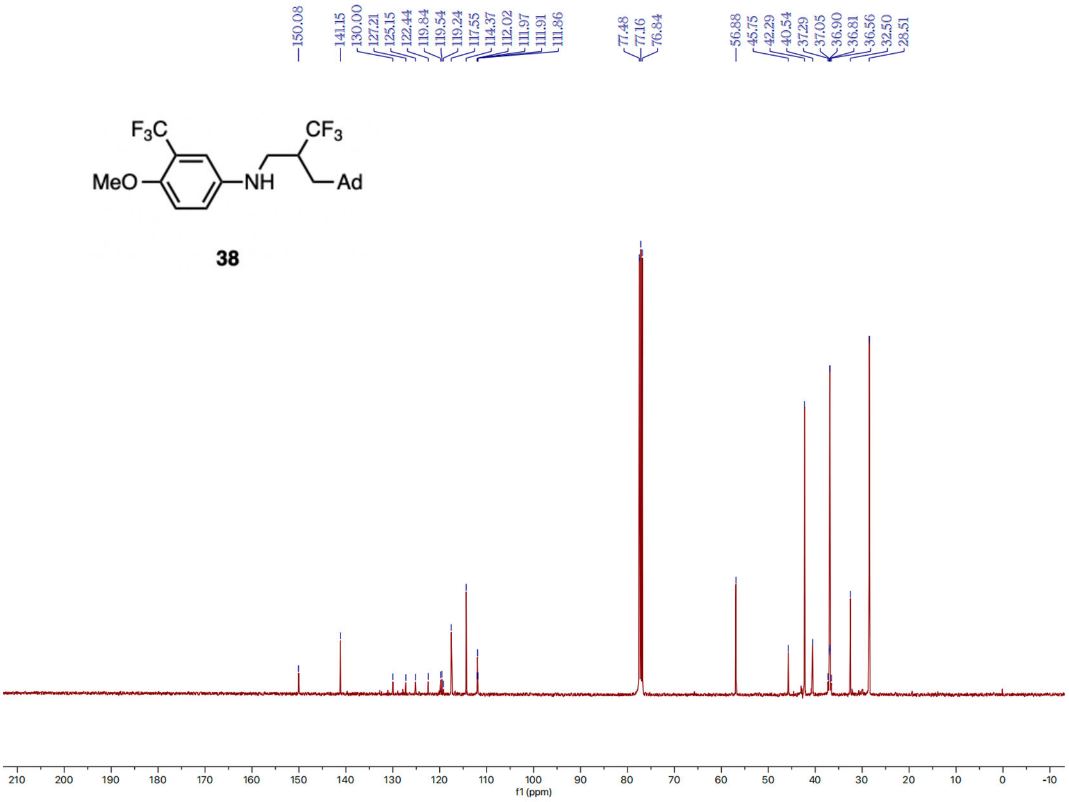

$^{13}$C NMR spectrum (101 MHz, Chloroform-*d*) of compound **38**

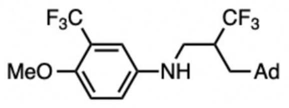

38

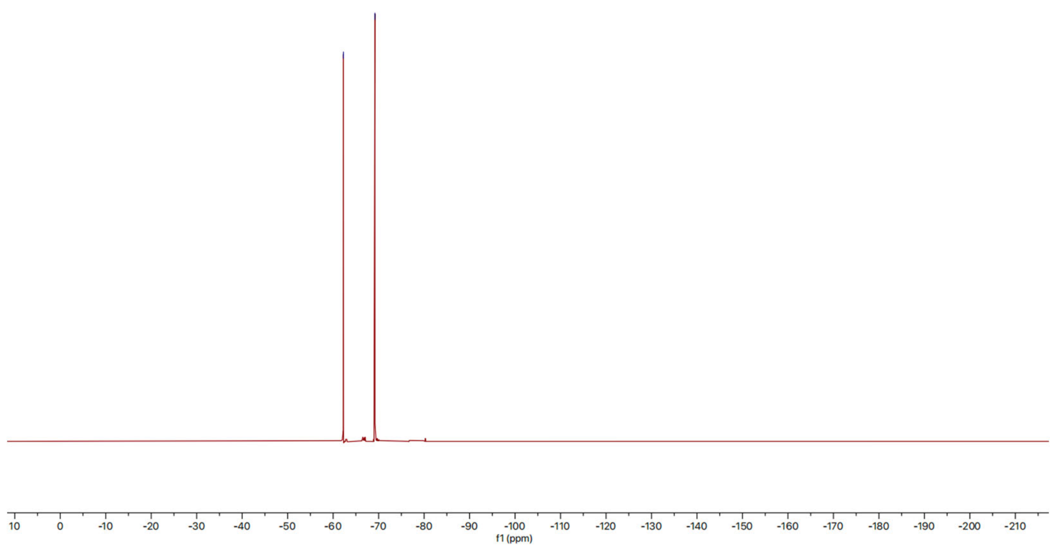


$^{19}F$ NMR spectrum (376 MHz, Chloroform-*d*) of compound **38**

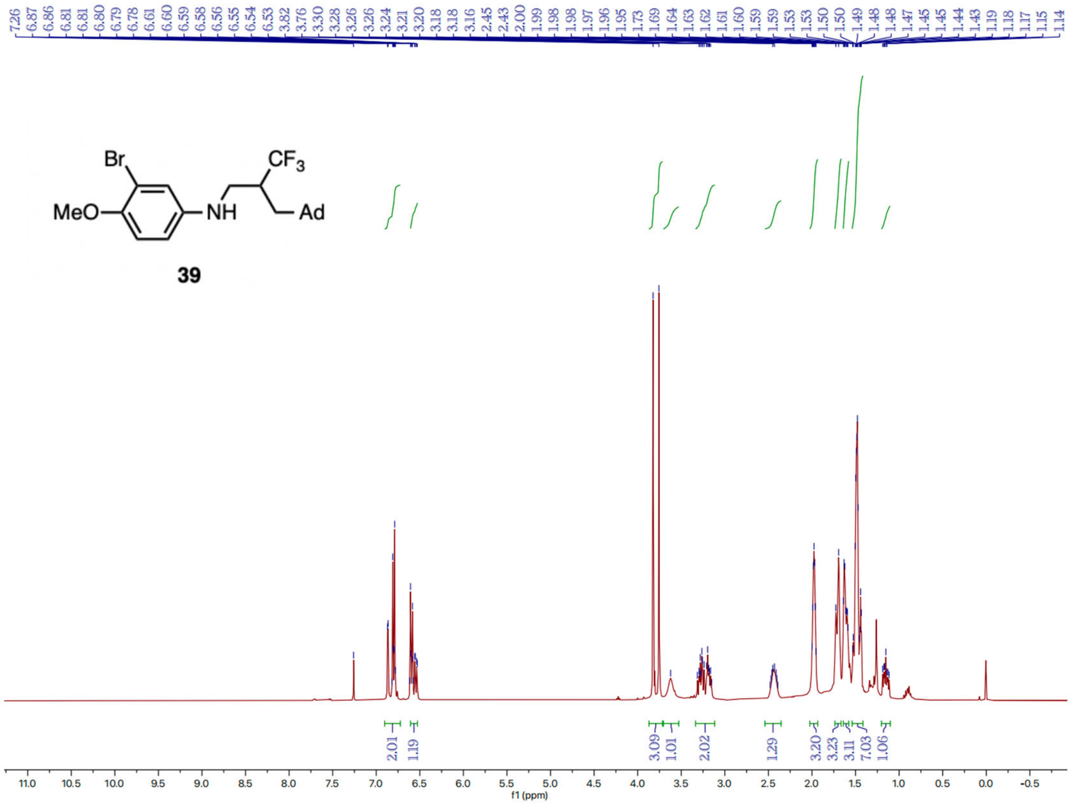


$^{1}$H NMR spectrum (400 MHz, Chloroform-*d*) of compound **39**

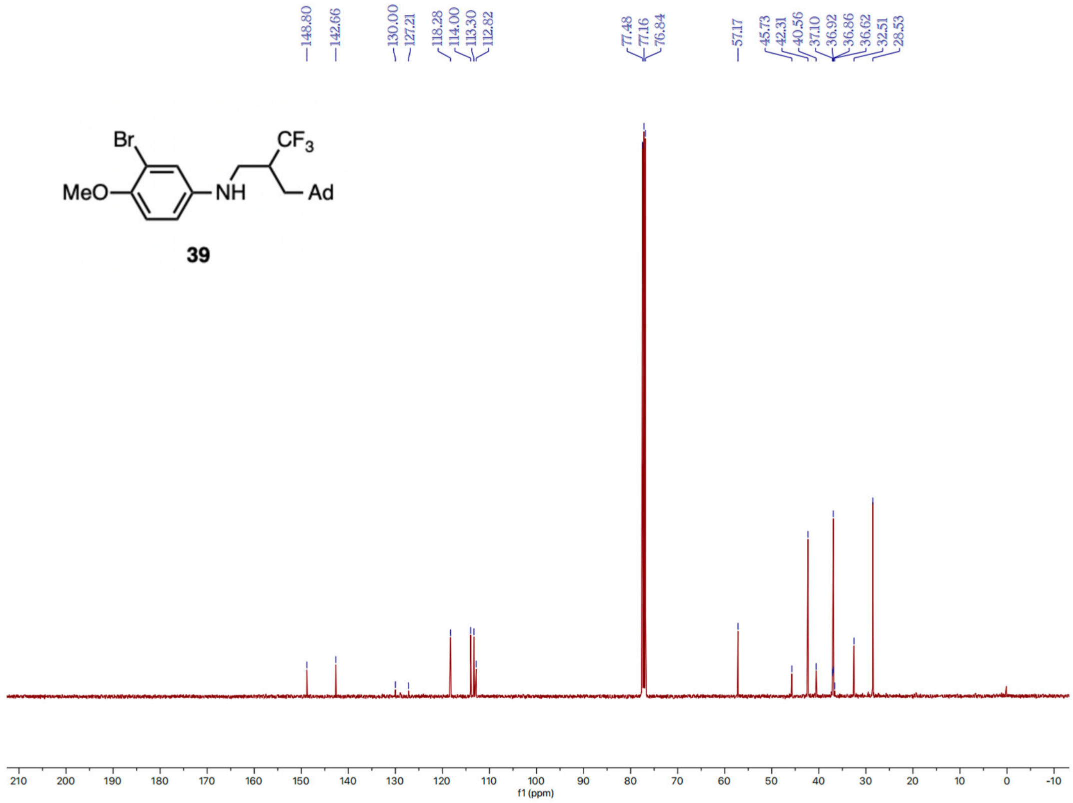


$^{13}$C NMR spectrum (101 MHz, Chloroform-*d*) of compound **39**

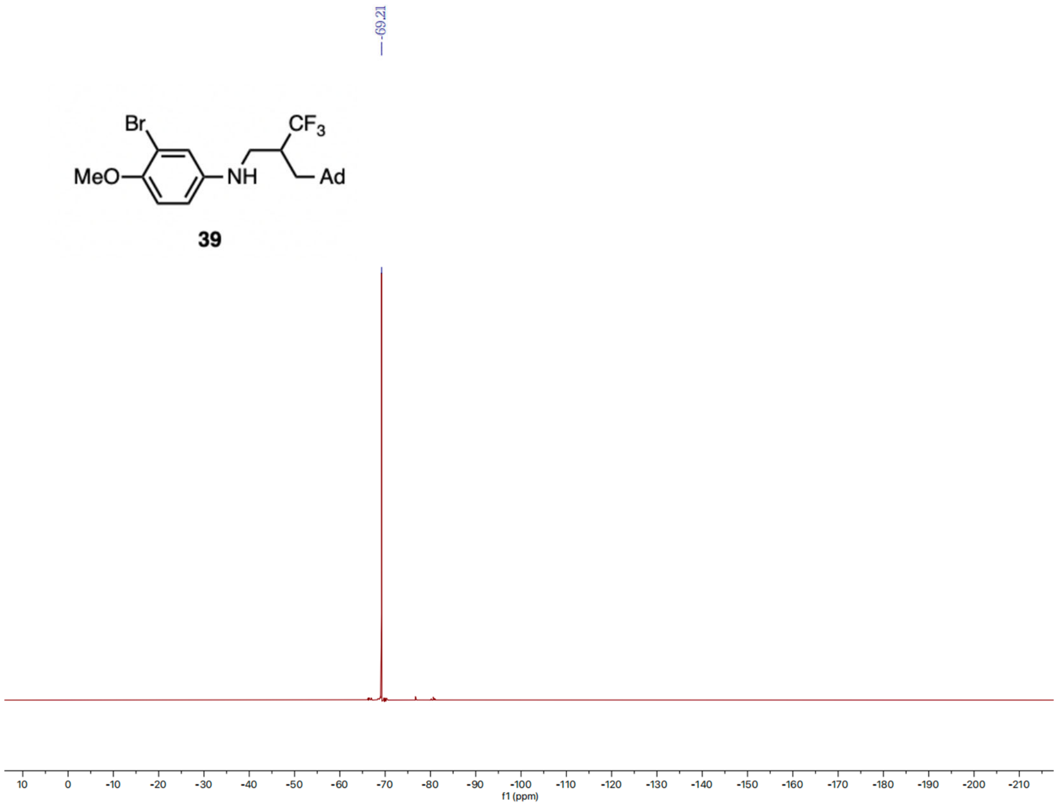


$^{19}$F NMR spectrum (376 MHz, Chloroform-*d*) of compound **39**

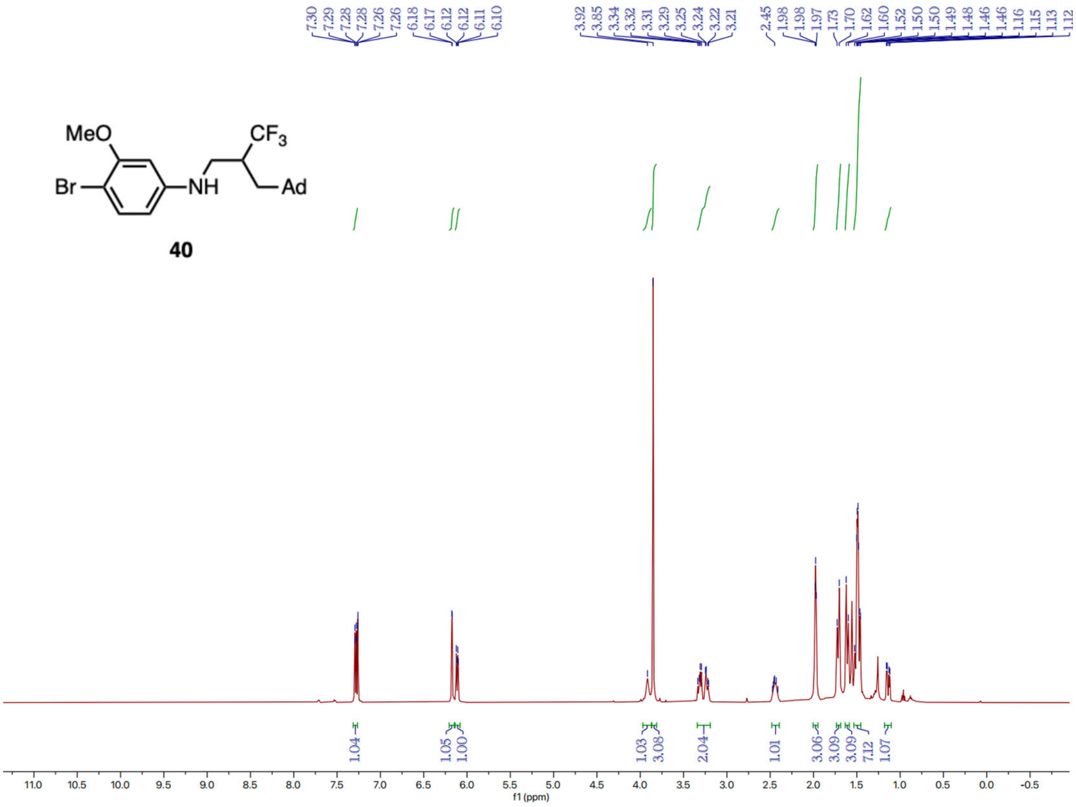

$^{1}$H NMR spectrum (400 MHz, Chloroform-*d*) of compound **40**

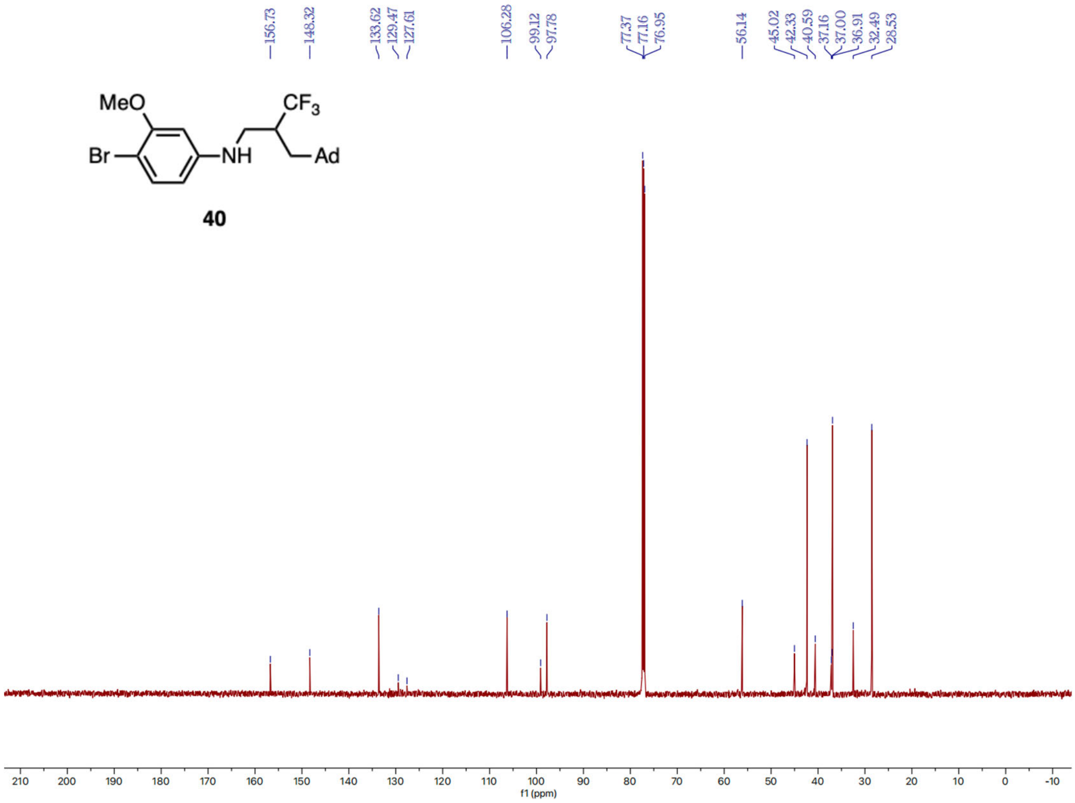

$^{13}$C NMR spectrum (151 MHz, Chloroform-*d*) of compound **40**

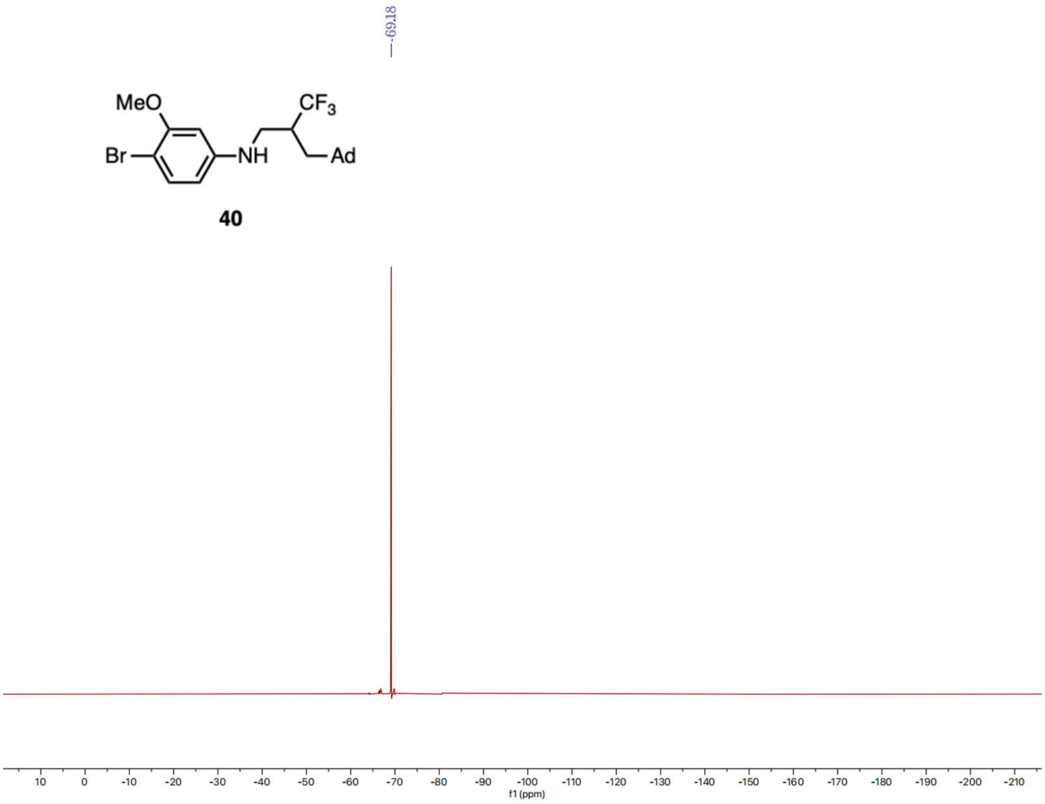


$^{19}$F NMR spectrum (376 MHz, Chloroform-*d*) of compound **40**

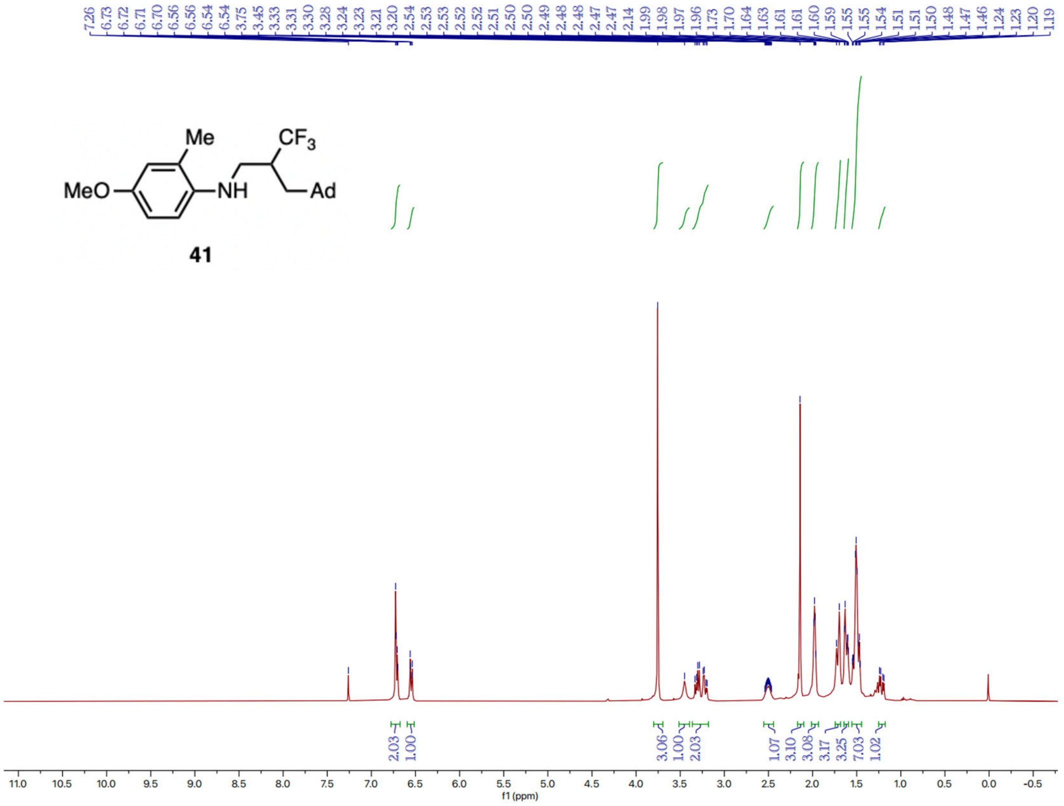

$^{1}$H NMR spectrum (400 MHz, Chloroform-*d*) of compound **41**

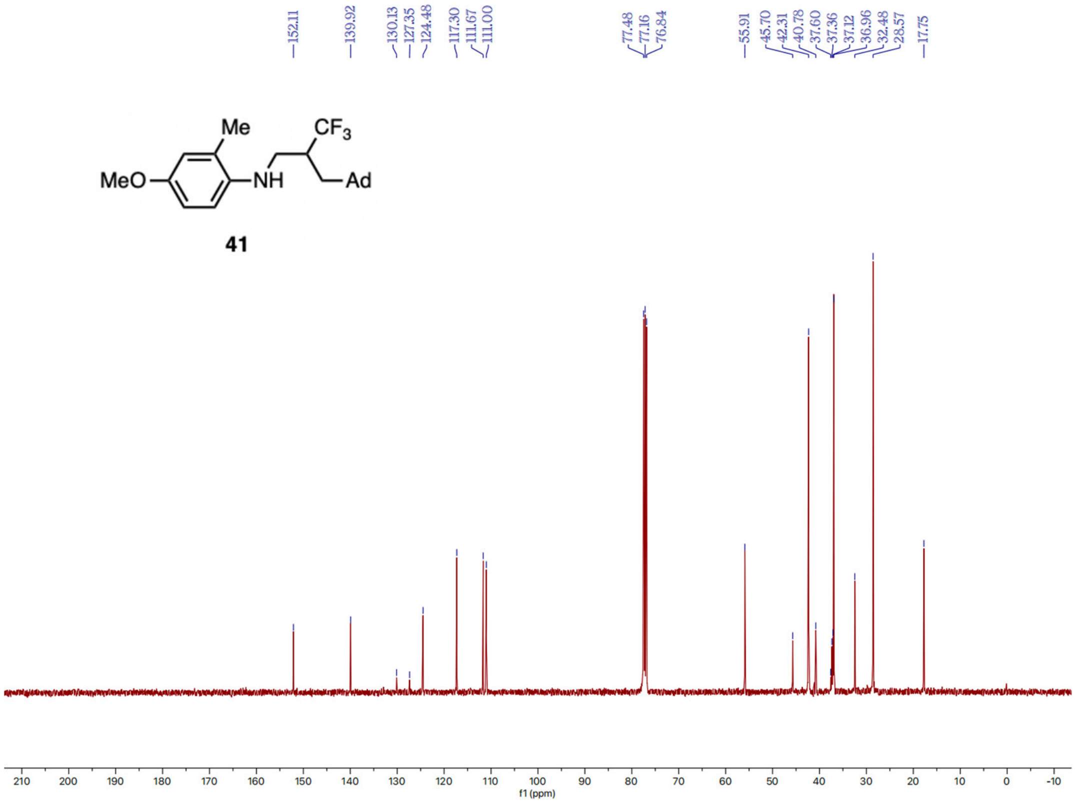

$^{13}$C NMR spectrum (101 MHz, Chloroform-*d*) of compound **41**

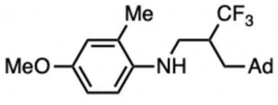


**41**

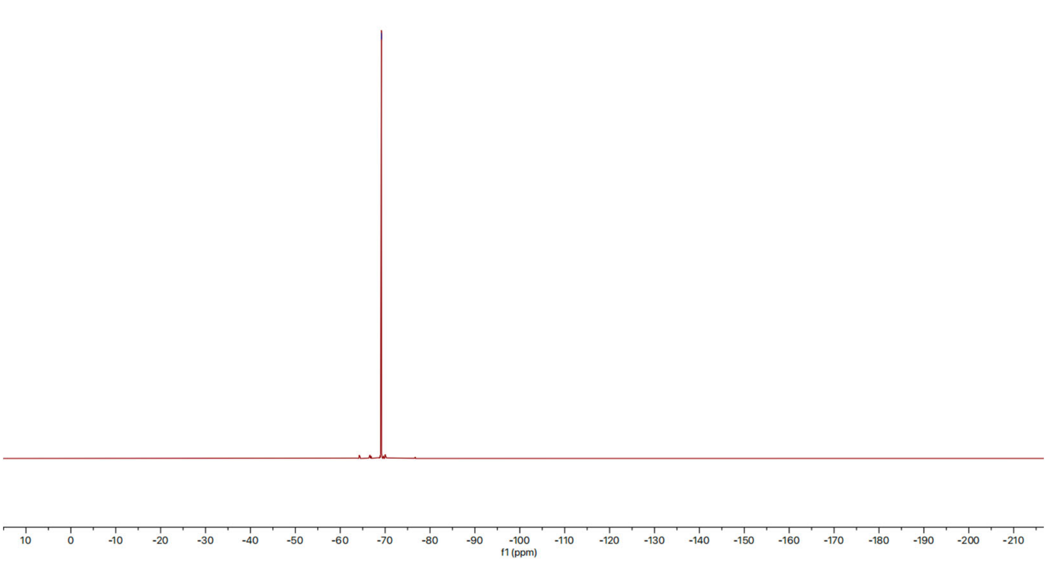


$^{19}$F NMR spectrum (376 MHz, Chloroform-*d*) of compound **41**

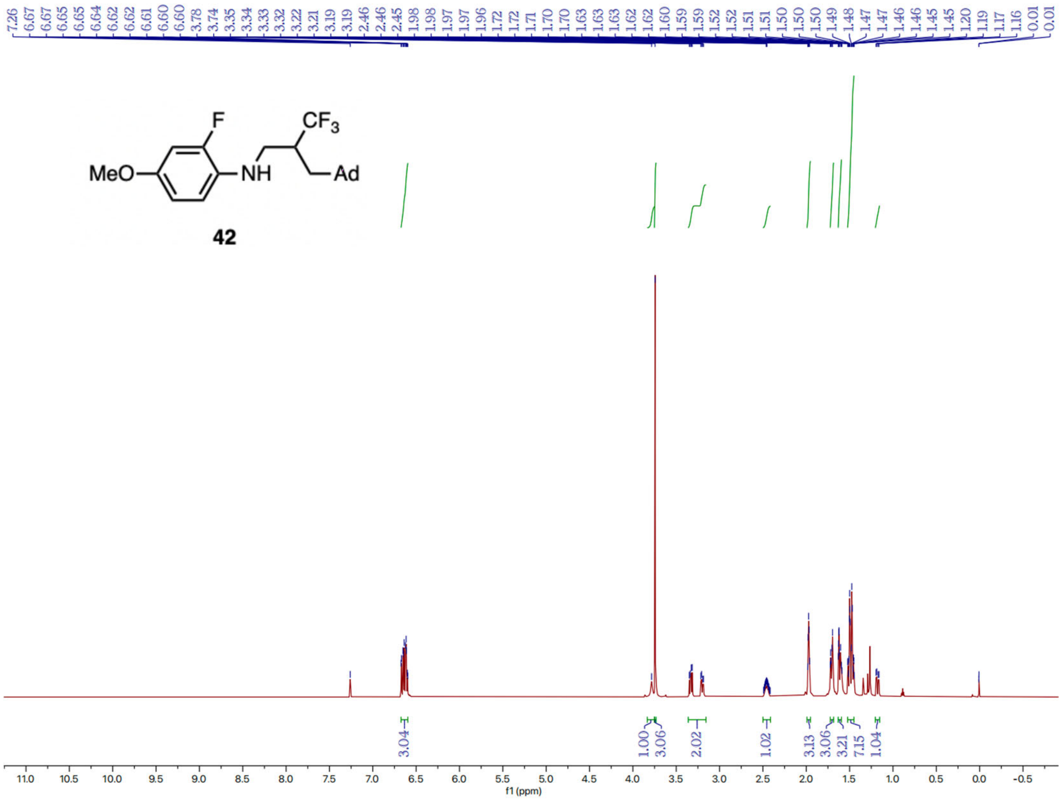

$^{1}$H NMR spectrum (600 MHz, Chloroform-*d*) of compound **42**

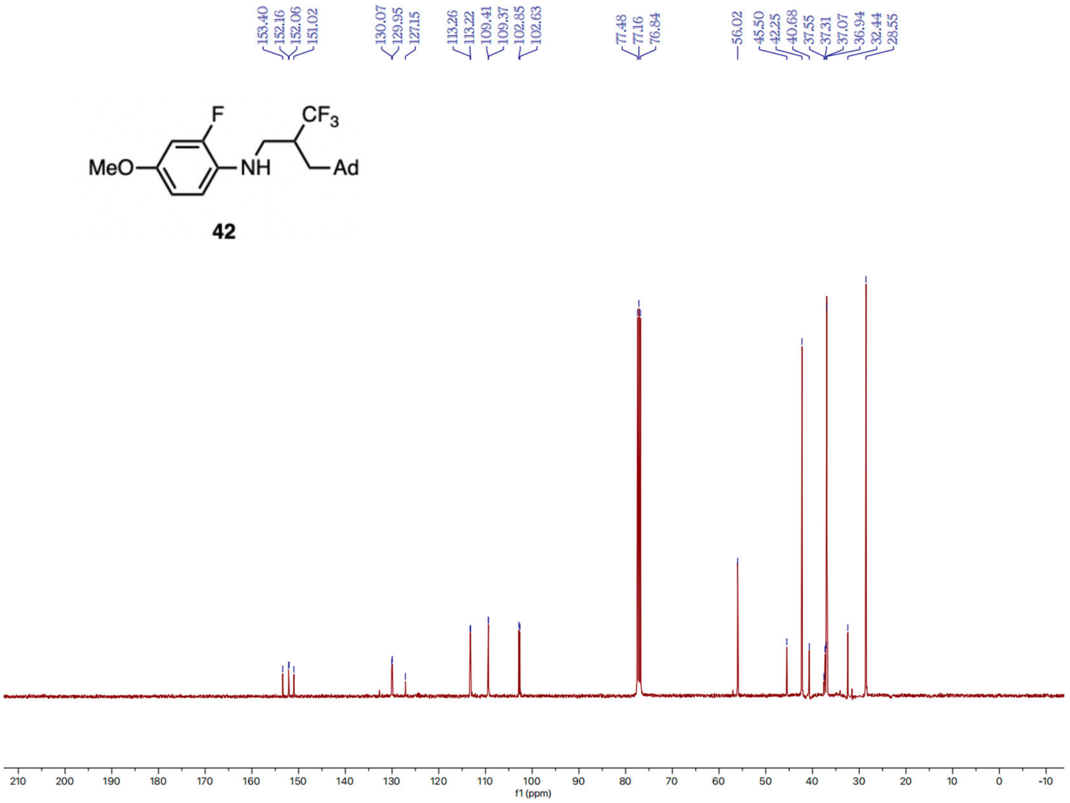

$^{13}$C NMR spectrum (101 MHz, Chloroform-*d*) of compound **42**

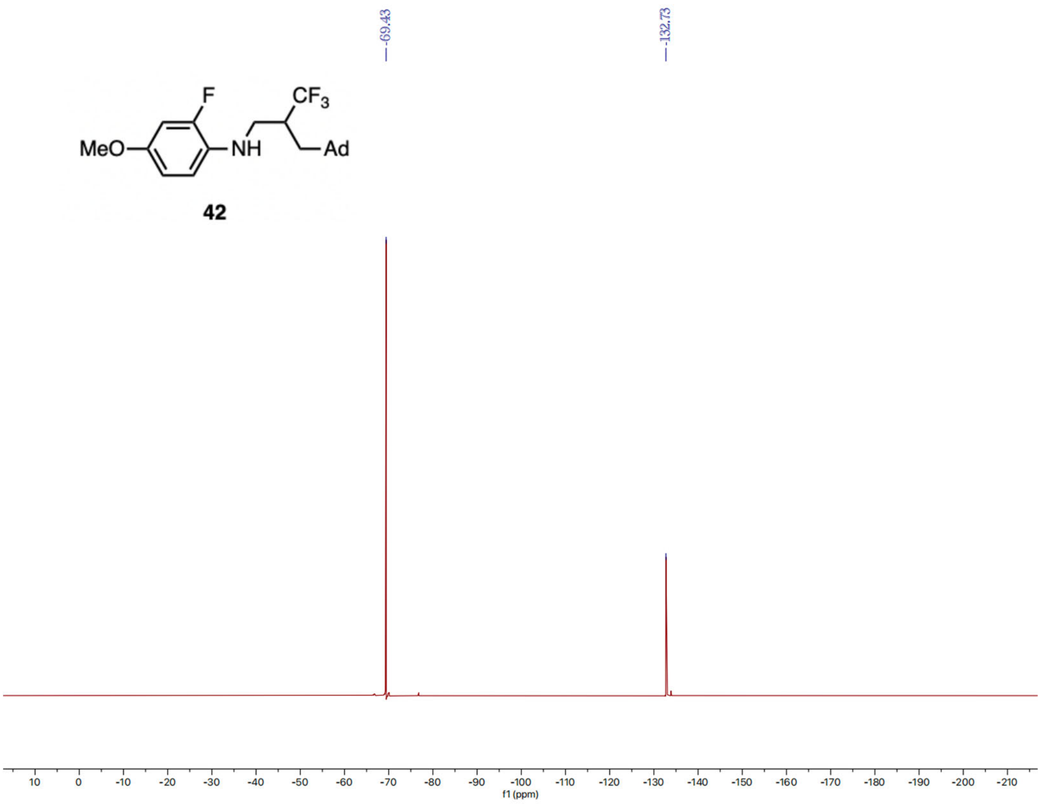


$^{19}$F NMR spectrum (470 MHz, Chloroform-*d*) of compound **42**

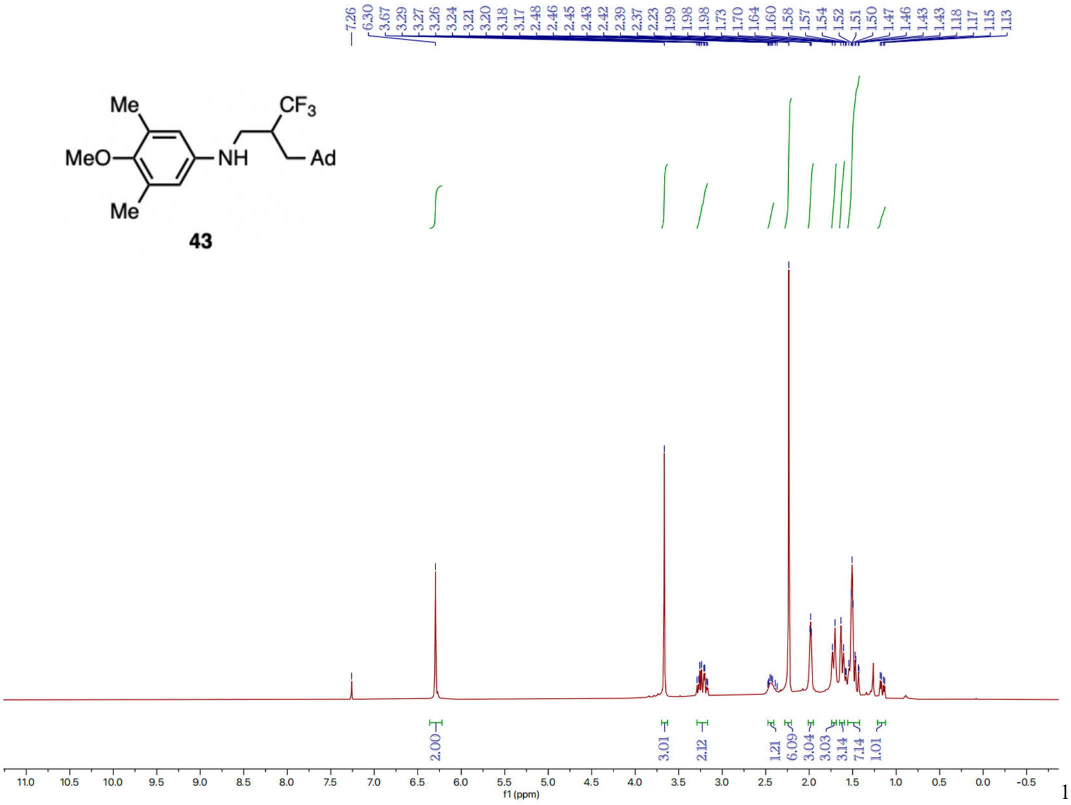


NMR spectrum (400 MHz, Chloroform-*d*) of compound **43**

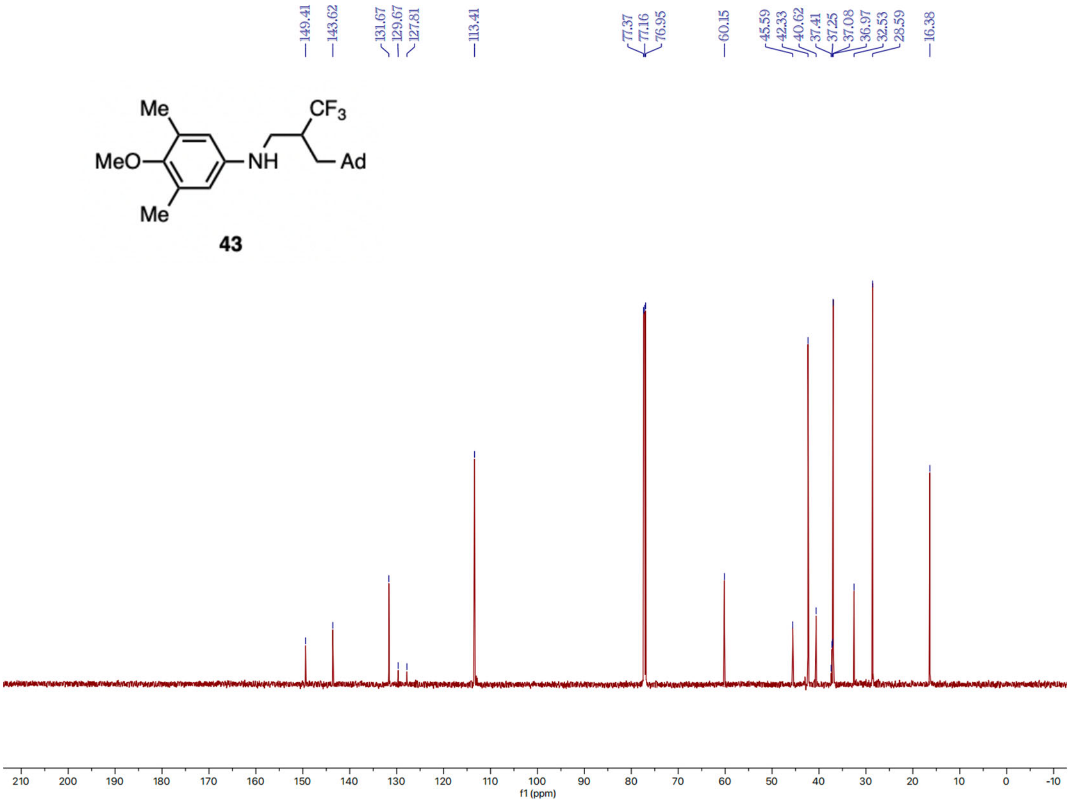


$^{13}C$ NMR spectrum (151 MHz, Chloroform-*d*) of compound **43**

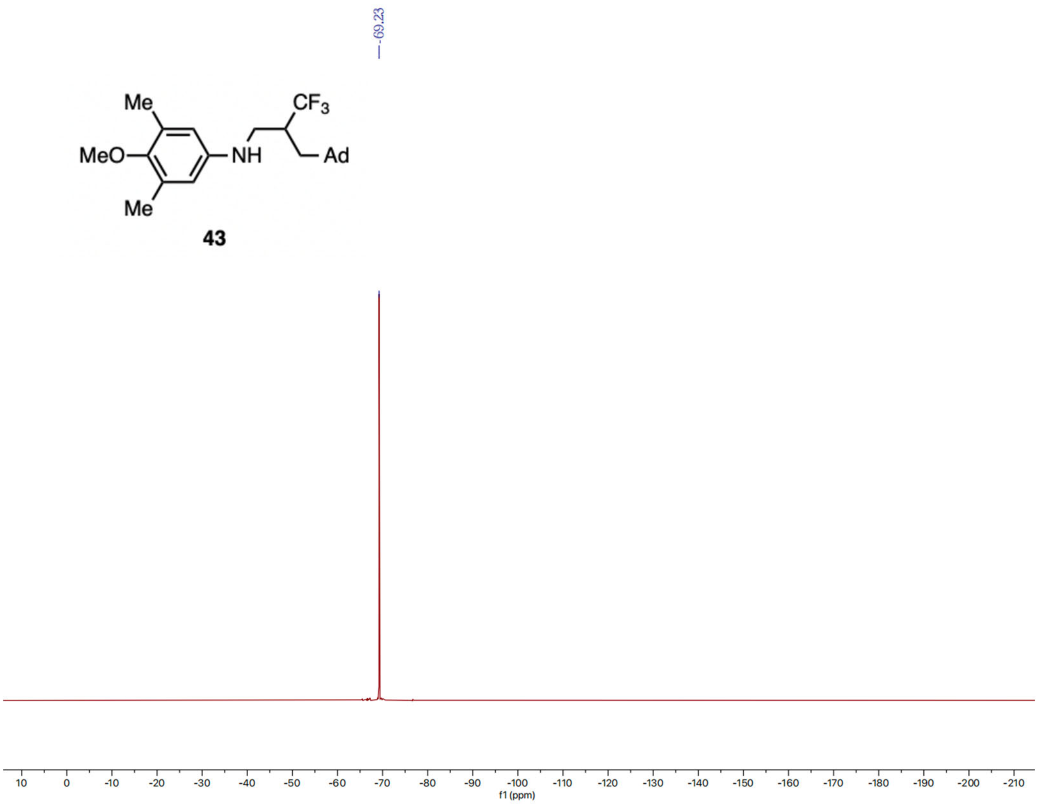


$^{19}$F NMR spectrum (376 MHz, Chloroform-*d*) of compound **43**

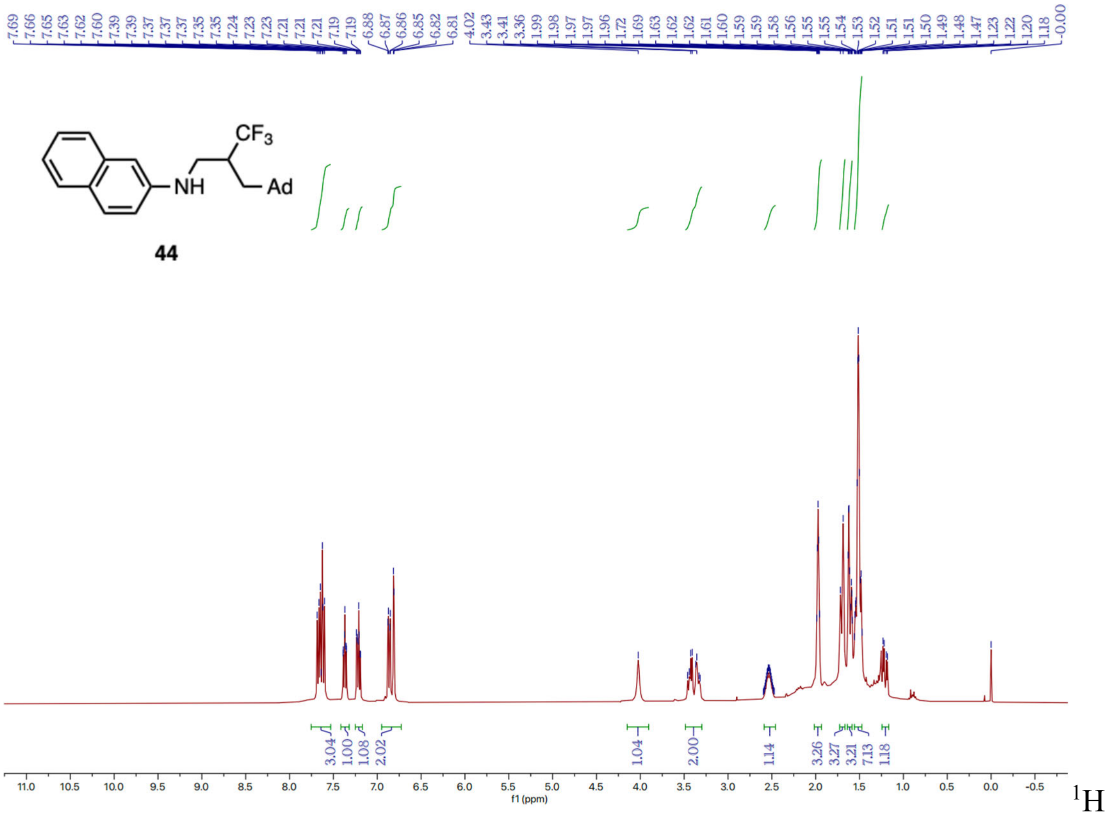

NMR spectrum (400 MHz, Chloroform-*d*) of compound **44**

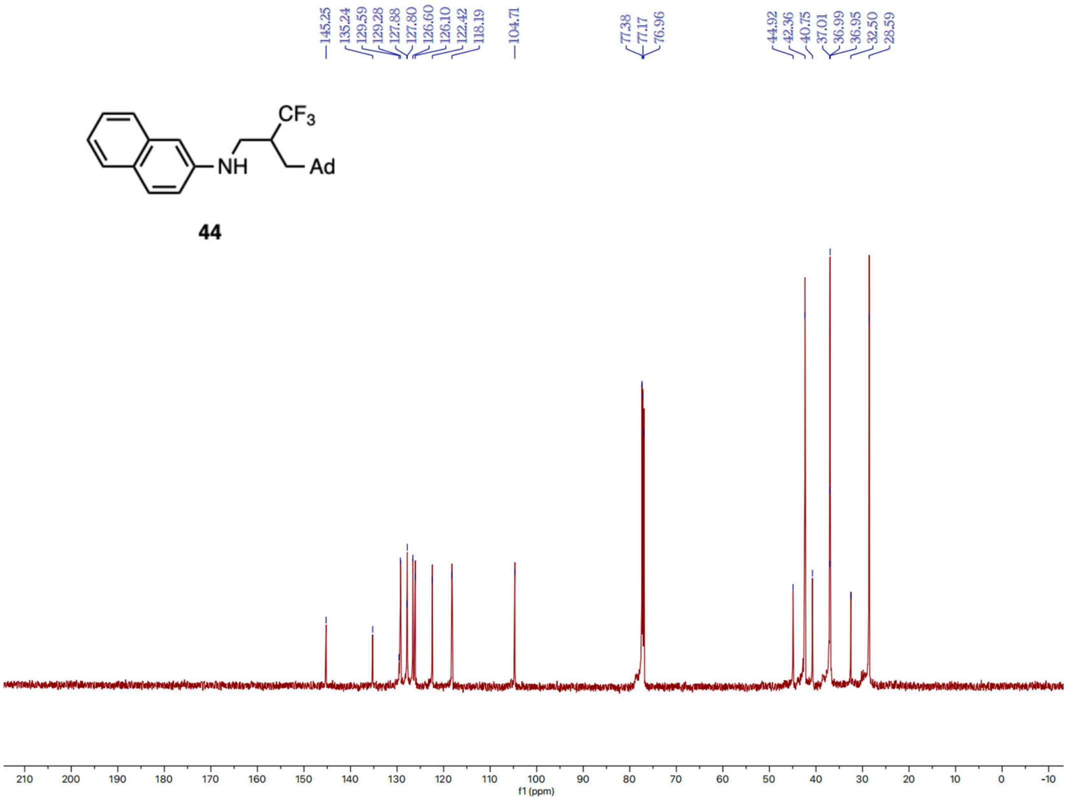

$^{13}$C NMR spectrum (151 MHz, Chloroform-*d*) of compound **44**

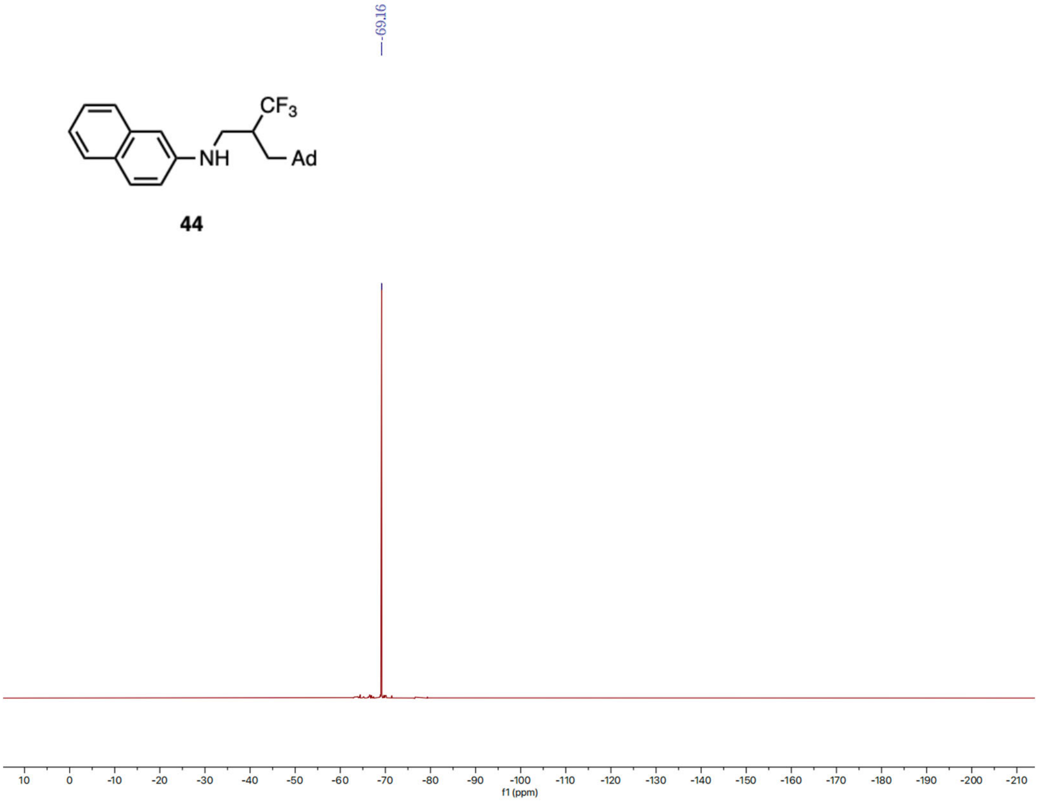


$^{19}F$ NMR spectrum (376 MHz, Chloroform-*d*) of compound **44**

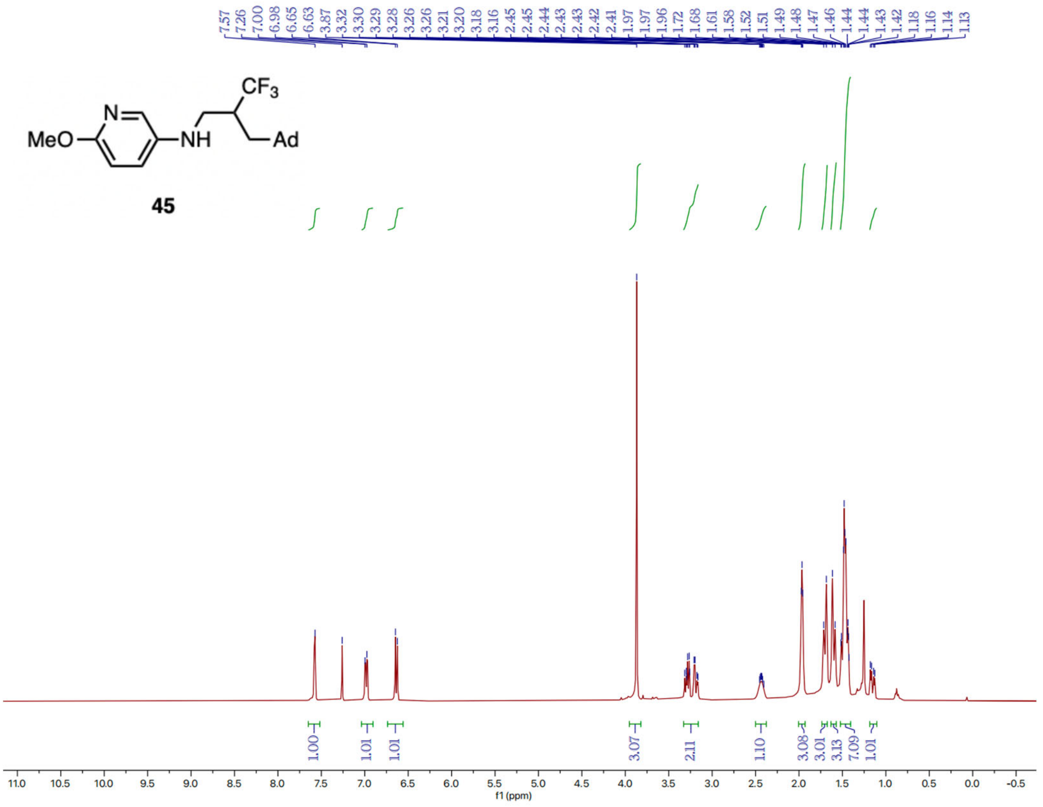


$^{1}$H NMR spectrum (400 MHz, Chloroform-*d*) of compound **45**

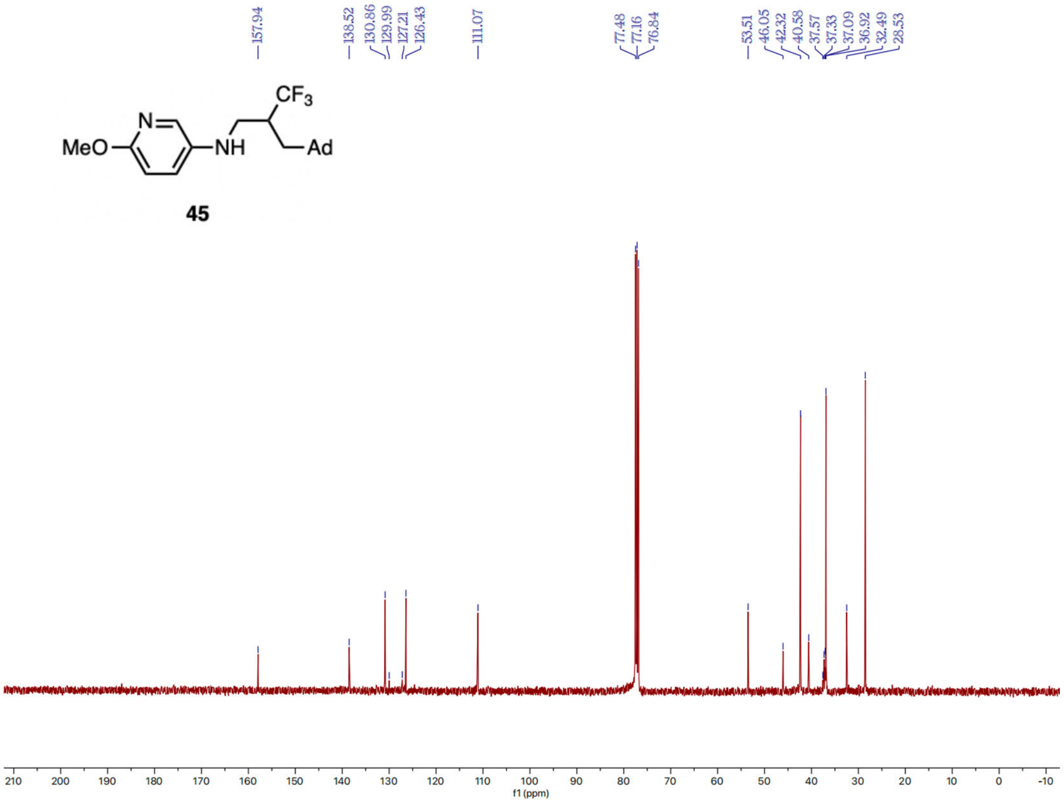


$^{13}$C NMR spectrum (101 MHz, Chloroform-*d*) of compound **45**

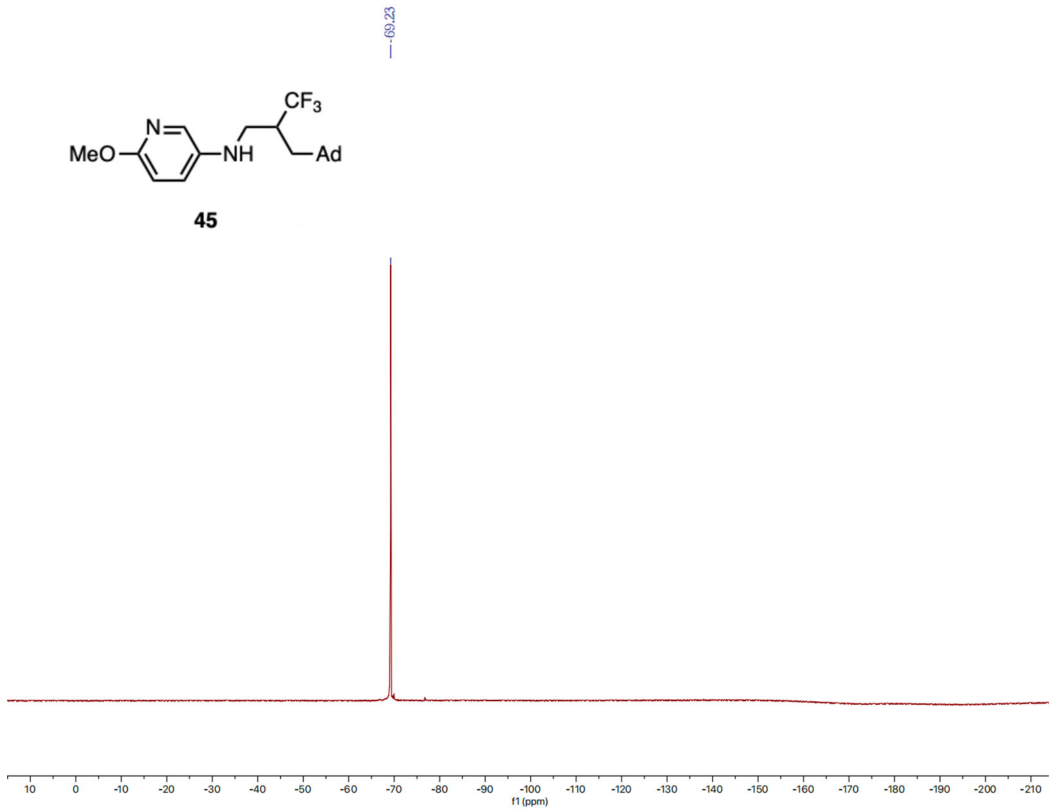


$^{19}$F NMR spectrum (376 MHz, Chloroform-*d*) of compound **45**

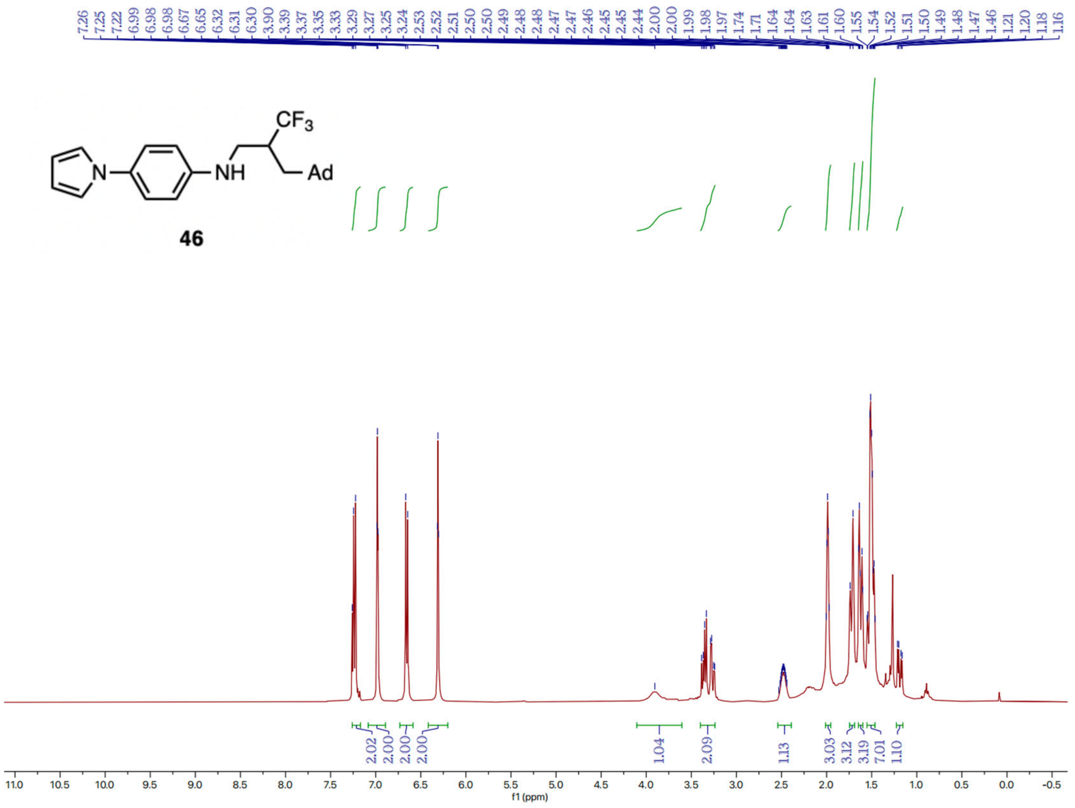

$^{1}$H NMR spectrum (400 MHz, Chloroform-*d*) of compound **46**

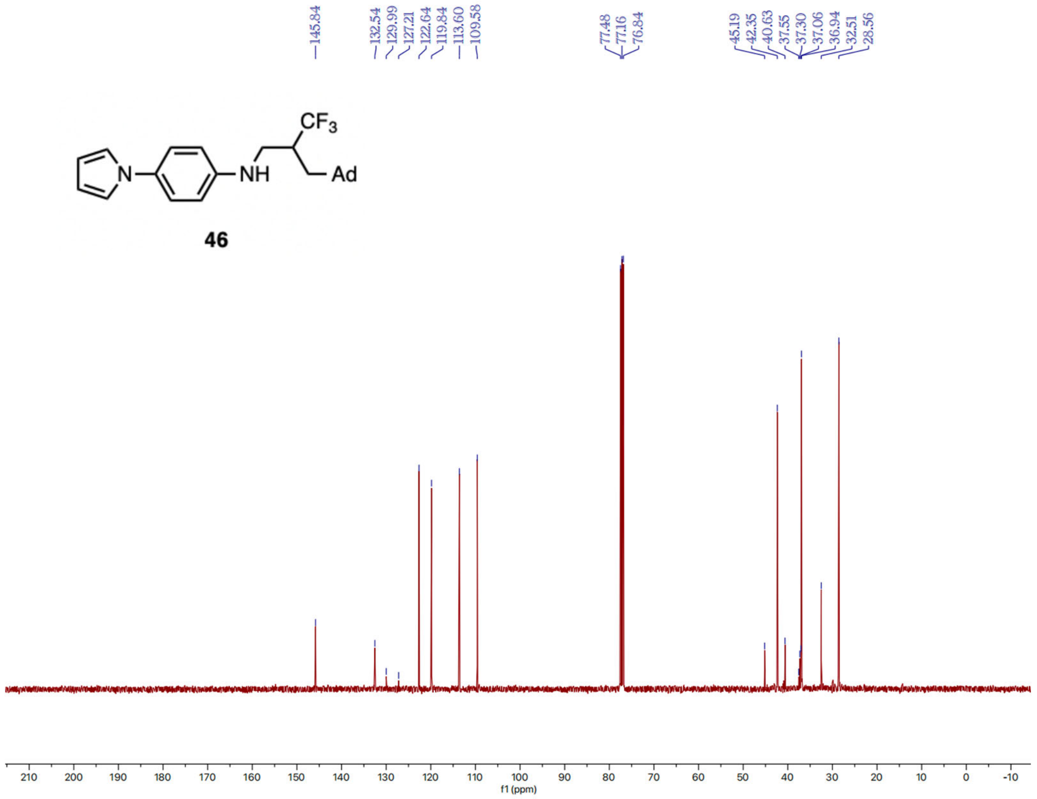

$^{13}$C NMR spectrum (101 MHz, Chloroform-*d*) of compound **46**

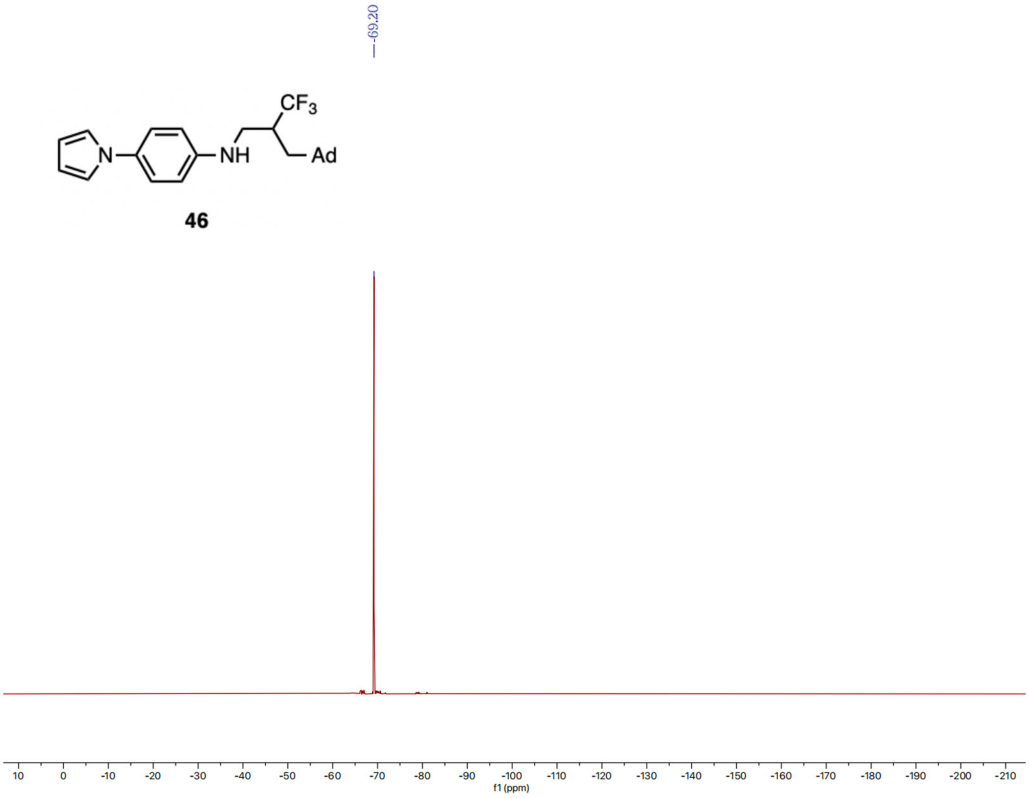


$^{19}$F NMR spectrum (376 MHz, Chloroform-*d*) of compound **46**

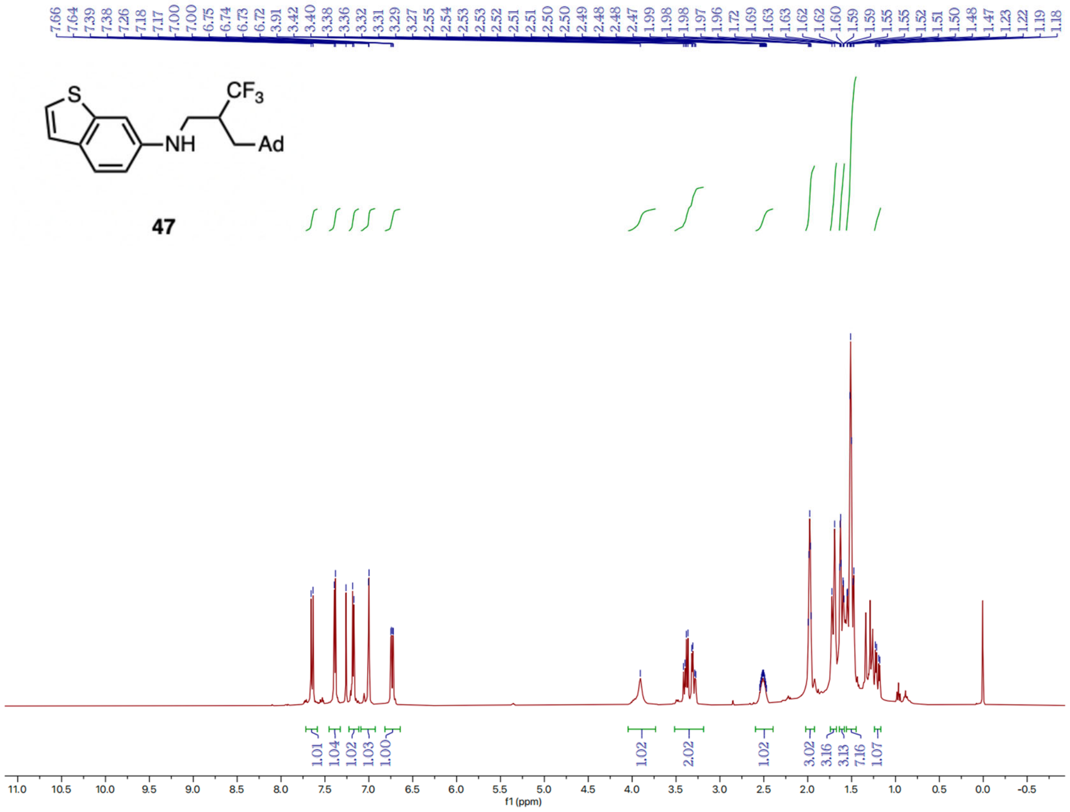

$^{1}$H NMR spectrum (400 MHz, Chloroform-*d*) of compound **47**

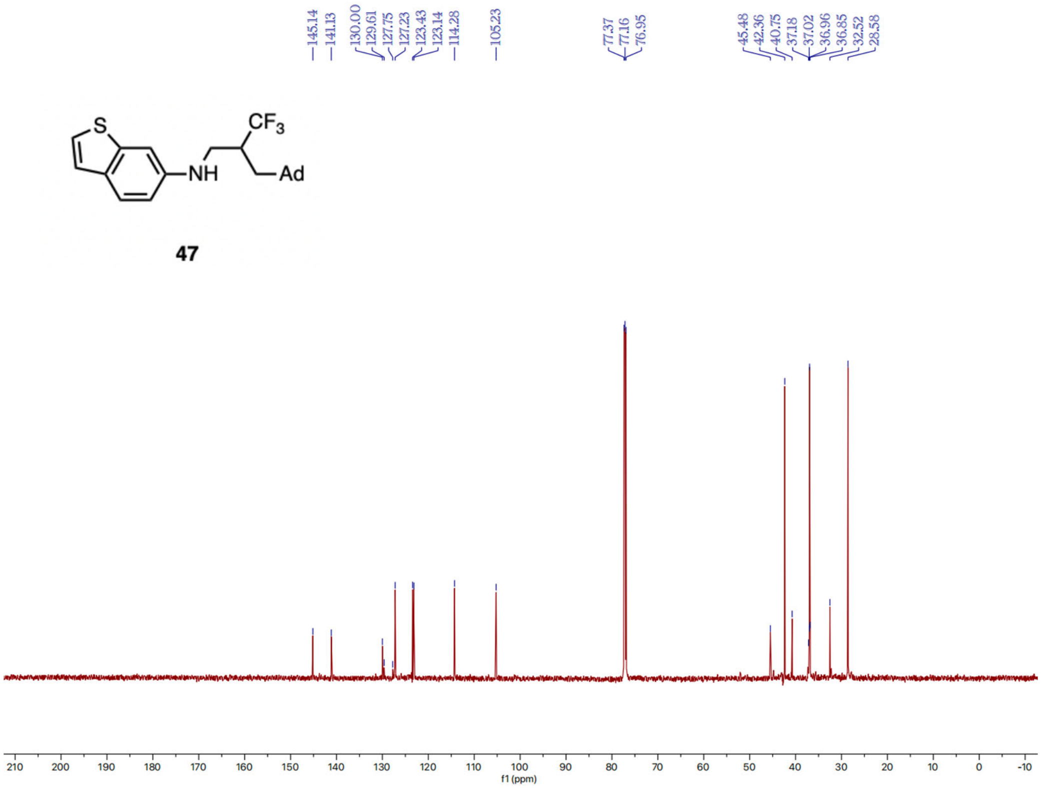

$^{13}$C NMR spectrum (151 MHz, Chloroform-*d*) of compound **47**

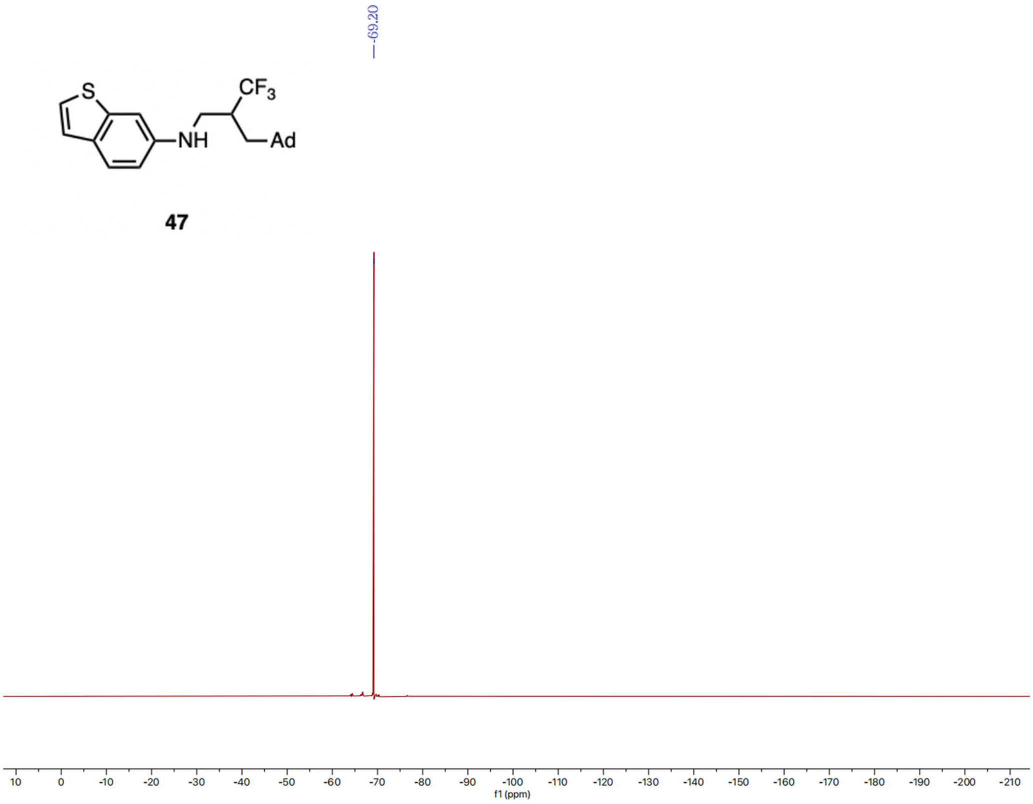


$^{19}F$ NMR spectrum (376 MHz, Chloroform-*d*) of compound **47**

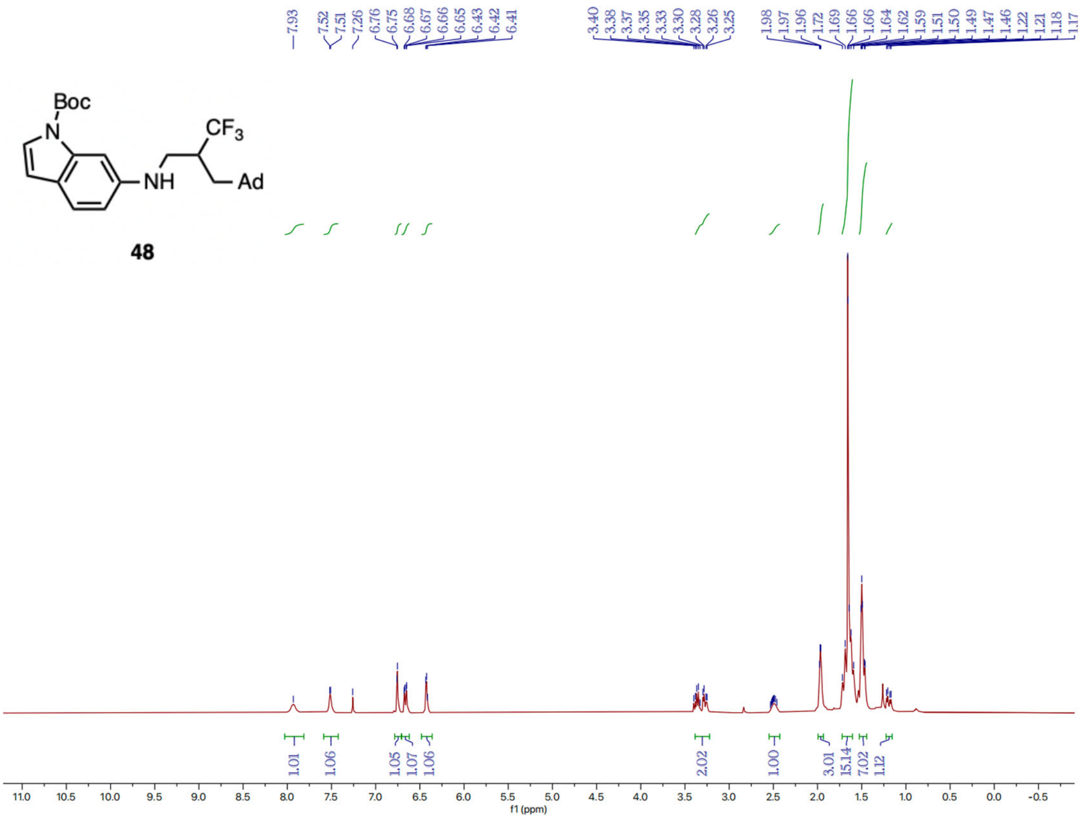

$^{1}H$ NMR spectrum (400 MHz, Chloroform-*d*) of compound **48**

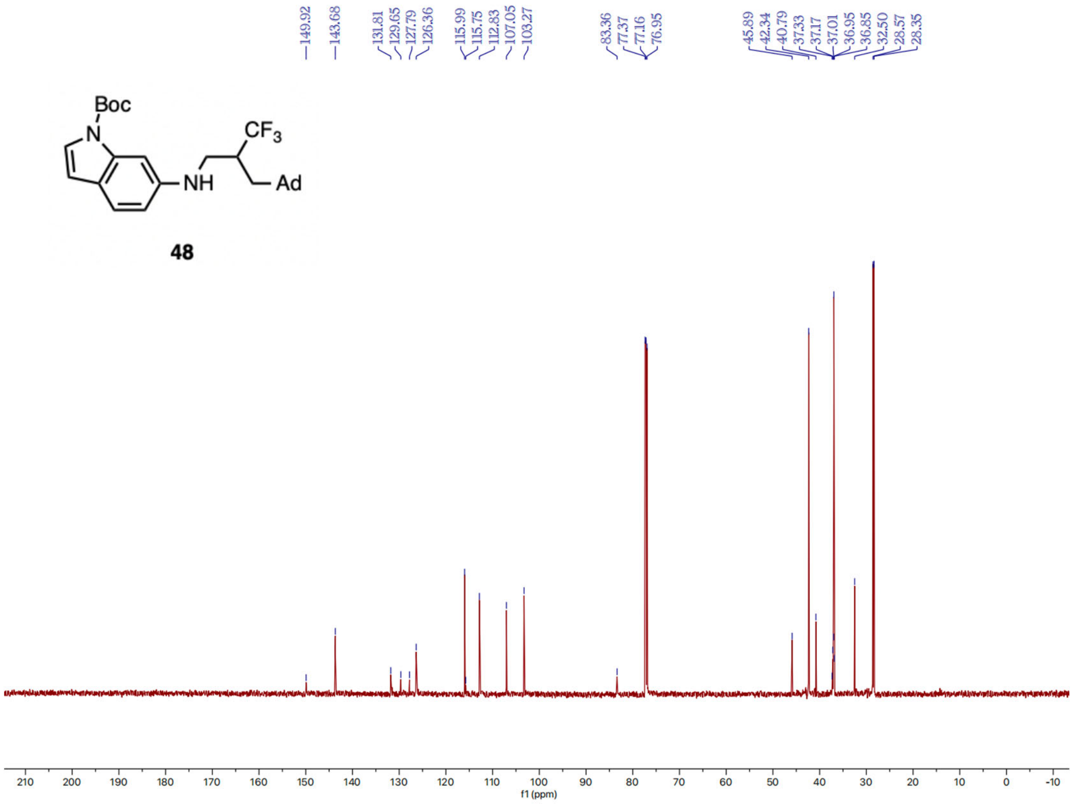

$^{13}C$ NMR spectrum (151 MHz, Chloroform-*d*) of compound **48**

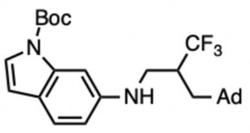


**48**

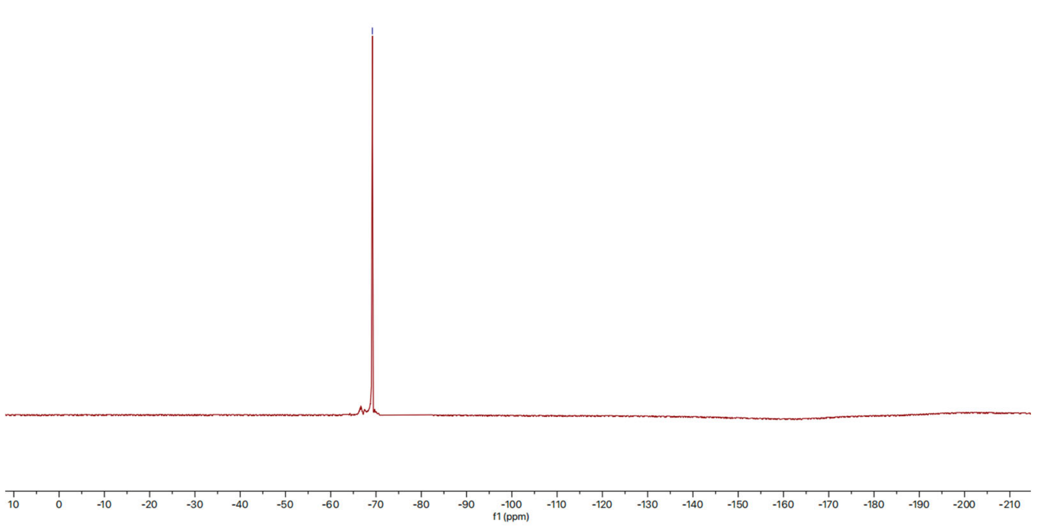


$^{19}F$ NMR spectrum (376 MHz, Chloroform-*d*) of compound **48**

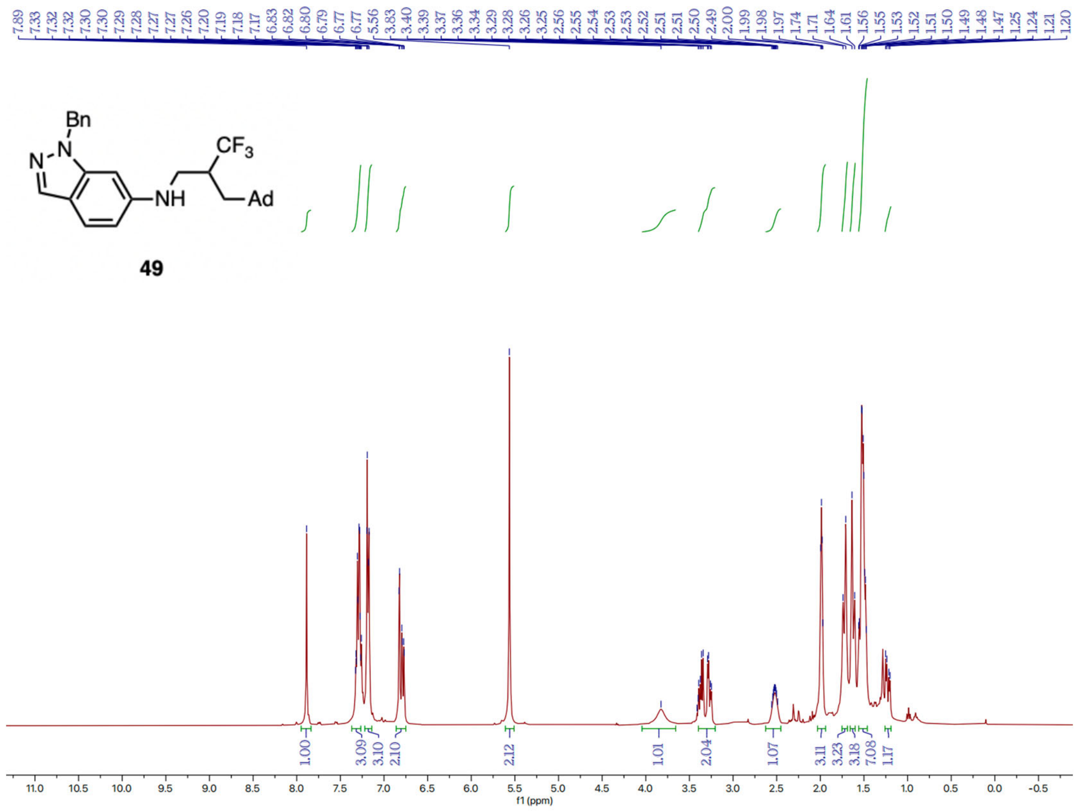

$^{1}$H NMR spectrum (400 MHz, Chloroform-*d*) of compound **49**

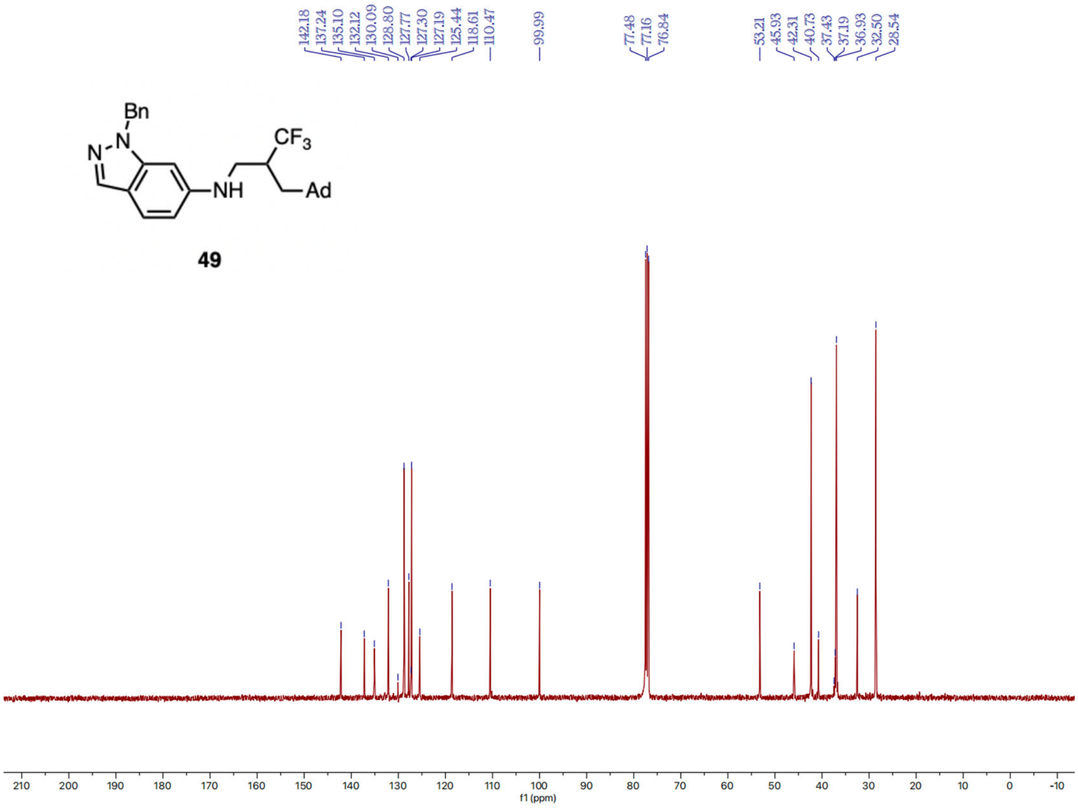

$^{13}$C NMR spectrum (101 MHz, Chloroform-*d*) of compound **49**

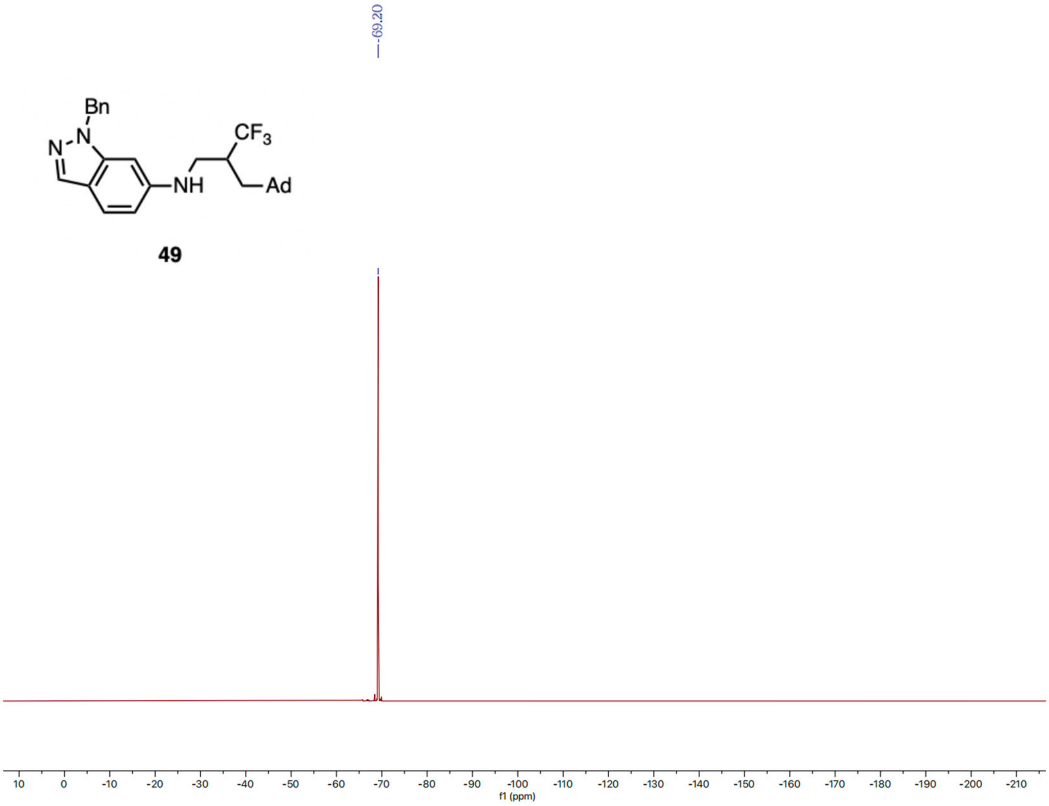


$^{19}$F NMR spectrum (376 MHz, Chloroform-*d*) of compound **49**

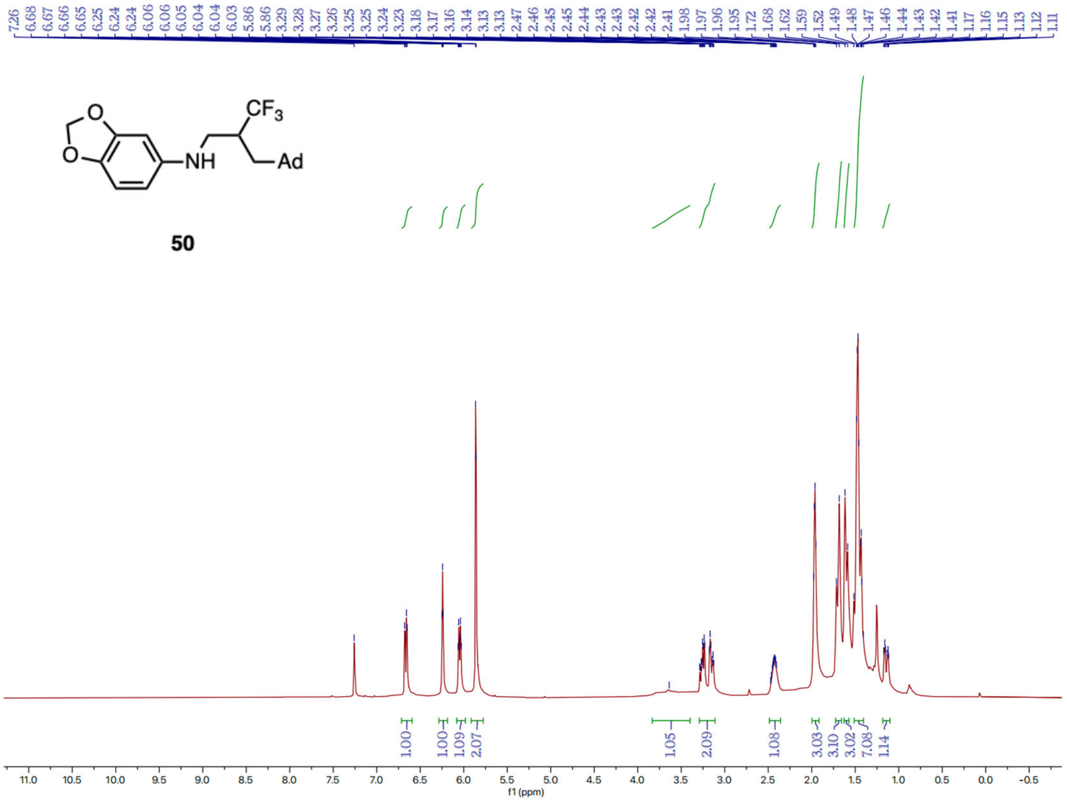


$^{1}$H NMR spectrum (400 MHz, Chloroform-*d*) of compound **50**

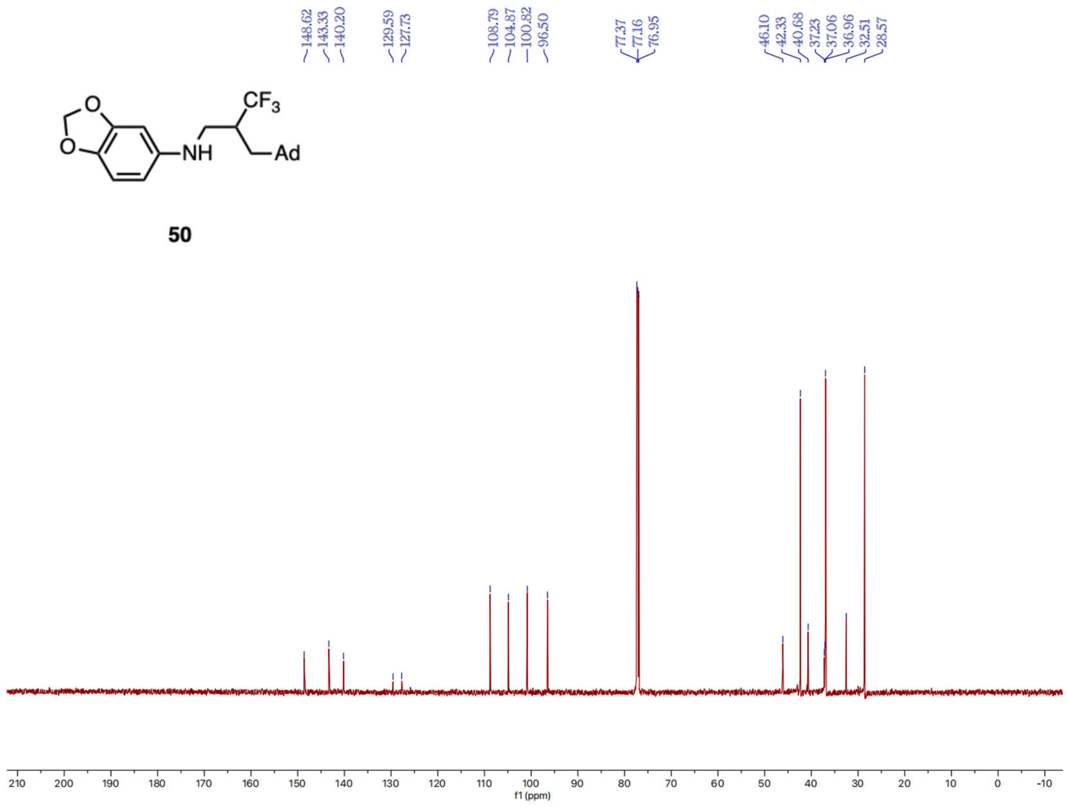


$^{13}$C NMR spectrum (151 MHz, Chloroform-*d*) of compound **50**

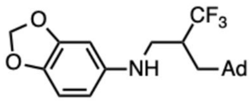

**50**

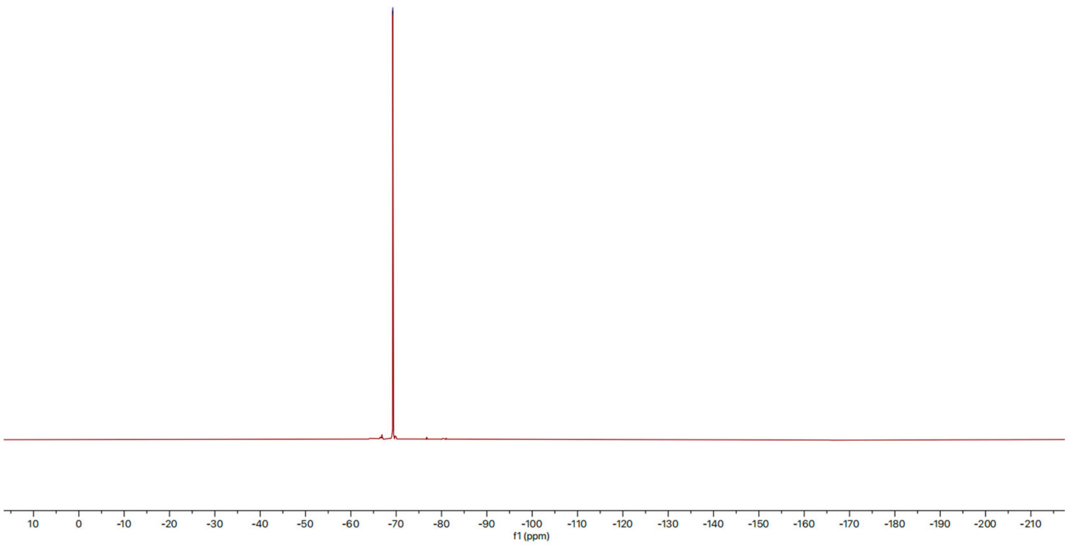


$^{19}$F NMR spectrum (376 MHz, Chloroform-*d*) of compound **50**

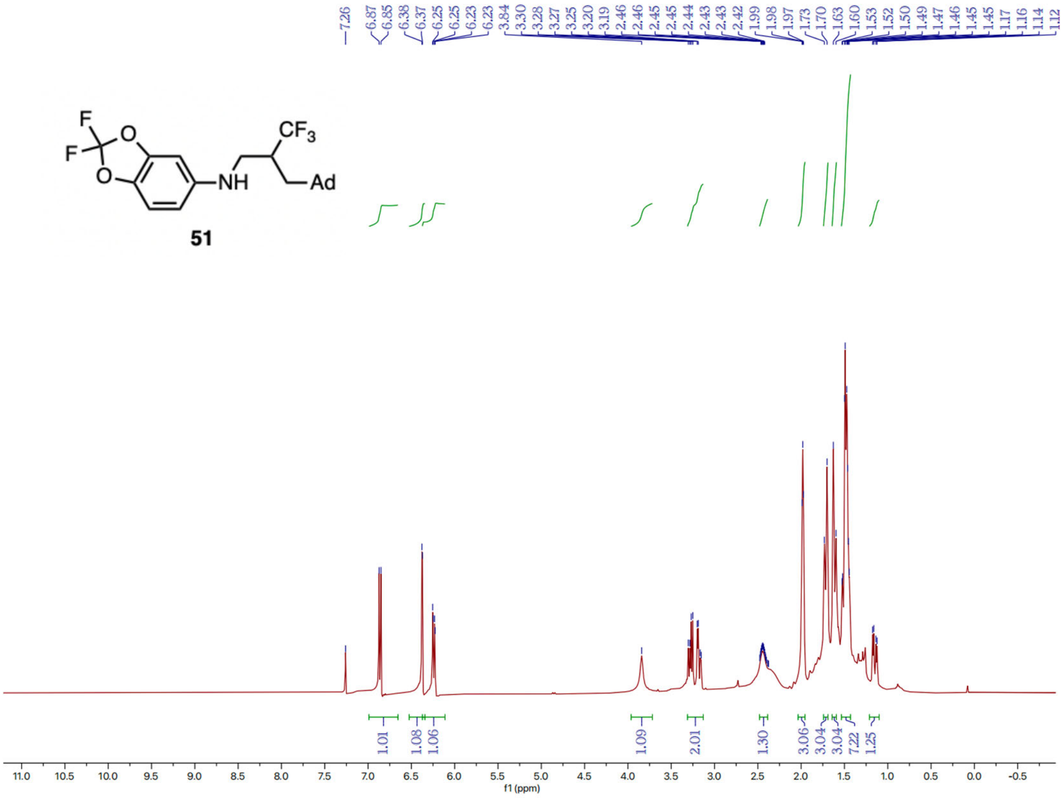

$^{1}$H NMR spectrum (400 MHz, Chloroform-*d*) of compound **51**

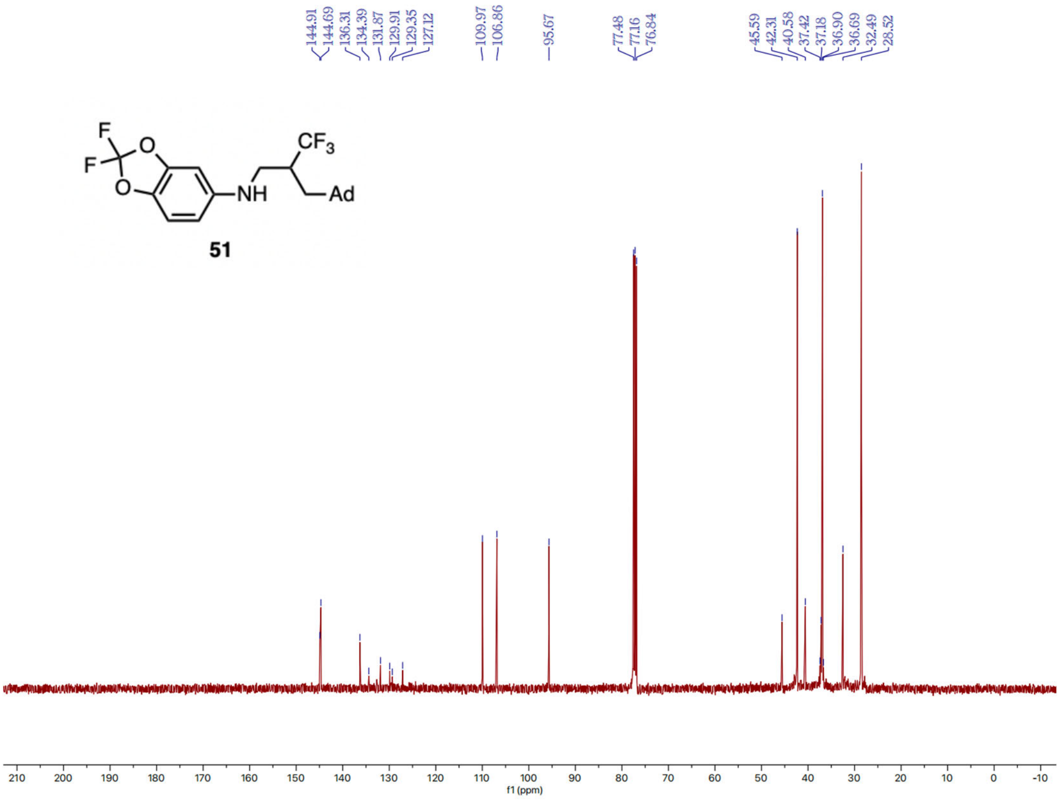

$^{13}$C NMR spectrum (101 MHz, Chloroform-*d*) of compound **51**

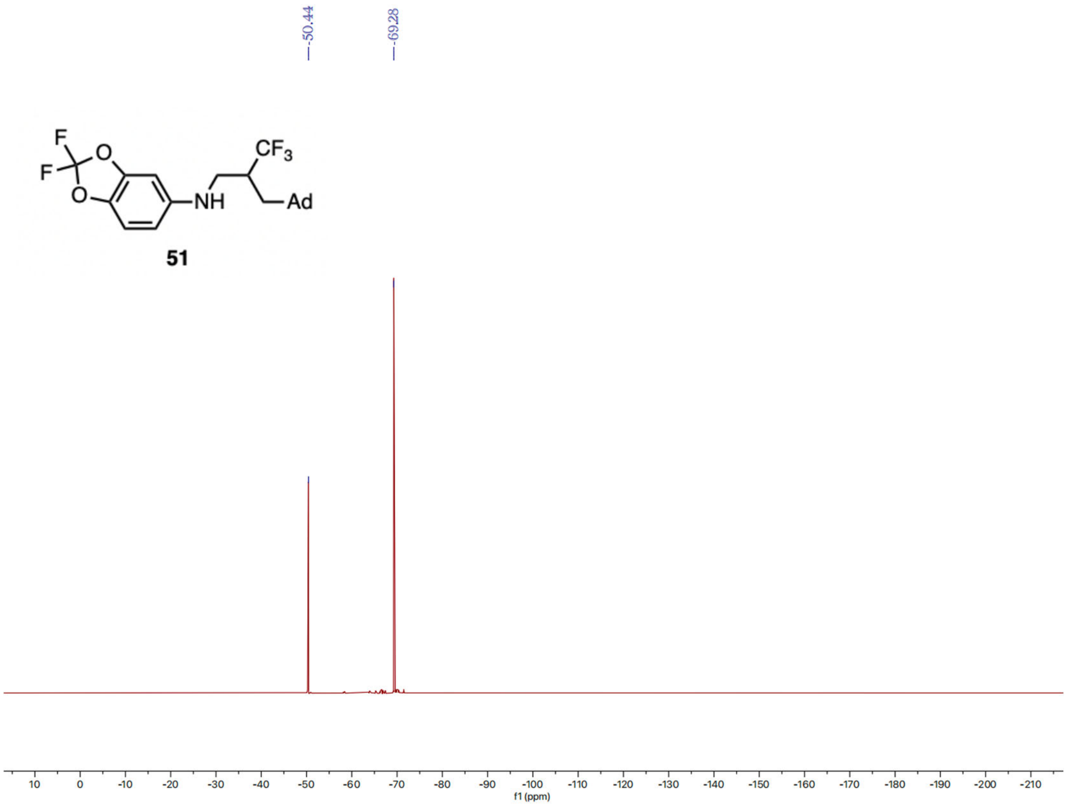


$^{19}$F NMR spectrum (376 MHz, Chloroform-*d*) of compound **51**

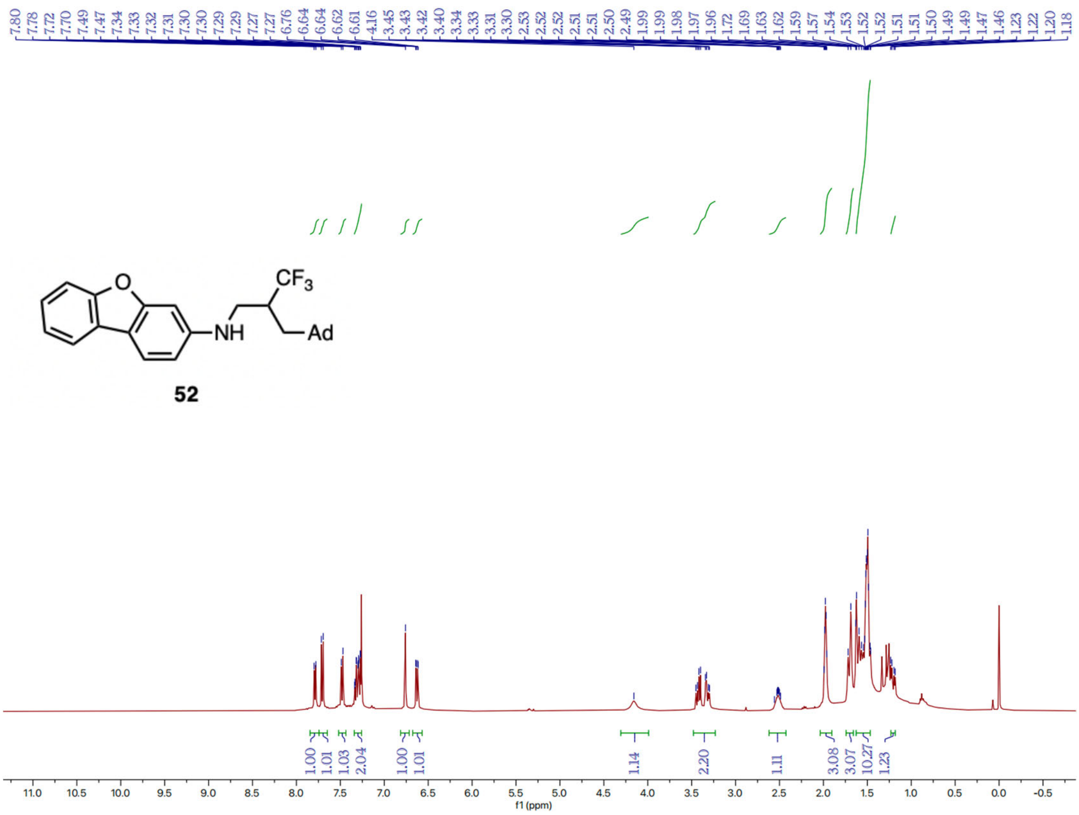

$^{1}H$ NMR spectrum (400 MHz, Chloroform-*d*) of compound **52**

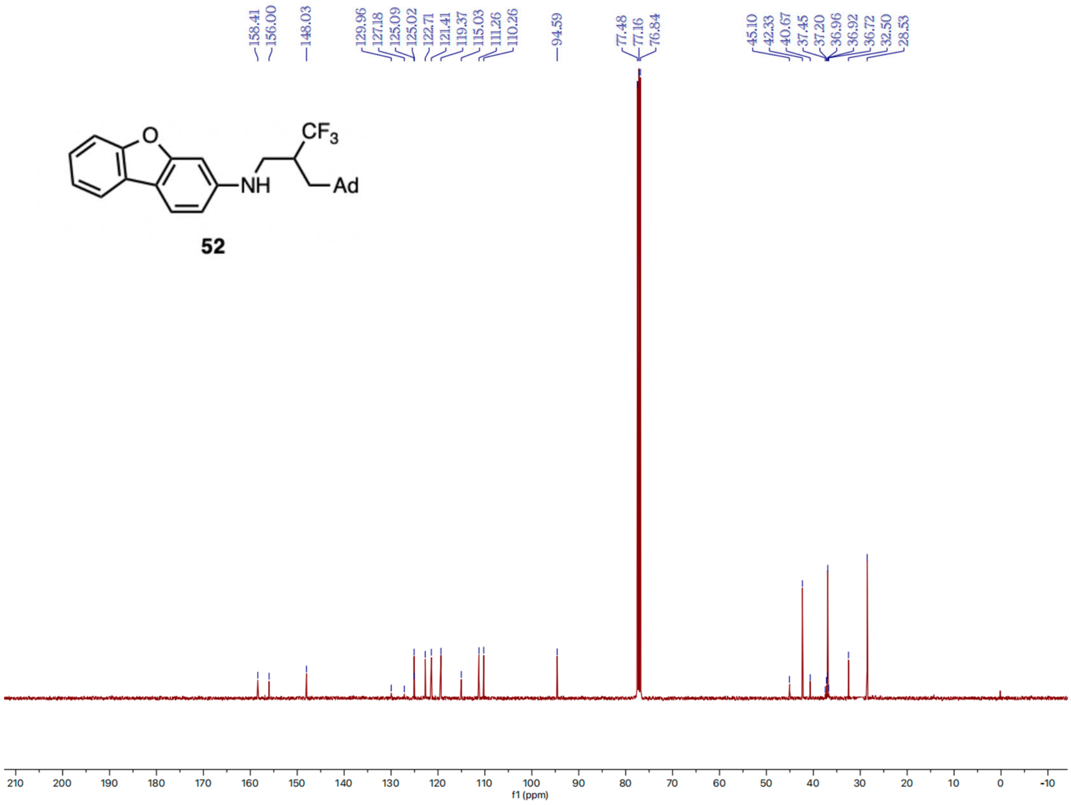

$^{13}C$ NMR spectrum (101 MHz, Chloroform-*d*) of compound **52**

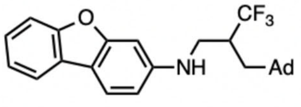


**52**

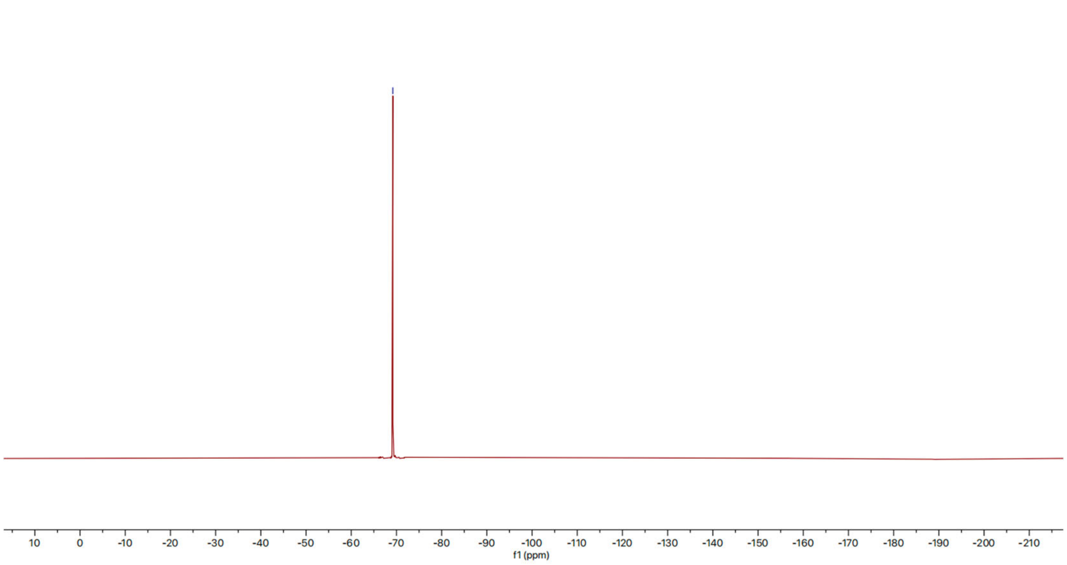


$^{19}$F NMR spectrum (376 MHz, Chloroform-*d*) of compound **52**

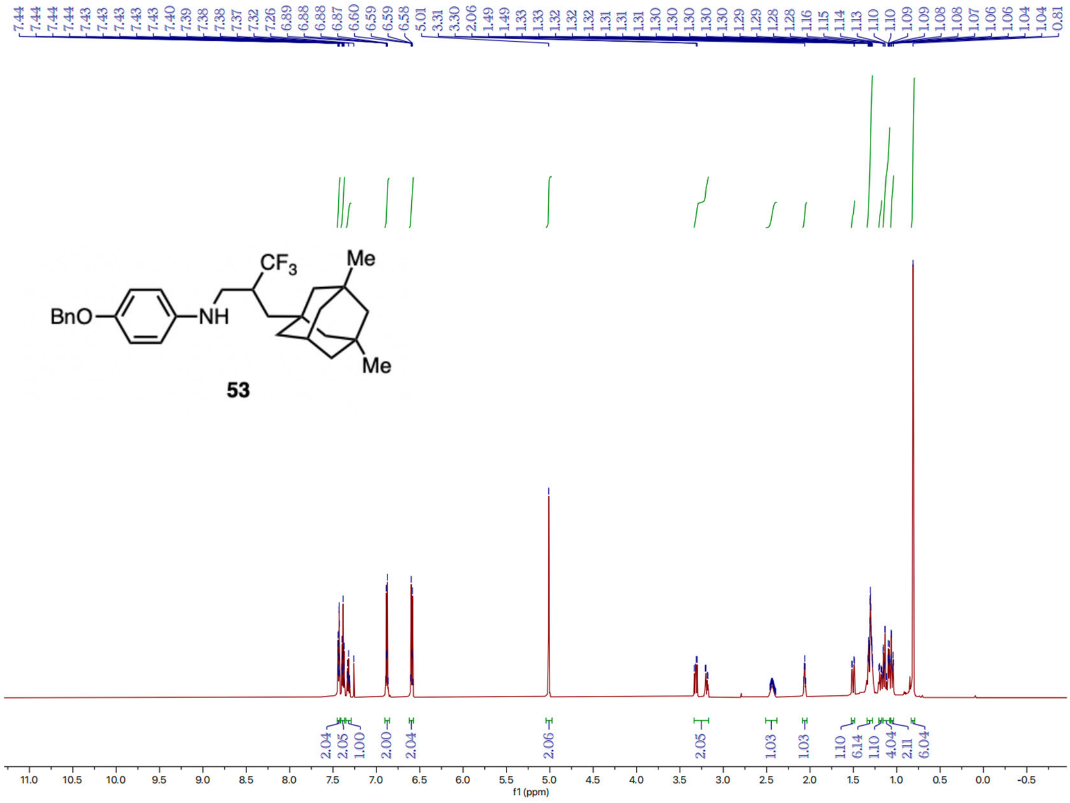

$^{1}$H NMR spectrum (600 MHz, Chloroform-*d*) of compound **53**

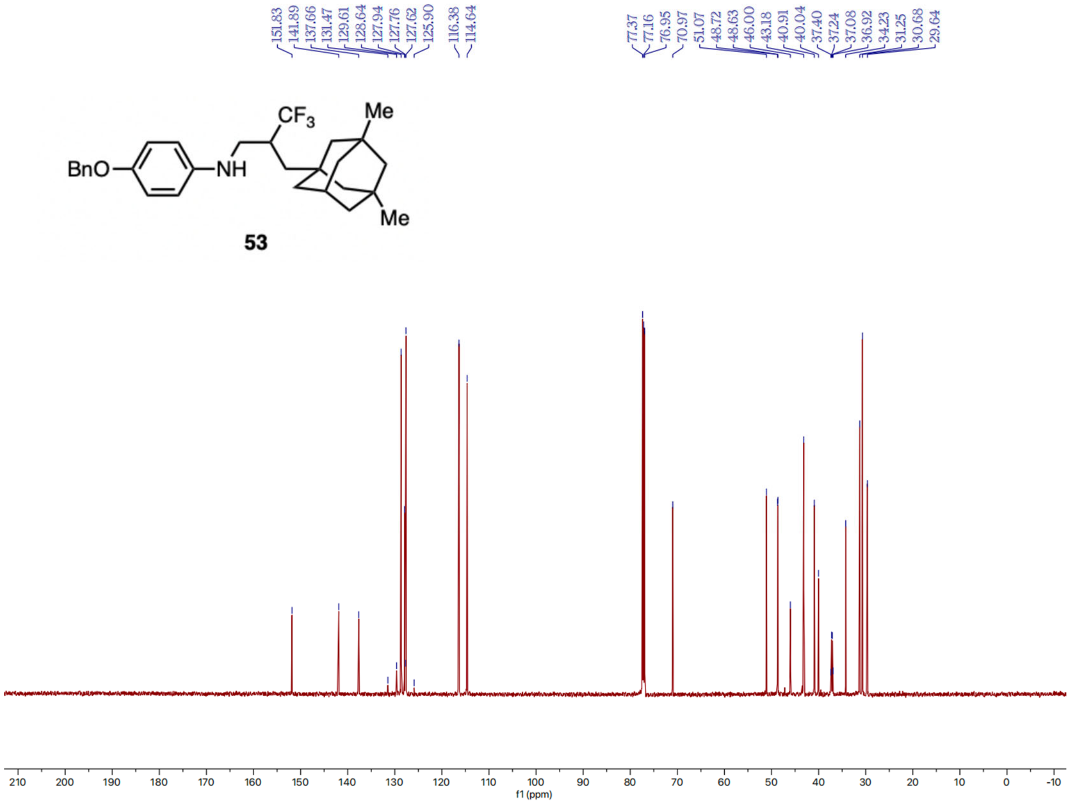

$^{13}$C NMR spectrum (151 MHz, Chloroform-*d*) of compound **53**

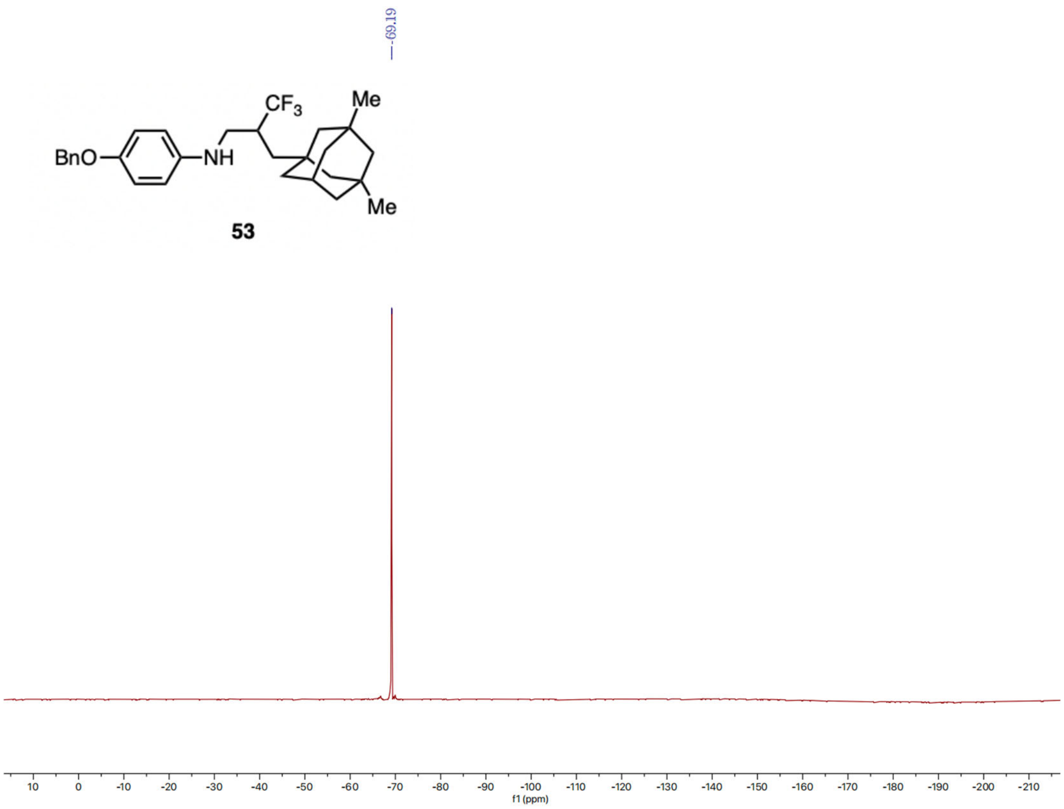


$^{19}$F NMR spectrum (376 MHz, Chloroform-*d*) of compound **53**

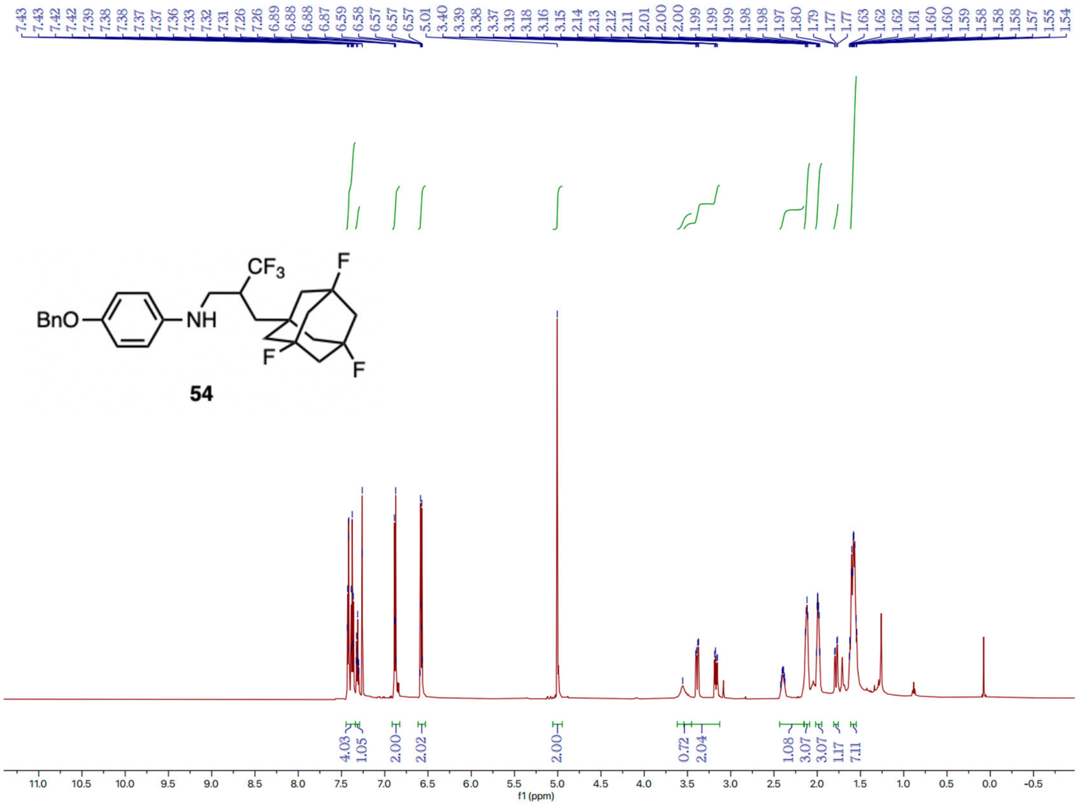


$^{1}H$ NMR spectrum (600 MHz, Chloroform-*d*) of compound **54**

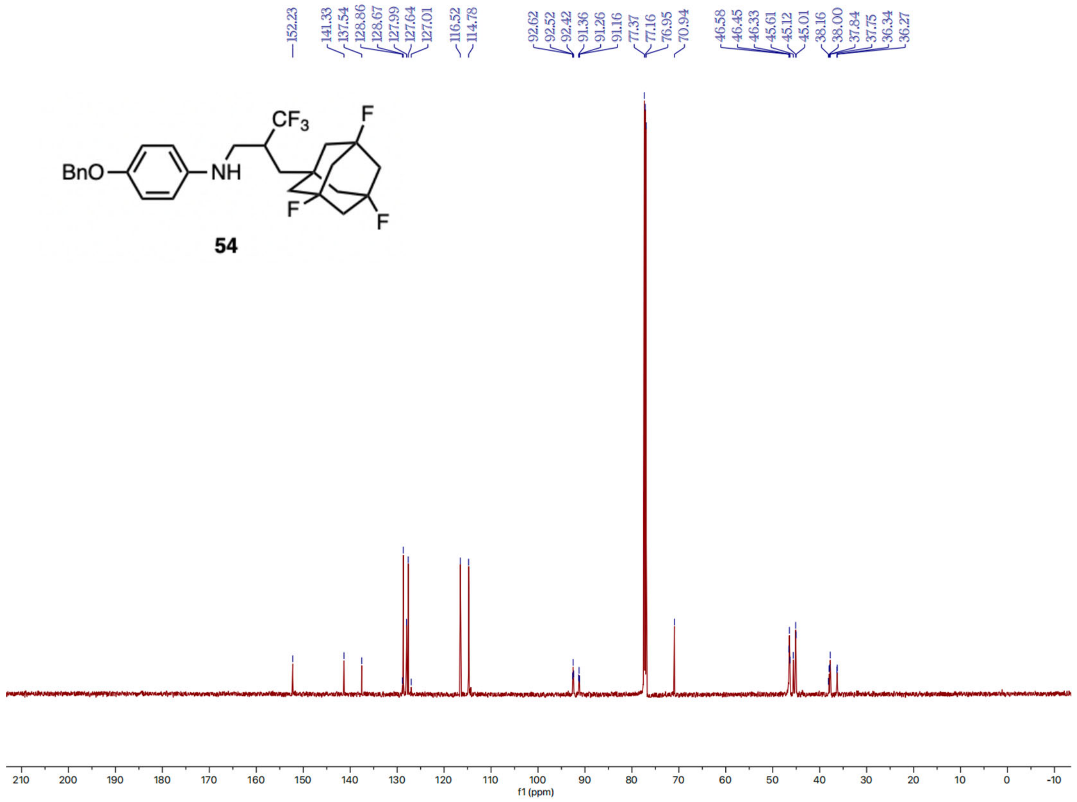


$^{13}C$ NMR spectrum (151 MHz, Chloroform-*d*) of compound **54**

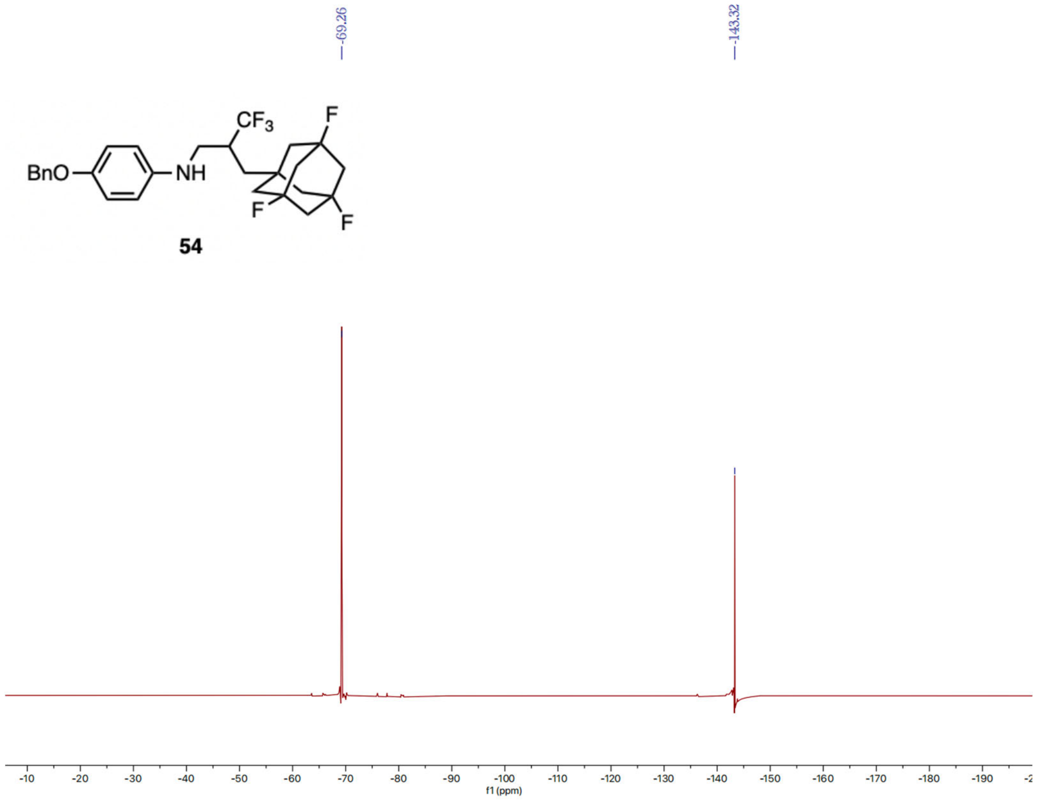


$^{19}$F NMR spectrum (565 MHz, Chloroform-*d*) of compound **54**

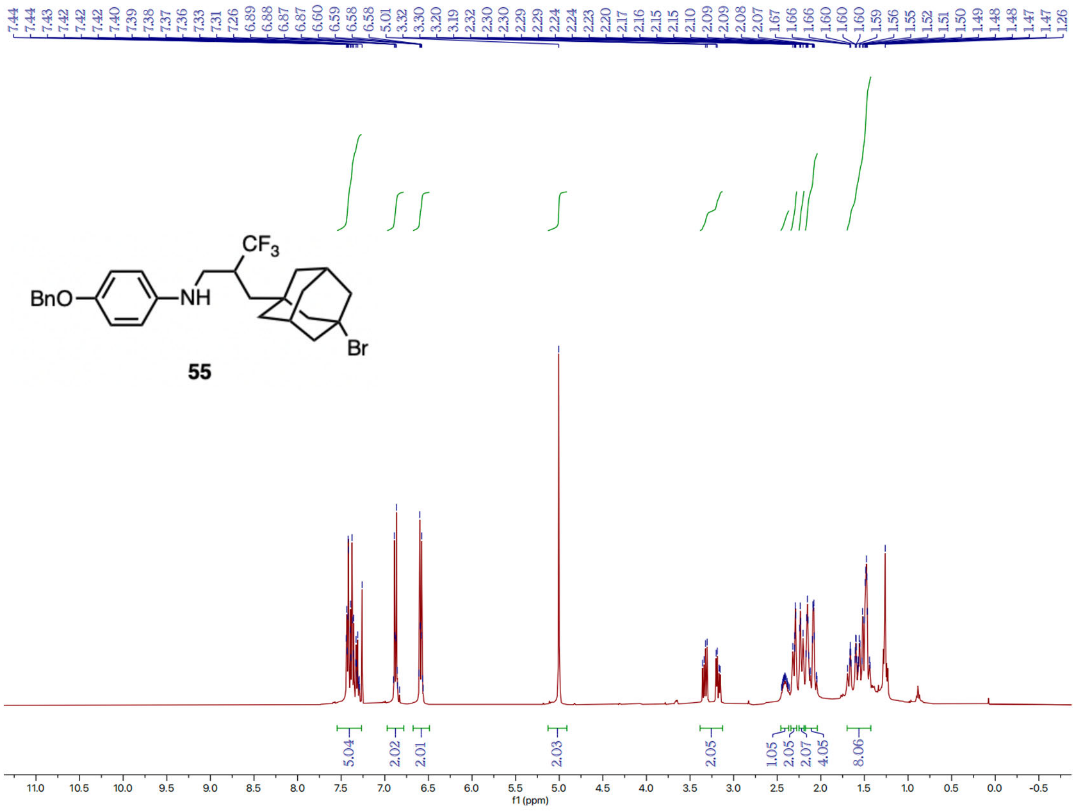

$^{1}$H NMR spectrum (400 MHz, Chloroform-*d*) of compound **55**

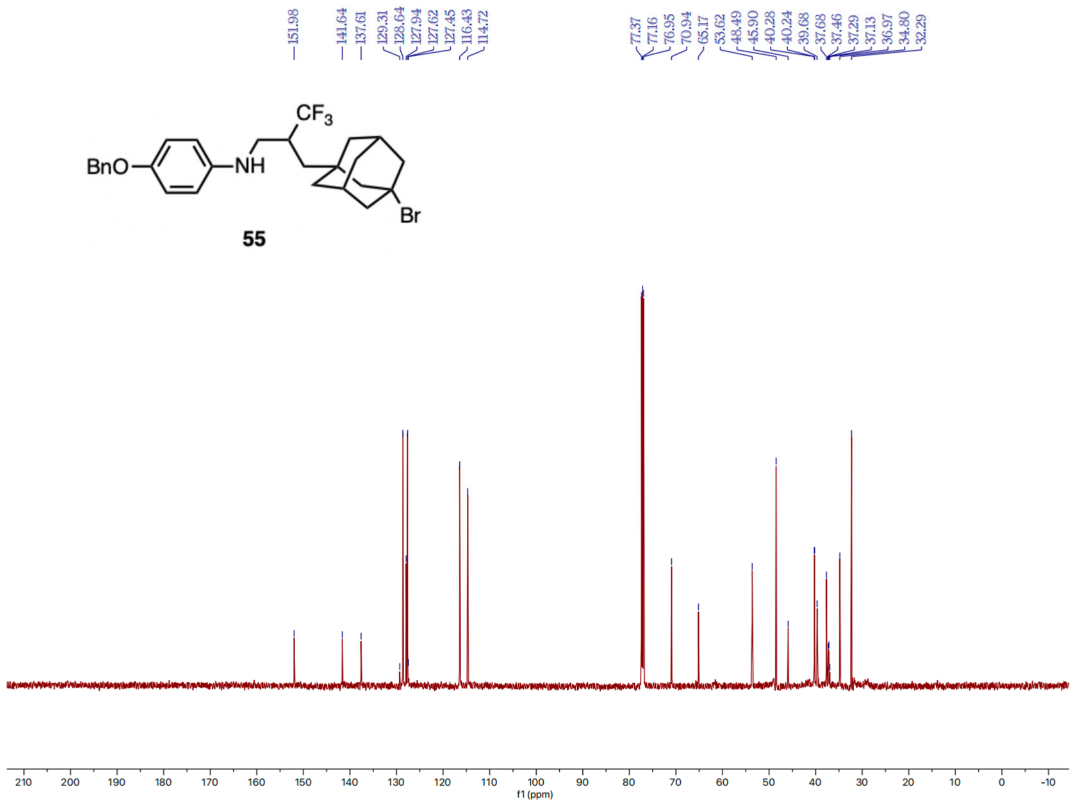

$^{13}$C NMR spectrum (151 MHz, Chloroform-*d*) of compound **55**

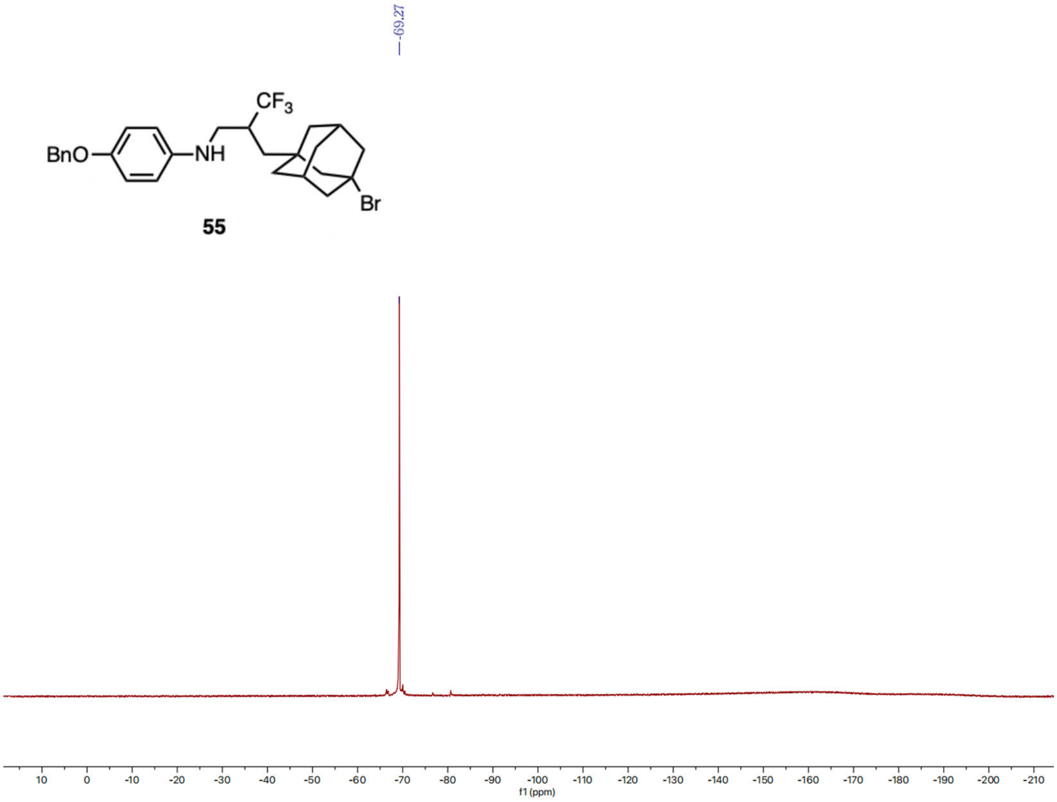


$^{19}F$ NMR spectrum (376 MHz, Chloroform-*d*) of compound **55**

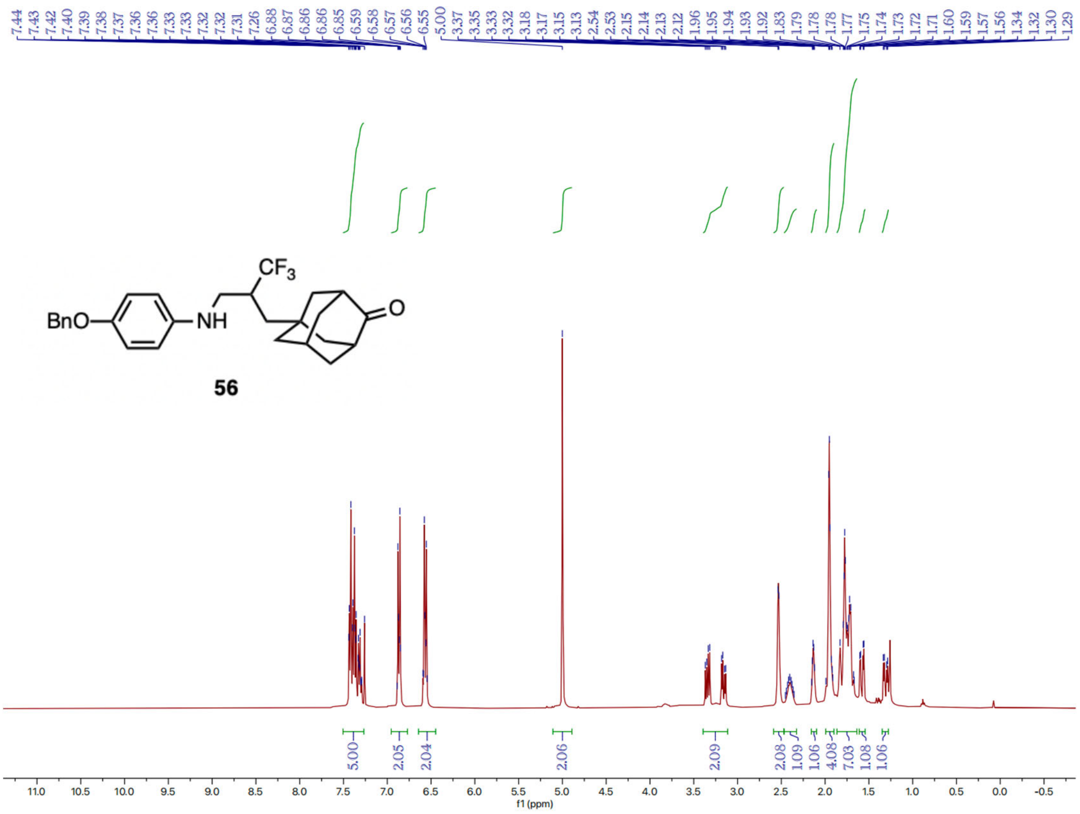

$^{1}$H NMR spectrum (400 MHz, Chloroform-*d*) of compound **56**

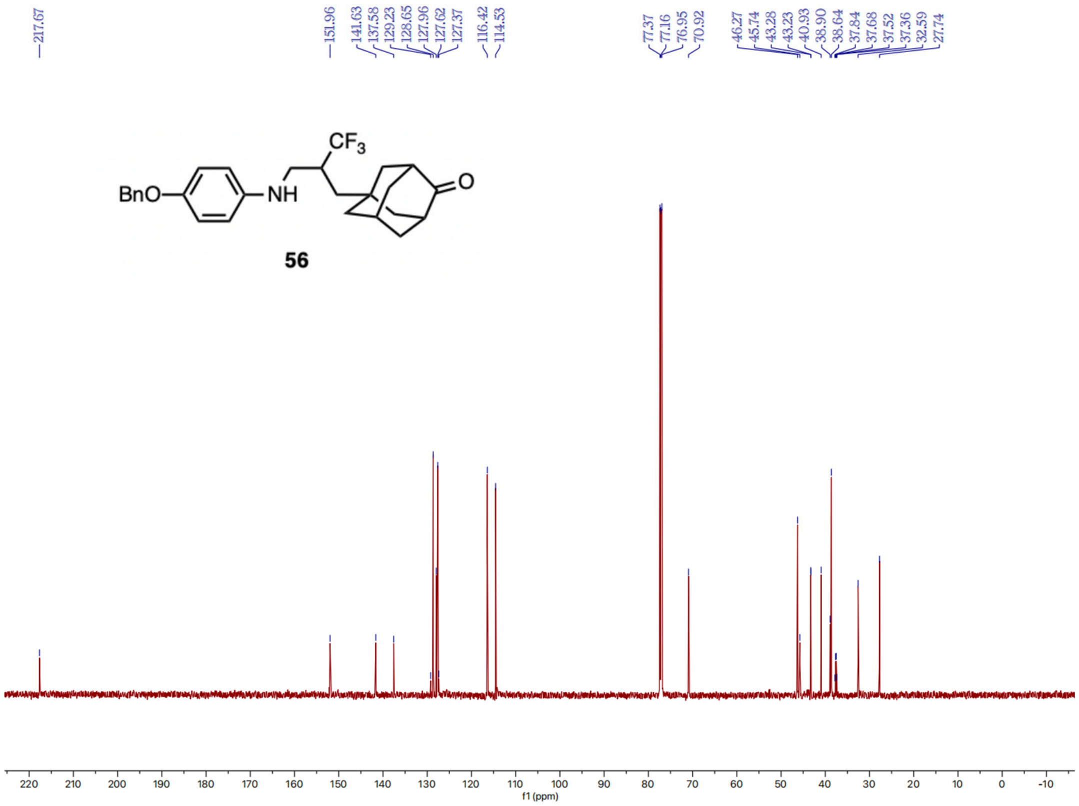

$^{13}$C NMR spectrum (151 MHz, Chloroform-*d*) of compound **56**

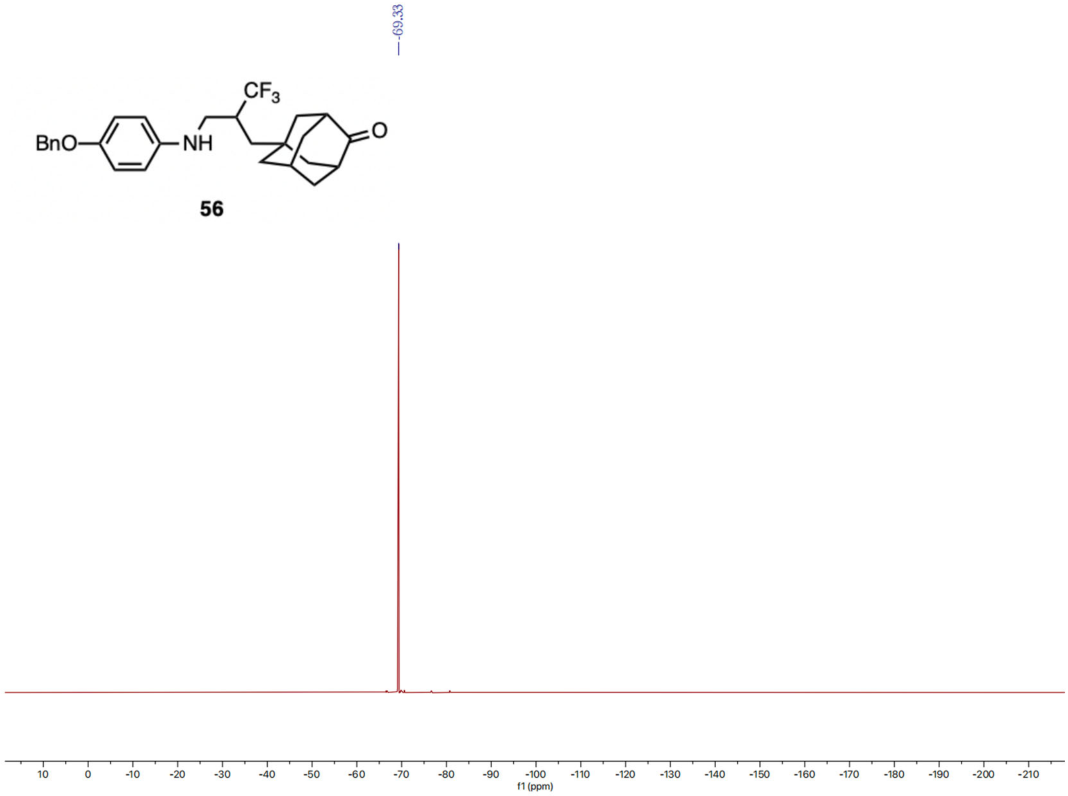


$^{19}$F NMR spectrum (376 MHz, Chloroform-*d*) of compound **56**

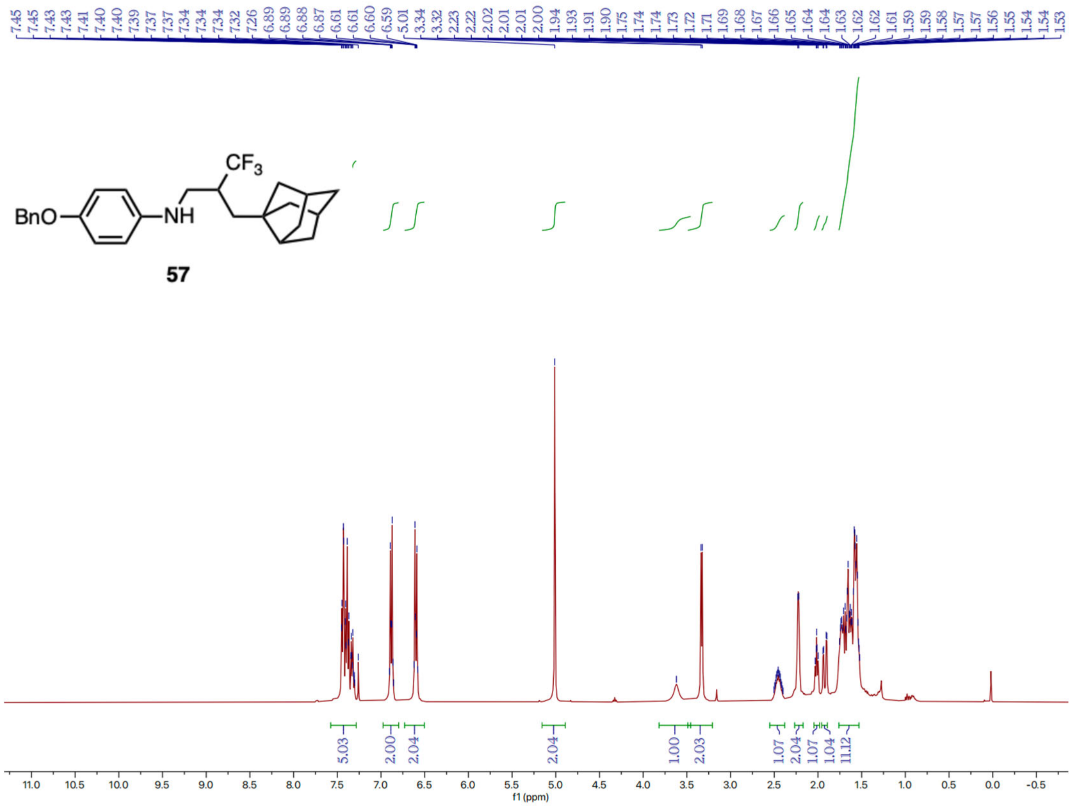

$^{1}H$ NMR spectrum (400 MHz, Chloroform-*d*) of compound **57**

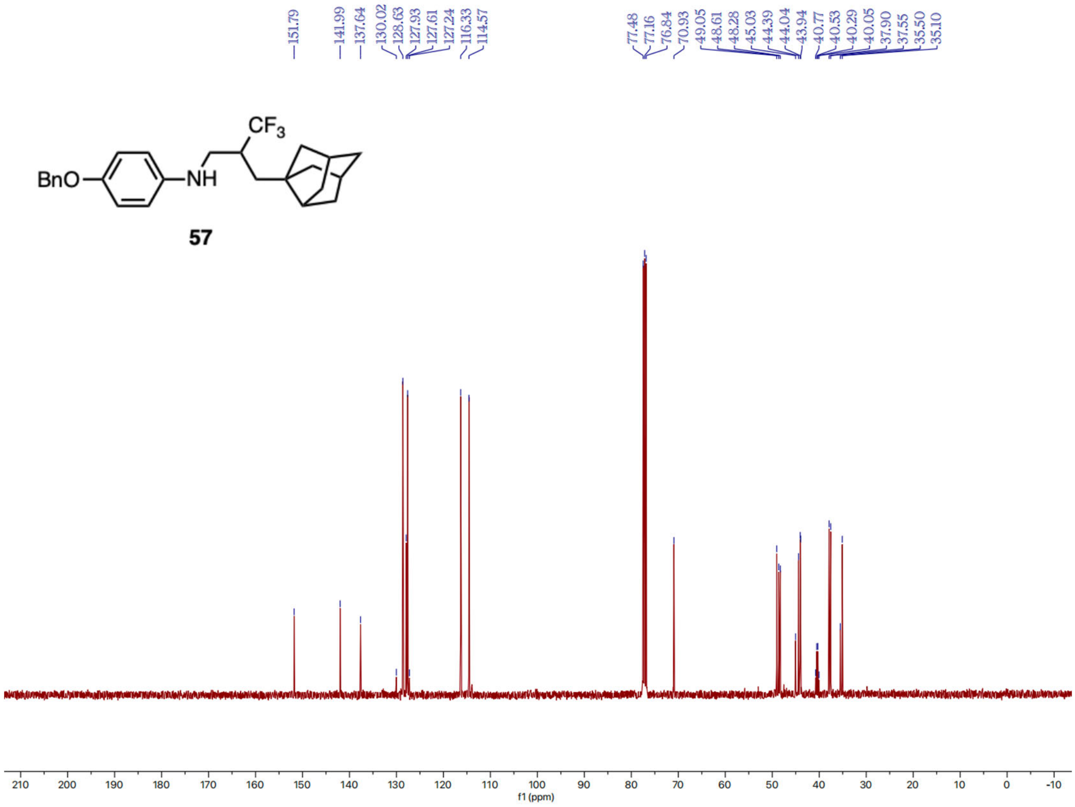

$^{13}C$ NMR spectrum (101 MHz, Chloroform-*d*) of compound **57**

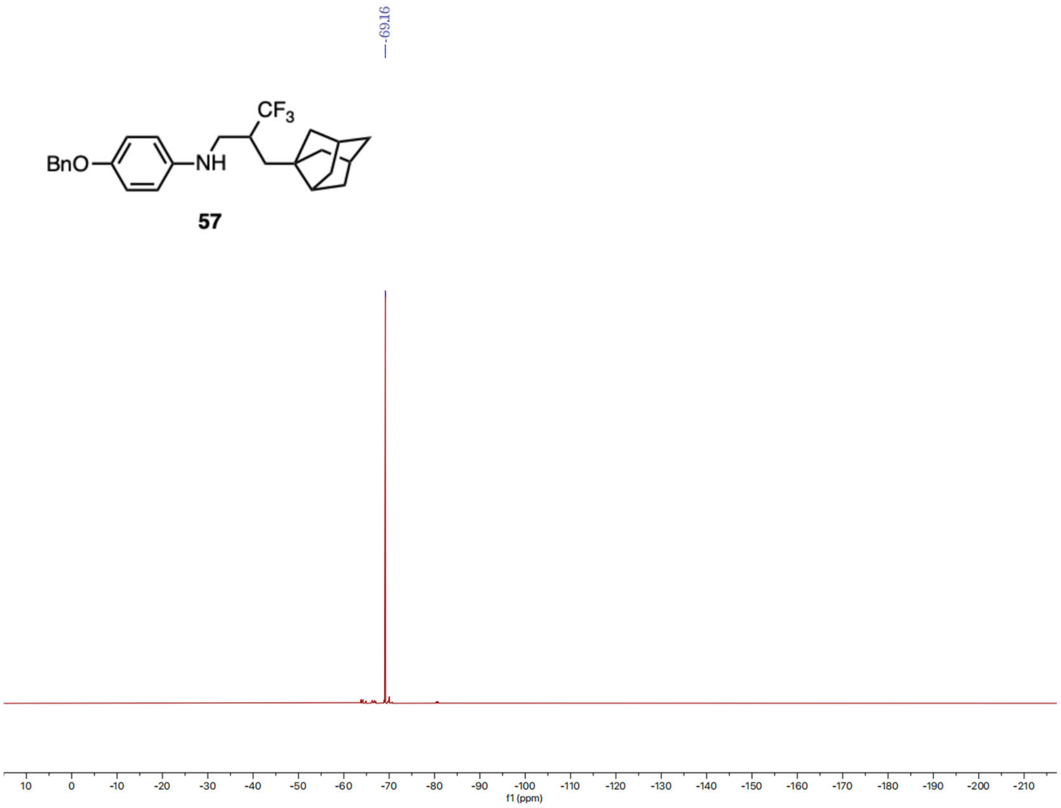


$^{19}$F NMR spectrum (376 MHz, Chloroform-*d*) of compound **57**

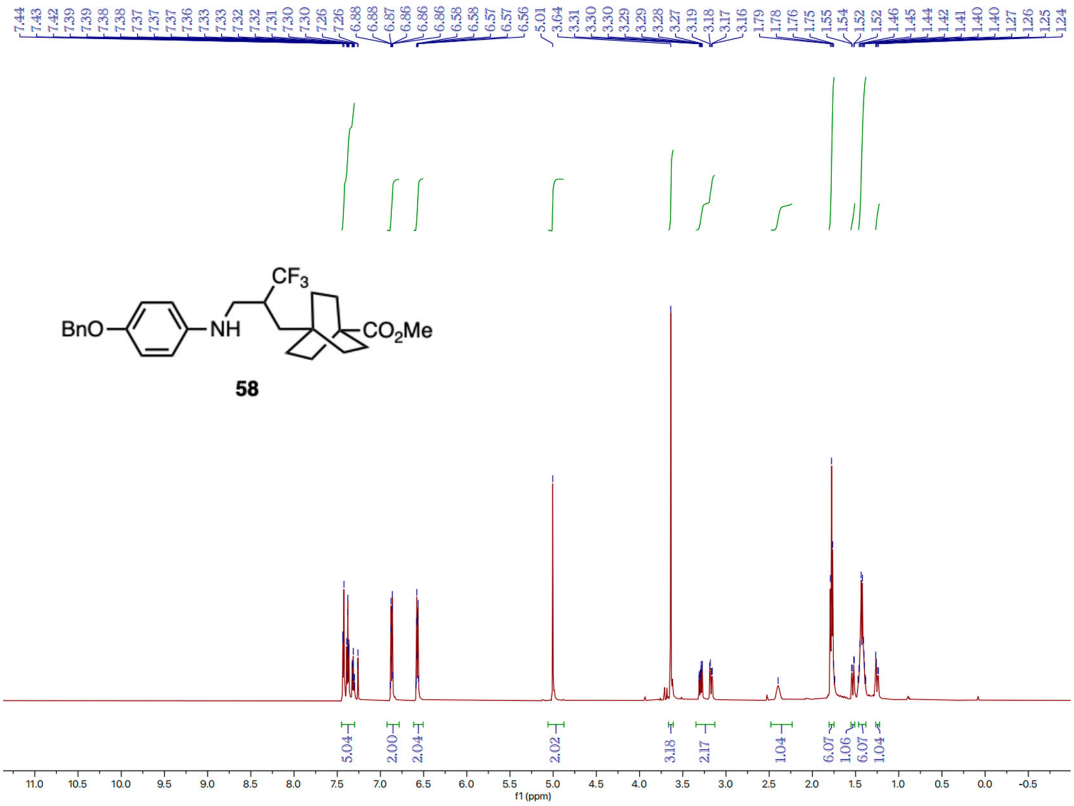

$^1$H NMR spectrum (600 MHz, Chloroform-*d*) of compound **58**

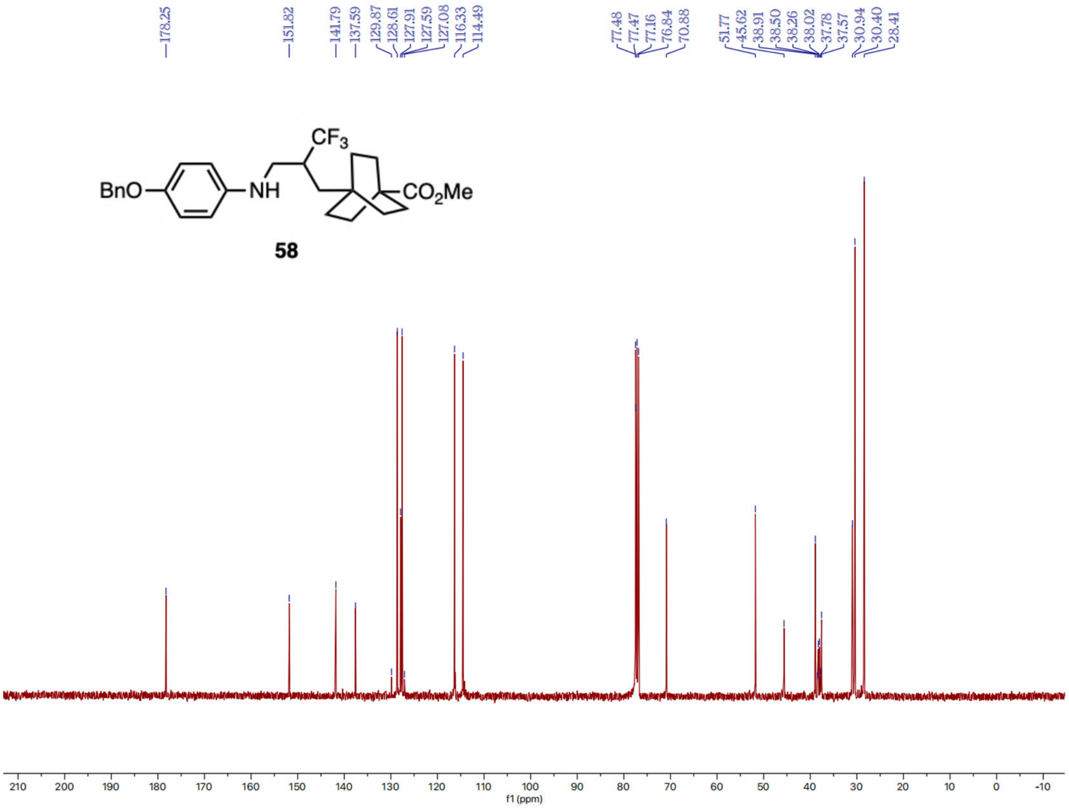

$^{13}$C NMR spectrum (101 MHz, Chloroform-*d*) of compound **58**

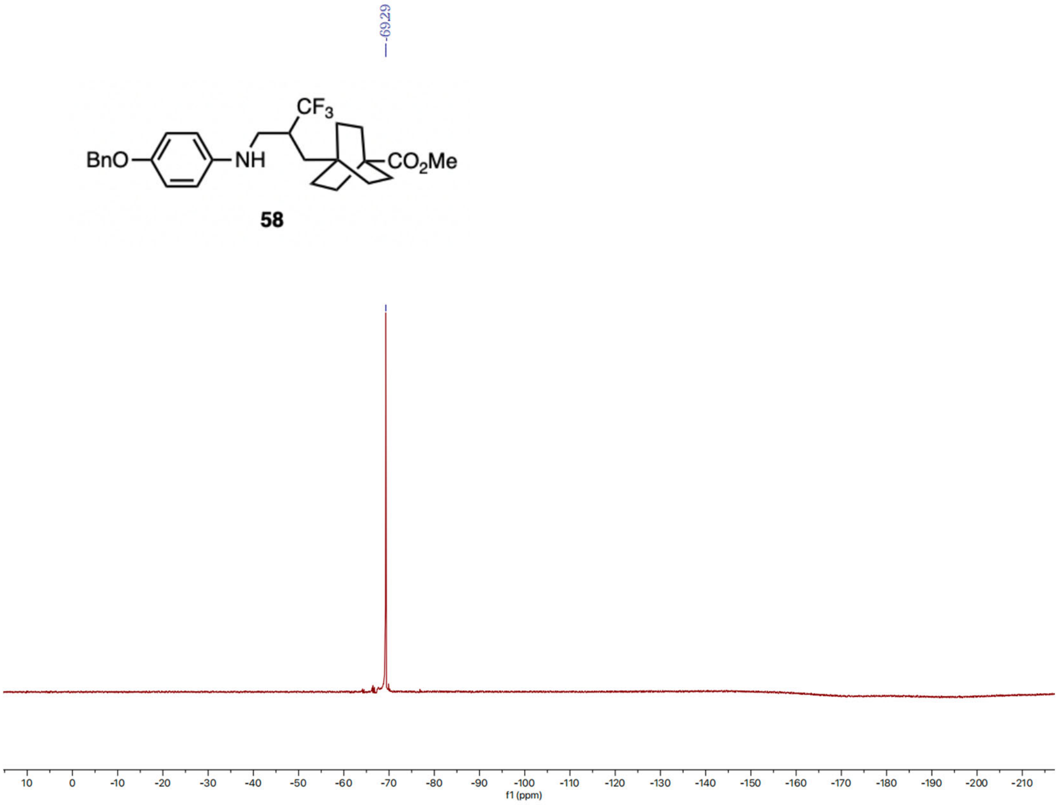

$^{19}F$ NMR spectrum (376 MHz, Chloroform-*d*) of compound **58**

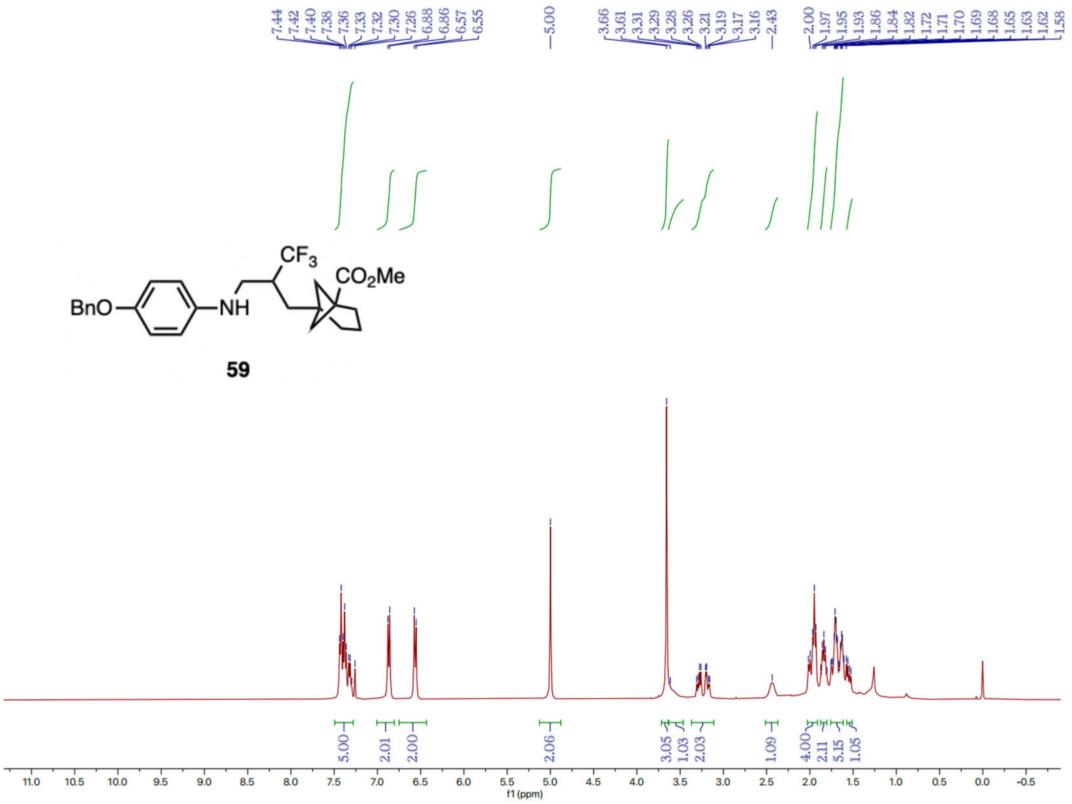

$^{1}$H NMR spectrum (400 MHz, Chloroform-*d*) of compound **59**

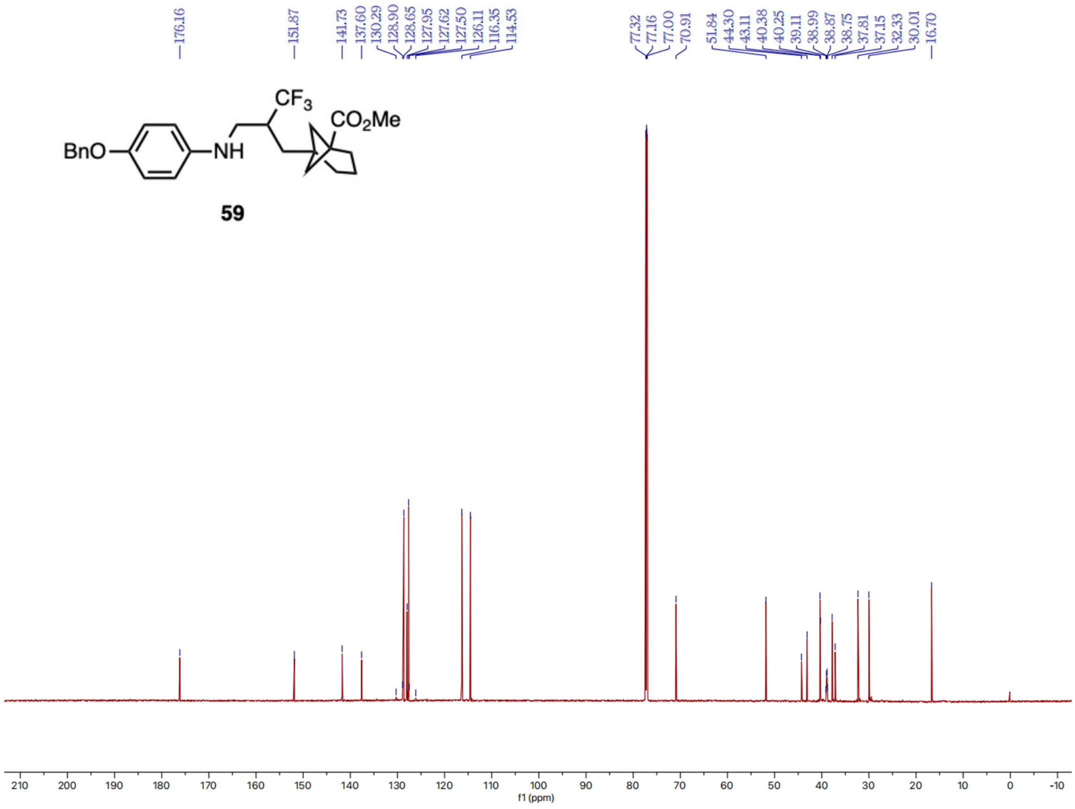

$^{13}$C NMR spectrum (201 MHz, Chloroform-*d*) of compound **59**

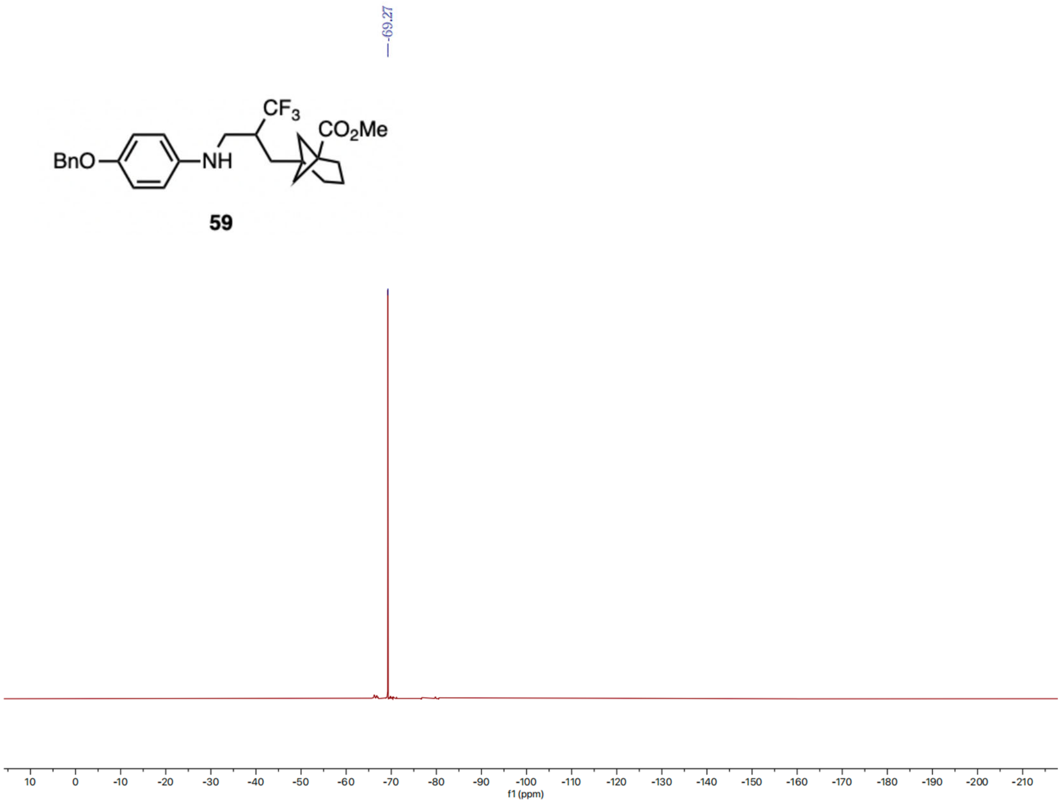


$^{19}$F NMR spectrum (376 MHz, Chloroform-*d*) of compound **59**

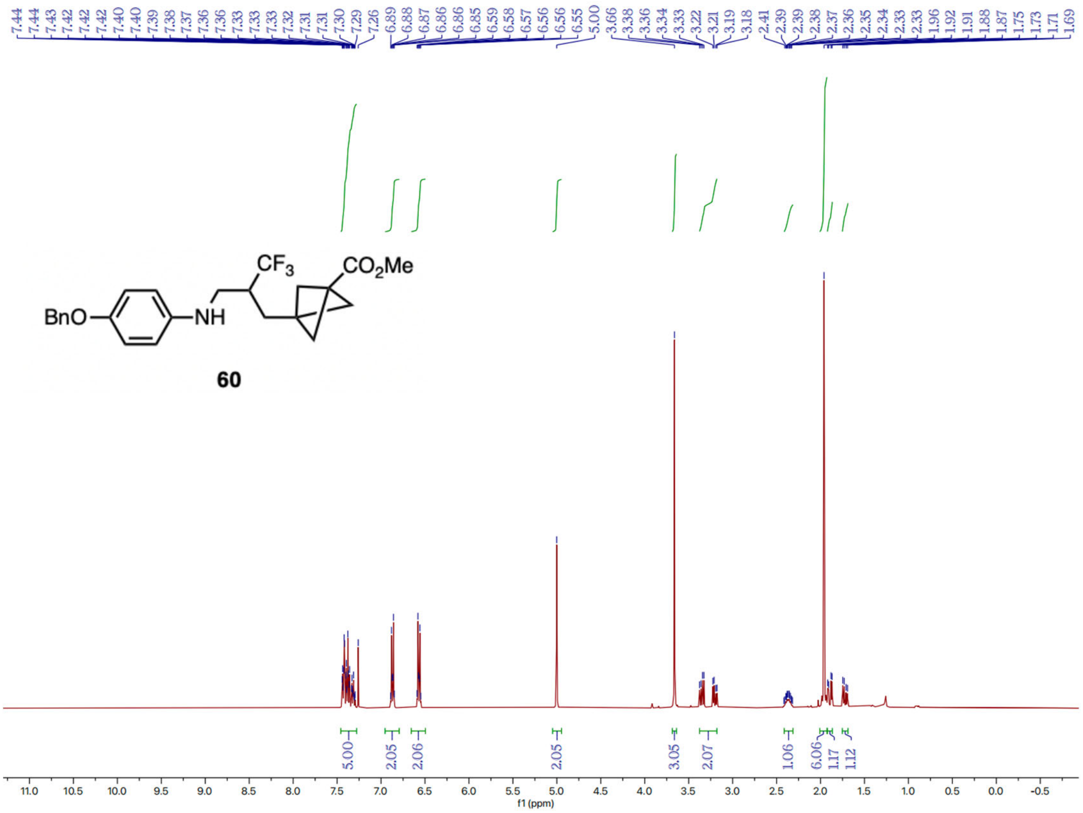

$^{1}$H NMR spectrum (400 MHz, Chloroform-*d*) of compound **60**

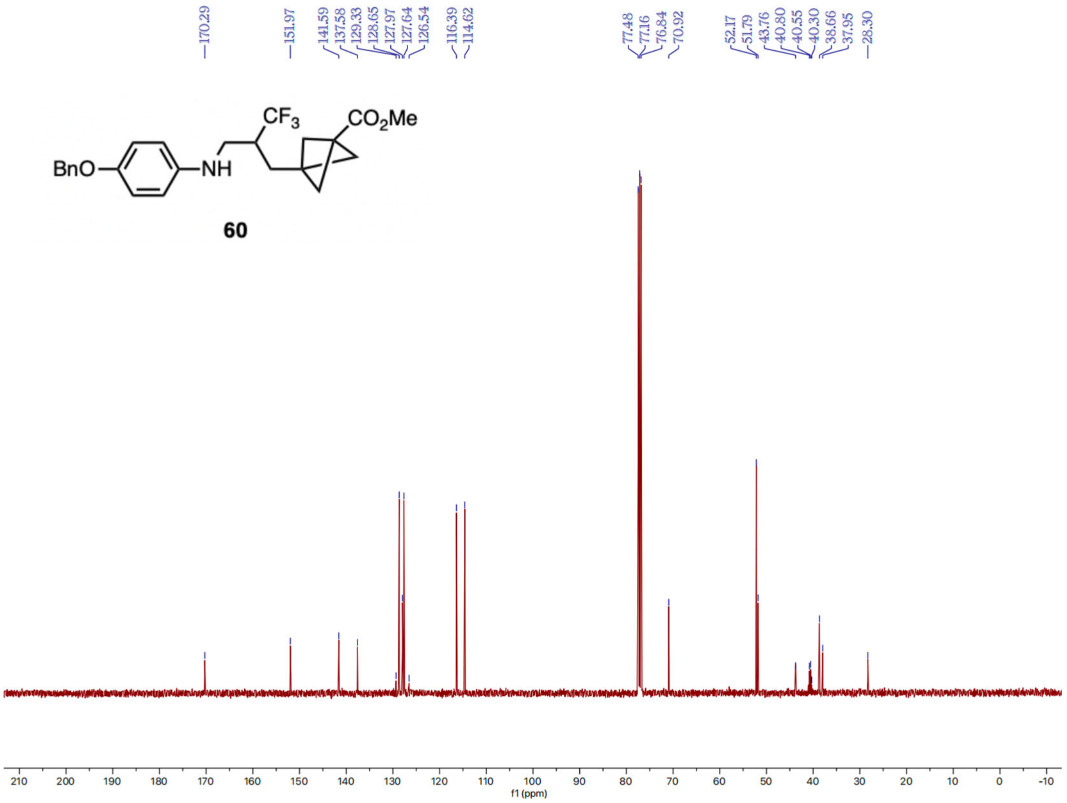

$^{13}$C NMR spectrum (101 MHz, Chloroform-*d*) of compound **60**

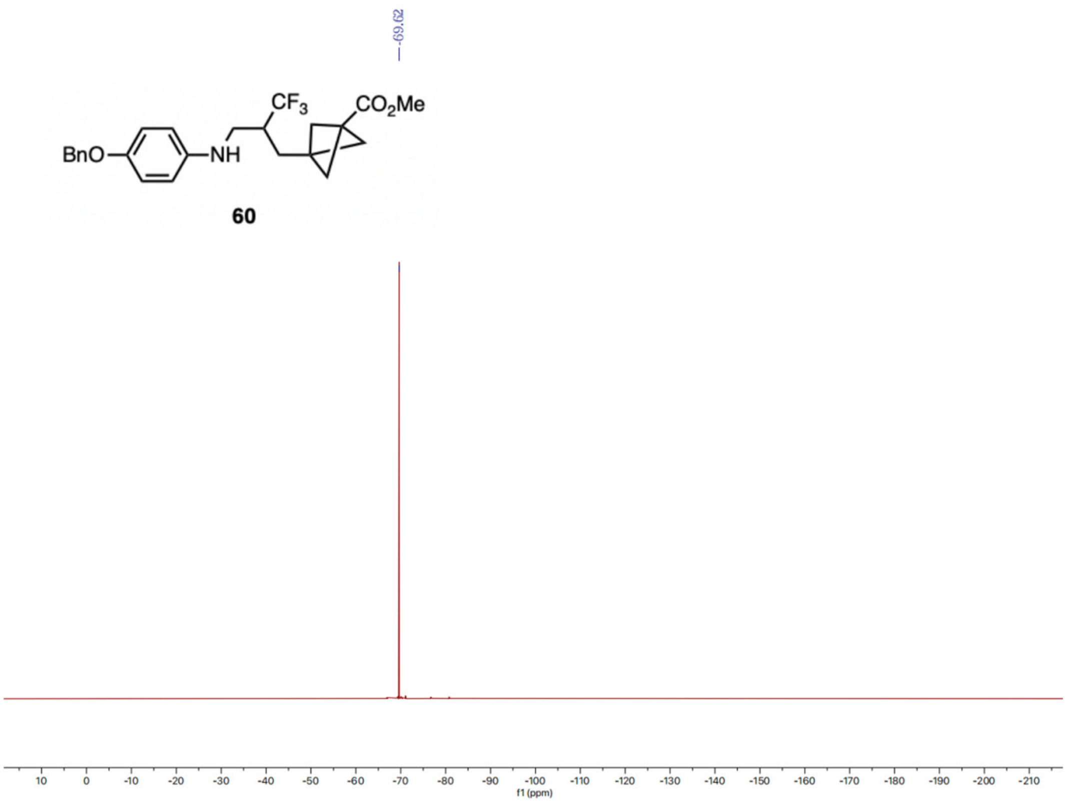


$^{19}$F NMR spectrum (376 MHz, Chloroform-*d*) of compound **60**

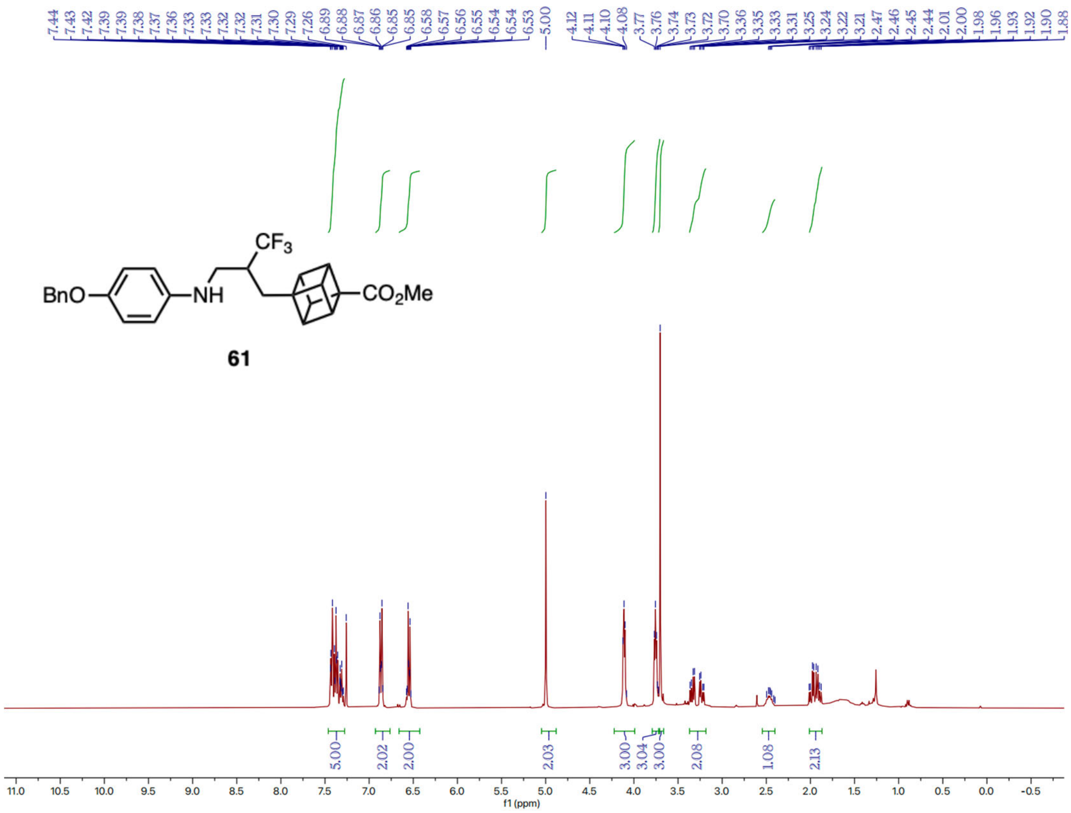


$^{1}H$ NMR spectrum (400 MHz, Chloroform-*d*) of compound **61**

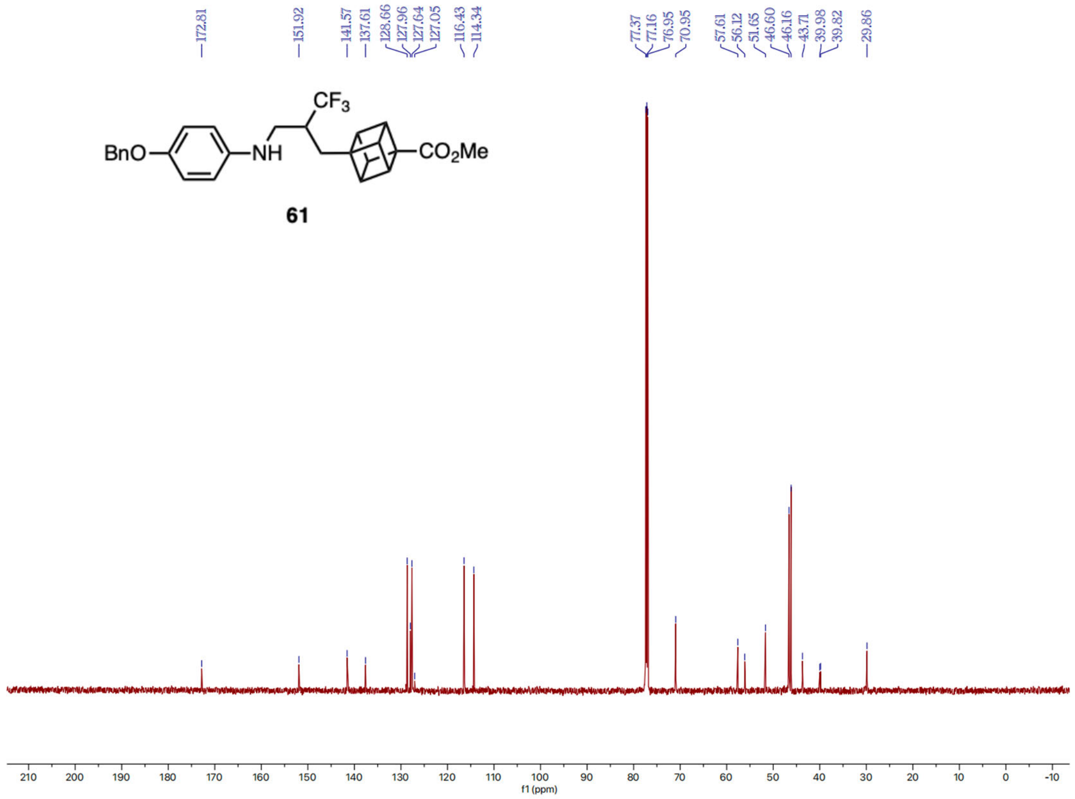


$^{13}C$ NMR spectrum (151 MHz, Chloroform-*d*) of compound **61**

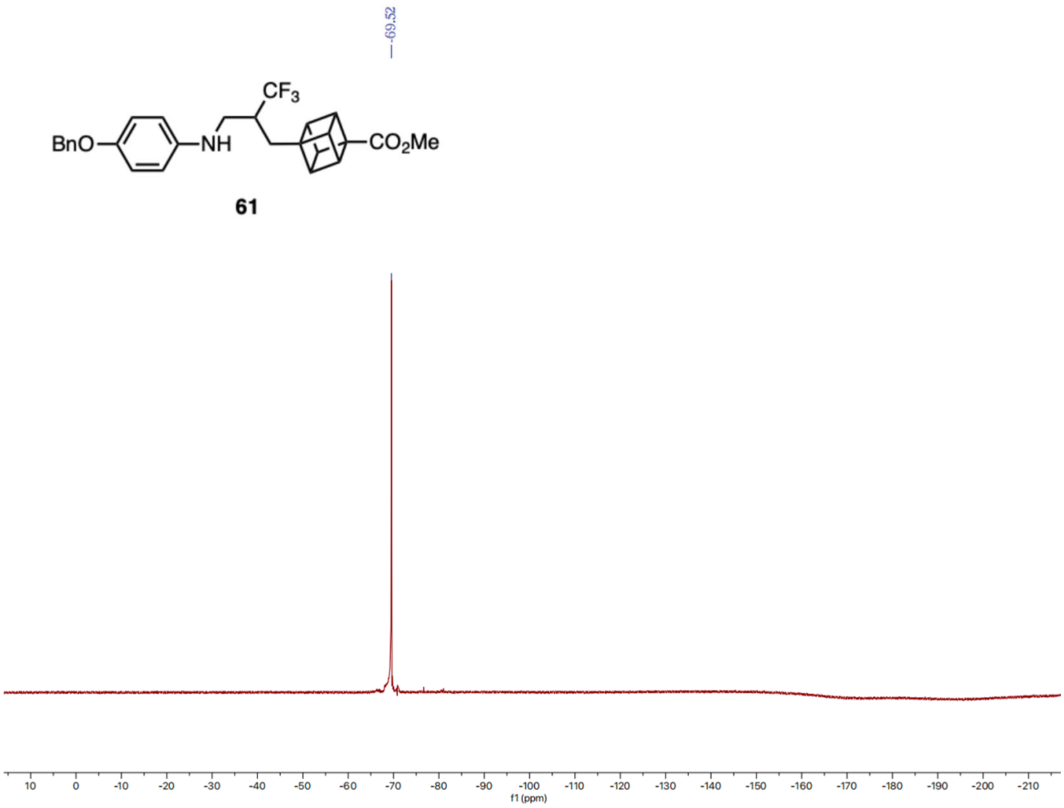


$^{19}$F NMR spectrum (376 MHz, Chloroform-*d*) of compound **61**

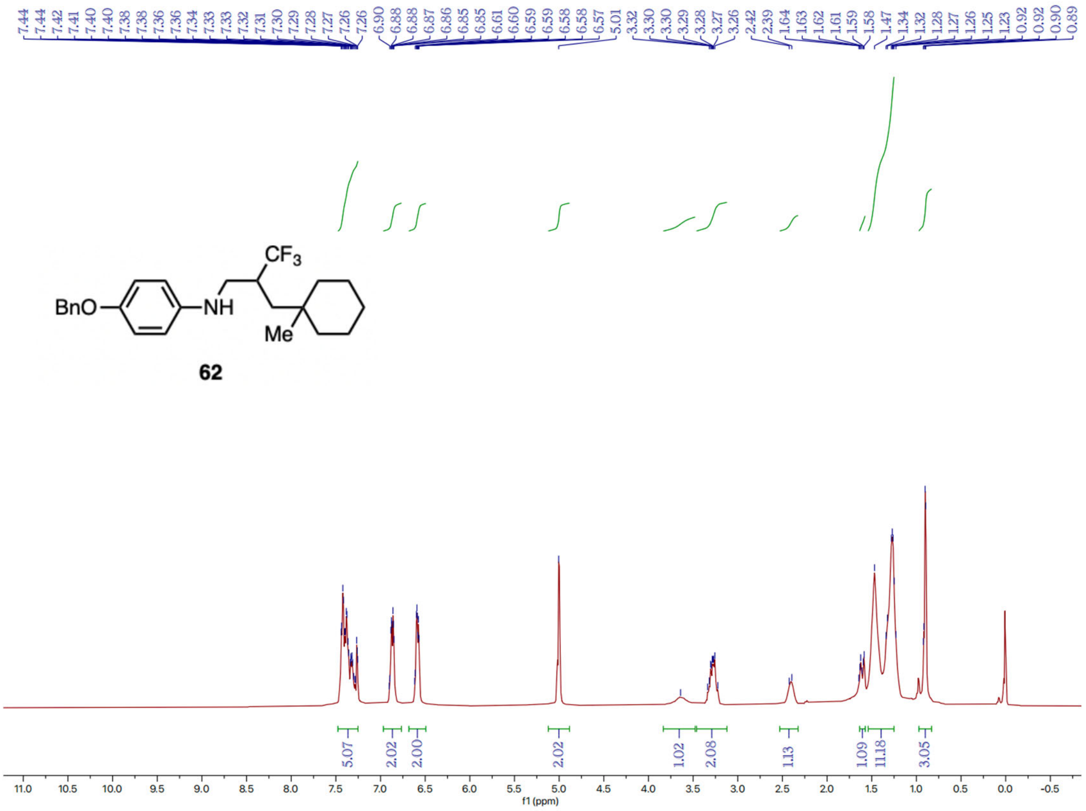


$^{1}$H NMR spectrum (400 MHz, Chloroform-*d*) of compound **62**

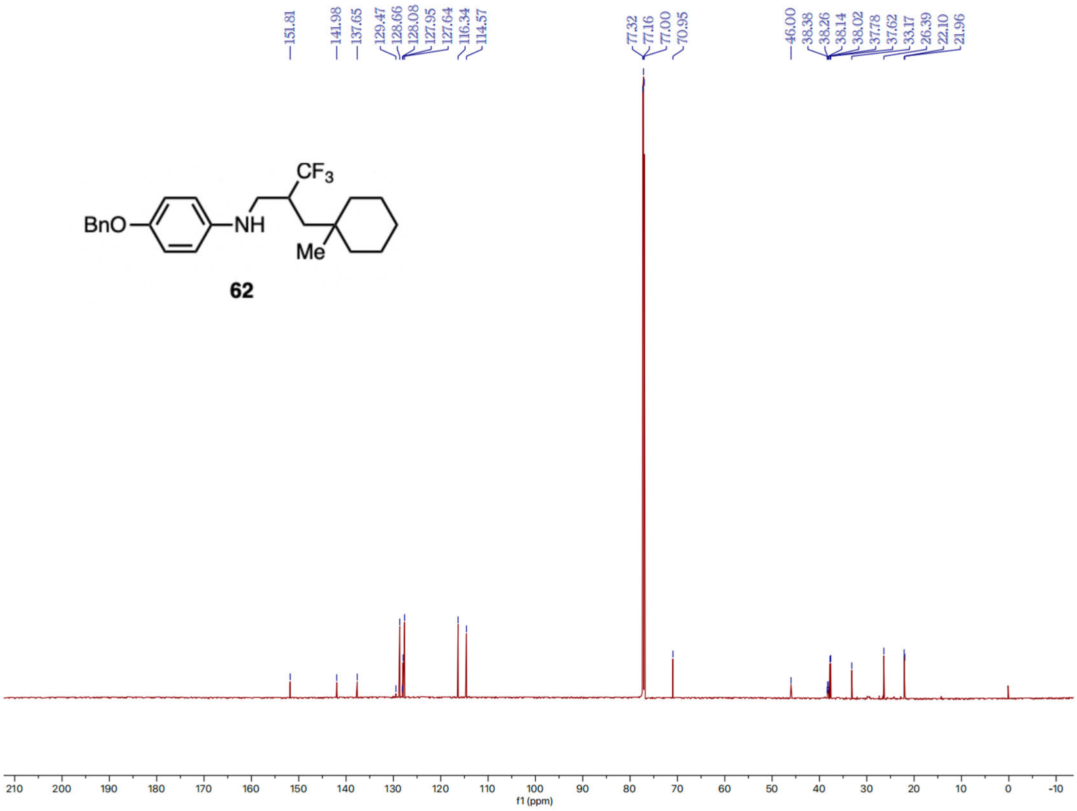


$^{13}$C NMR spectrum (201 MHz, Chloroform-*d*) of compound **62**

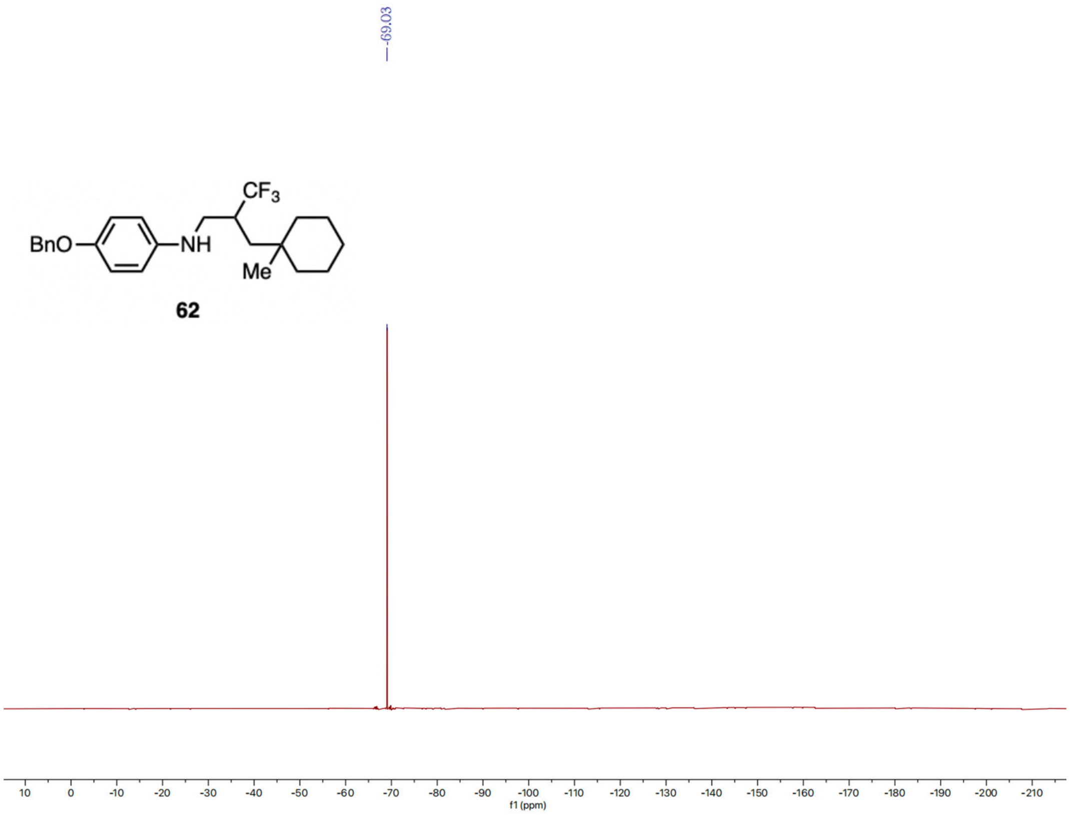


$^{19}F$ NMR spectrum (376 MHz, Chloroform-*d*) of compound **62**

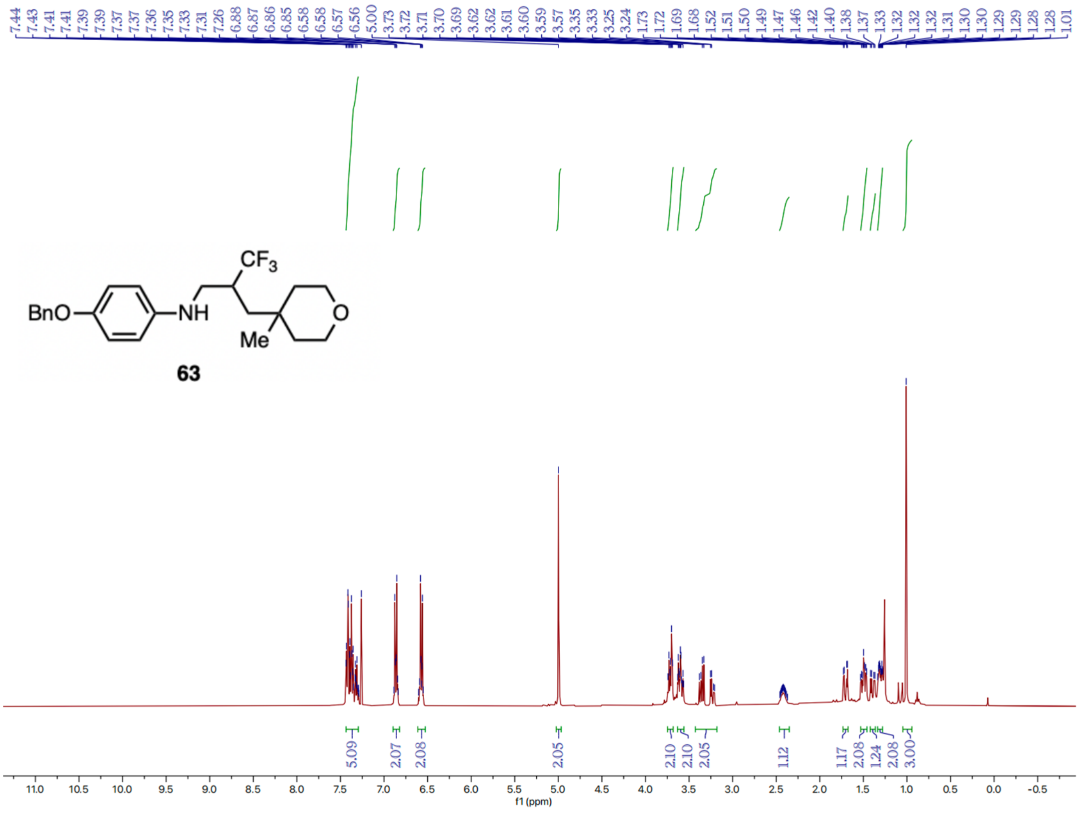

$^{1}$H NMR spectrum (400 MHz, Chloroform-*d*) of compound **63**

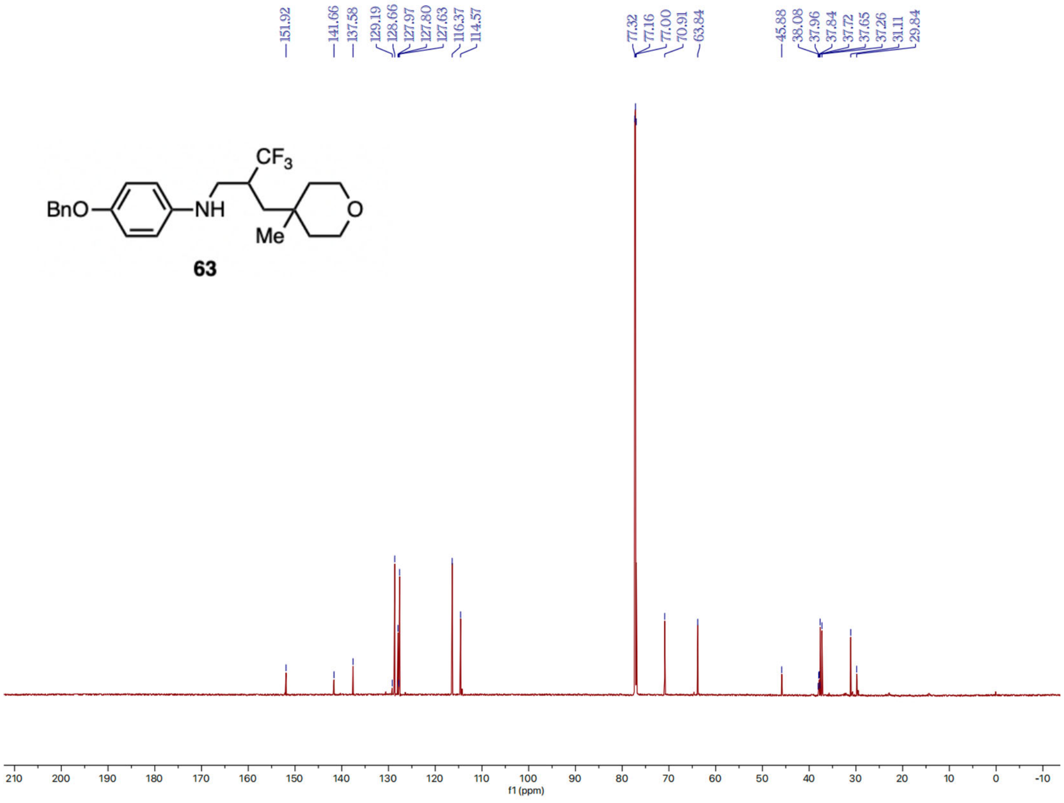

$^{13}$C NMR spectrum (201 MHz, Chloroform-*d*) of compound **63**

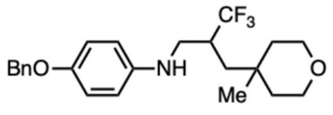


**63**

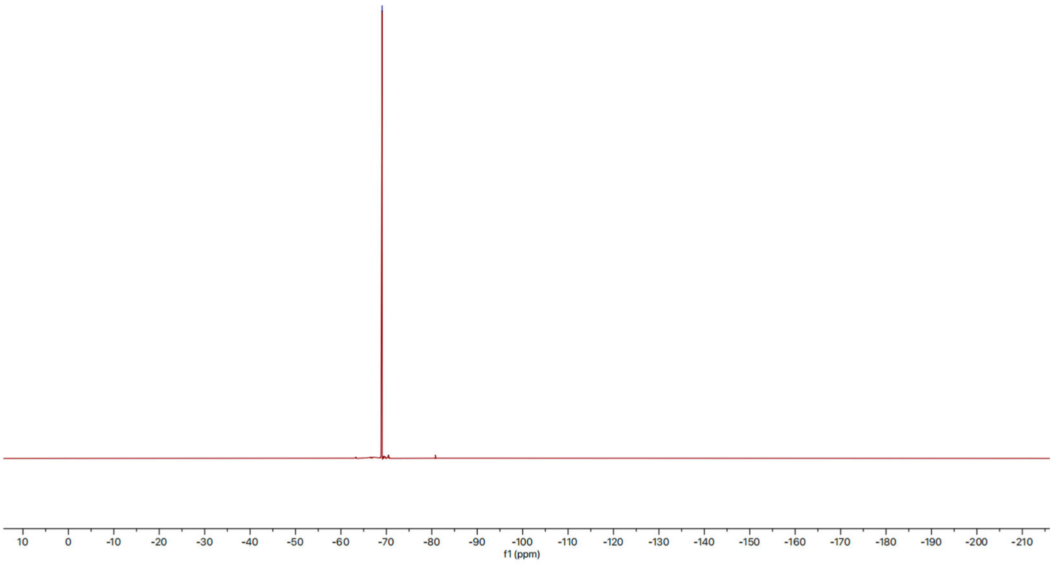


$^{19}F$ NMR spectrum (376 MHz, Chloroform-*d*) of compound **63**

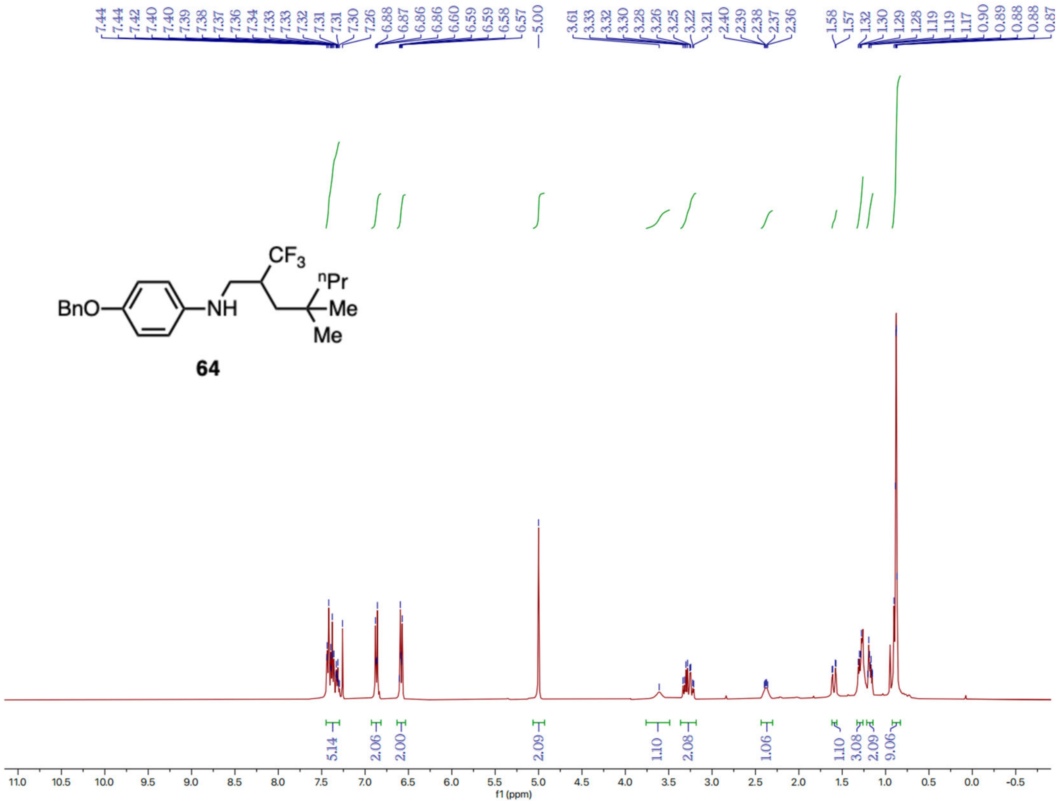


$^1$H NMR spectrum (400 MHz, Chloroform-*d*) of compound **64**

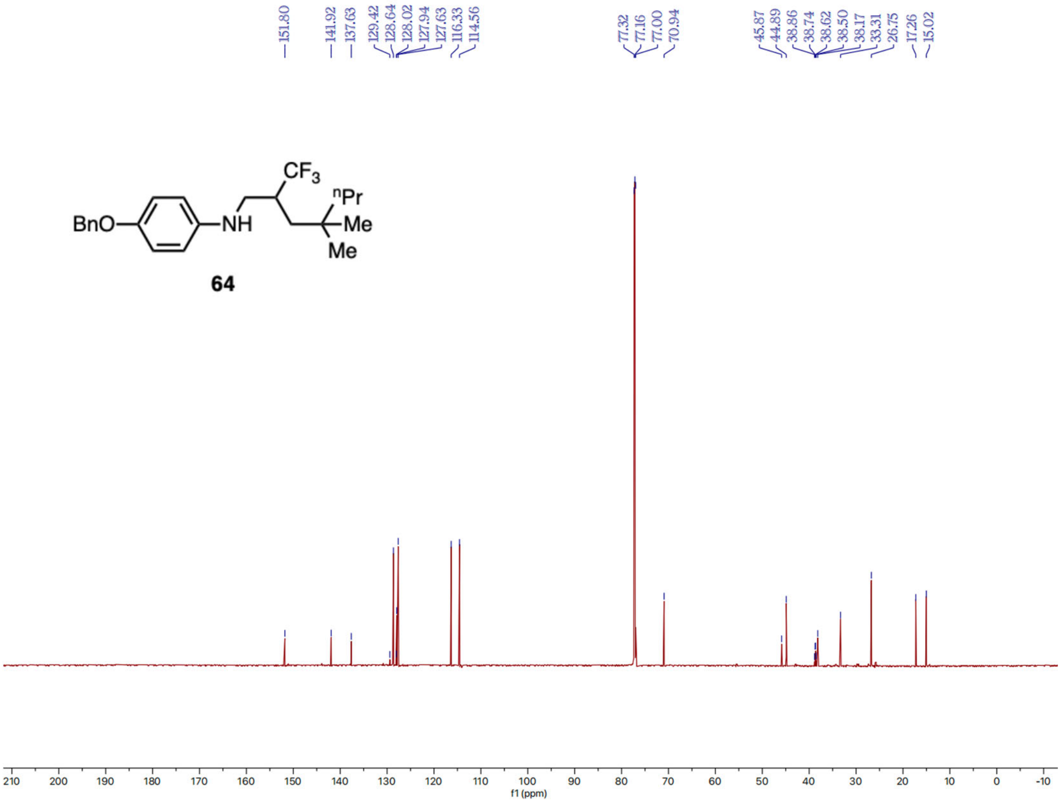


$^{13}$C NMR spectrum (201 MHz, Chloroform-*d*) of compound **64**

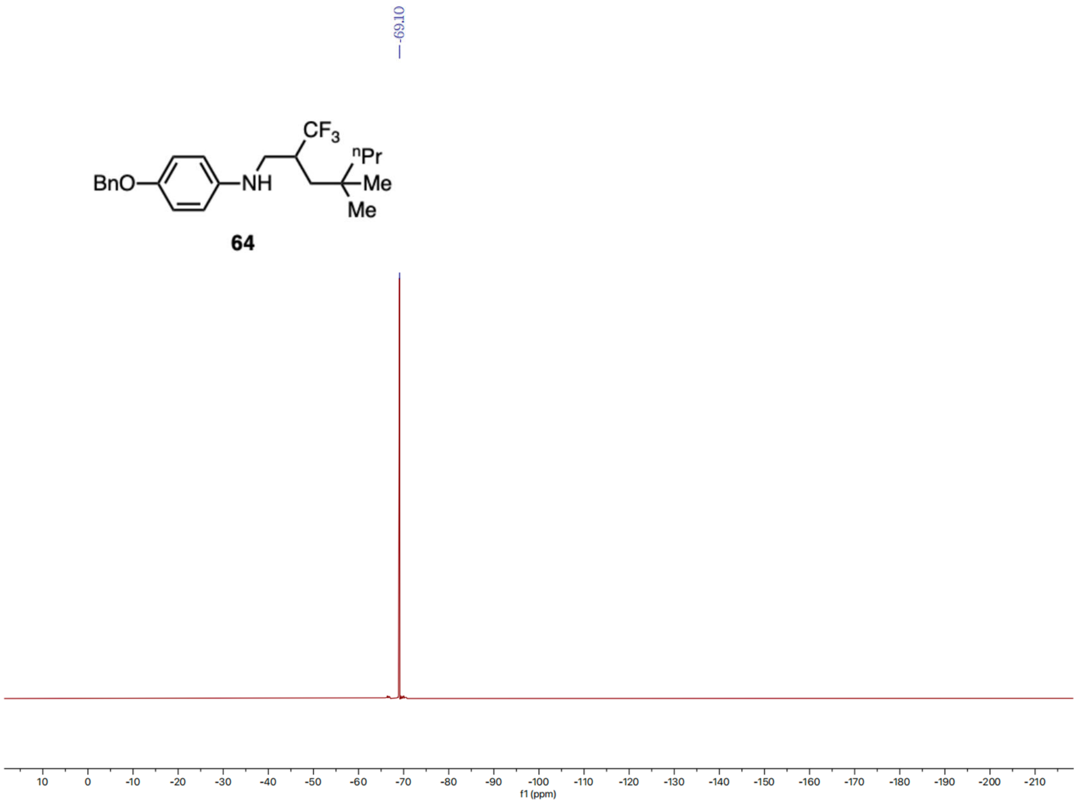


$^{19}$F NMR spectrum (376 MHz, Chloroform-*d*) of compound **64**

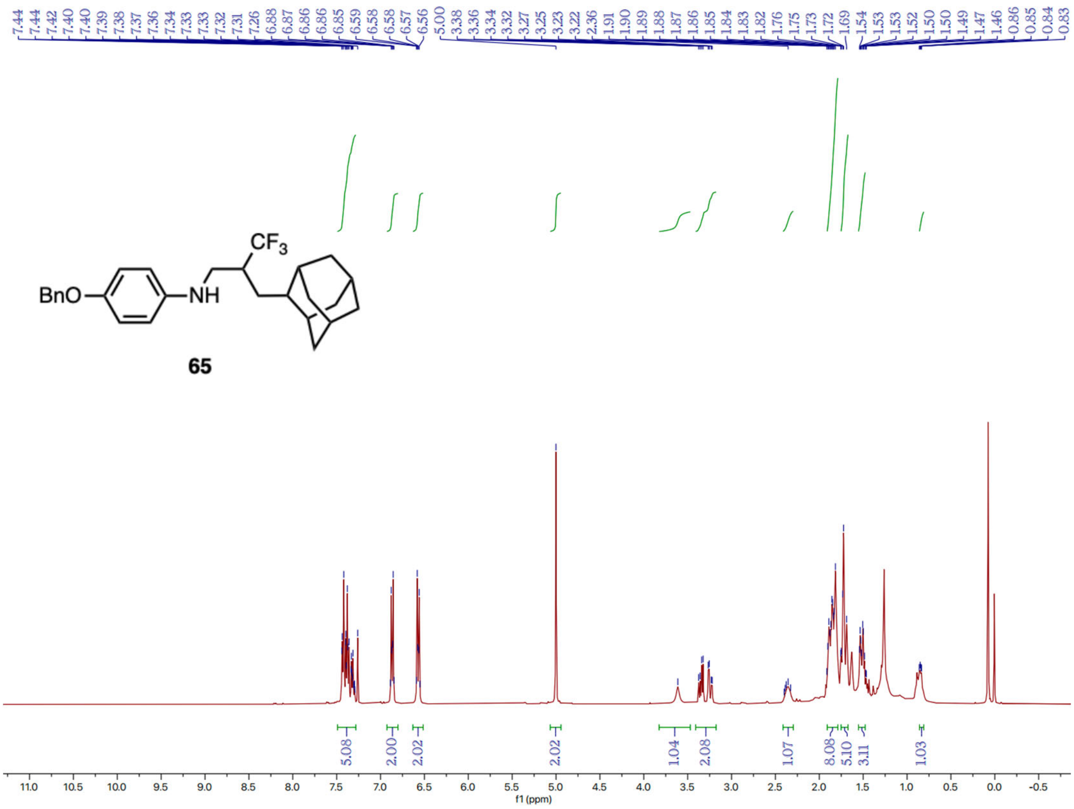


$^{1}H$ NMR spectrum (400 MHz, Chloroform-*d*) of compound **65**

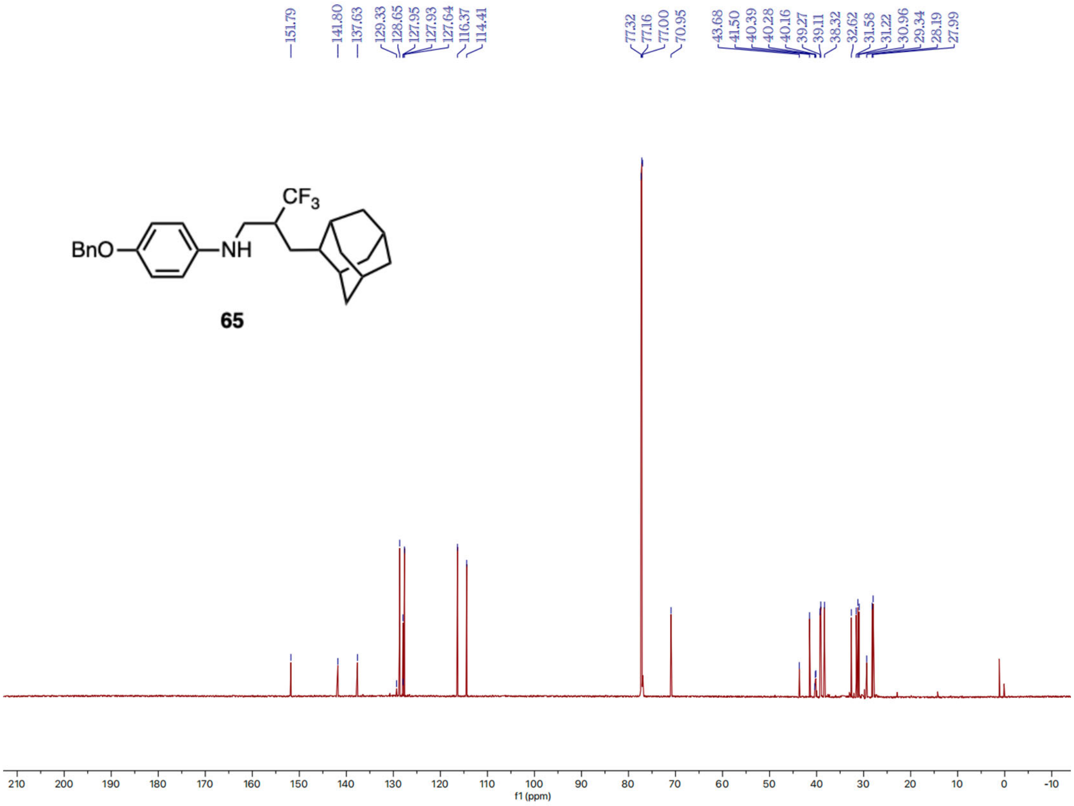


$^{13}C$ NMR spectrum (201 MHz, Chloroform-*d*) of compound **65**

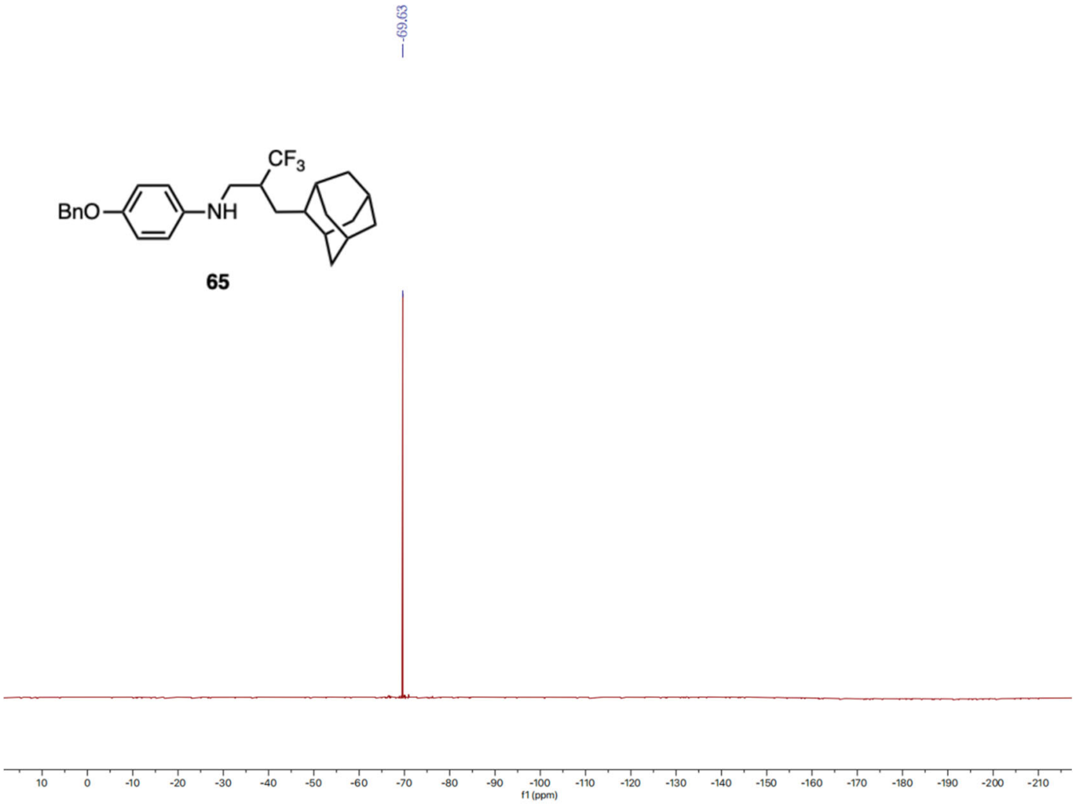


$^{19}F$ NMR spectrum (376 MHz, Chloroform-*d*) of compound **65**

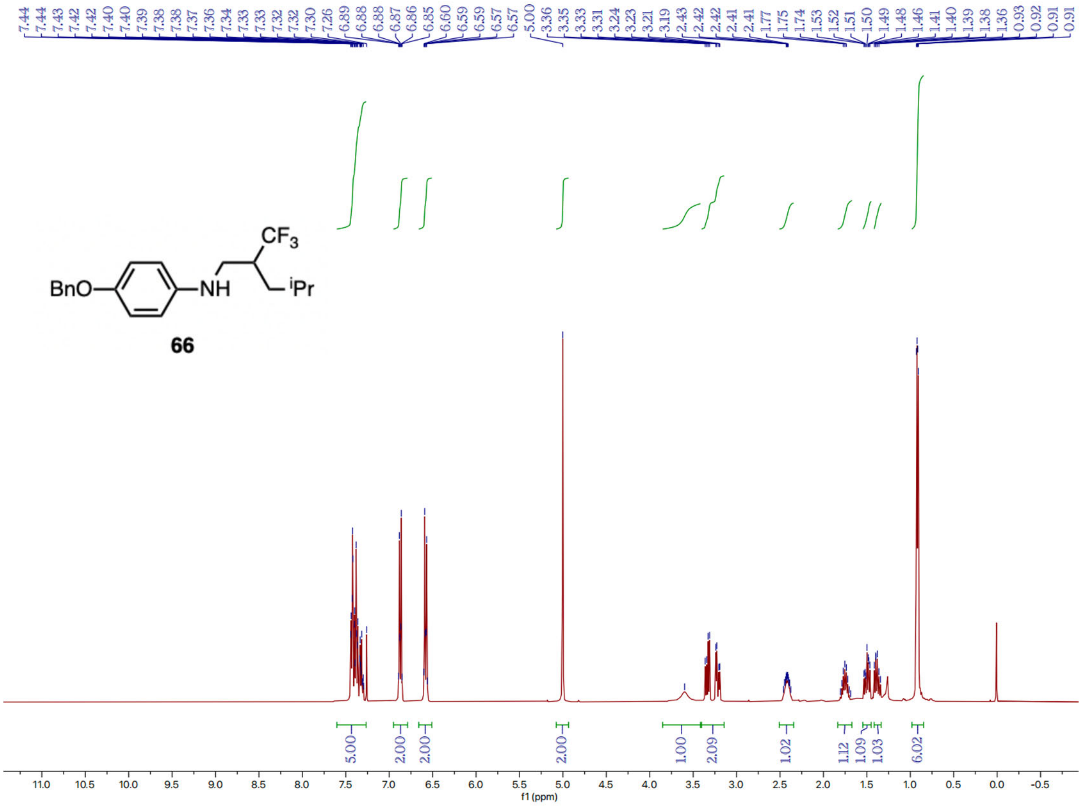

$^1H$ NMR spectrum (400 MHz, Chloroform-*d*) of compound **66**

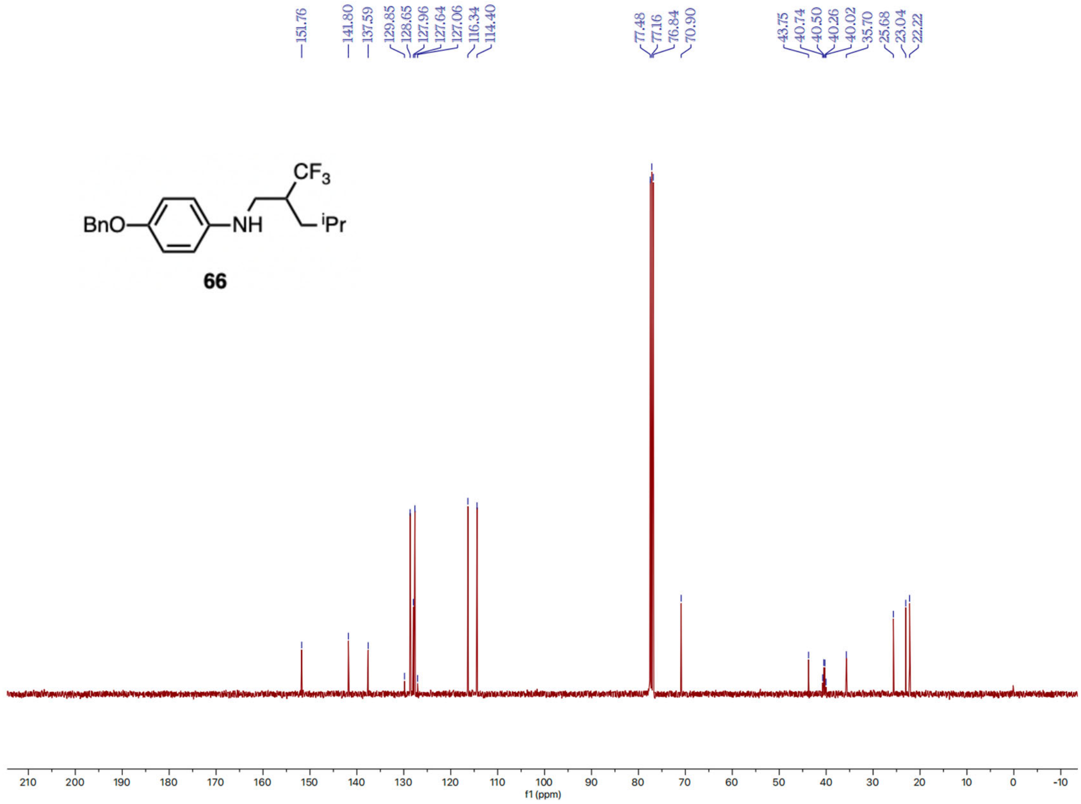

$^{13}C$ NMR spectrum (101 MHz, Chloroform-*d*) of compound **66**

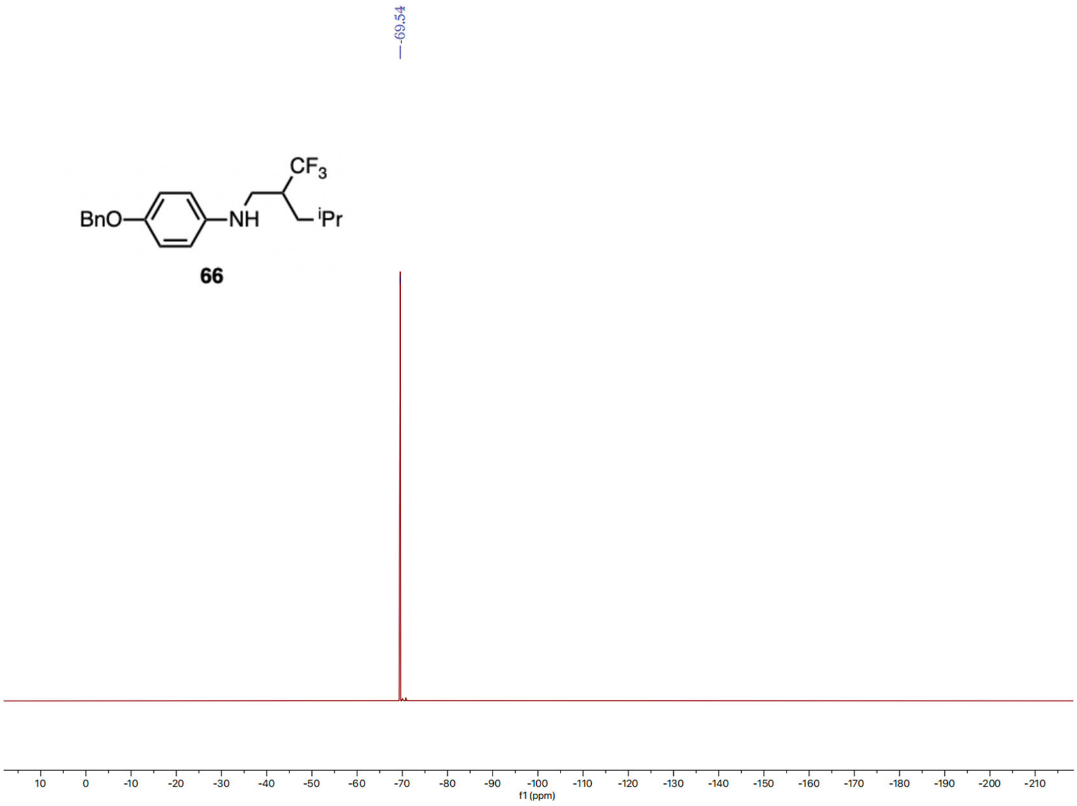


$^{19}$F NMR spectrum (376 MHz, Chloroform-*d*) of compound **66**

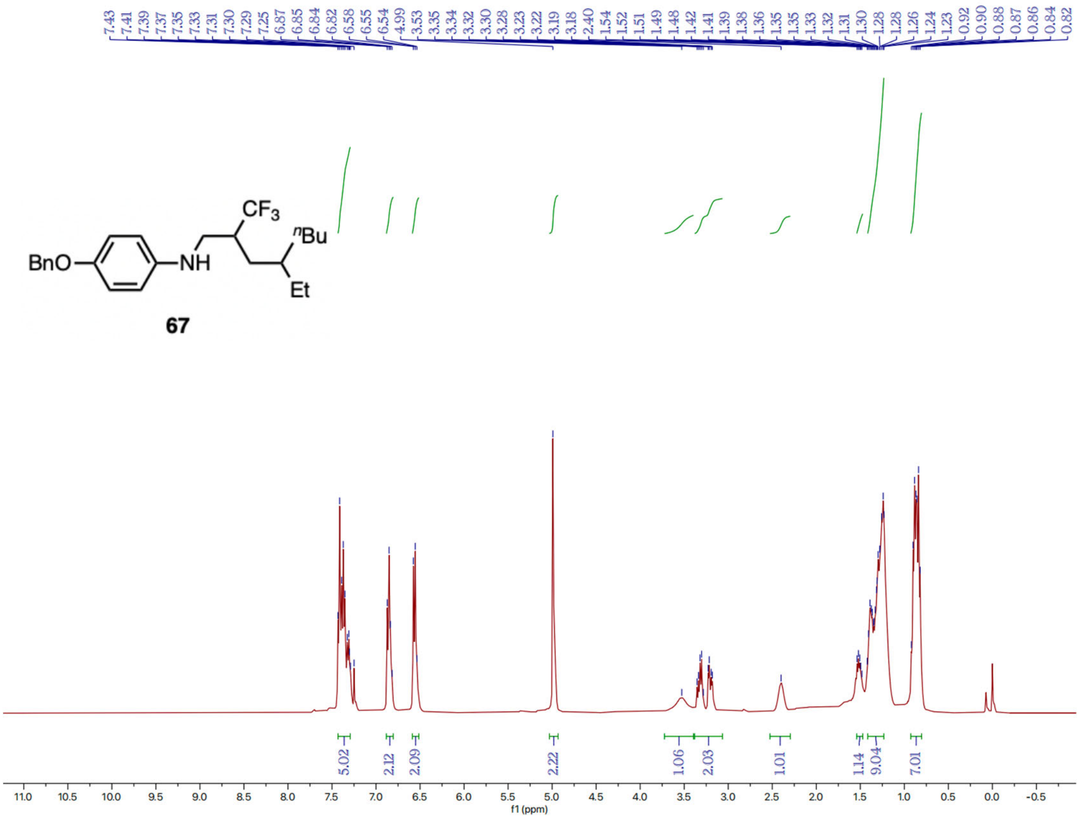

$^{1}$H NMR spectrum (400 MHz, Chloroform-*d*) of compound **67**

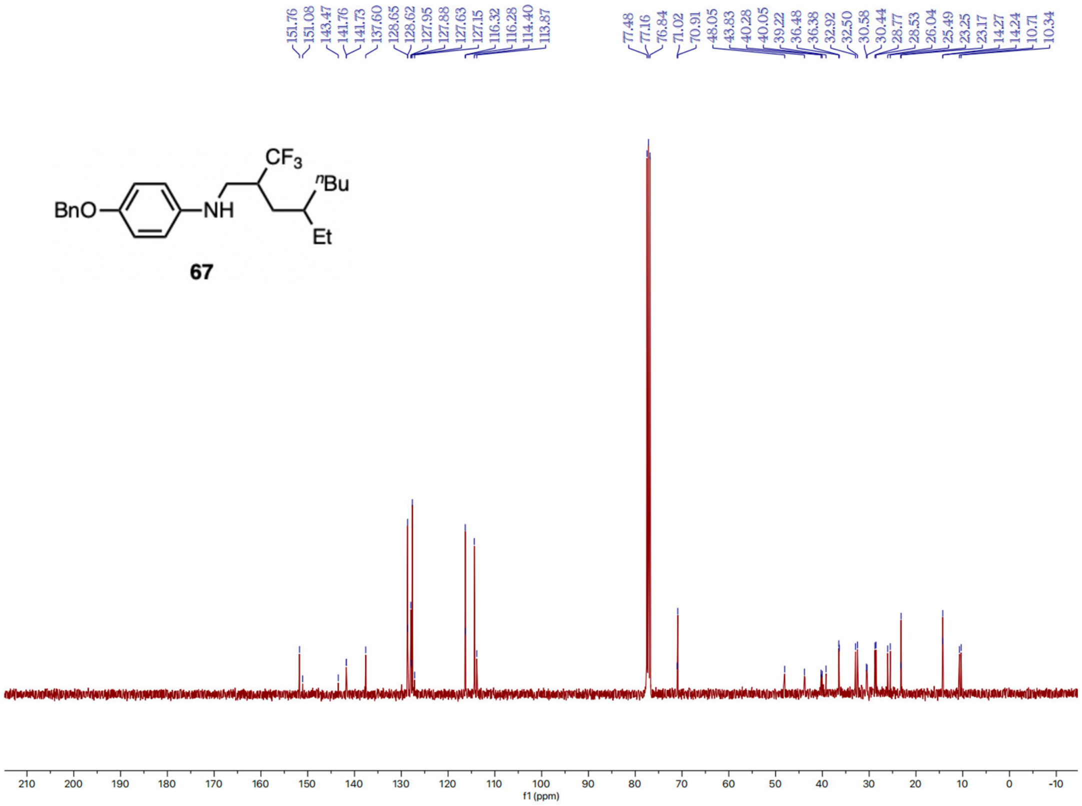

$^{13}$C NMR spectrum (101 MHz, Chloroform-*d*) of compound **67**

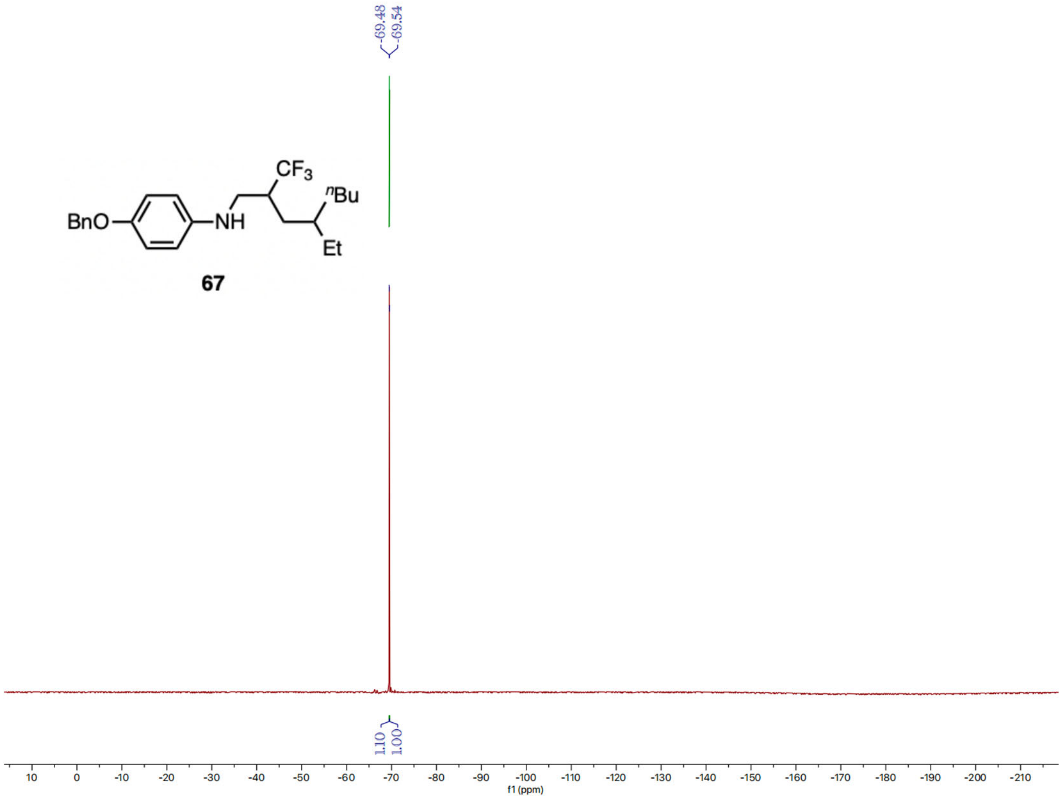


$^{19}$F NMR spectrum (376 MHz, Chloroform-*d*) of compound **67**

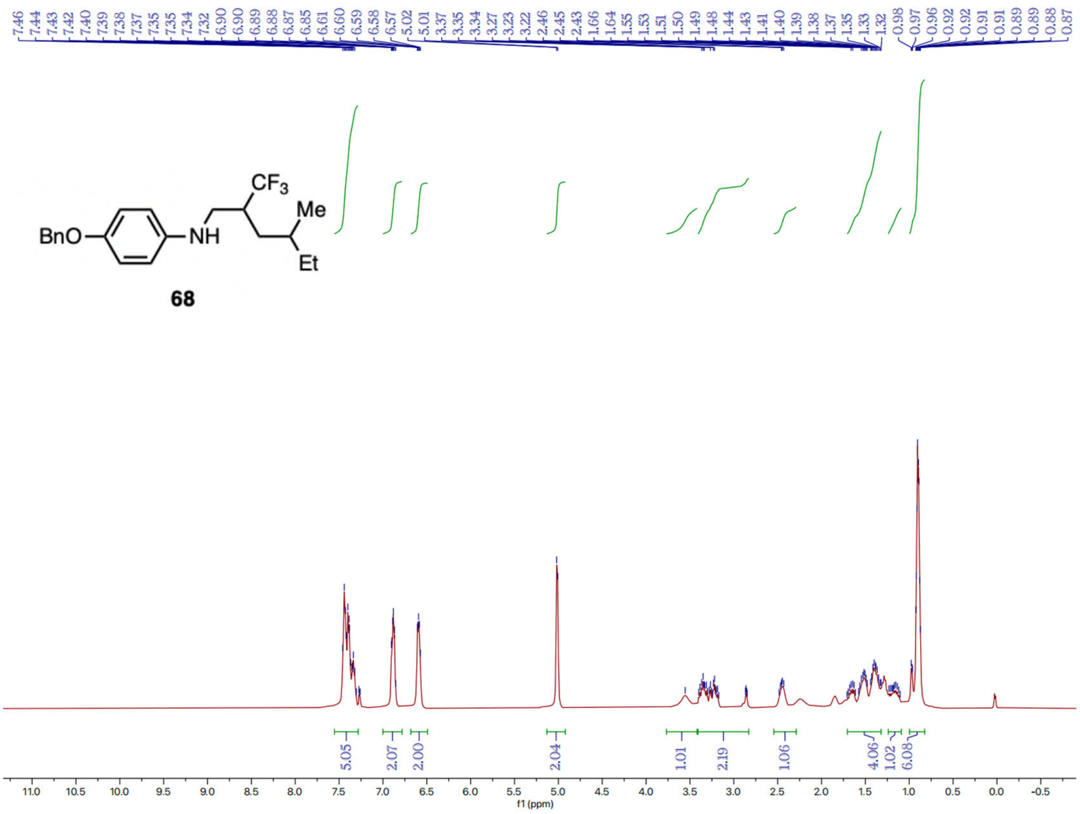

$^{1}$H NMR spectrum (400 MHz, Chloroform-*d*) of compound **68**

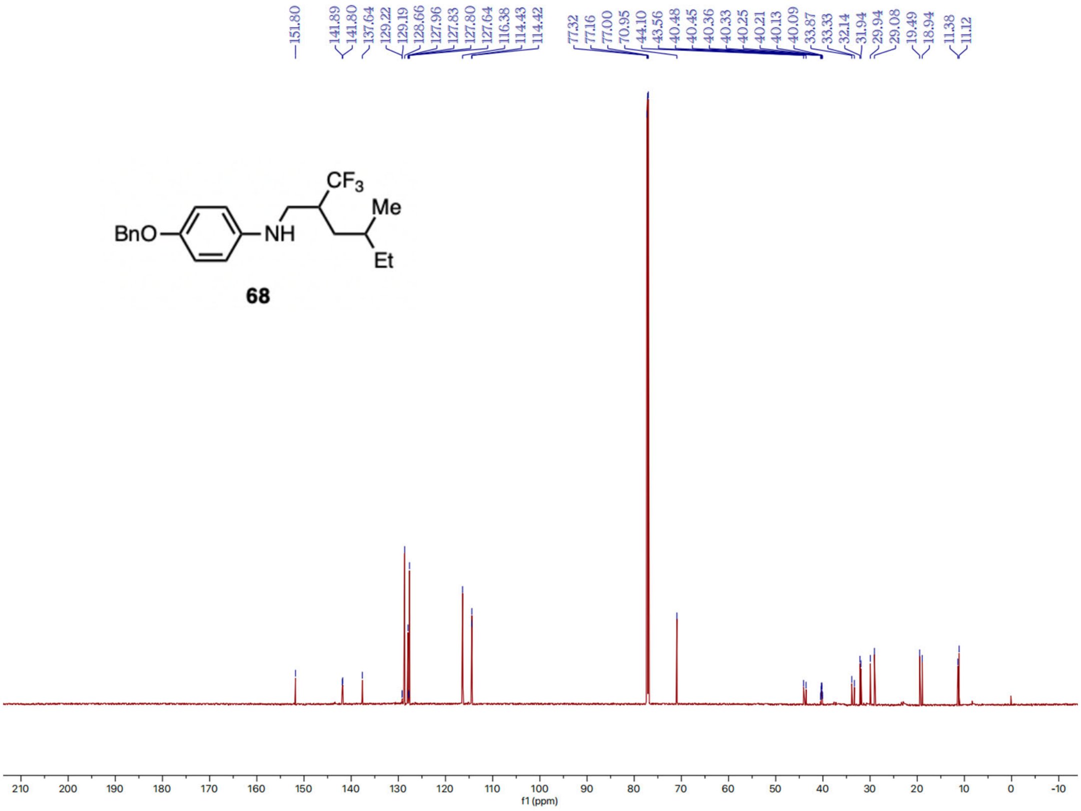

$^{13}$C NMR spectrum (201 MHz, Chloroform-*d*) of compound **68**

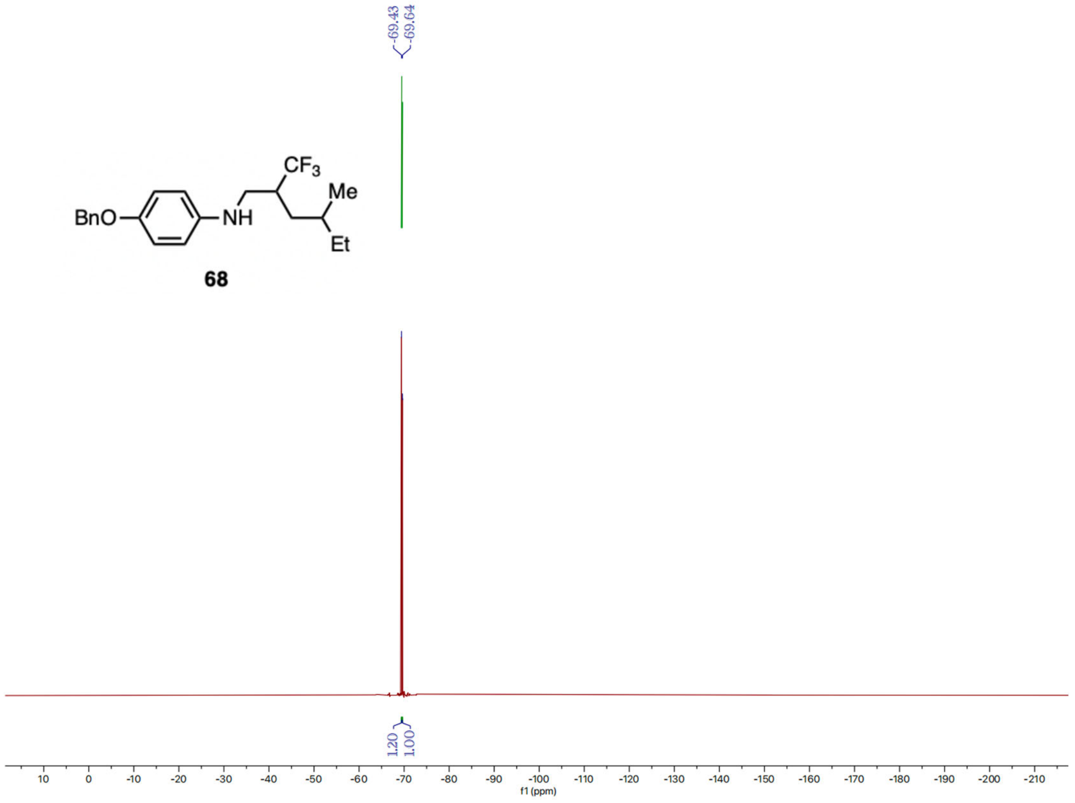



$^{19}F$ NMR spectrum (376 MHz, Chloroform-*d*) of compound **68**

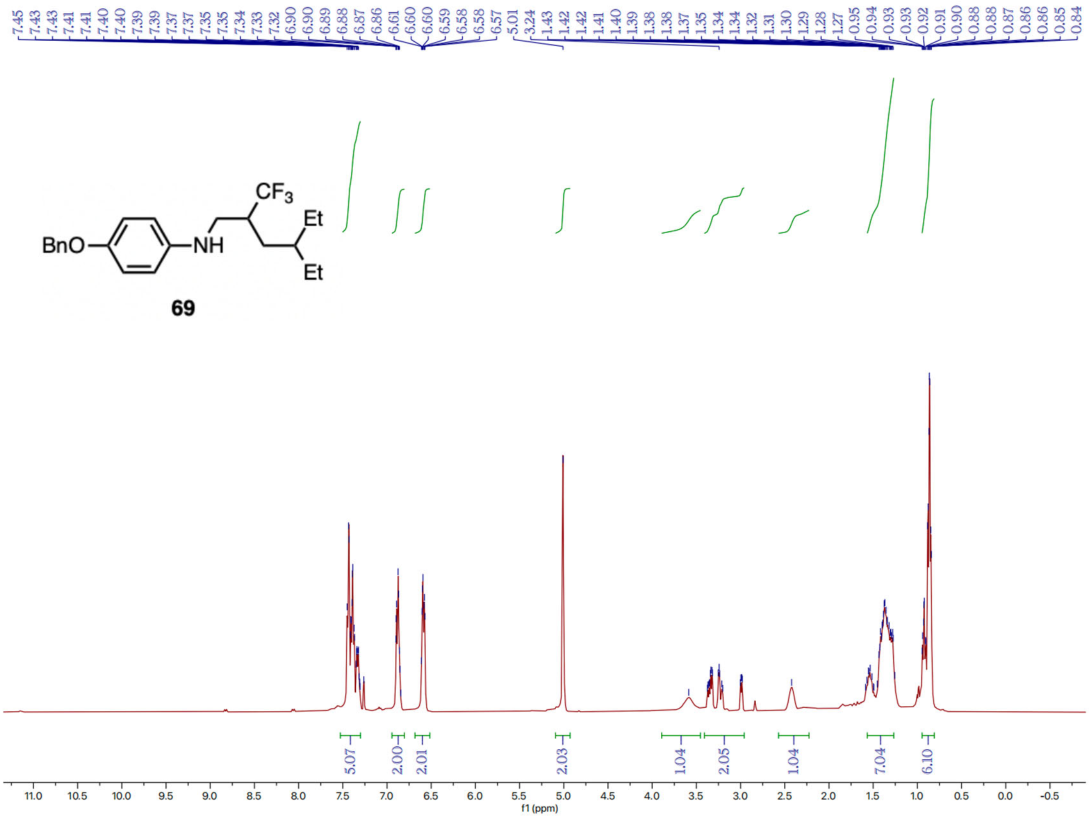


$^{1}$H NMR spectrum (400 MHz, Chloroform-*d*) of compound **69**

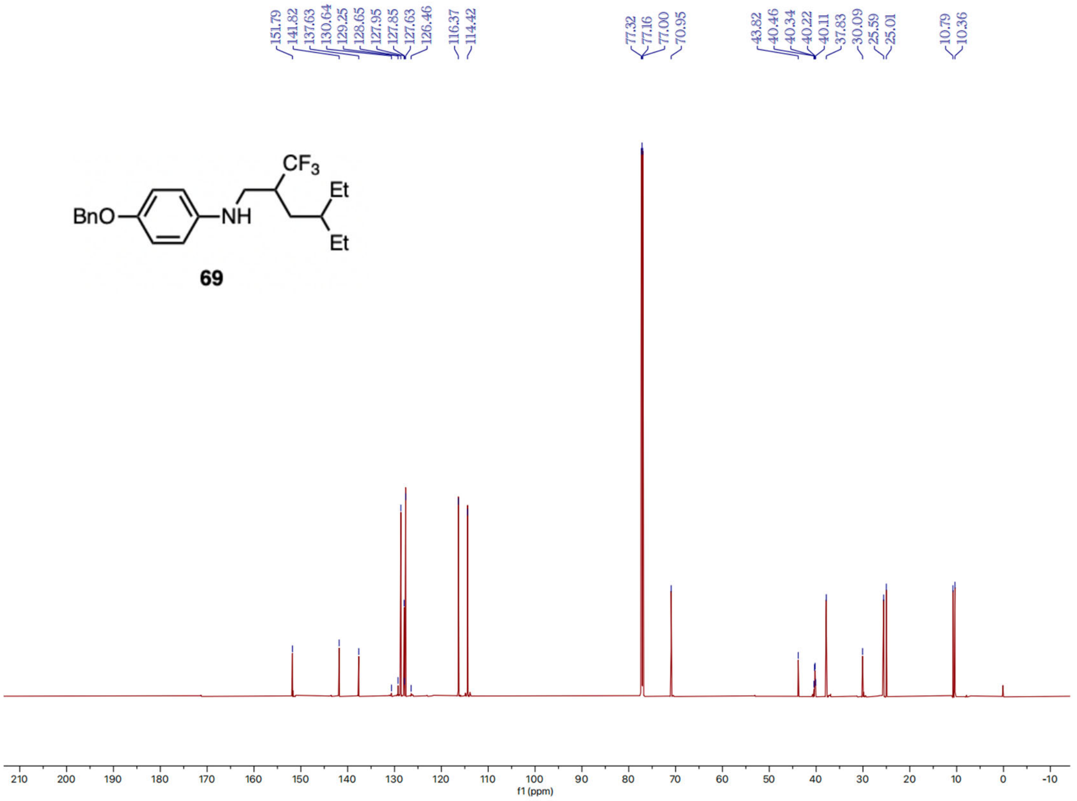


$^{13}$C NMR spectrum (201 MHz, Chloroform-*d*) of compound **69**

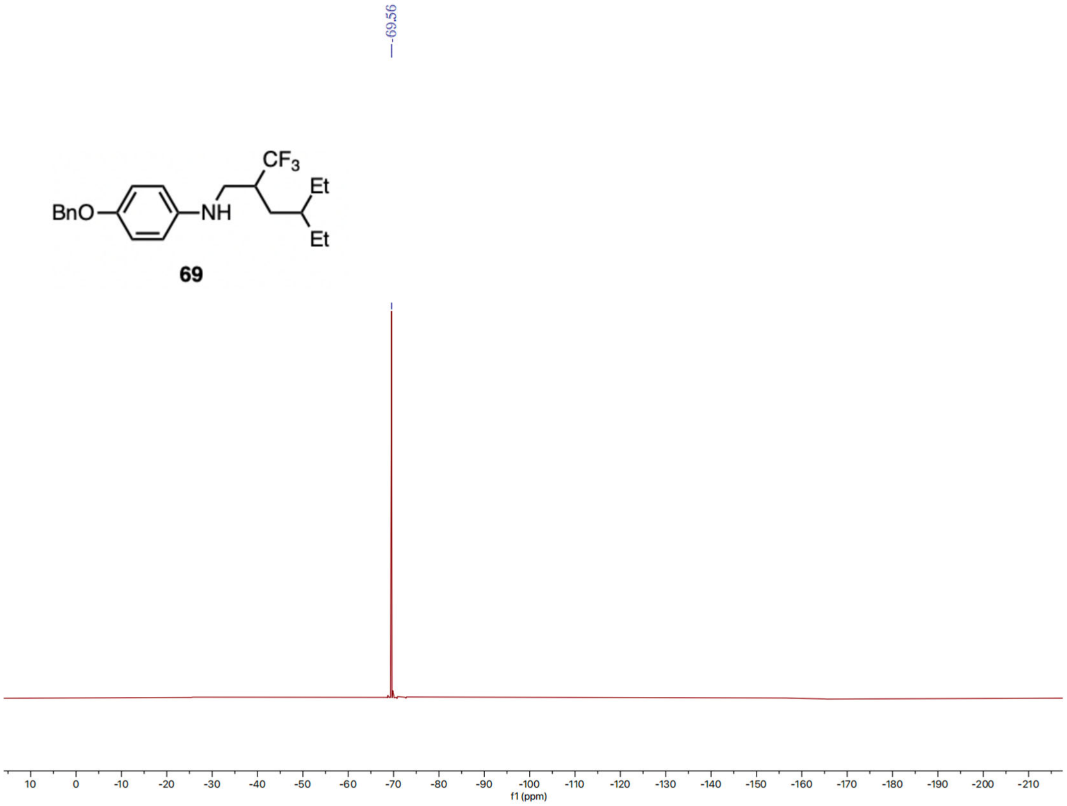


$^{19}$F NMR spectrum (376 MHz, Chloroform-*d*) of compound **69**

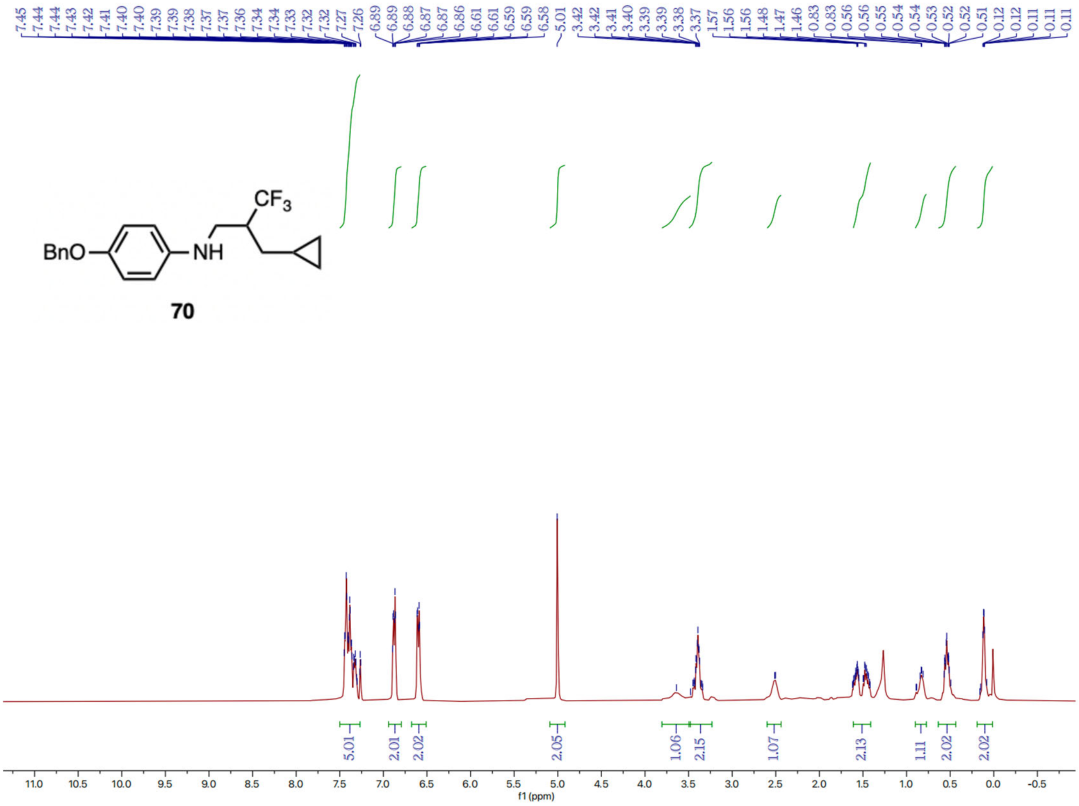


$^{1}$H NMR spectrum (400 MHz, Chloroform-*d*) of compound **70**

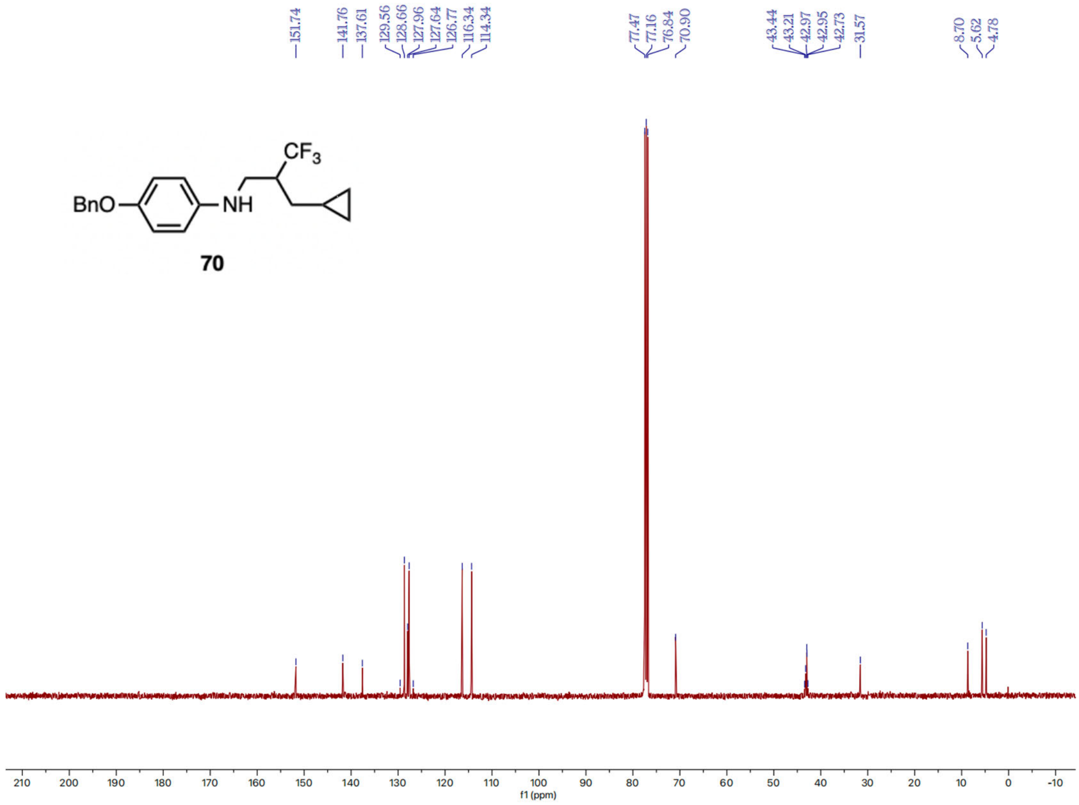


$^{13}$C NMR spectrum (101 MHz, Chloroform-*d*) of compound **70**

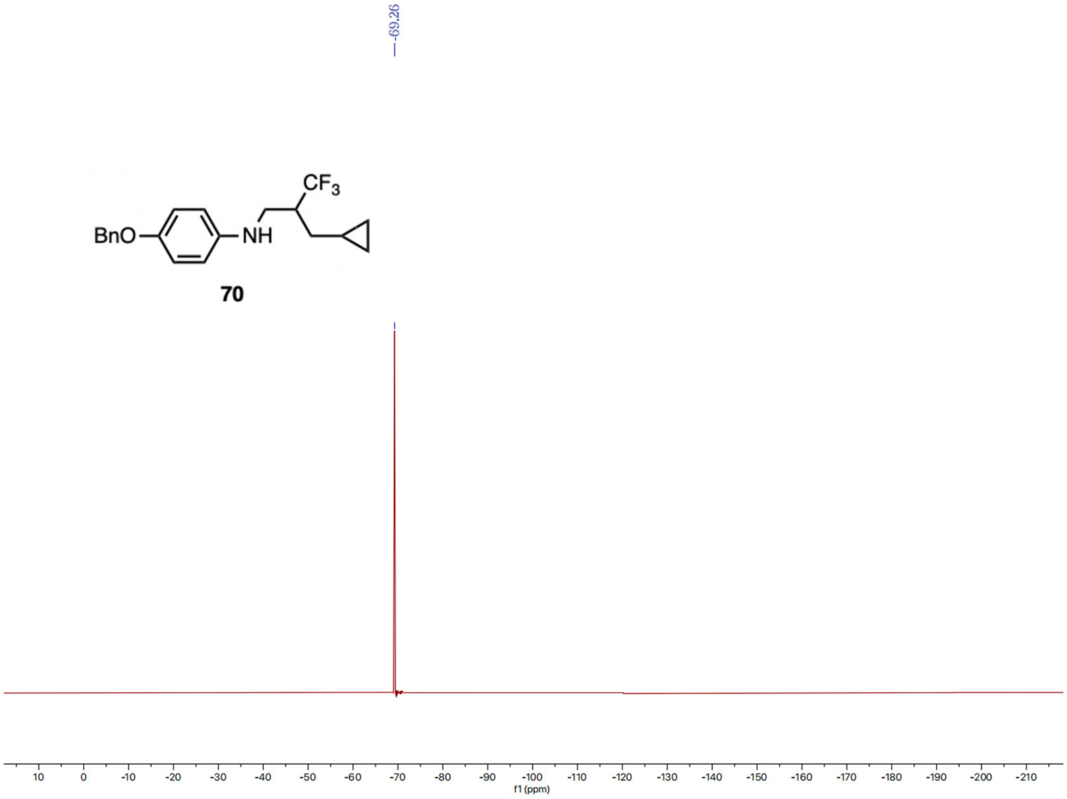


$^{19}$F NMR spectrum (376 MHz, Chloroform-*d*) of compound **70**

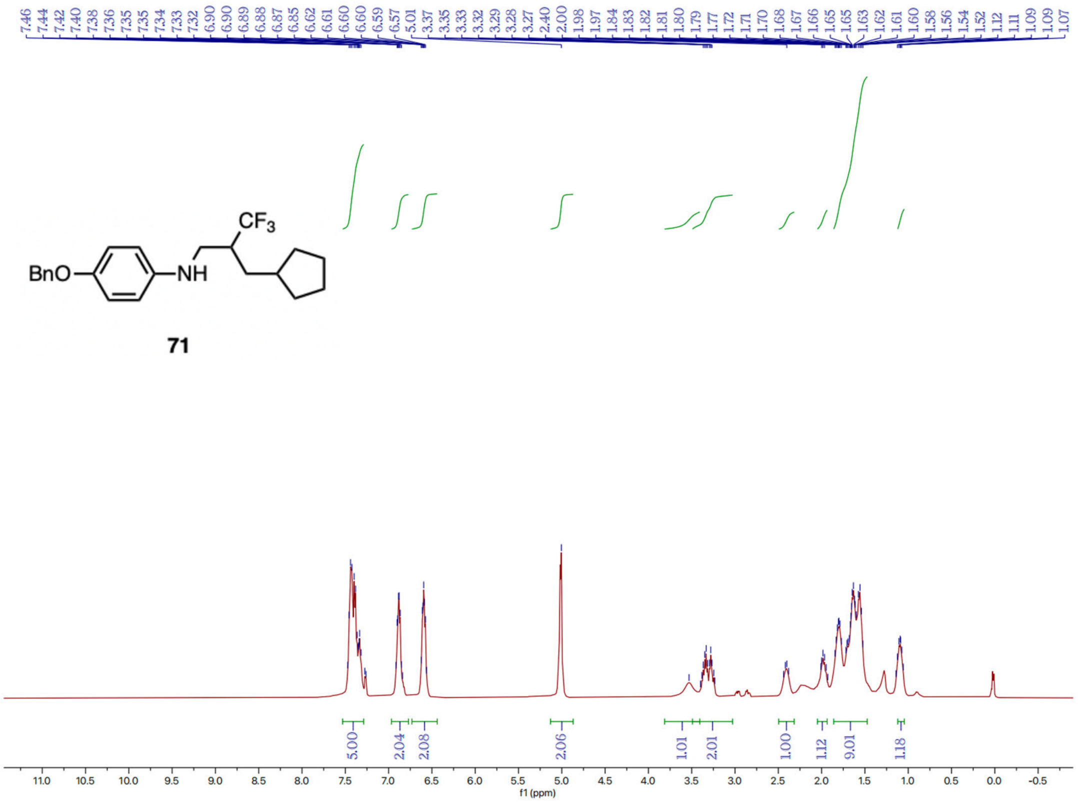

$^{1}$H NMR spectrum (400 MHz, Chloroform-*d*) of compound **71**

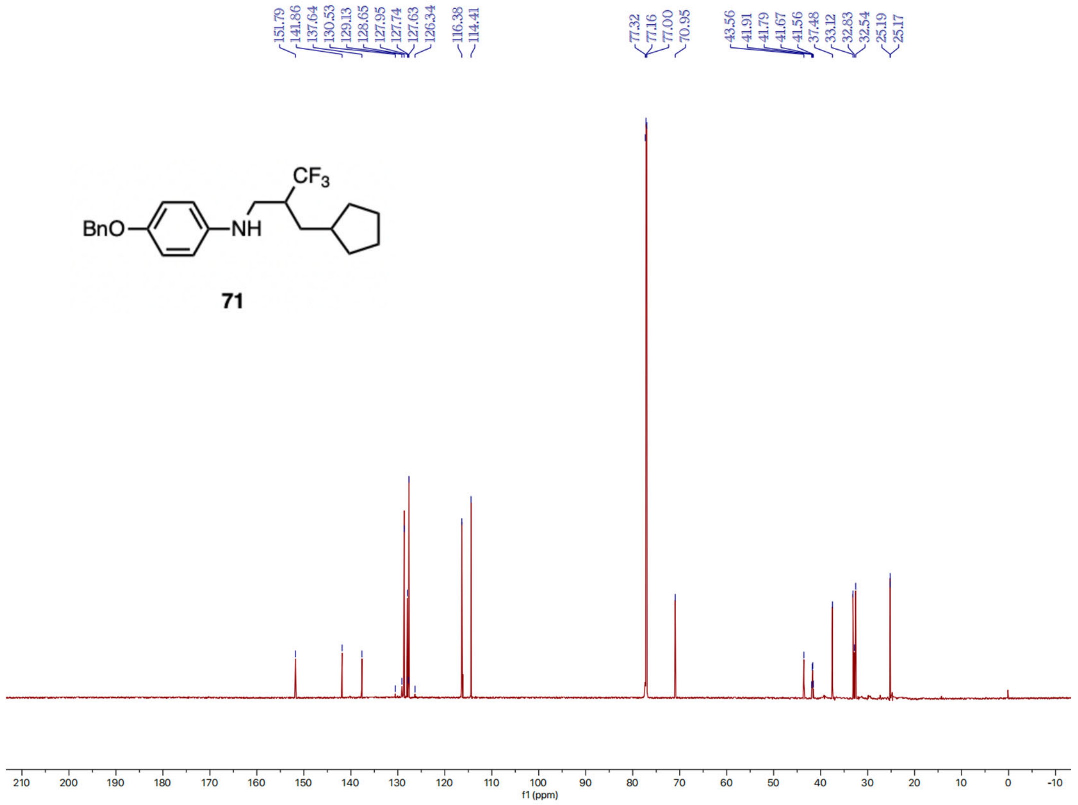

$^{13}$C NMR spectrum (201 MHz, Chloroform-*d*) of compound **71**

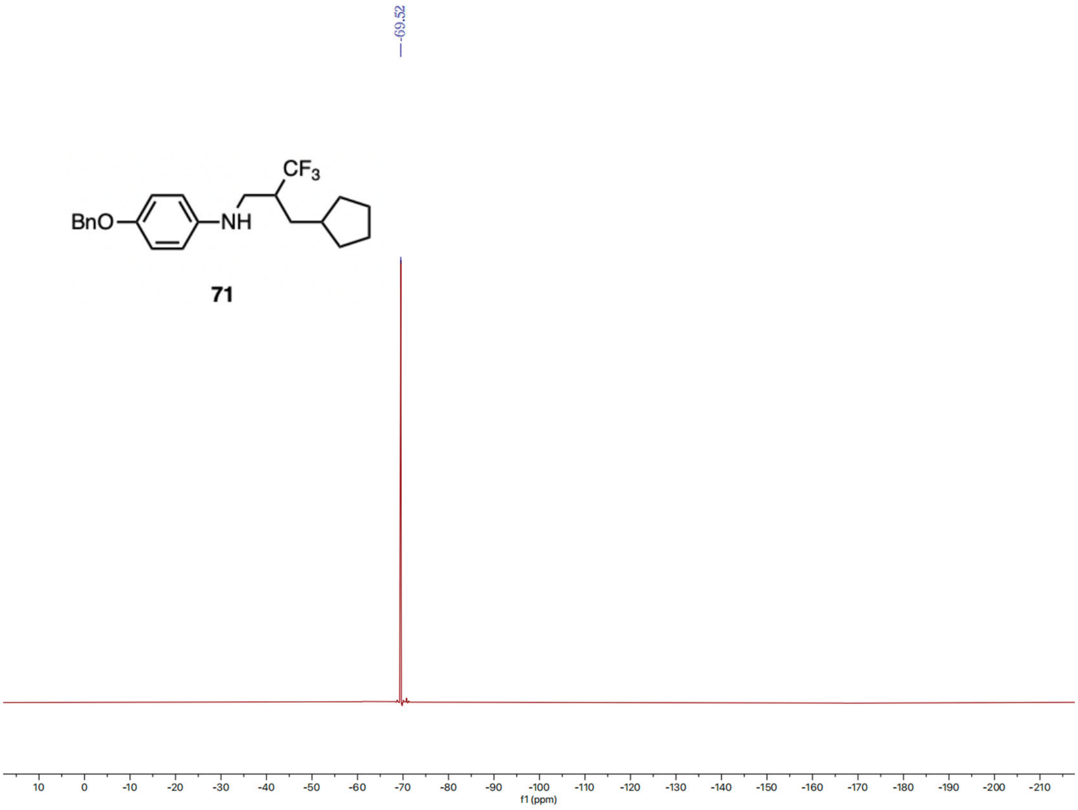


$^{19}F$ NMR spectrum (376 MHz, Chloroform-*d*) of compound **71**

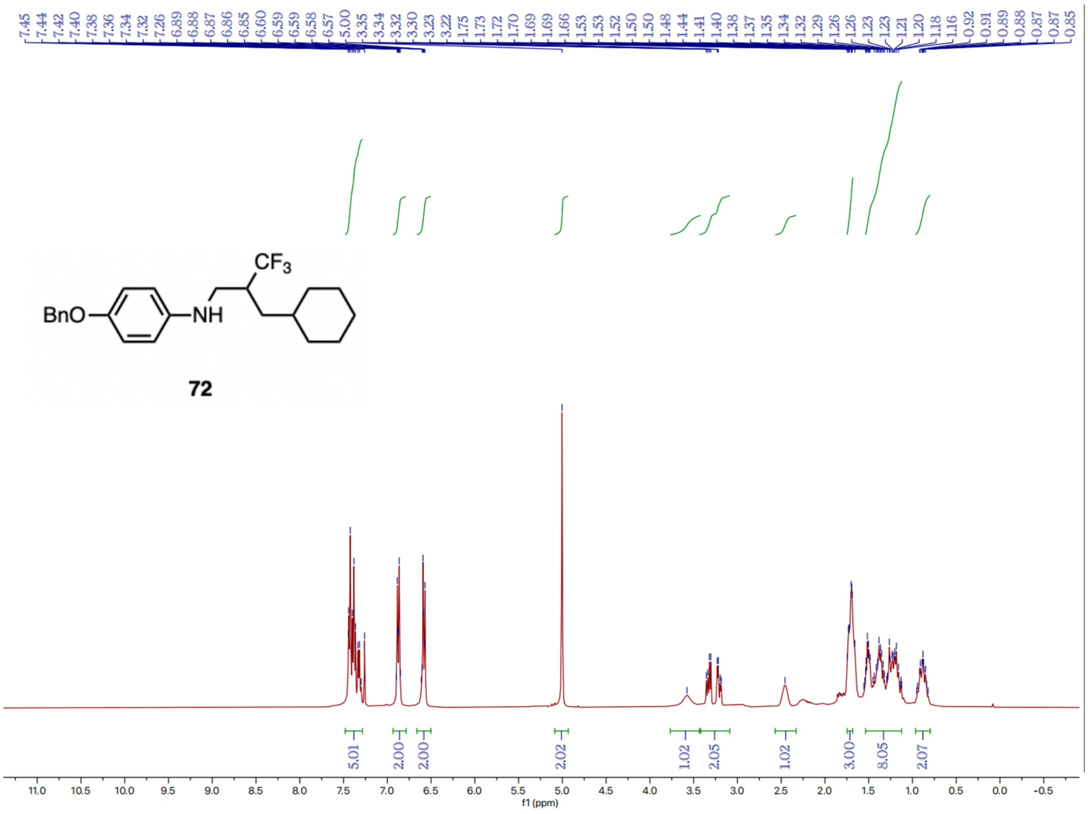

$^{1}$H NMR spectrum (400 MHz, Chloroform-*d*) of compound **72**

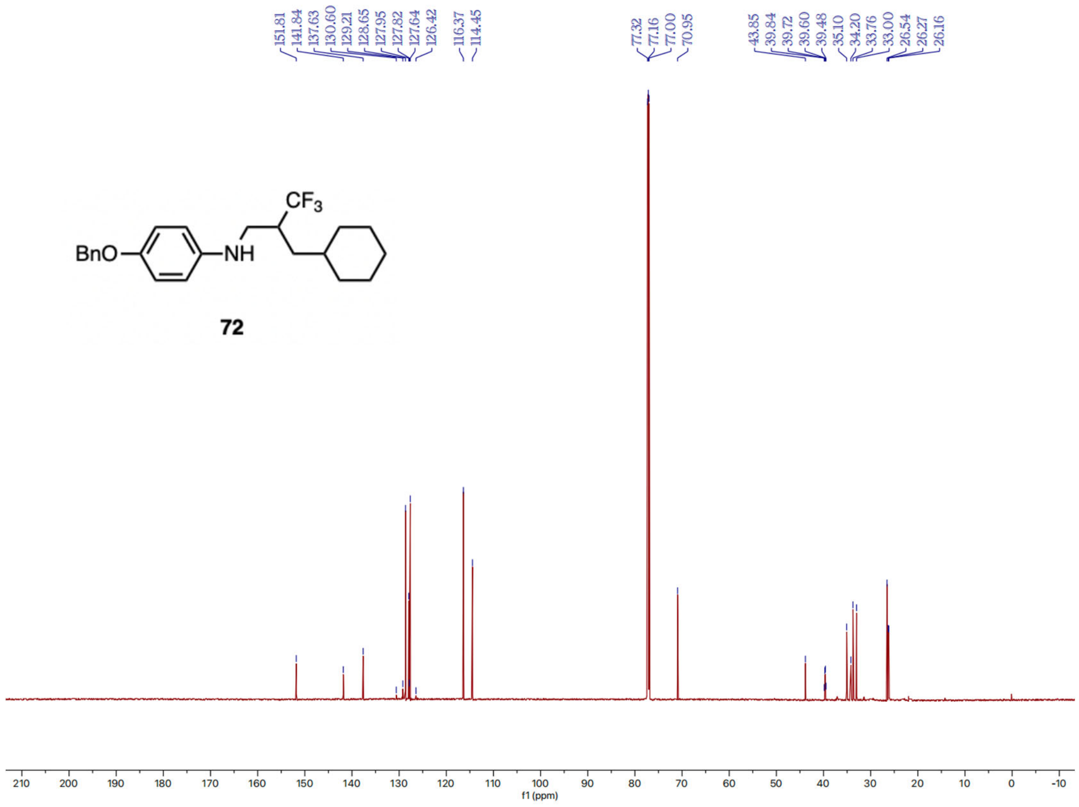

$^{13}$C NMR spectrum (201 MHz, Chloroform-*d*) of compound **72**

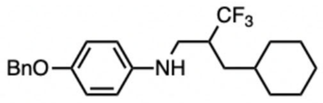


**72**

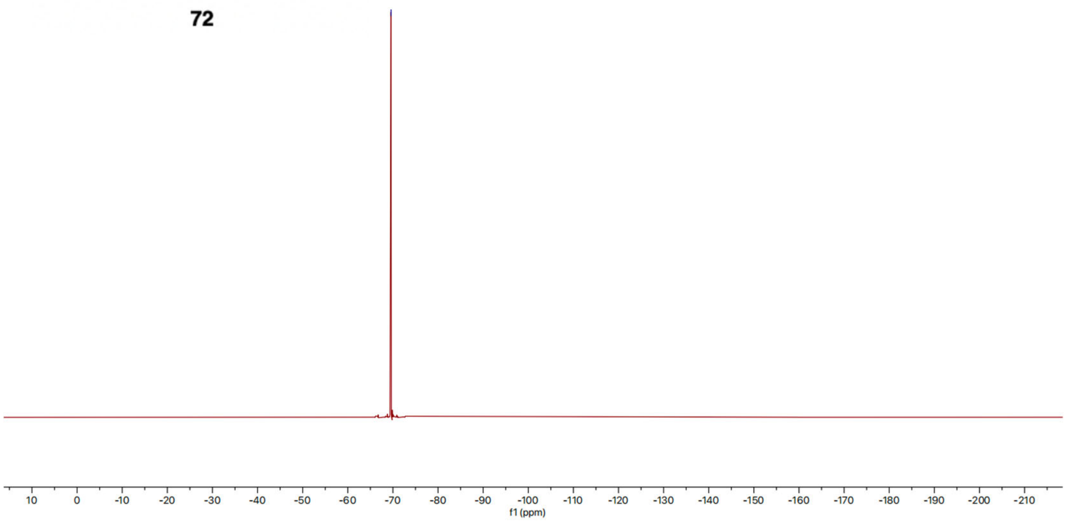


$^{19}F$ NMR spectrum (376 MHz, Chloroform-*d*) of compound **72**

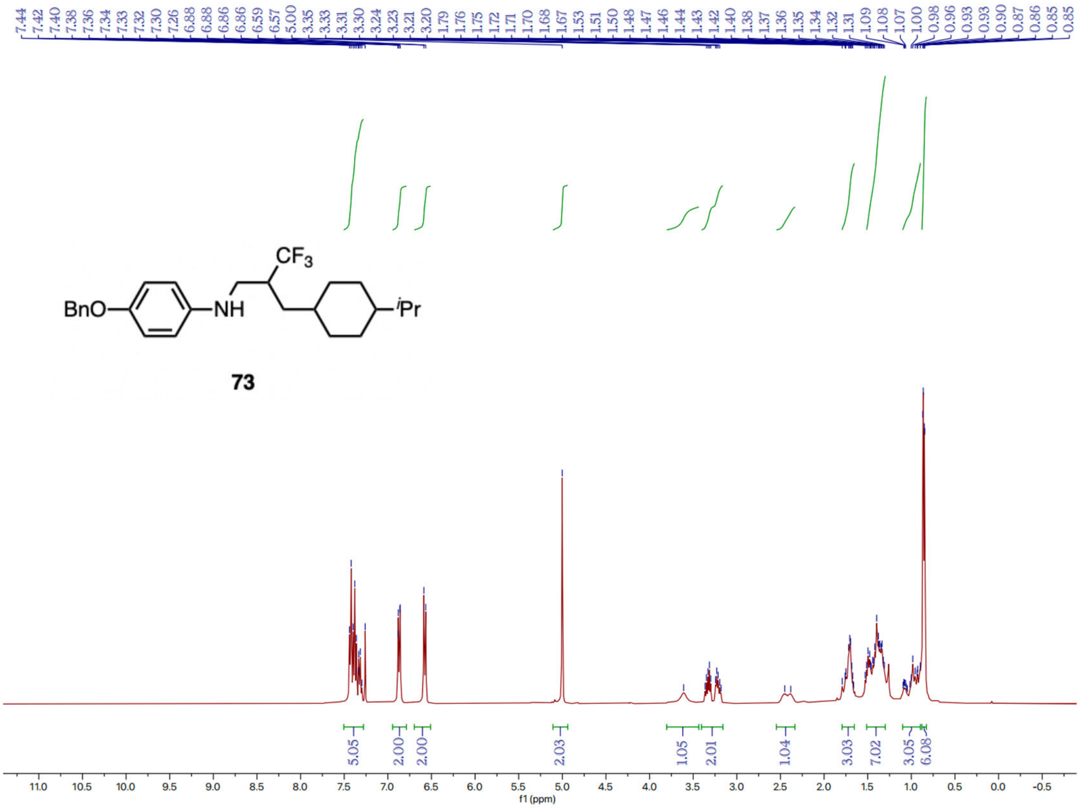

$^1H$ NMR spectrum (400 MHz, Chloroform-*d*) of compound **73**

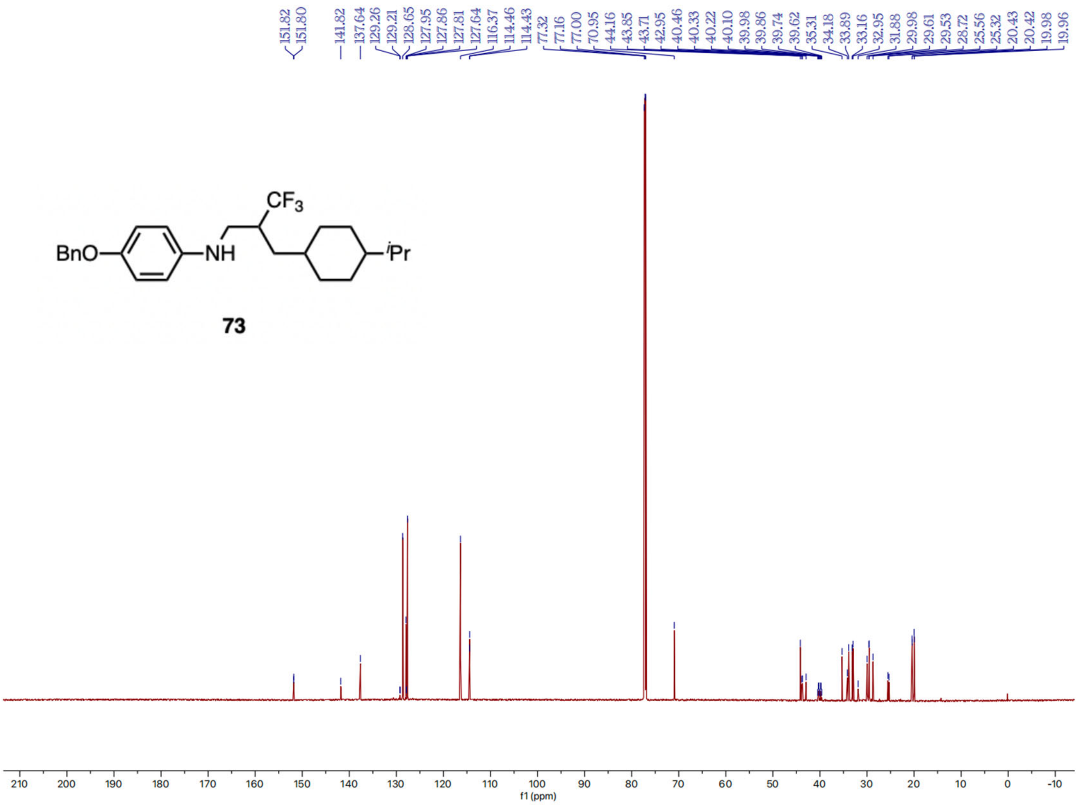

$^{13}C$ NMR spectrum (201 MHz, Chloroform-*d*) of compound **73**

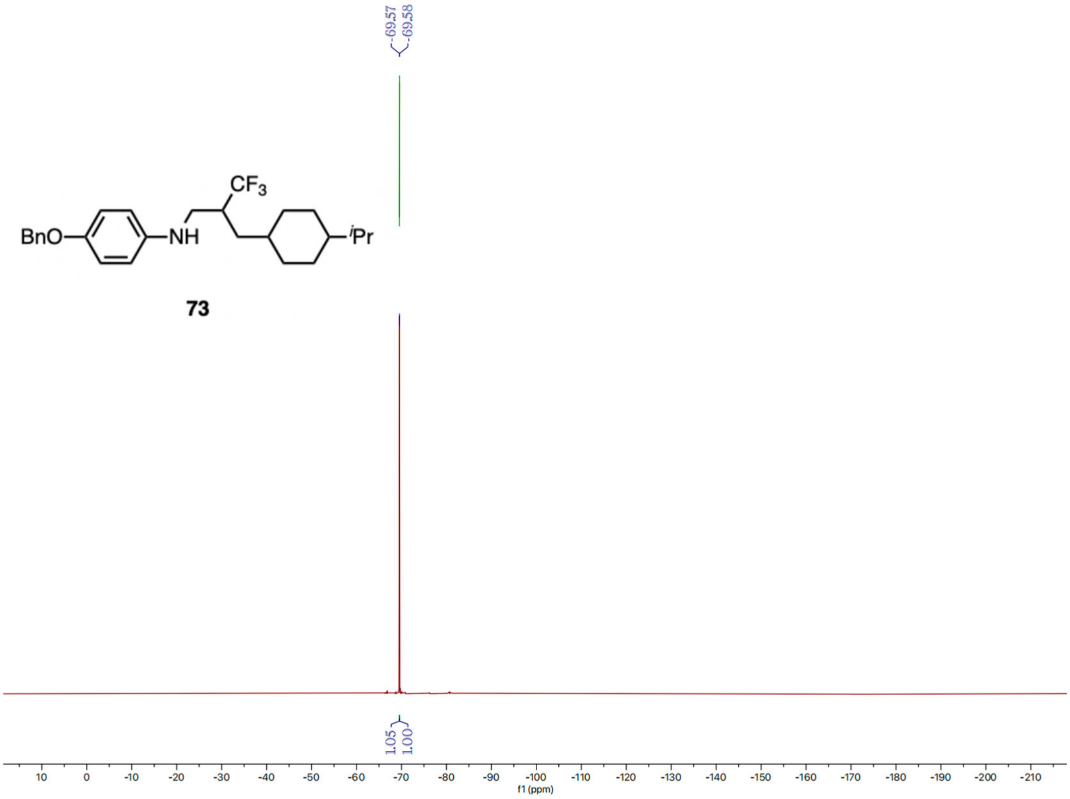


**73**

$^{19}$F NMR spectrum (376 MHz, Chloroform-*d*) of compound **73**

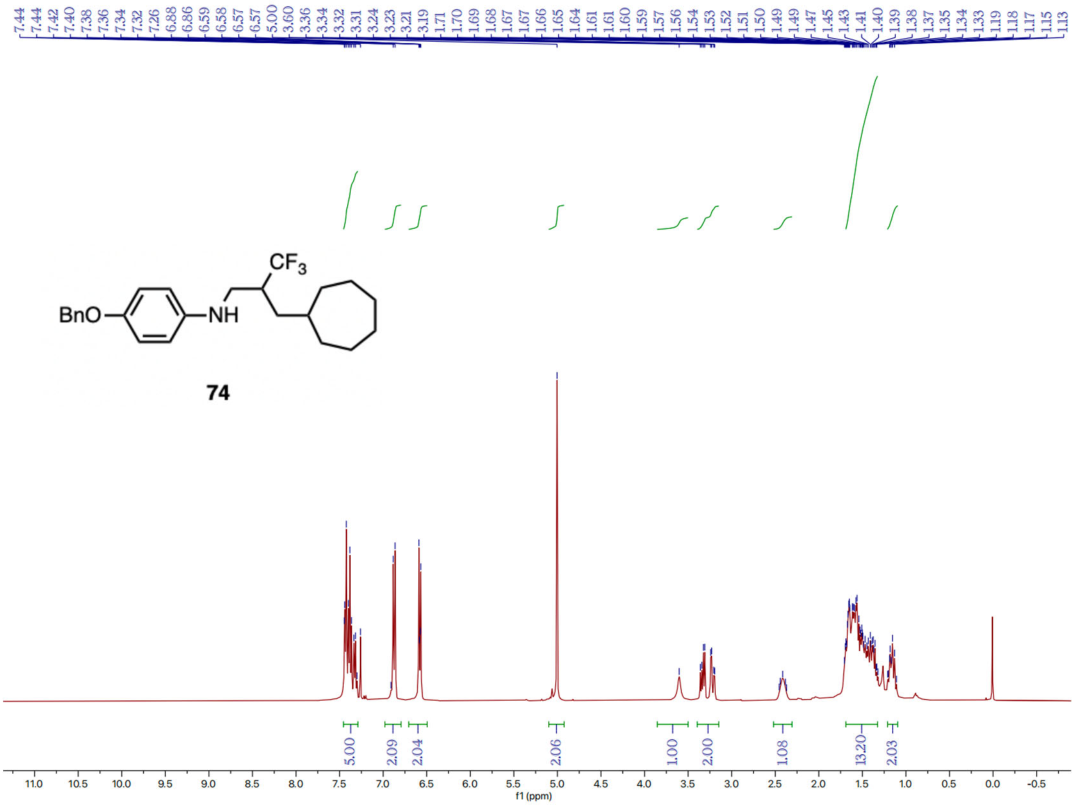


$^{1}$H NMR spectrum (400 MHz, Chloroform-*d*) of compound **74**

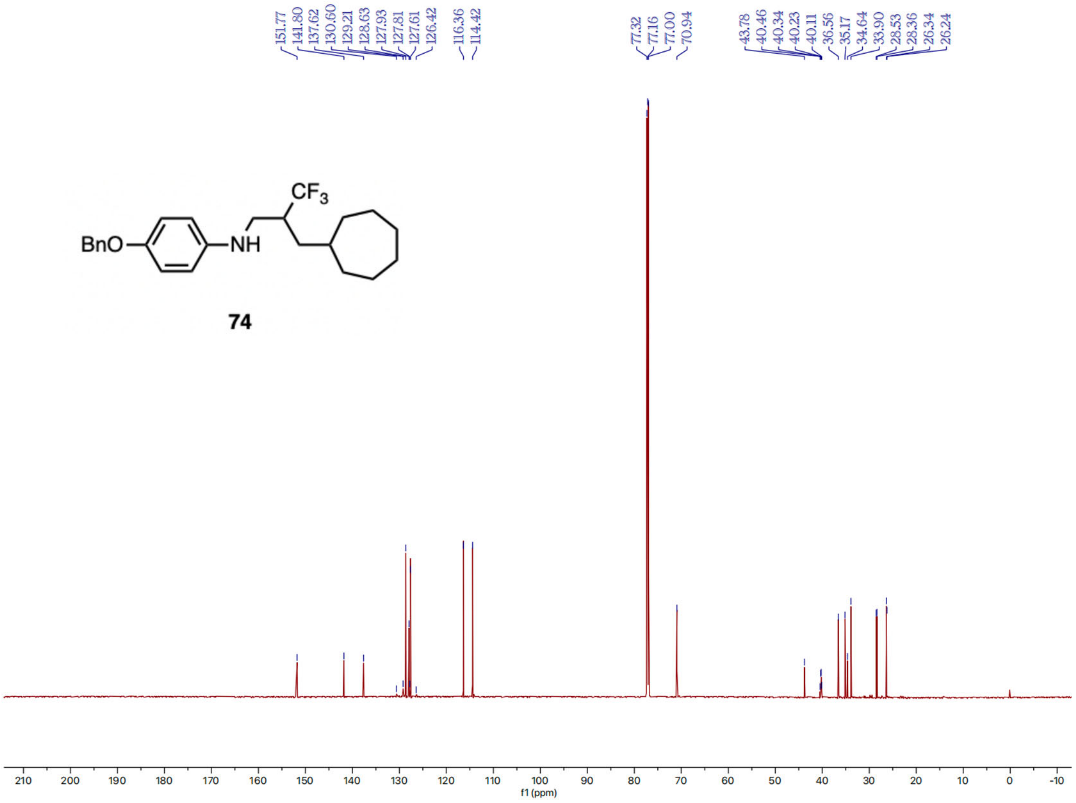


$^{13}$C NMR spectrum (201 MHz, Chloroform-*d*) of compound **74**

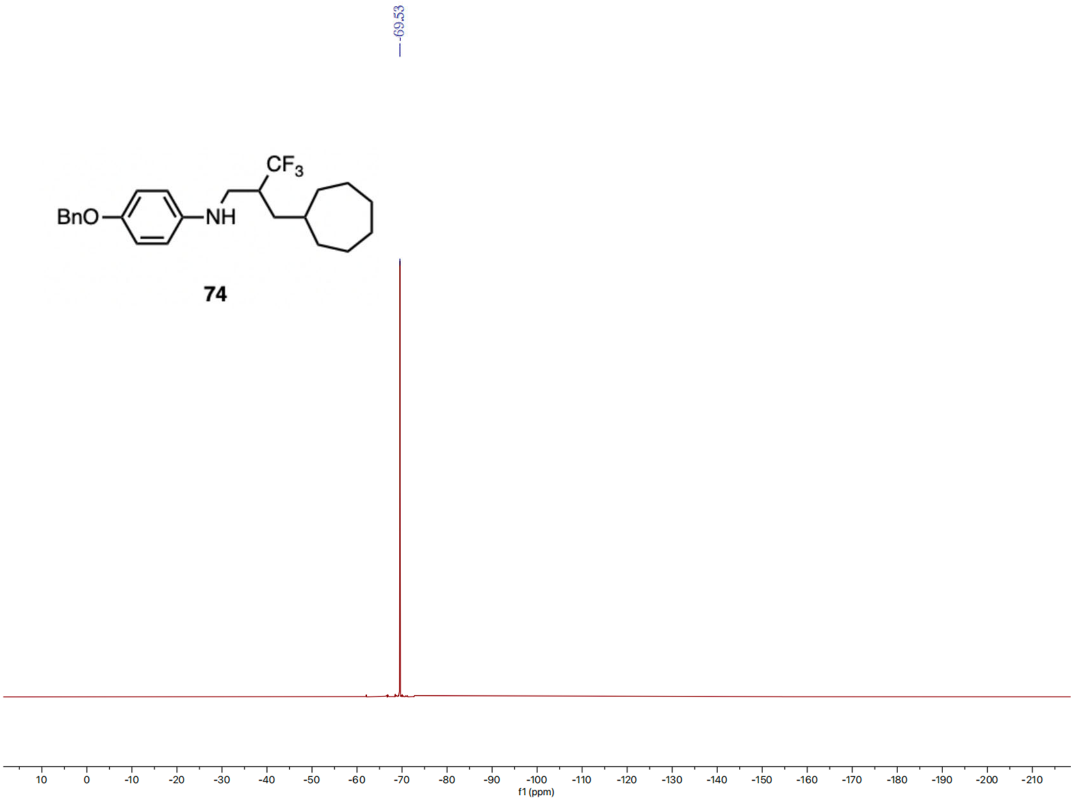


$^{19}$F NMR spectrum (376 MHz, Chloroform-*d*) of compound **74**

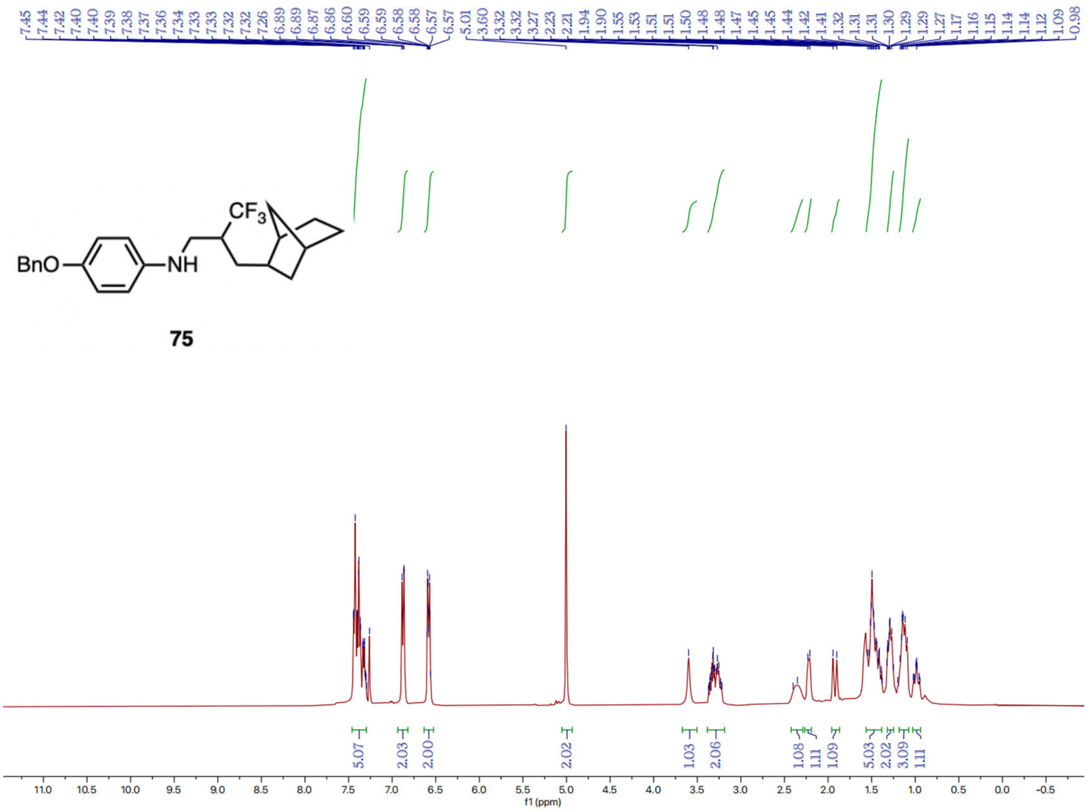


$^{1}$H NMR spectrum (400 MHz, Chloroform-*d*) of compound **75**

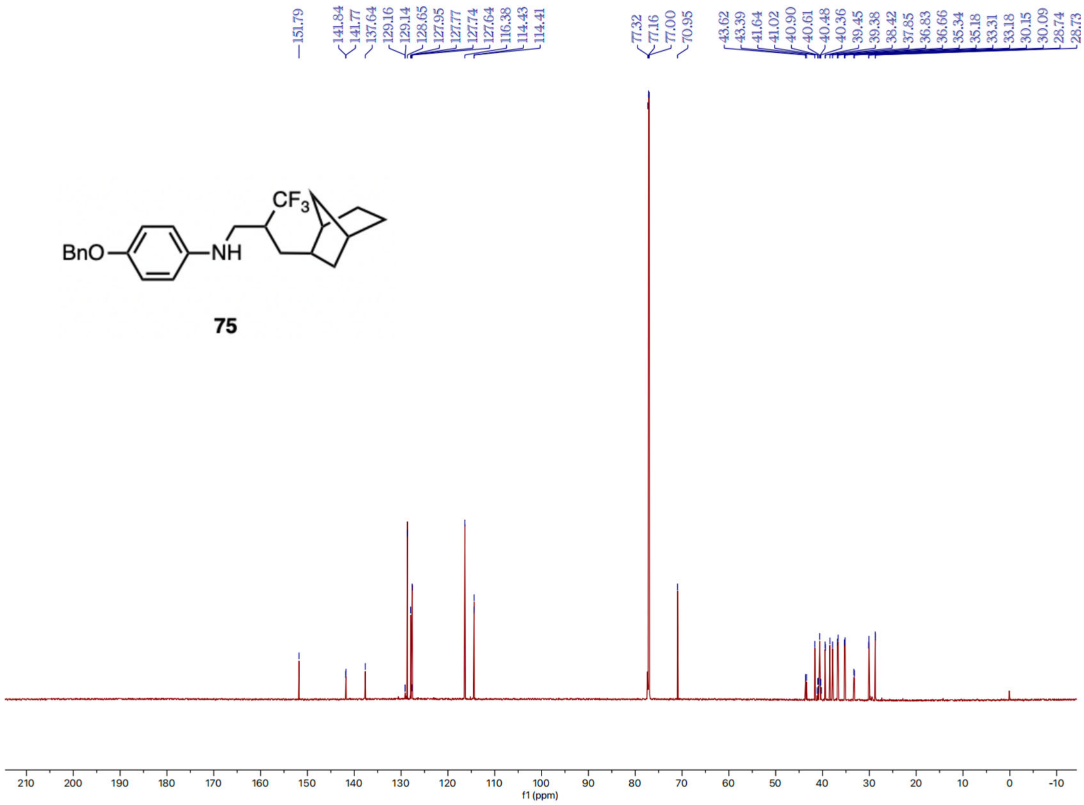


$^{13}$C NMR spectrum (201 MHz, Chloroform-*d*) of compound **75**

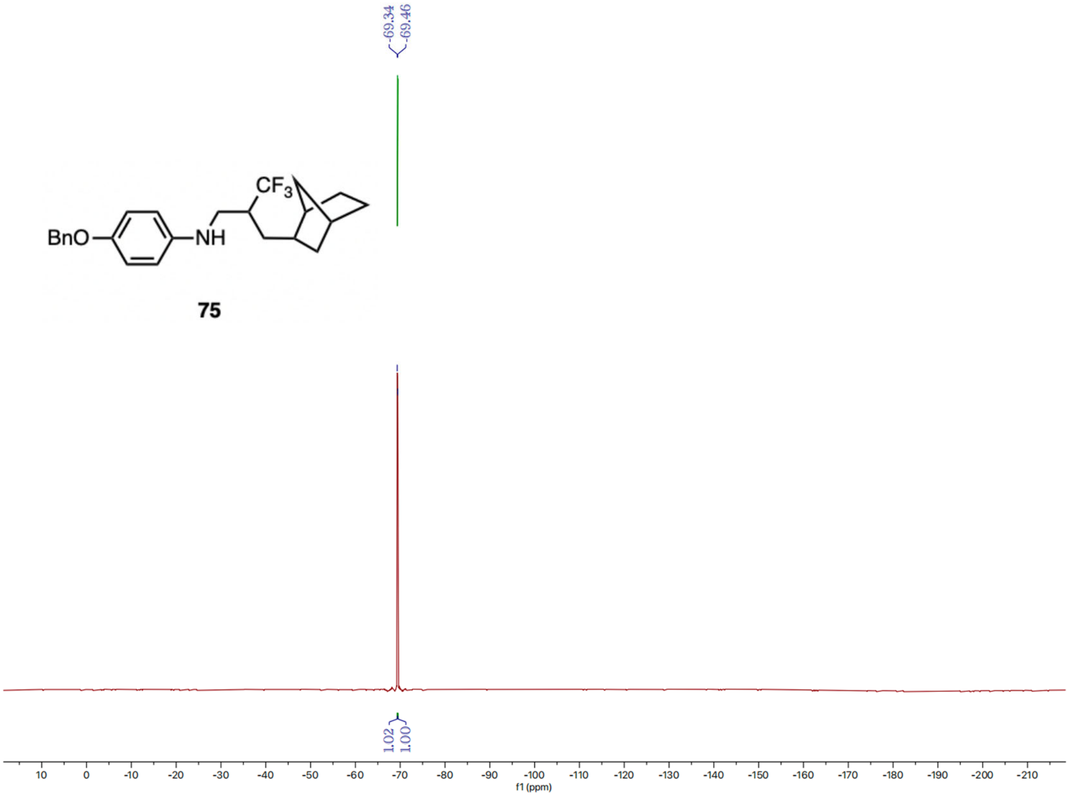


$^{19}$F NMR spectrum (376 MHz, Chloroform-*d*) of compound **75**

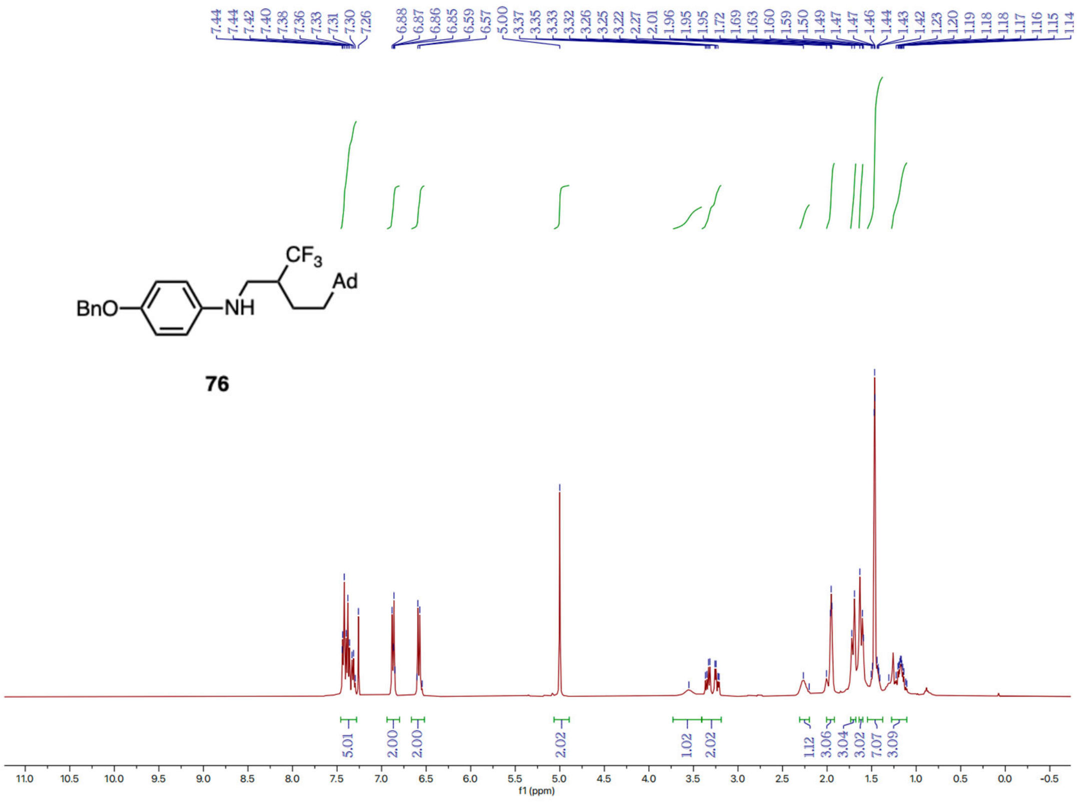

$^{1}$H NMR spectrum (400 MHz, Chloroform-*d*) of compound **76**

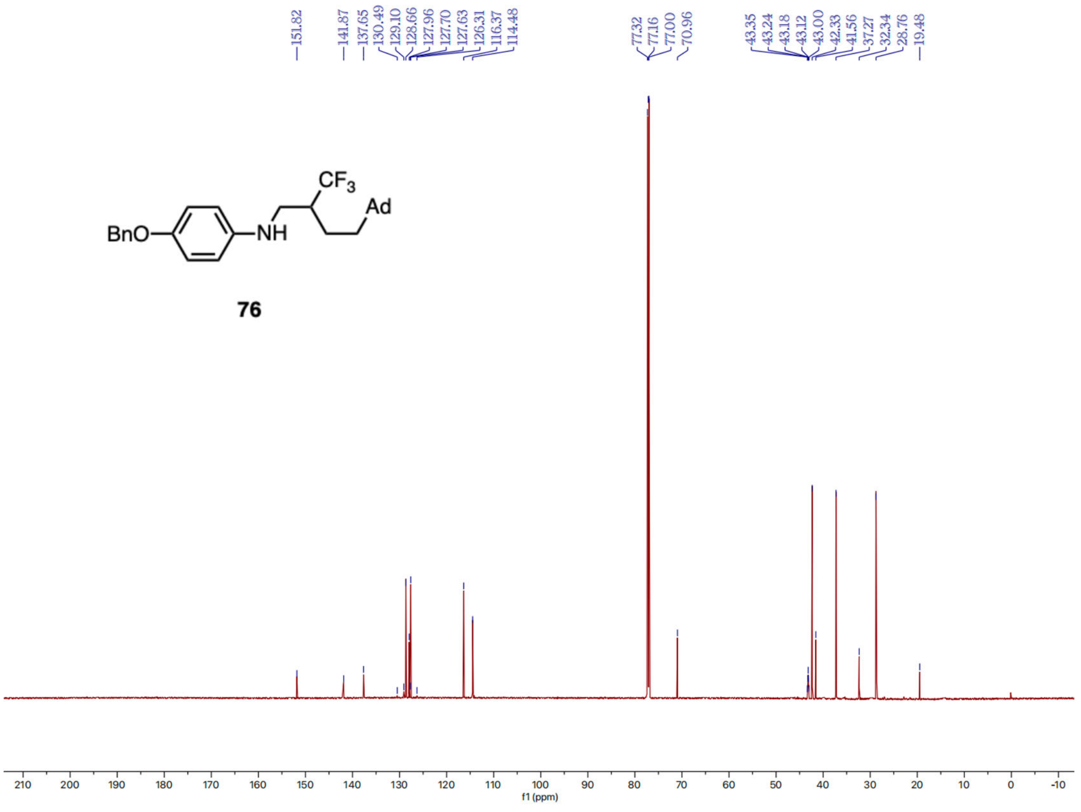

$^{13}$C NMR spectrum (201 MHz, Chloroform-*d*) of compound **76**

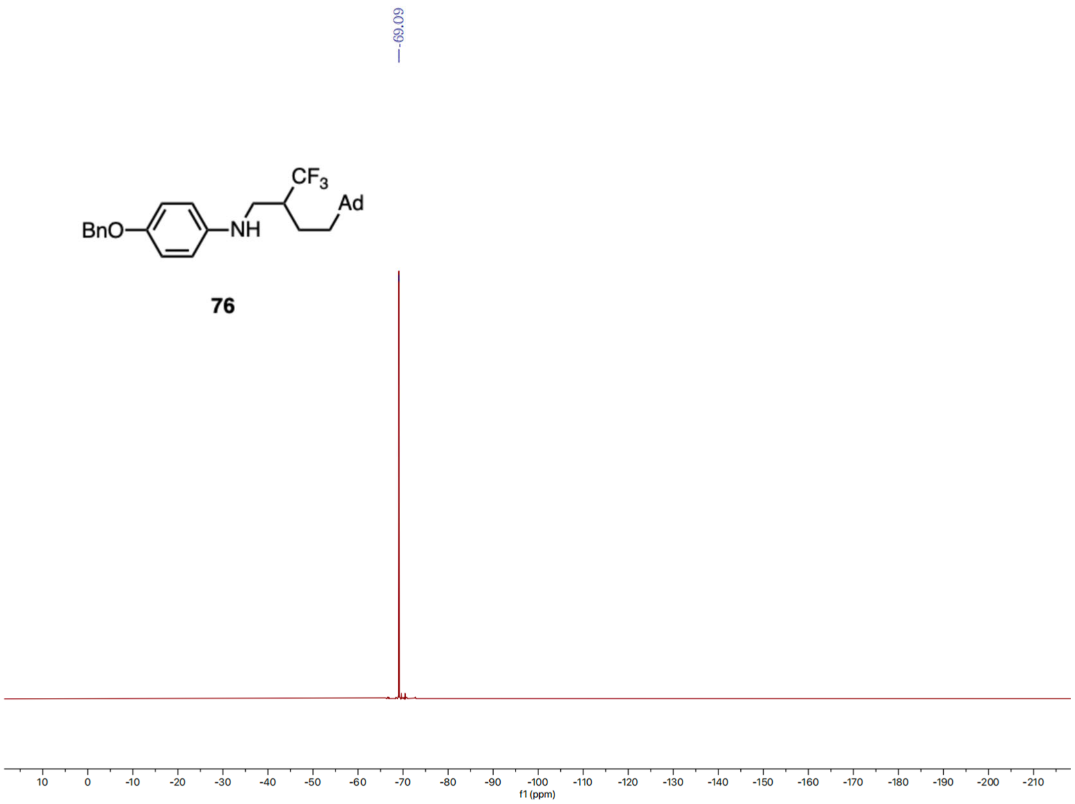


$^{19}F$ NMR spectrum (376 MHz, Chloroform-*d*) of compound **76**

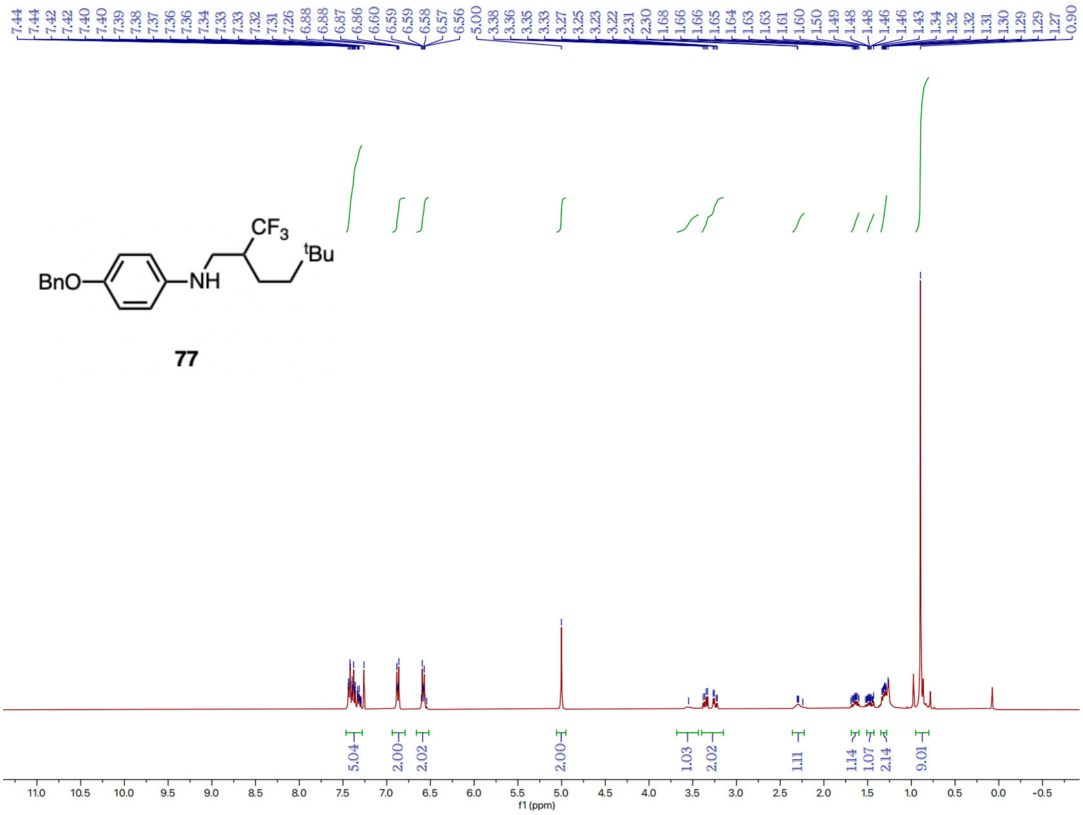


$^{1}$H NMR spectrum (400 MHz, Chloroform-*d*) of compound **77**

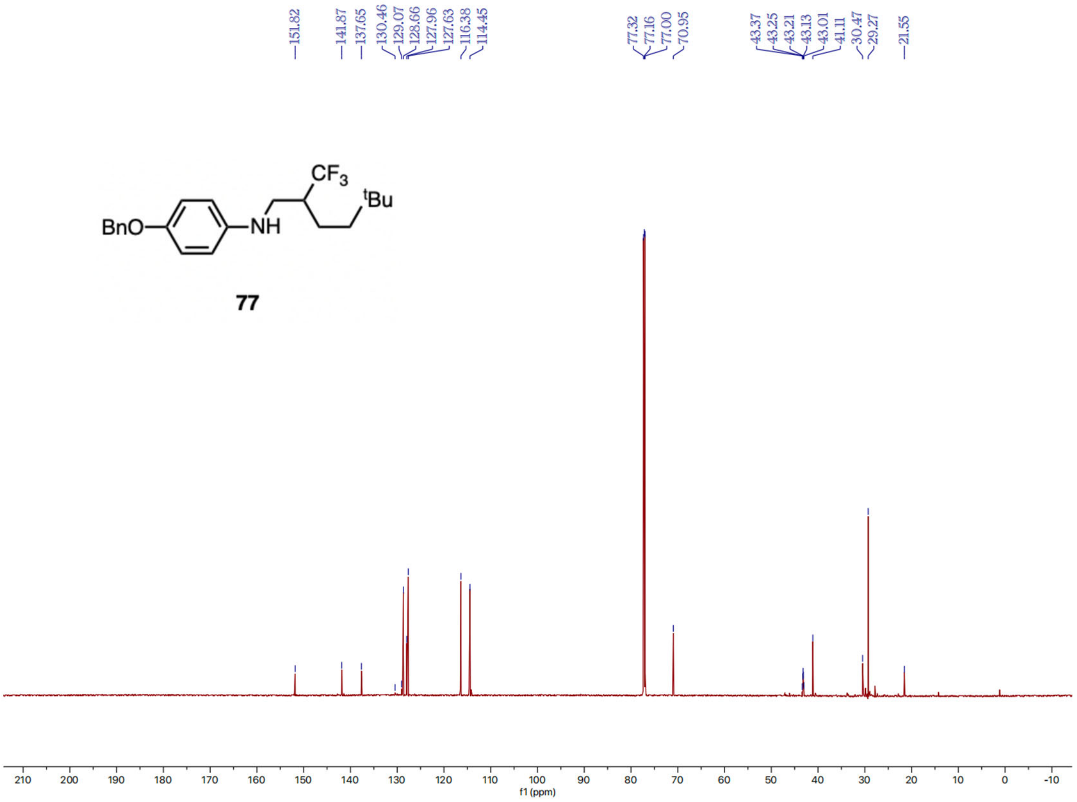


$^{13}$C NMR spectrum (201 MHz, Chloroform-*d*) of compound **77**

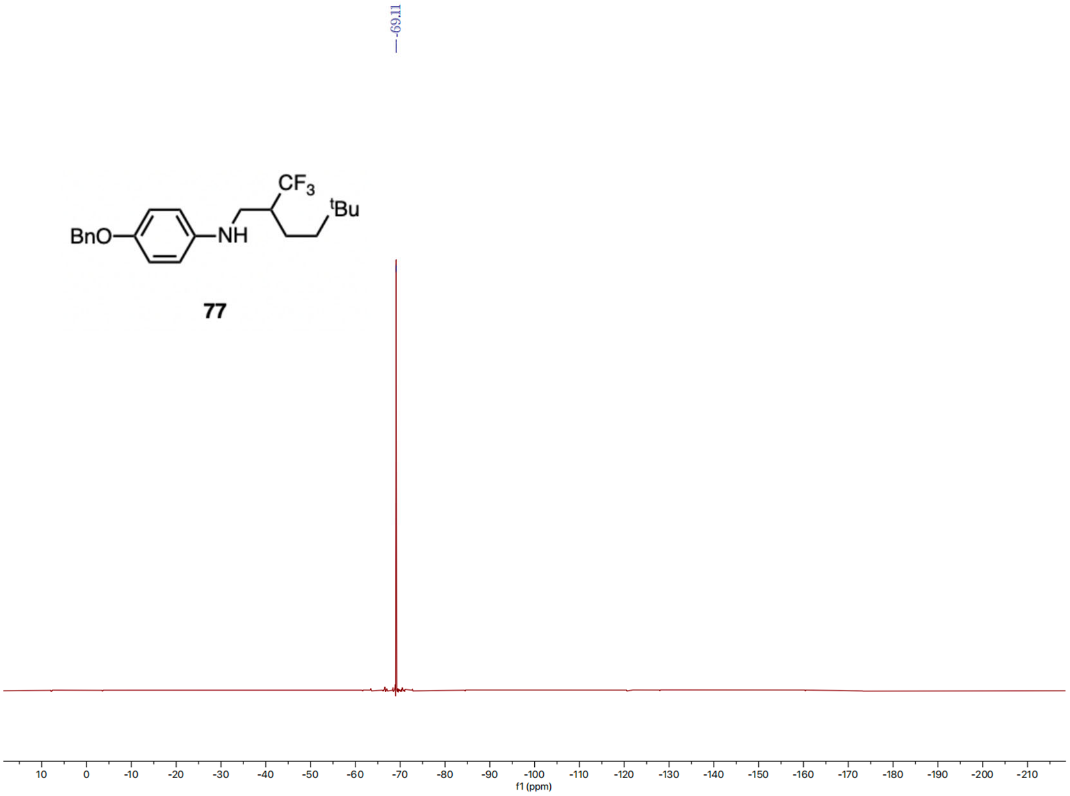


$^{19}F$ NMR spectrum (376 MHz, Chloroform-*d*) of compound **77**

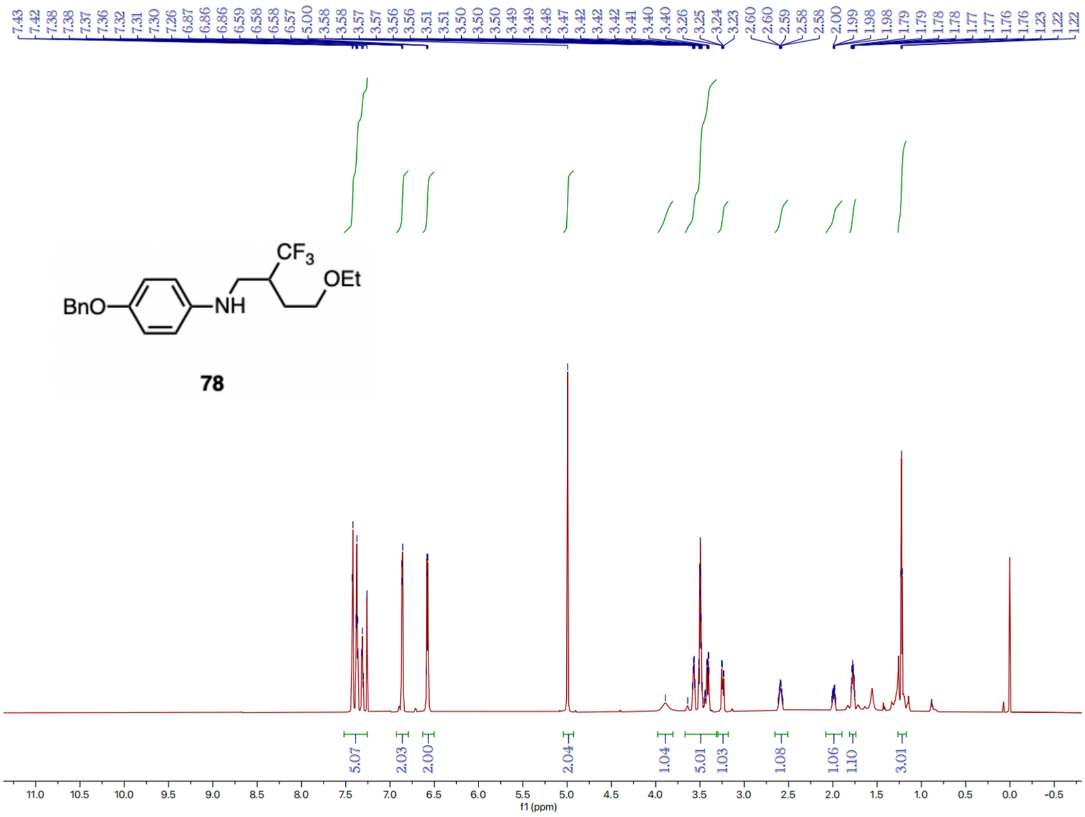

$^{1}$H NMR spectrum (800 MHz, Chloroform-*d*) of compound **78**

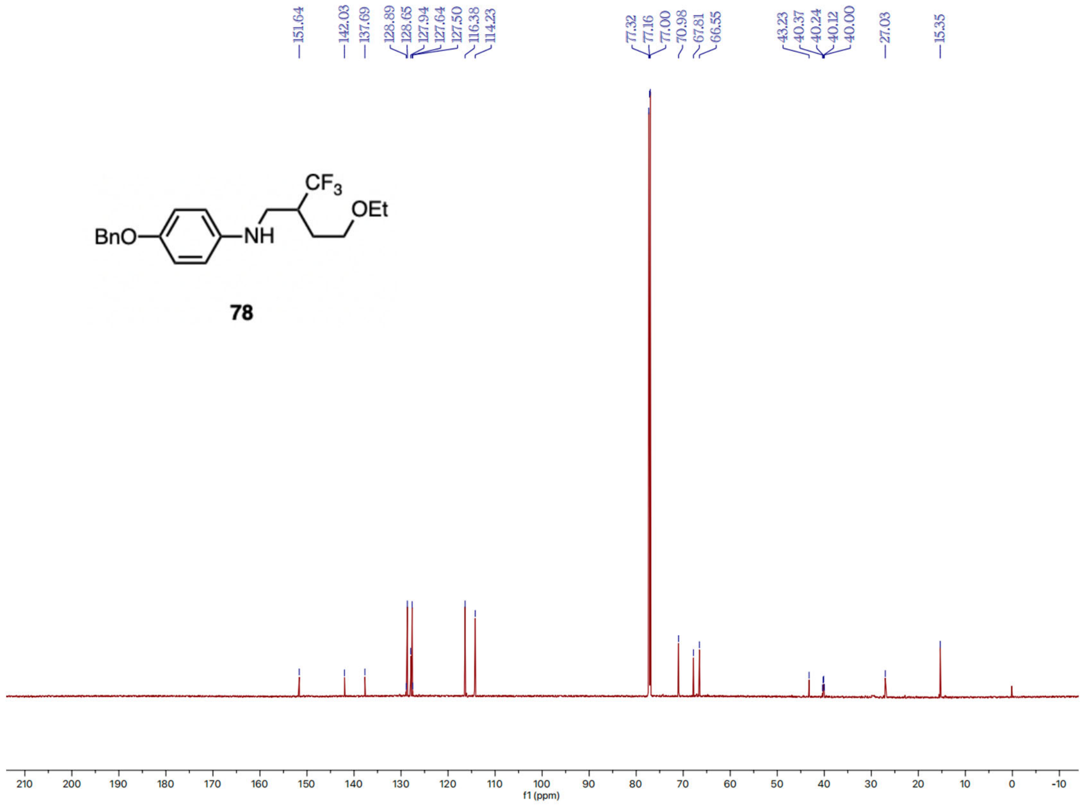

$^{13}$C NMR spectrum (201 MHz, Chloroform-*d*) of compound **78**

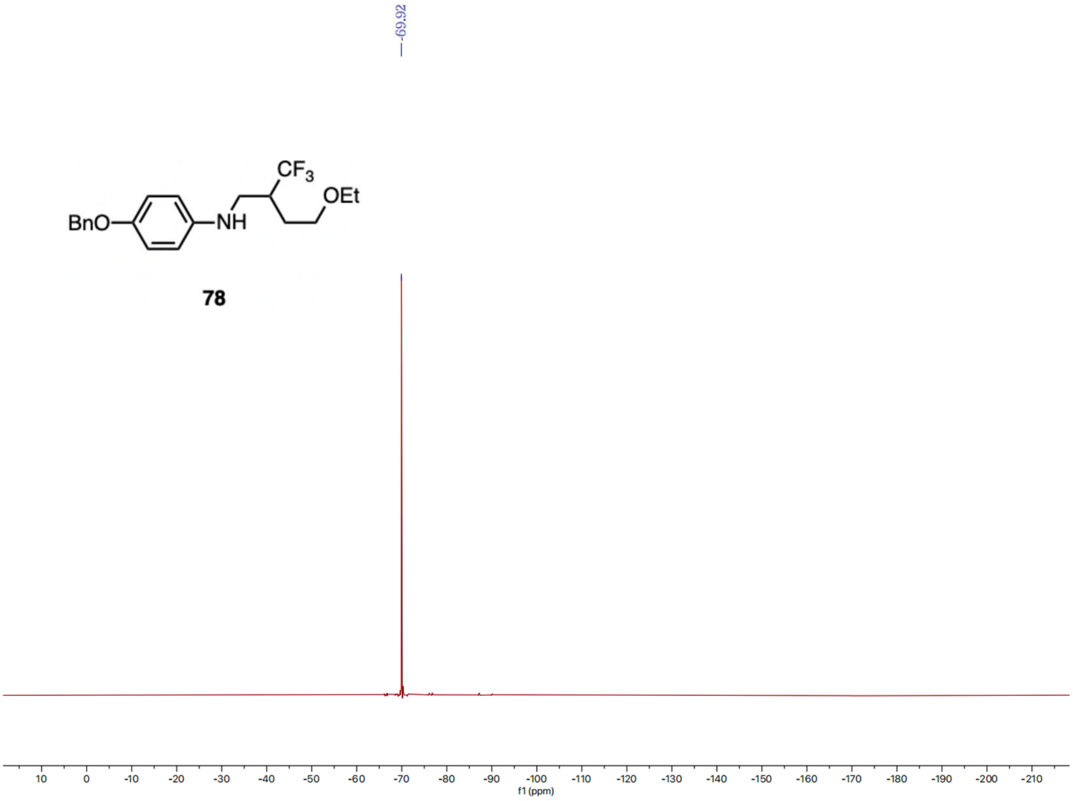


$^{19}F$ NMR spectrum (376 MHz, Chloroform-*d*) of compound **78**

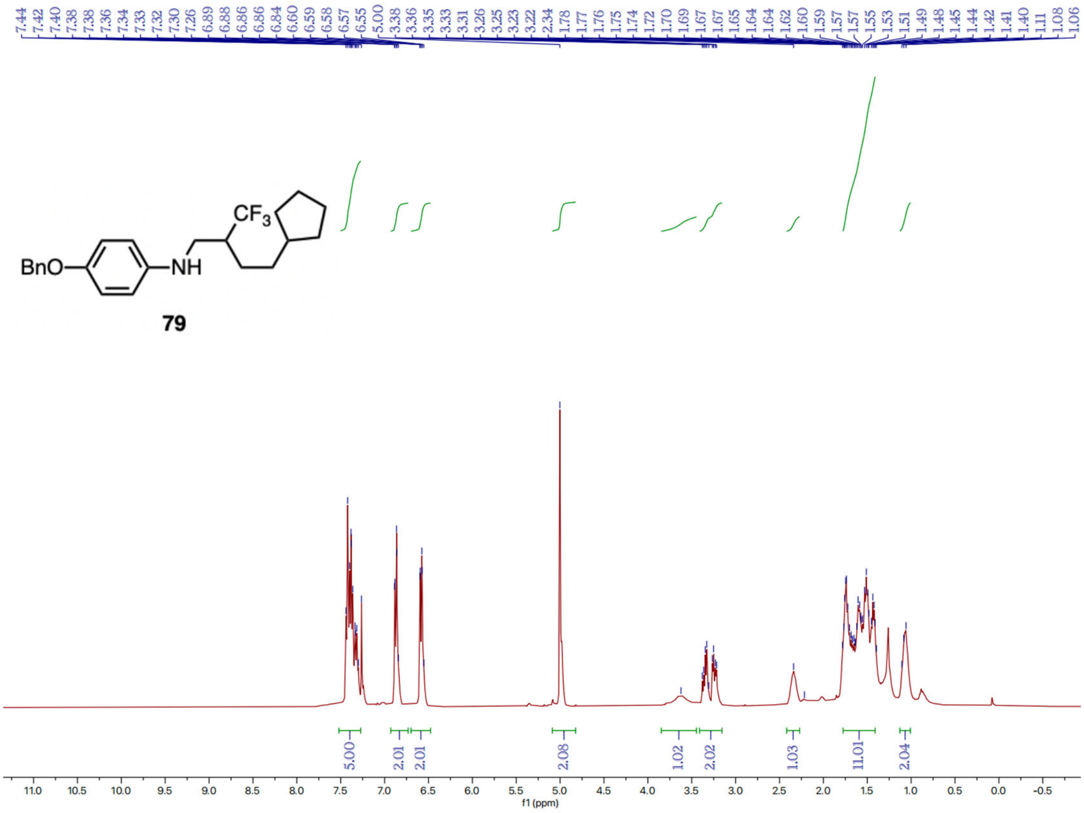

$^{1}$H NMR spectrum (400 MHz, Chloroform-*d*) of compound **79**

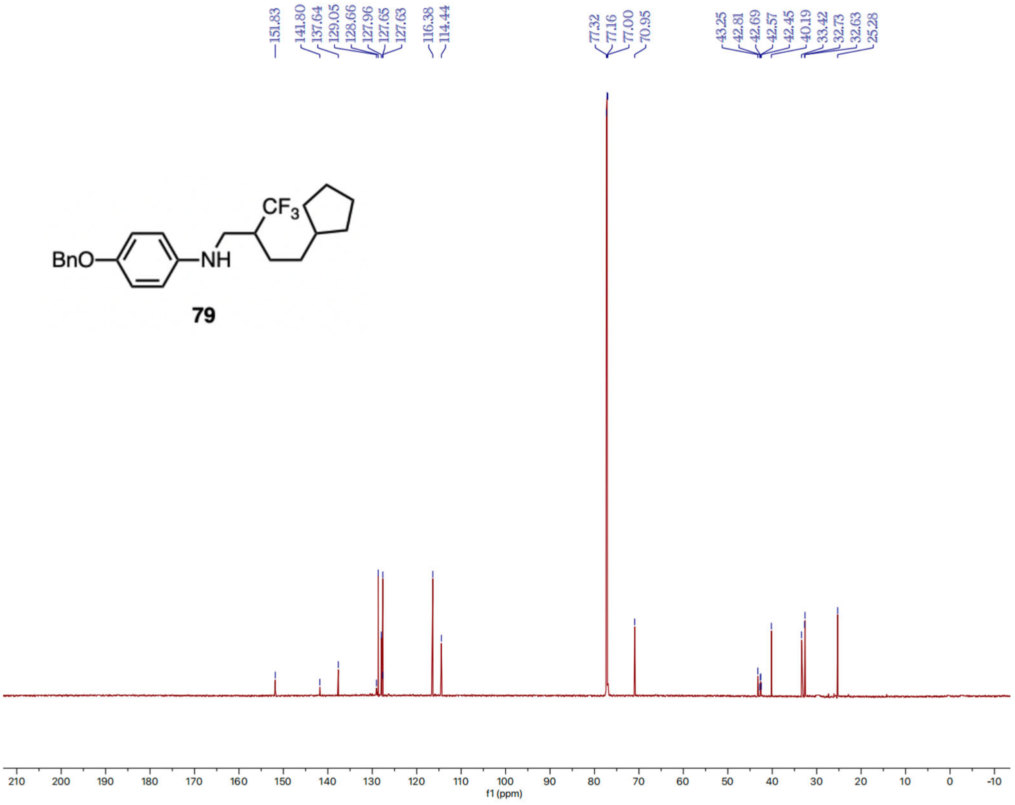

$^{13}$C NMR spectrum (201 MHz, Chloroform-*d*) of compound **79**

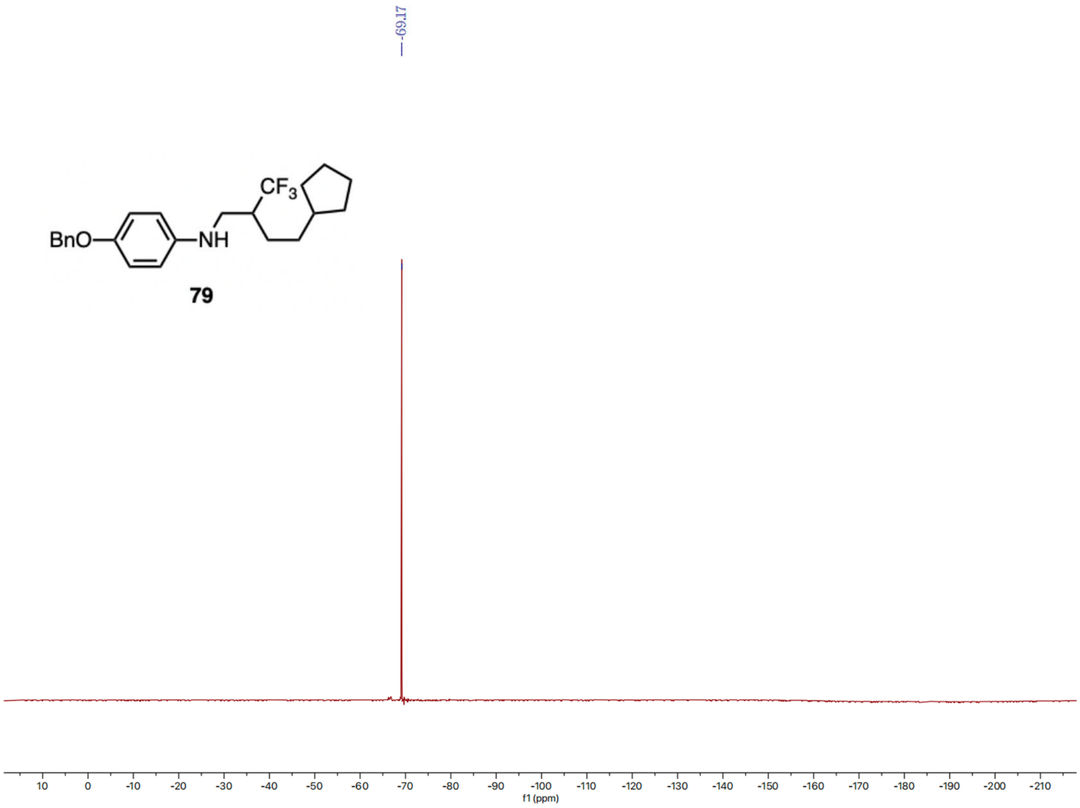

$^{19}$F NMR spectrum (376 MHz, Chloroform-*d*) of compound **79**

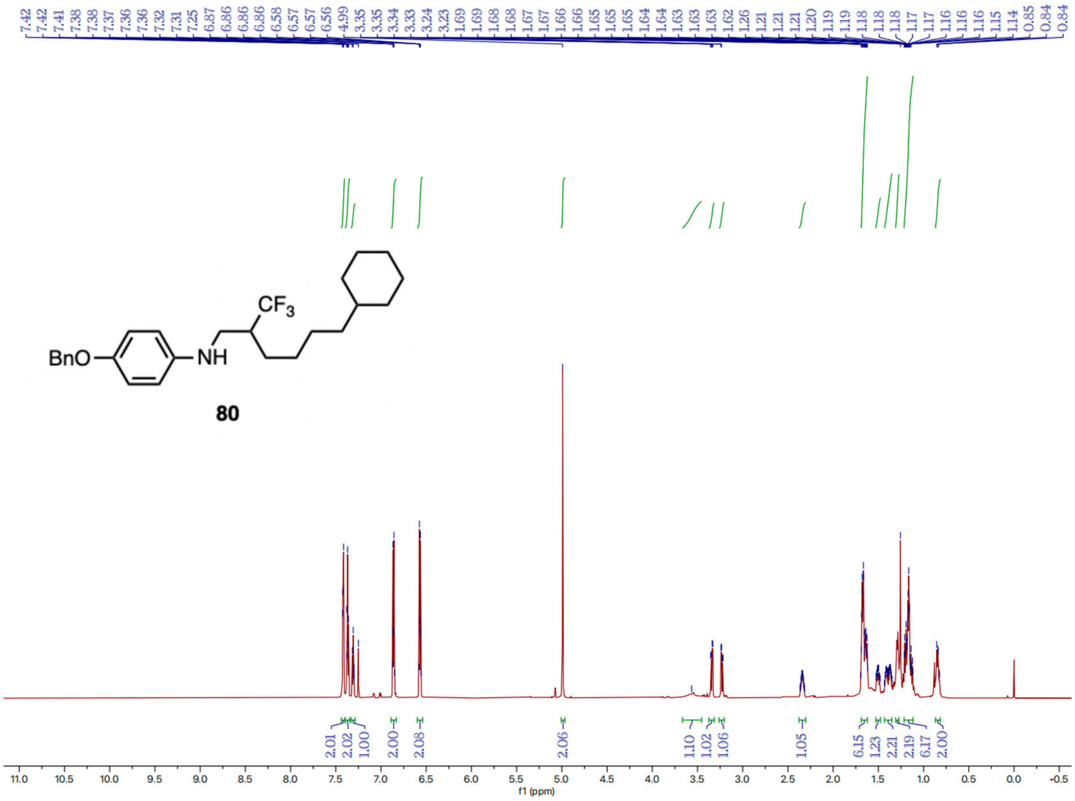


$^{1}$H NMR spectrum (800 MHz, Chloroform-*d*) of compound **80**

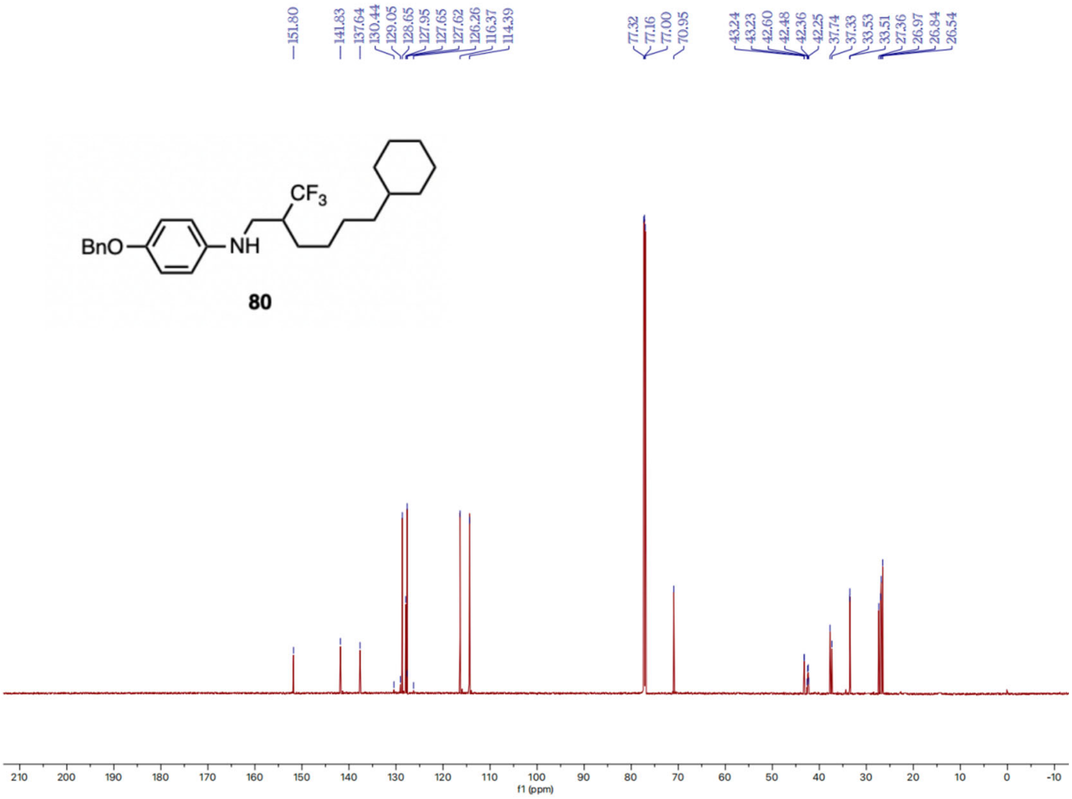


$^{13}$C NMR spectrum (201 MHz, Chloroform-*d*) of compound **80**

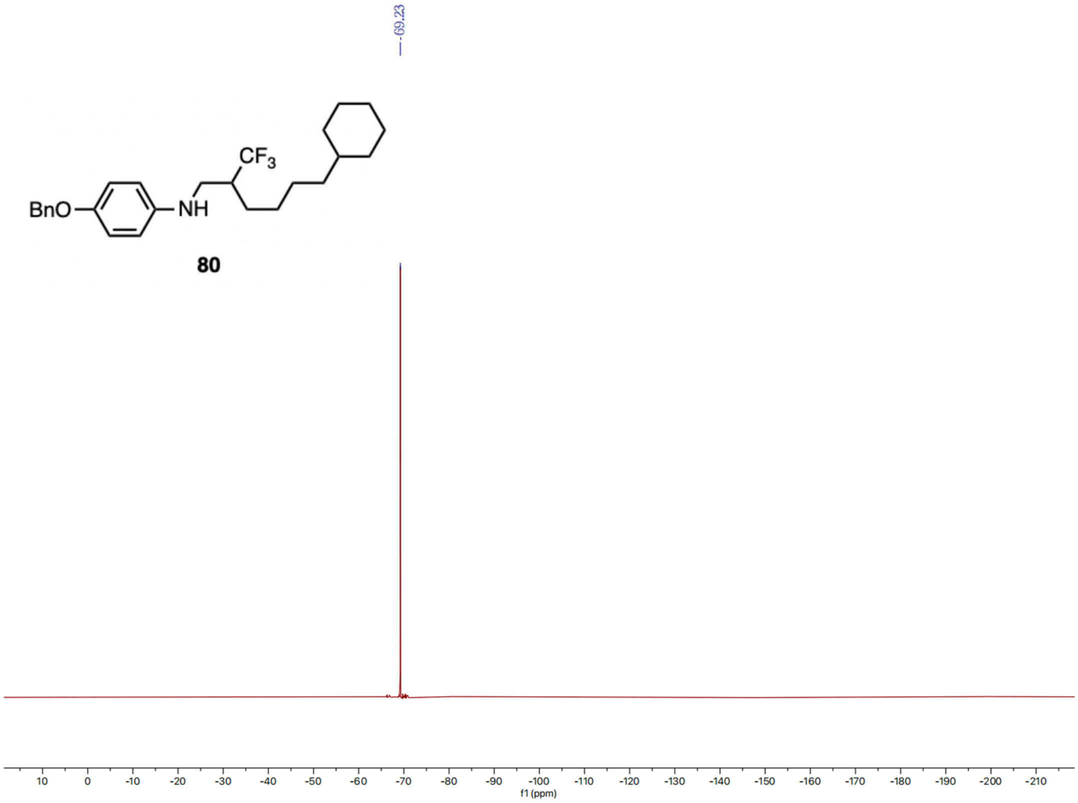


$^{19}F$ NMR spectrum (376 MHz, Chloroform-*d*) of compound **80**

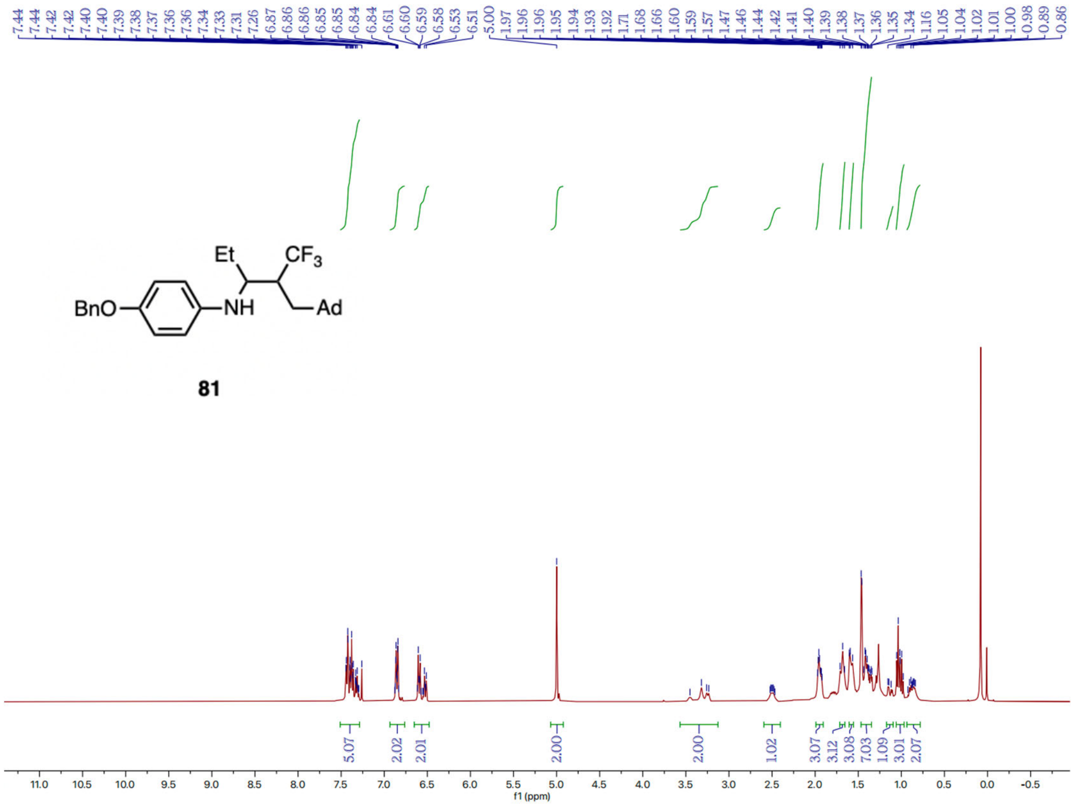


$^{1}$H NMR spectrum (400 MHz, Chloroform-*d*) of compound **81**

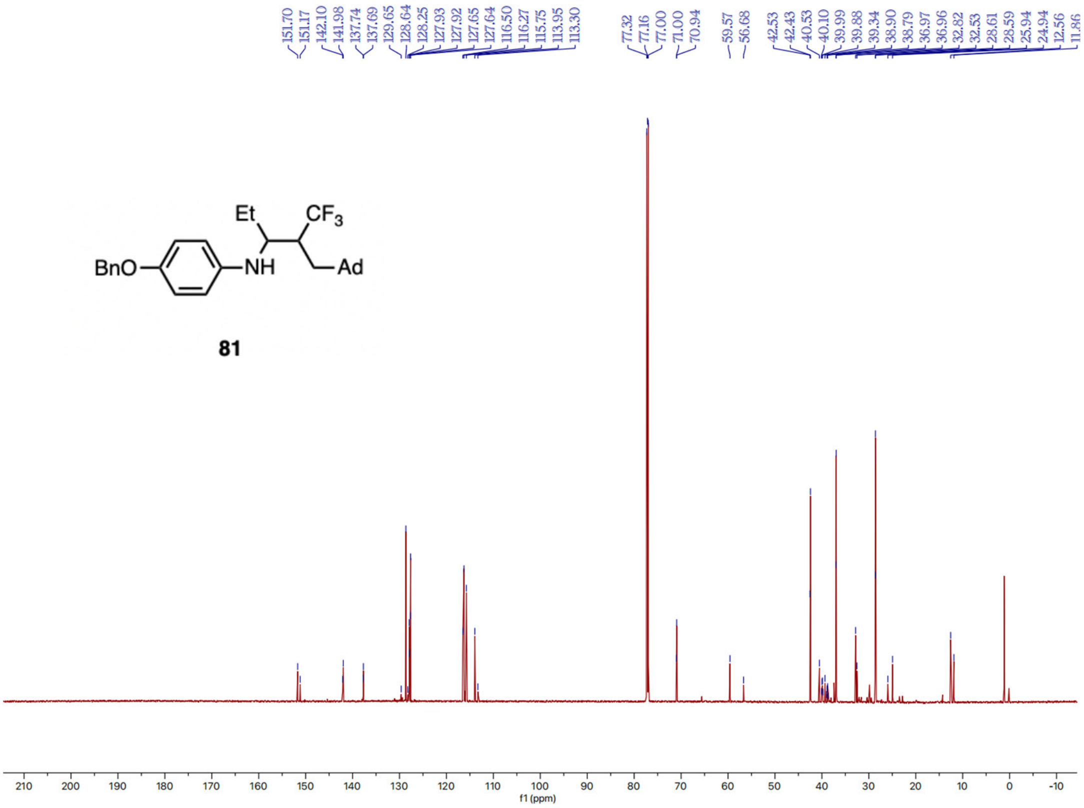


$^{13}$C NMR spectrum (201 MHz, Chloroform-*d*) of compound **81**

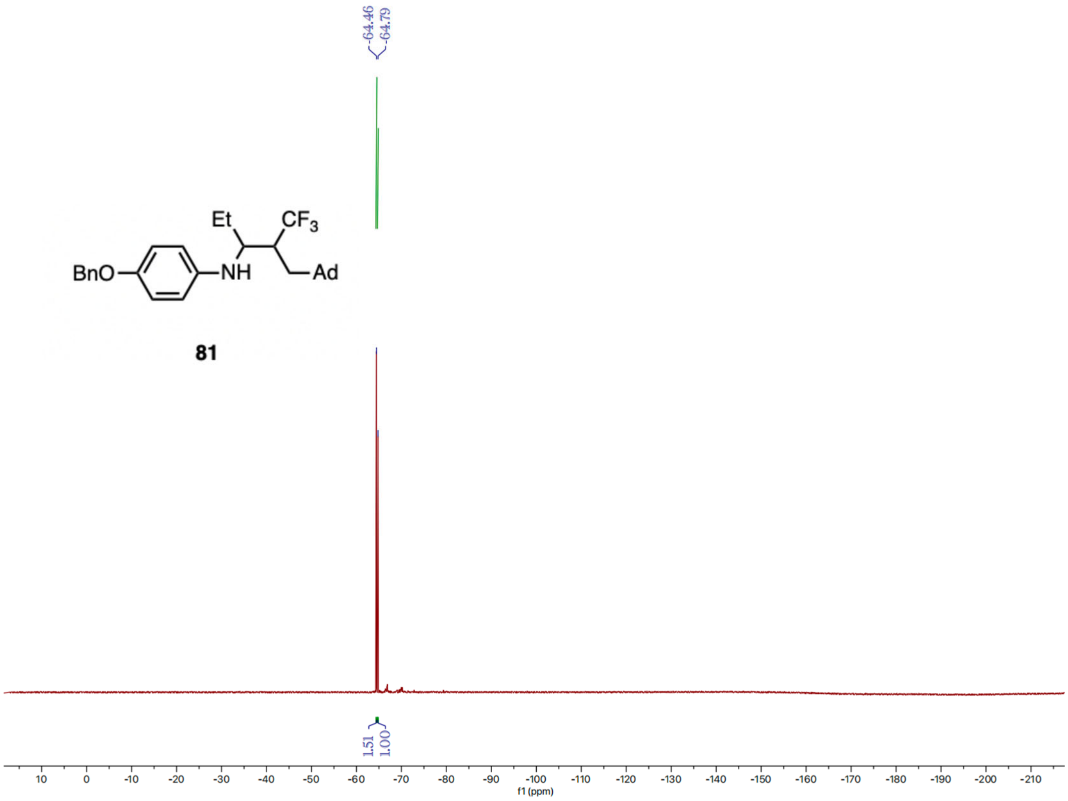


$^{19}F$ NMR spectrum (376 MHz, Chloroform-*d*) of compound **81**

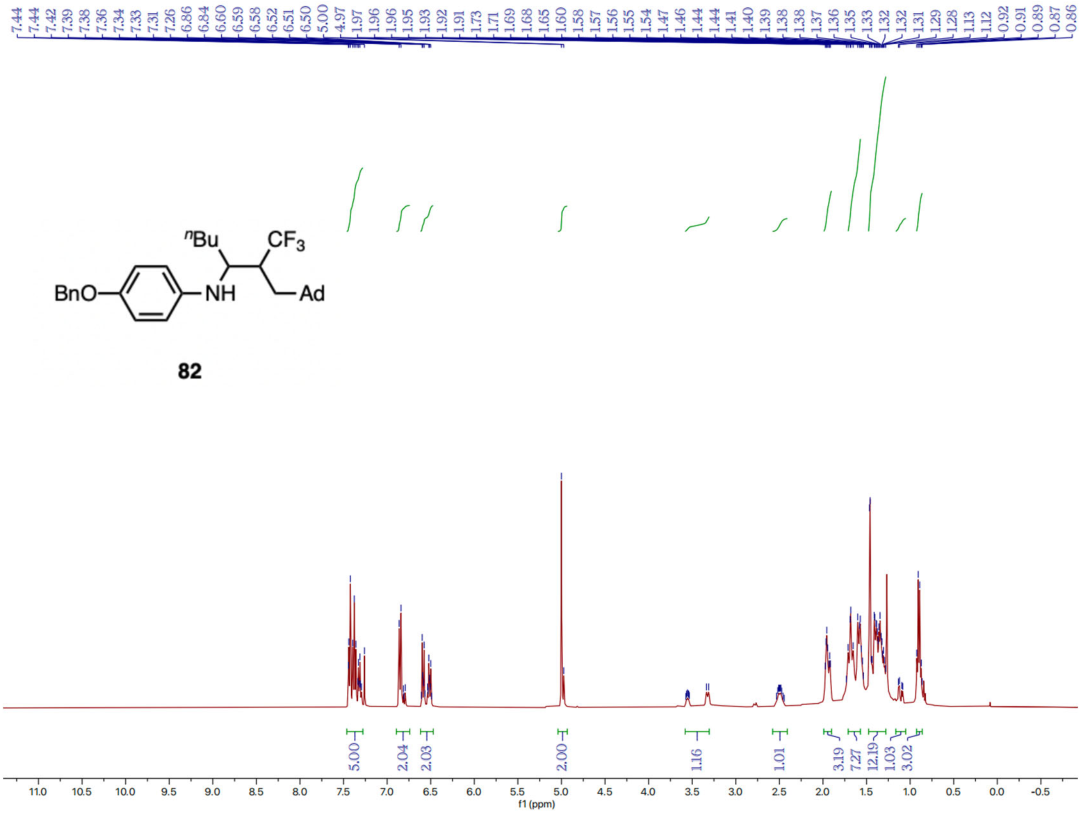


$^{1}$H NMR spectrum (400 MHz, Chloroform-*d*) of compound **82**

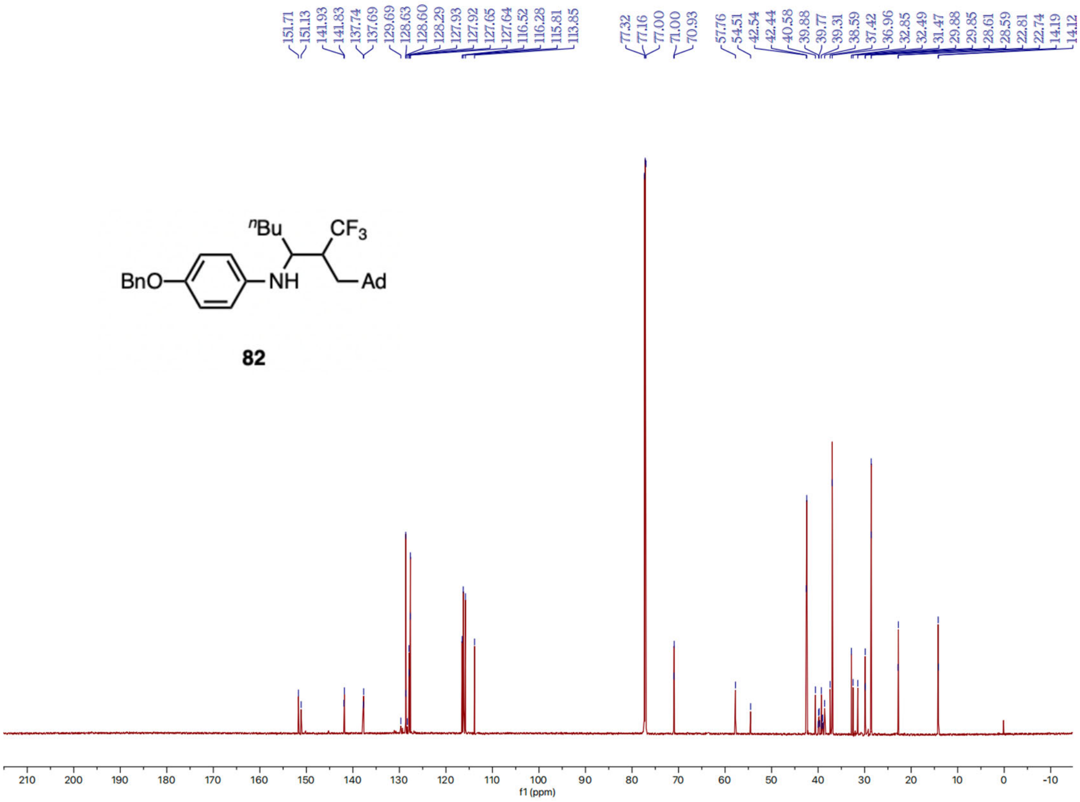


$^{13}$C NMR spectrum (201 MHz, Chloroform-*d*) of compound **82**

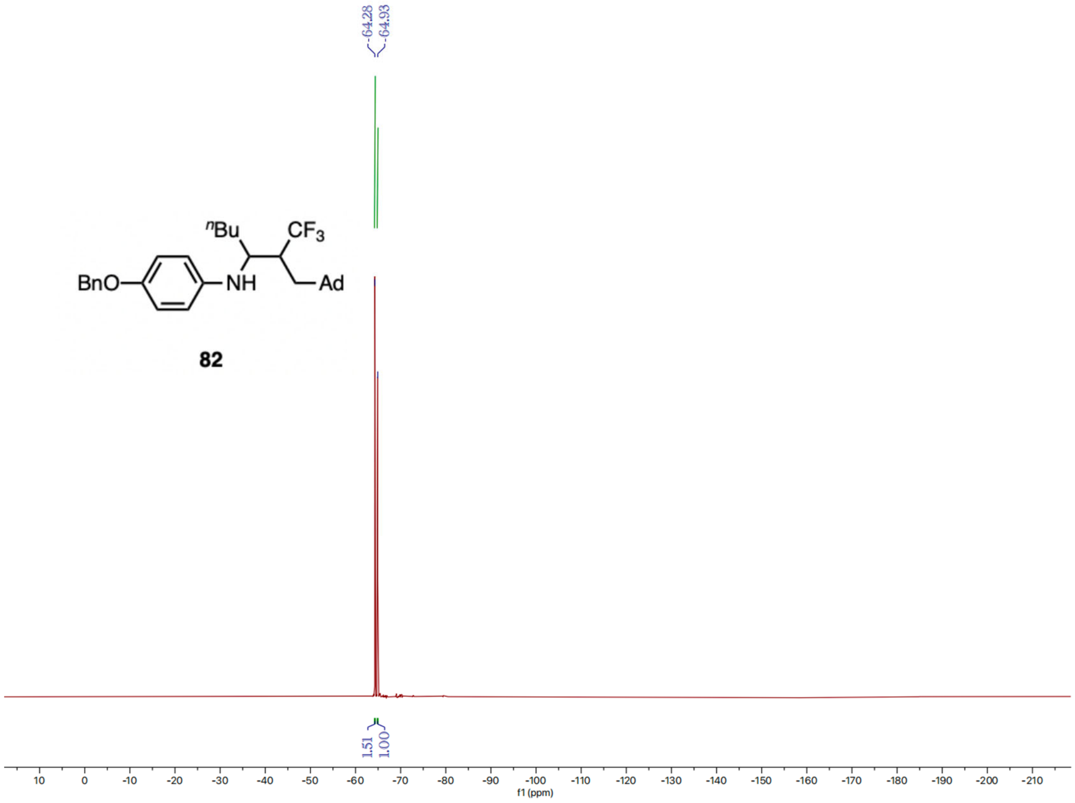


$^{19}$F NMR spectrum (376 MHz, Chloroform-*d*) of compound **82**

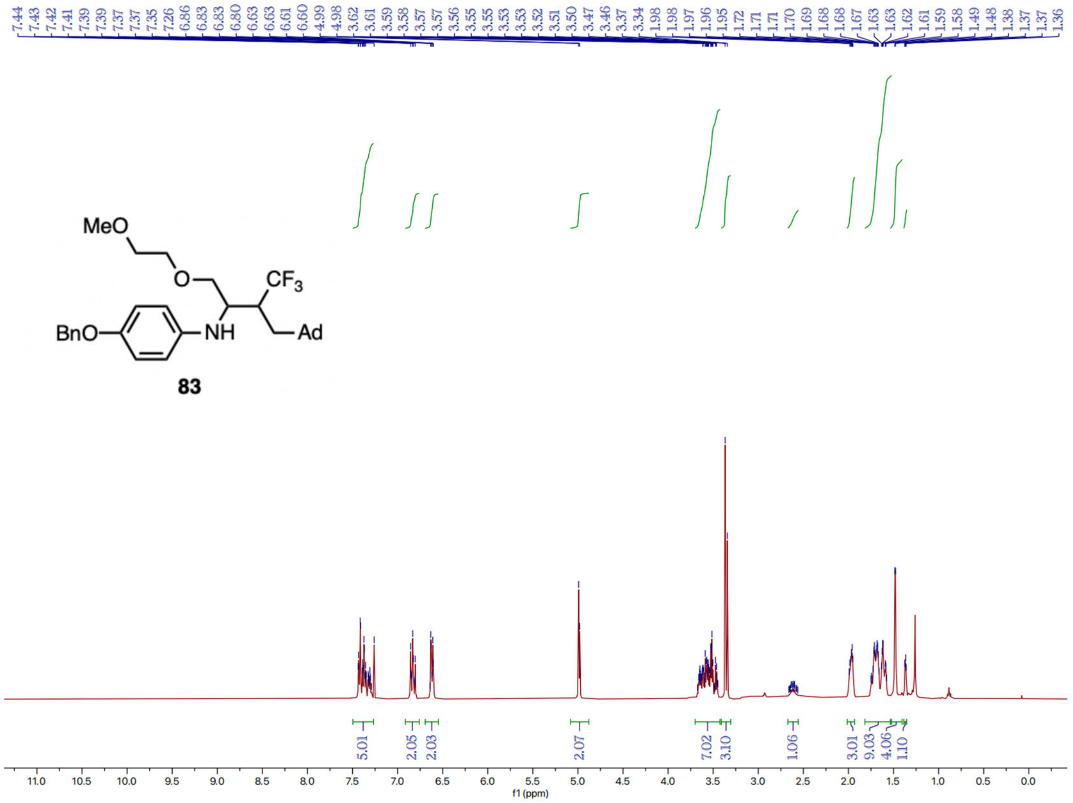


$^1$H NMR spectrum (400 MHz, Chloroform-*d*) of compound **83**

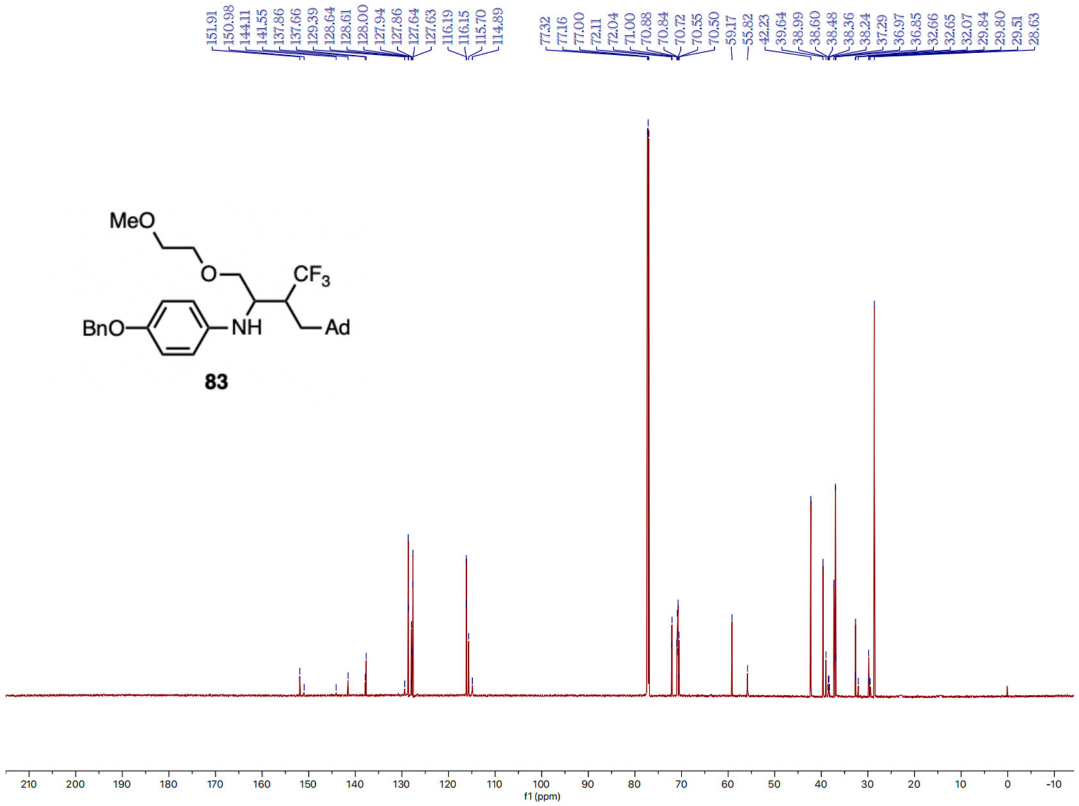


$^{13}$C NMR spectrum (201 MHz, Chloroform-*d*) of compound **83**

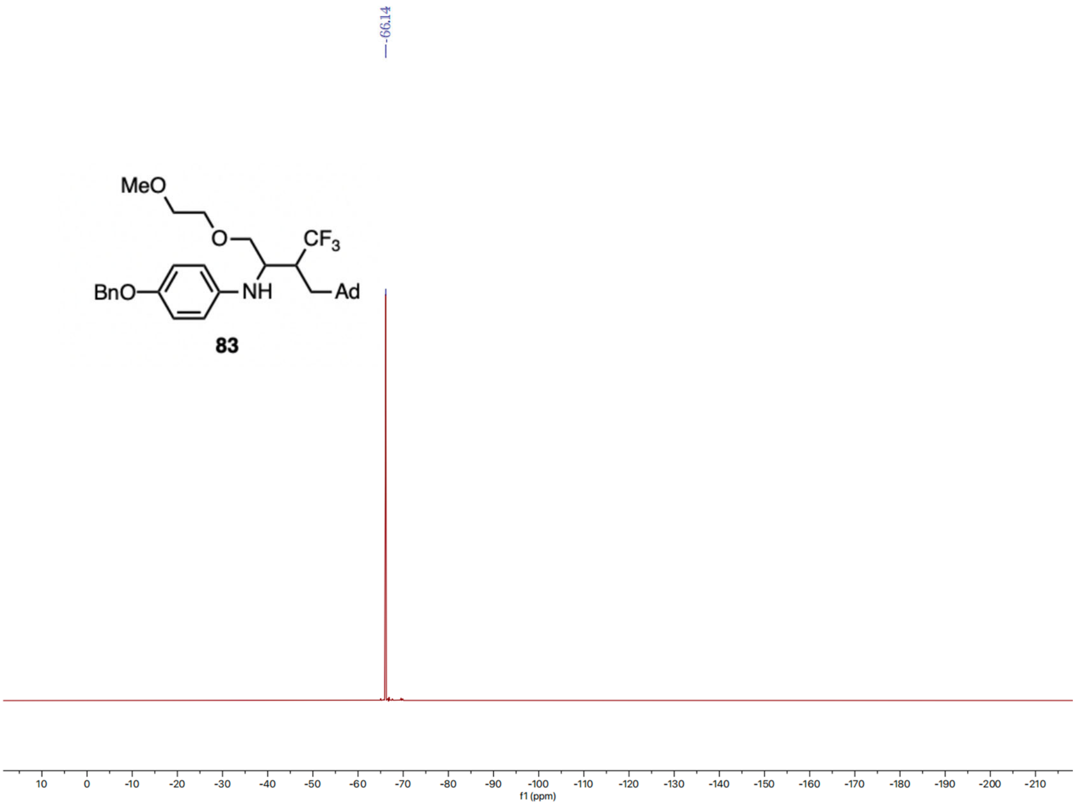


$^{19}$F NMR spectrum (376 MHz, Chloroform-*d*) of compound **83**

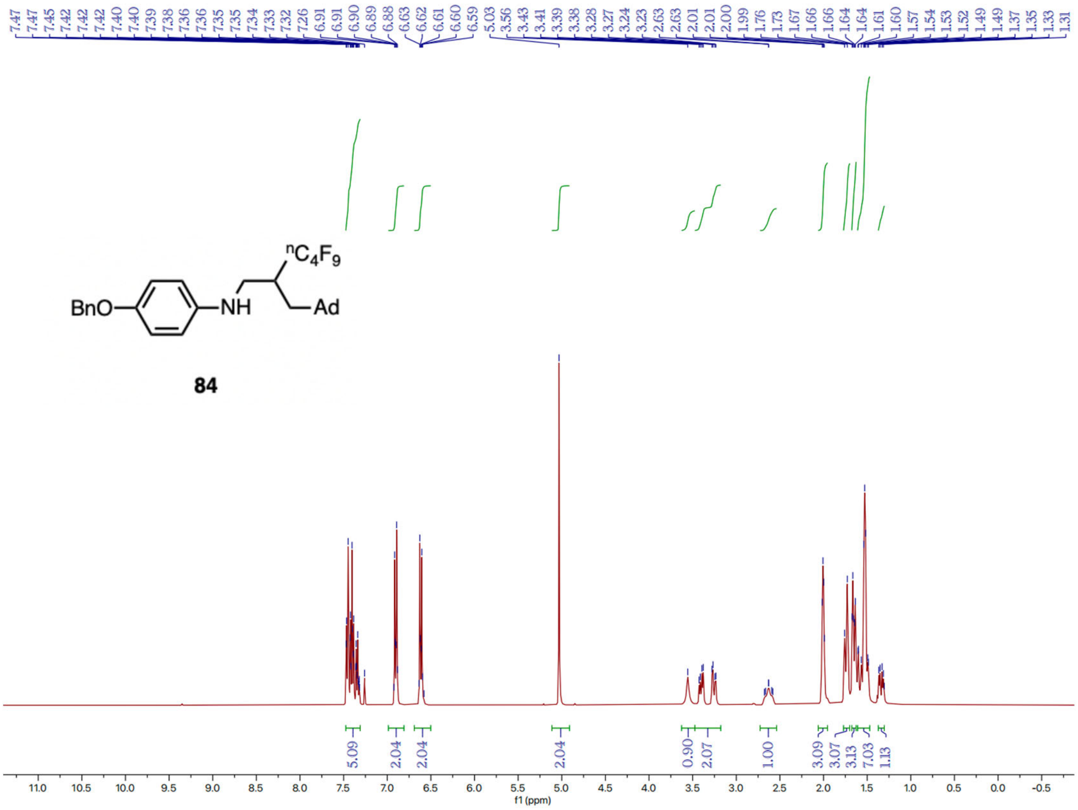


$^{1}H$ NMR spectrum (400 MHz, Chloroform-*d*) of compound **84**

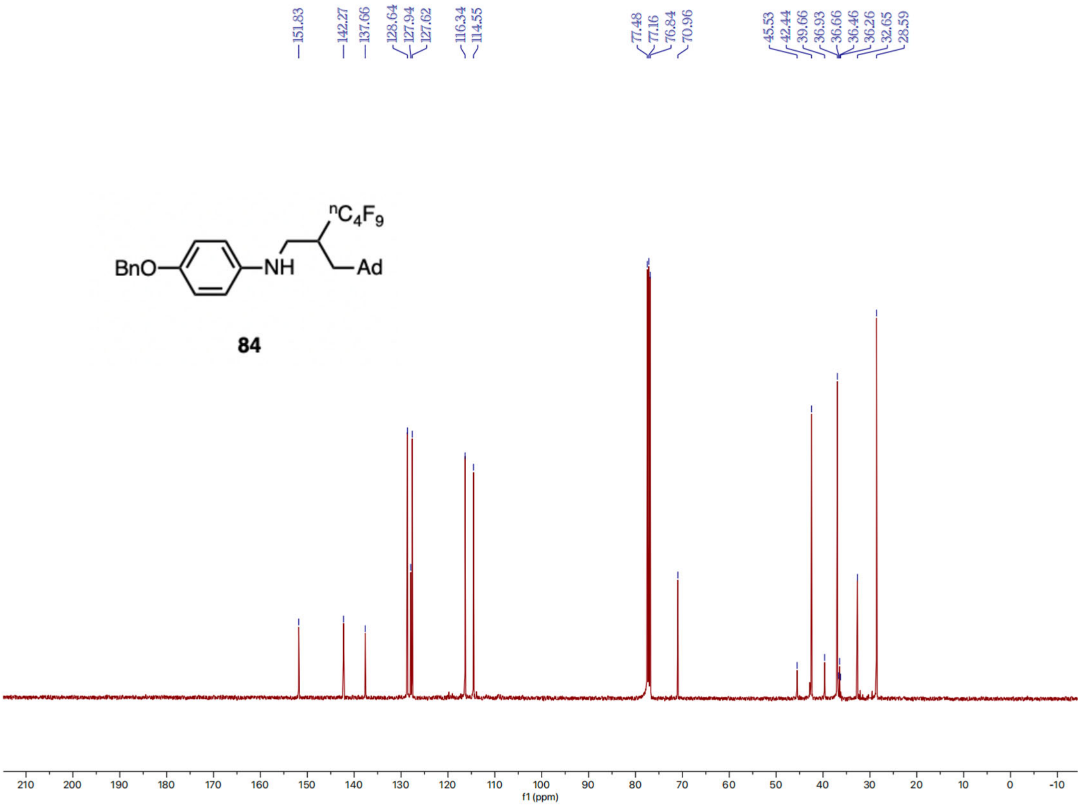


$^{13}C$ NMR spectrum (101 MHz, Chloroform-*d*) of compound **84**

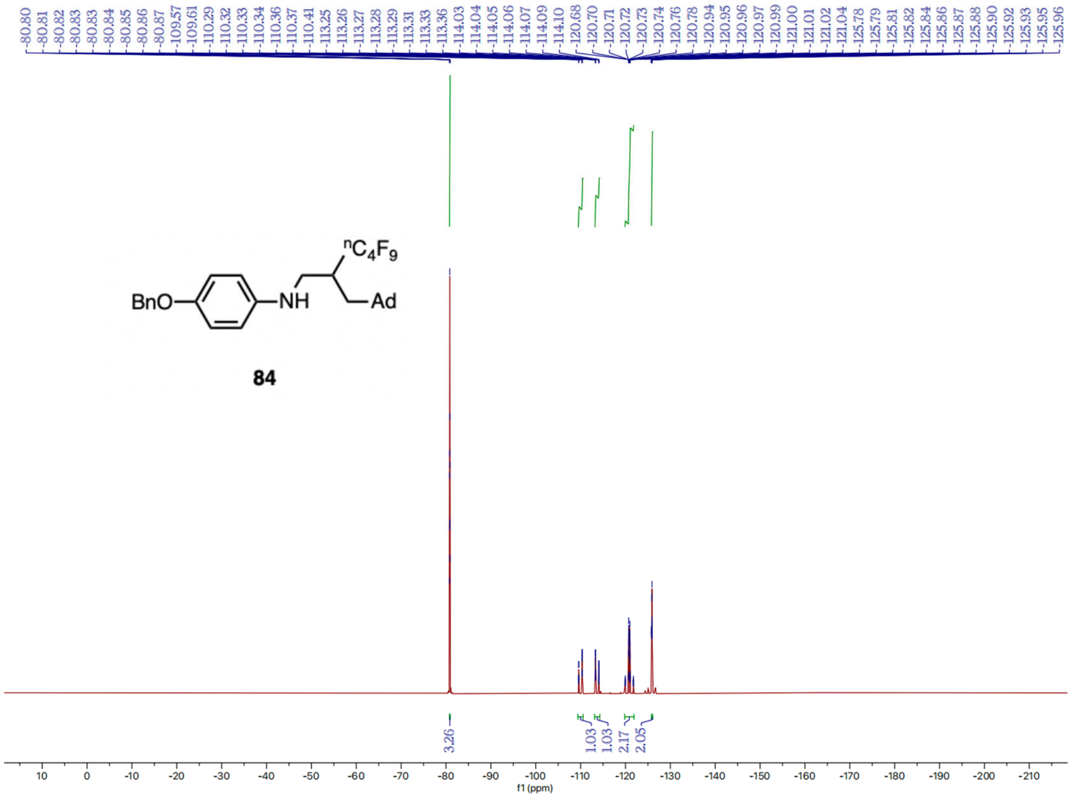

$^{19}$F NMR spectrum (376 MHz, Chloroform-*d*) of compound **84**

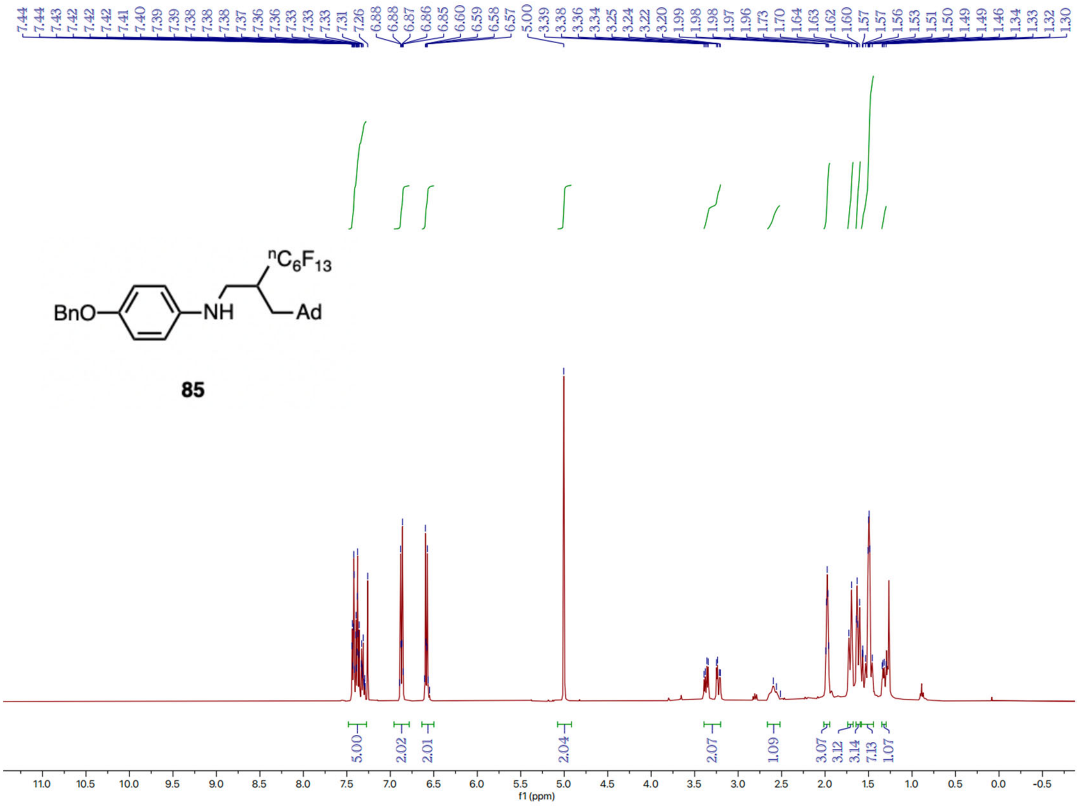

$^1H$ NMR spectrum (400 MHz, Chloroform-*d*) of compound **85**

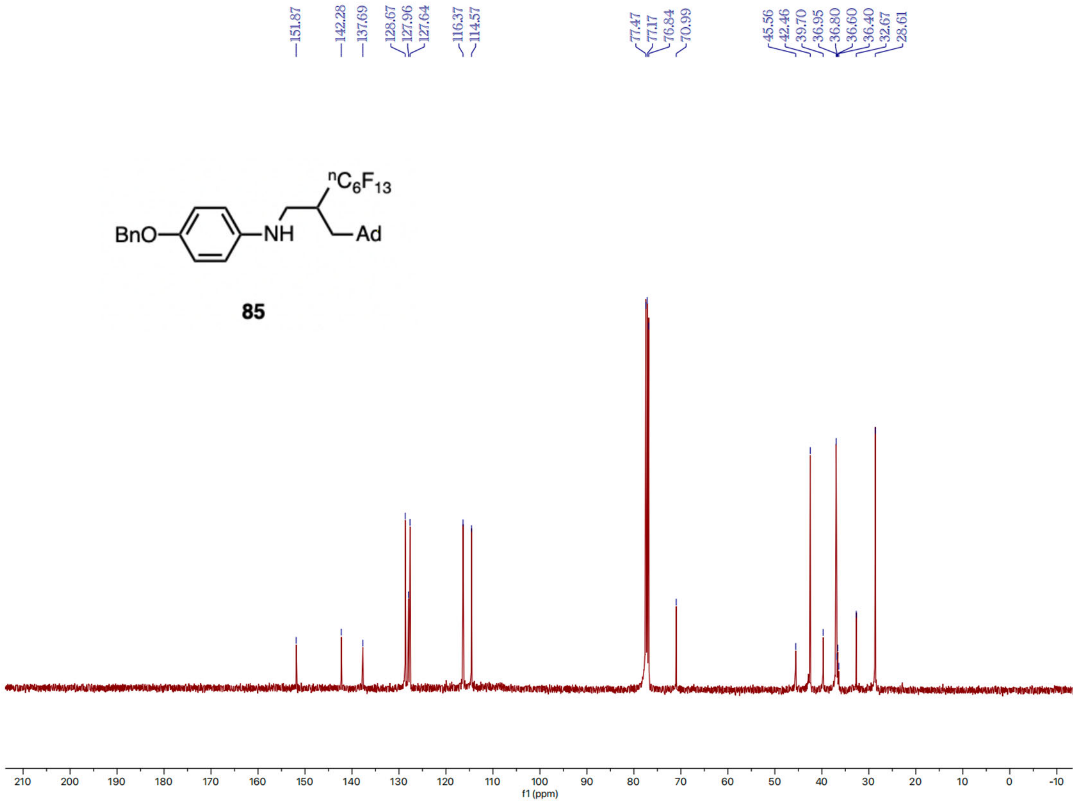

$^{13}C$ NMR spectrum (101 MHz, Chloroform-*d*) of compound **85**

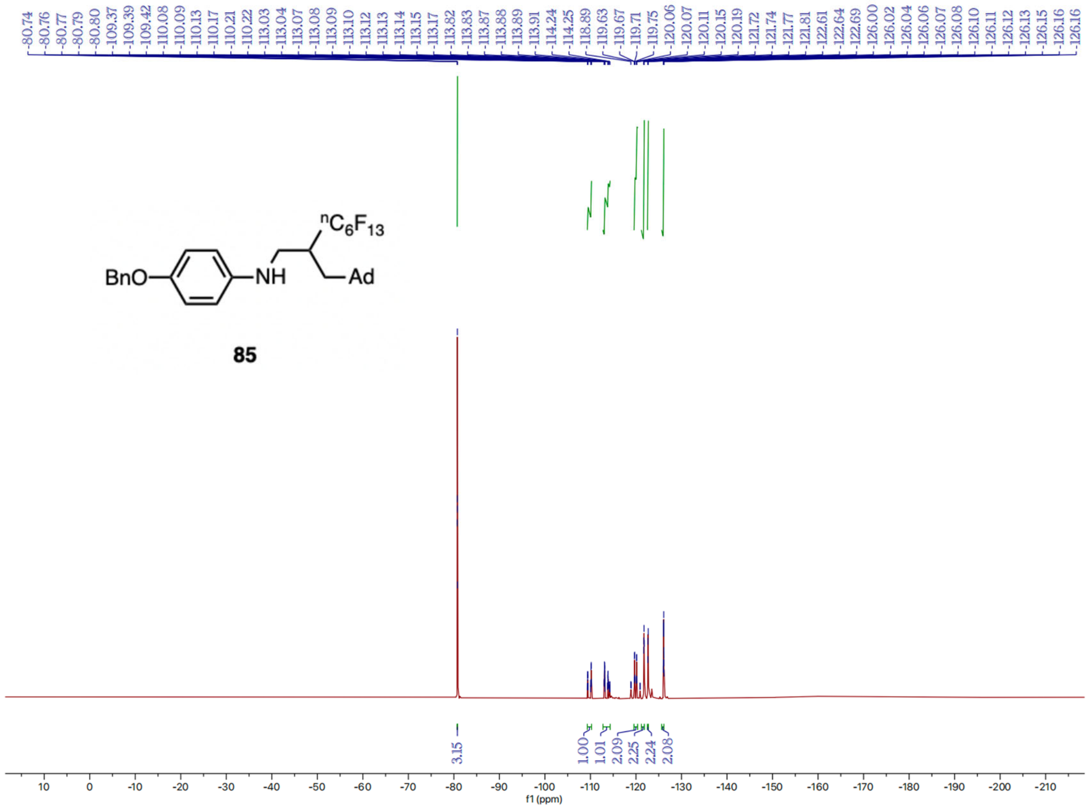

$^{19}$F NMR spectrum (376 MHz, Chloroform-*d*) of compound **85**

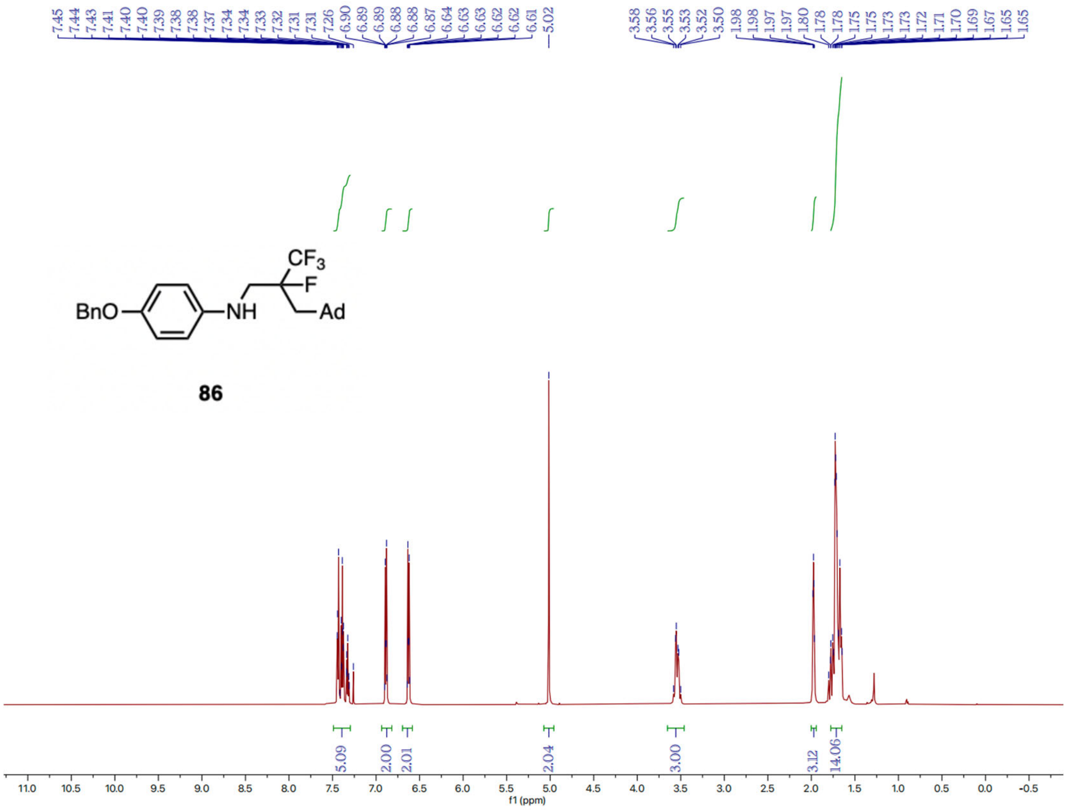


$^{1}$H NMR spectrum (600 MHz, Chloroform-*d*) of compound **86**

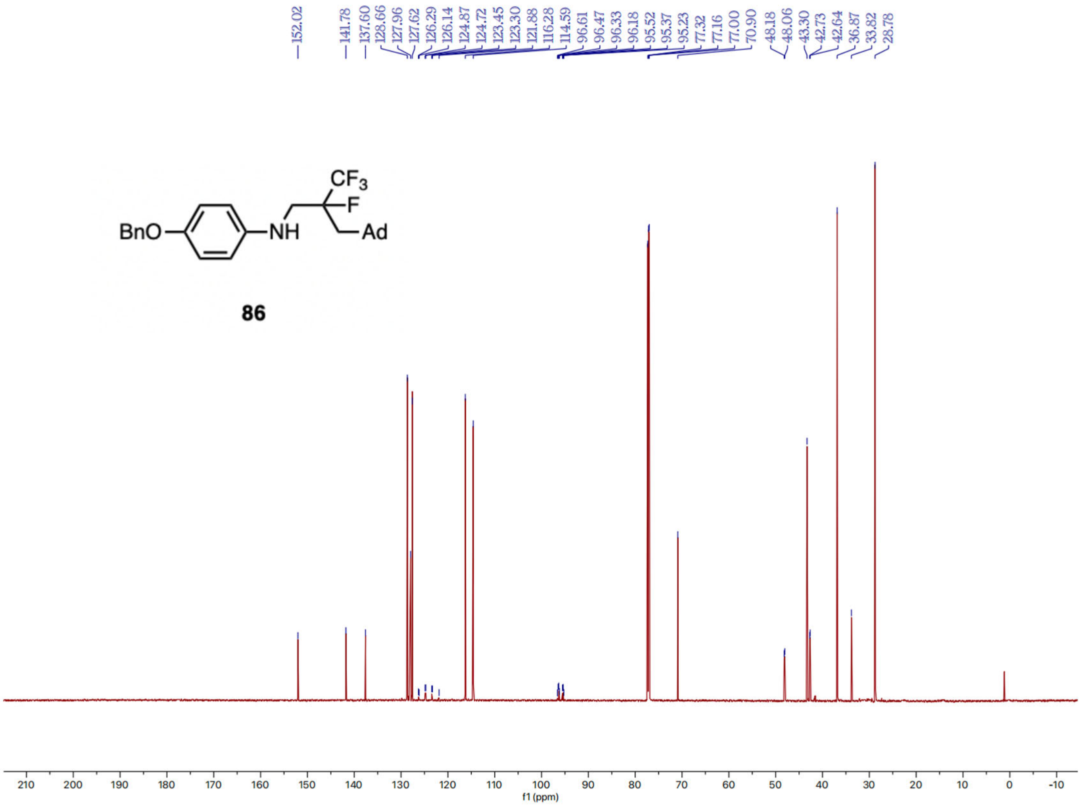


$^{13}$C NMR spectrum (201 MHz, Chloroform-*d*) of compound **86**

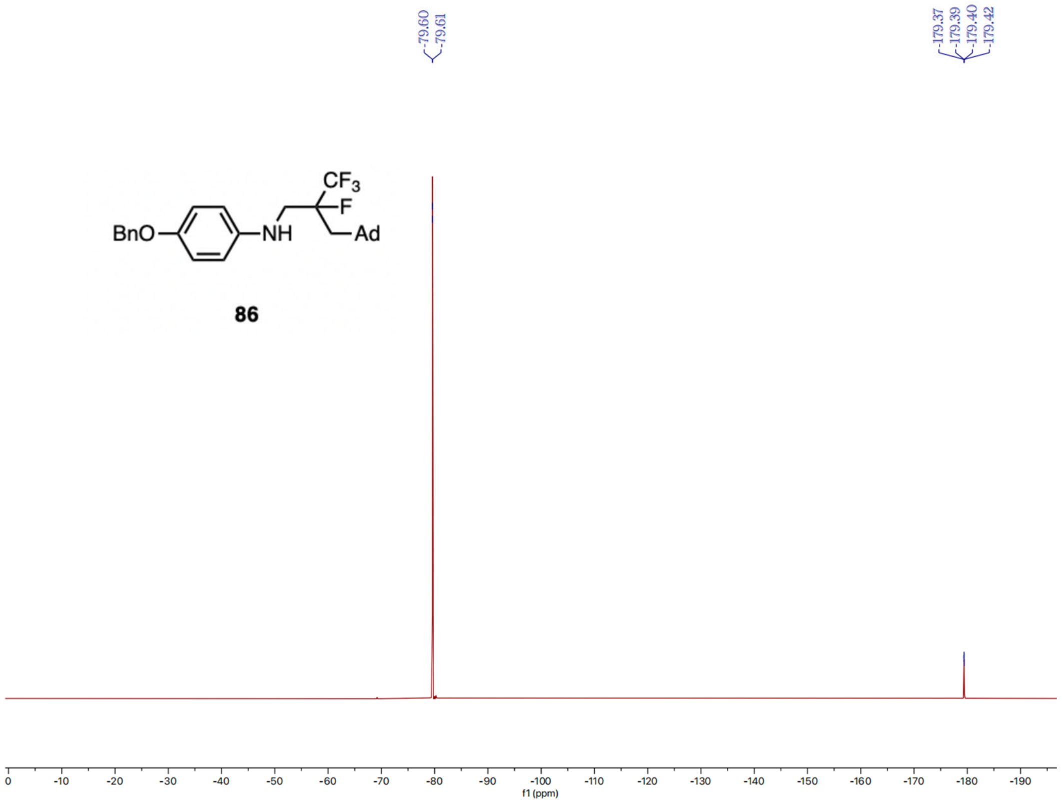

$^{19}$F NMR spectrum (565 MHz, Chloroform-*d*) of compound **86**

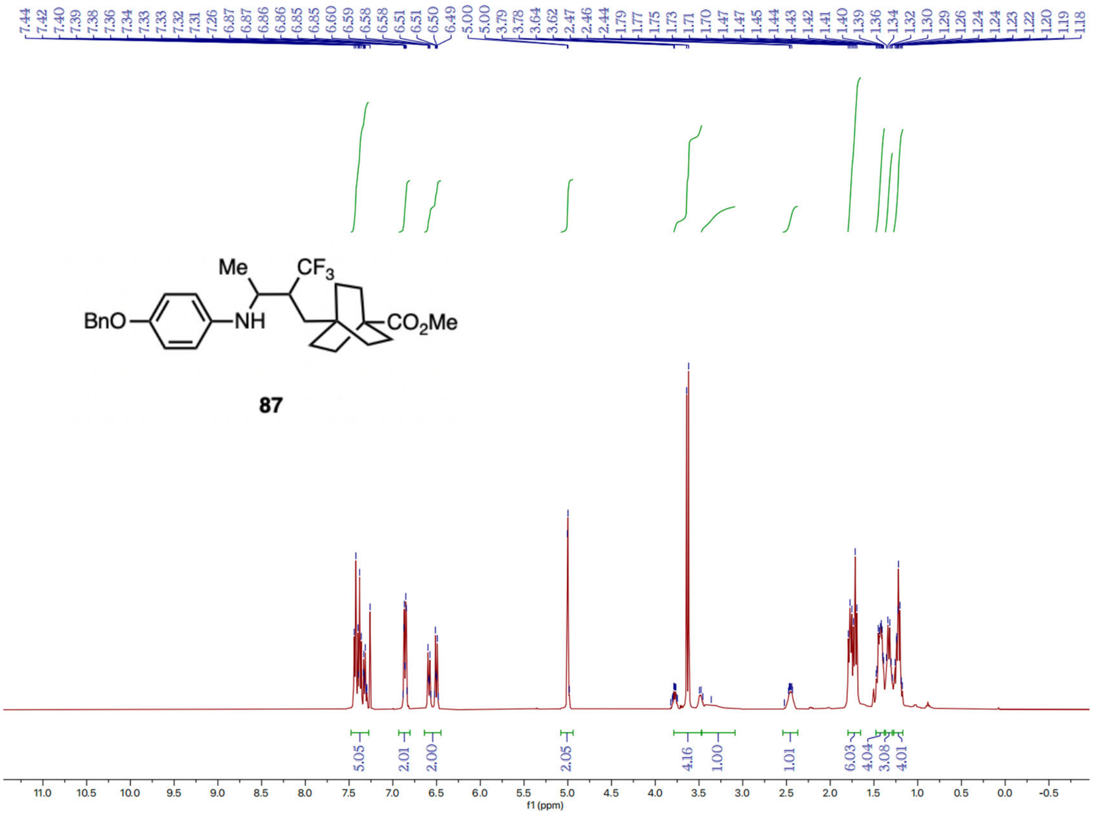


$^{1}$H NMR spectrum (400 MHz, Chloroform-*d*) of compound **87**

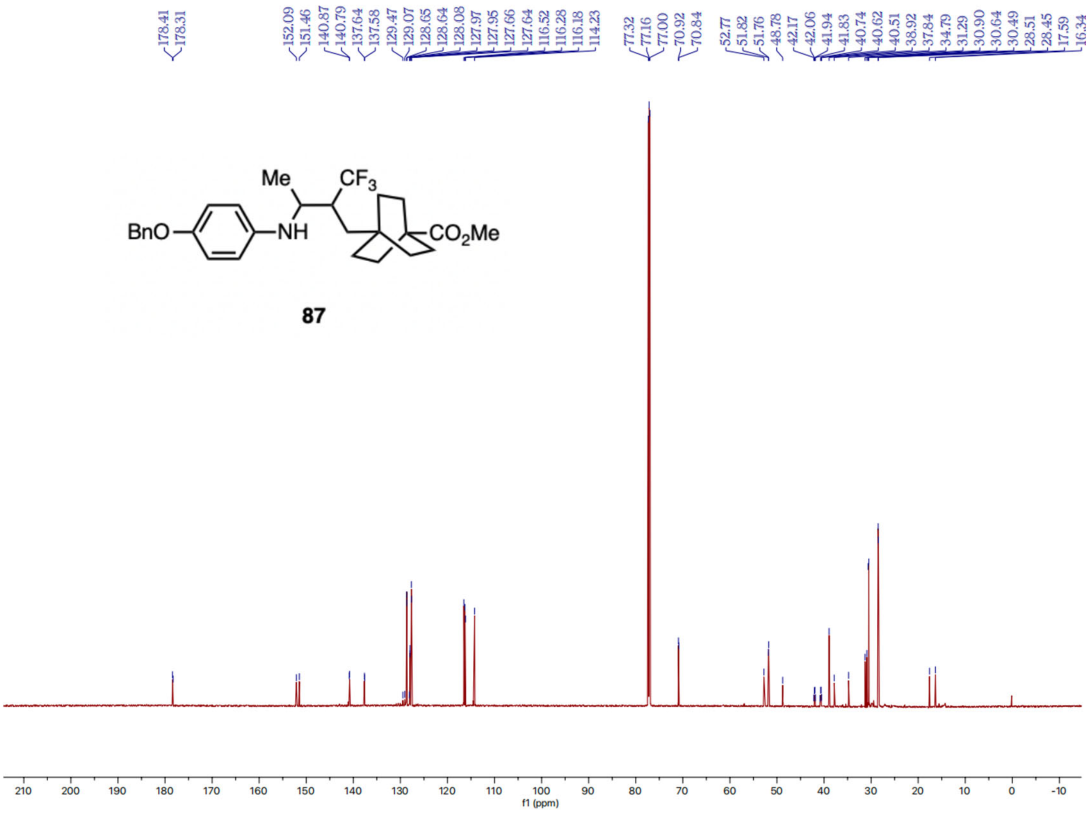


$^{13}$C NMR spectrum (201 MHz, Chloroform-*d*) of compound **87**

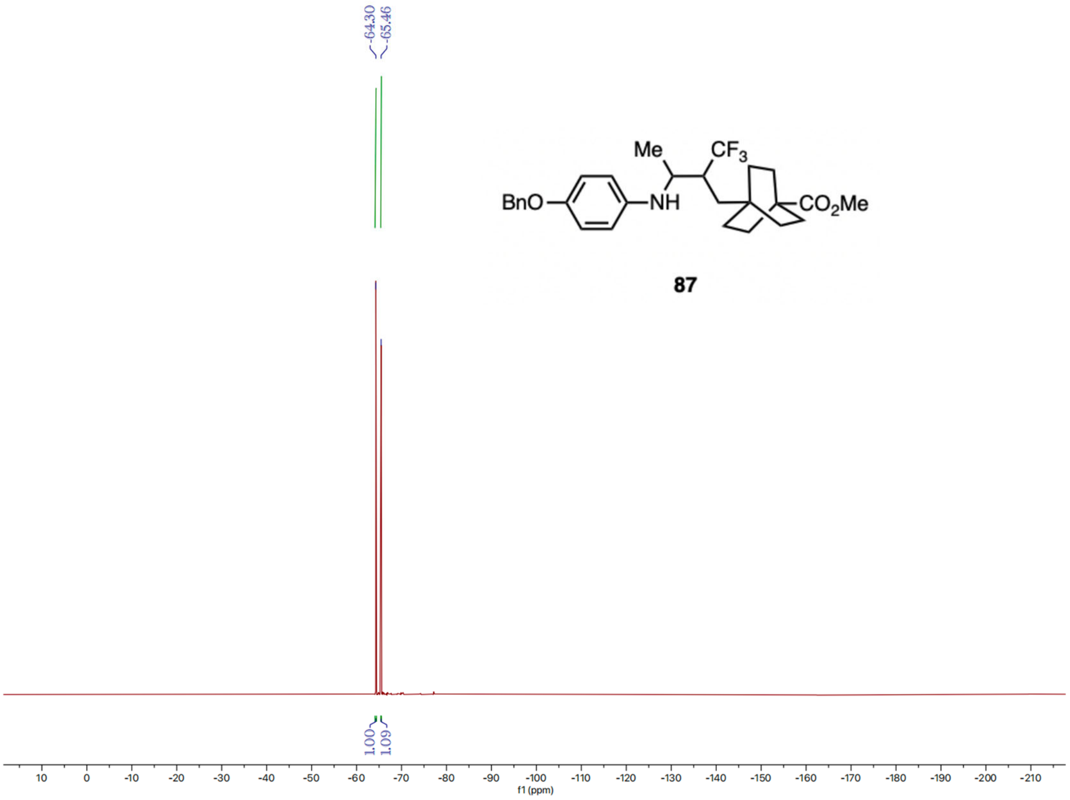


**87**

$^{19}F$ NMR spectrum (376 MHz, Chloroform-*d*) of compound **87**

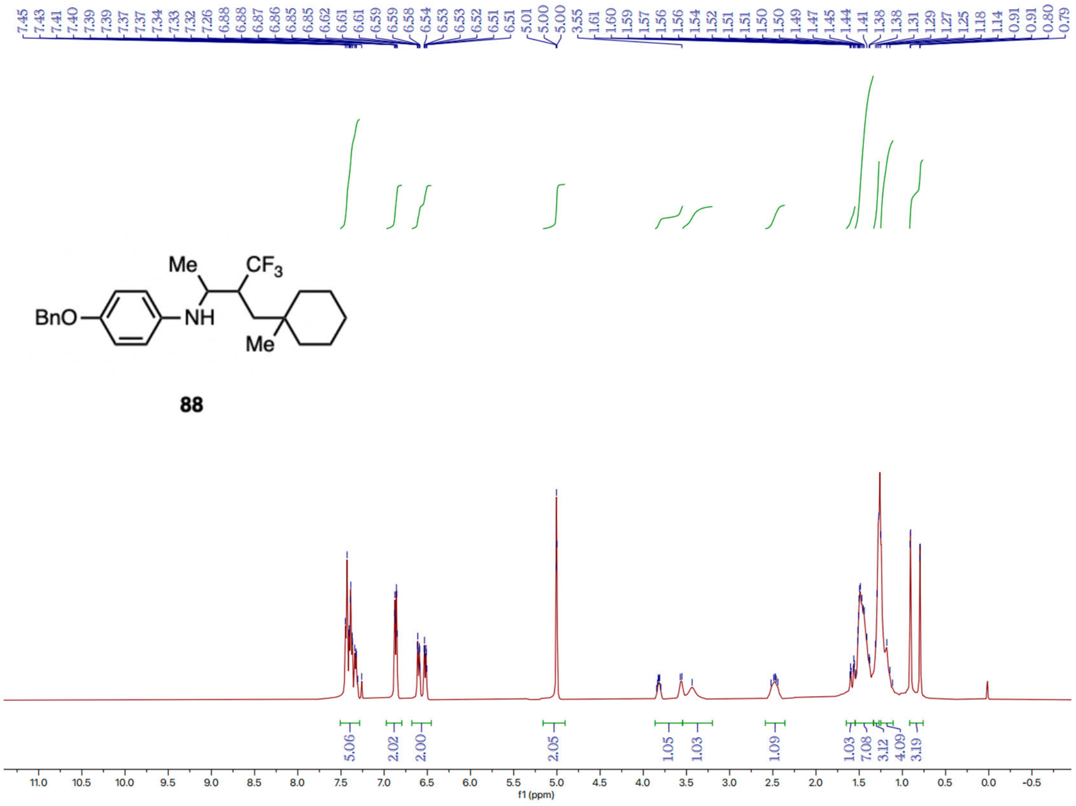


$^{1}$H NMR spectrum (400 MHz, Chloroform-*d*) of compound **88**

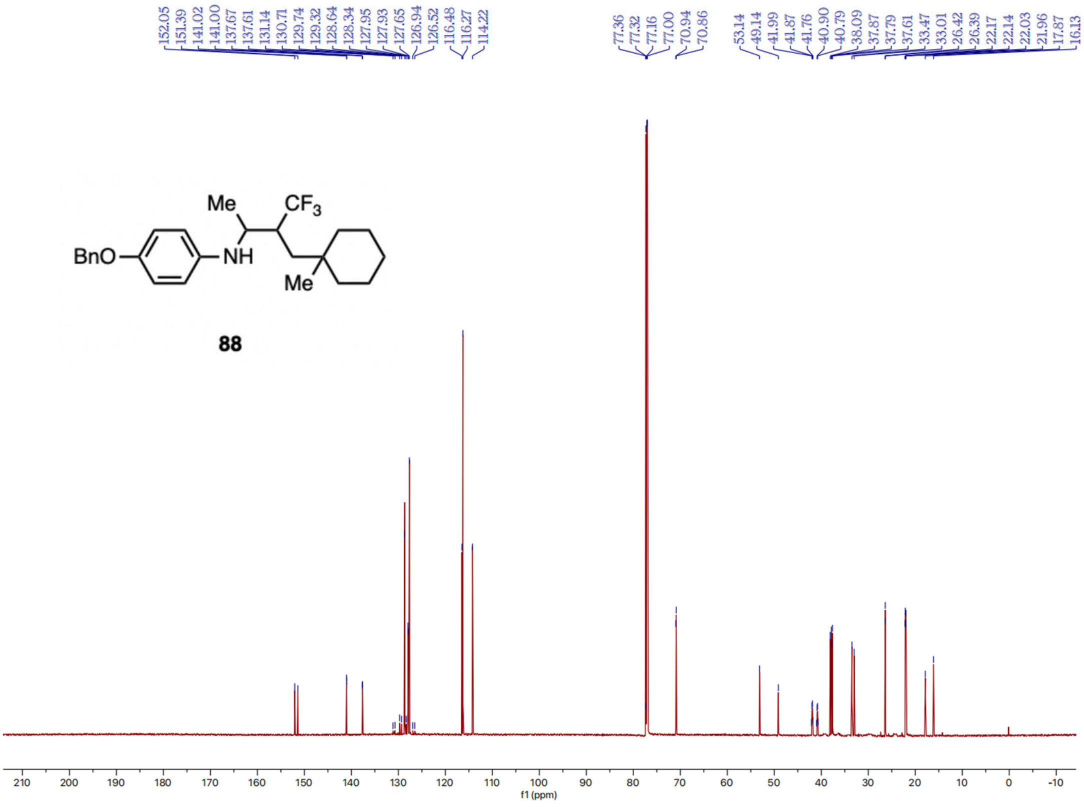


$^{13}$C NMR spectrum (201 MHz, Chloroform-*d*) of compound **88**

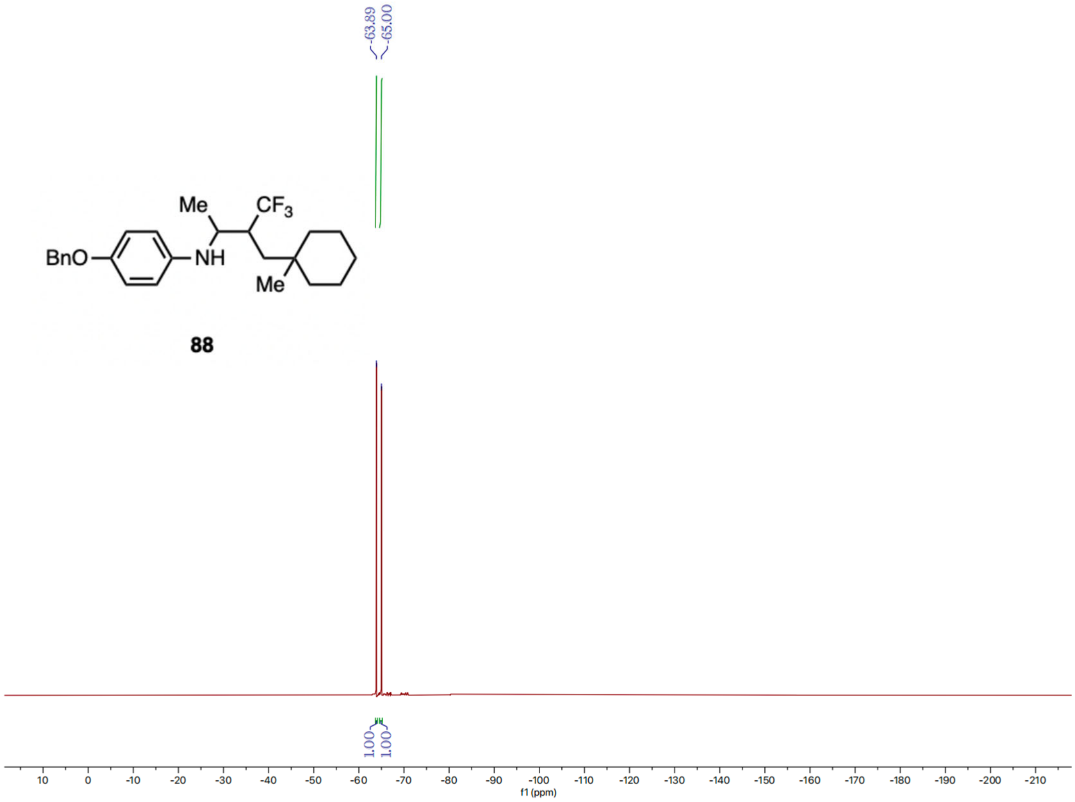


$^{19}$F NMR spectrum (376 MHz, Chloroform-*d*) of compound **88**

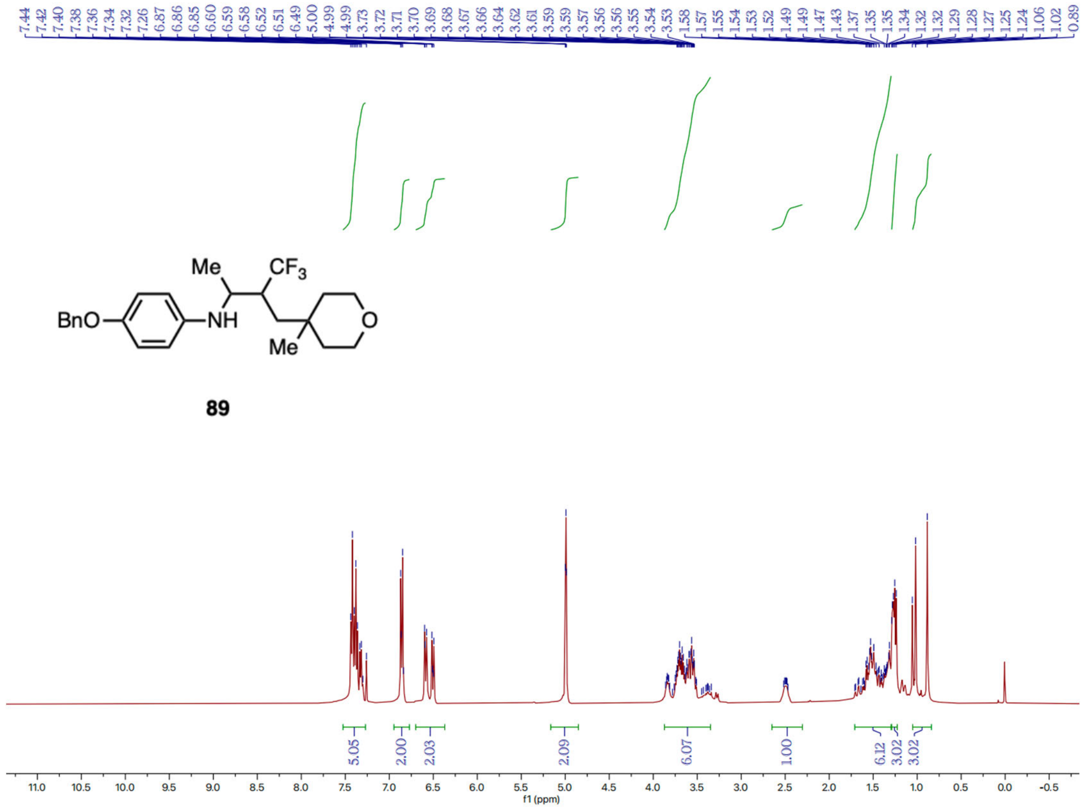


$^{1}$H NMR spectrum (400 MHz, Chloroform-*d*) of compound **89**

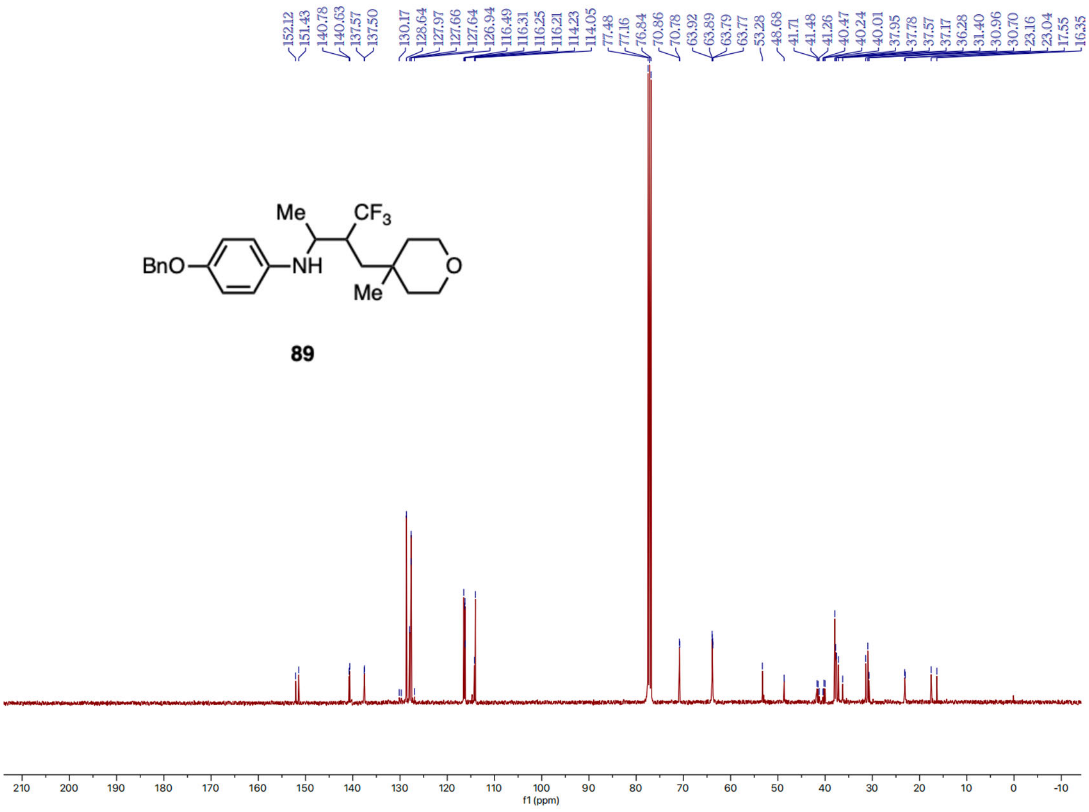


$^{13}$C NMR spectrum (101 MHz, Chloroform-*d*) of compound **89**

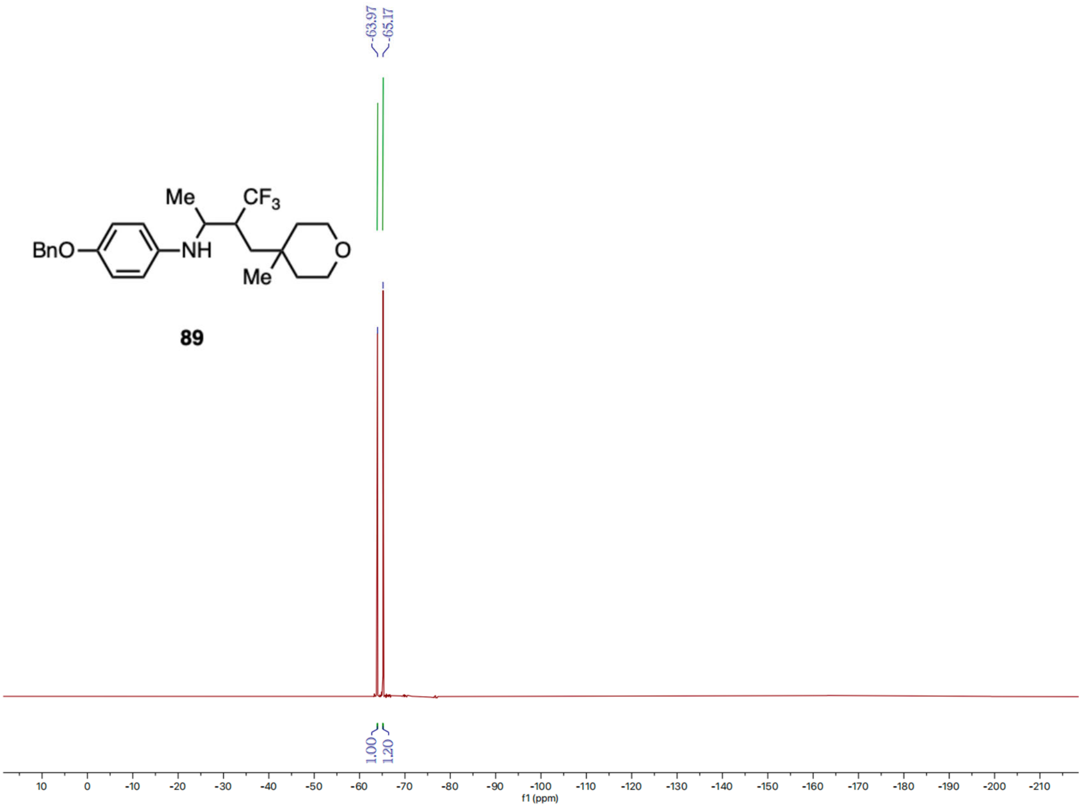


$^{19}$F NMR spectrum (376 MHz, Chloroform-*d*) of compound **89**

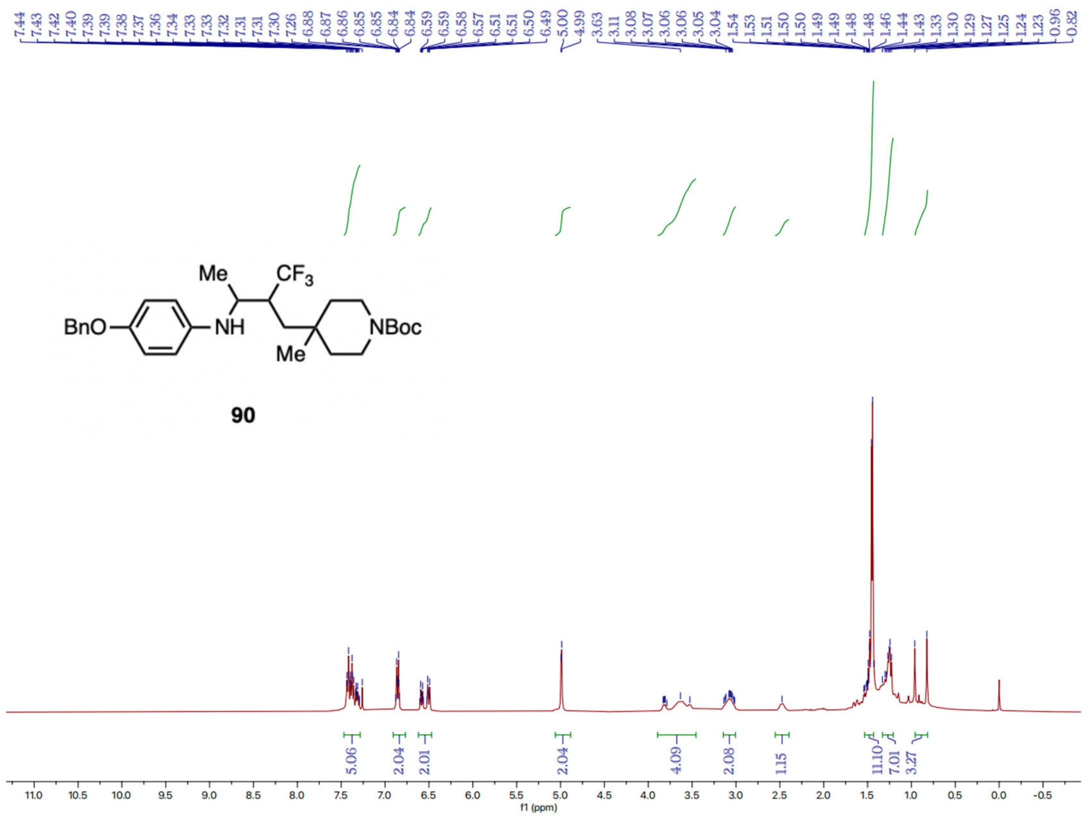

$^{1}$H NMR spectrum (400 MHz, Chloroform-*d*) of compound **90**

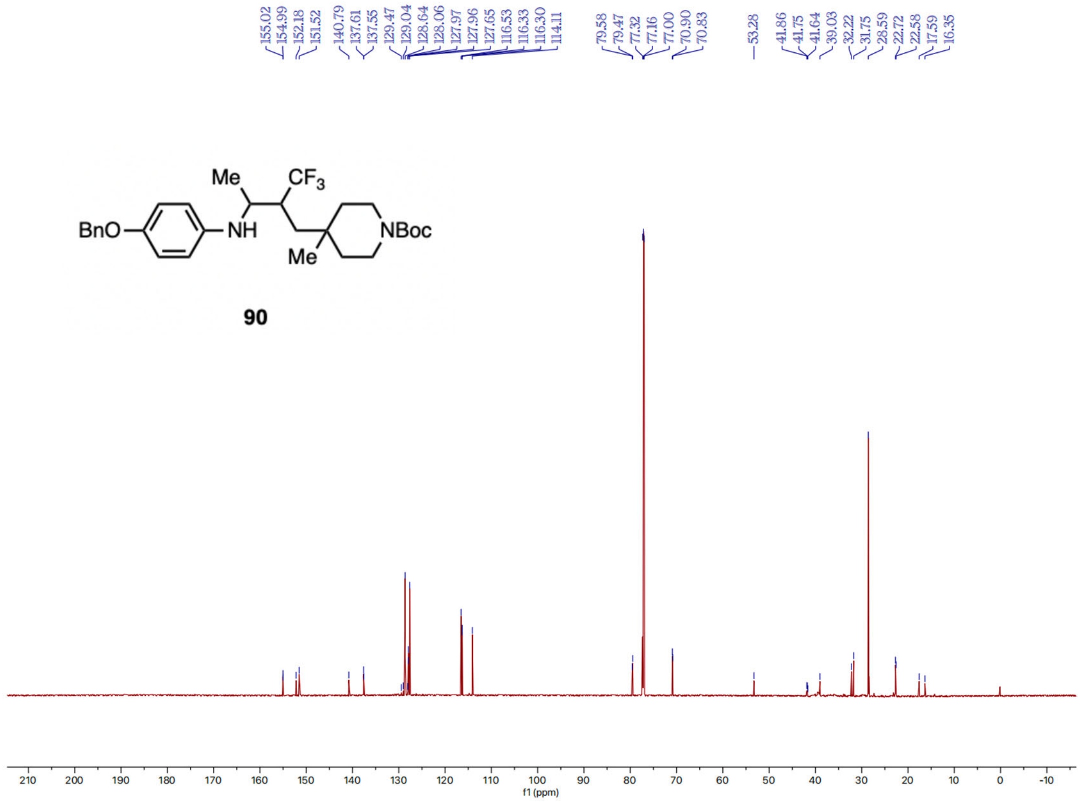

$^{13}$C NMR spectrum (201 MHz, Chloroform-*d*) of compound **90**

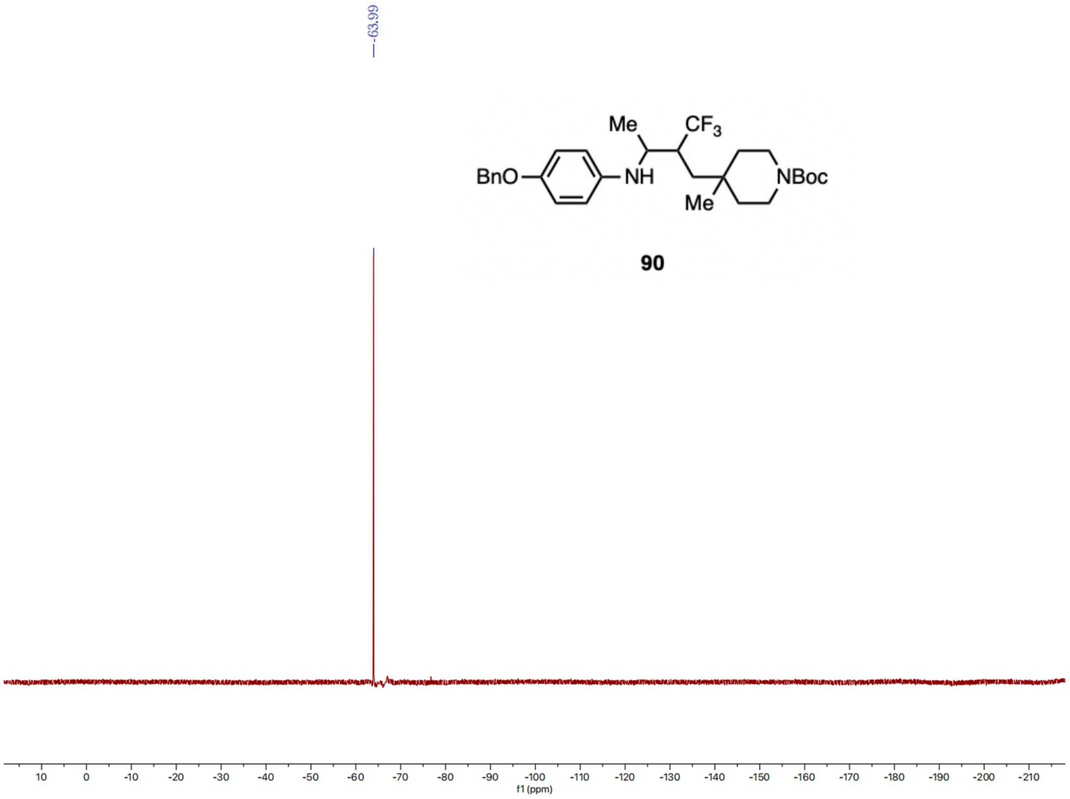

$^{19}$F NMR spectrum (376 MHz, Chloroform-*d*) of compound **90**

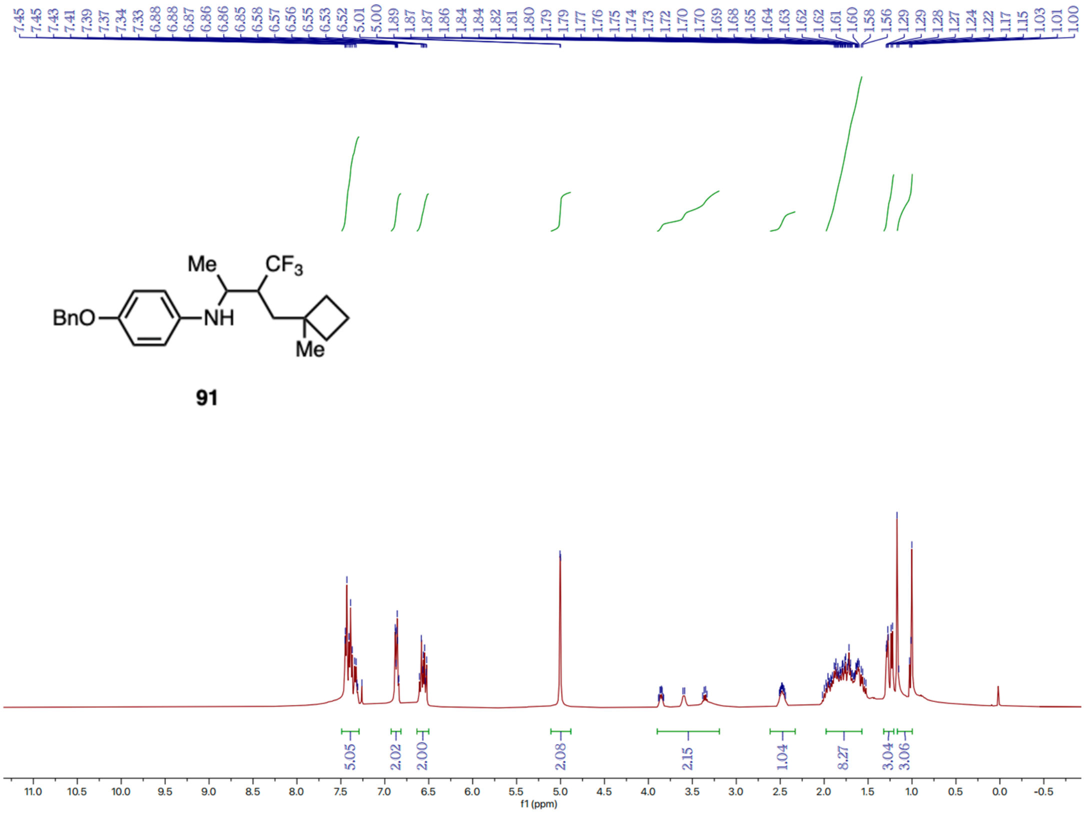

$^{1}H$ NMR spectrum (400 MHz, Chloroform-*d*) of compound **91**

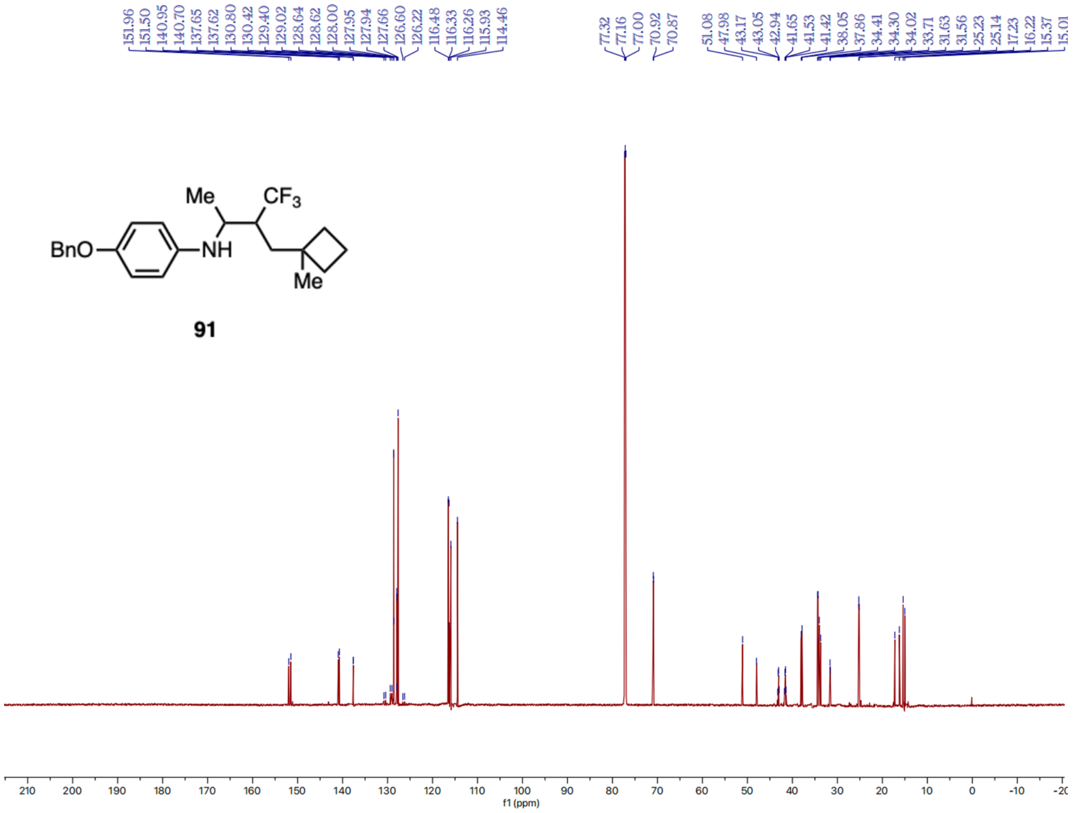

$^{13}C$ NMR spectrum (201 MHz, Chloroform-*d*) of compound **91**

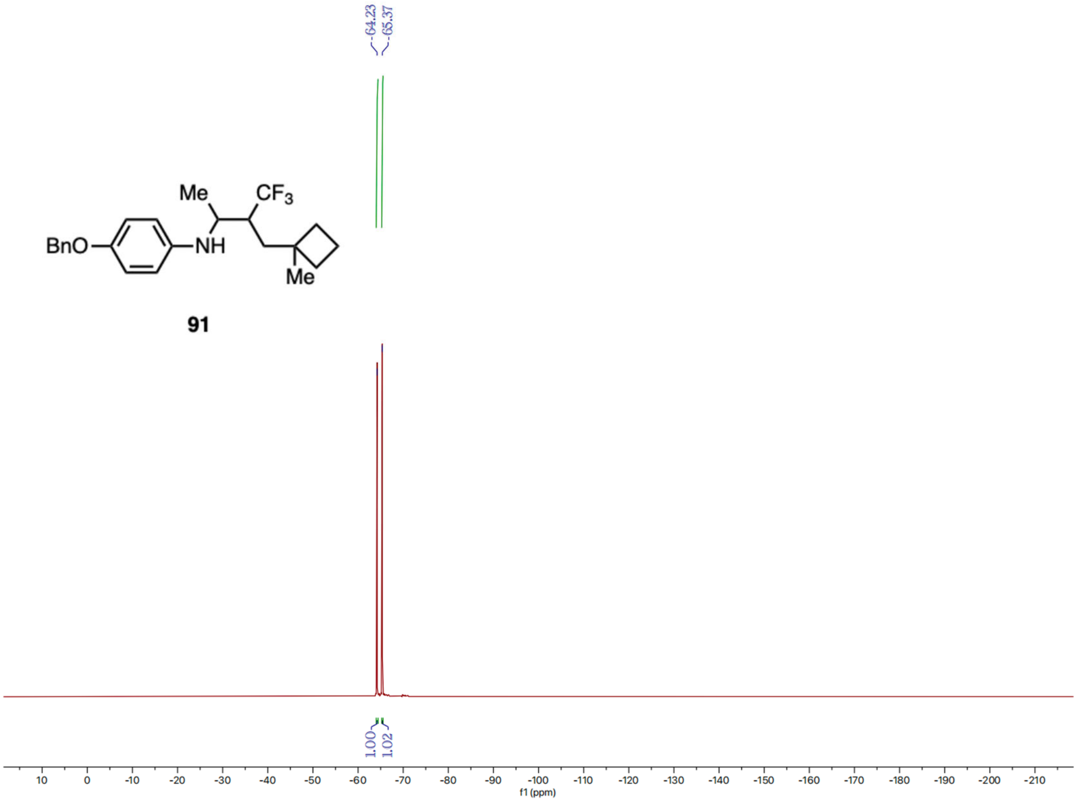


**91**

$^{19}$F NMR spectrum (376 MHz, Chloroform-*d*) of compound **91**

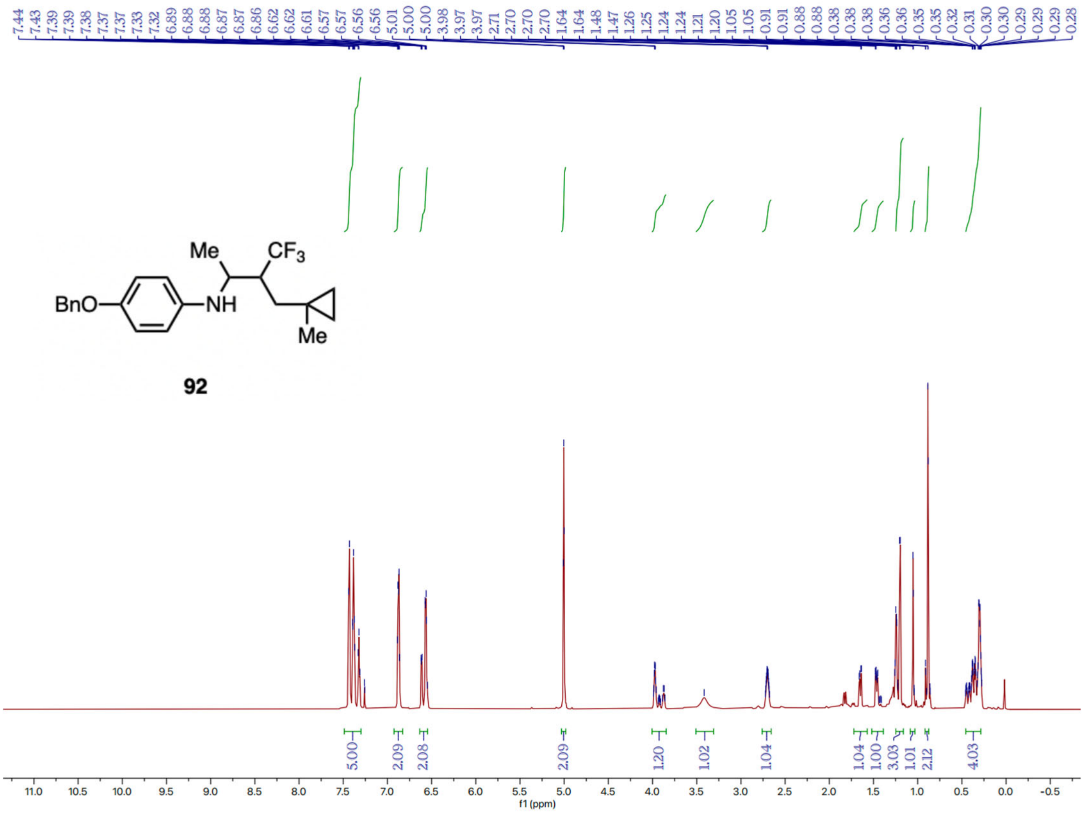


$^{1}$H NMR spectrum (800 MHz, Chloroform-*d*) of compound **92**

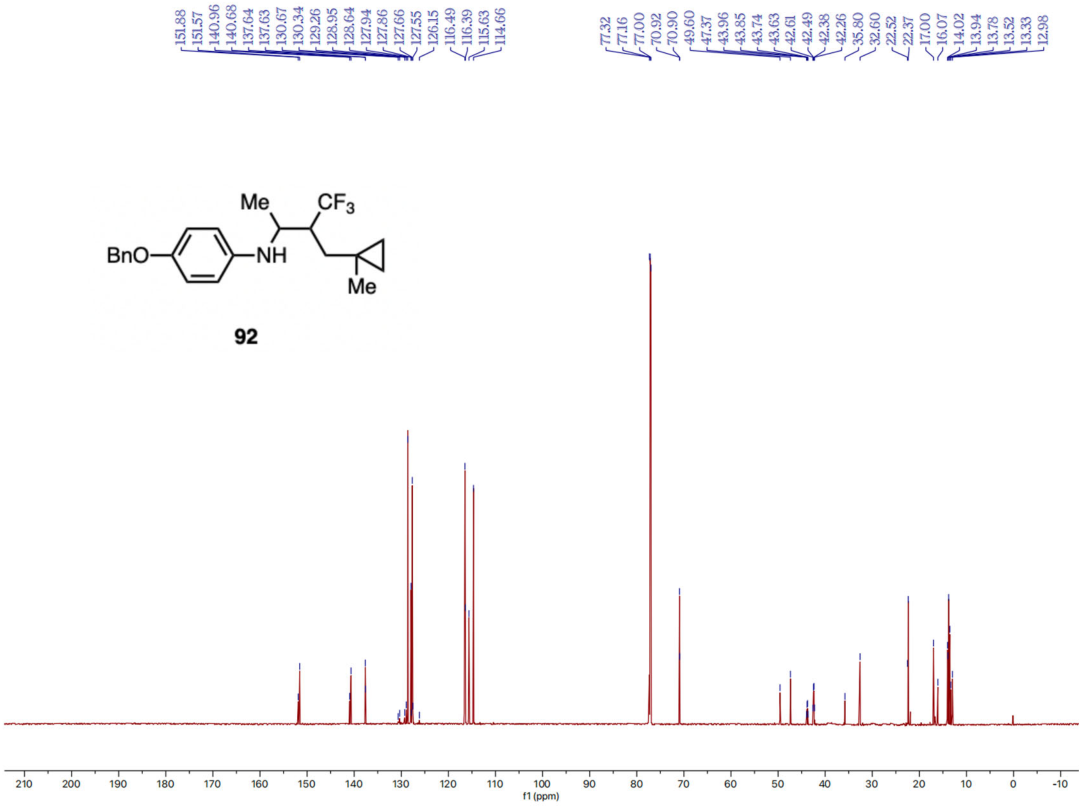


$^{13}$C NMR spectrum (201 MHz, Chloroform-*d*) of compound **92**

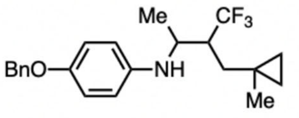

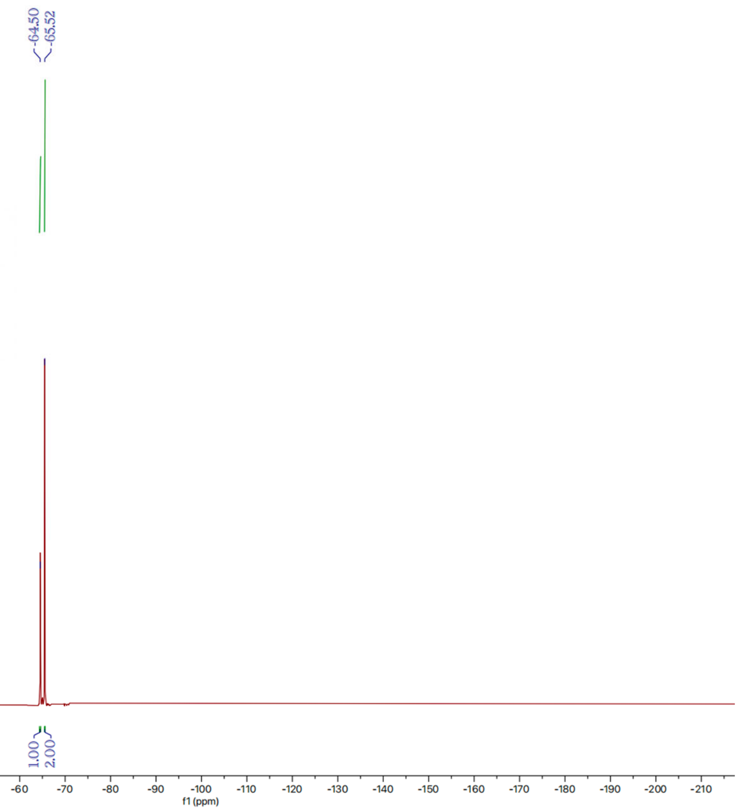


**92**

$^{19}$F NMR spectrum (376 MHz, Chloroform-*d*) of compound **92**

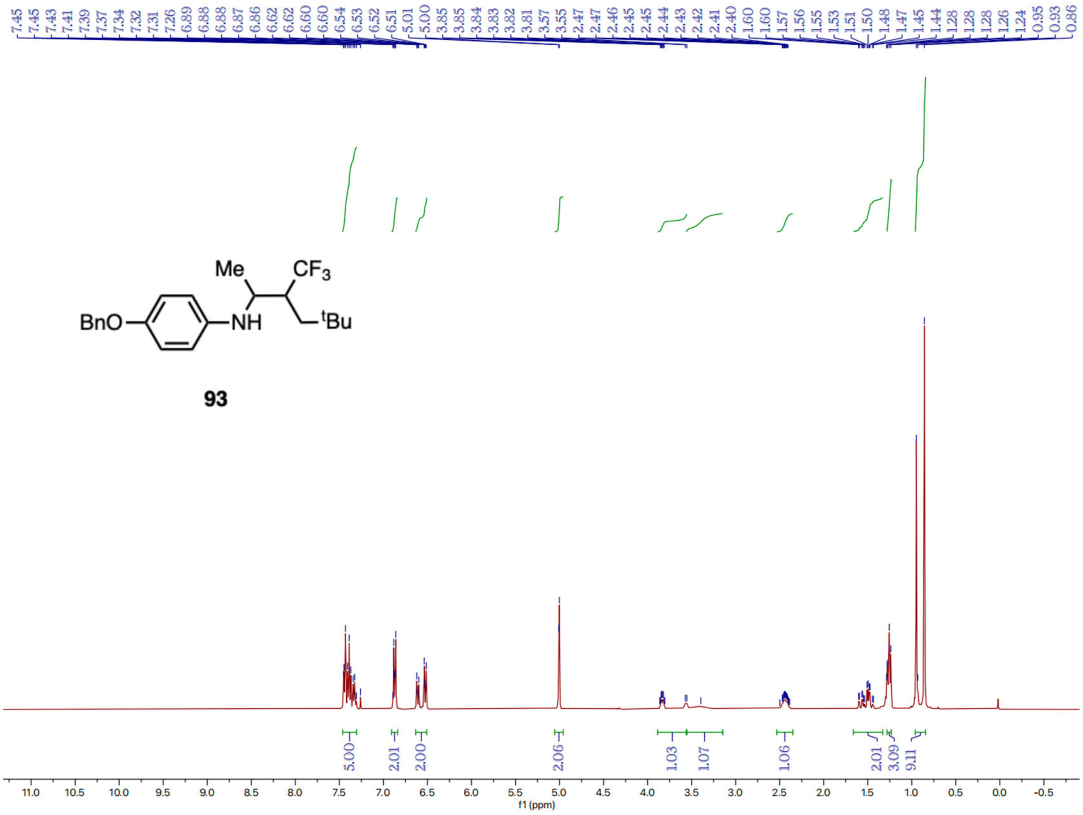

$^{1}$H NMR spectrum (400 MHz, Chloroform-*d*) of compound **93**

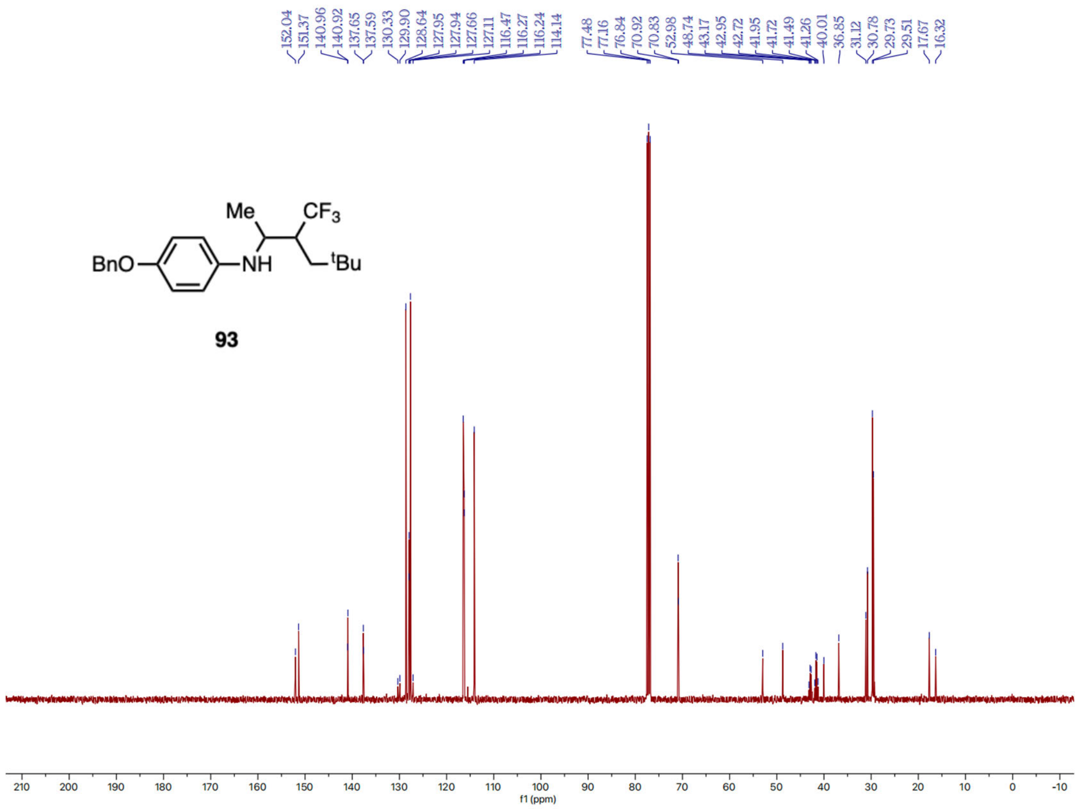

$^{13}$C NMR spectrum (101 MHz, Chloroform-*d*) of compound **93**

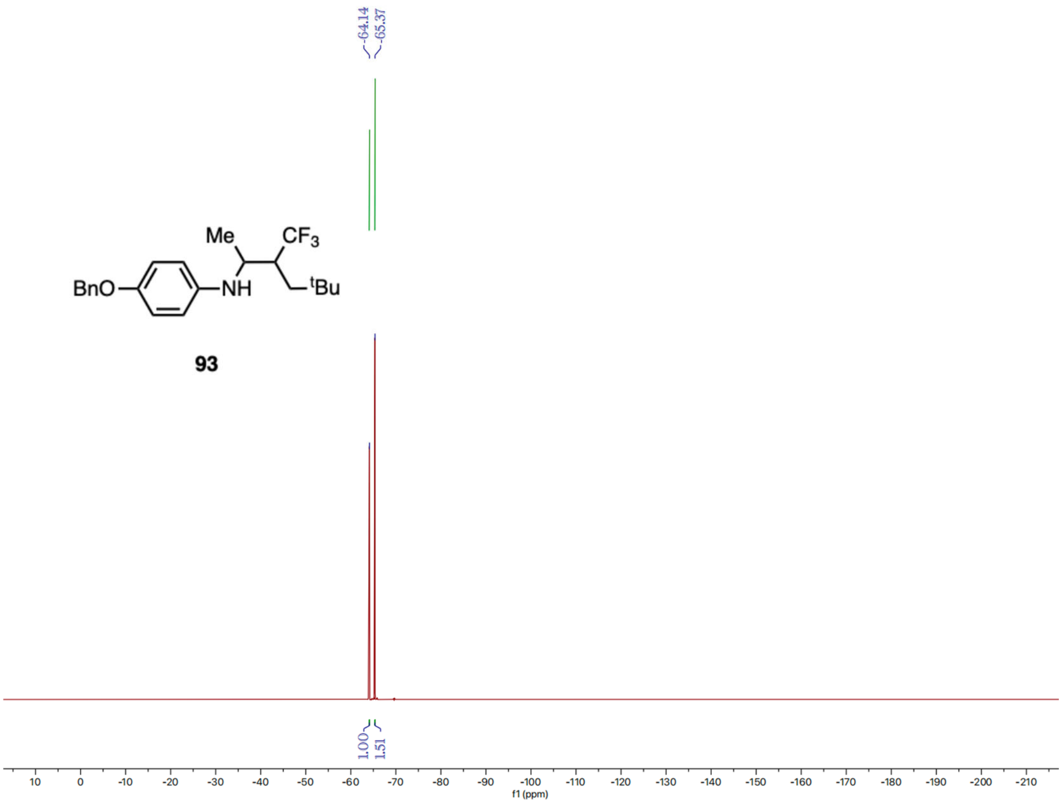


$^{19}$F NMR spectrum (376 MHz, Chloroform-*d*) of compound **93**

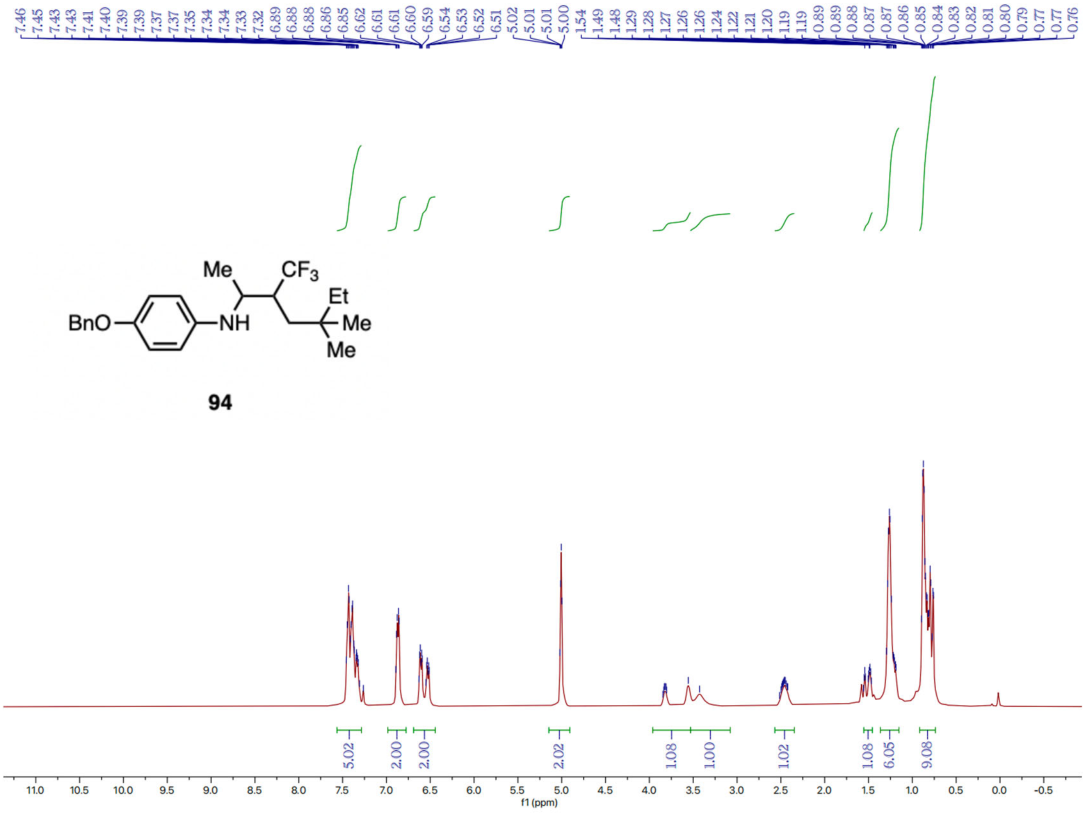


$^{1}$H NMR spectrum (400 MHz, Chloroform-*d*) of compound **94**

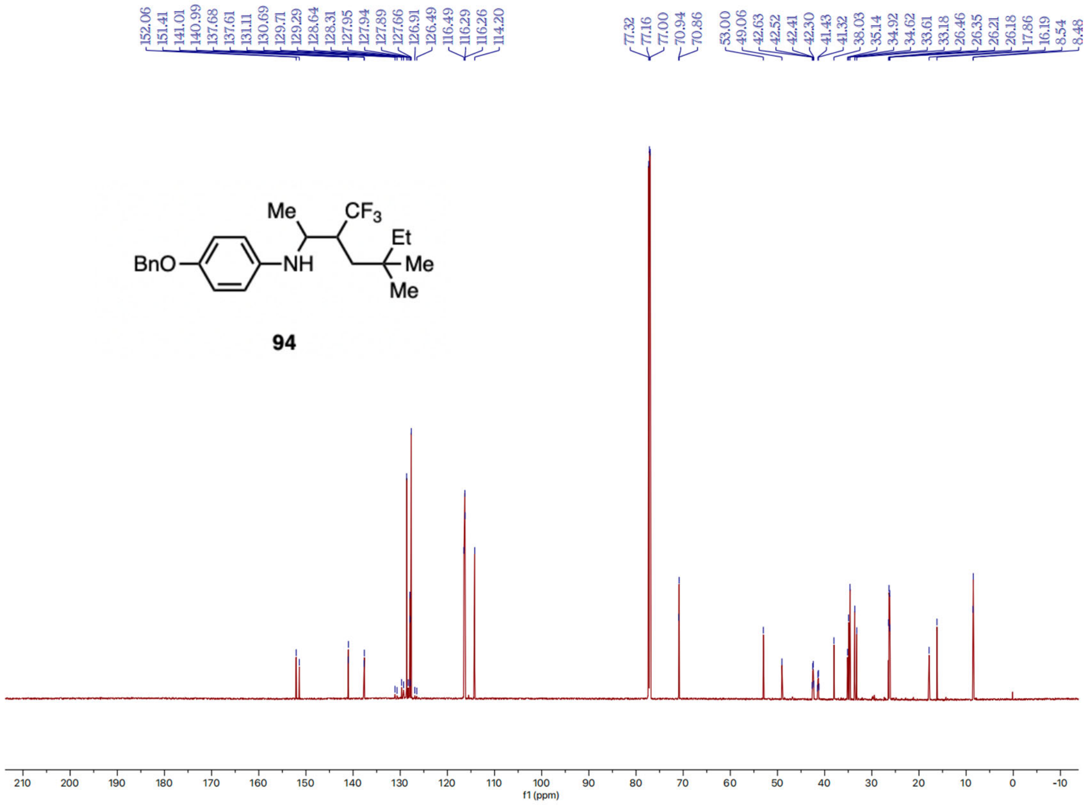


$^{13}$C NMR spectrum (201 MHz, Chloroform-*d*) of compound **94**

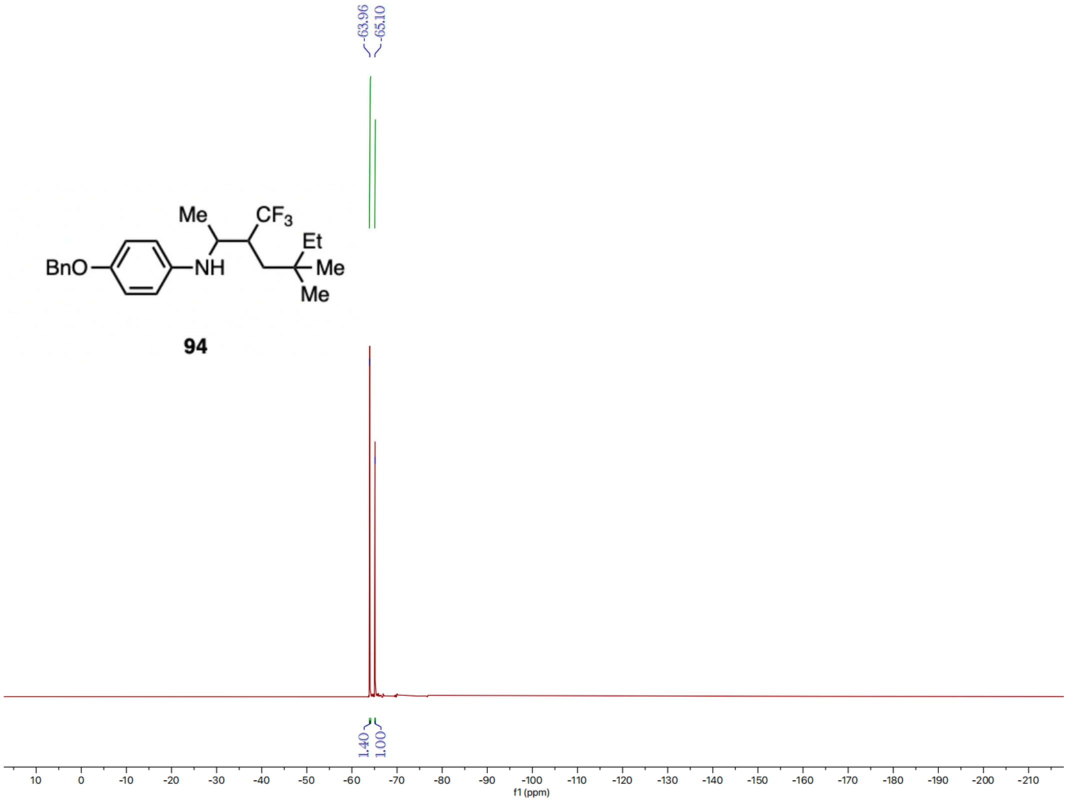


$^{19}F$ NMR spectrum (376 MHz, Chloroform-*d*) of compound **94**

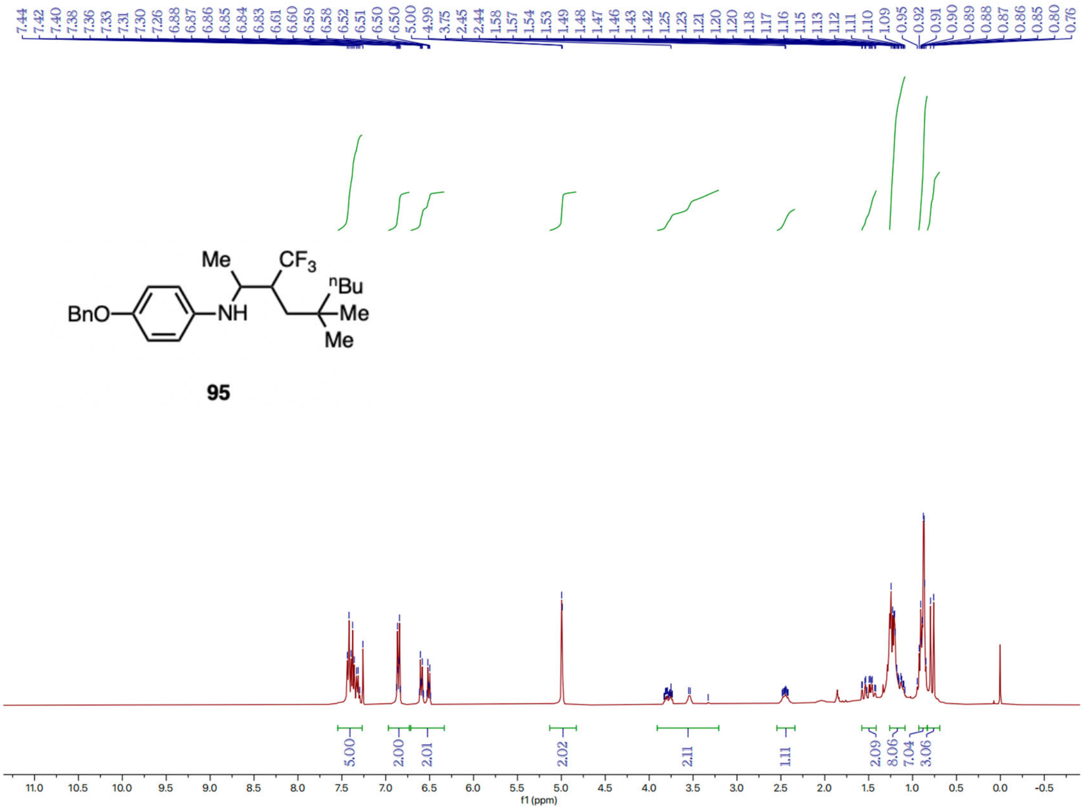

$^{1}$H NMR spectrum (400 MHz, Chloroform-*d*) of compound **95**

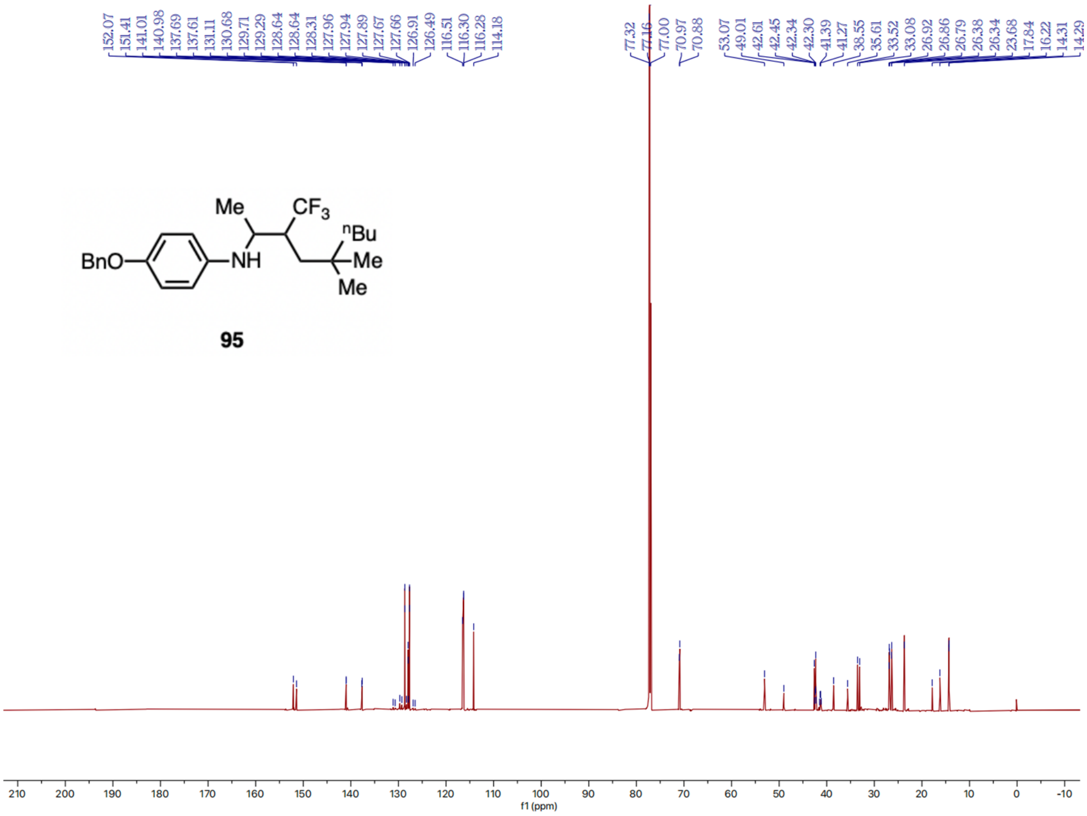

$^{13}$C NMR spectrum (201 MHz, Chloroform-*d*) of compound **95**

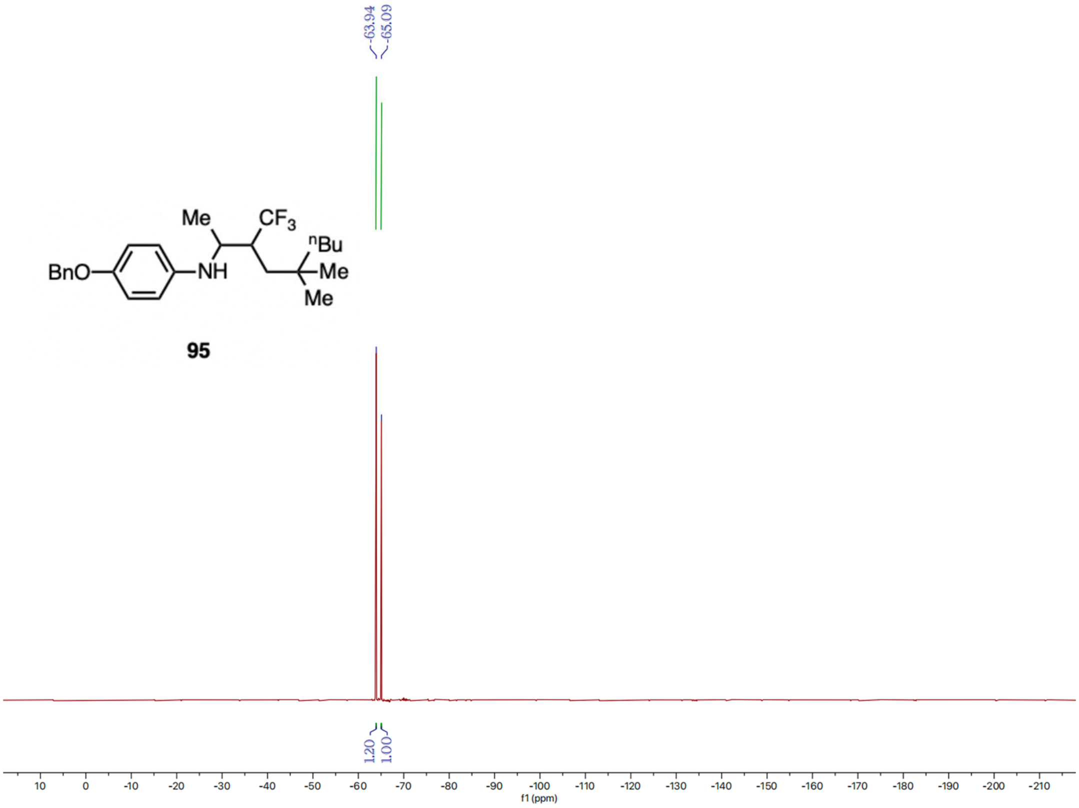


$^{19}$F NMR spectrum (376 MHz, Chloroform-*d*) of compound **95**

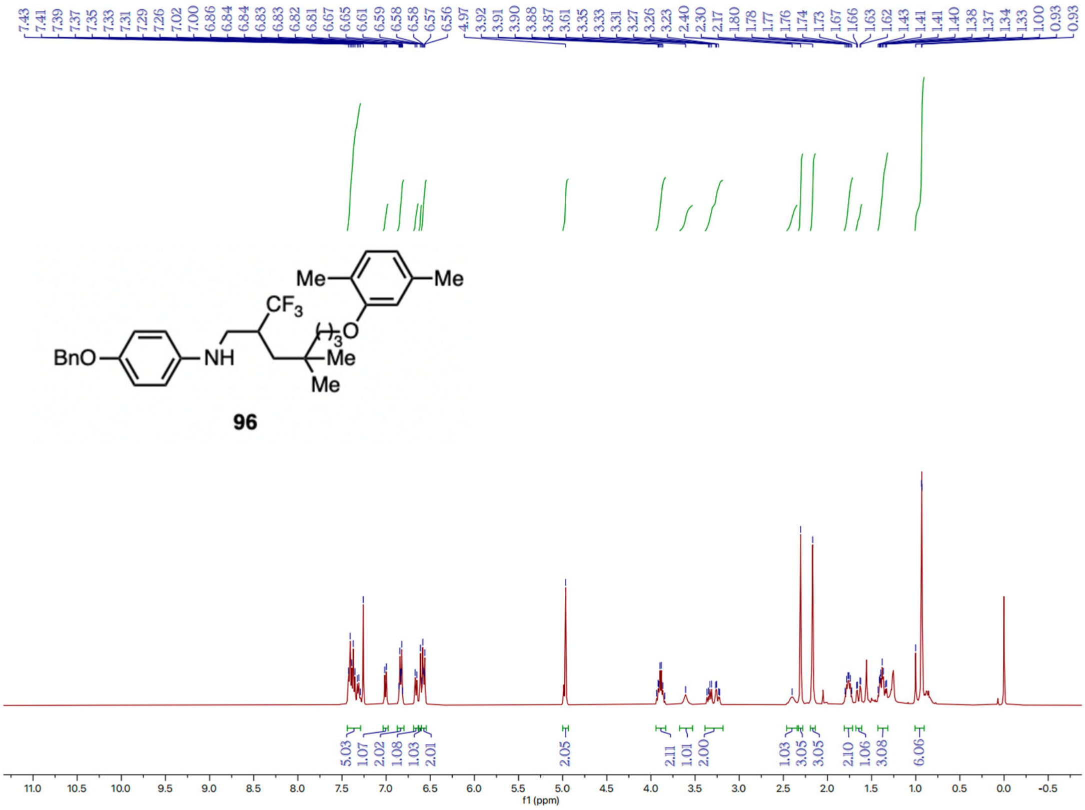

$^{1}$H NMR spectrum (400 MHz, Chloroform-*d*) of compound **96**

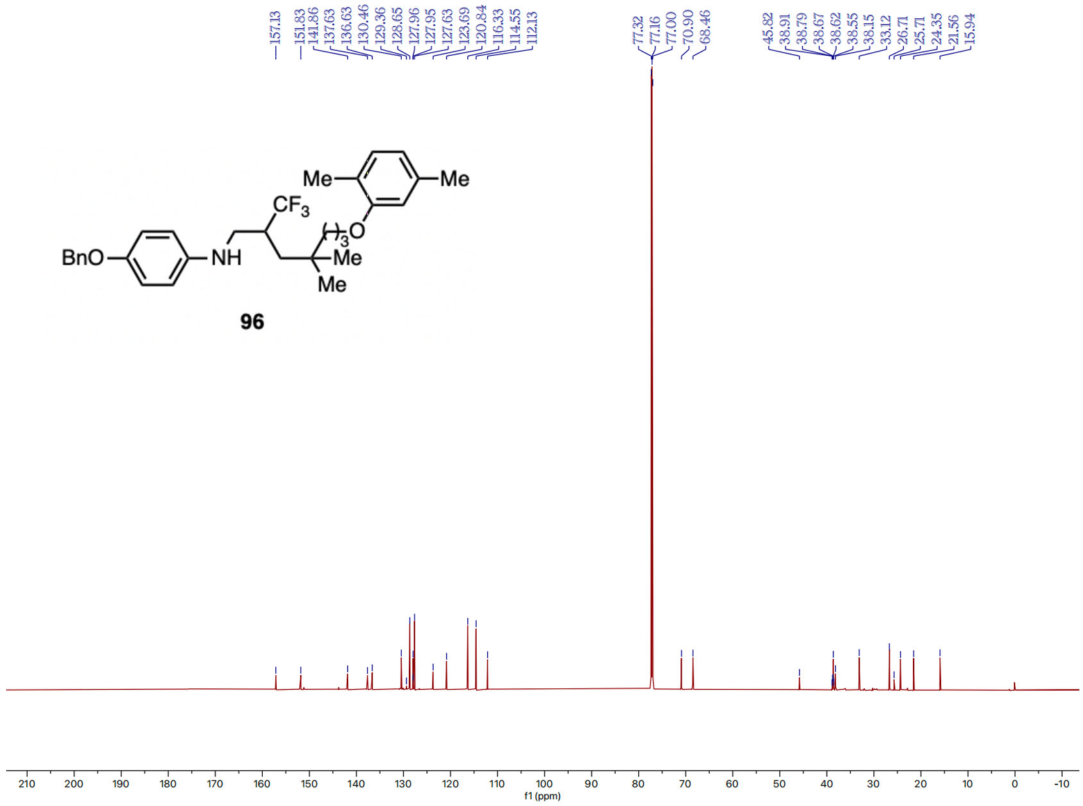

$^{13}$C NMR spectrum (201 MHz, Chloroform-*d*) of compound **96**

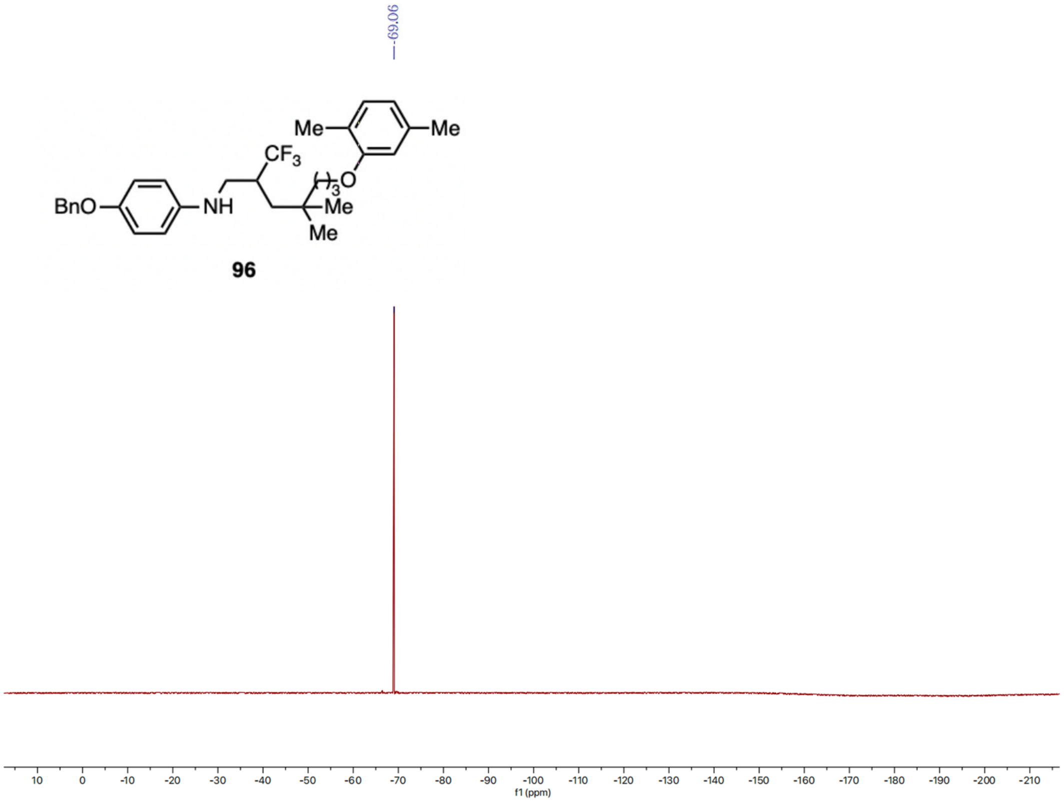


$^{19}$F NMR spectrum (376 MHz, Chloroform-*d*) of compound **96**

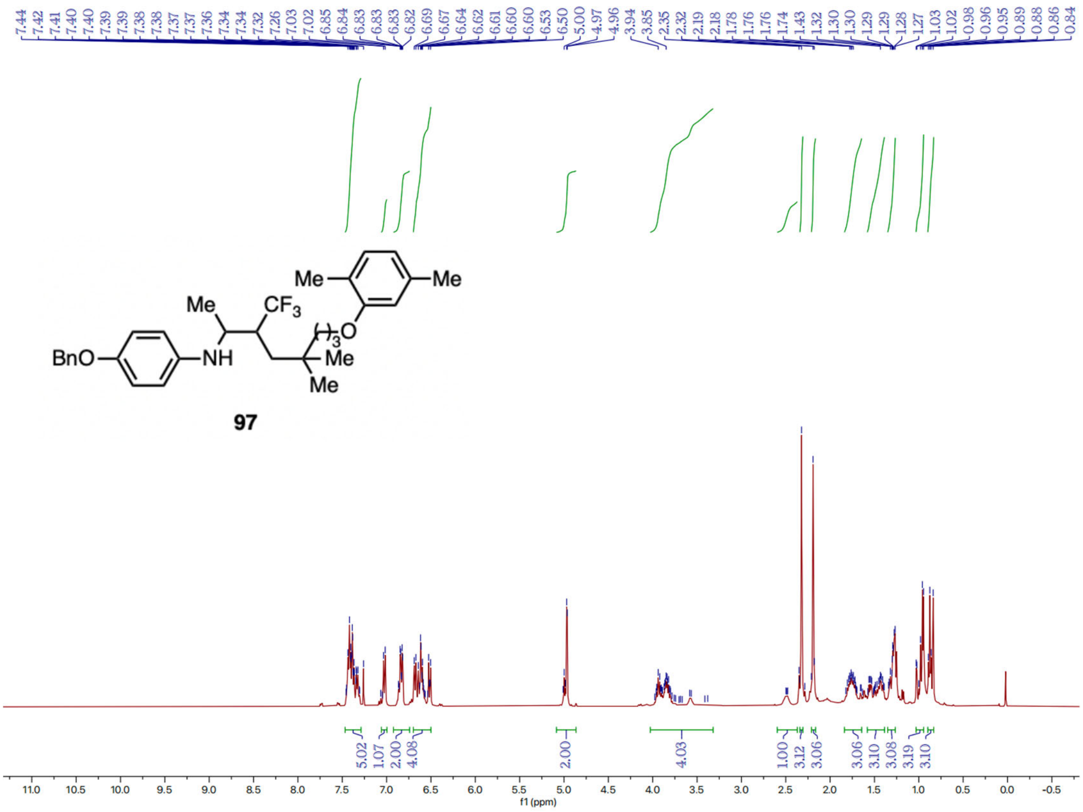


$^{1}$H NMR spectrum (400 MHz, Chloroform-*d*) of compound **97**

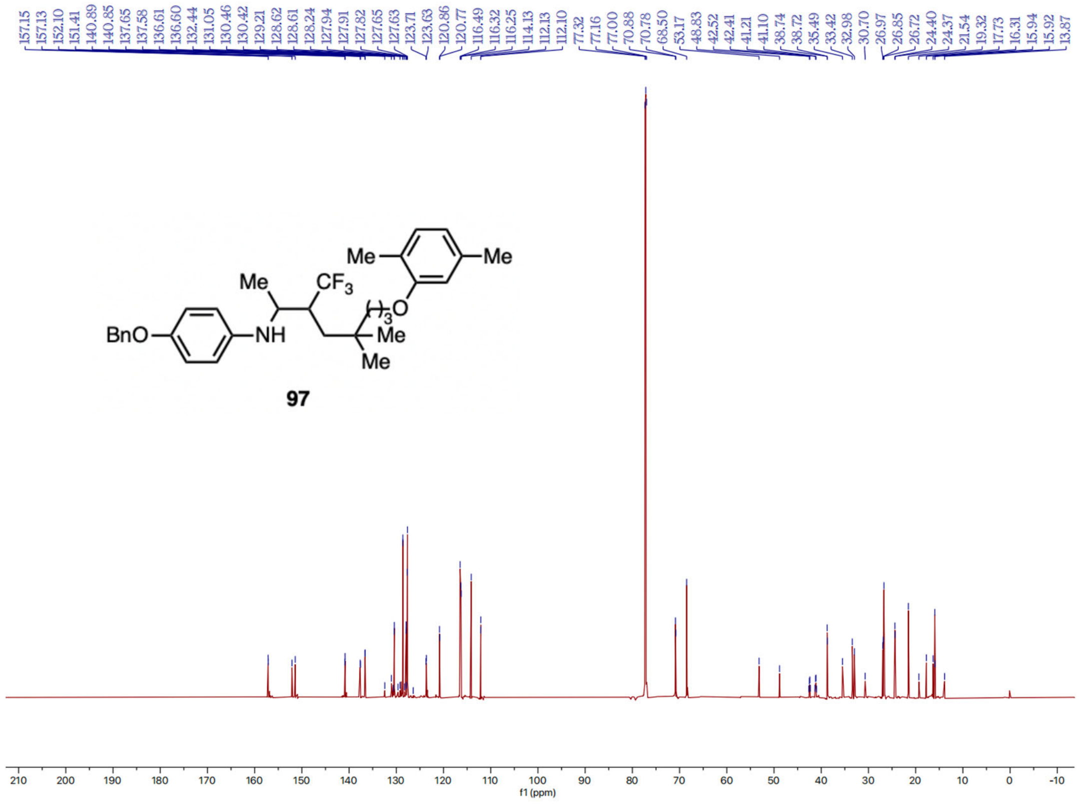


$^{13}$C NMR spectrum (201 MHz, Chloroform-*d*) of compound **97**

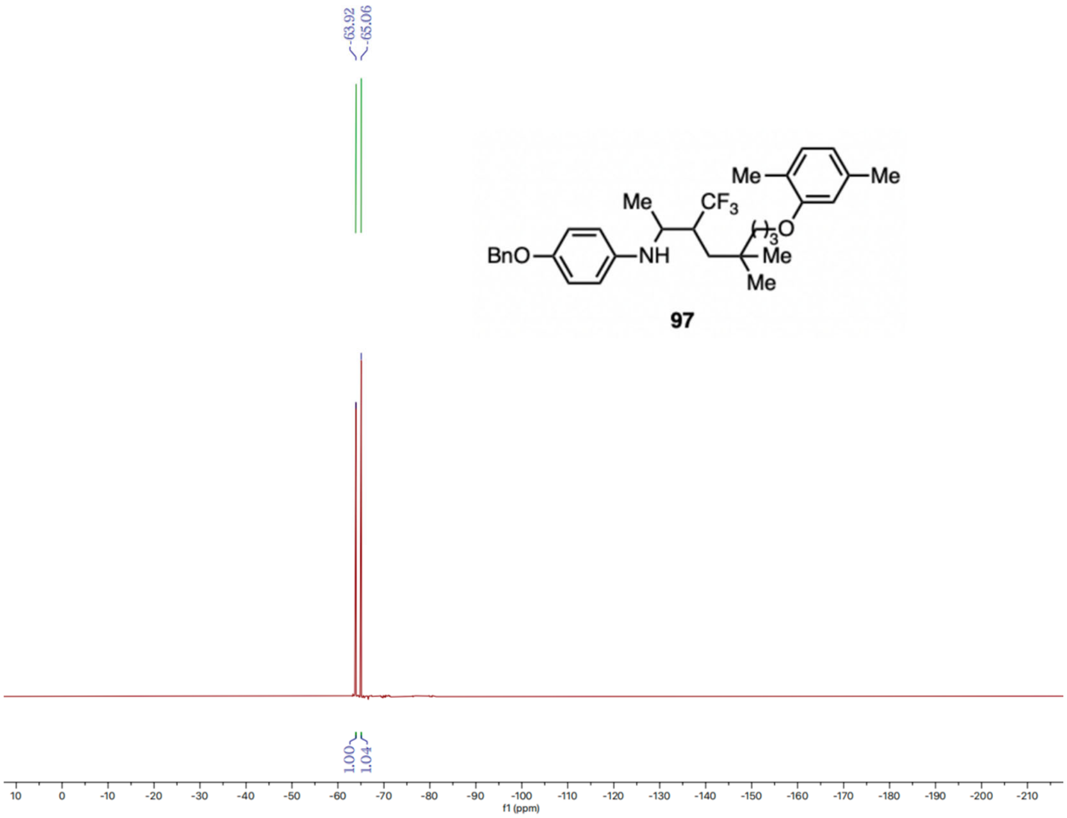


$^{19}$F NMR spectrum (376 MHz, Chloroform-*d*) of compound **97**

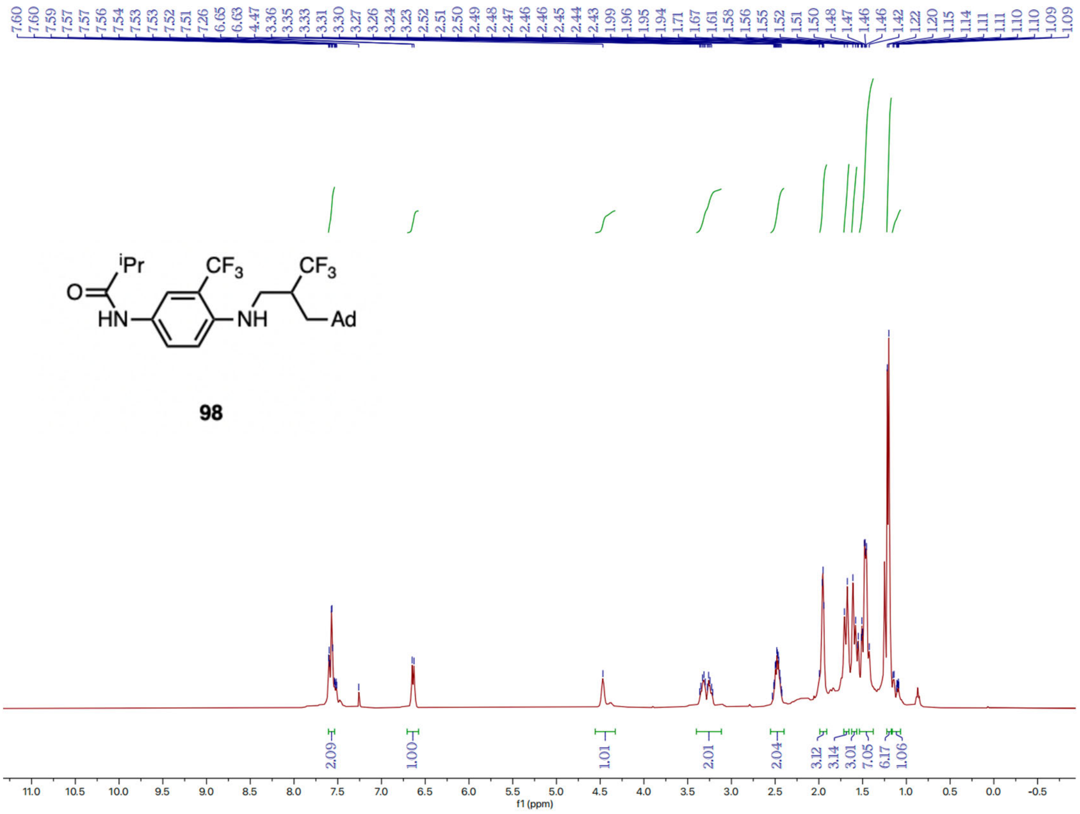

$^{1}$H NMR spectrum (400 MHz, Chloroform-*d*) of compound **98**

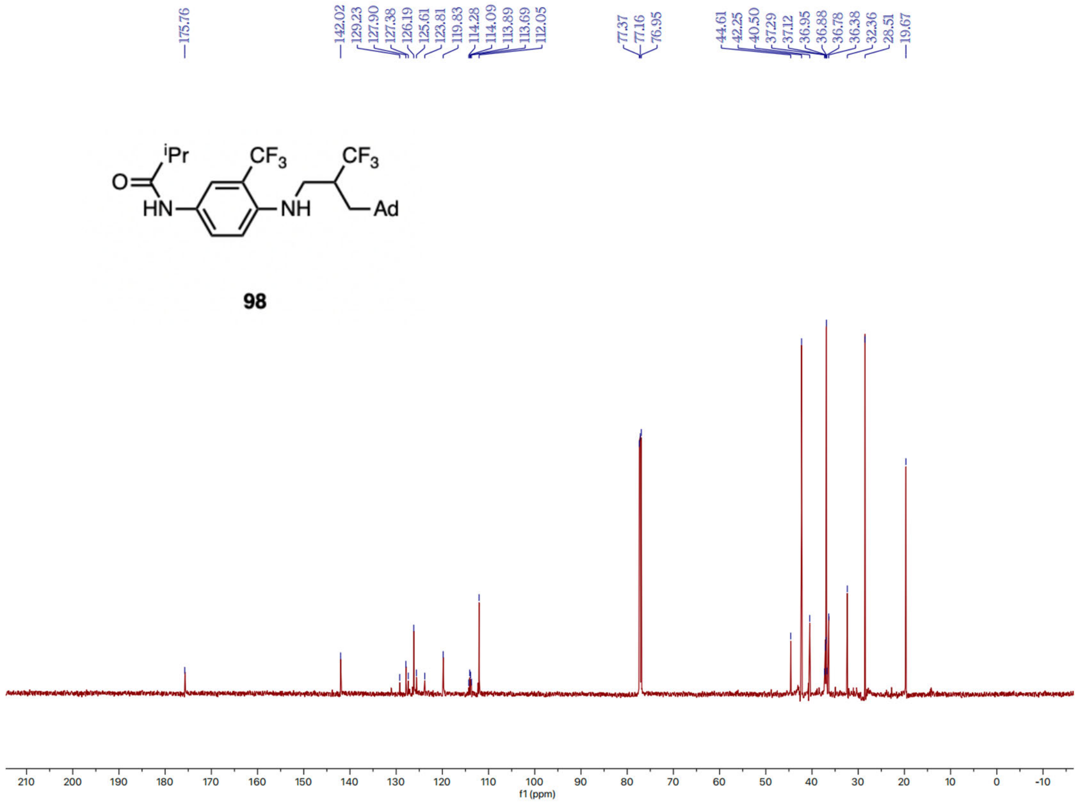

$^{13}$C NMR spectrum (151 MHz, Chloroform-*d*) of compound **98**

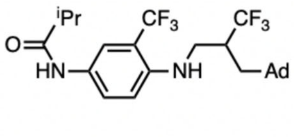


**98**

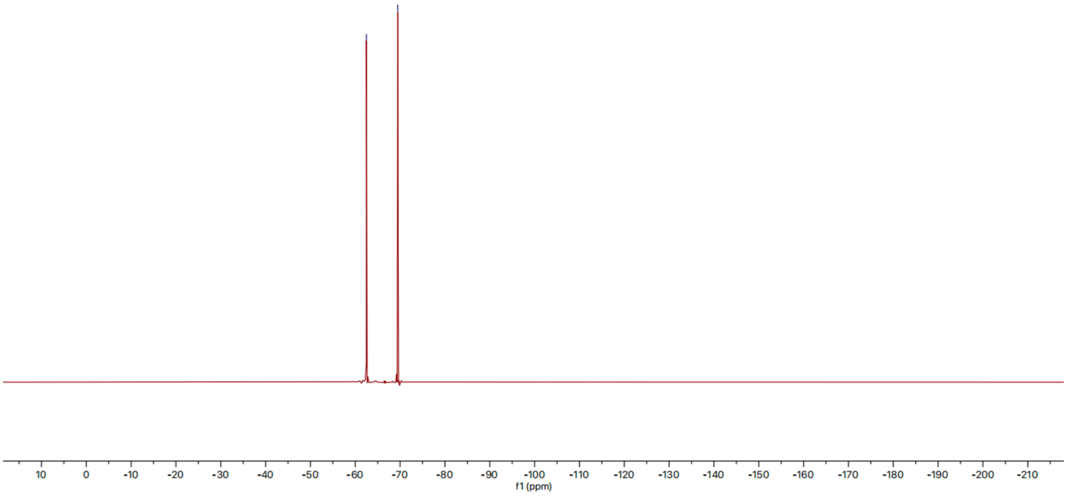


$^{19}$F NMR spectrum (376 MHz, Chloroform-*d*) of compound **98**

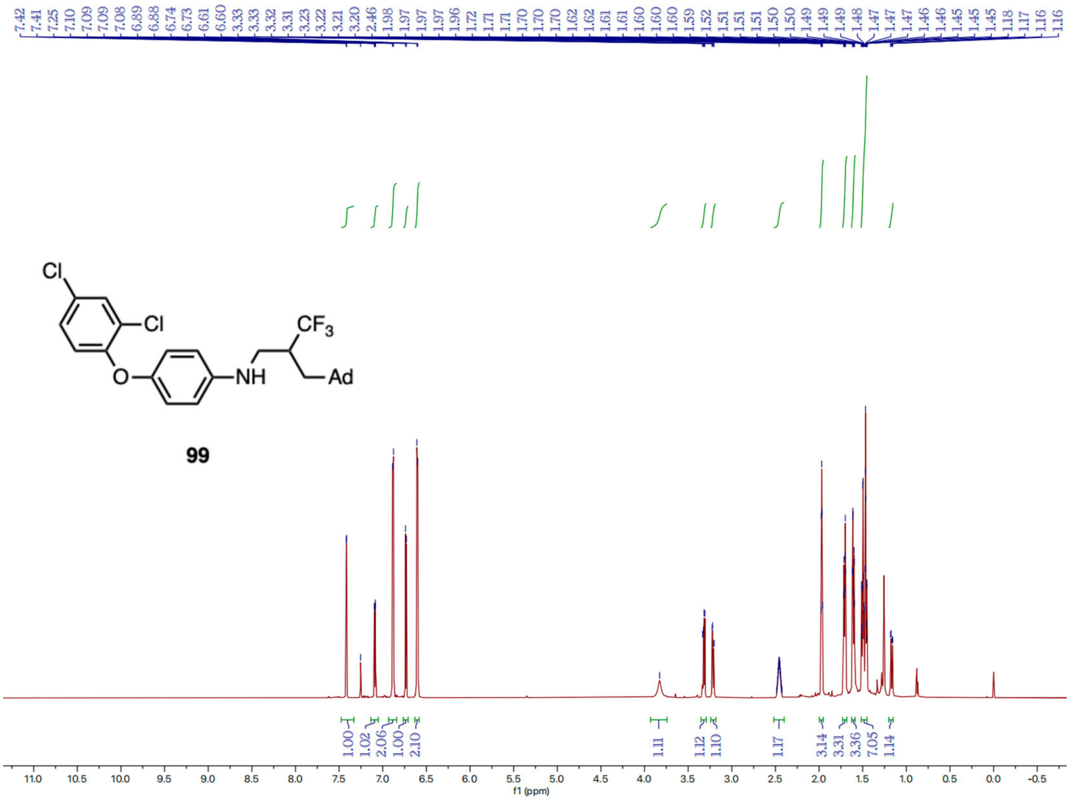

$^{1}$H NMR spectrum (800 MHz, Chloroform-*d*) of compound **99**

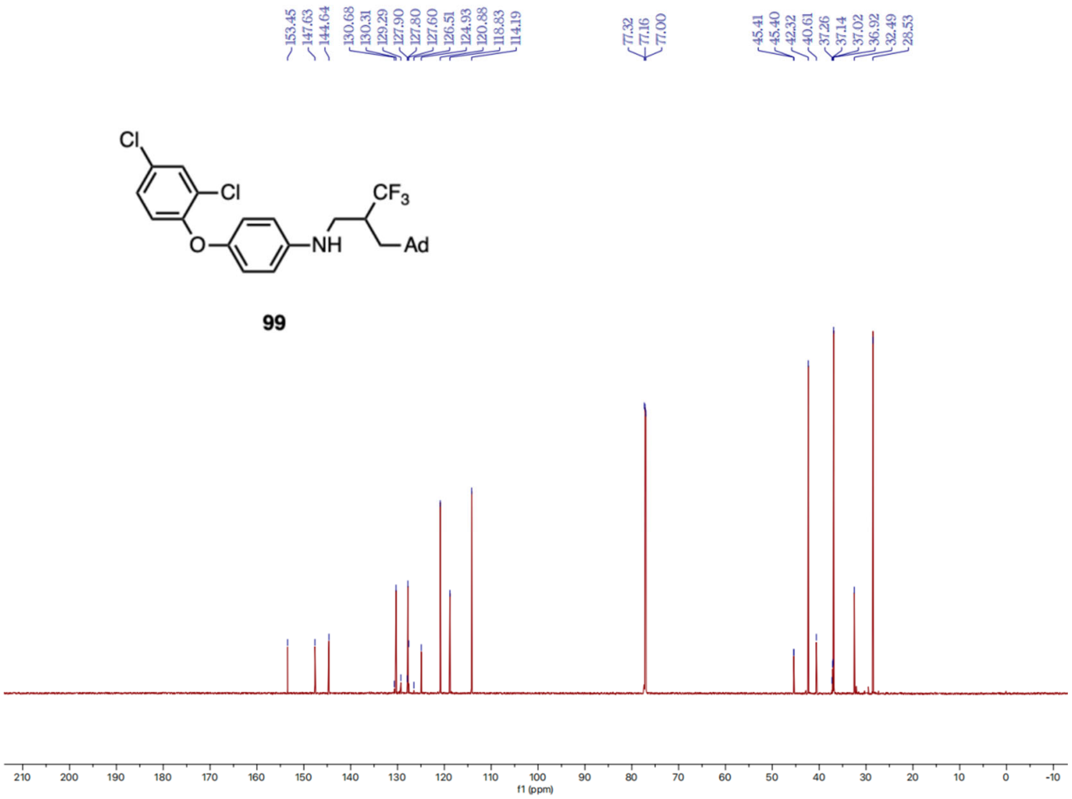

$^{13}$C NMR spectrum (201 MHz, Chloroform-*d*) of compound **99**

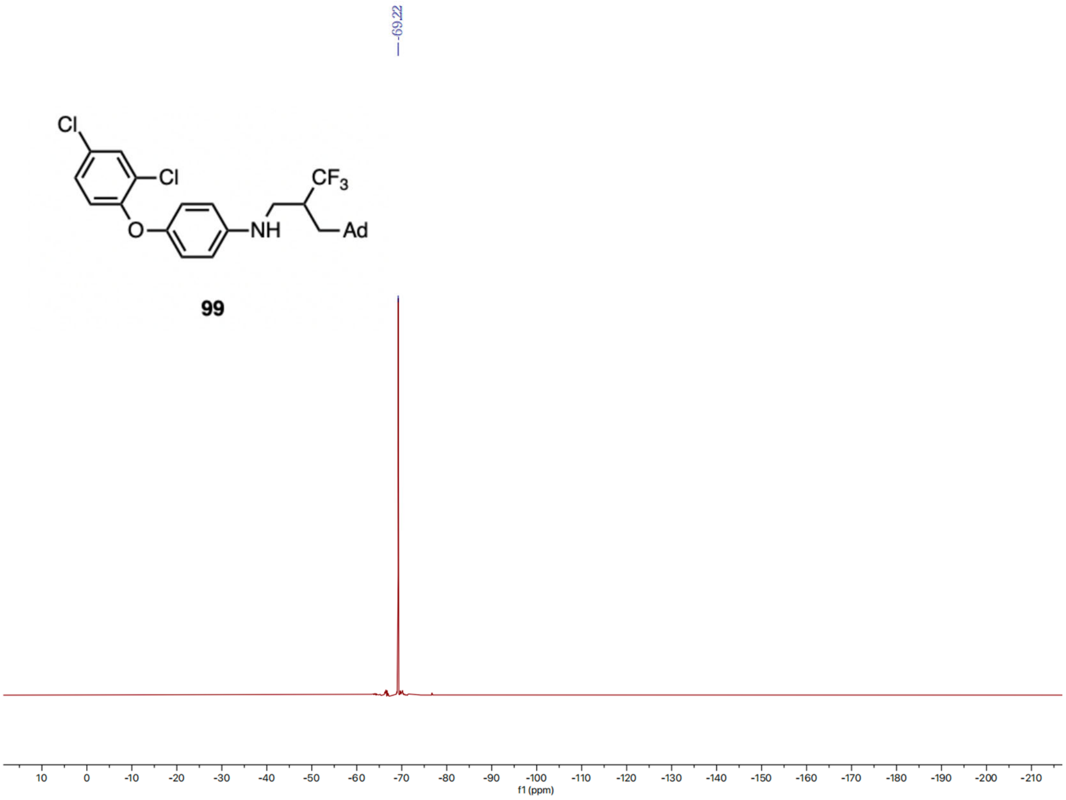


$^{19}$F NMR spectrum (376 MHz, Chloroform-*d*) of compound **99**

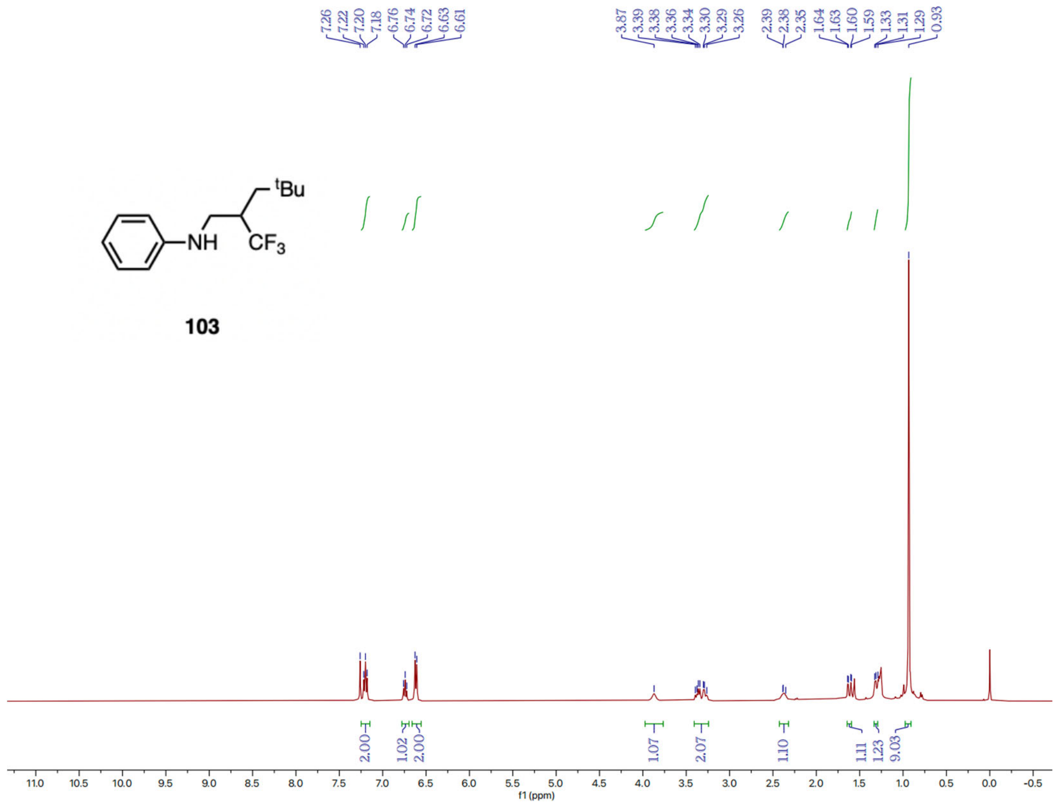

$^1H$ NMR spectrum (400 MHz, Chloroform-*d*) of compound **103**

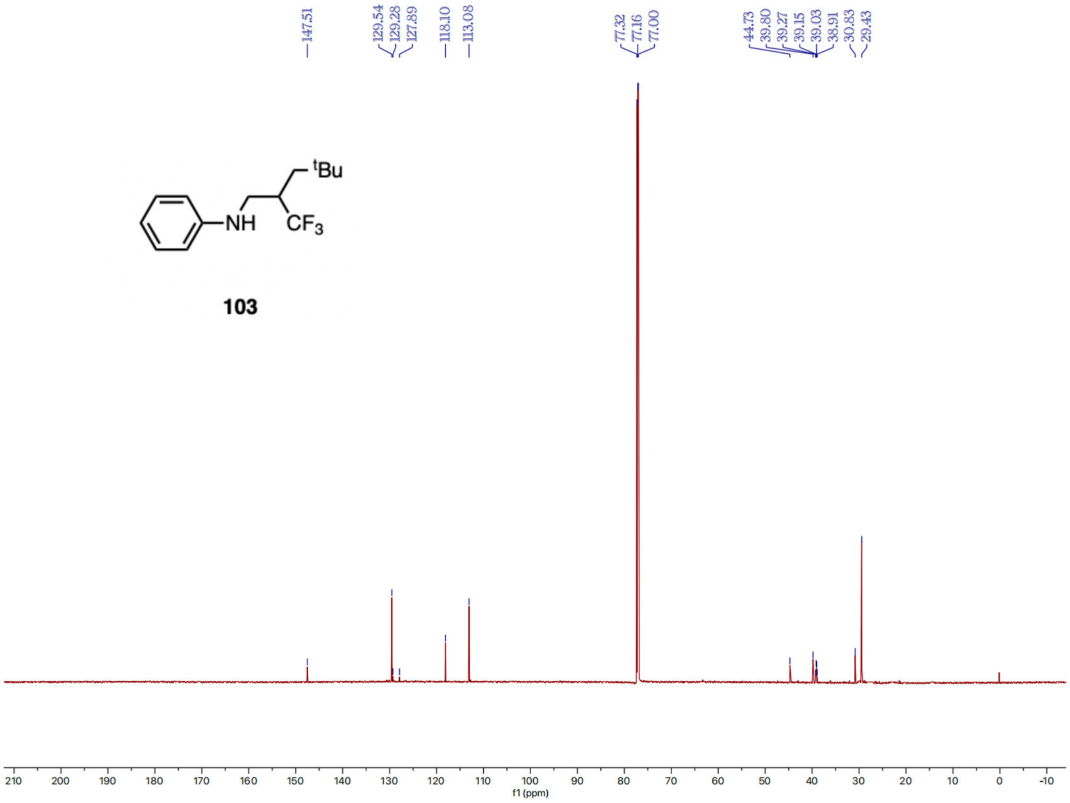

$^{13}C$ NMR spectrum (201 MHz, Chloroform-*d*) of compound **103**

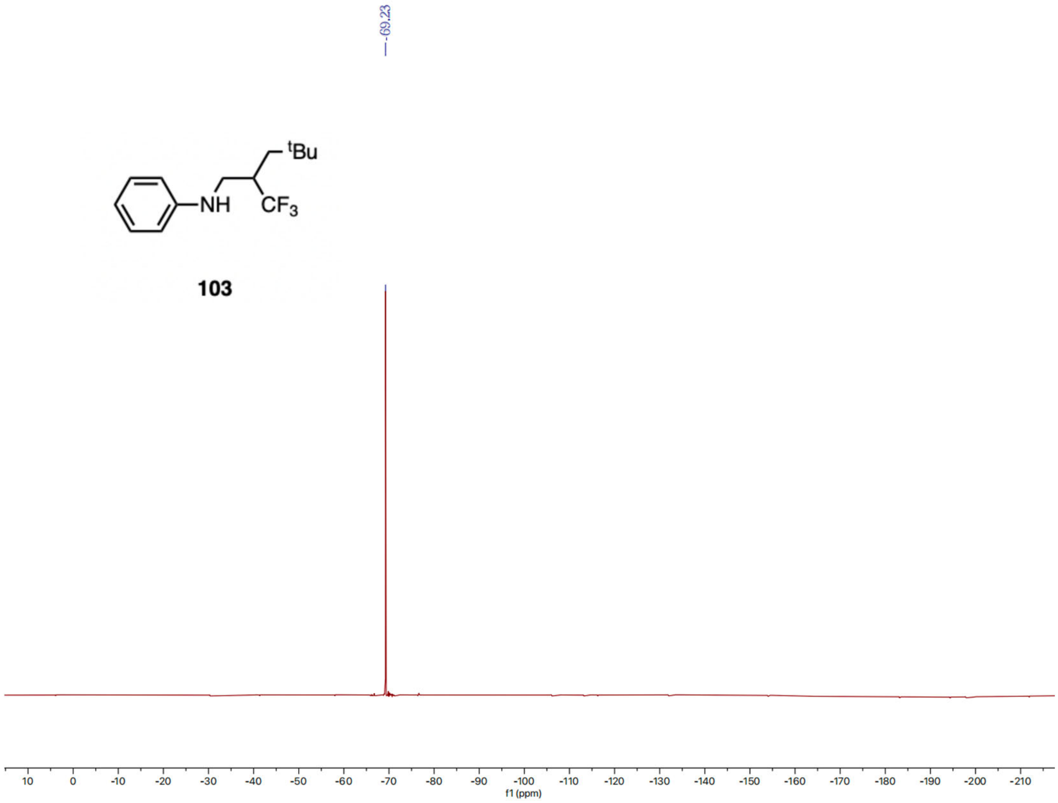


$^{19}$F NMR spectrum (376 MHz, Chloroform-*d*) of compound **103**

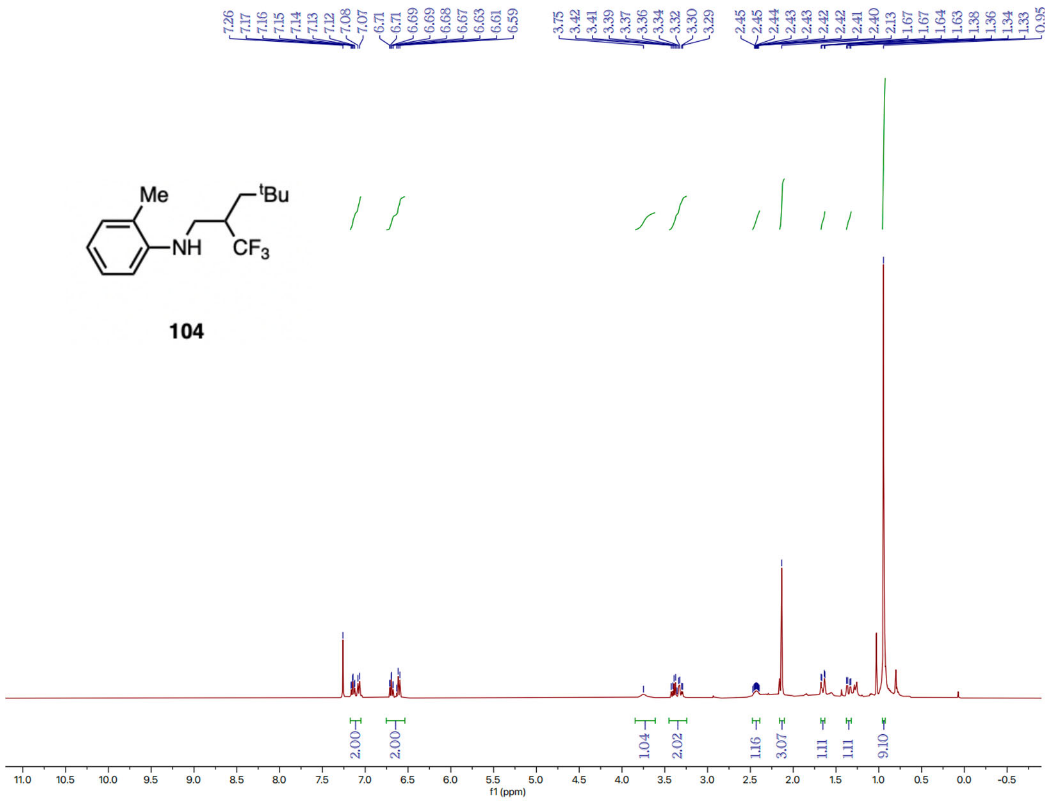

$^{1}$H NMR spectrum (400 MHz, Chloroform-*d*) of compound **104**

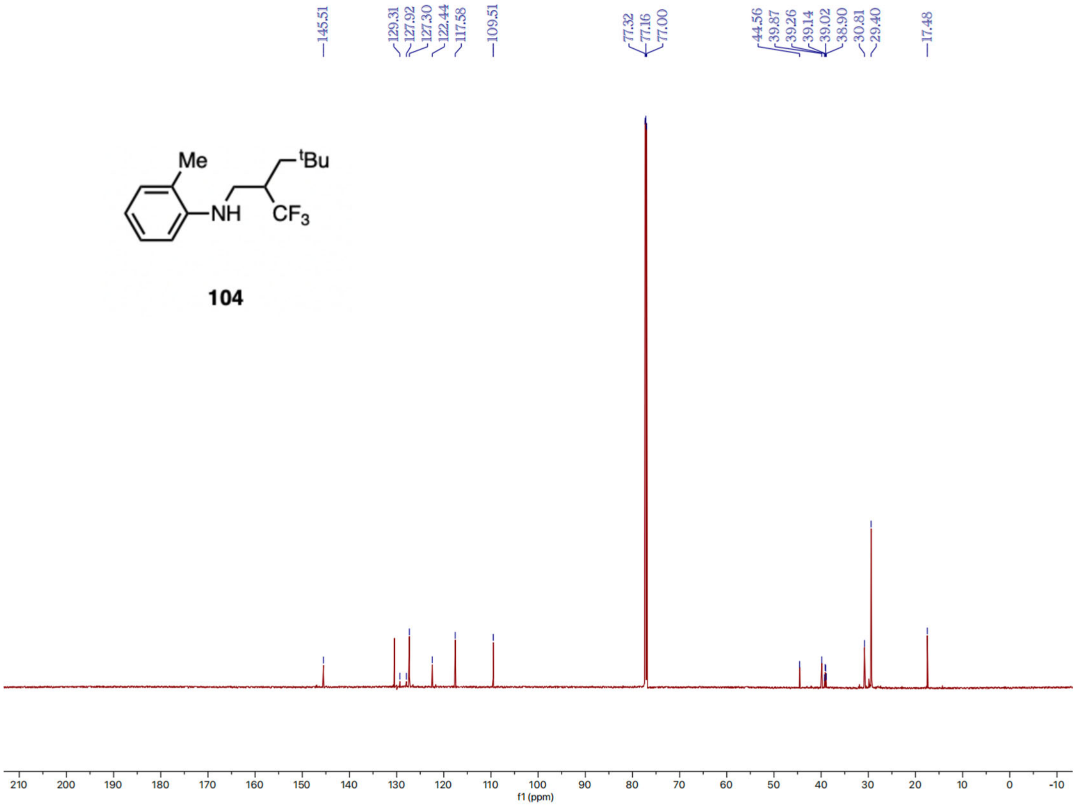

$^{13}$C NMR spectrum (201 MHz, Chloroform-*d*) of compound **104**

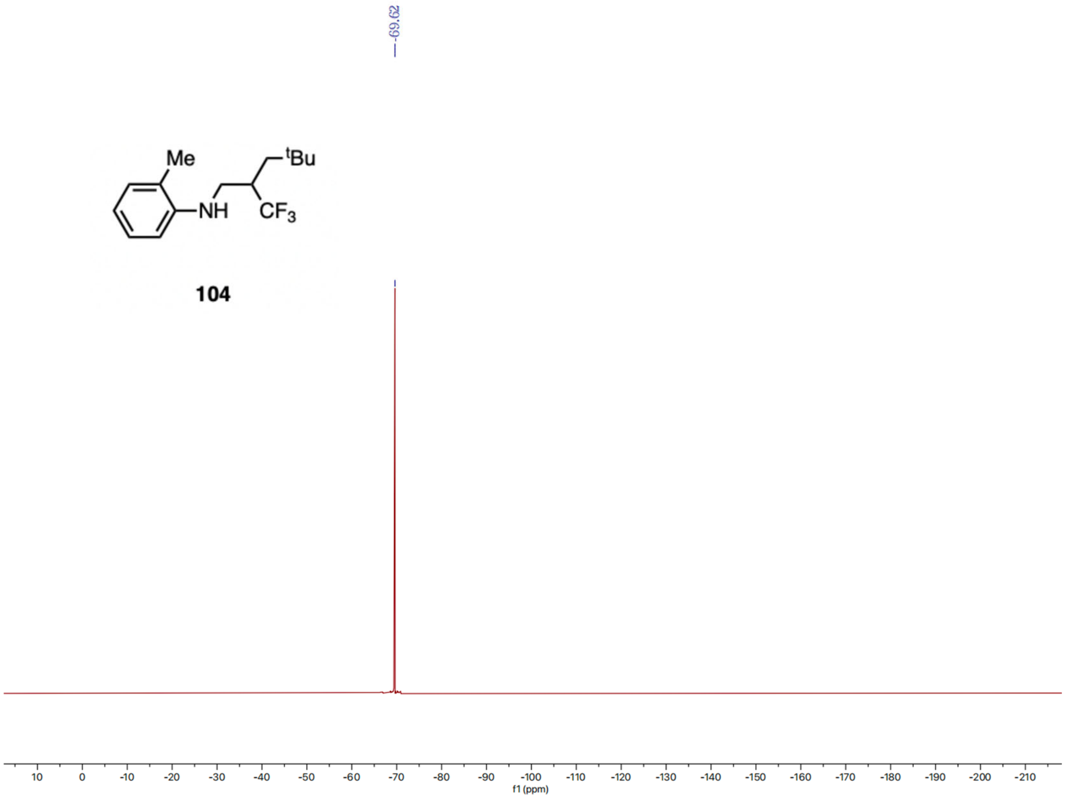


$^{19}F$ NMR spectrum (376 MHz, Chloroform-*d*) of compound **104**

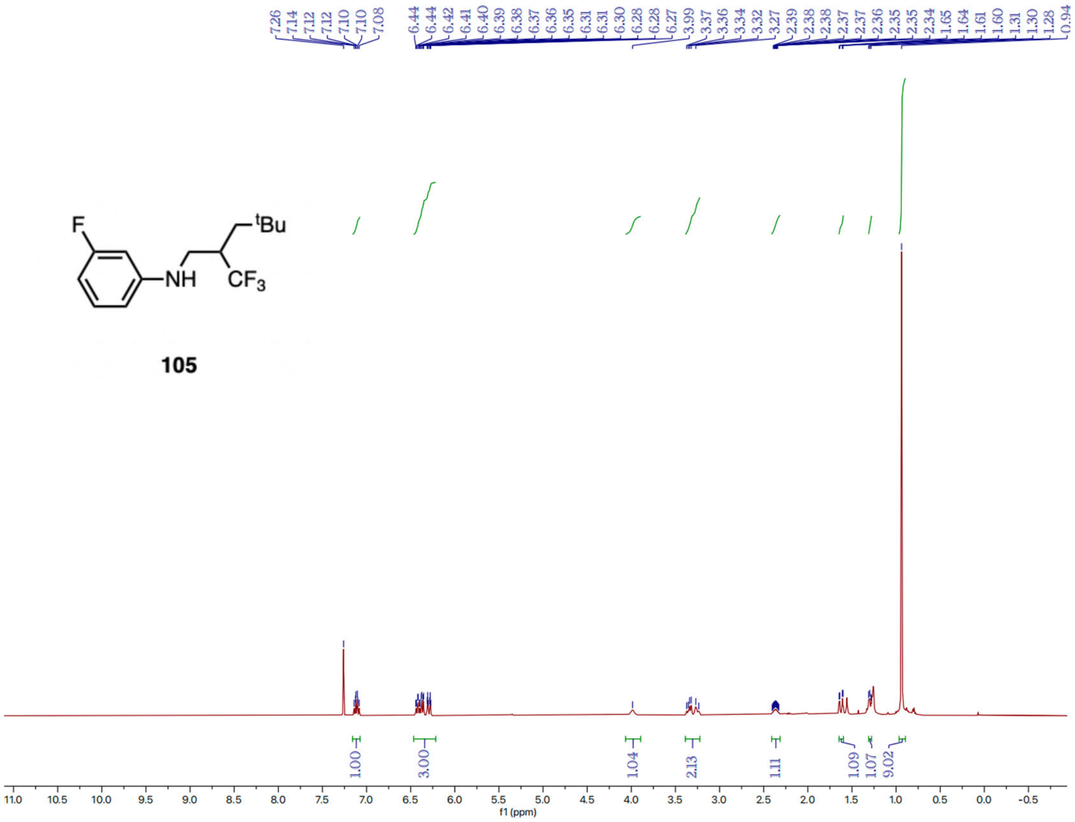

$^{1}$H NMR spectrum (400 MHz, Chloroform-*d*) of compound **105**

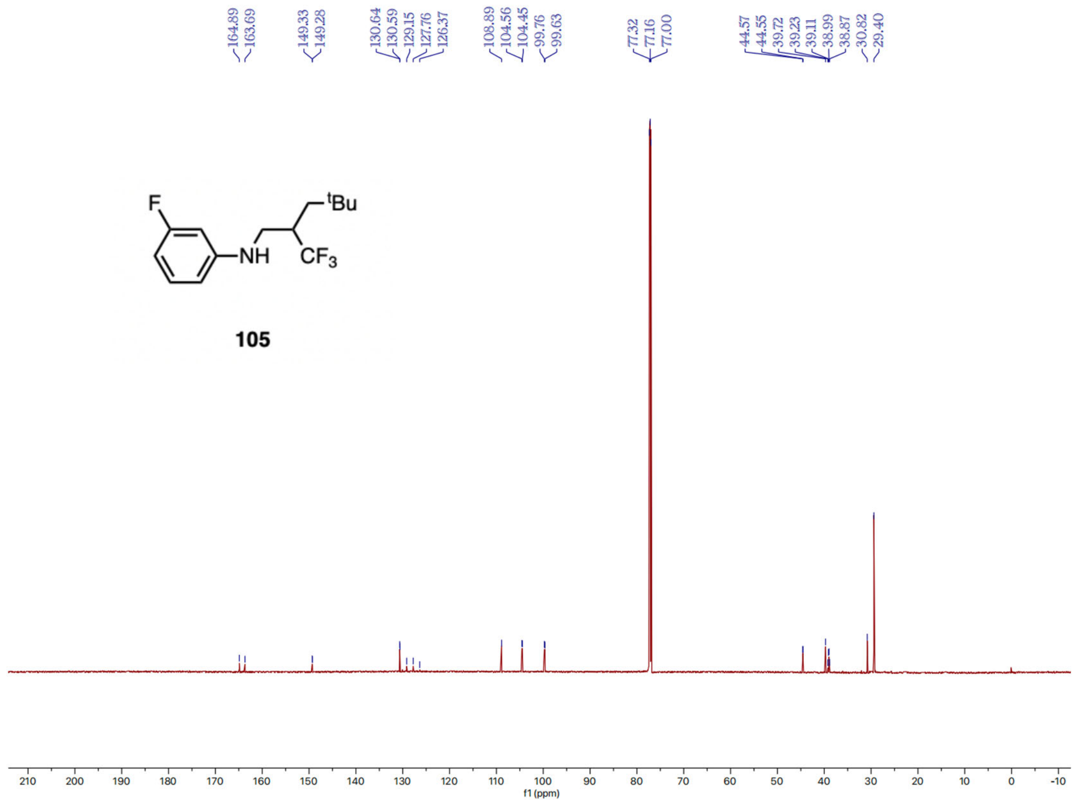

$^{13}$C NMR spectrum (201 MHz, Chloroform-*d*) of compound **105**

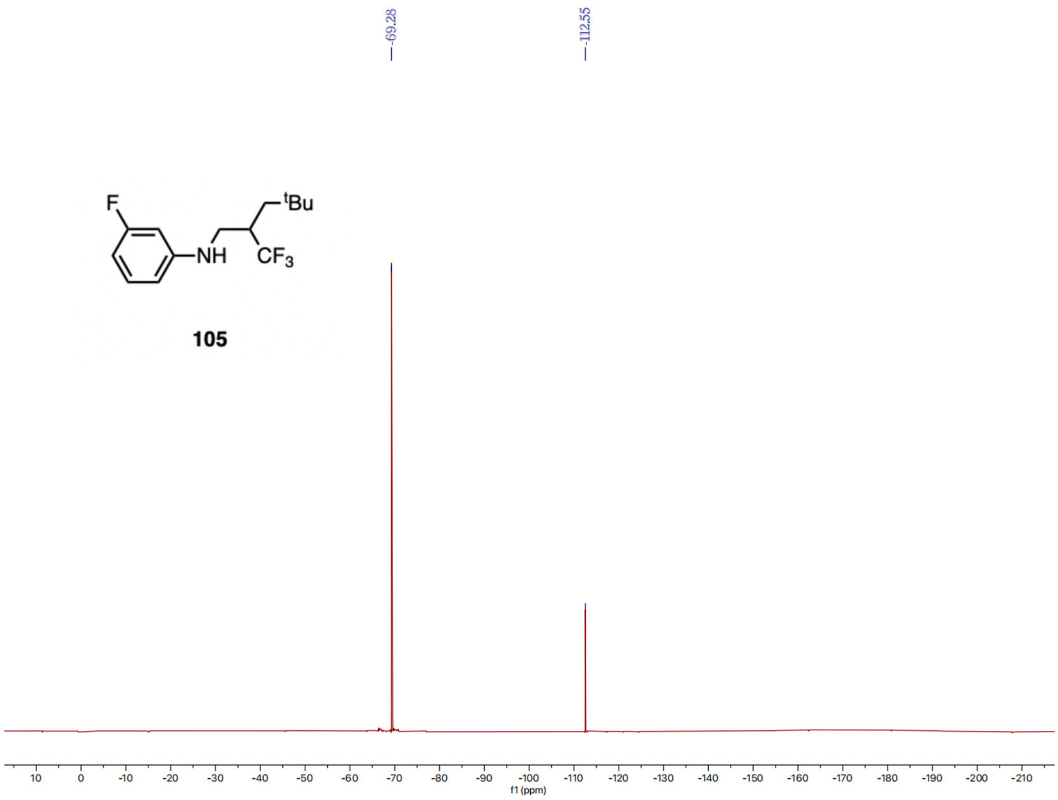


$^{19}F$ NMR spectrum (376 MHz, Chloroform-*d*) of compound **105**

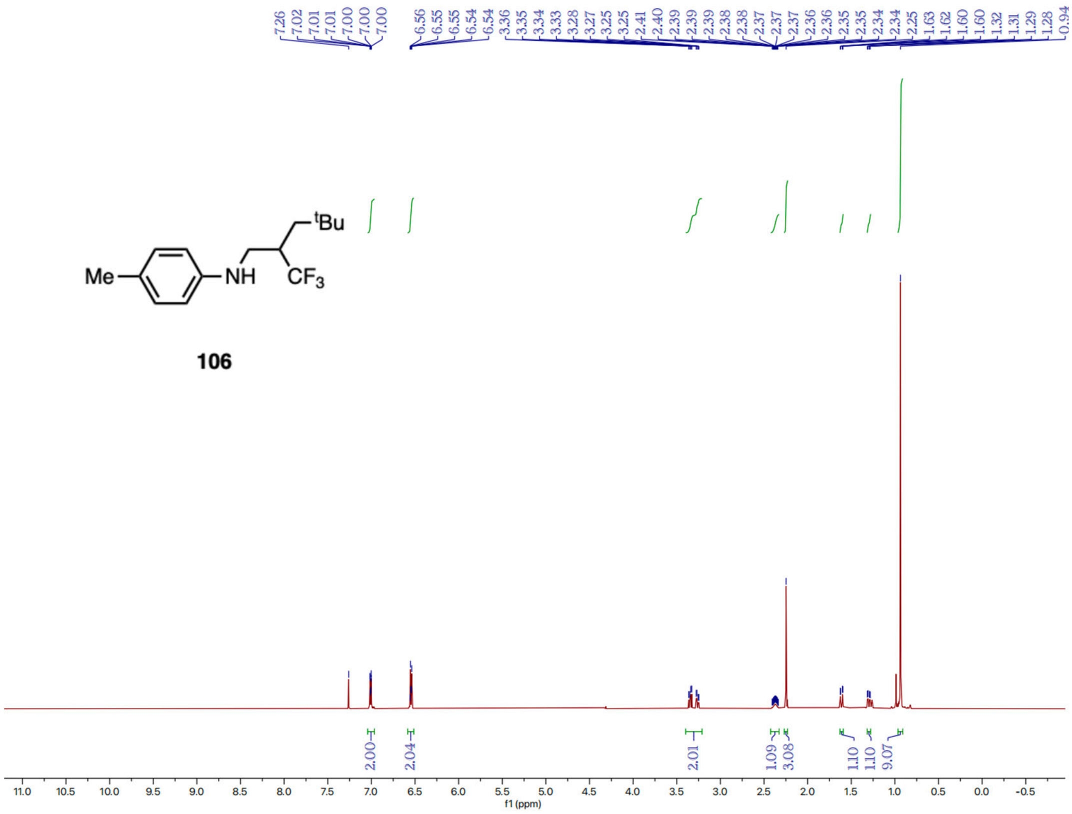


$^{1}$H NMR spectrum (600 MHz, Chloroform-*d*) of compound **106**

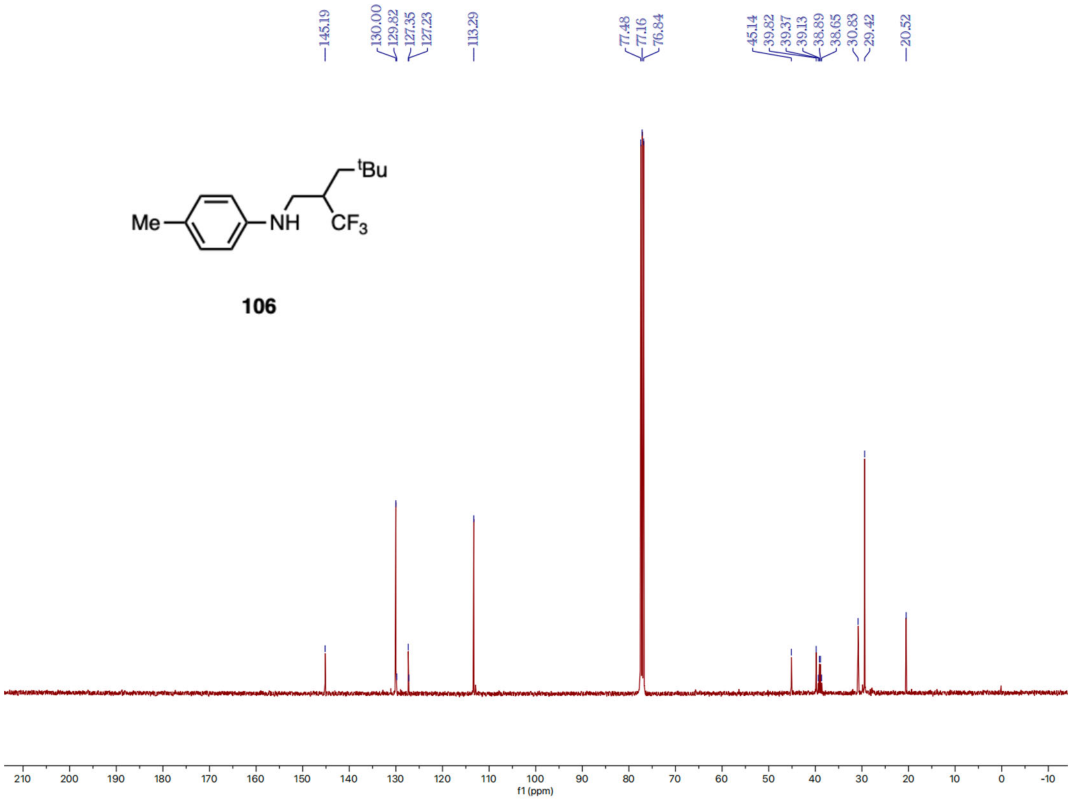


$^{13}$C NMR spectrum (101 MHz, Chloroform-*d*) of compound **106**

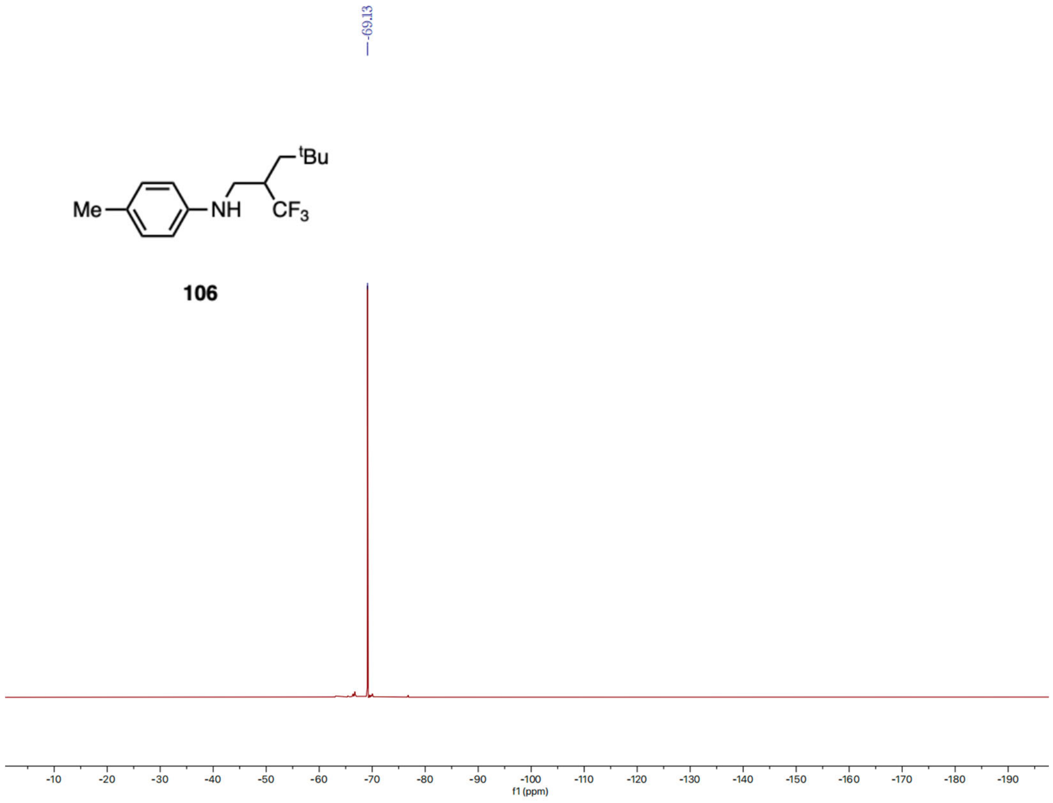


$^{19}$F NMR spectrum (565 MHz, Chloroform-*d*) of compound **106**

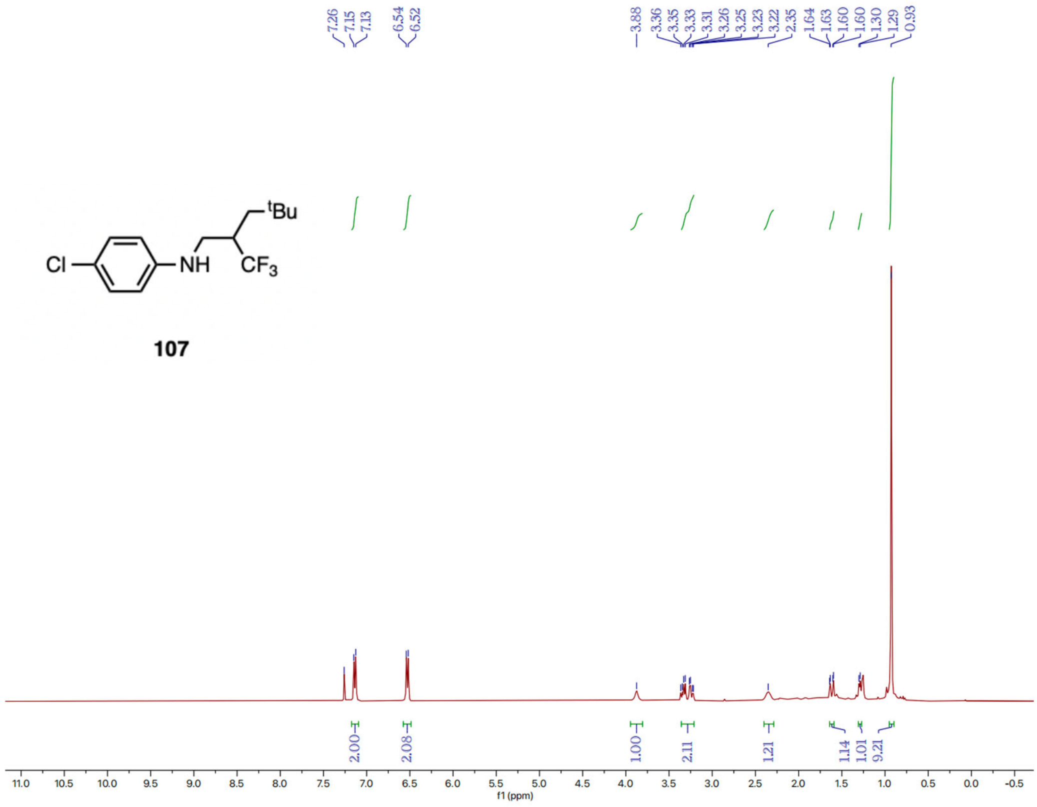

$^{1}$H NMR spectrum (400 MHz, Chloroform-*d*) of compound **107**

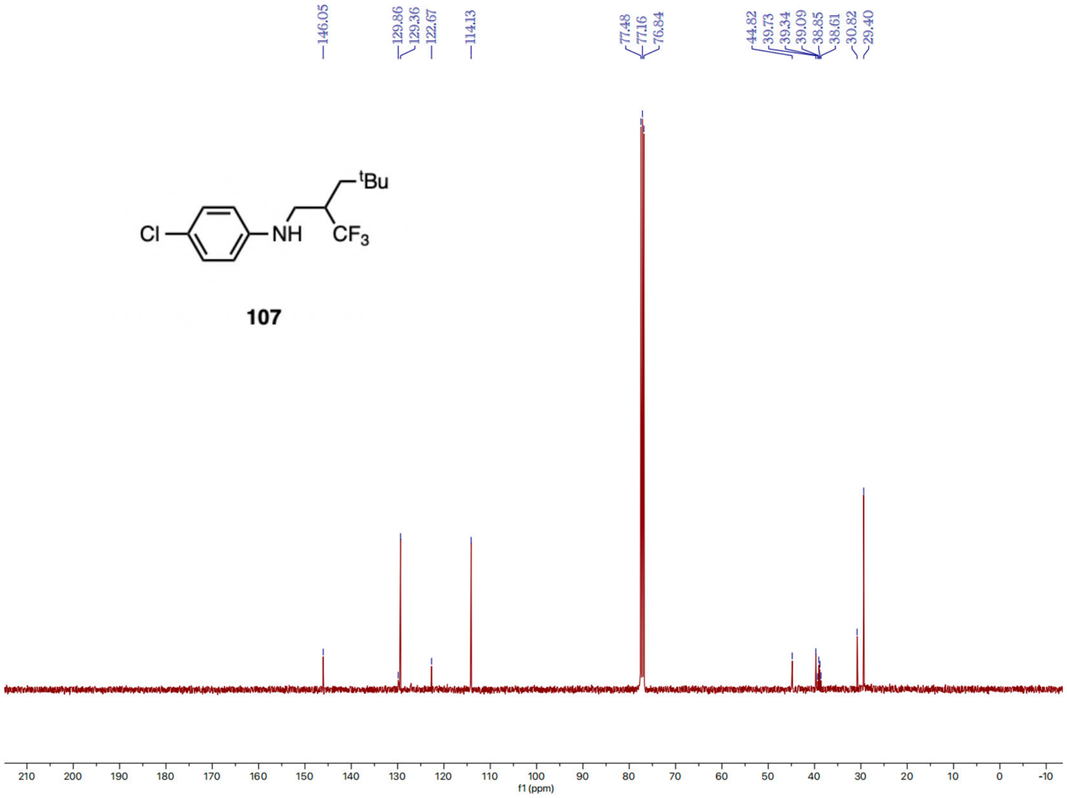

$^{13}$C NMR spectrum (101 MHz, Chloroform-*d*) of compound **107**

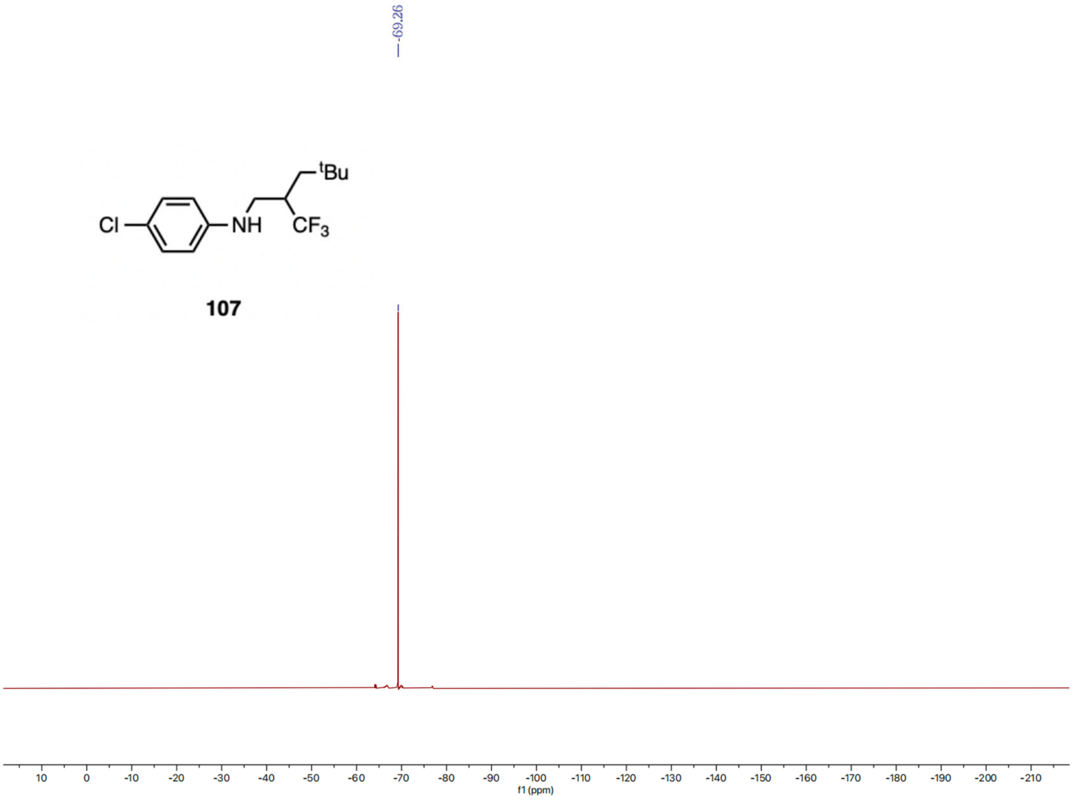

$^{19}$F NMR spectrum (376 MHz, Chloroform-*d*) of compound **107**

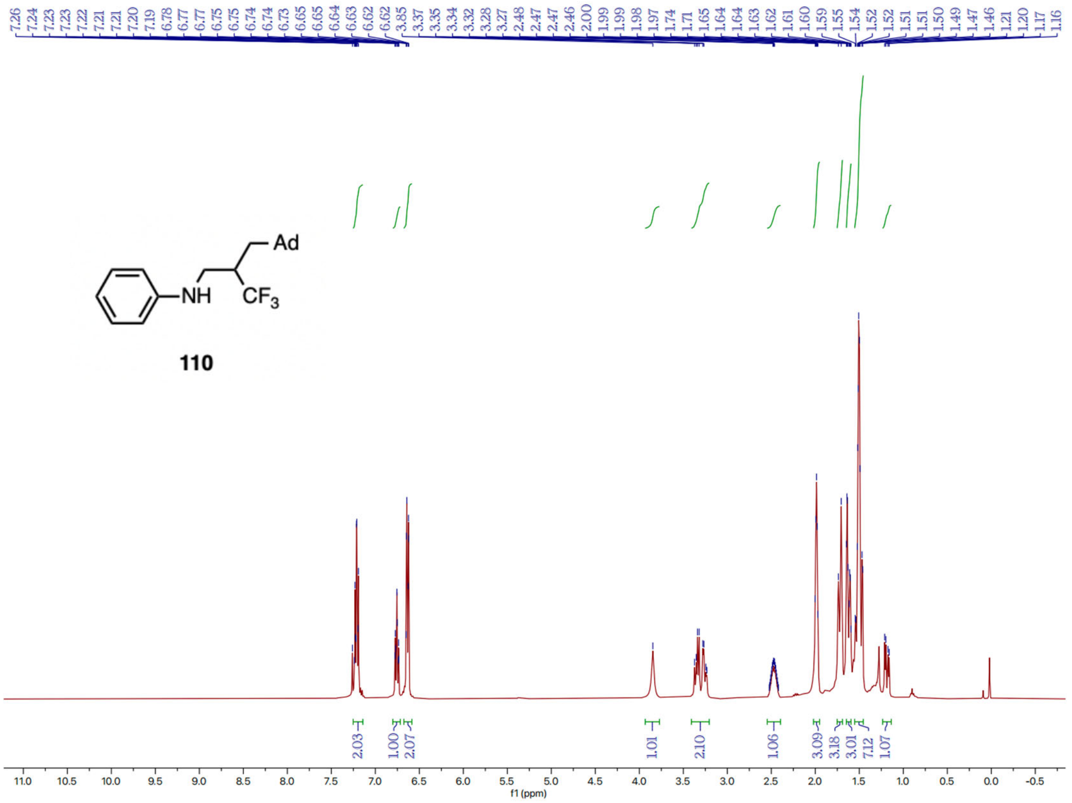


$^{1}$H NMR spectrum (400 MHz, Chloroform-*d*) of compound **110**

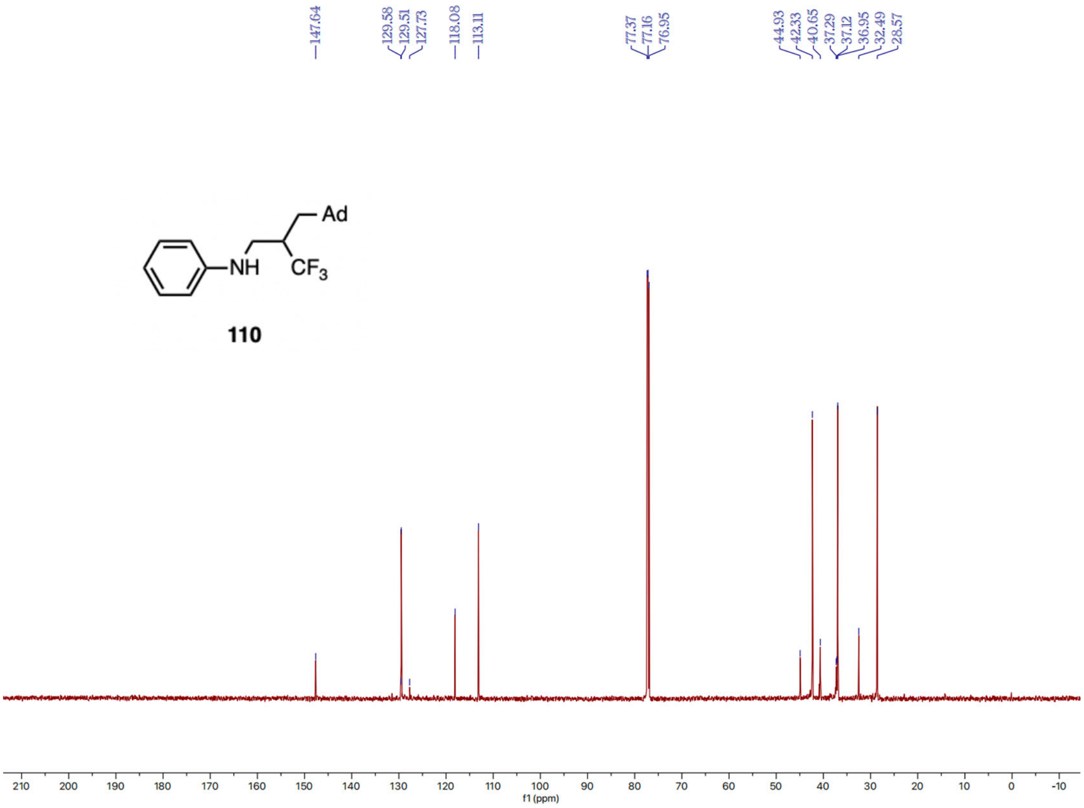


$^{13}$C NMR spectrum (151 MHz, Chloroform-*d*) of compound **110**

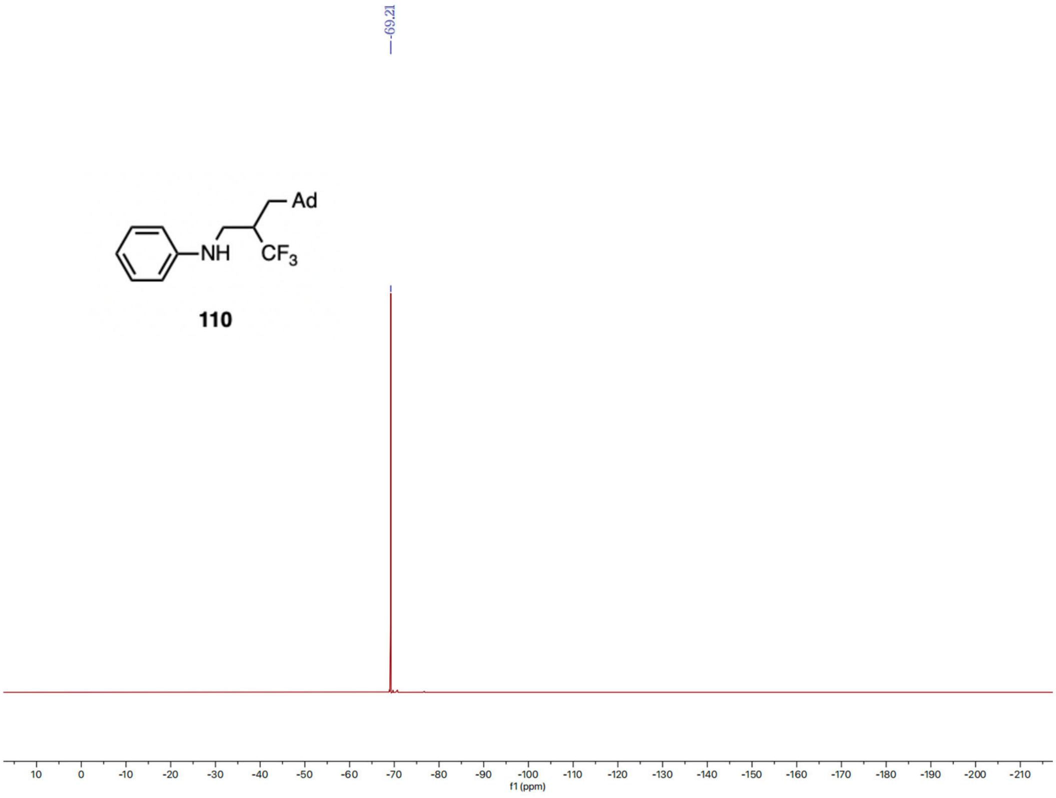


$^{19}F$ NMR spectrum (376 MHz, Chloroform-*d*) of compound **110**

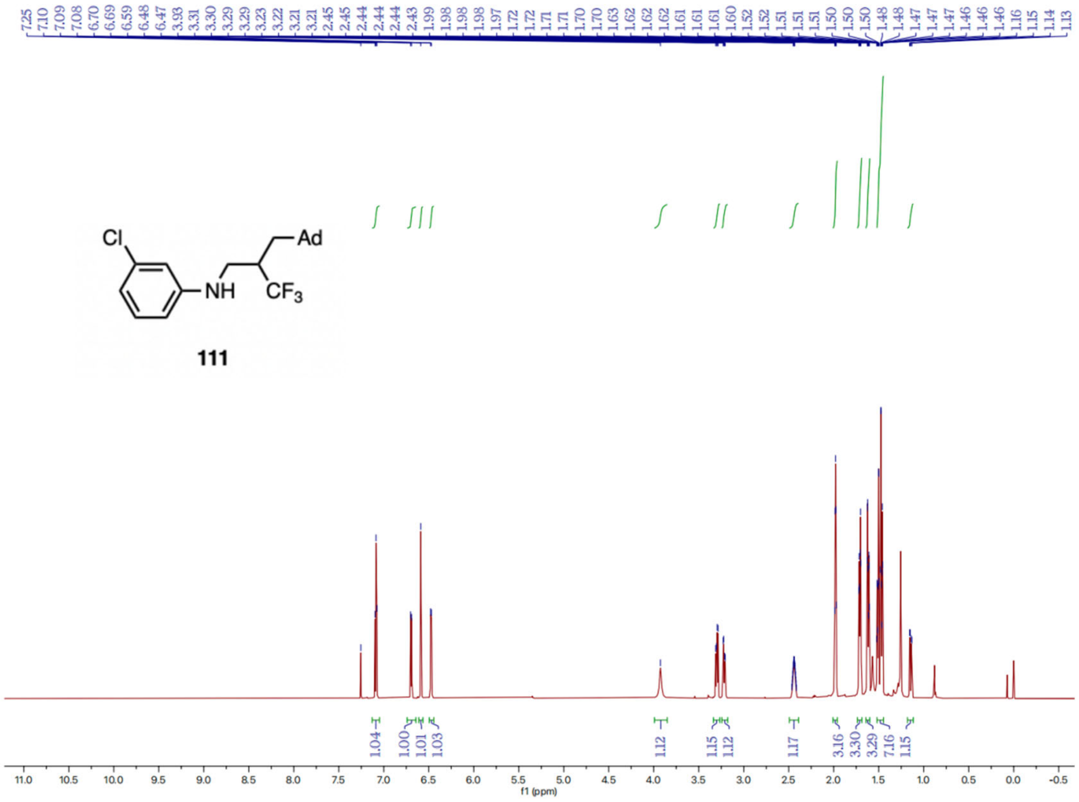

$^{1}$H NMR spectrum (800 MHz, Chloroform-*d*) of compound **111**

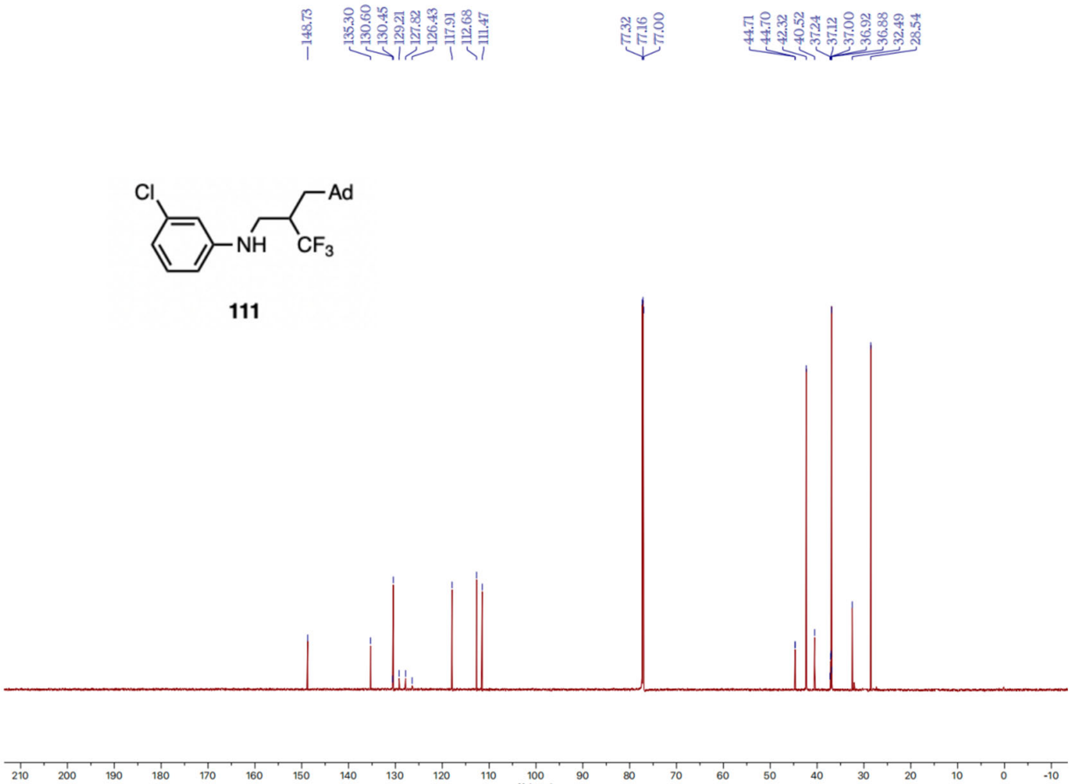

$^{13}$C NMR spectrum (201 MHz, Chloroform-*d*) of compound **111**

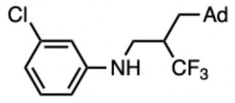


111

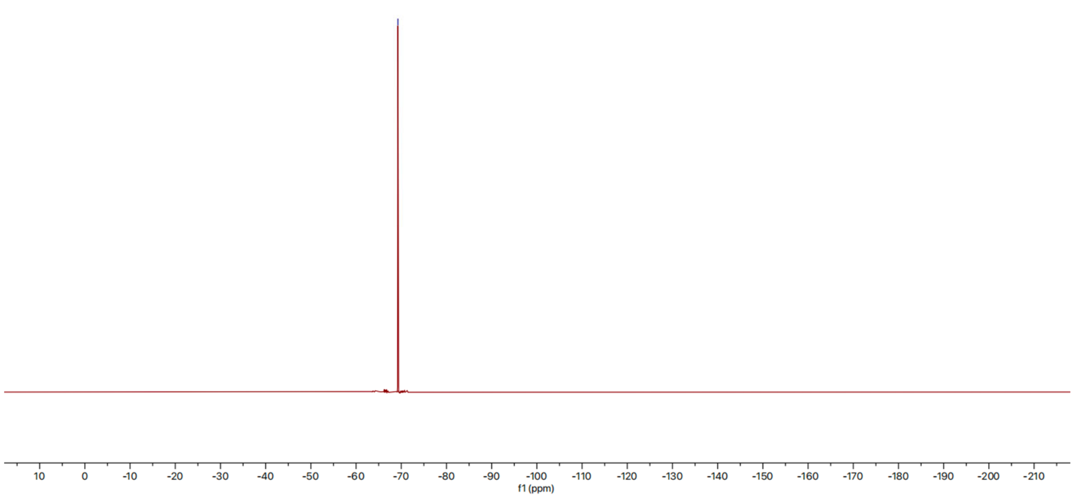


$^{19}$F NMR spectrum (376 MHz, Chloroform-*d*) of compound **111**

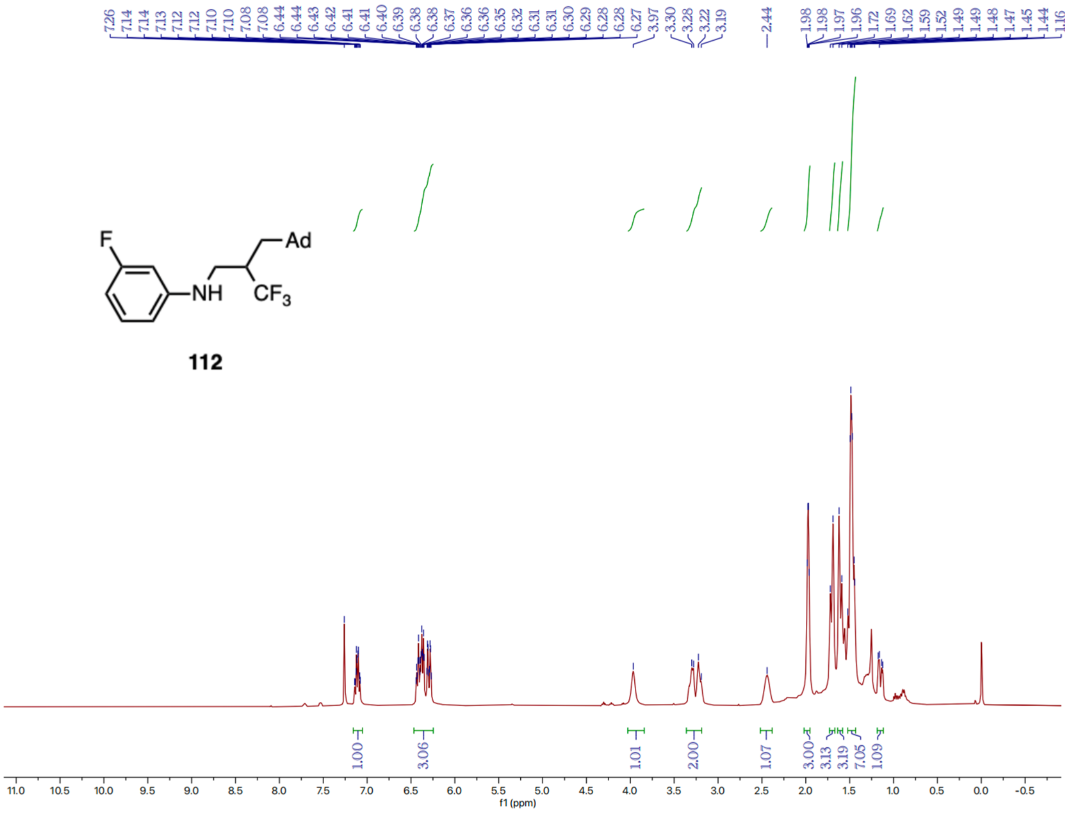


$^{1}$H NMR spectrum (400 MHz, Chloroform-*d*) of compound **112**

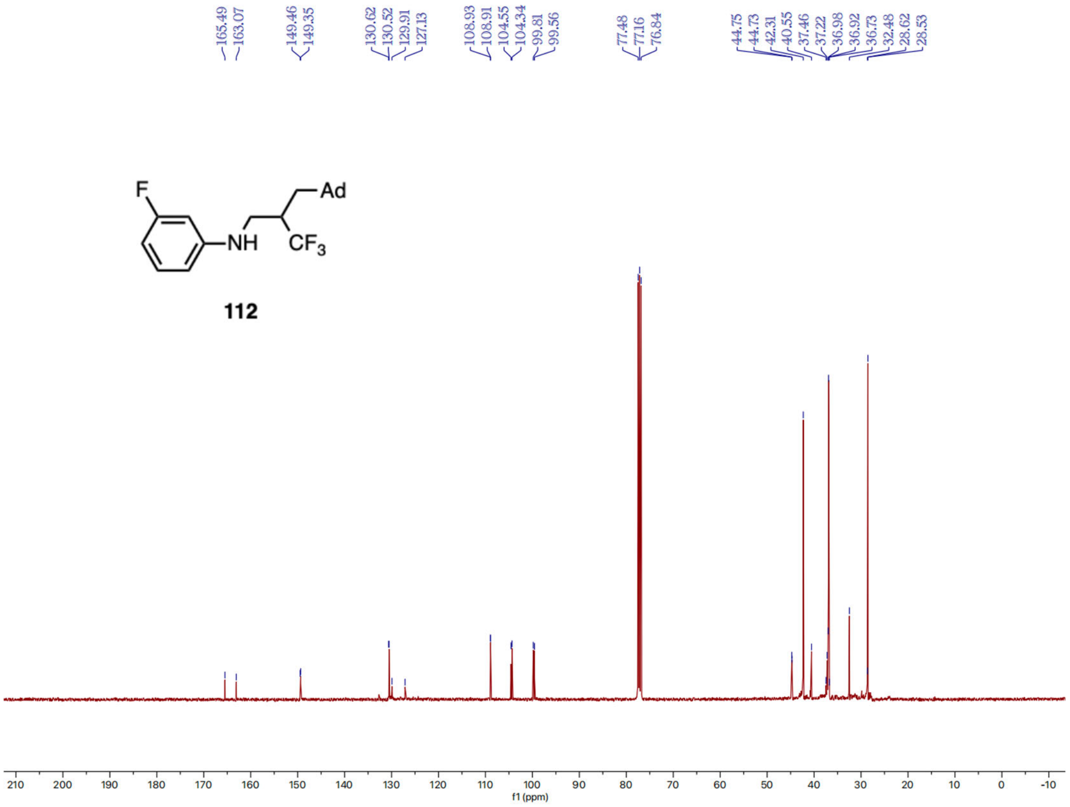


$^{13}$C NMR spectrum (101 MHz, Chloroform-*d*) of compound **112**

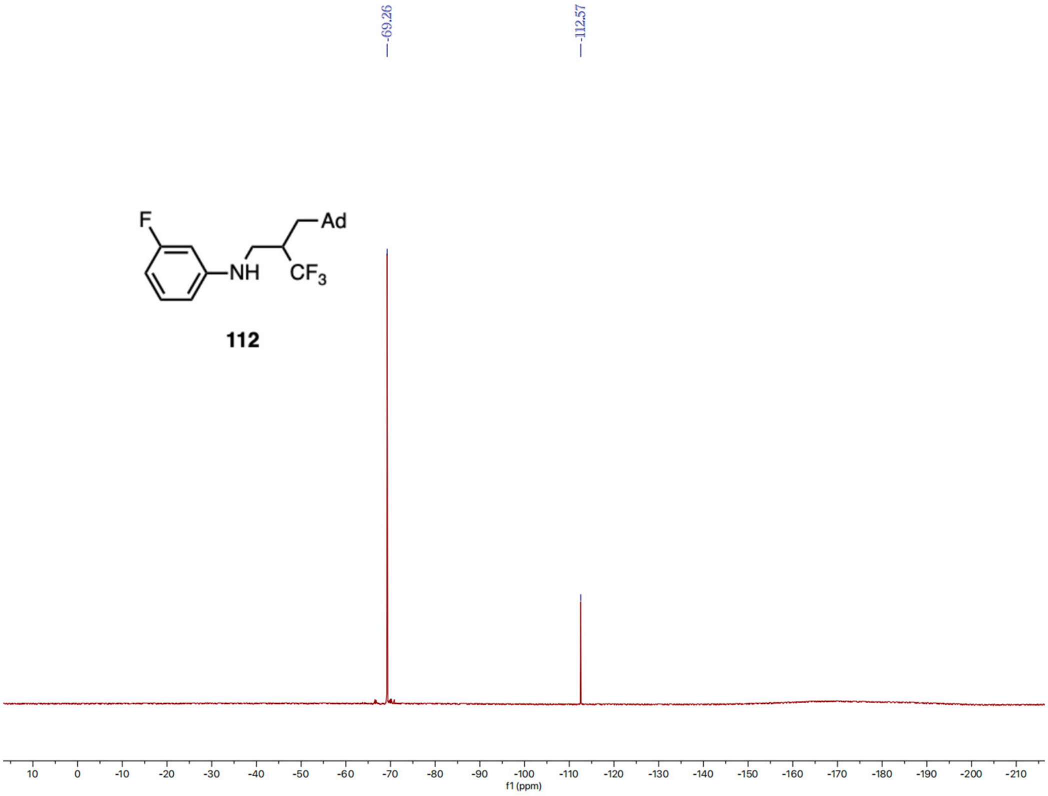


$^{19}F$ NMR spectrum (376 MHz, Chloroform-*d*) of compound **112**

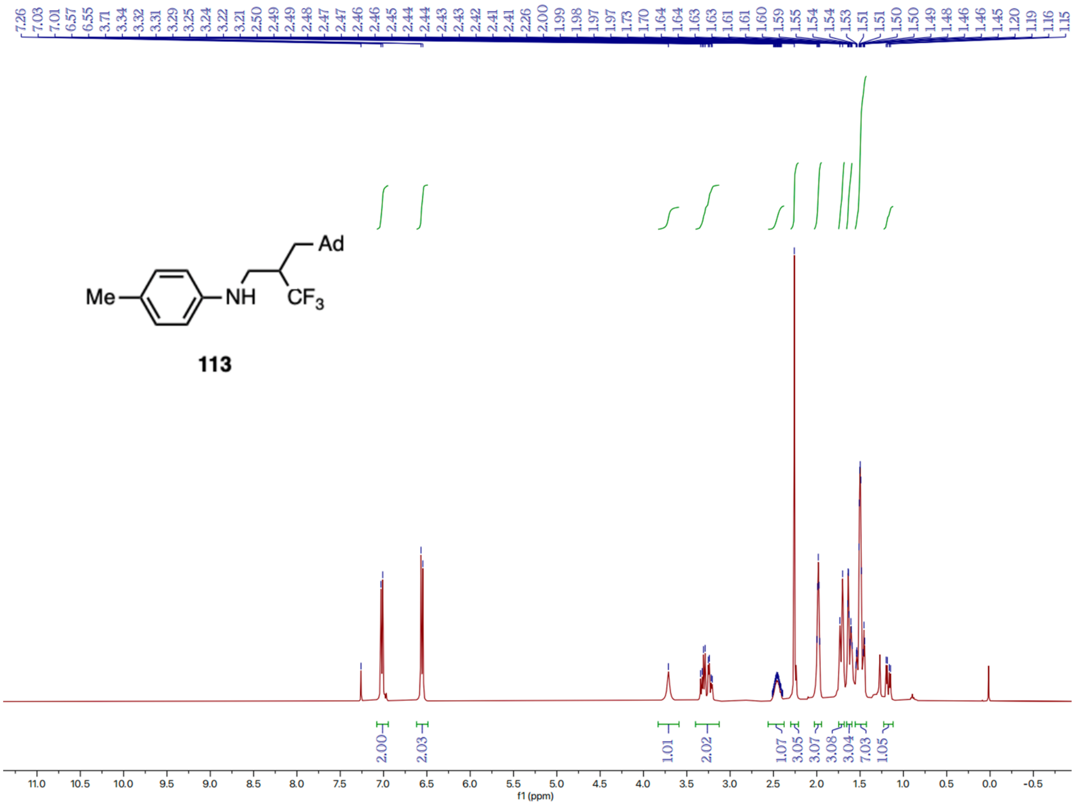

$^{1}$H NMR spectrum (400 MHz, Chloroform-*d*) of compound **113**

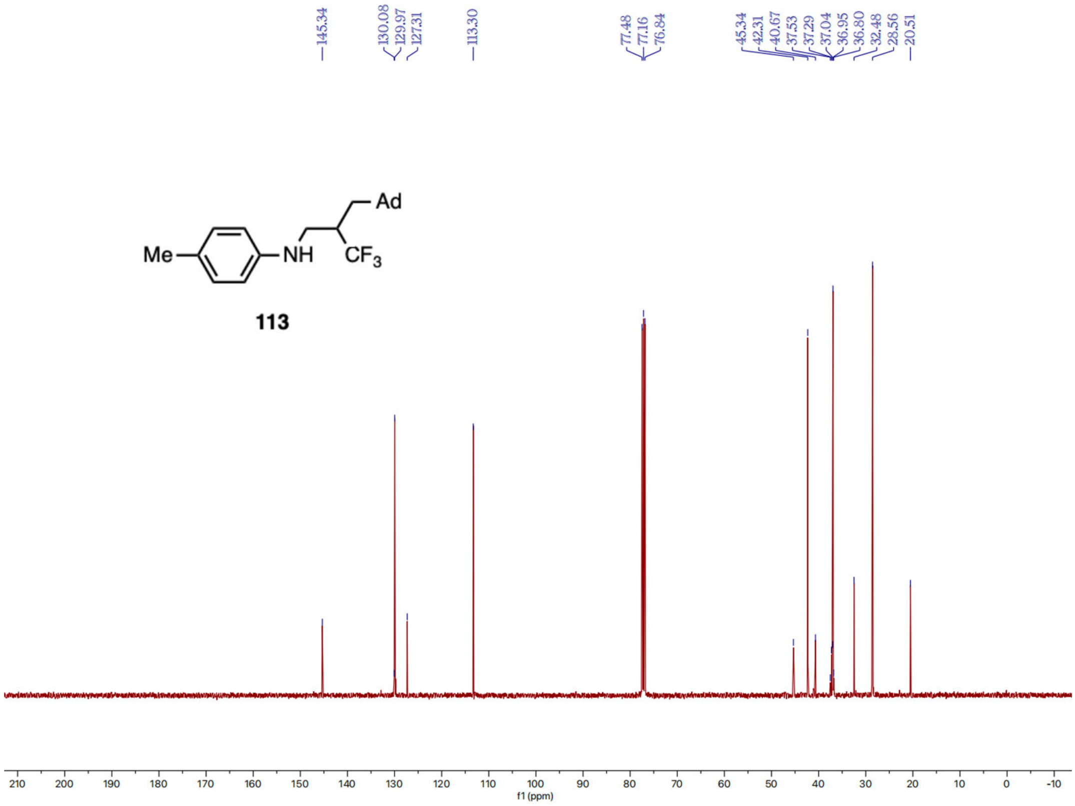

$^{13}$C NMR spectrum (101 MHz, Chloroform-*d*) of compound **113**

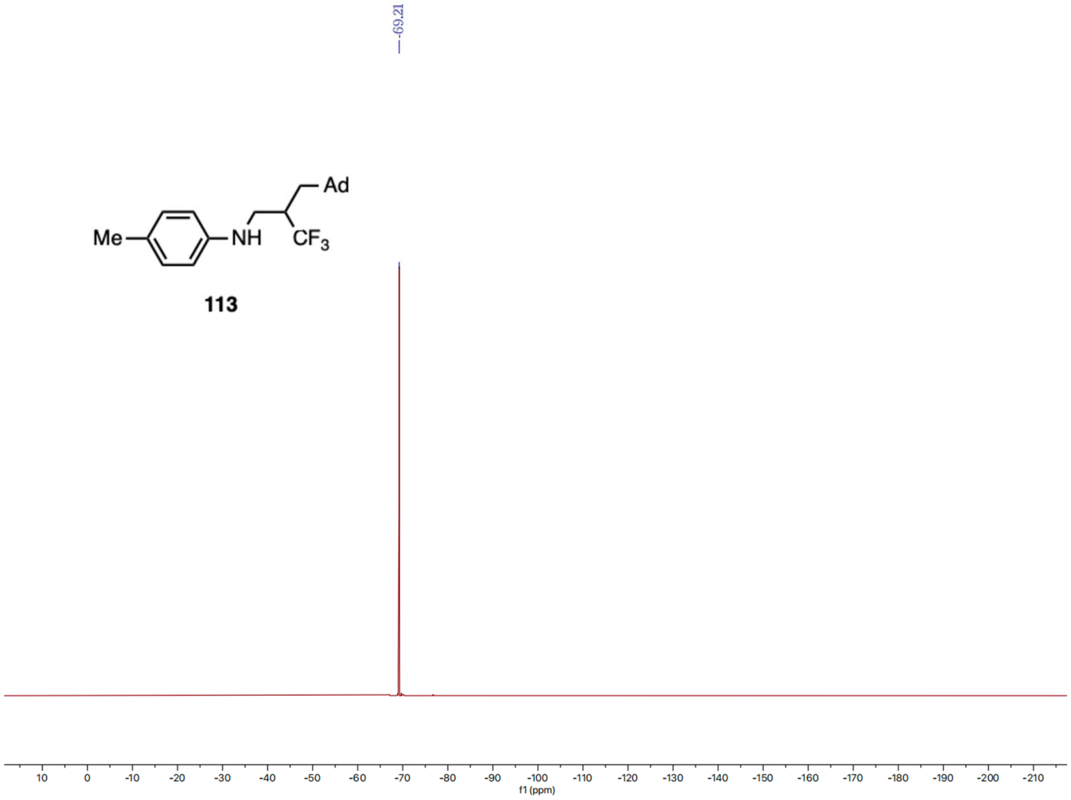


$^{19}$F NMR spectrum (376 MHz, Chloroform-*d*) of compound **113**

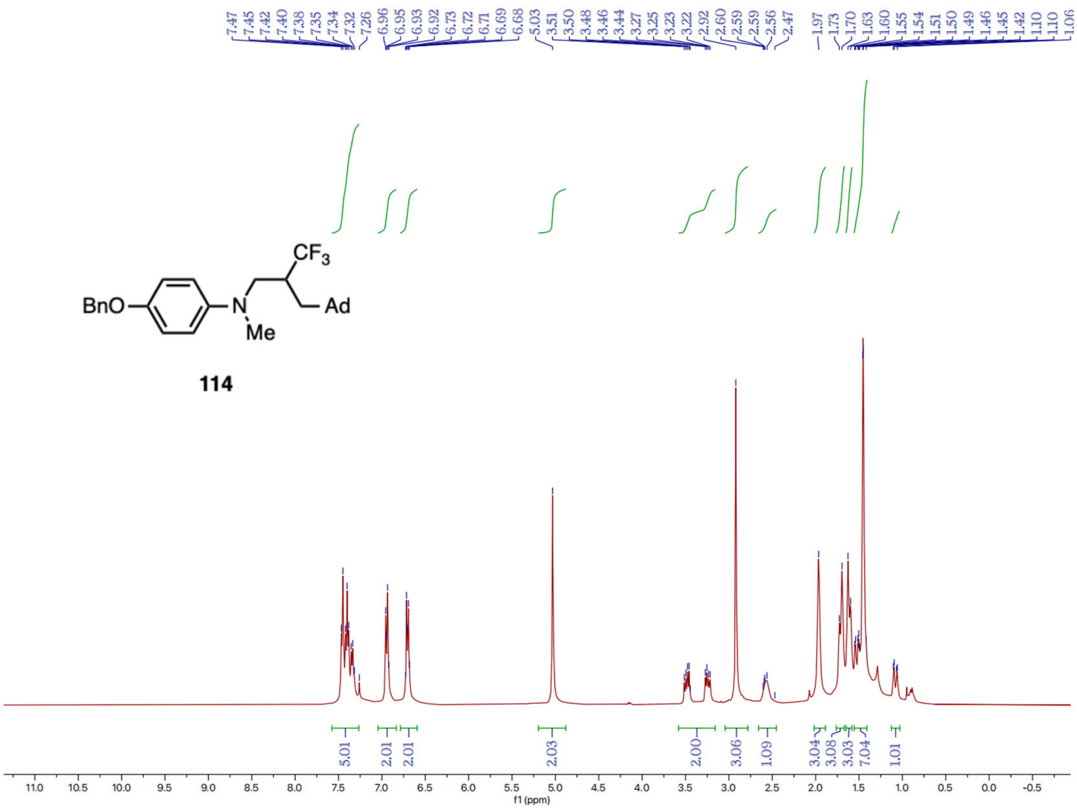

$^1H$ NMR spectrum (400 MHz, Chloroform-*d*) of compound **114**

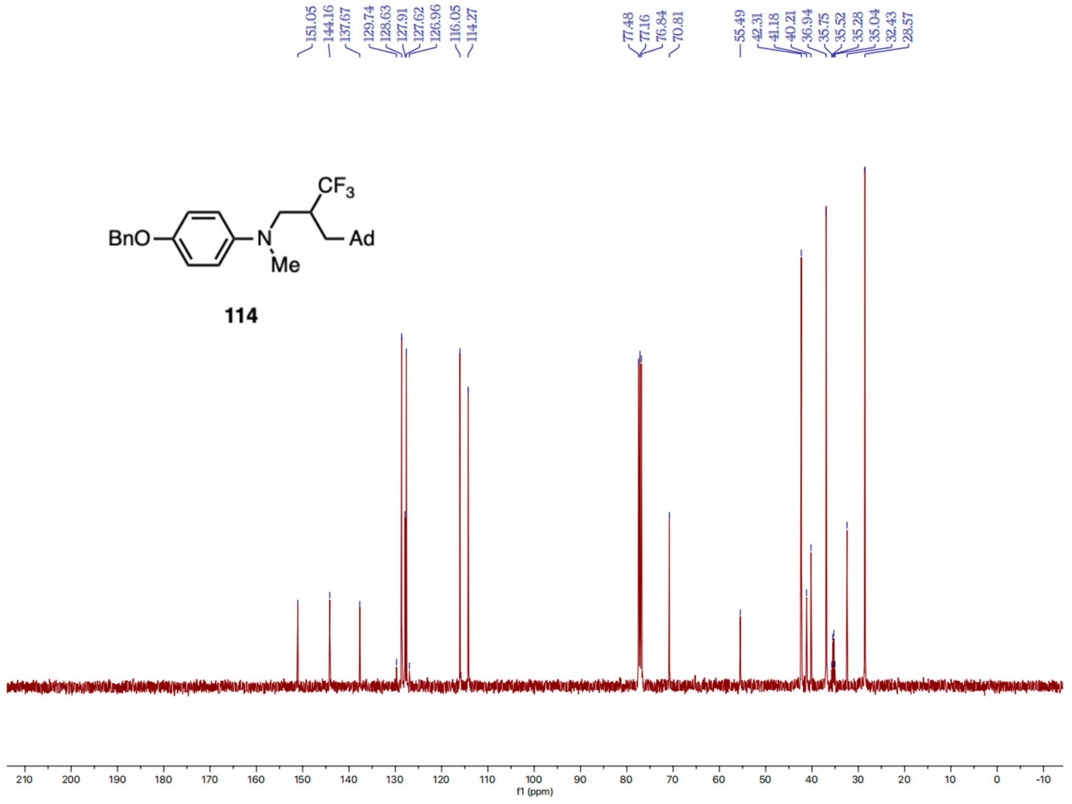

$^{13}C$ NMR spectrum (101 MHz, Chloroform-*d*) of compound **114**

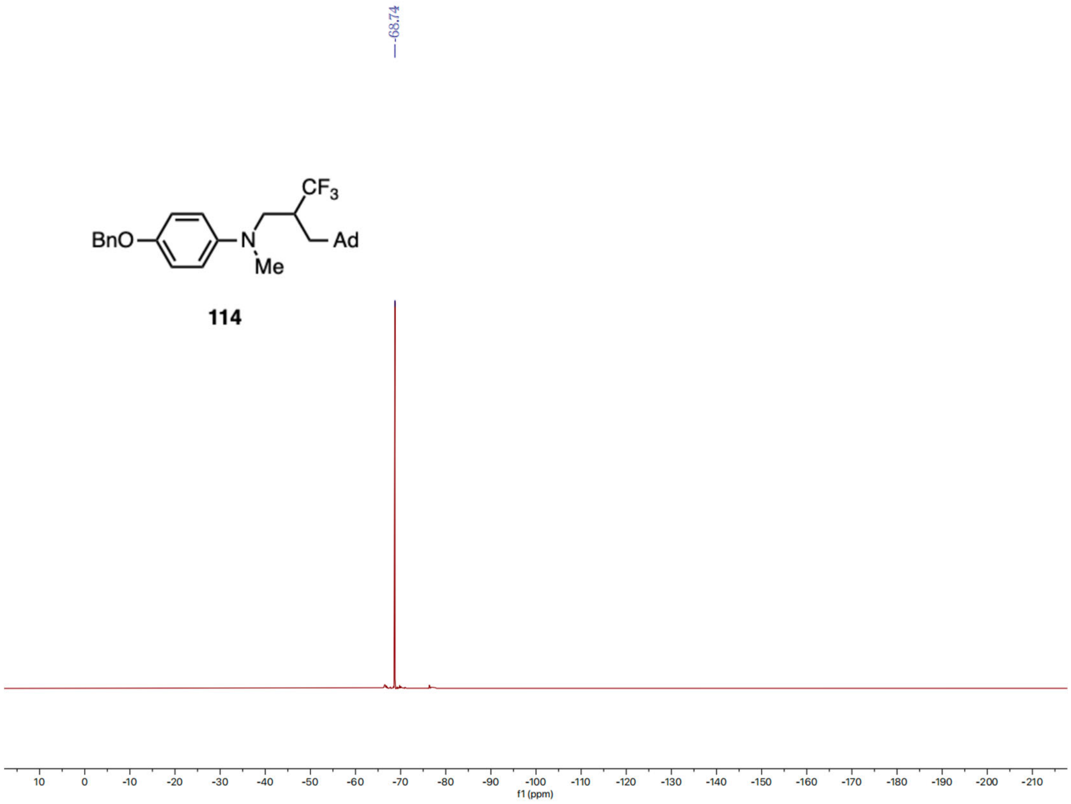


$^{19}$F NMR spectrum (376 MHz, Chloroform-*d*) of compound **114**

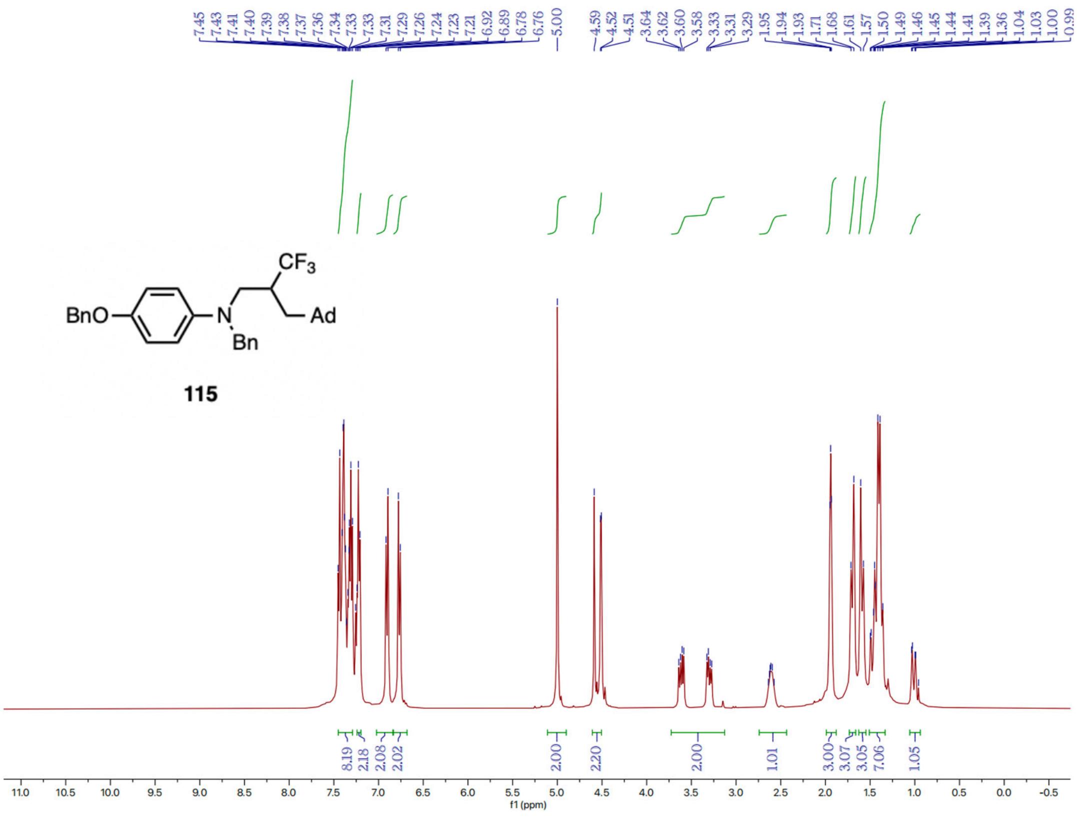

$^1$H NMR spectrum (400 MHz, Chloroform-*d*) of compound **115**

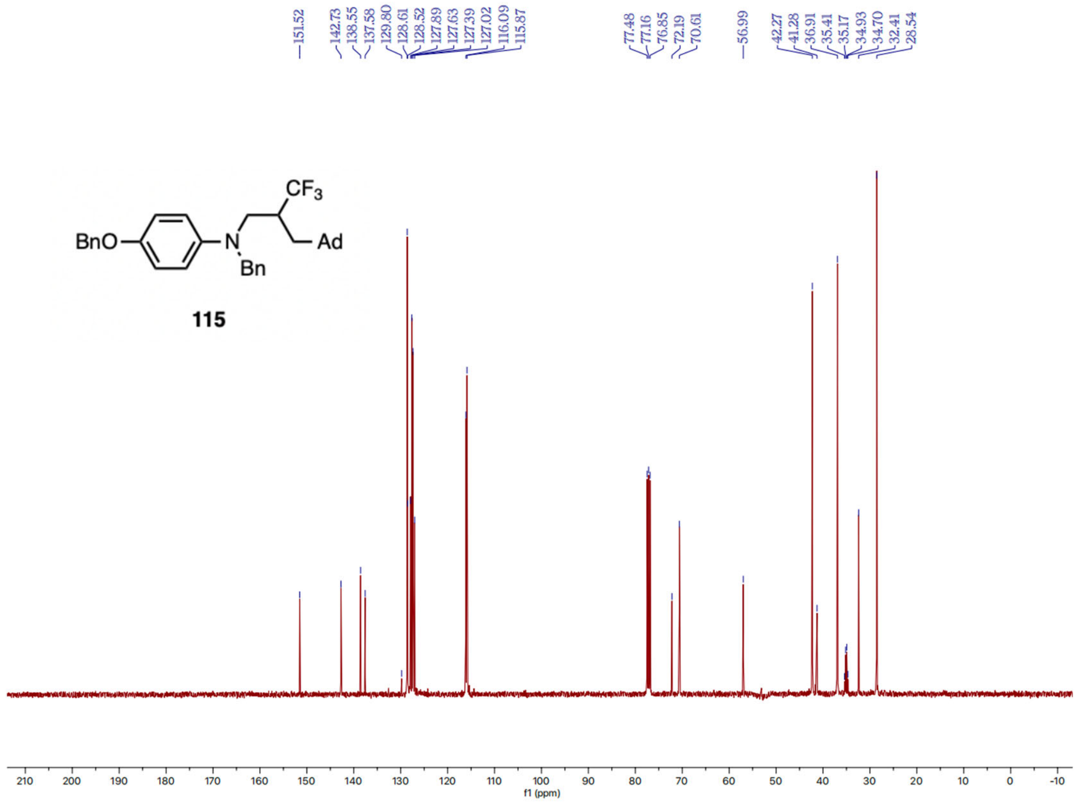

$^{13}$C NMR spectrum (101 MHz, Chloroform-*d*) of compound **115**

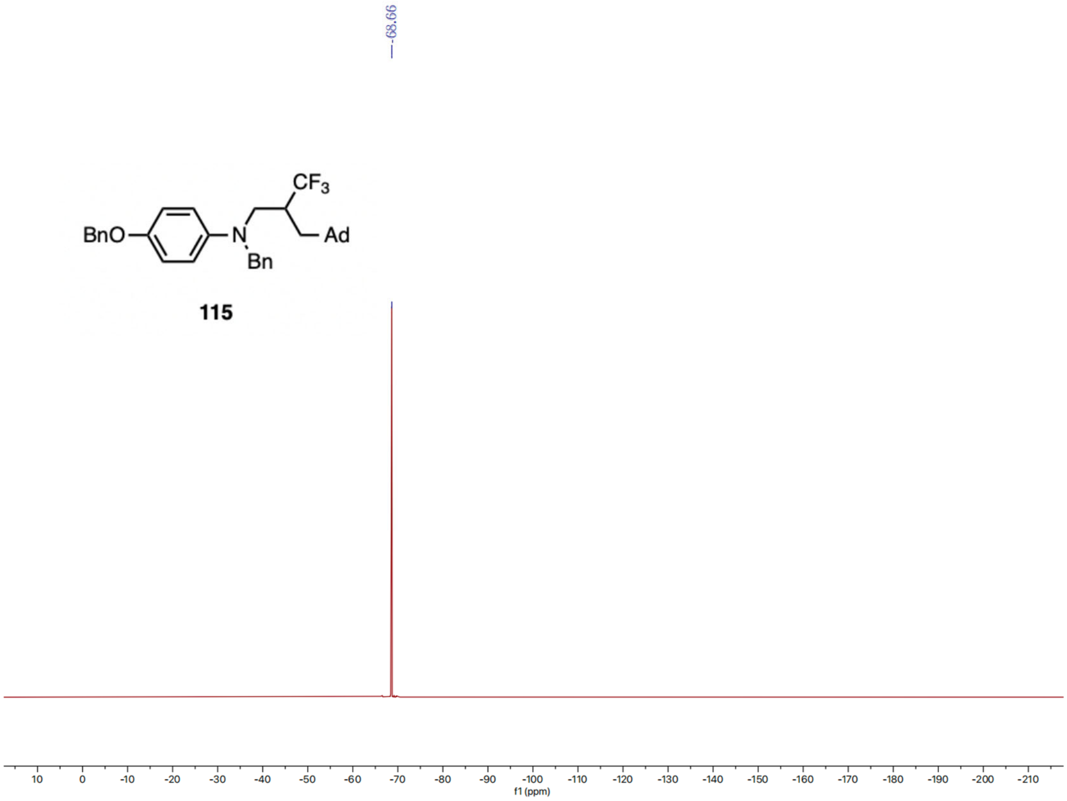


$^{19}$F NMR spectrum (376 MHz, Chloroform-*d*) of compound **115**

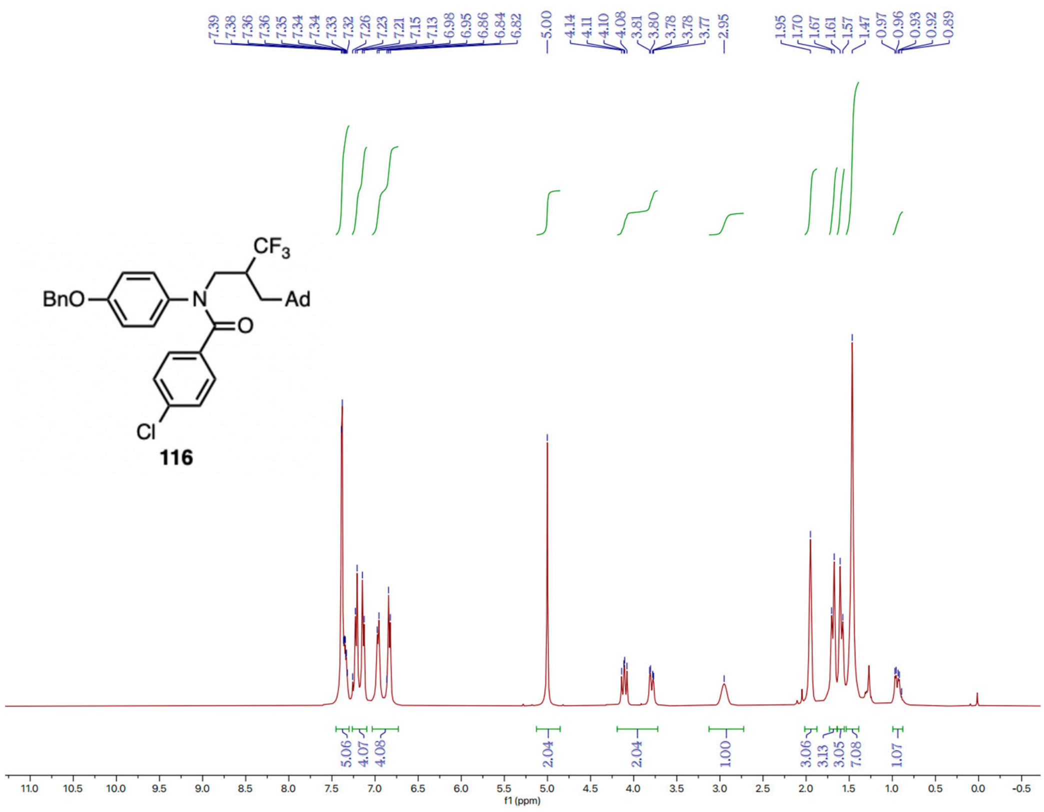

$^{1}$H NMR spectrum (400 MHz, Chloroform-*d*) of compound **116**

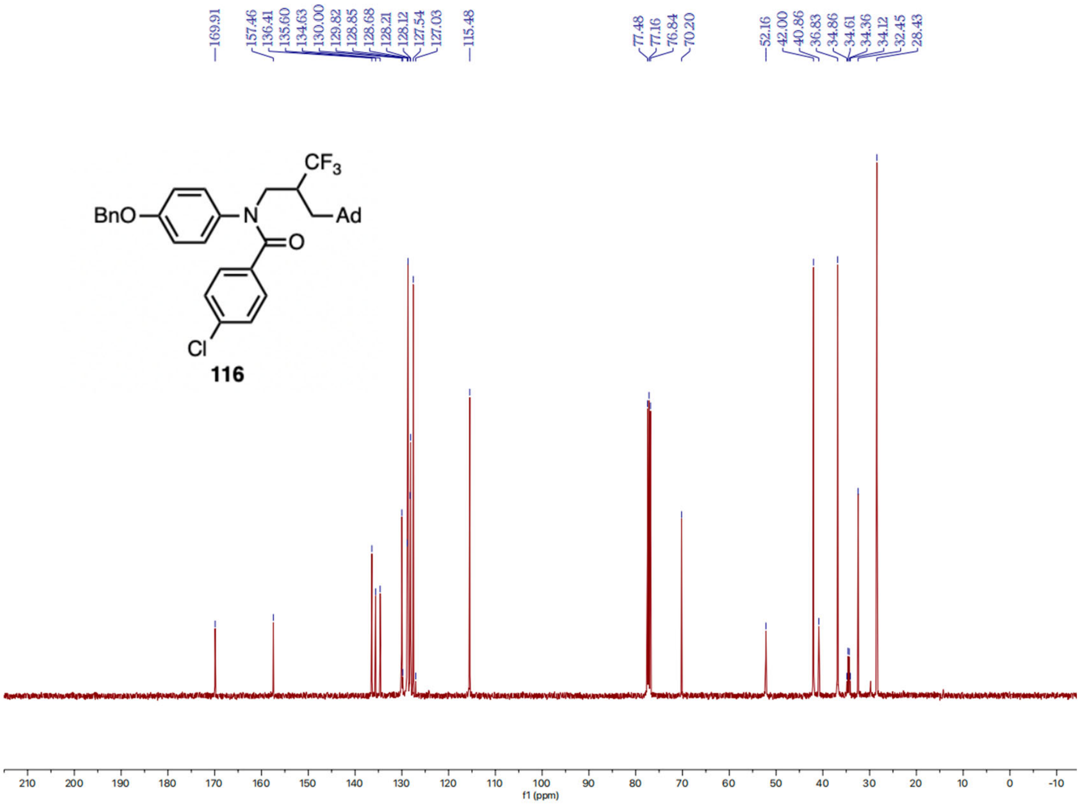

$^{13}$C NMR spectrum (101 MHz, Chloroform-*d*) of compound **116**

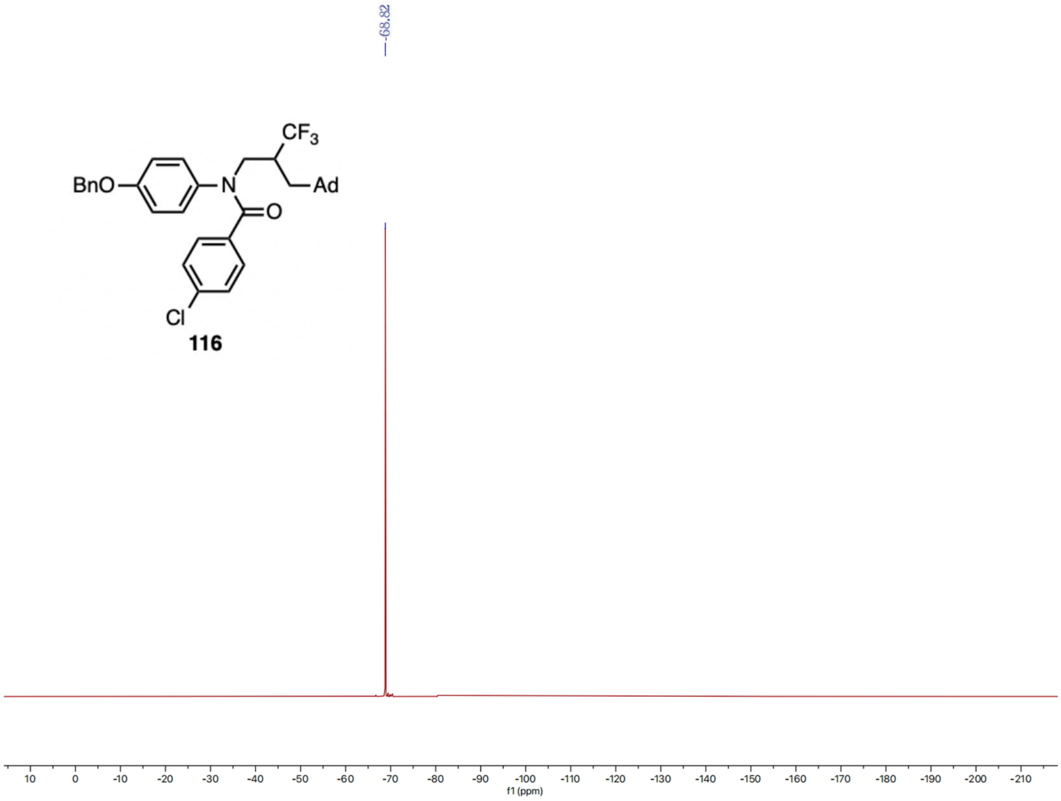


$^{19}$F NMR spectrum (376 MHz, Chloroform-*d*) of compound **116**

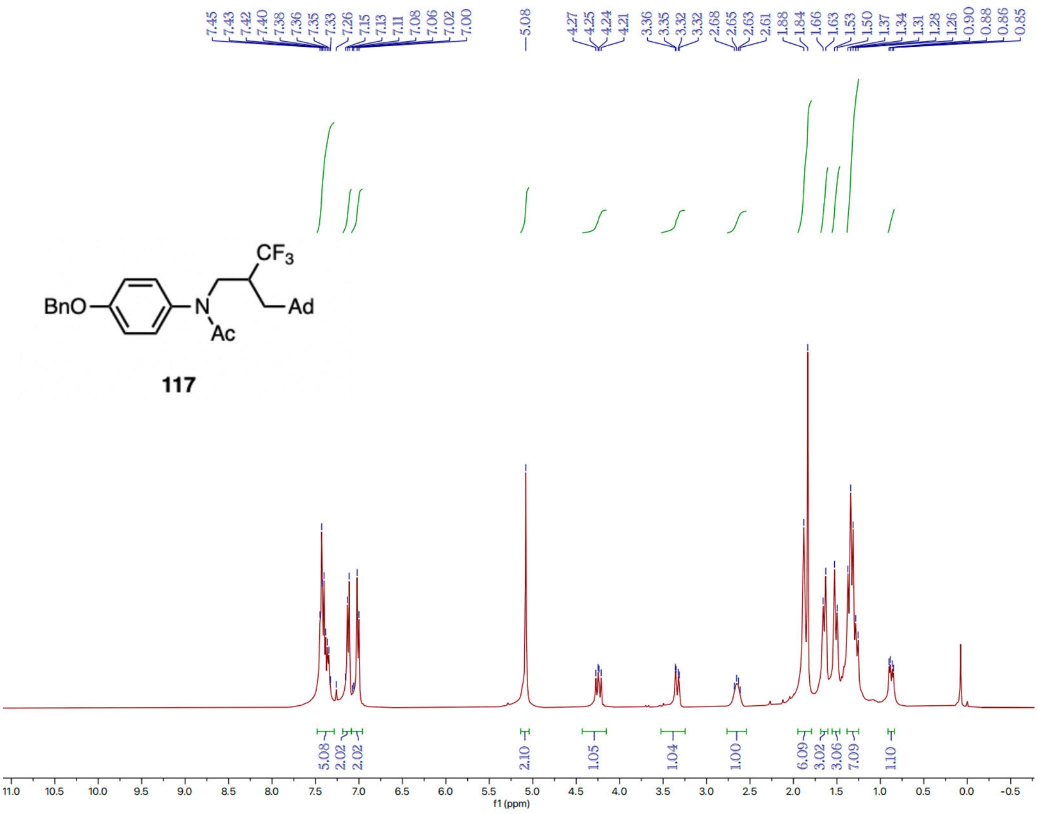

$^{1}$H NMR spectrum (400 MHz, Chloroform-*d*) of compound **117**

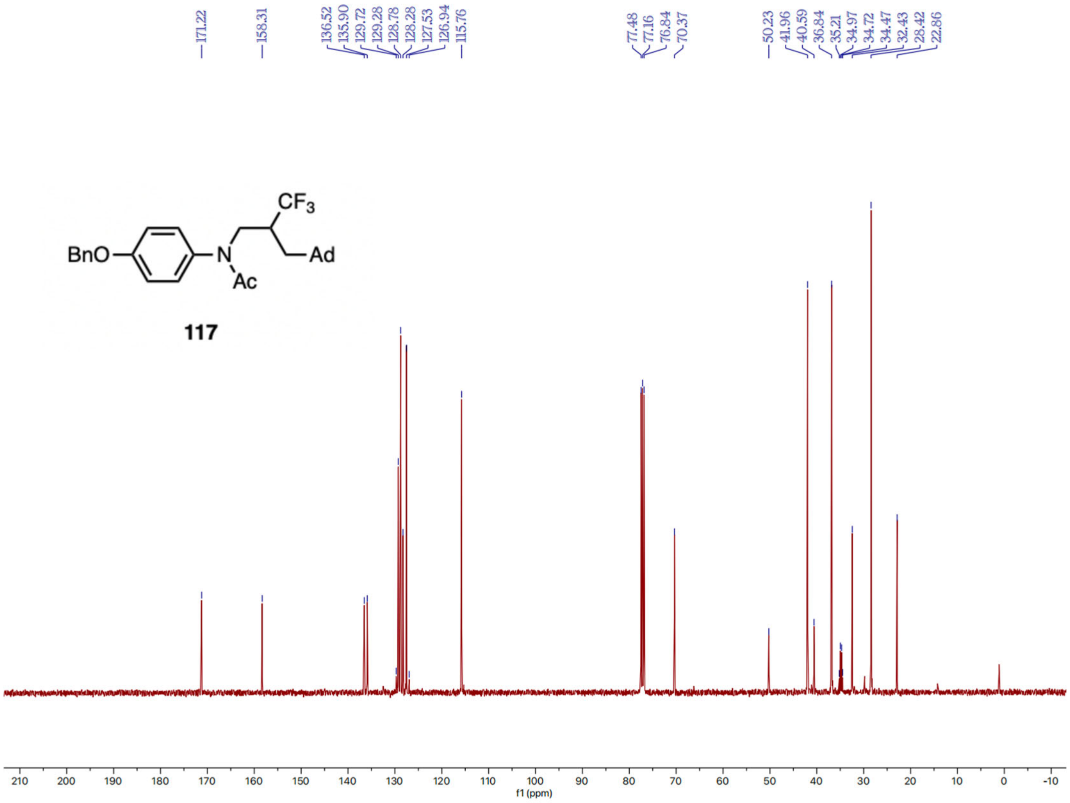

$^{13}$C NMR spectrum (101 MHz, Chloroform-*d*) of compound **117**

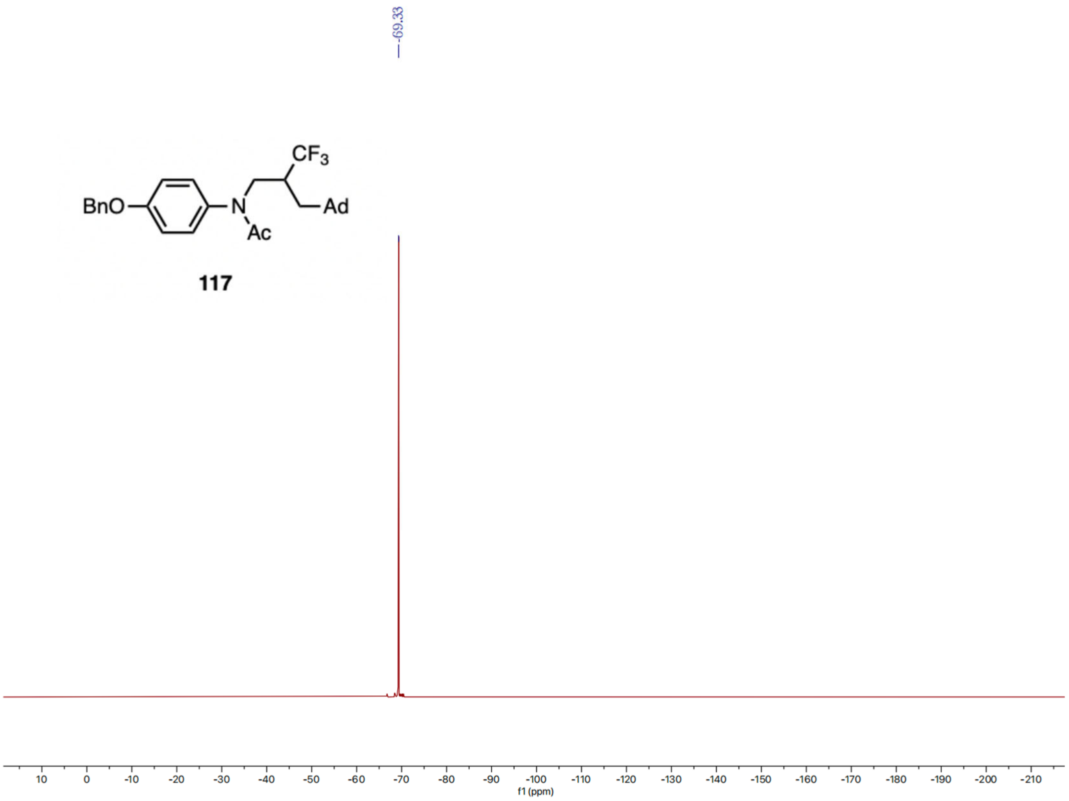


$^{19}$F NMR spectrum (376 MHz, Chloroform-*d*) of compound **117**

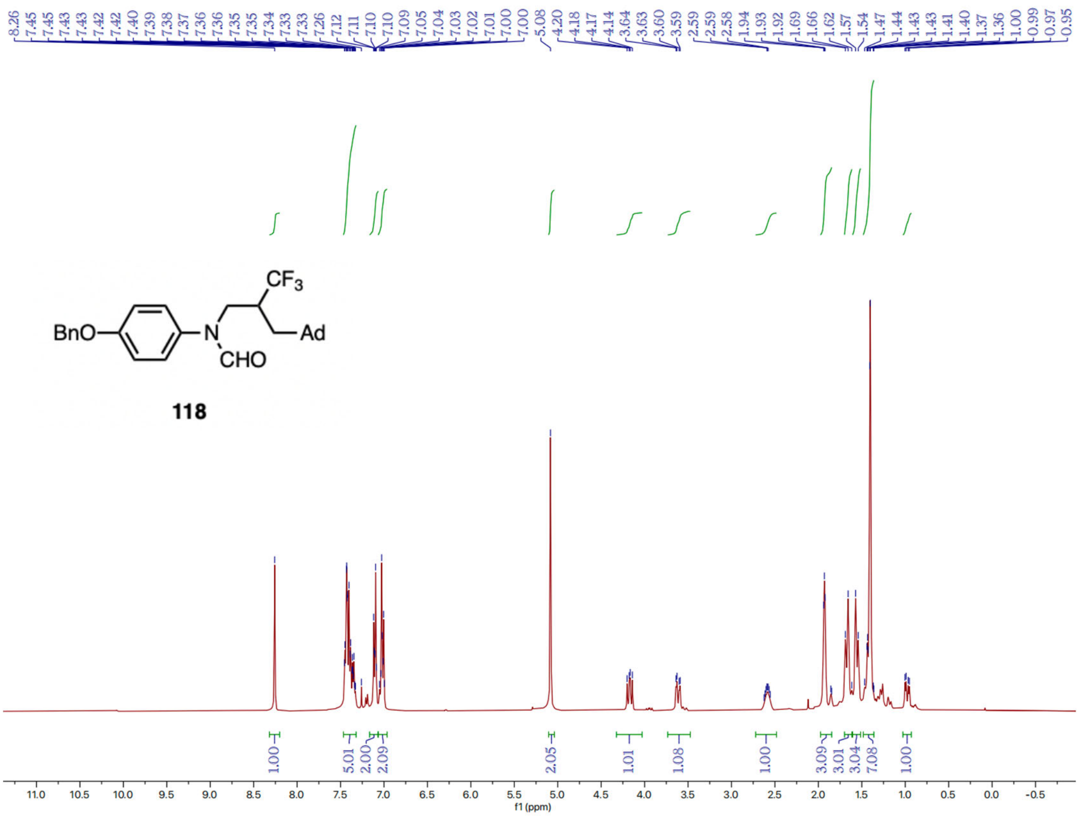

$^{1}$H NMR spectrum (400 MHz, Chloroform-*d*) of compound **118**

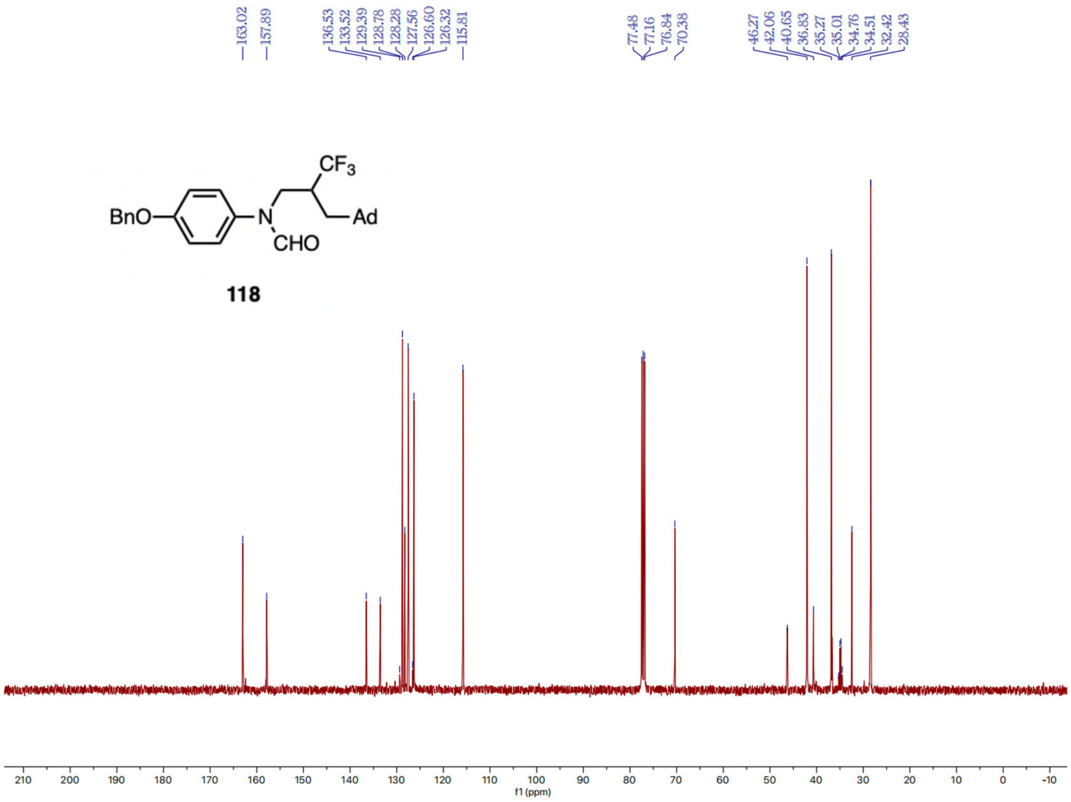

$^{13}$C NMR spectrum (101 MHz, Chloroform-*d*) of compound **118**

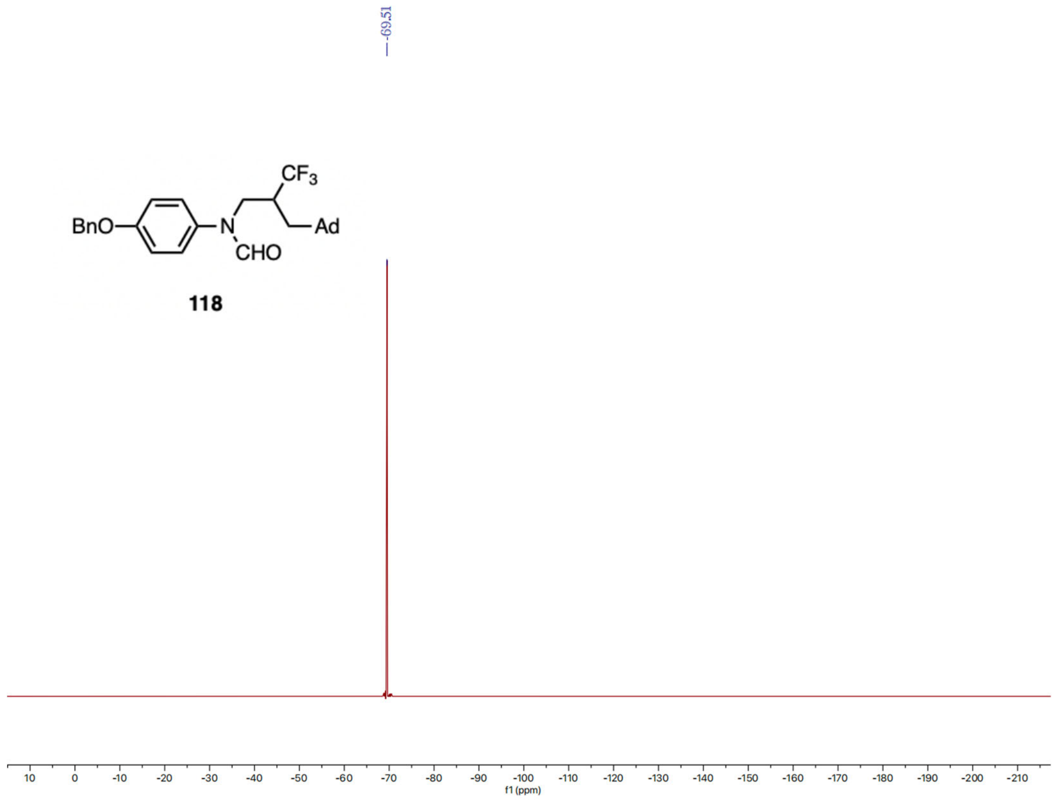


$^{19}$F NMR spectrum (376 MHz, Chloroform-*d*) of compound **118**

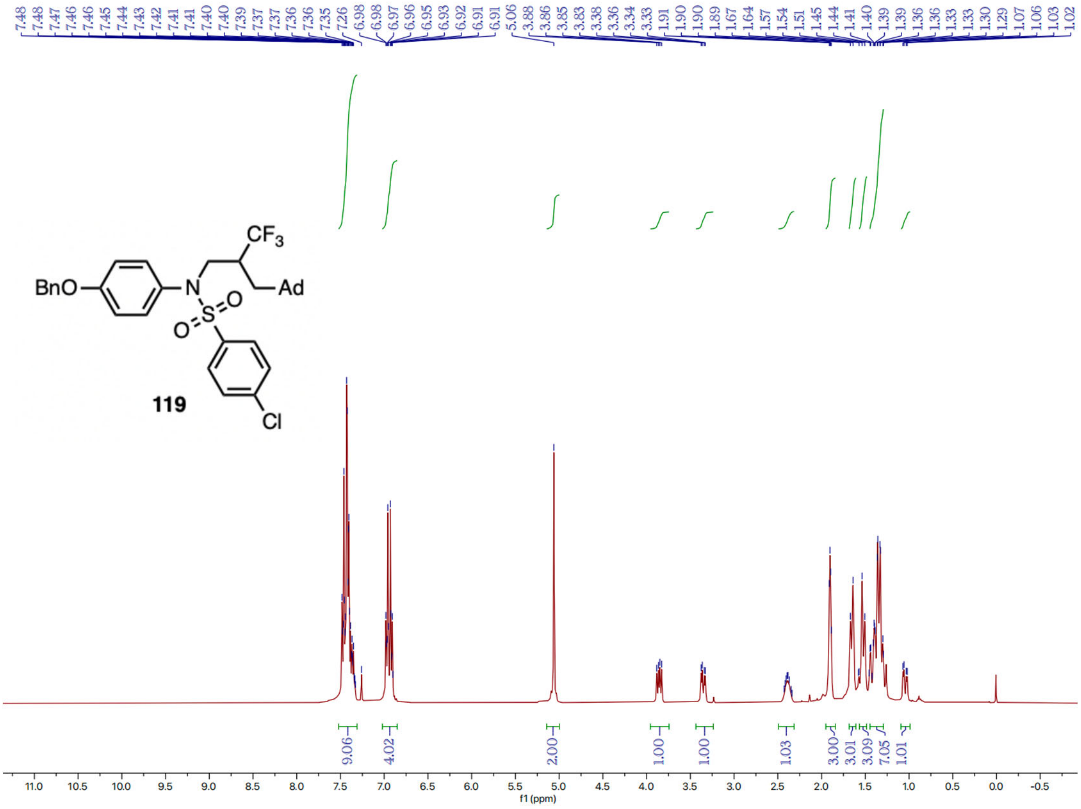


$^{1}$H NMR spectrum (400 MHz, Chloroform-*d*) of compound **119**

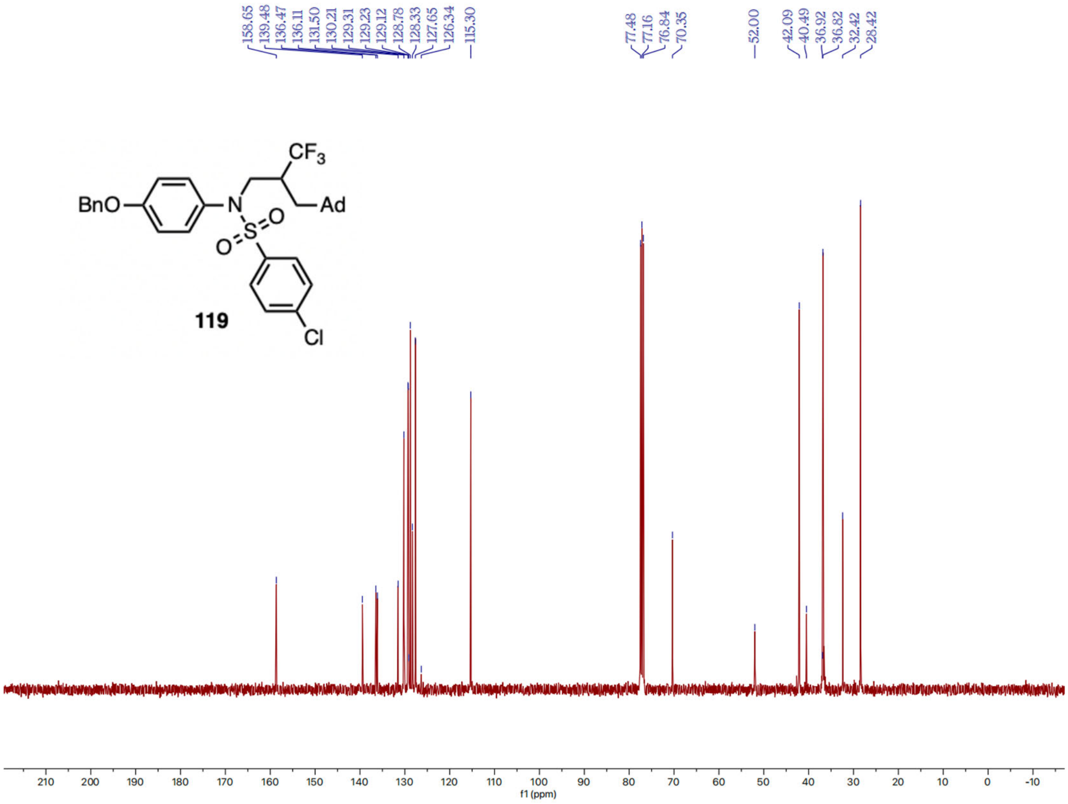


$^{13}$C NMR spectrum (101 MHz, Chloroform-*d*) of compound **119**

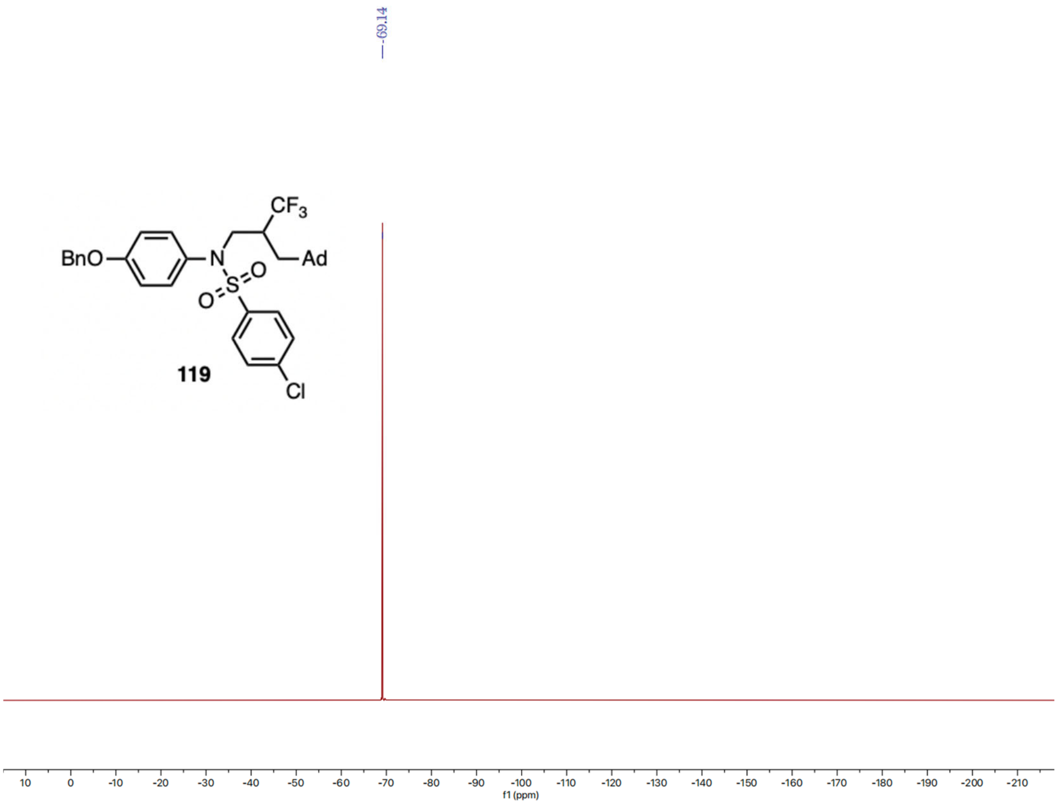


$^{19}$F NMR spectrum (376 MHz, Chloroform-*d*) of compound **119**

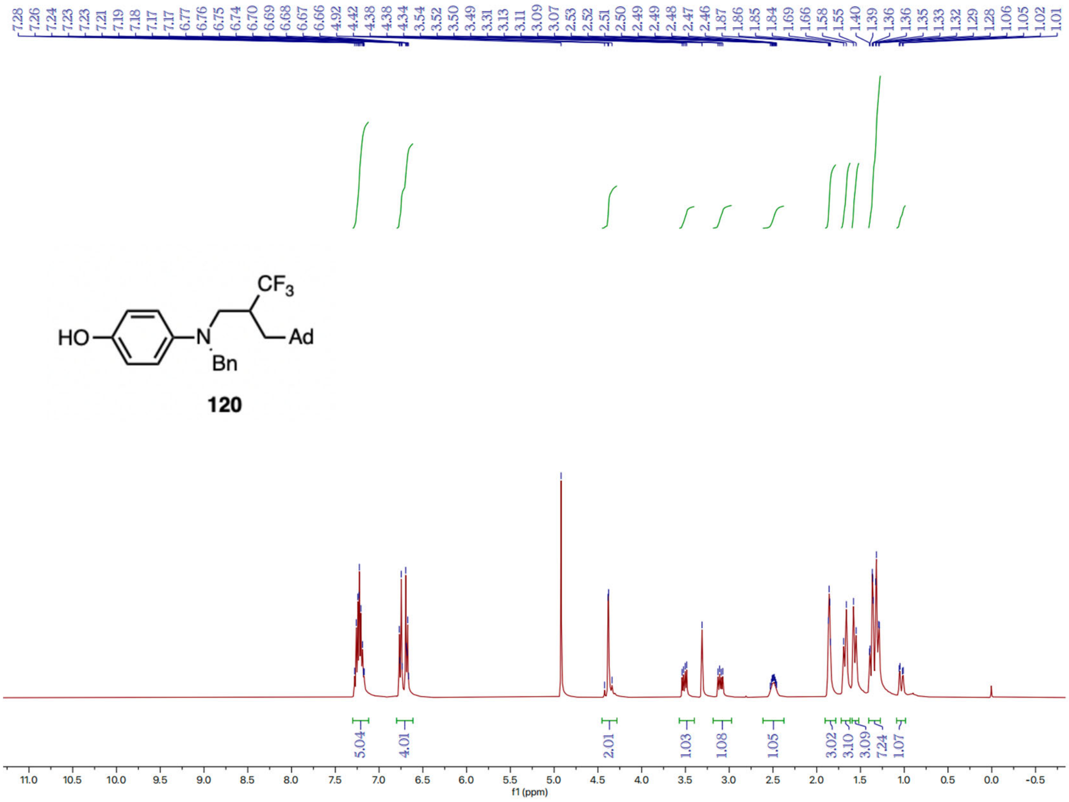


$^{1}$H NMR spectrum (376 MHz, Methanol-$d_4$) of compound **120**

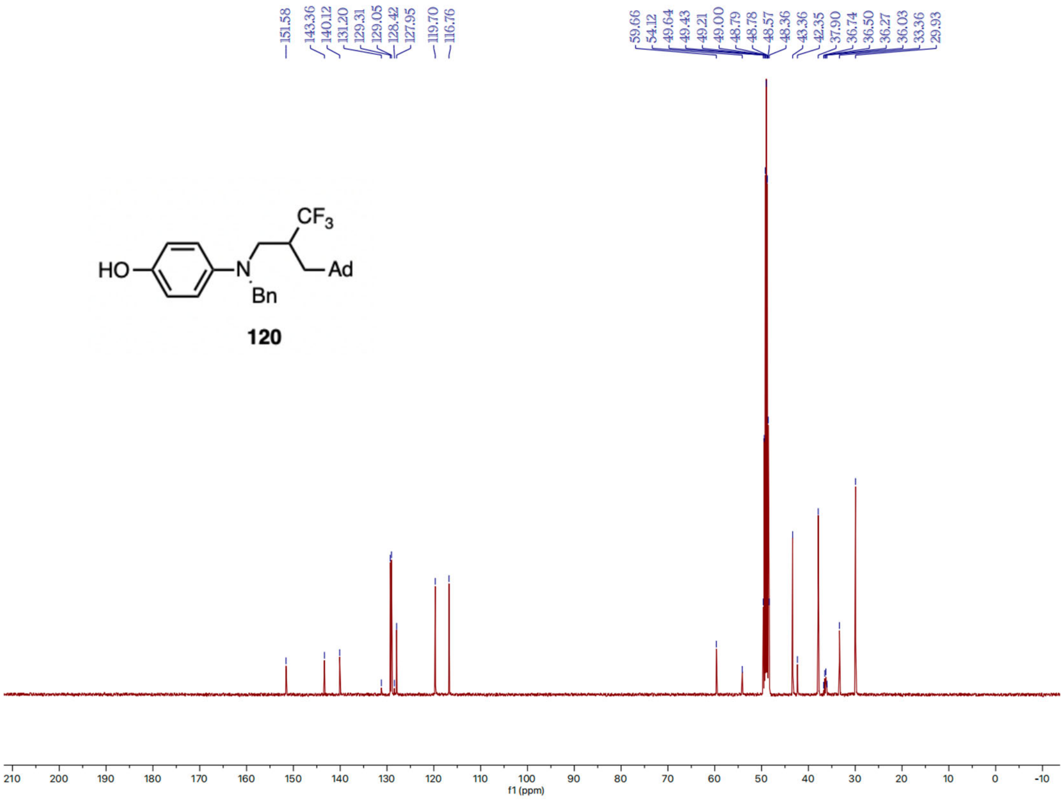


$^{13}$C NMR spectrum (376 MHz, Methanol-$d_4$) of compound **120**

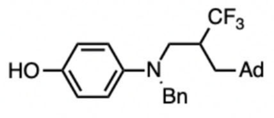


**120**

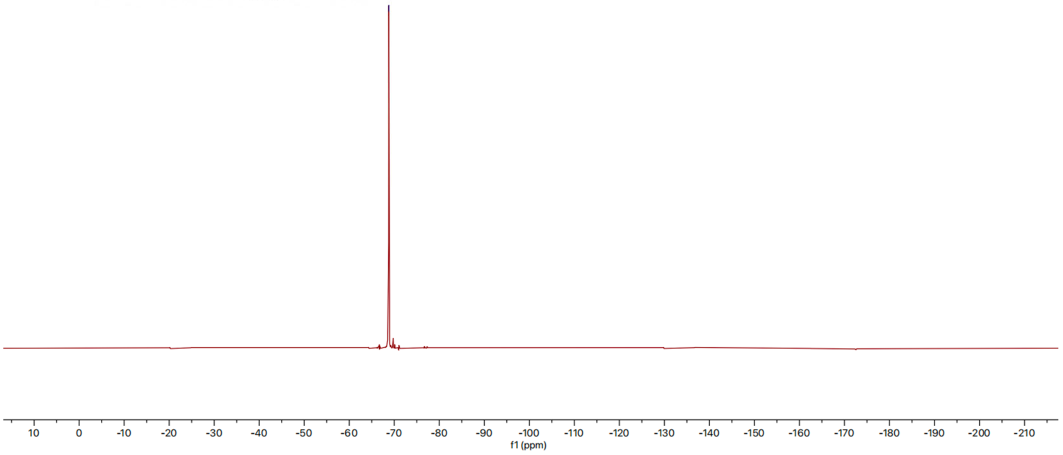


$^{19}$F NMR spectrum (376 MHz, Methanol-*d*$_4$) of compound **120**

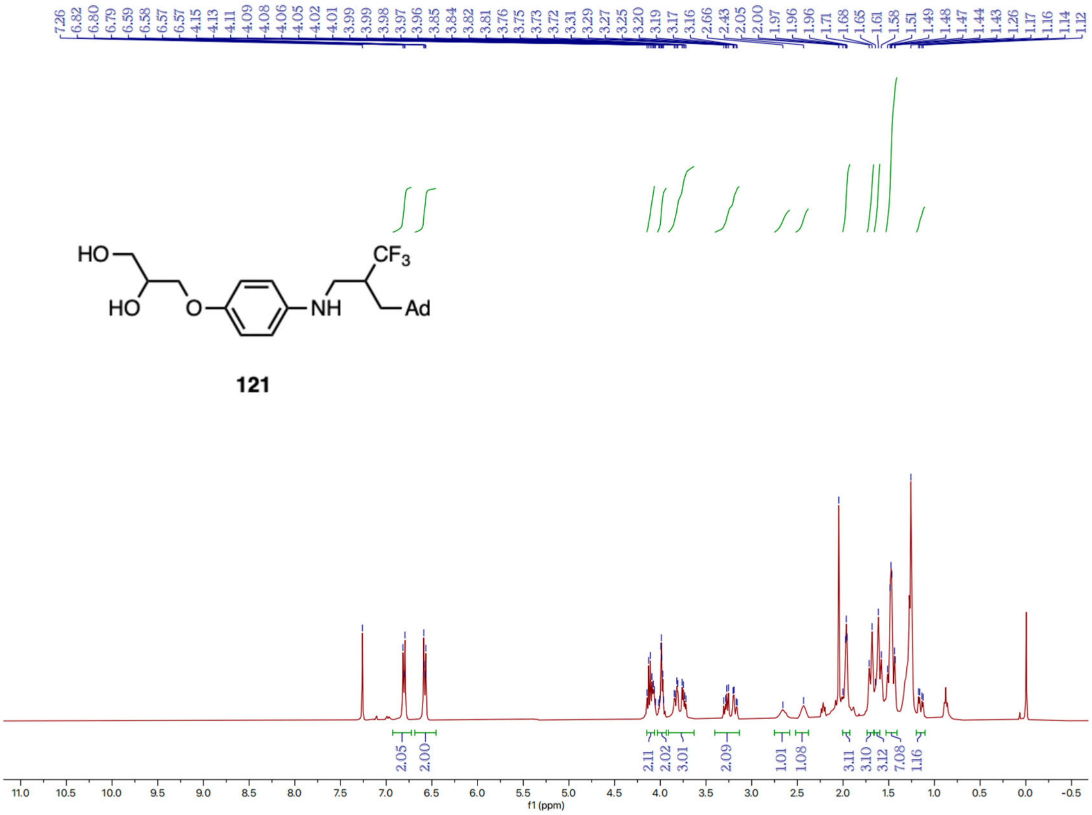

$^{1}$H NMR spectrum (400 MHz, Chloroform-*d*) of compound **121**

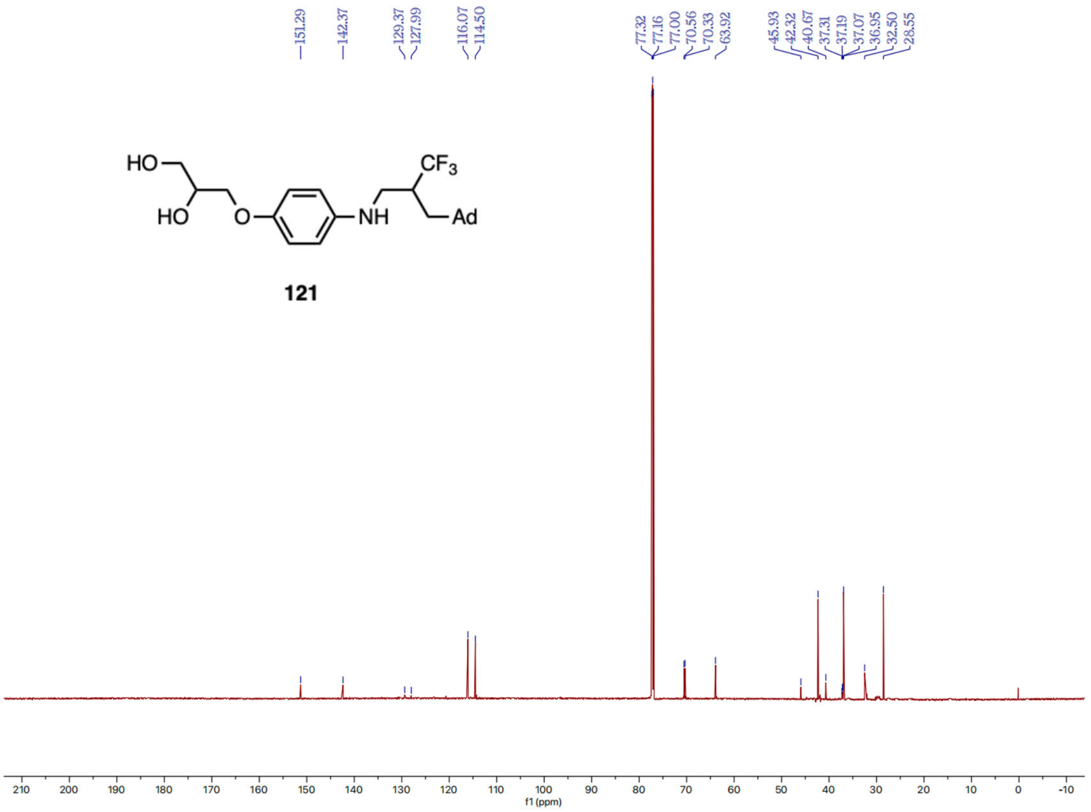

$^{13}$C NMR spectrum (201 MHz, Chloroform-*d*) of compound **121**

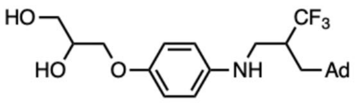



**121**

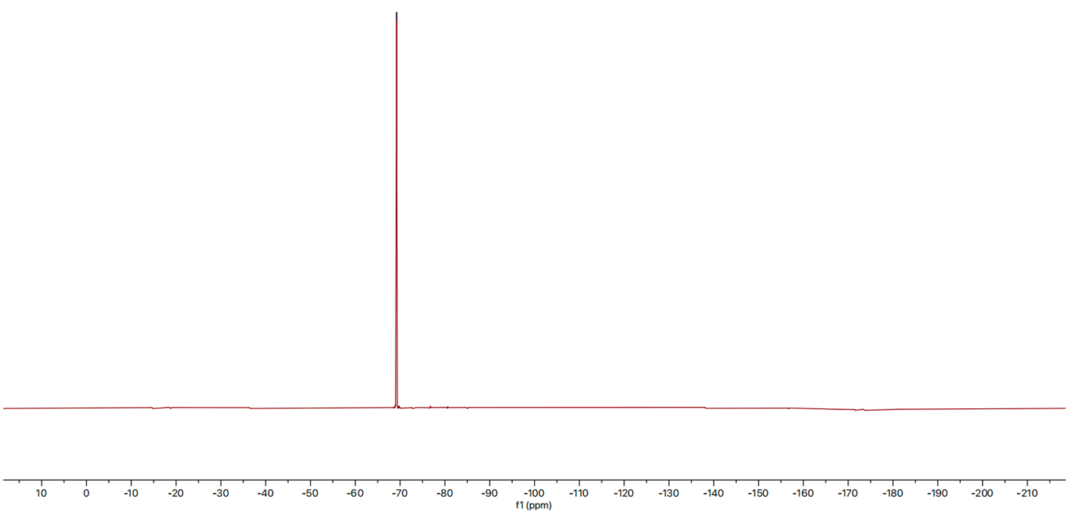



$^{19}$F NMR spectrum (376 MHz, Chloroform-*d*) of compound **121**

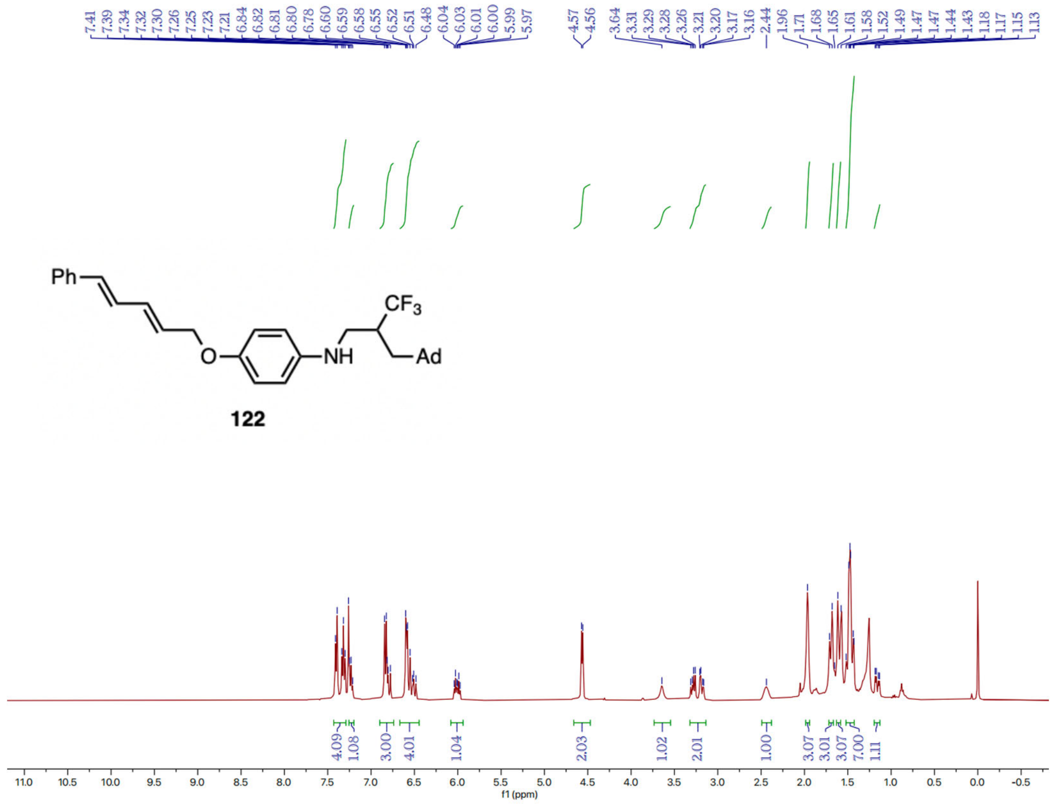

$^1$H NMR spectrum (400 MHz, Chloroform-*d*) of compound **122**

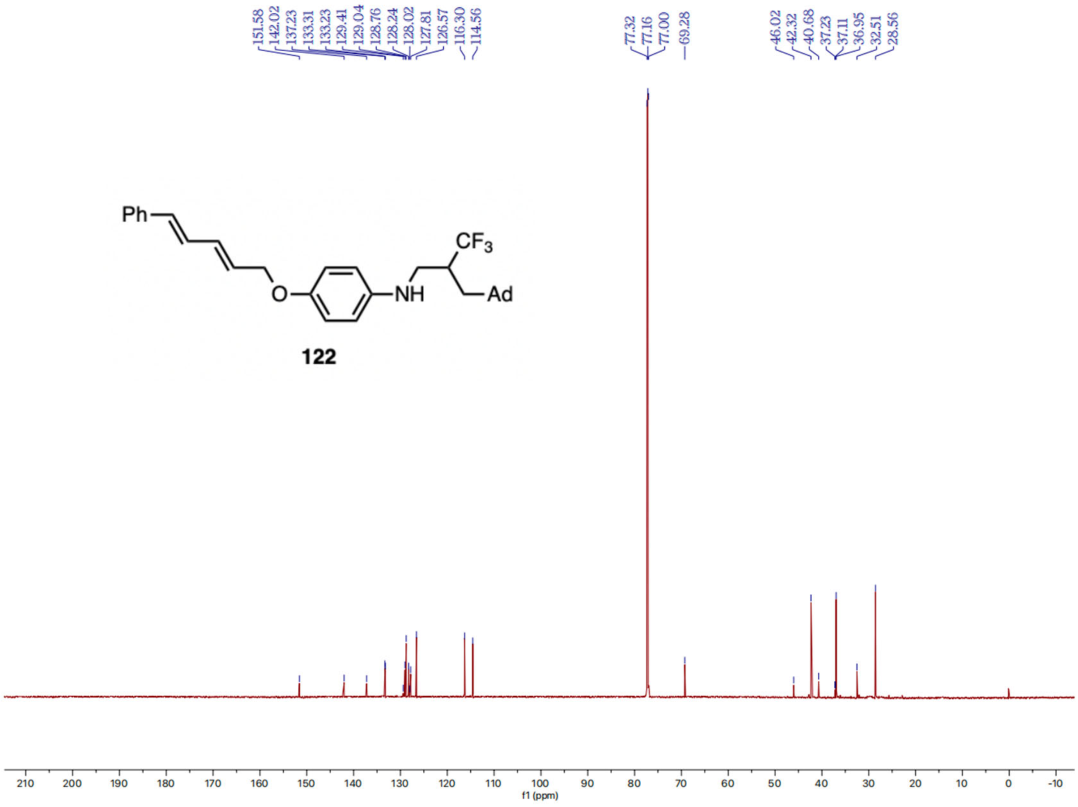

$^{13}$C NMR spectrum (101 MHz, Chloroform-*d*) of compound **122**

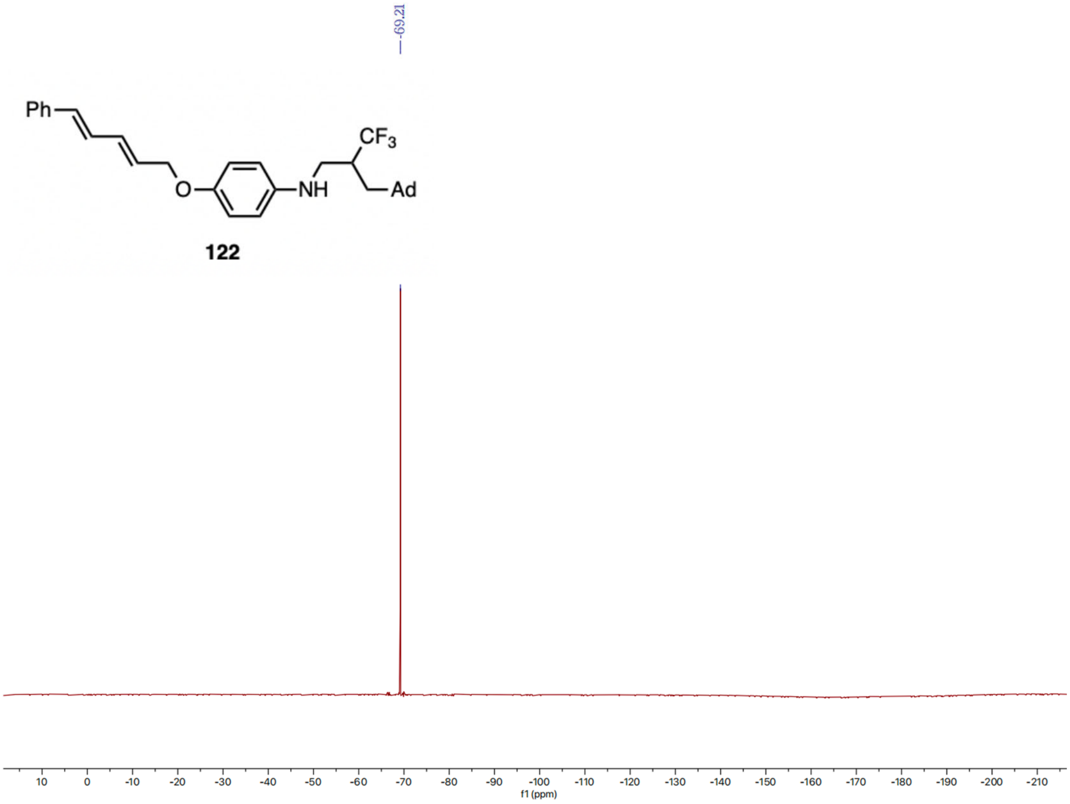


$^{19}$F NMR spectrum (376 MHz, Chloroform-*d*) of compound **122**

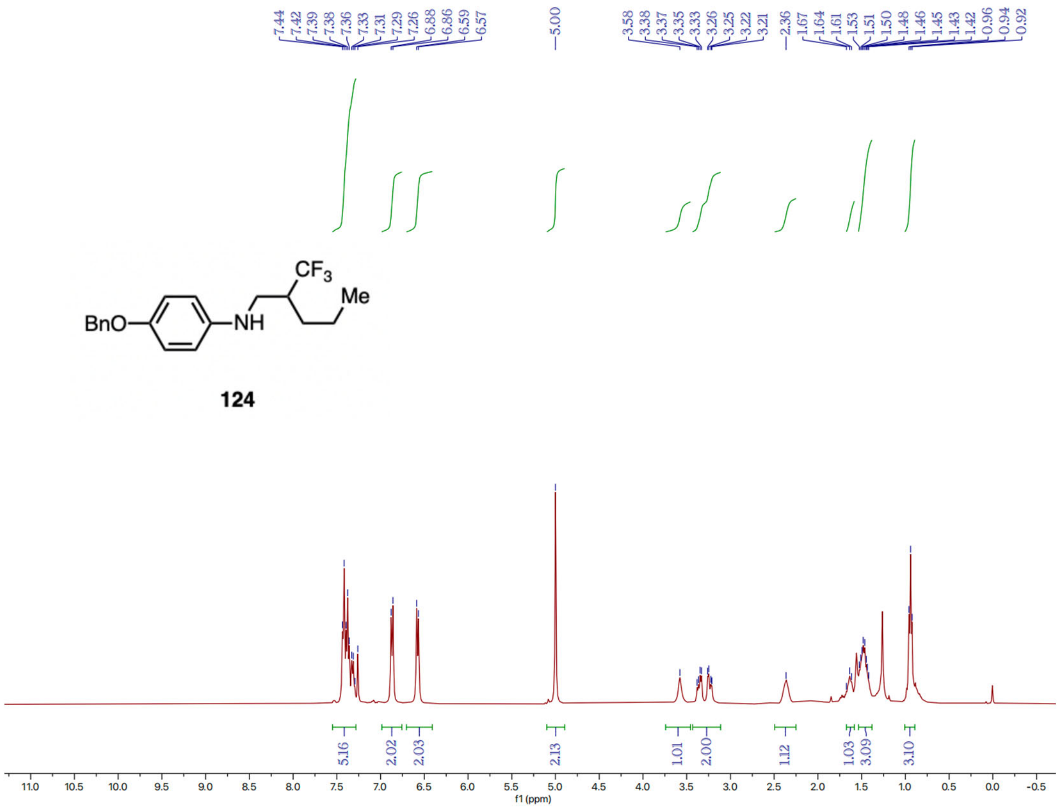


$^{1}$H NMR spectrum (400 MHz, Chloroform-*d*) of compound **124**

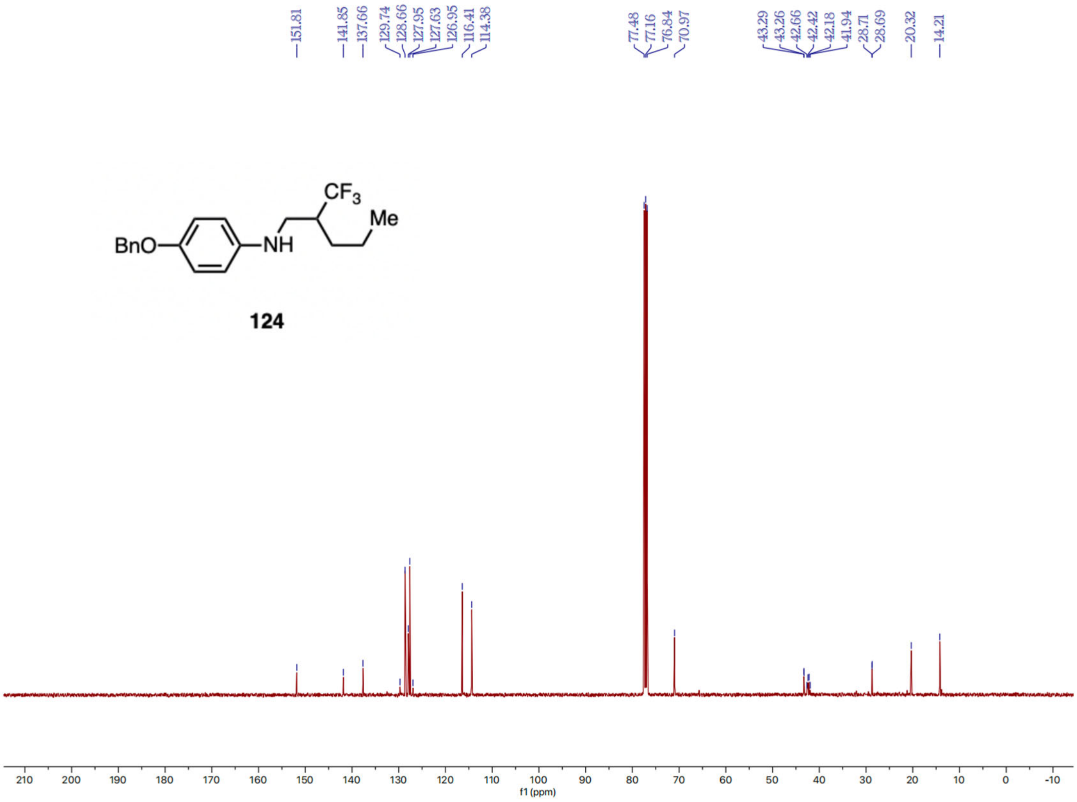


$^{13}$C NMR spectrum (101 MHz, Chloroform-*d*) of compound **124**

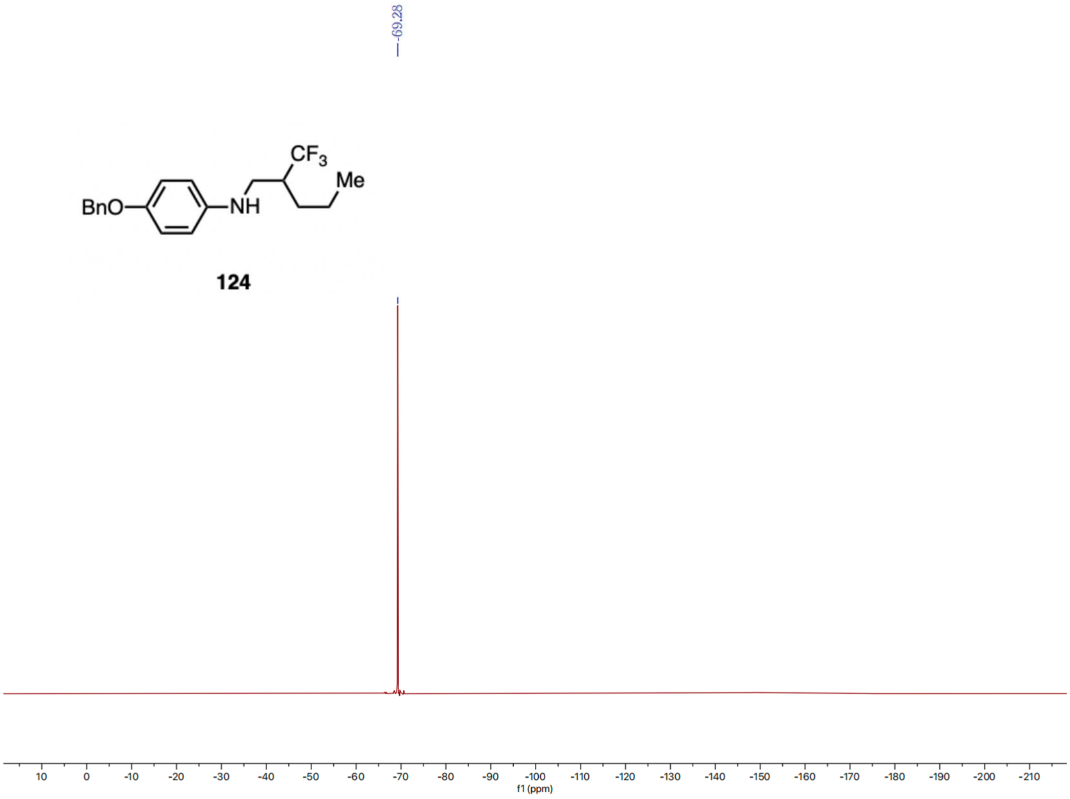


$^{19}$F NMR spectrum (376 MHz, Chloroform-*d*) of compound **124**

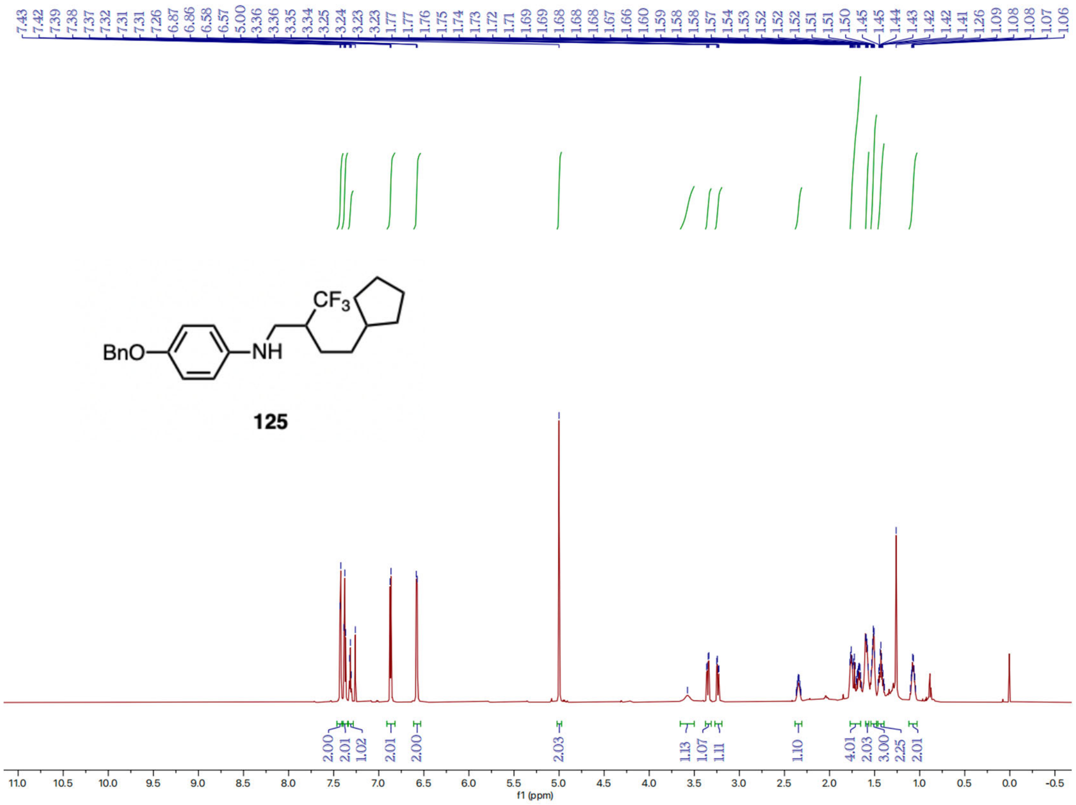

$^1$H NMR spectrum (800 MHz, Chloroform-*d*) of compound **125**

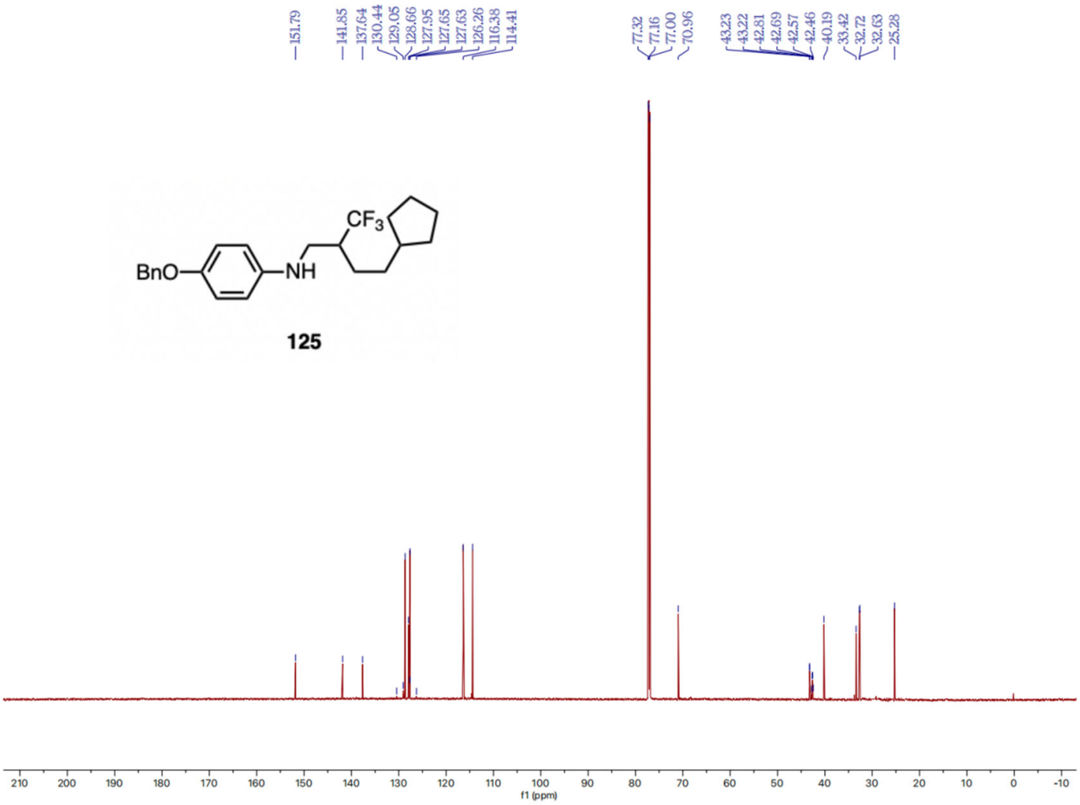

$^{13}$C NMR spectrum (201 MHz, Chloroform-*d*) of compound **125**

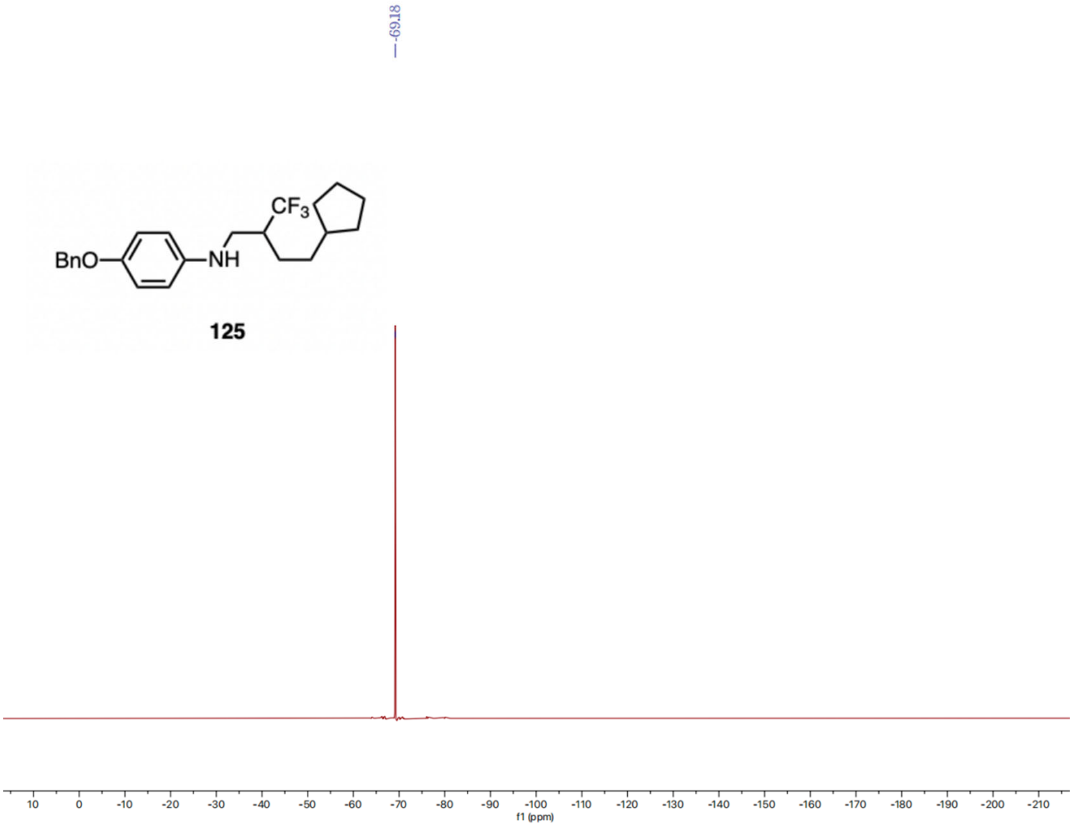


$^{19}$F NMR spectrum (471 MHz, Chloroform-*d*) of compound **125**

# References


[1] Fujii, S., Kikuchi,E., Watanabe, Y., Suzuyama, H. & Kagechika, H. Structural development of *N*-(4-phenoxyphenyl)benzamide derivatives as novel SPAK inhibitors blocking WNK kinase signaling. *Bioorg. Med. Chem. Lett.* **30**, 127408 (2020).

[2] Shaw, R., Sihag, N., Jain, S., Sharma, R. & Yadav, M. R. Photoinduced Alkyl/Aryl Radical Cascade for the Synthesis of Quaternary $CF_3$-Containing Oxindoles and Indoline Alkaloids. *J. Org. Chem.* **88**, 5652–5660 (2023).

[3] Li, G. X., Chen, R., Wu, L., Fu, Q., Zhang, X., & Tang, Z. Alkyl Transfer from C–C Cleavage. *Angew. Chem. Int. Ed.* **52**, 8432−8436 (2013).

[4] Shi, J. B., Wang, Y. B., Bu, Q. Q., Liu, B. Y., Dai, B. & Liu, N. Cr-Catalyzed Direct *ortho*-Aminomethylation of Phenols. *J. Org. Chem.* **86**, 17567−17580 (2021).

[5] De, S., Ghosh, S., Bhunia, S., Sheikh, J. A. & Bisai, A. Intramolecular direct dehydrohalide coupling promoted by kotbu: total synthesis of amaryllidaceae alkaloids anhydrolycorinone and oxoassoanine. *Org. Lett.* **14**, 4466–4469 (2012).

[6] Yi, X. W., Lei, S. Y., Liu, W. S., Che, F. R., Yu, C. Z., Liu, X. S., Wang, Z. H., Zhou, X. & Zhang, Y. X. Copper-Catalyzed Radical *N*-Demethylation of Amides Using *N*-Fluorobenzenesulfonimide as an Oxidant. *Org. Lett.* **22**, 4583–4587 (2020).

[7] Wang, Y. X., Zhang, F. P., Luan, Y. X. & Ye, M. Ligand-enabled Ni–Al bimetallic catalysis for nonchelated dual C–H annulation of arylformamides and alkynes. *Org. Lett.* **22**, 2230–2234 (2020).

[8] Pillaiyar, T., Rosato, F., Wozniak, M., Blavier, J., Charles, M., & Laschet, C., Structure-activity relationships of agonists for the orphan G protein-coupled receptor GPR27. *Eur. J. Med. Chem.* **225**, 113777 (2021).

[9] Kim, S. H., Kwon, J. H. & Yoon, S. H. An improved synthesis of 4'-hydroxydiclofenac. *Bull. Korean Chem. Soc.* **31**, 3007–3009 (2010).

[10] Sheng, T., Kang, G. W., Zhuang, Z., Chekshin, N., Wang, Z., Hu, L. & Yu, J. Q. Synthesis of *β*,*γ*-Unsaturated Aliphatic Acids via Ligand-Enabled Dehydrogenation. *J. Am. Chem. Soc.* **145**, 20951–20958 (2023).

[11] Zeng, H., Zhu, C. & Jiang, H. Single C($sp^3$)–F bond activation in a $CF_3$ group: *ipso*-defluorooxylation of (trifluoromethyl)alkenes with oximes. *Org. Lett.* **21**, 1130–1133 (2019).

[12] Xiao, F. S., Chen, F., Wang, S. & Sun, Q. Turning on catalysis: construction of triphenylphosphine moieties into porous frameworks. *ChemCatChem*. **12**, 3285–3289 (2020).

[13] Cao, W. D., Zhang, X. H., & Bard, A. J. Electrogenerated chemiluminescence. 75. Electrochemistry and ECL of 9,10-bis(2-naphthyl)anthracene. *J. Electroanal. Chem.* **566**, 409–413 (2004).
